# Hidden diversity in *Enterococcus faecalis* revealed by CRISPR2 screening: eco-evolutionary insights into a novel subspecies

**DOI:** 10.1128/spectrum.01428-25

**Published:** 2025-09-08

**Authors:** Vitor Luis Macena Leite, Adriana Rocha Faria, Clara Ferreira Guerra, Stephanie da Silva Rodrigues Souza, Andréa de Andrade Rangel Freitas, Jaqueline Martins Morais, Vânia Lúcia Carreira Merquior, Paul J. Planet, Lúcia Martins Teixeira

**Affiliations:** 1 Instituto de Microbiologia Paulo de Góes, Universidade Federal do Rio de Janeiro 28125, Rio de Janeiro, Rio de Janeiro, Brazil; 2 Faculdade de Medicina, Universidade Federal do Rio de Janeiro 28130, Rio de Janeiro, Rio de Janeiro, Brazil; 3 Department of Biological Sciences, University at Albany, State University of New York 1084, Albany, New York, USA; 4 Faculdade de Ciências Médicas, Universidade do Estado do Rio de Janeiro 28130, Rio de Janeiro, Rio de Janeiro, Brazil; 5 Perelman School of Medicine, University of Pennsylvania 6572, Philadelphia, Pennsylvania, USA; 6 Pediatric Infectious Disease Division, Children's Hospital of Philadelphia 6567, Philadelphia, Pennsylvania, USA; 7 Institute for Comparative Genomics, American Museum of Natural History 5963, New York, New York, USA; University of Florida College of Dentistry, Gainesville, Florida, USA

**Keywords:** subspecies separation, bacterial evolution, genomic epidemiology, one health, CRISPR-Cas, *Enterococcus faecalis*

## Abstract

*Enterococcus faecalis* is a commensal bacterium that colonizes the gut of humans and animals and is a major opportunistic pathogen, known for causing multidrug-resistant healthcare-associated infections (HAIs). Its ability to thrive in diverse environments and disseminate antimicrobial resistance genes (ARGs) across ecological niches highlights the importance of understanding its ecological, evolutionary, and epidemiological dynamics. The CRISPR2 locus has been used as a valuable marker for assessing clonality and phylogenetic relationships in *E. faecalis*. In this study, we identified a group of *E. faecalis* strains lacking CRISPR2, forming a distinct, well-supported clade. We demonstrate that this clade meets the genomic criteria for classification as a novel subspecies, here referred to as “subspecies B.” Through a comprehensive pangenome analysis and comparative genomics, we explored the adaptive ecological traits underlying this diversification process, identifying clade-specific features and their predicted functional roles. Our findings suggest that the frequent isolation of subspecies B from meat products and processing facilities may reflect dissemination routes involving environmental contamination (e.g., water, plants, soil) from avian species. The absence of key virulence traits required for pathogenicity in mammals, particularly humans, and the lack of clinically relevant resistance determinants indicate that subspecies B currently poses minimal threat to public health compared with the broadly disseminated “subspecies A.” Nevertheless, the unclear potential for genetic exchange between these subspecies and the frequent association of subspecies B with food sources calls for continued genomic surveillance of *E. faecalis* from a One Health perspective to detect and mitigate the emergence of high-risk variants in advance.

**IMPORTANCE:**

Exploring intraspecific genetic variability in generalist bacteria with pathogenic potential, such as *Enterococcus faecalis*, is a key to uncovering stable evolutionary trends. By screening the CRISPR2 locus across a representative set of genomes from diverse sources, this study reveals a previously unrecognized lineage within the population structure of *E. faecalis*, associated with underexplored nonhuman and nonhospital reservoirs. These findings broaden our knowledge of the species' genetic landscape and shed light on its adaptive strategies and patterns of ecological dissemination. By bridging phylogenetic patterns with variation in genetic defense systems and accessory traits, the study generates testable hypotheses about the genomic determinants and corresponding selective pressures that shape the species’ behavior and long-term dissemination. This work offers new perspectives on the eco-evolutionary dynamics of *E. faecalis* and highlights the value of genomic surveillance beyond clinical settings, in alignment with One Health principles.

## INTRODUCTION

*Enterococcus faecalis* is a gram-positive commensal bacterium that colonizes the gastrointestinal tract (GIT) of humans and other animals (1). Its broad distribution, extending to soil, water, and other natural sources (2), reflects its exceptional tolerance to adverse environmental conditions, including variations in temperature, pH, and exposure to antimicrobials (3, 4). As an opportunistic pathogen, *E. faecalis* is a major cause of healthcare-associated infections (HAIs) globally, mainly due to its remarkable ability to acquire multiple antimicrobial resistance genes (ARGs) through horizontal gene transfer (HGT), hampering treatment outcomes (5, 6).

A key factor in the genomic plasticity of *E. faecalis* is its ability to accumulate mobile genetic elements (MGEs), a process counterbalanced by Clustered Regularly Interspaced Short Palindromic Repeats (CRISPR) and their associated Cas proteins (CRISPR-Cas) (5). The CRISPR-Cas system functions as a bacterial adaptive immune mechanism, protecting against bacteriophage infections and limiting the uptake of exogenous DNA, including plasmids and other MGEs (7). The system’s activity relies on two core components: (i) the CRISPR array, composed of alternating direct repeats and short spacer sequences derived from foreign DNA, and (ii) Cas proteins, which mediate the recognition and cleavage of invading genetic material (7).

Three CRISPR loci have been described in *E. faecalis*: CRISPR1-Cas and CRISPR3-Cas (predicted to be functional), and CRISPR2, an orphan locus lacking *cas* genes (5). Despite being inactive as a defense system, CRISPR2 is highly conserved across *E. faecalis* genomes and considered part of the species’ core genome (8). Its maintenance raises questions about functional relevance, possibly as a regulatory noncoding RNA, a role supported by transcriptomic evidence, although not yet confirmed (9–11).

The variability of CRISPR2 spacer sequences has been used to assess *E. faecalis* genetic diversity, proposed as a useful marker for strain typing, especially in studies with limited resources where whole-genome sequencing is not feasible (12–14). When combined with multilocus sequence typing (MLST), CRISPR2 typing can provide enhanced phylogenetic resolution (12). However, potential associations between unique CRISPR2 signatures and ecological diversification within closely related *E. faecalis* strains remain unexplored.

Unlike *E. faecium*, population structure studies in *E. faecalis* have not identified well-defined specialist groups (6, 15). However, certain sequence types (STs) are part of high-risk enterococcal clonal complexes (HiRECCs) associated with a significant burden of HAIs and multidrug resistance (MDR), including vancomycin-resistant enterococci (VRE). These include ST2 and ST6 (CC2/6), ST9 (CC9), ST28 and ST87 (CC28/87), and ST103 (CC388), along with related variants (14, 16, 17). Although these lineages are largely confined to hospital settings, other STs (such as ST4, ST16, ST21, and ST40) exhibit broad ecological distribution and are frequently detected in diverse non-clinical environments (6, 14, 15, 18, 19).

Studies comprising isolates from a range of environments, host species, and geographical regions will deepen our understanding of the ecological and evolutionary dynamics shaping *E. faecalis* diversity (20). Thus, it is extremely worthwhile to explore inexpensive and feasible approaches providing complementary resolution to conventional typing methods (e.g., MLST), enabling the discrimination of subpopulations in early stages of ecological differentiation, which tend to be indistinguishable by multilocus sequence analyses targeting housekeeping genes (21, 22).

Initially, this study aimed to explore CRISPR2 sequence variability across a comprehensive data set of *E. faecalis* genomes representing multiple STs and diverse isolation sources. However, an unexpected finding shifted the focus of our research. A subset of isolates from genetically related STs was found to completely lack CRISPR2. This prompted us to reframe the study with two main objectives. First, we aimed to validate whether this CRISPR2-negative group constitutes a cohesive unit of bacterial diversity, distinct both genetically and ecologically, while also clarifying its taxonomic and phylogenetic position in relation to well-characterized *E. faecalis* lineages. Second, through comparative pangenomic analysis, we sought to identify the differential genetic content between both groups and discuss potential selective pressures driving cladogenesis within *E. faecalis*.

Intraspecific subdivisions in other bacterial species have been shown to carry important ecological and epidemiological implications. For example, nearly all *Salmonella enterica* infections in humans and other warm-blooded animals are caused by subspecies *enterica* (23, 24). Although the remaining subspecies are primarily isolated from cold-blooded hosts, they harbor unique putative virulence factors and may act as reservoirs of genetic diversity for the broader *S. enterica* population (23, 24).

In light of this, the genome-based approach conducted here offers novel insights into the population structure and niche breadth of *E. faecalis*, identifying key areas for future studies on the evolutionary forces shaping its genome.

## RESULTS

### Detection of *E. faecalis* strains lacking CRISPR2: genetic diversity, isolation sources, and CRISPR system profiles

**Fig 1:**
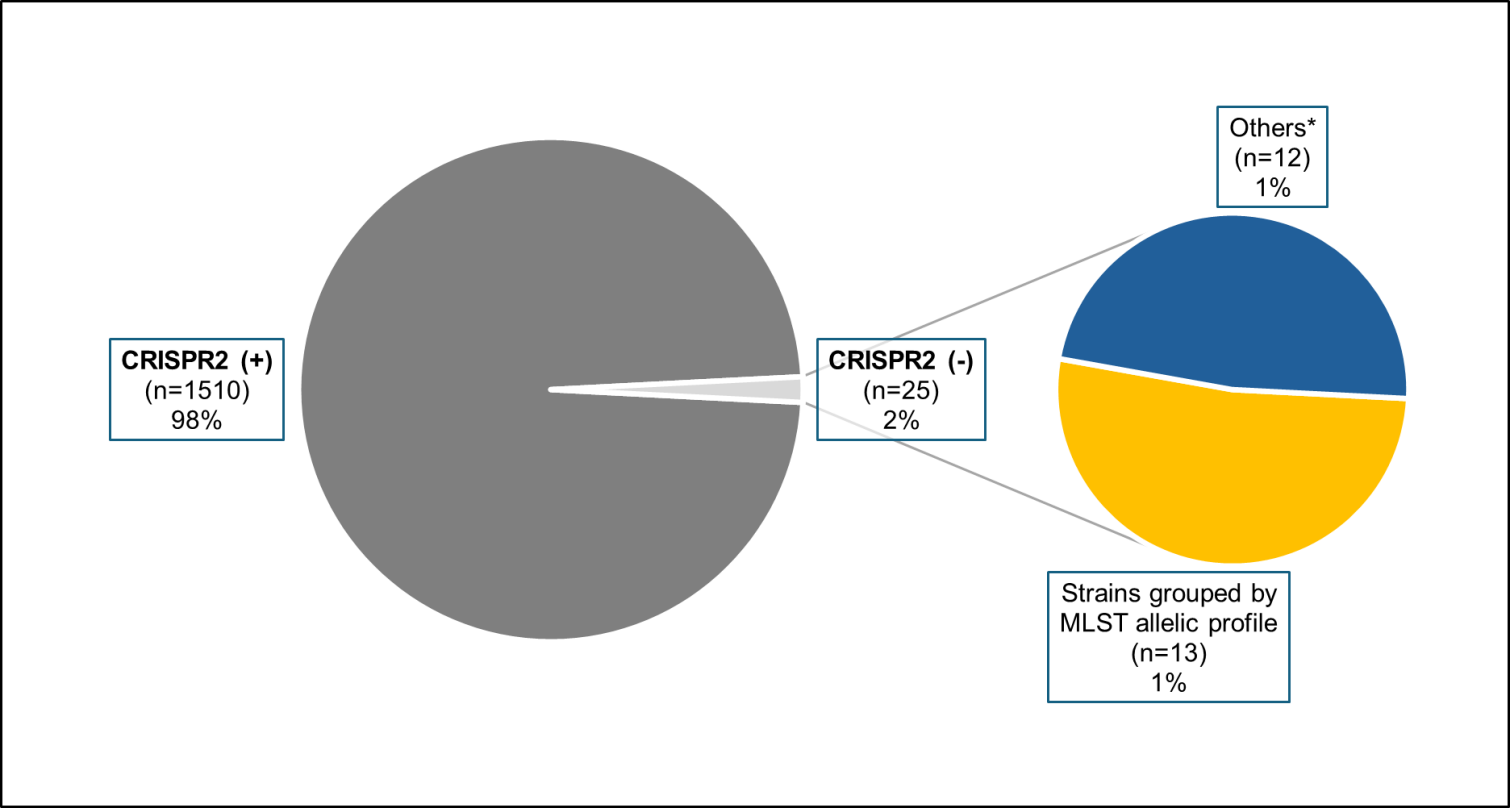
CRISPR2 status in *E. faecalis* genomic samples. The main pie chart categorizes the total genomes (*n* = 1,535) into CRISPR2-positive and CRISPR2-negative. The secondary pie chart further classifies CRISPR2-negative genomes into MLST allelic profile-related strains and others. *The category “others” includes genomes with no evident close relationship within the group based on MLST allelic profiles; genomes with statistics indicating low-quality sequencing or assembly; and genomes removed from Ref-Seq due to various inconsistencies.

We detected the presence of CRISPR2 loci in the vast majority of *E. faecalis* strains analyzed in this study (Fig. 1). All 71 draft genomes from our collection, representing isolates across 16 distinct STs, were CRISPR2-positive based on both *in silico* PCR screening and CRISPRCasFinder predictions. Similarly, CRISPR2 was identified by at least one of these methodologies in 98.3% (1,439 out of 1,464) of the *E. faecalis* genomes obtained from GenBank. These publicly available genomes corresponded to 208 distinct STs registered in the PubMLST database (Data set S1).

Interestingly, out of the remaining 25 genomes where CRISPR2 was not detected by any method, 13 were genetically related, sharing MLST alleles for specific genes. The majority of these were classified as members of ST228, followed by ST624, ST1468, and one strain with an allelic profile not yet registered in the PubMLST database (as of October 2024), which we refer to as “STx” for the purpose of this study (Table 1).

**TABLE 1:** Sampling details and MLST allelic profiles of the 16 *Enterococcus faecalis* strains lacking CRISPR2^*a*^

Strain	ST	MLST allelic profile							Isolation source	Country	Year of isolation	GenBank assembly accession
		*gdh*	*gyd*	*pstS*	*gki*	*aroE*	*xpt*	*yqiL*				
CVM N52662	228	45	5	44	53	48	41	42	Pork chop	United States	2014	GCA_002946755.1
CVM N52467									Ground beef	United States	2014	GCA_002947085.1
CVM N55265									Pork chop	United States	2014	GCA_002947435.1
CVM N54548									Ground turkey	United States	2014	GCA_002948035.1
CVM N53420									Pork chop	United States	2014	GCA_002948515.1
EN24									Chicken carcass rinse samples	New Zealand	2006	GCA_004126865.1
C144									Beef plant conveyor belts	Canada	2016	GCA_006541075.1
C146									Beef plant conveyor belts	Canada	2016	GCA_006541305.1
C138									Beef plant conveyor belts	Canada	2016	GCA_006541395.1
R30									Retail ground beef	Canada	2015	GCA_006541705.1
C116									Beef processing facility	Canada	2015	GCA_017942395.1
R48									Beef processing facility	Canada	2015	GCA_017939215.1
209EA1	624	59	5	78	53	82	36	42	Breast meat (*Gallus gallus domesticus*)	United States	unknown	GCA_003319425.1
DSM111623									Mouse gastrointestinal tract	Germany	2019	GCA_932751055.1
G81	1468	59	50	78	53	82	36	42	Beef plant ground product	Canada	2015	GCA_006541905.1
CVM N52587	STx	45	5	NF	53	48	41	42	Ground Beef	United States	2014	GCA_002947015.1

^*a*^ NF: not found an exact match for the respective locus in the PubMLST database.

The remaining 12 genomes presented several inconsistencies that compromised the reliability of CRISPR2 absence. These included signs of poor assembly or sequencing quality, inconclusive CRISPR predictions, removal from RefSeq due to various anomalies, and distinct MLST allelic profiles, indicating no genetic relatedness either to each other or to the 13-strain group (see Data set 1). Taken together, these issues suggested that CRISPR2 absence in these cases was more likely an artifact of suboptimal data than a genuine biological feature. Accordingly, these genomes were excluded from further analyses. To expand our data set, we subsequently screened newly deposited *E. faecalis* genomes in GenBank for matching or closely related MLST profiles (ST228, ST624, ST1468, and STx), identifying three additional CRISPR2-negative genomes belonging to ST228 and ST624 (Table 1). This brought the total number of high-confidence CRISPR2-negative genomes to 16, which became the focus of all downstream analyses.

In the selected representatives, the absence of CRISPR2 arrays was further verified through comparative analysis of the conserved genomic locus where this array is typically found. The analysis revealed a deletion spanning both the CRISPR2 array and the adjacent EF2063 gene, with the region between homologues of EF2064 and EF2061 devoid of CRISPR2 elements, except for a single vestigial sequence resembling the degenerated terminal repeat, as shown in Fig. S1.

Sample details for the 16 CRISPR2-negative, genetically related strains are summarized in Table 1. All shared identical alleles for the housekeeping genes *gki* [53] and *yqi*L [42], and all but the ST1468 strain also carried the *gyd* [5] allele. Most strains were isolated from meat products or surfaces in meat-processing environments, whereas one originated from the GIT of a mouse. The isolates spanned three continents and were collected between 2006 and 2019. Notably, all lacked CRISPR1-Cas but retained CRISPR3-Cas.

### Genomic evidence for a distinct *E. faecalis* subspecies: taxonomic and phylogenetic assessment

Motivated by the striking absence of CRISPR2 in this genetically related cluster of *E. faecalis* strains, we performed a genome-wide analysis to clarify their taxonomic classification and position within the broader evolutionary context of the species and its closest relatives.

**Fig 2:**
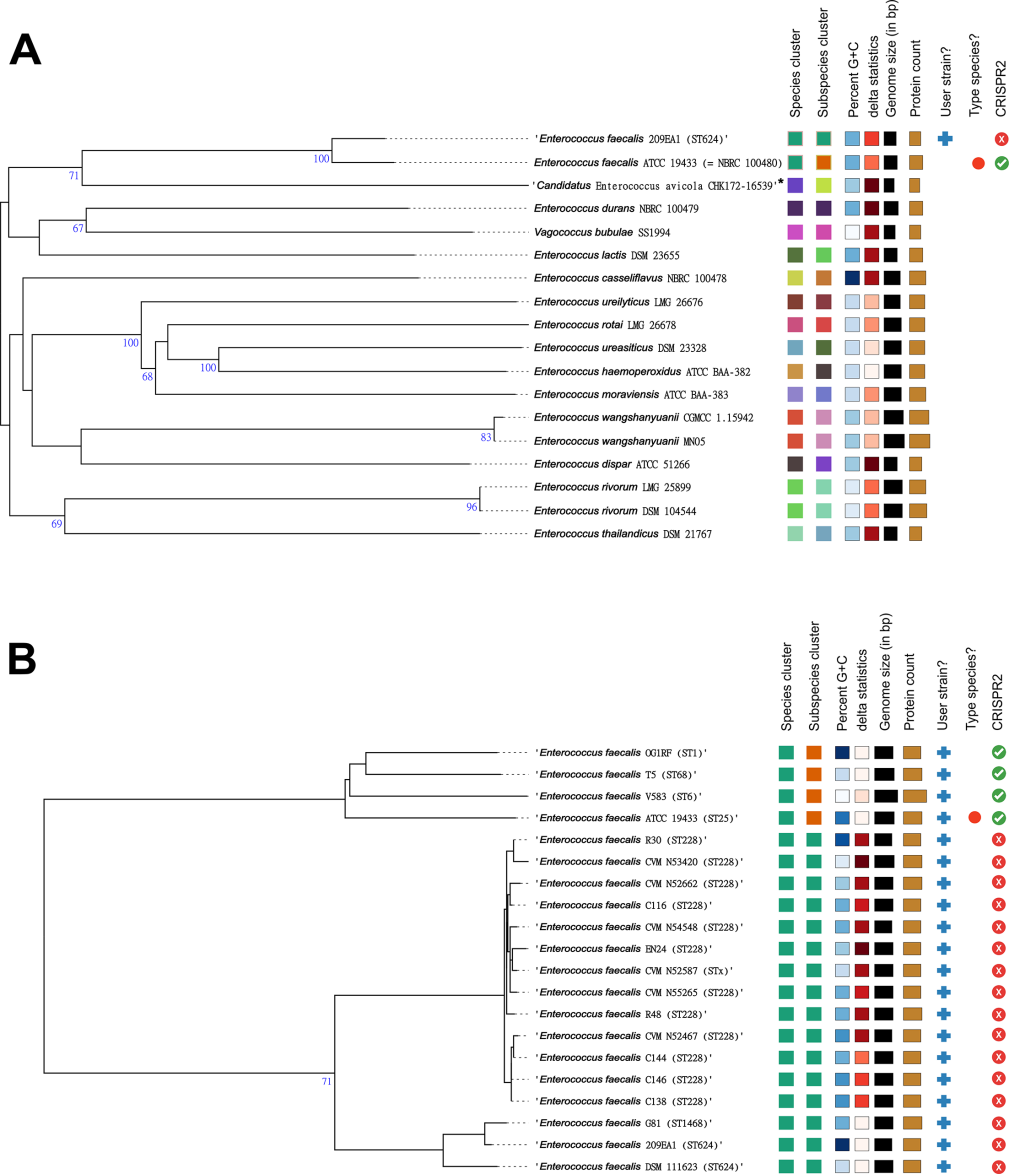
Species and subspecies delineation based on dDDH thresholds. Phylogenetic trees were inferred using FastME 2.1.6.1 from GBDP distances calculated from genome sequences. Branch lengths are scaled according to the GBDP distance formula d5. The numbers above the branches represent GBDP pseudo-bootstrap support values > 60% from 100 replications. The trees were midpoint-rooted. The taxonomic classifications at the species and subspecies levels are represented by color codes on the right side of each tree, based on the dDDH values obtained and the established thresholds (≥70% dDDH for species and ≥79% dDDH for subspecies) (**A**) Whole-genome sequence-based phylogeny of *E. faecalis* 209EA1 (representative strain of the genetically related group of *E. faecalis* lacking CRISPR2) and its closest related type-strains, as determined by TYGS by default. (**B**) Phylogeny focused exclusively on query genomes, encompassing all 16 *E. faecalis* strains lacking CRISPR2 along with *E. faecalis* reference strains, including the type strain ATCC 19433. Sequence types (STs) of each strain are shown in parentheses. An asterisk (*) indicates a type strain of a proposed species with nomenclatural status not yet validly published.

To assess their taxonomic placement, we submitted the 16 CRISPR2-negative genomes to the Type (Strain) Genome Server (TYGS). All were confirmed as *E. faecalis* yet consistently classified as a distinct subspecies from that of the designated type strain (*E. faecalis* ATCC 19433 = NBRC 100480). Taxonomic assignments and pairwise digital DNA:DNA hybridization (dDDH) values are provided in Data set S6. A simplified summary of this result is shown in Fig. 2A, where redundant genomes were excluded and strain 209EA1 was selected as representative of the CRISPR2-negative cluster. This genome was chosen based on its superior sequence and assembly quality, in accordance with the recommendations for prokaryotic genomes used for taxonomic purposes as defined by Riesco and Trujillo (25).

To further explore this novel subspecies differentiation, we conducted a targeted TYGS analysis including the 16 CRISPR2-negative genomes and four clonally distinct *E. faecalis* reference strains: the well-characterized commensal OG1RF (26), the hospital-adapted VRE isolate V583 (27, 28), the NCBI reference genome T5, and the species' type strain. Despite their phylogenetic and ecological diversity, all four reference strains were grouped within the same subspecies and formed a distinct clade from the one containing the CRISPR2-negative strains (Fig. 2B). For clarity, we hereafter refer to this major, CRISPR2-positive lineage as “subspecies A,” and to the CRISPR2-negative one as “subspecies B.”

We expanded our TYGS analysis to investigate whether subspecies B was exclusively composed of genomes lacking CRISPR2, and if the inclusion of additional *E. faecalis* representatives from diverse lineages could reveal new taxonomic subdivisions at the subspecies level. For this purpose, in addition to subspecies B genomes, we incorporated 39 genomes representing 28 distinct STs, encompassing both the genetic diversity from our local collection and other lineages known to harbor CRISPR3-*cas* systems, a trait consistently found in subspecies B (Data set S2). The analysis revealed that all 39 genomes were classified within subspecies A (see Data set S7; Fig. S1), reinforcing the absence of CRISPR2 as a presumptive feature of subspecies B. Based on these results, we established this set of 39 genomes as the representative subset of subspecies A for subsequent comparative genomic analyses against subspecies B, providing a comprehensive framework for capturing the genetic diversity of *E. faecalis*.

To further validate the observed cladogenesis and associated subspecies separation, we performed additional phylogenomic approaches using genome sets spanning broad and short evolutionary scales. The latter, focused on intraspecific resolution, is presented in the next section. Here, we contextualized our findings within the broader evolutionary landscape of the genus *Enterococcus*, using a data set of 117 genomes: 59 *E. faecalis* (including all subspecies A and B representatives plus four additional references) and 58 representative genomes from other *Enterococcus* species. When the type strain genomes were unavailable or suboptimal, higher-quality assemblies were prioritized (Data set S2). *Vagococcus fluvialis* DSM 5731 was selected as the outgroup. Phylogenetic inference was based on the protein sequences of 751 conserved single-copy orthologs present in at least 95.8% of the genomes (details in Materials and Methods).

**Fig 3:**
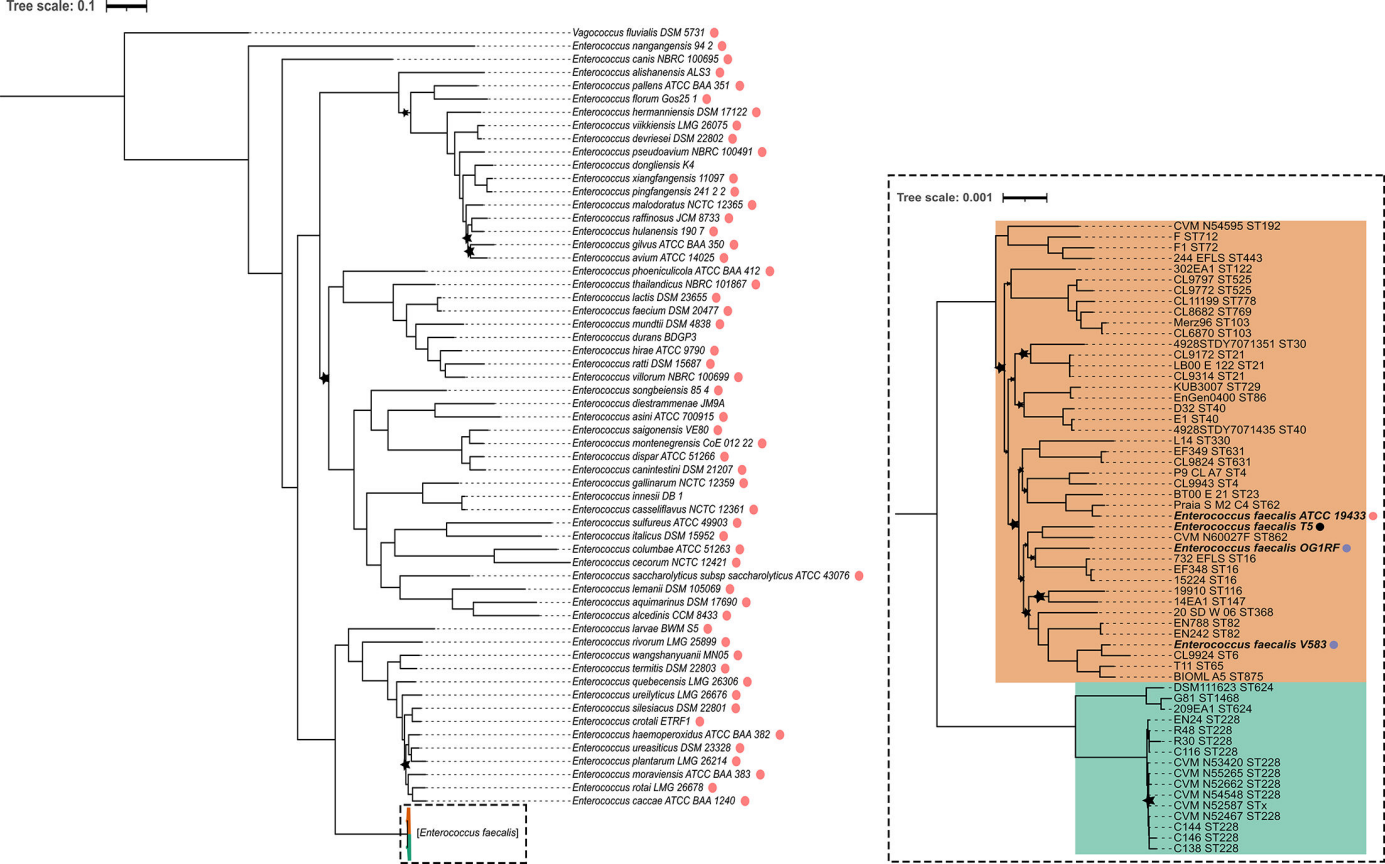
Comprehensive phylogeny of the *Enterococcus* genus illustrating subspecies-level cladogenesis of *E. faecalis*. The tree was estimated using IQ-TREE under the LG + F + I + R9 substitution model with 1,000 bootstrap replicates, based on the concatenation and alignment of 751 protein sequences corresponding to single-copy orthologs present in 95.8% of the 117 sampled enterococci and the outgroup strain *Vagococcus fluvialis* DSM 5731. The tree is presented at different scales. The global phylogeny on the left shows relationships between taxonomic species, with *E. faecalis* strains collapsed into their respective subspecies clades, which are highlighted by colored regions enclosed in a dashed rectangle. On the right, the *E. faecalis* clade is detailed at a fine scale, highlighting subspecies clusters (subspecies A in light orange and subspecies B in light teal). Red dots represent type strains; blue dots indicate *E. faecalis* model strains V583 and OG1RF; the black dot marks the *E. faecalis* reference genome from the NCBI data sets. Stars denote nodes with bootstrap support <95%.

The resulting tree confirmed the classification of *E. faecalis* subspecies A and B as a single taxonomic species, forming a clade clearly distinct from all other *Enterococcus* species (Fig. 3). Within this clade, the two subspecies clustered into well-supported subclades, reflecting the consistency between recent divergence and the taxonomic separation based on dDDH thresholds.

### Pangenome and reverse ecology analyses: gene content and predicted ecological differences between subspecies A and B

**Fig 4:**
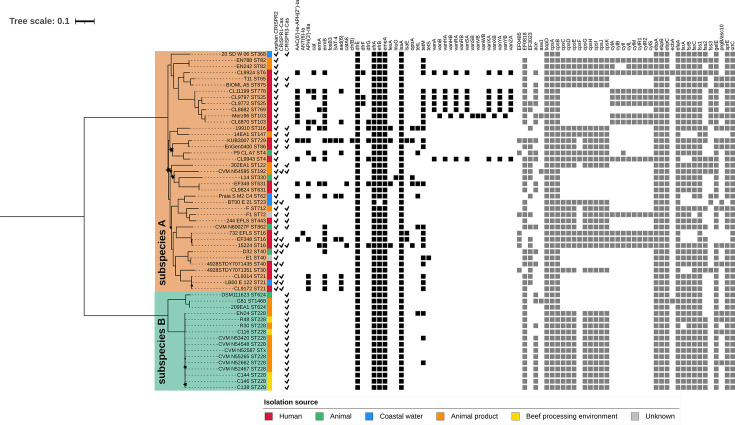
*E. faecalis* intraspecific phylogeny showcasing the distribution of CRISPR, antimicrobial resistance (AMR), and virulence genes across subspecies A and B. The tree was estimated using IQ-TREE under the GTR + G substitution model with 10,000 bootstrap replicates, based on SNPs extracted from the concatenated alignment of the 1,650 core genes present in all 55 sampled *E. faecalis* genomes. Rooted at the midpoint, the tree depicts *E. faecalis* subspecies A and B as the deepest divergences within the species, shown in colored ranges (subspecies A in light orange and subspecies B in light teal). Stars denote nodes with bootstrap support <95%. Strain names are followed by their assigned sequence types (STs) in each leaf label. The colored strip adjacent to the strains indicates their respective isolation sources: human (red), animal (green), coastal water (blue), animal product (orange), beef processing environments (yellow), or unknown origin (gray). Screened genetic traits are shown as binary data in the next panels (filled shapes indicate trait presence and omitted shapes indicate absence), with CRISPR loci as checkmarks, antimicrobial resistance genes as black squares, and virulence genes as dark gray squares.

The subset of 55 *E. faecalis* strains (39 subspecies A and 16 subspecies B), included in the previous phylogeny (Fig. 3), was selected for a pangenome analysis. This analysis aimed to enhance the phylogenetic resolution of the subspecies-level cladogenesis and identify genetic determinants potentially involved in their ecological differentiation. The pangenome comprised 9,950 genes, of which 1,650 were single-copy core genes. Single-nucleotide polymorphisms (SNPs) extracted from the core gene alignment were used to infer the intraspecific phylogeny presented in Fig. 4. CRISPR content, predicted antimicrobial resistance (AMR), and virulence-associated genes are also indicated.

Rooted at the midpoint, the tree reveals a clear subspecies-level divergence within *E. faecalis*, separating into two major clades that reflect the deepest divergences within the species. The larger clade comprising subspecies A genomes displays a more intricate topology with multiple subclades, interconnected by branches of varying lengths and generally lower statistical support. This clade includes most of the STs analyzed, encompassing strains isolated from diverse sources such as humans, animals, meat, and environmental samples (see Data set S1 for details).

Although CRISPR2 is universally present among genomes of subspecies A, the tree reveals a heterogeneous distribution of complete CRISPR-Cas systems (CRISPR1-Cas and CRISPR3-Cas) within this clade. The subspecies A clade also shows considerable variation in the number of AMR and virulence-associated genes, ranging from genomes with few such genes to strains with prominent MDR profiles, including all VRE isolates.

The clade corresponding to subspecies B was supported by the maximum bootstrap value, reinforcing its robustness. Two well-supported branches are observed: one includes strains from ST228 and its single-locus variant (SLV), STx, with internal divergence indistinguishable at the current tree scale; the other includes ST624 and ST1468, which are also SLVs of each other.

As previously noted, all subspecies B genomes lacked both CRISPR2 and CRISPR1-*cas* while uniformly retaining CRISPR3-*cas*. Unlike subspecies A, this group showed a more homogeneous AMR and virulence profile, particularly within the ST228 cluster. Efflux pump genes associated with MDR and biocide resistance (e.g., *drfE, efrA/B,* and *lsaA*) (29, 30) were common, although also present in most subspecies A genomes. Three subspecies B strains carried *tet*M, and one also harbored *tet*L.

Although not unique to subspecies B, a broad array of virulence genes was identified in most genomes of this group, especially within the ST228 cluster. These genes include homologs of the *E. faecalis* V583 ORF EF0818, which encodes a family 8 polysaccharide lyase (31); the *bopD* gene, encoding a sugar-binding transcriptional regulator essential for biofilm production (32); the capsule production-associated *cps* operon (except for the *cpsF* gene) (33); the *ebpA/B/C* genes, responsible for encoding endocarditis- and biofilm-associated pili (34); the *efaA* gene, which encodes the *E. faecalis* antigen A (35); the *srtC* gene (also known as *bps*), encoding a biofilm- and pilus-associated sortase (36); the *fss1* gene, encoding a fibrinogen-binding MSCRAMM (Microbial Surface Components Recognizing Adhesive Matrix Molecules) (37); and the *gelE* and *sprE* genes, which encode the secreted proteases gelatinase and serine protease, respectively. These proteases are regulated by the Fsr quorum-sensing system, encoded by the *fsrA/B/C* locus, which was also detected in these genomes (38, 39).

On the other hand, key differences in the resistome and virulome between subspecies A and B were observed. The *ermB* gene and homologs of EF3023 were statistically underrepresented in subspecies B (*P* values < 0.005 after Benjamini-Hochberg correction). The *ermB* gene encodes an enzyme responsible for resistance to macrolides, lincosamides, and streptogramin B (MLS_B_) (40), whereas EF3023 encodes HylA, a virulence-associated enzyme whose exact role remains to be elucidated (41). However, in the comparative epidemiological context, it is also worth noting that other AMR and virulence genes were exclusively detected in subspecies A. These include determinants involved in high-level aminoglycoside resistance (HLAR) (*aac(6′)-Ie-aph(2′)-Ia*, *ant(6)-Ia, aph(3’)-IIIa*), glycopeptide resistance (*van* genes), oxazolidinone resistance (*cfr(B*) and *optrA*), as well as the operon encoding the pore-forming exotoxin cytolysin (*cylL_L_*, *cylL_S_*, *cylM*, *cylB*, *cylA*, and *cylI*) and its regulatory genes (*cylR1* and *cylR2*) (16, 42).

Interestingly, the detection of the *ace* gene, which encodes the virulence-associated adhesin to collagen of *E. faecalis* (Ace), revealed subspecies-specific variations in similarity to its corresponding reference sequence in VFDB (NP_814829). Specifically, genes from subspecies A showed higher similarity, as indicated by elevated coverage (100% in most cases) and identity percentages (>95% in all cases) (details are shown in Data set S3). In contrast, sequences from subspecies B exhibited uniformly lower similarity, with reduced coverage and identity (both indices < 85% in all cases) relative to the same reference sequence.

Given the evolutionary distance between subspecies A and B and the generalist lifestyle of *E. faecalis* (6), we aimed to infer differential ecological properties based on their genomes. For this purpose, we conducted a pangenome-wide association analysis using Scoary (43), which identified 664 genes significantly associated with either subspecies. Of these, 59.3% (394/664) were overrepresented in subspecies A and 40.7% (270/664) in subspecies B (Data set S4). Functional categorization with COGclassifier (44) revealed significant homology to known proteins for 73.9% of subspecies A-enriched genes (291/394) and 55.2% of subspecies B-enriched genes (149/270). Although a large fraction (particularly in subspecies B) could not be assigned to known COG categories, some informative patterns emerged.

**Fig 5:**
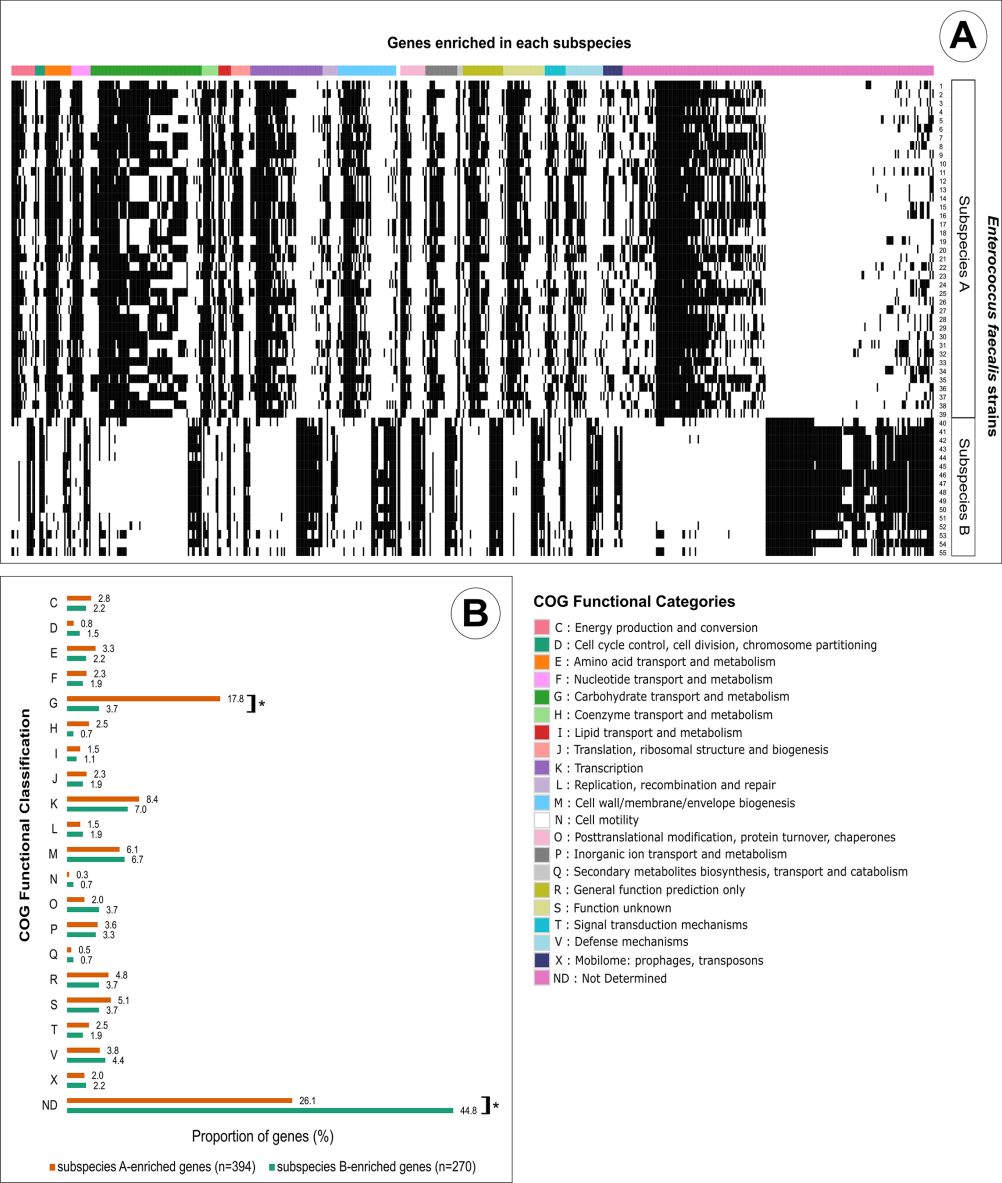
COG functional classification of subspecies-enriched genes. (**A**) Heatmap showing the distribution of the 664 genes significantly associated with subspecies A and B, color-coded by their respective COG functional categories. Black-filled cells represent the presence of a gene in each strain. (**B**) Comparison of gene frequencies across COG functional categories between subspecies A and subspecies B. Each bar represents the proportion of genes annotated in a given functional category in relation to the total number of overrepresented genes in the respective subspecies. An asterisk (*) indicates a *P*-value < 0.01 (chi-squared test).

Functionally annotated genes from both subspecies were assigned to the same 20 COG categories (Fig. 5A). Remarkably, subspecies A exhibited a significantly higher proportion of genes involved in carbohydrate transport and metabolism (COG category G; *P* = 0.002785) compared with subspecies B (Fig. 5B). No significant differences were observed in the distribution of other functional categories.

To identify candidate niche-specifying genes that marked the origin of each subspecies clade (i.e., genes potentially responsible for functional novelties that redefined the ecological niche of their most recent common ancestor) (21, 45, 46), we conducted an additional filtration of the subspecies-enriched genes. Specifically, we focused on those conserved in all members of one clade while absent in all members of the other. This approach led to the preliminary identification of 31 genes associated with subspecies A and 76 genes associated with subspecies B (Data set S5).

Building on this, we sought to further elucidate the selective pressures that shaped the emergence, maintenance, and diversification of these subspecies by employing a reverse ecology approach (21). This allowed us to infer the likely ecological roles provided by these candidate niche-specifying genes based on their predicted functions. However, the automatic annotation performed by Prokka revealed that 74.2% (23/31) of the candidate niche-specifying genes in subspecies A and 81.6% (62/76) in subspecies B encoded “hypothetical proteins,” indicating a lack of significant matches with known sequences in the databases included in Prokka.

Recognizing these gaps, we conducted a more comprehensive manual annotation of all candidate niche-specifying genes. We utilized BLASTp to compare each sequence against the UniProtKB reference proteomes, Swiss-Prot database, and unreviewed TrEMBL entries, thereby expanding the search coverage. For the follow-up discussion, we focused on the best alignment matches exhibiting significant similarity indicative of homology (see Materials and Methods section for details). Additionally, we examined these sequences against the InterPro protein signature databases to identify protein family memberships, domains, conserved sites, and other features critical for functional characterization.

We observed that 20 genes from subspecies A and 20 from subspecies B exhibited significant similarity to the same reference sequences from *E. faecalis* strain ATCC 700802 /V583 in our BLASTp analysis against UniProtKB. Notably, the nuanced differences in sequence similarity between these genes and the reference sequences suggest that they are likely subspecies-specific allelic variants of the same 20 genes, rather than representing 40 distinct orthologous groups (Table 2).

**TABLE 2:** List of *E. faecalis* subspecies-specific core allelic variants and their putative biological roles based on similarity searches^*a*^

UniProtKB best protein matches										
Putative biological roles^*b*^	Gene names	Annotation	Length (Amino acids)	Subspecies A alignment metrics			Subspecies B alignment metrics			UniProtKB accession
				Identity (%)	Score	E-value	Identity (%)	Score	E-value	
Horizontal gene transfer	EF_1340	Pheromone cAM373 lipoprotein	166	100	838	1.8e-113	94	787	1.0e-105	H7C6X9
	comFC, EF_1765	Competence protein F	228	97.4	1207	1.1e-167	93.4	1165	2.9e-161	Q834A5
Stress response	EF_3078	UPF0637 protein EF_3078	203	98	1038	9.7e-143	94.6	1009	2.6e-138	Q82ZH9
Antibiotic resistance	EF_1102	DUF1093 domain-containing protein (YxeA-like)	133	100	690	5.7e-92	94.7	649	1.0e-85	Q836K9
Regulation of enzymatic activity and other functions	EF_3054	PepSY domain containing-lipoprotein, putative	208	96.6	1006	1.1e-137	82.2	827	1.1e-110	Q82ZK1
Unknown function	EF_2967	DUF1146 domain-containing protein	74	100	384	3.7e-47	89.2	351	4.0e-42	Q82ZT0
Metabolism; biosynthesis of essential compounds; energy production and conversion	EF_2916	Hydrolase, haloacid dehalogenase-like family protein, 5’-nucleotidase	217	98.6	1105	4.6e-152	92.1	1035	8.1e-142	Q82ZY1
Carbohydrate metabolism and virulence	EF_2504	Big-domain containing protein	233	99.6	1164	6.0e-161	92.3	1078	7.7e-148	Q831K1
Protein synthesis; translation, ribosomal structure and biogenesis	EF_2484	Diphthamide synthase domain-containing protein	216	99.5	1116	3.4e-154	94.4	1057	3.3e-145	Q831M0
Horizontal gene transfer and regulation of gene expression	EF_1985	Late competence protein ComG	117	94.9	559	1.7e-72	90.6	528	8.9e-68	Q833G9
Nucleotide transport and metabolism; biosynthesis of essential compounds and regulation of gene expression	purK-1, purK, EF_1786	N5-carboxyaminoimidazole ribonucleotide synthase, N5-CAIR synthase, 6.3.4.18, 5-(carboxyamino) imidazole ribonucleotide synthetase	374	96.8	1856	0	93.3	1796	0	Q833Y5
Defense mechanism	EF_1651	Abortive infection protein (Rce1-like)	217	97.7	1084	2.8e-149	94.9	1053	1.5e-144	Q834J9
Septum cleavage; virulence; cell wall/membrane/envelope biogenesis	EF_1546	LysM domain protein	208	97.6	1016	3.5e-139	93.4	987	1.0e-134	Q834T7
Regulation of gene expression and stress response	EF_1369	Transcriptional regulator, Cro/CI family	116	98.3	571	2.3e-74	94.8	542	6.0e-70	Q835K8
Multifunctional; general function prediction only	EF_1296	Acetyltransferase, GNAT family	198	100	1031	7.7e-142	93.4	965	9.0E-132	Q835S7
Antibiotic resistance	EF_1101	Methyltransferase domain-containing protein/S-adenosyl-L-methionine (SAM)-dependent methyltransferase superfamily	244	98.8	1259	4.6e-175	94.2	1212	6.7e-168	Q836L0
Regulation of gene expression; adhesion, virulence, and stress response	EF_0876	Mga helix-turn-helix domain- containing protein	474	99.6	2474	0	93.5	2528	0	Q837G6
Adhesion, virulence	EF_0095	Membrane lipoprotein, putative (PsaA homolog)	261	100	1308	0	93.1	1331	0	Q839R3
Cell cycle control, cell division, chromosome partitioning	EF_2629	Uncharacterized protein	236	97.9	1137	9.8e-157	91.9	1085	8.3e-149	Q830Y5
Unknown function	EF_1105	Transmembrane uncharacterized protein	101	99	501	3.6e-64	92.1	470	1.9e-59	Q836K6

^*a*^ All BLASTp best hits listed in the table correspond to alignments with protein sequences predicted from the *Enterococcus faecalis* ATCC 700802/V583 model strain, as annotated automatically in the UniProtKB/TrEMBL database, except for the EF_3078 encoded protein (Q82ZH9), which has already been reviewed and included in the Swiss-Prot database.

^*b*^ The putative biological roles assigned to each protein cluster were inferred based on their associated COG functional categories, literature review of the UniProtKB best hits, and/or the predicted domains identified by InterProScan.

The putative biological functions of the proteins encoded by these subspecies-specific allelic variants are summarized in Table 2. These predictions highlight several proteins involved in fundamental cellular processes, including enzymes critical for biosynthesis (e.g., those required for protein synthesis), stress response mechanisms, regulation of gene expression, and energy production. Additionally, some proteins are potentially implicated in horizontal gene transfer, defense mechanisms, virulence, or possess currently unknown functions.

Furthermore, an additional 21 candidate niche-specifying genes from subspecies B also exhibited significant similarity to sequences from *E. faecalis* ATCC 700802 /V583, a member of subspecies A (see Data set S5 for further details on these genes and the best alignment matches for their encoded proteins). To mitigate potential sampling biases, we focused our analysis on candidate niche-specifying genes that did not exhibit significant similarity to sequences from the opposing subspecies (Data set S5). This approach ensures that the genes analyzed are more likely to be truly subspecies-specific orthologs.

Following this stringent filtering process, we identified 11 orthologous groups unique to subspecies A strains and 35 unique to subspecies B strains. Detailed information on the proteins encoded by these genes is provided in Tables 3 and 4, respectively. The implications of these findings are discussed in the following section.

**TABLE 3:** List of *E. faecalis* subspecies A-specific proteins and their predicted biological roles based on similarity searches^*a*^

UniProtKB best protein matches							
Putative biological roles^*b*^	Gene names	Annotation	Length (Amino acids)	Subspecies A alignment metrics			UniProtKB accession
				Identity (%)	Score	E-value	
Regulation of gene expression	EF_2063	SoxS-like Transcriptional regulator, AraC family	287	97.6	1467	0	Q833A4
Secondary metabolites biosynthesis, transport, and catabolism; phosphorus metabolism	EF_1926	5-bromo-4-chloroindolyl phosphate hydrolysis protein	179	97.8	887	1.6e-120	Q833L9
Unknown function	EF_1925	Transmembrane uncharacterized protein	281	99.3	1457	0	Q833M0
Transport and binding and energy metabolism	dcuC, EF_1920	C4-dicarboxylate anaerobic carrier DcuC	457	99.8	2221	0	Q833M4
Amino acid transport and metabolism; stress response and antibiotic resistance	EF_1919	Acetyltransferase, GNAT family	162	100	851	1.4e-115	Q833M5
Cell wall/membrane/envelope biogenesis; transport and antibiotic resistance	EF_1672	Peptide ABC superfamily ATP binding cassette transporter permease	427	99.8	2076	0	Q834I0
Carbohydrate transport and metabolism; virulence	scrB-1, EF_1603	Sucrose-6-phosphate hydrolase, 3.2.1.26, Invertase	486	98.8	2566	0	Q834P0
Carbohydrate transport and metabolism; virulence	EF_1602	Glycosyl hydrolase, family 13 (associated with sucrose uptake PTS-system)	557	99.6	2970	0	Q834P1
Secondary metabolites biosynthesis, transport and catabolism; stress response	EF_1138	Oxidoreductase, aldo/keto reductase family	274	99.3	1427	0	Q836H3
Unknown function	EF_1137	Membrane uncharacterized protein	91	98.9	470	3.1e-59	Q836H4
Unknown function	EF_1098	DUF898 family transmembrane protein	101	100	559	5.1e-73	Q836L3

^*a*^ All BLASTp best hits listed in the table correspond to alignments with protein sequences predicted from the *Enterococcus faecalis* ATCC 700802/V583 model strain, as annotated automatically in the UniProtKB/TrEMBL database.

^*b*^ The putative biological roles assigned to each protein cluster were inferred based on their associated COG functional categories, literature review of the UniProtKB best hits, and/or the predicted domains identified by InterProScan.

**TABLE 4:** List of *E. faecalis* subspecies B-specific proteins and their predicted biological roles based on similarity searches

Putative biological roles^*a*^	Gene names	Annotation (UniProtKB best matches and InterProScan prediction)	Length (Amino acids)	Subspecies B alignment metrics			UniProtKB accession	Organism	
				Identity (%)	Score	E-value			
Transport of substrates; defense mechanisms	AB986_01280	ABC transporter domain-containing protein	285	43.7	571	2.5e-69	A0A0J6CTD2	*Alkalihalobacillus macyae*	
Adhesion	IV74_GL000625	OmcB-like DUF11 domain/Fimbrial isopeptide formation D2 domain/LPXTG cell wall anchor motif-containing protein	3404	31.8	1770	0	A0A0R2HW93	*Carnobacterium divergens* DSM 20623	
Adhesion	MPTP_1905	Putative secreted protein/pectate lyase-like adhesive domain-/Ig-like domain-containing protein	328	49.1	774	3.3e-94	F3YCR8	*Melissococcus plutonius* (strain ATCC 35311/CIP 104052/LMG 20360/NCIMB 702443)	
Adhesion and other functions	NCTC13940 _00134	Bacterial Ig-like domain-containing protein	870	35	754	2.8e-85	A0A2 × 3GVB8	*Listeria fleischmannii* subsp. *fleischmannii*	
Adhesion and virulence	BC_3526	Collagen adhesion protein/T-Q ester bond containing domain/LPXTG cell wall anchor motif-containing protein	1093	38.8	889	1.4e-101	Q81AN6	*Bacillus cereus* (strain ATCC 14579/DSM 31/CCUG 7414/JCM 2152/NBRC 15305/NCIMB 9373/NCTC 2599/NRRL B-3711)	
Adhesion and virulence	UC3_00332	LPXTG-domain-containing protein cell wall anchor domain	115	31.3	139	8.6e-9	R3WLQ5	*Enterococcus phoeniculicola* ATCC BAA-412	
Adhesion and virulence	UC3_00333	MucBP domain-containing protein	693	38.5	1228	1.7e-156	R3WMU0	*Enterococcus phoeniculicola* ATCC BAA-412	
Adhesion and virulence	UC3_00333	MucBP domain-containing protein	693	68.4	2646	0	R3WMU0	*Enterococcus phoeniculicola* ATCC BAA-412	
Adhesion and virulence	HSIEG1_102	MucBP domain-containing protein	425	45.8	1012	6.0e-128	T0U5T6	*Enterococcus* sp. HSIEG1	
Adhesion; Carbohydrate metabolism and virulence	FRY98_04280	Chitin-/GlcNAc-binding protein	449	46.7	936	5.9e-120	A0A5D0CXW7	*Paenibacillus faecis*	
Adhesion; Stress response and virulence	UC3_00330	Cell wall-binding WxL domain-containing protein	219	57	627	1.3e-79	R3U4B6	*Enterococcus phoeniculicola* ATCC BAA-412	
Bacteriocin self-immunity; posttranslational modification, protein turnover, chaperones	abi, NRIC_35570	Abortive infection protein/Type II CAAX prenyl endopeptidase Rce1-like	286	35.9	379	5.3e-42	A0A4P5PCT2	*Enterococcus florum*	
Bacteriocin self-immunity; posttranslational modification, protein turnover, chaperones	ESP02_23640	CAAX amino protease/Type II CAAX prenyl endopeptidase Rce1-like	218	50.9	565	2.8e-70	A0A4Y3JRV7	*Enterococcus* sp. NBRC 3427	
Biosynthesis of essential compounds; cell motility	A6E74_01935, ETH01_21050	Glycosyl transferase	442	69.1	1648	0	A0A179EVB0	*Enterococcus thailandicus*	
Carbohydrate transport and metabolism	UAW_01409	PTS system, beta-glucoside-specific IIABC component/ BglF-like	619	59.2	1876	0	R2QSR1	*Enterococcus haemoperoxidus* ATCC BAA-382	
Defense mechanism	cas1, CBF29_ 03235	CRISPR-associated endonuclease Cas1, 3.1.-.-	304	67.1	1132	9.8e-154	A0A430B1Z4	*Vagococcus elongatus*	
Defense mechanism	CBF29_03245	CRISPR-associated protein Csn2 subfamily St	346	54	1001	2.2e-132	A0A430B207	*Vagococcus elongatus*	
Defense mechanism	cas2, CBF29_ 03240	CRISPR-associated endoribonuclease Cas2, 3.1.-.-	102	81.8	427	6.7e-53	A0A430B2E7	*Vagococcus elongatus*	
Energy metabolism	SPHA_34787	NADH dehydrogenase subunit 2	335	40	84	*0.75*	A0A812CH67	*Sepia pharaonis* (Pharaoh cuttlefish)	
Metal ion transmembrane transport; Inorganic ion transport and metabolism	corA_2, NCTC8129_02554	Mg + 2 and co2 transporter	311	67.9	1114	9.4e-151	A0A377KMT5	*Enterococcus durans*	
Post-transcriptional regulation and stress response	A5888_001238	HicA mRNA interferase family protein	60	73.3	237	3.8e-25	A0A242KE25	*Enterococcus* sp. 9E7_DIV0242	
Post-transcriptional regulation; stress response; defense mechanisms	CBF37_01225	HicB family protein	151	58.7	438	1.9e-56	A0A430A2G3	*Vagococcus vulneris*	
Regulation of gene expression; carbohydrate transport and metabolism	CP373A1_10785	PTS sugar transporter/PRD domain-containing protein/putative transcriptional antiterminator/LicT-like	282	54.6	851	1.2e-111	A0A174SGH7	*Clostridium paraputrificum*	
Regulation of gene expression and virulence	ESP02_06520	M protein trans-acting positive regulator	485	58.6	1557	0	A0A4Y3JLF1	*Enterococcus* sp. NBRC 3427	
Regulation of gene expression; Stress response and DNA repair	EVU97_10270	CarD family transcriptional regulator	160	35.1	79	*1.5*	A0A4Q4BV54	*Dermacoccus* sp. 147Ba	
Stress response; inorganic ion transport and metabolism	A5844_001477	Manganese-containing catalase	275	94.2	1483	0	A0A242K1K0	*Enterococcus* sp. 10 A9_DIV0425	
Stress response; virulence; posttranslational modification, protein turnover, chaperones	SIR_1863	Thioredoxin domain-containing protein	172	35	172	9.8e-13	T1ZG32	*Streptococcus intermedius* B196	
Stress response; cell-cycle progression and virulence	CIG19_09425	ClpP/crotonase-like domain-containing protein	279	44.1	541	6.1e-65	A0A2I0FWK9	*Enterobacterales bacterium* CwR94	
Stress response; efflux; inorganic ion transport and metabolism	I573_00550	Cation-transporting P-type ATPase N-terminal domain-containing protein	892	76.8	3466	0	S0KVB7	*Enterococcus sulfureus* ATCC 49903	
Transport of substrates	FHU40_000868	ABC-type transport system involved in multi-copper enzyme maturation permease subunit	269	21.8	114	*0.002*	A0A7W4VTG0	*Nocardioides soli*	
Unknown function	IV74_GL000624	Membrane uncharacterized protein	64	42.2	129	1.5e-8	A0A0R2HWD6	*Carnobacterium divergens* DSM 20623	
Unknown function	SAMN05444955_10515	Uncharacterized protein	229	33.5	241	2.5e-21	A0A1H8D6H3	*Lihuaxuella thermophila*	
Unknown function	CR203_23345	Uncharacterized protein	119	44	301	4.8e-33	A0A3A9KJ78	*Salipaludibacillus neizhouensis*	
Unknown function	C5L23_001335	Uncharacterized protein	524	25.8	435	3.2e-44	A0A4R5N7W9	*Leuconostoc fallax*	
Unknown function	HSIEG1_1210	DUF916 and DUF3324 domains cell surface containing protein	340	42.8	639	8.1e-78	T0VFK5	*Enterococcus* sp. HSIEG1	

^*a*^ The putative biological roles assigned to each protein cluster were inferred based on their associated COG functional categories, literature review of the UniProtKB best hits, and/or the predicted domains identified by InterProScan. Italic values in the table indicate non-significant UniProtKB best hits (E-value > 1e-6), based on BLASTp searches against the database sequences available at the time of the analysis.

## DISCUSSION

The incidental discovery of a genetically cohesive group of *E. faecalis* strains lacking CRISPR2 not only challenges the long-standing assumption that this locus is part of the species' core genome (8, 12) but also highlights an unprecedented subspecies-level separation within *E. faecalis*, based on current genomic criteria for bacterial taxonomy (25, 47, 48). In addition, the evidence of two well-supported and deeply divergent clades questions earlier views that the species lacks internal phylogenetic structure or clearly distinct lineages (6, 15).

From an epidemiological standpoint, the absence of association between subspecies B and human clinical sources, along with the uniform lack of traits commonly found in high-risk lineages (e.g., acquired multidrug resistance and virulence genes linked to treatment failure and increased pathogenicity in enterococcal infections; see reviews [16, 49] for comprehensive overviews) suggests that this subspecies may not pose a significant threat to human health, either as pathogens or as reservoirs of clinically relevant ARGs. However, it is also reasonable to consider that because subspecies B is not commonly found in humans and most molecular epidemiology studies on enterococci are centered around human clinical isolates, our understanding of its niche breadth and ecological roles within a one-health continuum is currently limited.

Drawing from the existing literature and available metadata on strains classified as subspecies B, including the STs represented in our study as well as their single- and double-locus variants cataloged in the PubMLST database (https://pubmlst.org/), it appears that this subspecies is predominantly associated with animal-related sources. Notably, it has been isolated not only from meat and meat processing facilities but also directly from cloacal samples of chickens and wild birds such as the common kingfisher (*Alcedo atthis*). These reports are primarily linked to ST228 (50–52) and reinforce the need for broader surveillance strategies that include non-human reservoirs of enterococci.

The consistent presence of CRISPR3-Cas across all subspecies B genomes suggests limited permissiveness to HGT. As the acquisition of MGEs has been central to the evolution of MDR *E. faecalis* and *E. faecium* lineages (5), and given the established role of CRISPR3-Cas in constraining HGT in *E. faecalis* (53), it is likely that subspecies B evolved under selective pressures distinct from those driving the expansion of high-risk, hospital-adapted strains of subspecies A.

An additional facet of this divergence can be seen in the variability of the *ace* gene between subspecies A and B. The Ace protein of *E. faecalis* mediates binding to host extracellular matrix proteins, such as collagen types I and IV and laminin (54, 55), playing a crucial role in the colonization and infection of various tissues (56, 57). Notably, the detection of *ace* genes in subspecies B genomes with significantly lower similarity to the VFDB reference sequence (NP_814829) compared with the *ace* sequences in subspecies A representatives may suggest tropism for different host environments, especially because this gene is known to be highly conserved among *E. faecalis* isolates (28).

Given that enterococci are hypothesized to have been core members of the gut microbiome in the last common ancestor of mammals, birds, reptiles, and insects (58), it is plausible that the divergence between subspecies A and B was driven by host-specific adaptations and reduced gene flow over time.

Notably, subspecies A exhibited a significantly higher proportion of genes involved in carbohydrate transport and metabolism, supporting the hypothesis that subspecies A may be better adapted to environments with greater availability or diversity of carbohydrates, such as the human gut (59–61). In contrast, this suggests that subspecies B may be adapted to environments where carbohydrate variety is limited or where alternative carbon sources are more prevalent, such as the guts of animals with more restrictive diets compared to humans (62). Carbohydrate availability in the host gut has been indicated as a major driver of enterococcal speciation since the emergence of the genus (63), which enhances the plausibility of our findings.

The significantly higher proportion of unclassified genes in subspecies B suggests the presence of niche-specific adaptations, possibly reflecting ecological contexts distinct from the well-studied human-associated environments where subspecies A thrives. Further research is required to elucidate the roles of these genes and their contributions to subspecies B’s fitness in specific conditions, which will enhance our understanding of *E. faecalis* niche breadth and its complex eco-evolutionary dynamics.

The prediction of subspecies-specific genes and allelic variants (i.e., likely synapomorphic traits and candidate niche-specifying genes) provides valuable targets for understanding the genetic basis of subspecies B’s emancipation and unique ecology (21, 45). Although we cannot confirm whether such allelic variations correspond to actual adaptive mutations or to fixed neutral mutations solely based on these findings, the high degree of non-synonymous substitutions between subspecies A and B sequences suggests potential functional divergence (64).

One example is the EF1546-encoded protein, which contains a LysM domain, a well-characterized motif in autolysins responsible for binding bacterial cell wall peptidoglycan and facilitating precise catalytic cleavage during daughter cell separation (65). Controlled septum cleavage is critical for defining cell size and chain length in *E. faecalis*, factors directly impacting bacterial fitness (65). Mutations within the LysM domain have previously been associated with elongated cell chains, impaired immune evasion, and reduced virulence in *E. faecalis* (65–68). Given that such phenotypic alterations can profoundly affect bacterial morphology and its interaction with host defenses, the divergence in LysM-containing proteins between subspecies A and B may underlie their differential success in host colonization.

Subspecies-specific sequence divergence was also observed in key transcriptional regulators, which may influence the adaptive responses of *E. faecalis* to varying environmental pressures. The EF1369 gene, encoding a Cro/CI family transcriptional regulator, has been linked to stress tolerance, including survival under high-salt conditions (8% NaCl), acidic environments (pH 2.8), and within mouse peritoneal macrophages (69, 70). Similarly, the EF0876 gene, encoding a Mga helix-turn-helix protein, plays a crucial role in virulence regulation, particularly in host tissue adhesion in response to carbon metabolism (71, 72). Notably, inactivation of EF0876 resulted in a 100-fold reduction in gut colonization in mice in a previous study (72), highlighting its importance for *E. faecalis* fitness in the GIT. Mga family regulators, including key *E. faecalis* regulons like *EbpR* (73), are activated by elevated CO_2_ levels (74, 75), typically found inside mammalian hosts (~5-6%), as opposed to atmospheric levels in natural settings (~0.036%) (76, 77). Given the significant role of EF1369 and EF0876 in mediating *E. faecalis* colonization and survival under mammalian host conditions, and considering that these findings are based on subspecies A (i.e., strains derived from the V583 clinical isolate) (69, 72), subspecies-specific sequence divergence in these regulators could impact the fitness of subspecies B under similar selective pressures.

Although the adaptive significance of subspecies-specific allelic variants of core genes is less predictable, the acquisition of entirely novel genes encoding new cellular functions is more likely to enable a strain to exploit previously inaccessible resources and establish itself within a new ecological niche (i.e., niche-specifying genes) (21, 46, 78).

The syntenic organization of many subspecies A-specific genes (i.e., based on the gene arrangement of corresponding homologs in the V583 genome sequence) (27) suggests that they may have been co-selected under a shared ecological pressure, either acquired by the most recent common ancestor of subspecies A or lost in subspecies B.

For instance, the proteins encoded by EF1919 and EF1920 were annotated as a GNAT family acetyltransferase and a C4-dicarboxylate anaerobic carrier DcuC, respectively. Although GNAT members are involved in various cellular processes, and this one has not been linked to any specific pathway so far, DcuC carriers are mainly responsible for succinate efflux produced during glucose fermentation (79). Interestingly, most bacteria containing *dcuC* homologs are pathogenic, and all known DcuC family members are from enteric bacteria (79). This pattern supports the hypothesis that this locus may be integral to a key carbon and energy metabolic pathway in subspecies A strains, particularly under anaerobic conditions in the gut, the primary habitat of *E. faecalis* (1, 80). In contrast, the absence of these genes in subspecies B could indicate a competitive disadvantage for gut colonization, compared with subspecies A.

Interestingly, many other subspecies A-specific genes seem to encode ecologically relevant determinants involved in *E. faecalis’s* ability to either colonize, persist, or cause infection in a host environment. Notably, transcriptomic data have supported the crucial involvement of the EF1672 gene, encoding an ABC superfamily protein permease, in *E. faecalis* physiological adaptation during the course of infection in a mammalian host model, being hypothesized to be one of the core genes required for the species to thrive within a host, and thus a potential target for antimicrobial agents and vaccines to treat and prevent enterococcal infections (81).

Another example implies that subspecies B strains might lack or have a reduced ability to utilize sucrose as a carbon and energy source. This is supported by the exclusive detection of determinants of two operons encoding a sucrose PTS (phosphoenolpyruvate: sugar phosphotransferase system) transporter (EF1602) and sucrose metabolism (EF1603, also named *scrB-1*) within subspecies A strains. Previous reports have shown that both loci likely contribute to virulence since they were upregulated in *E. faecalis* strains grown in human urine (82), where sucrose levels can be increased in high-sugar diets (83). Knockout mutants of EF1603-04 showed reduced virulence in a *Caenorhabditis elegans* infection model (84), which corroborates the differential role of these loci in *E. faecalis* pathogenesis associated with sucrose’s contribution to growth in infection. Additionally, studies have demonstrated that sucrose utilization enhances the expression of *E. faecalis* virulence-associated determinants and the production of biofilm matrix components (e.g., eDNA and EPS) in biofilms (85). Altogether, these findings support the genetic basis of subspecies A’s pathogenic potential in contrast to subspecies B by highlighting the contribution of sucrose uptake and metabolism in infections caused by *E. faecalis*, which seems consistent with this species (specifically, subspecies A) being a notorious agent of UTIs and aligned with the relevance of biofilm formation in the context of HAIs (49).

Additionally, the subspecies A-specific EF1138 gene encodes an oxidoreductase of the aldo/keto reductase family, strongly related to *E. faecalis* V583 stress response induced by bovine bile exposure, being potentially implicated in bile salt modification (86, 87). Bile tolerance mechanisms are critical for bacterial survival and colonization of the GIT, where bile’s emulsifying action poses a challenge (88). The absence of EF1138 in subspecies B may suggest that bile is not a strong selective pressure in its core ecological niche or that subspecies B has evolved alternative mechanisms to tolerate bile exposure or to cope with different bile salt compositions, which includes the possibility of adaptation to a different host range (89–91).

Although the specific ecological role of birds in the lifecycle of subspecies B remains uncertain, previous studies support the hypothesis that wild migratory birds act as reservoirs for genetically diverse *E. faecalis* strains. Some of these strains are prevalent in poultry, indicating possible transmission routes from wild to domestic birds (20, 92). These findings reinforce the idea that subspecies B may have evolved to thrive in non-mammalian hosts, particularly avian species, whose gut microbiota composition is consistently distinct from that of mammals (93).

Furthermore, according to conclusions of a study by Chaillou and colleagues on meat microbiota (94), subspecies B’s common isolation from meat products and processing environments likely points to two primary sources of contamination: (i) animal-derived microbiota, particularly from the skin or gut, which may include *Enterococcus*, and (ii) environmental psychrotrophic bacteria, largely represented by *Firmicutes* associated with water reservoirs, plants, or plant-derived animal feed (94). These hypotheses are not mutually exclusive in the context of *E. faecalis* but rather suggest multiple potential dissemination pathways.

Our efforts to obtain further insights into the unique ecological niche of subspecies B focused on the functional annotation of its specific genes, a reverse ecology approach (21, 45). Despite the significant similarity between subspecies B sequences and their closest matches in the UniProtKB database, notable differences in sequence identity were observed, making functional predictions less definitive. Nevertheless, the detection of conserved protein signatures provided valuable insights into potential ecological roles. Interestingly, most of the predicted homologs were derived from taxa commonly associated with meat microbiota and the contamination routes proposed by Chaillou et al., such as *Vagococcus*, *Carnobacterium*, *Leuconostoc*, various enterococcal species, and others (94). This correlation suggests that these genetic determinants may confer adaptations to shared ecological niches, potentially aiding subspecies B in coping with the selective pressures of these environments.

One of our most compelling findings was the detection of sequences potentially encoding the toxin-antitoxin (TA) protein components HicA and HicB, along with a ClpP/crotonase-like domain-containing protein. These elements may play a coordinated role in stress responses linked to environmental conditions such as nutrient deprivation and heat shock (95, 96). It has also been suggested that TA systems, including HicAB, are typically abundant in free-living bacteria but tend to be lost in host-associated prokaryotes (97, 98). This raises the possibility that subspecies B’s recurrent isolation from meat products could be influenced by environmental contamination routes, potentially involving water, plants, or soil.

We also identified subspecies B-specific traits that may be involved in the uptake and metabolism of alternative carbohydrates when glucose is scarce, which could suggest an adaptive advantage in diverse environments. Notably, we detected protein clusters resembling the IIABC component (BglF-like permease) of a PTS beta-glucoside-specific system and a PRD (PTS regulation domains)-containing protein (LicT-like transcriptional antiterminator) (99, 100). These elements likely participate in the transport and positive regulation of genes linked to beta-glucoside metabolism, responding to substrates like salicin and arbutin found primarily in plant material but rarely in mammals (101).

Moreover, we observed the exclusive presence of putative carbohydrate-binding proteins in subspecies B, such as the GlcNAc-binding protein A (GbpA) and a WxL domain-containing protein. The GbpA homolog contains domains frequently associated with cellulose- and chitin-binding capabilities, polysaccharides predominantly found in plants and arthropods, or fungi, respectively (102, 103). In *Vibrio cholerae*, GbpA facilitates adhesion to zooplankton chitinous surfaces in aquatic habitats and to GlcNAc moieties on human intestinal epithelium (104). This dual functionality strongly suggests an exaptive process where a protein initially evolved for environmental persistence (e.g., attachment to chitin) later adapted to facilitate host colonization (105). By analogy, a similar exaptation in *E. faecalis* subspecies B could enable these bacteria to persist in environmental reservoirs (potentially associating with arthropods or adhering to plant cell walls) and also to colonize animal gut surfaces via GlcNAc binding (104, 105).

The WxL domain-containing proteins, commonly associated with adhesion to cellulose and xylan in other gram-positive bacteria, such as *Enterococcus faecium* (106), reinforce this notion. These polysaccharides, abundant in plant cell walls, are often present in animal diets as dietary fibers. Interestingly, the ability of gut bacteria to adhere to these fibers confers a competitive advantage, especially in environments where plant-based feeds are supplemented with xylanase, facilitating the growth of beneficial microbes like lactobacilli (106, 107). Thus, the exclusive presence of a WxL domain-containing protein in subspecies B suggests that these bacteria might have adapted to adhere to dietary fibers in the gastrointestinal tract of animals, potentially contributing to their isolation from meat products.

Collectively, the subspecies B-specific GlcNAc-binding and WxL domain-containing proteins exemplify how exaptation might facilitate bacterial adaptation to shifting habitats, supporting the two potential transmission routes proposed by Chaillou et al. (94).

Notably, we identified three proteins with MucBP domains in subspecies B, two of which share the highest similarity with putative adhesins from *Enterococcus phoeniculicola* ATCC BAA-412, a species isolated from the uropygial gland of the wild Red-billed Woodhoopoe (*Phoeniculus purpureus*) (108). MucBP domains are commonly linked to adherence within the gastrointestinal mucosa (109), and their presence in a bird-associated enterococcal genome reinforces the possibility that subspecies B has adapted to avian hosts.

Our findings suggest that the niche breadth of *E. faecalis* is likely underestimated, potentially encompassing a range of unexplored or insufficiently studied hosts and environments, which may explain the infrequent reporting of subspecies B-associated STs. By employing a reverse ecology approach, we have provided several insights into the hypothetical ecological properties and potential niches of subspecies B, aligning with current guidelines for the taxonomic description of prokaryotes from genome data (25).

Nonetheless, the limitations inherent to our methods must be acknowledged. Specifically, the focus on clade-specific genes to identify past adaptations conserved through periodic selection inevitably overlooks other genetic determinants that may have contributed to the lineage’s evolution and differentiation, as highlighted by Lassalle et al. (21). Additionally, experimental validation is essential to confirm the speculated roles of clade-specific genes or alleles and corresponding selective pressures in driving the ecological differentiation between *E. faecalis* subspecies A and B, following similar approaches used in related studies (45, 63, 110, 111).

Despite these limitations, our study highlights how genome-based exploratory approaches, utilizing publicly available data, can provide novel insights into the eco-evolutionary dynamics of bacterial species while simultaneously revealing important knowledge gaps. In the present study, analyzing CRISPR distribution patterns across diverse genomes (spanning a broad range of STs, isolation sources, etc.) within the operational definition of a single species enabled us to identify and partially characterize a distinct cluster of *E. faecalis* strains, corresponding to a phylogenetically, ecologically, and taxonomically cohesive unit—subspecies B, using approaches proposed by previous studies (21, 25, 45, 46).

Our findings reinforce the need to reassess *E. faecalis*’s population structure in light of hidden genetic diversity in under-sampled environments and hosts (e.g., nonhuman isolates), as suggested by other studies (20, 80). This underrepresentation likely obscures the existence of host- or environment-specific clades in advanced stages of speciation, such as subspecies B. Such efforts are critical from a One Health perspective, especially given previous evidence that *E. faecalis*’s adaptation to the hospital environment may be a byproduct of its evolution in a wider range of ecological niches (6, 63, 112).

Although opportunities for genetic exchange between subspecies A and B are likely provided in overlapping compartments of their multidimensional niche breadths (113), it remains to be elucidated whether and how gene flow between individuals of these clades is maintained.

## MATERIALS AND METHODS

### *E. faecalis* genomic data

Initially, this study included 71 whole genome sequences of *E. faecalis* strains from our local bacterial culture collection, selected based on data availability rather than specific inclusion criteria. These strains were originally isolated from diverse sources, primarily in the state of Rio de Janeiro, Brazil, including 60 from hospitalized patients, eight from coastal waters, and three from wild birds admitted to wildlife rehabilitation centers, with isolation dates ranging from 2005 to 2017. At this preliminary stage, the goal was to explore CRISPR2 sequence variability across a broad representation of *E. faecalis* genomes, without pre-established hypotheses regarding the ecological or clinical origins of the isolates.

Genomic DNA extraction, purification, and sequencing followed a previously described protocol (114), using the Illumina HiSeq 2500 platform (Illumina Inc., San Diego, CA, USA). Information on local bacterial strains and access details for their corresponding raw sequencing data are provided in the Data set S1. *De novo* genome assemblies were performed with the Unicycler v0.5.0 pipeline (115) on the BV-BRC suite (116), with default parameters and automatic read trimming by Trim Galore v0.6.5 (117).

In addition, 1,464 complete and draft *E. faecalis* genomes, available in GenBank until April 1, 2020, were downloaded for further analysis. All genomes were assigned sequence types (STs) using *in silico* MLST based on PubMLST typing schemes (118, 119). Targeted searches for genomes representing specific STs, flagged as relevant based on initial findings, were conducted in the updated GenBank database (up to May 30, 2022), resulting in the inclusion of three additional *E. faecalis* genomes isolated between 2015 and 2019. Throughout this study, specific subsets of genomes were analyzed to address emerging research questions as new patterns were identified. The rationale for these subsets is detailed alongside the corresponding results to provide contextual clarity and maintain a logical flow of information.

Assembly statistics were retrieved from GenBank to assess the quality of the genomic data, specifically for the initial subset of strains in which CRISPR2 was not detected. Genomes were excluded if they failed to meet quality thresholds recommended by Riesco and Trujillo (25)—including a minimum of 50 × sequencing depth, fewer than 500 contigs, N50 >5 kb, >90% completeness, and <5% contamination—or if they exhibited atypical genomic features inconsistent with *E. faecalis*, such as a divergent GC content relative to the reference genome GCA_000393015.1 (expected GC%: 37.5). Data supporting these evaluations are provided in Data set S1.

All *E. faecalis* genomes analyzed in this study, including strain metadata and accession numbers, are shown in Data set S1. Genome annotation was carried out using Prokka v1.14.5 (120).

### CRISPR screening and genomic context analysis

All *E. faecalis* genomes were screened for the presence of CRISPR2 loci using the following two complementary approaches: the CRISPRCasFinder online tool (121) and an *in silico* PCR program described in reference (122), employing previously described primer sets (123). CRISPRCasFinder detects candidate CRISPR-*cas* systems within a query genome, allowing us to distinguish CRISPR2 loci from other *E. faecalis* CRISPR systems (CRISPR1-*cas* and CRISPR3-*cas*) using two criteria: (i) the presence of signature direct repeats (123), and (ii) the absence of adjacent *cas* genes. Functional CRISPR-*cas* loci (CRISPR1-*cas* and CRISPR3-*cas*) identified by CRISPRCasFinder were also noted for subsequent analysis.

For CRISPR2 detection via *in silico* PCR, any amplification product was considered indicative of the locus’s presence, regardless of size, as the primers were designed to target its conserved flanking regions in the chromosome (123). This differs from the detection of CRISPR1-*cas* and CRISPR3-*cas* systems, which rely on primers targeting the *cas* genes. The variation in product size across strains reflects the number of spacers within the CRISPR2 array. CRISPR2 absence was inferred only when both screening methods (CRISPRCasFinder and *in silico* PCR) failed to detect the array.

To investigate the genomic context of CRISPR2 absence, we first identified conserved flanking genes based on the known CRISPR2 locus location (intergenic region EF2063-EF2061) in the *E. faecalis* V583 genome (123). A pairwise genome alignment using progressiveMauve (124) was performed between V583 and the CRISPR2-negative strain 209EA1, enabling the identification of conserved syntenic regions. This analysis revealed that both CRISPR2 and EF2063 were absent in the latter, with EF2064 and EF2061 emerging as conserved boundaries flanking the deleted region. Based on this, BLASTn was used to locate EF2064 and EF2061 in a curated subset of genomes to define extraction coordinates, including the V583 reference, OG1RF, the type strain ATCC 19433, and the NCBI reference genome for the species (strain T5), as well as four high-quality CRISPR2-negative assemblies (209EA1, C116, C144, and EN24). Accession numbers for all assemblies are provided in Data set S1. BED files were generated from BLAST results, and sequences were retrieved using bedtools getfasta v2.30.0 with strand-specific extraction (“-s”) (125). The retrieved regions were aligned using MAFFT v7.515 with the “—auto” option (126). The resulting alignments were inspected visually in Geneious Prime (127) to detect potential remnants of CRISPR2, with particular attention to conserved direct repeats and the degenerated terminal repeat sequences previously described (123, 128).

### Detection of antimicrobial resistance and virulence-associated genes

Genomes of interest were screened for ARGs and virulence genes against the Comprehensive Antibiotic Resistance Database (CARD; http://arpcard.mcmaster.ca) (129) and the Virulence Factor Database (VFDB; http://www.mgc.ac.cn/VFs/) (130), respectively, using ABRicate v1.0.0 (131) with default parameters.

### Genome-based taxonomy

Taxonomic confirmation of the genomes of interest was conducted using the Type (Strain) Genome Server (TYGS), a high-throughput platform designed for genome-based taxonomic analysis (47, 132). This platform is interconnected with the List of Prokaryotic Names with Standing in Nomenclature (LPSN) database (48). The algorithm assigns each query genome to its corresponding species and subspecies based on established digital DNA:DNA hybridization (dDDH) thresholds—70% for species delineation and 70%–80% for subspecies (133, 134) — using pairwise comparisons against the closest type strains in the TYGS database. Using FastME 2.1.6.1 (135), TYGS also provides a genome-scale phylogeny based on the Genome BLAST Distance Phylogeny method (GBDP), including the query strains and the automatically selected closest type strains, providing branch support values and a treelikeness indicator (47, 132).

### Orthology inference and interspecific *Enterococcus* spp. phylogeny

To evaluate the population structure of *E. faecalis* within a broader evolutionary framework, we selected 118 genomes for phylogenetic analysis. This data set included 55 *E. faecalis* genomes representing the species’ genetic diversity within the scope of this study, four well-characterized *E. faecalis* reference genomes (OG1RF, V583, T5), including the type strain (ATCC 19433 = NBRC 100480), 58 genomes representing other validly published *Enterococcus* species (as of June 13, 2024), and the *Vagococcus fluvialis* DSM 5731 genome as an outgroup. The selection of *E. faecalis* genomes was based on initial genomic and taxonomic analyses, which are detailed in the Results section. Accession numbers, strain names, and taxonomic information for all 59 non-*E*. *faecalis* genomes are provided in Data set S2. Of these, 54 were type strains available in GenBank as of the search date, with exceptions also noted in Data set S2.

To identify orthologous gene groups (orthogroups), we used OrthoFinder (v2.5.5) with the multiple sequence alignment (MSA) option (“-M msa”) across the 118 genomes (136, 137). OrthoFinder’s relaxed approach, which allows for the inclusion of single-copy orthogroups present in the majority of genomes rather than strictly in all genomes, is particularly suited for highly divergent species (136, 137). This relaxed data criterion improves the phylogenetic signal by incorporating genes that, although not universally present, are conserved in a large proportion of genomes (Further details on the basis for this approach are described in the method’s paper [137]). Consequently, 751 orthogroups were selected, each containing single-copy genes present in at least 95.8% of the genomes. By default, the concatenated MSA of these orthogroups was then generated using MAFFT-linsi (126).

The maximum-likelihood phylogenetic tree was constructed using IQ-TREE v2.3.5 (138). ModelFinder Plus (139) was employed to automatically determine the best-fit amino acid substitution model (LG + F + I + R9) via the “-m MFP” option in IQ-TREE. Branch support was assessed with 1,000 ultrafast bootstrap replicates with UFBoot2 (140). The resulting phylogeny was visualized and customized using iTOL (141).

### *E. faecalis* pangenome analysis and intraspecific phylogeny

Pangenome analysis of the 55 *E. faecalis* genomes, representing the species’ genetic diversity within the scope of this study (Data set S1), was conducted using Roary v3.13.0 (142). Out of the 9,950 orthologous gene clusters identified, 1,650 were predicted as core genes (i.e., present in all genomes), and these were then used to build a core gene alignment with MAFFT v7.477 (126). Single-nucleotide polymorphisms (SNPs) were extracted from the alignment using SNP-sites v2.5.1 (143) and then used as input for maximum-likelihood tree inference by IQ-TREE (138) under the generalized time reversible (GTR) model of nucleotide substitution with the Gamma model of rate heterogeneity. Branch support was assessed with 10,000 ultrafast bootstrap replicates using UFBoot2 (140). The resulting phylogeny was rooted at the midpoint and customized using iTOL (141).

### Analysis of clade-level enriched genes

Based on the *E. faecalis* pangenome analysis described in the previous topic, we used Scoary v1.6.16 (43) to identify genes whose frequencies differed significantly between “subspecies A” and “subspecies B”—arbitrary labels employed in this study solely to facilitate comparisons—indicating enrichment or depletion in either group. Genes were considered significantly associated with a given subspecies if they had a *P* value of less than 0.05 after applying the Benjamini-Hochberg correction for multiple comparisons (43). The genes enriched in each subspecies were then classified into their respective COG functional categories (144, 145) using COGClassifier v1.0.5 (44) for a broader functional comparison approach. To further assess differences in the distribution of functional categories between subspecies, we applied the chi-squared test, with statistical significance defined as *P*-value < 0.01.

### Analysis of candidate niche-specifying genes

To identify genes potentially involved in niche differentiation between *E. faecalis* subspecies, we filtered gene clusters that were consistently present in all genomes of one subspecies while absent in the other. These clade-specific genes likely encode functions central to the ecological adaptation of the subspecies, differentiating them from their nearest relatives (21, 46).

Due to the frequent occurrence of inaccurate or incomplete annotations in these genes, initially annotated using Prokka v1.14.5 (120), we performed a secondary functional analysis on representative protein sequences from each clade-specific gene cluster, as identified in the Roary output files. Representative protein sequences were re-annotated through BLASTp (146) searches against the UniProtKB database (147), including reference proteomes and entries from TrEMBL and Swiss-Prot (as of April 2023). Homology was inferred based on sequence similarity, with a cutoff of E-value <1e-6 (148).

Additionally, protein sequences were further characterized using the InterProScan tool (149), which facilitated the identification of conserved domains, active sites, and other protein signatures of known biological function, thus providing insights into potential functional roles of these niche-specifying genes.

## Data Availability

The raw sequence reads for the local E. faecalis strains have been deposited in the NCBI Sequence Read Archive (SRA) under the BioProject accession numbers PRJNA1186978, PRJNA695567, and PRJNA503970. Genome assemblies analyzed in this study include these local strains and publicly available assemblies from GenBank, with accession numbers provided in the Data sets S1 and S2

## SUPPLEMENTAL MATERIAL

Data S1 spectrum.01428-25-s0001.xlsx *E. faecalis* genomes analyzed in this study, including GenBank assemblies, strains from our local collection, and those selected for pangenome analysis.

Data S2 spectrum.01428-25-s0002.xlsx Non-*E. faecalis* enterococcal genomes analyzed in this study, including type strains and NCBI-designated reference genomes.

Data S3 spectrum.01428-25-s0003.xlsx Presence/absence summary of resistance and virulence genes screened against the CARD and VFDB databases, including BLAST alignment metrics.

Data S4 spectrum.01428-25-s0004.xlsx Subspecies-associated genes identified by pangenome-wide association analysis, with functional annotations of those enriched in each group.

Data S5 spectrum.01428-25-s0005.xlsx Candidate niche-specifying genes for each subspecies, along with their corresponding functional annotation data.

Data S6 spectrum.01428-25-s0006.pdf TYGS analysis report for the 16 *E. faecalis* subspecies B genomes.

Data S7 spectrum.01428-25-s0007.pdf TYGS analysis report for the 39 *E. faecalis* subspecies A genomes.

Supplemental figures spectrum.01428-25-s0008.pdf Fig. S1 and S2.

ASM does not own the copyrights to Supplemental Material that may be linked to, or accessed through, an article. The authors have granted ASM a non-exclusive, world-wide license to publish the Supplemental Material files. Please contact the corresponding author directly for reuse.

## References

[1] Lebreton F, Willems RJL, Gilmore MS. 2014. *Enterococcus* diversity, origins in nature, and gut colonization, p 1–56. In Enterococci: from commensals to leading causes of drug resistant infection. Massachusetts Eye and Ear Infirmary, Boston, Massachusetts, USA. 24649510

[2] Fisher K, Phillips C. 2009. The ecology, epidemiology and virulence of Enterococcus. Microbiology (Reading, Engl) 155:1749–1757. doi:10.1099/mic.0.026385-0 19383684

[3] Morandi S, Brasca M, Alfieri P, Lodi R, Tamburini A. 2005. Influence of pH and temperature on the growth of Enterococcus faecium and Enterococcus faecalis. Lait 85. doi:10.1051/lait:2005006

[4] Gaca AO, Lemos JA. 2019. Adaptation to adversity: the intermingling of stress tolerance and pathogenesis in Enterococci. Microbiol Mol Biol Rev 83:e00008-19. doi:10.1128/MMBR.00008-19 31315902 PMC6710459

[5] Johnson CN, Sheriff EK, Duerkop BA, Chatterjee A. 2021. Let me upgrade you: impact of mobile genetic elements on enterococcal adaptation and evolution. J Bacteriol 203:e0017721. doi:10.1128/JB.00177-21 34370561 PMC8508098

[6] Pöntinen AK, Top J, Arredondo-Alonso S, Tonkin-Hill G, Freitas AR, Novais C, Gladstone RA, Pesonen M, Meneses R, Pesonen H, Lees JA, Jamrozy D, Bentley SD, Lanza VF, Torres C, Peixe L, Coque TM, Parkhill J, Schürch AC, Willems RJL, Corander J. 2021. Apparent nosocomial adaptation of Enterococcus faecalis predates the modern hospital era. Nat Commun 12:1523. doi:10.1038/s41467-021-21749-5 33750782 PMC7943827

[7] Hille F, Charpentier E. 2016. CRISPR-Cas: biology, mechanisms and relevance. Phil Trans R Soc B 371:20150496. doi:10.1098/rstb.2015.0496 27672148 PMC5052741

[8] Weaver KE. 2019. Enterococcal genetics. Microbiol Spectr 7:GPP3-0055-2018. doi:10.1128/microbiolspec.gpp3-0055-2018 PMC11590681 30848235

[9] Varahan S, Hancock LE. 2016. To defend or not to defend: that’s the question. mSphere 1. doi:10.1128/mSphere.00127-16 PMC4894676 27306929

[10] Fouquier d’Hérouel A, Wessner F, Halpern D, Ly-Vu J, Kennedy SP, Serror P, Aurell E, Repoila F. 2011. A simple and efficient method to search for selected primary transcripts: non-coding and antisense RNAs in the human pathogen Enterococcus faecalis. Nucleic Acids Res 39:e46. doi:10.1093/nar/gkr012 21266481 PMC3074167

[11] Innocenti N, Golumbeanu M, Fouquier d’Hérouël A, Lacoux C, Bonnin RA, Kennedy SP, Wessner F, Serror P, Bouloc P, Repoila F, Aurell E. 2015. Whole-genome mapping of 5’ RNA ends in bacteria by tagged sequencing: a comprehensive view in Enterococcus faecalis. RNA 21:1018–1030. doi:10.1261/rna.048470.114 25737579 PMC4408782

[12] Hullahalli K, Rodrigues M, Schmidt BD, Li X, Bhardwaj P, Palmer KL. 2015. Comparative analysis of the orphan CRISPR2 locus in 242 Enterococcus faecalis strains. PLoS One 10:e0138890. doi:10.1371/journal.pone.0138890 26398194 PMC4580645

[13] Ping S, Mayorga-Reyes N, Price VJ, Onuoha M, Bhardwaj P, Rodrigues M, Owen J, Palacios Araya D, Akins RL, Palmer KL. 2021. Characterization of presumptive vancomycin-resistant enterococci recovered during infection control surveillance in Dallas, Texas, USA. Access Microbiol 3:000214. doi:10.1099/acmi.0.000214 34151166 PMC8209702

[14] Gawryszewska I, Malinowska K, Kuch A, Chrobak-Chmiel D, Trokenheim LL-, Hryniewicz W, Sadowy E. 2017. Distribution of antimicrobial resistance determinants, virulence-associated factors and clustered regularly interspaced palindromic repeats loci in isolates of Enterococcus faecalis from various settings and genetic lineages. Pathog Dis 75:ftx021. doi:10.1093/femspd/ftx021 28334141 PMC5827583

[15] Palmer KL, Godfrey P, Griggs A, Kos VN, Zucker J, Desjardins C, Cerqueira G, Gevers D, Walker S, Wortman J, Feldgarden M, Haas B, Birren B, Gilmore MS. 2012. Comparative genomics of enterococci: variation in Enterococcus faecalis, clade structure in E. faecium, and defining characteristics of E. gallinarum and E. casseliflavus. mBio 3:e00318–11. doi:10.1128/mBio.00318-11 22354958 PMC3374389

[16] Geraldes C, Tavares L, Gil S, Oliveira M. 2022. Enterococcus virulence and resistant traits associated with its permanence in the hospital environment. Antibiotics (Basel) 11:857. doi:10.3390/antibiotics11070857 35884110 PMC9311936

[17] Raven KE, Reuter S, Gouliouris T, Reynolds R, Russell JE, Brown NM, Török ME, Parkhill J, Peacock SJ. 2016. Genome-based characterization of hospital-adapted Enterococcus faecalis lineages. Nat Microbiol 1:15033. doi:10.1038/nmicrobiol.2015.33 27572164

[18] Dai D, Wang H, Xu X, Chen C, Song C, Jiang D, Du P, Zhang Y, Zeng H. 2018. The emergence of multi-resistant Enterococcus faecalis clonal complex, CC4, causing nosocomial infections. J Med Microbiol 67:1069–1077. doi:10.1099/jmm.0.000761 29923823

[19] Zischka M, Künne CT, Blom J, Wobser D, Sakιnç T, Schmidt-Hohagen K, Dabrowski PW, Nitsche A, Hübner J, Hain T, et al.. 2015. Comprehensive molecular, genomic and phenotypic analysis of a major clone of Enterococcus faecalis MLST ST40. BMC Genomics 16:175. doi:10.1186/s12864-015-1367-x 25887115 PMC4374294

[20] León-Sampedro R, Del Campo R, Rodriguez-Baños M, Lanza VF, Pozuelo MJ, Francés-Cuesta C, Tedim AP, Freitas AR, Novais C, Peixe L, Willems RJL, Corander J, González Candelas F, Baquero F, Coque TM. 2019. Phylogenomics of Enterococcus faecalis from wild birds: new insights into host-associated differences in core and accessory genomes of the species. Environ Microbiol 21:3046–3062. doi:10.1111/1462-2920.14702 31162871

[21] Lassalle F, Muller D, Nesme X. 2015. Ecological speciation in bacteria: reverse ecology approaches reveal the adaptive part of bacterial cladogenesis. Res Microbiol 166:729–741. doi:10.1016/j.resmic.2015.06.008 26192210

[22] Shapiro BJ, Friedman J, Cordero OX, Preheim SP, Timberlake SC, Szabó G, Polz MF, Alm EJ. 2012. Population genomics of early events in the ecological differentiation of bacteria. Science 336:48–51. doi:10.1126/science.1218198 22491847 PMC3337212

[23] Desai PT, Porwollik S, Long F, Cheng P, Wollam A, Bhonagiri-Palsikar V, Hallsworth-Pepin K, Clifton SW, Weinstock GM, McClelland M. 2013. Evolutionary genomics of Salmonella enterica subspecies. mBio 4. doi:10.1128/mBio.00198-13 PMC3604774 23462113

[24] Park CJ, Andam CP. 2020. Distinct but Intertwined evolutionary histories of multiple Salmonella enterica subspecies. mSystems 5:e00515-19. doi:10.1128/mSystems.00515-19 PMC6967386 31937675

[25] Riesco R, Trujillo ME. 2024. Update on the proposed minimal standards for the use of genome data for the taxonomy of prokaryotes. Int J Syst Evol Microbiol 74:e006300. doi:10.1099/ijsem.0.006300 PMC10963913 38512750

[26] Bourgogne A, Garsin DA, Qin X, Singh KV, Sillanpaa J, Yerrapragada S, Ding Y, Dugan-Rocha S, Buhay C, Shen H, et al.. 2008. Large scale variation in Enterococcus faecalis illustrated by the genome analysis of strain OG1RF. Genome Biol 9:R110. doi:10.1186/gb-2008-9-7-r110 18611278 PMC2530867

[27] Paulsen IT, Banerjei L, Myers GSA, Nelson KE, Seshadri R, Read TD, Fouts DE, Eisen JA, Gill SR, Heidelberg JF, et al.. 2003. Role of mobile DNA in the evolution of vancomycin-resistant Enterococcus faecalis . Science 299:2071–2074. doi:10.1126/science.1080613 12663927

[28] Nallapareddy SR, Singh KV, Duh RW, Weinstock GM, Murray BE. 2000. Diversity of ace, a gene encoding a microbial surface component recognizing adhesive matrix molecules, from different strains of Enterococcus faecalis and evidence for production of ace during human infections. Infect Immun 68:5210–5217. doi:10.1128/IAI.68.9.5210-5217.2000 10948146 PMC101780

[29] Jonas BM, Murray BE, Weinstock GM. 2001. Characterization of emeA, a NorA homolog and multidrug resistance efflux pump, in Enterococcus faecalis. Antimicrob Agents Chemother 45:3574–3579. doi:10.1128/AAC.45.12.3574-3579.2001 11709342 PMC90871

[30] Fernández-Fuentes MA, Abriouel H, Ortega Morente E, Pérez Pulido R, Gálvez A. 2014. Genetic determinants of antimicrobial resistance in gram positive bacteria from organic foods. Int J Food Microbiol 172:49–56. doi:10.1016/j.ijfoodmicro.2013.11.032 24361832

[31] Lepage E, Brinster S, Caron C, Ducroix-Crepy C, Rigottier-Gois L, Dunny G, Hennequet-Antier C, Serror P. 2006. Comparative genomic hybridization analysis of Enterococcus faecalis: identification of genes absent from food strains. J Bacteriol 188:6858–6868. doi:10.1128/JB.00421-06 16980489 PMC1595521

[32] Hufnagel M, Koch S, Creti R, Baldassarri L, Huebner J. 2004. A putative sugar-binding transcriptional regulator in a novel gene locus in Enterococcus faecalis contributes to production of biofilm and prolonged bacteremia in mice. J Infect Dis 189:420–430. doi:10.1086/381150 14745699

[33] Thurlow LR, Thomas VC, Hancock LE. 2009. Capsular polysaccharide production in Enterococcus faecalis and contribution of CpsF to capsule serospecificity. J Bacteriol 191:6203–6210. doi:10.1128/JB.00592-09 19684130 PMC2753019

[34] Nallapareddy SR, Singh KV, Sillanpää J, Garsin DA, Höök M, Erlandsen SL, Murray BE. 2006. Endocarditis and biofilm-associated pili of Enterococcus faecalis. J Clin Invest 116:2799–2807. doi:10.1172/JCI29021 17016560 PMC1578622

[35] Low YL, Jakubovics NS, Flatman JC, Jenkinson HF, Smith AW. 2003. Manganese-dependent regulation of the endocarditis-associated virulence factor EfaA of Enterococcus faecalis. J Med Microbiol 52:113–119. doi:10.1099/jmm.0.05039-0 12543916

[36] Kemp KD, Singh KV, Nallapareddy SR, Murray BE. 2007. Relative contributions of Enterococcus faecalis OG1RF sortase-encoding genes, srtA and bps (srtC), to biofilm formation and a murine model of urinary tract infection. Infect Immun 75:5399–5404. doi:10.1128/IAI.00663-07 17785477 PMC2168291

[37] Sillanpää J, Nallapareddy SR, Houston J, Ganesh VK, Bourgogne A, Singh KV, Murray BE, Höök M. 2009. A family of fibrinogen-binding MSCRAMMs from Enterococcus faecalis. Microbiology (Reading) 155:2390–2400. doi:10.1099/mic.0.027821-0 19389755 PMC2739004

[38] Qin X, Singh KV, Weinstock GM, Murray BE. 2001. Characterization of fsr, a regulator controlling expression of gelatinase and serine protease in Enterococcus faecalis OG1RF. J Bacteriol 183:3372–3382. doi:10.1128/JB.183.11.3372-3382.2001 11344145 PMC99635

[39] Qin X, Singh KV, Weinstock GM, Murray BE. 2000. Effects of Enterococcus faecalis fsr genes on production of gelatinase and a serine protease and virulence. Infect Immun 68:2579–2586. doi:10.1128/IAI.68.5.2579-2586.2000 10768947 PMC97462

[40] Portillo A, Ruiz-Larrea F, Zarazaga M, Alonso A, Martinez JL, Torres C. 2000. Macrolide resistance genes in Enterococcus spp. Antimicrob Agents Chemother 44:967–971. doi:10.1128/AAC.44.4.967-971.2000 10722498 PMC89799

[41] Johnson AO, Shipman BM, Hunt BC, Learman BS, Brauer AL, Zhou SP, Hageman Blair R, De Nisco NJ, Armbruster CE. 2024. Function and contribution of two putative Enterococcus faecalis glycosaminoglycan degrading enzymes to bacteremia and catheter-associated urinary tract infection. Infect Immun 92:e0019924. doi:10.1128/iai.00199-24 38842305 PMC11238560

[42] Van Tyne D, Martin MJ, Gilmore MS. 2013. Structure, function, and biology of the Enterococcus faecalis cytolysin. Toxins (Basel) 5:895–911. doi:10.3390/toxins5050895 23628786 PMC3709268

[43] Brynildsrud O, Bohlin J, Scheffer L, Eldholm V. 2016. Rapid scoring of genes in microbial pan-genome-wide association studies with Scoary. Genome Biol 17:238. doi:10.1186/s13059-016-1108-8 27887642 PMC5124306

[44] Shimoyama Y. 2022. COGclassifier: a tool for classifying prokaryote protein sequences into COG functional category. Available from: https://github.com/moshi4/COGclassifier

[45] Lassalle F, Campillo T, Vial L, Baude J, Costechareyre D, Chapulliot D, Shams M, Abrouk D, Lavire C, Oger-Desfeux C, Hommais F, Guéguen L, Daubin V, Muller D, Nesme X. 2011. Genomic species are ecological species as revealed by comparative genomics in Agrobacterium tumefaciens. Genome Biol Evol 3:762–781. doi:10.1093/gbe/evr070 21795751 PMC3163468

[46] Shapiro BJ, Polz MF. 2014. Ordering microbial diversity into ecologically and genetically cohesive units. Trends Microbiol 22:235–247. doi:10.1016/j.tim.2014.02.006 24630527 PMC4103024

[47] Meier-Kolthoff JP, Carbasse JS, Peinado-Olarte RL, Göker M. 2022. TYGS and LPSN: a database tandem for fast and reliable genome-based classification and nomenclature of prokaryotes. Nucleic Acids Res 50:D801–D807. doi:10.1093/nar/gkab902 34634793 PMC8728197

[48] Parte AC. 2014. LPSN—list of prokaryotic names with standing in nomenclature. Nucl Acids Res 42:D613–D616. doi:10.1093/nar/gkt1111 24243842 PMC3965054

[49] Fiore E, Van Tyne D, Gilmore MS. 2019. Pathogenicity of enterococci. Microbiol Spectr 7. doi:10.1128/microbiolspec.gpp3-0053-2018 PMC6629438 31298205

[50] Holman DB, Klima CL, Gzyl KE, Zaheer R, Jones TH, Mcallister TA. 2021. Antimicrobial resistance in Enterococcus spp. bioRxiv. doi:10.1101/2021.05.28.446255 PMC8597637 34787441

[51] Splichalova P, Svec P, Ghosh A, Zurek L, Oravcova V, Radimersky T, Bohus M, Literak I. 2015. Prevalence, diversity and characterization of enterococci from three coraciiform birds. Antonie Van Leeuwenhoek 107:1281–1289. doi:10.1007/s10482-015-0422-6 25772302

[52] Fertner ME, Olsen RH, Bisgaard M, Christensen H. 2011. Transmission and genetic diversity of Enterococcus faecalis among layer chickens during hatch. Acta Vet Scand 53:56. doi:10.1186/1751-0147-53-56 22017822 PMC3214791

[53] Price VJ, Huo W, Sharifi A, Palmer KL. 2016. CRISPR-Cas and restriction-modification act additively against conjugative antibiotic resistance plasmid transfer in Enterococcus faecalis. mSphere 1:mSphere doi:10.1128/mSphere.00064-16 PMC4894674 27303749

[54] Tomita H, Ike Y. 2004. Tissue-specific adherent Enterococcus faecalis strains that show highly efficient adhesion to human bladder carcinoma T24 cells also adhere to extracellular matrix proteins . Infect Immun 72:5877–5885. doi:10.1128/IAI.72.10.5877-5885.2004 15385489 PMC517594

[55] Nallapareddy SR, Qin X, Weinstock GM, Höök M, Murray BE. 2000. Enterococcus faecalis adhesin, ace, mediates attachment to extracellular matrix proteins collagen type IV and laminin as well as collagen type I. Infect Immun 68:5218–5224. doi:10.1128/IAI.68.9.5218-5224.2000 10948147 PMC101781

[56] Singh KV, Nallapareddy SR, Sillanpää J, Murray BE. 2010. Importance of the collagen adhesin ace in pathogenesis and protection against Enterococcus faecalis experimental endocarditis. PLoS Pathog 6:e1000716. doi:10.1371/journal.ppat.1000716 20072611 PMC2798748

[57] Lebreton F, Riboulet-Bisson E, Serror P, Sanguinetti M, Posteraro B, Torelli R, Hartke A, Auffray Y, Giard JC. 2009. Ace, which encodes an adhesin in Enterococcus faecalis, is regulated by Ers and is involved in virulence. Infect Immun 77:2832–2839. doi:10.1128/IAI.01218-08 19433548 PMC2708572

[58] Van Tyne D, Gilmore MS. 2014. Friend turned foe: evolution of enterococcal virulence and antibiotic resistance. Annu Rev Microbiol 68:337–356. doi:10.1146/annurev-micro-091213-113003 25002090 PMC4384341

[59] Payling L, Fraser K, Loveday SM, Sims I, Roy N, McNabb W. 2020. The effects of carbohydrate structure on the composition and functionality of the human gut microbiota. Trends in Food Science & Technology 97:233–248. doi:10.1016/j.tifs.2020.01.009

[60] Mora-Flores LP, Moreno-Terrazas Casildo R, Fuentes-Cabrera J, Pérez-Vicente HA, de Anda-Jáuregui G, Neri-Torres EE. 2023. The role of carbohydrate intake on the gut microbiome: a weight of evidence systematic review. Microorganisms 11:1728. doi:10.3390/microorganisms11071728 37512899 PMC10385781

[61] Ley RE, Lozupone CA, Hamady M, Knight R, Gordon JI. 2008. Worlds within worlds: evolution of the vertebrate gut microbiota. Nat Rev Microbiol 6:776–788. doi:10.1038/nrmicro1978 18794915 PMC2664199

[62] Adebowale TO, Yao K, Oso AO. 2019. Major cereal carbohydrates in relation to intestinal health of monogastric animals: a review. Anim Nutr 5:331–339. doi:10.1016/j.aninu.2019.09.001 31890909 PMC6920401

[63] Lebreton F, Manson AL, Saavedra JT, Straub TJ, Earl AM, Gilmore MS. 2017. Tracing the enterococci from paleozoic origins to the hospital. Cell 169:849–861. doi:10.1016/j.cell.2017.04.027 28502769 PMC5499534

[64] Savolainen O, Lascoux M, Merilä J. 2013. Ecological genomics of local adaptation. Nat Rev Genet 14:807–820. doi:10.1038/nrg3522 24136507

[65] Salamaga B, Turner RD, Elsarmane F, Galley NF, Kulakauskas S, Mesnage S. 2023. A moonlighting role for LysM peptidoglycan binding domains underpins Enterococcus faecalis daughter cell separation. Commun Biol 6:428. doi:10.1038/s42003-023-04808-z 37072531 PMC10113225

[66] Weiser JN. 2013. The battle with the host over microbial size. Curr Opin Microbiol 16:59–62. doi:10.1016/j.mib.2013.01.001 23395472 PMC3622179

[67] Dalia AB, Weiser JN. 2011. Minimization of bacterial size allows for complement evasion and is overcome by the agglutinating effect of antibody. Cell Host Microbe 10:486–496. doi:10.1016/j.chom.2011.09.009 22100164 PMC3222866

[68] Salamaga B, Prajsnar TK, Jareño-Martinez A, Willemse J, Bewley MA, Chau F, Ben Belkacem T, Meijer AH, Dockrell DH, Renshaw SA, Mesnage S. 2017. Bacterial size matters: multiple mechanisms controlling septum cleavage and diplococcus formation are critical for the virulence of the opportunistic pathogen Enterococcus faecalis. PLoS Pathog 13:e1006526. doi:10.1371/journal.ppat.1006526 28742152 PMC5542707

[69] Michaux C, Hartke A, Martini C, Reiss S, Albrecht D, Budin-Verneuil A, Sanguinetti M, Engelmann S, Hain T, Verneuil N, Giard JC. 2014. Involvement of Enterococcus faecalis small RNAs in stress response and virulence. Infect Immun 82:3599–3611. doi:10.1128/IAI.01900-14 24914223 PMC4187846

[70] Shioya K, Michaux C, Kuenne C, Hain T, Verneuil N, Budin-Verneuil A, Hartsch T, Hartke A, Giard JC. 2011. Genome-wide identification of small RNAs in the opportunistic pathogen Enterococcus faecalis V583. PLoS One 6:e23948. doi:10.1371/journal.pone.0023948 21912655 PMC3166299

[71] Hondorp ER, McIver KS. 2007. The Mga virulence regulon: infection where the grass is greener. Mol Microbiol 66:1056–1065. doi:10.1111/j.1365-2958.2007.06006.x 18001346

[72] Rigottier-Gois L, Madec C, Navickas A, Matos RC, Akary-Lepage E, Mistou MY, Serror P. 2015. The surface rhamnopolysaccharide epa of Enterococcus faecalis is a key determinant of intestinal colonization. J Infect Dis 211:62–71. doi:10.1093/infdis/jiu402 25035517

[73] Bourgogne A, Thomson LC, Murray BE. 2010. Bicarbonate enhances expression of the endocarditis and biofilm associated pilus locus, ebpR-ebpABC, in Enterococcus faecalis. BMC Microbiol 10. doi:10.1186/1471-2180-10-17 PMC2824692 20092636

[74] Dai Z, Koehler TM. 1997. Regulation of anthrax toxin activator gene (atxA) expression in Bacillus anthracis: temperature, not CO2/bicarbonate, affects AtxA synthesis. Infect Immun 65:2576–2582. doi:10.1128/iai.65.7.2576-2582.1997 9199422 PMC175364

[75] Caparon MG, Geist RT, Perez-Casal J, Scott JR. 1992. Environmental regulation of virulence in group A streptococci: transcription of the gene encoding M protein is stimulated by carbon dioxide. J Bacteriol 174:5693–5701. doi:10.1128/jb.174.17.5693-5701.1992 1512202 PMC206517

[76] Cummins EP, Selfridge AC, Sporn PH, Sznajder JI, Taylor CT. 2014. Carbon dioxide-sensing in organisms and its implications for human disease. Cell Mol Life Sci 71:831–845. doi:10.1007/s00018-013-1470-6 24045706 PMC3945669

[77] Bahn Y-S, Cox GM, Perfect JR, Heitman J. 2005. Carbonic anhydrase and CO2 sensing during Cryptococcus neoformans growth, differentiation, and virulence. Curr Biol 15:2013–2020. doi:10.1016/j.cub.2005.09.047 16303560

[78] Cohan FM. 2002. What are bacterial species? Annu Rev Microbiol 56:457–487. doi:10.1146/annurev.micro.56.012302.160634 12142474

[79] Janausch IG, Zientz E, Tran QH, Kröger A, Unden G. 2002. C4-dicarboxylate carriers and sensors in bacteria. Biochimica et Biophysica Acta (BBA) - Bioenergetics 1553:39–56. doi:10.1016/S0005-2728(01)00233-X 11803016

[80] He Q, Hou Q, Wang Y, Li J, Li W, Kwok LY, Sun Z, Zhang H, Zhong Z. 2018. Comparative genomic analysis of Enterococcus faecalis: insights into their environmental adaptations. BMC Genomics 19:527. doi:10.1186/s12864-018-4887-3 29996769 PMC6042284

[81] Frank KL, Colomer-Winter C, Grindle SM, Lemos JA, Schlievert PM, Dunny GM. 2014. Transcriptome analysis of Enterococcus faecalis during mammalian infection shows cells undergo adaptation and exist in a stringent response state. PLoS One 9:e115839. doi:10.1371/journal.pone.0115839 25545155 PMC4278851

[82] Vebø HC, Solheim M, Snipen L, Nes IF, Brede DA. 2010. Comparative genomic analysis of pathogenic and probiotic Enterococcus faecalis isolates, and their transcriptional responses to growth in human urine. PLoS One 5:e12489. doi:10.1371/journal.pone.0012489 20824220 PMC2930860

[83] Tasevska N, Runswick SA, McTaggart A, Bingham SA. 2005. Urinary sucrose and fructose as biomarkers for sugar consumption. Cancer Epidemiol Biomarkers Prev 14:1287–1294. doi:10.1158/1055-9965.EPI-04-0827 15894688

[84] Maadani A, Fox KA, Mylonakis E, Garsin DA. 2007. Enterococcus faecalis mutations affecting virulence in the Caenorhabditis elegans model host. Infect Immun 75:2634–2637. doi:10.1128/IAI.01372-06 17307944 PMC1865755

[85] Kim MA, Rosa V, Min KS. 2020. Characterization of Enterococcus faecalis in different culture conditions. Sci Rep 10:21867. doi:10.1038/s41598-020-78998-5 33318537 PMC7736865

[86] Bøhle LA, Færgestad EM, Veiseth-Kent E, Steinmoen H, Nes IF, Eijsink VGH, Mathiesen G. 2010. Identification of proteins related to the stress response in Enterococcus faecalis V583 caused by bovine bile. Proteome Sci 8:37. doi:10.1186/1477-5956-8-37 20579342 PMC2907315

[87] Pfeiler EA, Azcarate-Peril MA, Klaenhammer TR. 2007. Characterization of a novel bile-inducible operon encoding a two-component regulatory system in Lactobacillus acidophilus. J Bacteriol 189:4624–4634. doi:10.1128/JB.00337-07 17449631 PMC1913432

[88] Begley M, Gahan CGM, Hill C. 2005. The interaction between bacteria and bile. FEMS Microbiol Rev 29:625–651. doi:10.1016/j.femsre.2004.09.003 16102595

[89] Une M, Hoshita T. 1994. Natural occurrence and chemical synthesis of bile alcohols, higher bile acids, and short side chain bile acids. Hiroshima J Med Sci 43:37–67. 7928396

[90] Hofmann AF, Hagey LR, Krasowski MD. 2010. Bile salts of vertebrates: structural variation and possible evolutionary significance. J Lipid Res 51:226–246. doi:10.1194/jlr.R000042 19638645 PMC2803226

[91] Haslewood GA. 1967. Bile salt evolution. J Lipid Res 8:535–550. 4862128

[92] Petersen A, Chadfield MS, Christensen JP, Christensen H, Bisgaard M. 2008. Characterization of small-colony variants of Enterococcus faecalis isolated from chickens with amyloid arthropathy. J Clin Microbiol 46:2686–2691. doi:10.1128/JCM.00343-08 18579713 PMC2519495

[93] Waite DW, Taylor MW. 2015. Exploring the avian gut microbiota: current trends and future directions. Front Microbiol 6:673. doi:10.3389/fmicb.2015.00673 26191057 PMC4490257

[94] Chaillou S, Chaulot-Talmon A, Caekebeke H, Cardinal M, Christieans S, Denis C, Desmonts MH, Dousset X, Feurer C, Hamon E, Joffraud J-J, La Carbona S, Leroi F, Leroy S, Lorre S, Macé S, Pilet M-F, Prévost H, Rivollier M, Roux D, Talon R, Zagorec M, Champomier-Vergès M-C. 2015. Origin and ecological selection of core and food-specific bacterial communities associated with meat and seafood spoilage. ISME J 9:1105–1118. doi:10.1038/ismej.2014.202 25333463 PMC4409155

[95] Jørgensen MG, Pandey DP, Jaskolska M, Gerdes K. 2009. HicA of Escherichia coli defines a novel family of translation-independent mRNA interferases in bacteria and archaea. J Bacteriol 191:1191–1199. doi:10.1128/JB.01013-08 19060138 PMC2631989

[96] Winter AJ, Williams C, Isupov MN, Crocker H, Gromova M, Marsh P, Wilkinson OJ, Dillingham MS, Harmer NJ, Titball RW, Crump MP. 2018. The molecular basis of protein toxin HicA-dependent binding of the protein antitoxin HicB to DNA. J Biol Chem 293:19429–19440. doi:10.1074/jbc.RA118.005173 30337369 PMC6302177

[97] Makarova KS, Grishin NV, Koonin EV. 2006. The HicAB cassette, a putative novel, RNA-targeting toxin-antitoxin system in archaea and bacteria. Bioinformatics 22:2581–2584. doi:10.1093/bioinformatics/btl418 16895922

[98] Pandey DP, Gerdes K. 2005. Toxin-antitoxin loci are highly abundant in free-living but lost from host-associated prokaryotes. Nucleic Acids Res 33:966–976. doi:10.1093/nar/gki201 15718296 PMC549392

[99] Graille M, Zhou C-Z, Receveur-Bréchot V, Collinet B, Declerck N, van Tilbeurgh H. 2005. Activation of the LicT transcriptional antiterminator involves a domain swing/lock mechanism provoking massive structural changes. J Biol Chem 280:14780–14789. doi:10.1074/jbc.M414642200 15699035

[100] Le Coq D, Lindner C, Krüger S, Steinmetz M, Stülke J. 1995. New beta-glucoside (bgl) genes in Bacillus subtilis: the bglP gene product has both transport and regulatory functions similar to those of BglF, its Escherichia coli homolog. J Bacteriol 177:1527–1535. doi:10.1128/jb.177.6.1527-1535.1995 7883710 PMC176769

[101] Kiliç AO, Tao L, Zhang Y, Lei Y, Khammanivong A, Herzberg MC. 2004. Involvement of Streptococcus gordonii beta-glucoside metabolism systems in adhesion, biofilm formation, and in vivo gene expression. J Bacteriol 186:4246–4253. doi:10.1128/JB.186.13.4246-4253.2004 15205427 PMC421613

[102] Folders J, Tommassen J, van Loon LC, Bitter W. 2000. Identification of a chitin-binding protein secreted by Pseudomonas aeruginosa. J Bacteriol 182:1257–1263. doi:10.1128/JB.182.5.1257-1263.2000 10671445 PMC94410

[103] Schnellmann J, Zeltins A, Blaak H, Schrempf H. 1994. The novel lectin-like protein CHB1 is encoded by a chitin-inducible Streptomyces olivaceoviridis gene and binds specifically to crystalline alpha-chitin of fungi and other organisms. Mol Microbiol 13:807–819. doi:10.1111/j.1365-2958.1994.tb00473.x 7815940

[104] Kirn TJ, Jude BA, Taylor RK. 2005. A colonization factor links Vibrio cholerae environmental survival and human infection. Nature 438:863–866. doi:10.1038/nature04249 16341015

[105] Martínez JL. 2018. Ecology and evolution of chromosomal gene transfer between environmental microorganisms and pathogens. Microbiol Spectr 6. doi:10.1128/microbiolspec.mtbp-0006-2016 PMC11633556 29350130

[106] Hao Z, Zhang W, Wang X, Wang Y, Qin X, Luo H, Huang H, Su X. 2022. Identification of WxL and S-layer proteins from Lactobacillus brevis with the ability to bind cellulose and xylan. Int J Mol Sci 23:4136. doi:10.3390/ijms23084136 35456954 PMC9026416

[107] Shaw CN, Kim M, Eastridge ML, Yu Z. 2016. Effects of different sources of physically effective fiber on rumen microbial populations. Animal 10:410–417. doi:10.1017/S1751731115001986 26365790

[108] Law-Brown J, Meyers PR. 2003. Enterococcus phoeniculicola sp. nov., a novel member of the enterococci isolated from the uropygial gland of the red-billed woodhoopoe, Phoeniculus purpureus*.* Int J Syst Evol Microbiol 53:683–685. doi:10.1099/ijs.0.02334-0 12807187

[109] Etzold S, Kober OI, Mackenzie DA, Tailford LE, Gunning AP, Walshaw J, Hemmings AM, Juge N. 2014. Structural basis for adaptation of Lactobacilli to gastrointestinal mucus. Environ Microbiol 16:888–903. doi:10.1111/1462-2920.12377 24373178

[110] Campillo T, Renoud S, Kerzaon I, Vial L, Baude J, Gaillard V, Bellvert F, Chamignon C, Comte G, Nesme X, Lavire C, Hommais F. 2014. Analysis of hydroxycinnamic acid degradation in Agrobacterium fabrum reveals a coenzyme A-dependent, beta-oxidative deacetylation pathway. Appl Environ Microbiol 80:3341–3349. doi:10.1128/AEM.00475-14 24657856 PMC4018866

[111] Materna AC, Friedman J, Bauer C, David C, Chen S, Huang IB, Gillens A, Clarke SA, Polz MF, Alm EJ. 2012. Shape and evolution of the fundamental niche in marine Vibrio. ISME J 6:2168–2177. doi:10.1038/ismej.2012.65 22832347 PMC3504960

[112] Hourigan D, Stefanovic E, Hill C, Ross RP. 2024. Promiscuous, persistent and problematic: insights into current enterococcal genomics to guide therapeutic strategy. BMC Microbiol 24:103. doi:10.1186/s12866-024-03243-2 38539119 PMC10976773

[113] Baquero F, Coque TM, Galán JC, Martinez JL. 2021. The origin of niches and species in the bacterial world. Front Microbiol 12:657986. doi:10.3389/fmicb.2021.657986 33815348 PMC8010147

[114] Freitas A de AR, Souza S da SR, Faria AR, Planet PJ, Merquior VLC, Teixeira LM. 2022. Draft genome sequences of two commensal Enterococcus faecalis strains isolated from american black vultures (Coragyps atratus) in Brazil. Microbiol Resour Announc 11. doi:10.1128/mra.00057-22 PMC9387230 35862905

[115] Wick RR, Judd LM, Gorrie CL, Holt KE. 2017. Unicycler: resolving bacterial genome assemblies from short and long sequencing reads. PLoS Comput Biol 13:e1005595. doi:10.1371/journal.pcbi.1005595 28594827 PMC5481147

[116] Olson RD, Assaf R, Brettin T, Conrad N, Cucinell C, Davis JJ, Dempsey DM, Dickerman A, Dietrich EM, Kenyon RW, et al.. 2023. Introducing the bacterial and viral bioinformatics resource center bacterial and viral bioinformatics resource center (BV-BRC): a resource combining PATRIC, IRD and ViPR. Nucleic Acids Res 51:D678–D689. doi:10.1093/nar/gkac1003 36350631 PMC9825582

[117] Krueger F. 2012. Trim Galore: a wrapper tool around cutadapt and fastqc to consistently apply quality and adapter trimming to fastq files, with some extra functionality for MspI-digested RRBS-type (reduced representation bisufite-seq) libraries. Available from: https://www.bioinformatics.babraham.ac.uk/projects/trim_galore

[118] Seemann T. 2014. MLST: scan contig files against PubMLST typing schemes. Available from: https://github.com/tseemann/mlst

[119] Jolley KA, Bray JE, Maiden MCJ. 2018. Open-access bacterial population genomics: BIGSdb software, the PubMLST.org website and their applications. Wellcome Open Res 3:124. doi:10.12688/wellcomeopenres.14826.1 30345391 PMC6192448

[120] Seemann T. 2014. Prokka: rapid prokaryotic genome annotation. Bioinformatics 30:2068–2069. doi:10.1093/bioinformatics/btu153 24642063

[121] Couvin D, Bernheim A, Toffano-nioche C, Touchon M, Rocha EPC, Vergnaud G, Michalik J, Bertrand N, Gautheret D, Pourcel C, Roux D. 2018. CRISPRCasFinder, an update of CRISRFinder, includes a portable version enhanced performance and integrates search for Cas proteins. Nucleic Acids Res 46:246–251. doi:10.1093/nar/gky425 PMC6030898 29790974

[122] Bikandi J, San Millán R, Rementeria A, Garaizar J. 2004. In silico analysis of complete bacterial genomes: PCR, AFLP-PCR and endonuclease restriction. Bioinformatics 20:798–799. doi:10.1093/bioinformatics/btg491 14752001

[123] Palmer KL, Gilmore MS. 2010. Multidrug-resistant enterococci lack CRISPR-cas. mBio 1:e00227-10. doi:10.1128/mBio.00227-10 21060735 PMC2975353

[124] Darling AE, Mau B, Perna NT. 2010. progressiveMauve: multiple genome alignment with gene gain, loss and rearrangement. PLoS One 5:e11147. doi:10.1371/journal.pone.0011147 20593022 PMC2892488

[125] Quinlan AR, Hall IM. 2010. BEDTools: a flexible suite of utilities for comparing genomic features. Bioinformatics 26:841–842. doi:10.1093/bioinformatics/btq033 20110278 PMC2832824

[126] Katoh K, Standley DM. 2013. MAFFT multiple sequence alignment software version 7: improvements in performance and usability. Mol Biol Evol 30:772–780. doi:10.1093/molbev/mst010 23329690 PMC3603318

[127] Kearse M, Moir R, Wilson A, Stones-Havas S, Cheung M, Sturrock S, Buxton S, Cooper A, Markowitz S, Duran C, Thierer T, Ashton B, Meintjes P, Drummond A. 2012. Geneious basic: an integrated and extendable desktop software platform for the organization and analysis of sequence data. Bioinformatics 28:1647–1649. doi:10.1093/bioinformatics/bts199 22543367 PMC3371832

[128] Hullahalli K, Rodrigues M, Palmer KL. 2017. Exploiting CRISPR-Cas to manipulate Enterococcus faecalis populations. eLife 6:e26664. doi:10.7554/eLife.26664 28644125 PMC5491264

[129] McArthur AG, Waglechner N, Nizam F, Yan A, Azad MA, Baylay AJ, Bhullar K, Canova MJ, De Pascale G, Ejim L, et al.. 2013. The comprehensive antibiotic resistance database. Antimicrob Agents Chemother 57:3348–3357. doi:10.1128/AAC.00419-13 23650175 PMC3697360

[130] Chen L, Yang J, Yu J, Yao Z, Sun L, Shen Y, Jin Q. 2005. VFDB: a reference database for bacterial virulence factors. Nucleic Acids Res 33:D325–8. doi:10.1093/nar/gki008 15608208 PMC539962

[131] Seemann T. 2016. ABRicate: mass screening of contigs for antimicrobial and virulence genes. Available from: https://github.com/tseemann/abricate

[132] Meier-Kolthoff JP, Göker M. 2019. TYGS is an automated high-throughput platform for state-of-the-art genome-based taxonomy. Nat Commun 10:2182. doi:10.1038/s41467-019-10210-3 31097708 PMC6522516

[133] Meier-Kolthoff JP, Hahnke RL, Petersen J, Scheuner C, Michael V, Fiebig A, Rohde C, Rohde M, Fartmann B, Goodwin LA, Chertkov O, Reddy TBK, Pati A, Ivanova NN, Markowitz V, Kyrpides NC, Woyke T, Göker M, Klenk HP. 2014. Complete genome sequence of DSM 30083(T), the type strain (U5/41(T)) of Escherichia coli, and a proposal for delineating subspecies in microbial taxonomy. Stand Genomic Sci 9:2. doi:10.1186/1944-3277-9-2 25780495 PMC4334874

[134] Meier-Kolthoff JP, Auch AF, Klenk HP, Göker M. 2013. Genome sequence-based species delimitation with confidence intervals and improved distance functions. BMC Bioinformatics 14:1–14. doi:10.1186/1471-2105-14-60 23432962 PMC3665452

[135] Lefort V, Desper R, Gascuel O. 2015. FastME 2.0: a comprehensive, accurate, and fast distance-based phylogeny inference program. Mol Biol Evol 32:2798–2800. doi:10.1093/molbev/msv150 26130081 PMC4576710

[136] Emms DM, Kelly S. 2019. OrthoFinder: phylogenetic orthology inference for comparative genomics. Genome Biol 20:238. doi:10.1186/s13059-019-1832-y 31727128 PMC6857279

[137] Emms DM, Kelly S. 2018. STAG: species tree inference from all genes. Evolutionary Biology. doi:10.1101/267914

[138] Minh BQ, Schmidt HA, Chernomor O, Schrempf D, Woodhams MD, von Haeseler A, Lanfear R. 2020. IQ-TREE 2: new models and efficient methods for phylogenetic inference in the genomic era. Mol Biol Evol 37:1530–1534. doi:10.1093/molbev/msaa015 32011700 PMC7182206

[139] Kalyaanamoorthy S, Minh BQ, Wong TKF, von Haeseler A, Jermiin LS. 2017. ModelFinder: fast model selection for accurate phylogenetic estimates. Nat Methods 14:587–589. doi:10.1038/nmeth.4285 28481363 PMC5453245

[140] Hoang DT, Chernomor O, von Haeseler A, Minh BQ, Vinh LS. 2018. UFBoot2: improving the ultrafast bootstrap approximation. Mol Biol Evol 35:518–522. doi:10.1093/molbev/msx281 29077904 PMC5850222

[141] Letunic I, Bork P. 2024. Interactive tree of life (iTOL) v6: recent updates to the phylogenetic tree display and annotation tool. Nucleic Acids Res 52:W78–W82. doi:10.1093/nar/gkae268 38613393 PMC11223838

[142] Page AJ, Cummins CA, Hunt M, Wong VK, Reuter S, Holden MTG, Fookes M, Falush D, Keane JA, Parkhill J. 2015. Roary: rapid large-scale prokaryote pan genome analysis. Bioinformatics 31:3691–3693. doi:10.1093/bioinformatics/btv421 26198102 PMC4817141

[143] Page AJ, Taylor B, Delaney AJ, Soares J, Seemann T, Keane JA, Harris SR. 2016. SNP-sites: rapid efficient extraction of SNPs from multi-FASTA alignments. Microb Genom 2:e000056. doi:10.1099/mgen.0.000056 28348851 PMC5320690

[144] Galperin MY, Wolf YI, Makarova KS, Vera Alvarez R, Landsman D, Koonin EV. 2021. COG database update: focus on microbial diversity, model organisms, and widespread pathogens. Nucleic Acids Res 49:D274–D281. doi:10.1093/nar/gkaa1018 33167031 PMC7778934

[145] Tatusov RL, Galperin MY, Natale DA, Koonin EV. 2000. The COG database: a tool for genome-scale analysis of protein functions and evolution. Nucleic Acids Res 28:33–36. doi:10.1093/nar/28.1.33 10592175 PMC102395

[146] Altschul SF, Gish W, Miller W, Myers EW, Lipman DJ. 1990. Basic local alignment search tool. J Mol Biol 215:403–410. doi:10.1016/S0022-2836(05)80360-2 2231712

[147] Bateman A, Martin MJ, Orchard S, Magrane M, Ahmad S, Alpi E, Bowler-Barnett EH, Britto R, Bye-A-Jee H, Cukura A, et al.. 2023. UniProt: the universal protein knowledgebase in 2023. Nucleic Acids Res 51. doi:10.1093/nar/gkac1052 PMC9825514 36408920

[148] Pearson WR. 2013. An introduction to sequence similarity (“homology”) searching. Curr Protoc Bioinformatics Chapter 3:3. doi:10.1002/0471250953.bi0301s42 PMC3820096 23749753

[149] Paysan-Lafosse T, Blum M, Chuguransky S, Grego T, Pinto BL, Salazar GA, Bileschi ML, Bork P, Bridge A, Colwell L, et al.. 2023. InterPro in 2022. Nucleic Acids Res 51:D418–D427. doi:10.1093/nar/gkac993 36350672 PMC9825450

