# Supplementary material for: Hidden diversity in Enterococcus faecalis revealed by CRISPR2 screening: eco-evolutionary insights into a novel subspecies
Source: Microbiol Spectr. 2025 Sep 8;13(10):e01428-25. doi: 10.1128/spectrum.01428-25 (PMC12502612; doi:10.1128/spectrum.01428-25)
Supplement: Data S6 — TYGS analysis report for the 16 E. faecalis subspecies B genomes. [file spectrum.01428-25-s0006.pdf]

TYPE (STRAIN) GENOME SERVER

JOB SUMMARY

PRINT DATE:	2024-06-25 13:55:44 +0200
JOB ID:	20d8d245-47f7-456b-a70c-c28f8115c2c2
RESULT PAGE:	https://tygs.dsmz.de/user_results/show?guid=20d8d245-47f7-456b-a70c-c28f8115c2c2

Table 1: Phylogenies

Publication-ready versions of both the genome-scale GBDP tree and the 16S rRNA gene sequence tree can be customized and exported either in SVG (vector graphic) or PNG format from within the phylogeny viewers in your TYGS result page. For publications the **SVG format is recommended** because it is lossless, always keeps its high resolution and can also be easily converted to other popular formats such as PDF or EPS. Please follow the link provided above!

Table 2: Identification

The below list contains the result of the TYGS species identification routine.

Explanation of remarks that might occur in the below table:

remark [R1]: The TYGS type strain database is automatically updated on an almost daily basis. However, if a particular type strain genome is not available in the TYGS database, this can have several reasons which are detailed in the FAQ. You can request an extended 16S rRNA gene analysis via the 16S tree viewer found in your result page to detect **not yet genome-sequenced** type strains relevant for your study.

remark [R2]: > 70% dDDH value (formula d_4) and (almost) minimal dDDH values for gene-content formulae d_0 and d_6 indicate a potentially unreliable identification result and should thus be checked via the 16S rRNA gene sequence similarity. Such strong deviations can, in principle, be caused by sequence contamination.

remark [R3]: G+C content difference of > 1 % indicates a potentially unreliable identification result because within species G+C content varies no more than 1 %, if computed from genome sequences (PMID: 24505073).

Strain	Conclusion	Identification result	Remark
**'Enterococcus faecalis 209EA1 (ST624)'**	belongs to known species	*Enterococcus faecalis*	
**'Enterococcus faecalis C116 (ST228)'**	belongs to known species	*Enterococcus faecalis*	see [R2]
**'Enterococcus faecalis C138 (ST228)'**	belongs to known species	*Enterococcus faecalis*	
**'Enterococcus faecalis C144 (ST228)'**	belongs to known species	*Enterococcus faecalis*	see [R2]
**'Enterococcus faecalis C146 (ST228)'**	belongs to known species	*Enterococcus faecalis*	
**'Enterococcus faecalis CVM N52467 (ST228)'**	belongs to known species	*Enterococcus faecalis*	
**'Enterococcus faecalis CVM N52587 (STx)'**	belongs to known species	*Enterococcus faecalis*	
**'Enterococcus faecalis CVM N52662 (ST228)'**	belongs to known species	*Enterococcus faecalis*	
**'Enterococcus faecalis CVM N53420 (ST228)'**	belongs to known species	*Enterococcus faecalis*	
**'Enterococcus faecalis CVM N54548 (ST228)'**	belongs to known species	*Enterococcus faecalis*	
**'Enterococcus faecalis CVM N55265 (ST228)'**	belongs to known species	*Enterococcus faecalis*	
**'Enterococcus faecalis DSM 111623 (ST624)'**	belongs to known species	*Enterococcus faecalis*	see [R2]

Strain	Conclusion	Identification result	Remark
**'Enterococcus faecalis EN24 (ST228)'**	belongs to known species	*Enterococcus faecalis*	
**'Enterococcus faecalis G81 (ST1468)'**	belongs to known species	*Enterococcus faecalis*	
**'Enterococcus faecalis R30 (ST228)'**	belongs to known species	*Enterococcus faecalis*	see [R2]
**'Enterococcus faecalis R48 (ST228)'**	belongs to known species	*Enterococcus faecalis*	see [R2]

Table 3: Pairwise comparisons of user genomes vs. type-strain genomes

The following table contains the pairwise dDDH values between your user genomes and the selected type-strain genomes. The dDDH values are provided along with their confidence intervals (C.I.) for the three different GBDP formulas:

- formula d_0 (a.k.a. GGDC formula 1): length of all HSPs divided by total genome length
- formula d_4 (a.k.a. GGDC formula 2): sum of all identities found in HSPs divided by overall HSP length
- formula d_6 (a.k.a. GGDC formula 3): sum of all identities found in HSPs divided by total genome length

Note: Formula d_4 is independent of genome length and is thus robust against the use of incomplete draft genomes. For other reasons for preferring formula d_4, see the FAQ.

Query	Subject	d_0	C.I. d_0	d_4	C.I. d_4	d_6	C.I. d_6	Diff. G+C Percent
**'Enterococcus faecalis C116 (ST228).fna'**	**'Enterococcus faecalis R30 (ST228).fna'**	99.2	[98.4 - 99.5]	100.0	[99.9 - 100.0]	99.6	[99.3 - 99.8]	0.12
**'Enterococcus faecalis C144 (ST228).fna'**	**'Enterococcus faecalis C146 (ST228).fna'**	100.0	[100.0 - 100.0]	100.0	[99.9 - 100.0]	100.0	[100.0 - 100.0]	0.01
**'Enterococcus faecalis C138 (ST228).fna'**	**'Enterococcus faecalis C146 (ST228).fna'**	100.0	[100.0 - 100.0]	100.0	[100.0 - 100.0]	100.0	[100.0 - 100.0]	0.01
**'Enterococcus faecalis C138 (ST228).fna'**	**'Enterococcus faecalis C144 (ST228).fna'**	100.0	[100.0 - 100.0]	100.0	[99.9 - 100.0]	100.0	[100.0 - 100.0]	0.01
**'Enterococcus faecalis CVM N55265 (ST228).fna'**	**'Enterococcus faecalis R30 (ST228).fna'**	97.4	[95.9 - 98.4]	99.9	[99.8 - 100.0]	98.8	[98.0 - 99.2]	0.12
**'Enterococcus faecalis R30 (ST228).fna'**	**'Enterococcus faecalis R48 (ST228).fna'**	96.3	[94.4 - 97.6]	99.9	[99.7 - 99.9]	98.1	[97.0 - 98.8]	0.14
**'Enterococcus faecalis CVM N53420 (ST228).fna'**	**'Enterococcus faecalis R30 (ST228).fna'**	91.4	[88.4 - 93.7]	99.9	[99.8 - 99.9]	95.0	[93.0 - 96.4]	0.26
**'Enterococcus faecalis CVM N54548 (ST228).fna'**	**'Enterococcus faecalis R30 (ST228).fna'**	96.0	[94.0 - 97.4]	99.9	[99.7 - 99.9]	97.9	[96.8 - 98.7]	0.14
**'Enterococcus faecalis C138 (ST228).fna'**	**'Enterococcus faecalis CVM N52467 (ST228).fna'**	98.2	[96.9 - 98.9]	99.9	[99.8 - 99.9]	99.1	[98.5 - 99.5]	0.02
**'Enterococcus faecalis CVM N52662 (ST228).fna'**	**'Enterococcus faecalis R30 (ST228).fna'**	95.6	[93.5 - 97.1]	99.9	[99.8 - 99.9]	97.7	[96.5 - 98.5]	0.18
**'Enterococcus faecalis CVM N52587 (STx).fna'**	**'Enterococcus faecalis R30 (ST228).fna'**	96.3	[94.3 - 97.6]	99.9	[99.8 - 99.9]	98.1	[97.0 - 98.8]	0.21
**'Enterococcus faecalis C144 (ST228).fna'**	**'Enterococcus faecalis CVM N52467 (ST228).fna'**	98.2	[96.9 - 98.9]	99.9	[99.8 - 99.9]	99.1	[98.5 - 99.5]	0.04
**'Enterococcus faecalis C146 (ST228).fna'**	**'Enterococcus faecalis CVM N52467 (ST228).fna'**	98.2	[96.9 - 98.9]	99.9	[99.8 - 99.9]	99.1	[98.5 - 99.5]	0.03
**'Enterococcus faecalis C116 (ST228).fna'**	**'Enterococcus faecalis EN24 (ST228).fna'**	96.0	[93.9 - 97.3]	99.8	[99.6 - 99.9]	97.9	[96.8 - 98.6]	0.05
**'Enterococcus faecalis C116 (ST228).fna'**	**'Enterococcus faecalis R48 (ST228).fna'**	97.1	[95.4 - 98.2]	99.8	[99.6 - 99.9]	98.5	[97.7 - 99.1]	0.02
**'Enterococcus faecalis C146 (ST228).fna'**	**'Enterococcus faecalis R30 (ST228).fna'**	95.8	[93.7 - 97.2]	99.8	[99.6 - 99.9]	97.8	[96.6 - 98.6]	0.11
**'Enterococcus faecalis EN24 (ST228).fna'**	**'Enterococcus faecalis R30 (ST228).fna'**	97.0	[95.3 - 98.1]	99.8	[99.7 - 99.9]	98.5	[97.6 - 99.1]	0.17
**'Enterococcus faecalis C116 (ST228).fna'**	**'Enterococcus faecalis CVM N52662 (ST228).fna'**	97.0	[95.3 - 98.1]	99.8	[99.6 - 99.9]	98.5	[97.6 - 99.1]	0.06
**'Enterococcus faecalis CVM N52587 (STx).fna'**	**'Enterococcus faecalis CVM N54548 (ST228).fna'**	96.4	[94.5 - 97.7]	99.8	[99.6 - 99.9]	98.1	[97.1 - 98.8]	0.07

Query	Subject	d_0	C.I. d_0	d_4	C.I. d_4	d_6	C.I. d_6	Diff. G+C Percent
**'Enterococcus faecalis C116 (ST228).fna'**	**'Enterococcus faecalis CVM N55265 (ST228).fna'**	96.1	[94.1 - 97.4]	99.8	[99.6 - 99.9]	97.9	[96.8 - 98.7]	0.0
**'Enterococcus faecalis CVM N52467 (ST228).fna'**	**'Enterococcus faecalis CVM N54548 (ST228).fna'**	96.6	[94.7 - 97.8]	99.8	[99.6 - 99.9]	98.2	[97.2 - 98.9]	0.06
**'Enterococcus faecalis C116 (ST228).fna'**	**'Enterococcus faecalis CVM N54548 (ST228).fna'**	94.6	[92.2 - 96.3]	99.8	[99.6 - 99.9]	97.0	[95.6 - 98.0]	0.02
**'Enterococcus faecalis C116 (ST228).fna'**	**'Enterococcus faecalis CVM N52587 (STx).fna'**	94.8	[92.5 - 96.5]	99.8	[99.6 - 99.9]	97.2	[95.8 - 98.1]	0.09
**'Enterococcus faecalis CVM N54548 (ST228).fna'**	**'Enterococcus faecalis CVM N55265 (ST228).fna'**	97.1	[95.5 - 98.2]	99.8	[99.6 - 99.9]	98.6	[97.7 - 99.1]	0.02
**'Enterococcus faecalis CVM N52587 (STx).fna'**	**'Enterococcus faecalis CVM N55265 (ST228).fna'**	99.6	[99.1 - 99.8]	99.8	[99.7 - 99.9]	99.8	[99.6 - 99.9]	0.09
**'Enterococcus faecalis C144 (ST228).fna'**	**'Enterococcus faecalis R30 (ST228).fna'**	95.8	[93.7 - 97.2]	99.8	[99.6 - 99.9]	97.8	[96.6 - 98.5]	0.12
**'Enterococcus faecalis CVM N52662 (ST228).fna'**	**'Enterococcus faecalis CVM N55265 (ST228).fna'**	99.4	[98.8 - 99.7]	99.8	[99.6 - 99.9]	99.7	[99.5 - 99.9]	0.06
**'Enterococcus faecalis C138 (ST228).fna'**	**'Enterococcus faecalis R30 (ST228).fna'**	95.9	[93.8 - 97.3]	99.8	[99.6 - 99.9]	97.8	[96.7 - 98.6]	0.11
**'Enterococcus faecalis C138 (ST228).fna'**	**'Enterococcus faecalis R48 (ST228).fna'**	92.3	[89.5 - 94.5]	99.7	[99.5 - 99.8]	95.6	[93.8 - 96.9]	0.03
**'Enterococcus faecalis C144 (ST228).fna'**	**'Enterococcus faecalis CVM N52662 (ST228).fna'**	95.0	[92.7 - 96.6]	99.7	[99.4 - 99.8]	97.3	[95.9 - 98.2]	0.06
**'Enterococcus faecalis C144 (ST228).fna'**	**'Enterococcus faecalis CVM N55265 (ST228).fna'**	96.6	[94.8 - 97.8]	99.7	[99.5 - 99.8]	98.3	[97.3 - 98.9]	0.0
**'Enterococcus faecalis CVM N52587 (STx).fna'**	**'Enterococcus faecalis CVM N52662 (ST228).fna'**	98.4	[97.3 - 99.1]	99.7	[99.5 - 99.8]	99.3	[98.7 - 99.6]	0.03
**'Enterococcus faecalis C144 (ST228).fna'**	**'Enterococcus faecalis CVM N54548 (ST228).fna'**	96.1	[94.0 - 97.4]	99.7	[99.5 - 99.8]	97.9	[96.8 - 98.7]	0.02
**'Enterococcus faecalis CVM N52662 (ST228).fna'**	**'Enterococcus faecalis CVM N54548 (ST228).fna'**	95.5	[93.3 - 97.0]	99.7	[99.5 - 99.8]	97.6	[96.4 - 98.4]	0.04
**'Enterococcus faecalis C138 (ST228).fna'**	**'Enterococcus faecalis CVM N55265 (ST228).fna'**	96.6	[94.7 - 97.8]	99.7	[99.5 - 99.8]	98.2	[97.2 - 98.9]	0.01
**'Enterococcus faecalis C116 (ST228).fna'**	**'Enterococcus faecalis C138 (ST228).fna'**	94.6	[92.1 - 96.3]	99.7	[99.5 - 99.8]	97.0	[95.6 - 98.0]	0.01
**'Enterococcus faecalis C146 (ST228).fna'**	**'Enterococcus faecalis R48 (ST228).fna'**	92.3	[89.4 - 94.4]	99.7	[99.6 - 99.9]	95.5	[93.7 - 96.8]	0.03
**'Enterococcus faecalis C116 (ST228).fna'**	**'Enterococcus faecalis C146 (ST228).fna'**	94.5	[92.0 - 96.2]	99.7	[99.5 - 99.8]	96.9	[95.5 - 97.9]	0.01
**'Enterococcus faecalis CVM N52587 (STx).fna'**	**'Enterococcus faecalis EN24 (ST228).fna'**	94.0	[91.5 - 95.9]	99.7	[99.5 - 99.8]	96.7	[95.2 - 97.7]	0.04
**'Enterococcus faecalis CVM N52467 (ST228).fna'**	**'Enterococcus faecalis R48 (ST228).fna'**	92.8	[90.0 - 94.9]	99.7	[99.5 - 99.8]	95.9	[94.2 - 97.1]	0.06
**'Enterococcus faecalis C146 (ST228).fna'**	**'Enterococcus faecalis CVM N54548 (ST228).fna'**	96.1	[94.1 - 97.4]	99.7	[99.5 - 99.8]	97.9	[96.8 - 98.7]	0.03
**'Enterococcus faecalis C146 (ST228).fna'**	**'Enterococcus faecalis CVM N55265 (ST228).fna'**	96.6	[94.8 - 97.8]	99.7	[99.5 - 99.8]	98.3	[97.3 - 98.9]	0.0

Query	Subject	d_0	C.I. d_0	d_4	C.I. d_4	d_6	C.I. d_6	Diff. G+C Percent
**'Enterococcus faecalis CVM N54548 (ST228).fna'**	**'Enterococcus faecalis R48 (ST228).fna'**	92.4	[89.5 - 94.5]	99.7	[99.5 - 99.8]	95.6	[93.8 - 96.9]	0.0
**'Enterococcus faecalis CVM N55265 (ST228).fna'**	**'Enterococcus faecalis R48 (ST228).fna'**	92.6	[89.8 - 94.7]	99.7	[99.5 - 99.8]	95.8	[94.0 - 97.0]	0.02
**'Enterococcus faecalis C116 (ST228).fna'**	**'Enterococcus faecalis CVM N52467 (ST228).fna'**	95.0	[92.8 - 96.6]	99.7	[99.4 - 99.8]	97.3	[96.0 - 98.2]	0.04
**'Enterococcus faecalis C138 (ST228).fna'**	**'Enterococcus faecalis CVM N54548 (ST228).fna'**	96.1	[94.1 - 97.4]	99.7	[99.5 - 99.8]	97.9	[96.8 - 98.7]	0.03
**'Enterococcus faecalis C146 (ST228).fna'**	**'Enterococcus faecalis CVM N52587 (STx).fna'**	95.4	[93.2 - 96.9]	99.7	[99.4 - 99.8]	97.5	[96.3 - 98.4]	0.1
**'Enterococcus faecalis C138 (ST228).fna'**	**'Enterococcus faecalis CVM N52662 (ST228).fna'**	94.9	[92.6 - 96.5]	99.7	[99.4 - 99.8]	97.2	[95.9 - 98.1]	0.07
**'Enterococcus faecalis C116 (ST228).fna'**	**'Enterococcus faecalis C144 (ST228).fna'**	94.5	[92.0 - 96.2]	99.7	[99.4 - 99.8]	97.0	[95.5 - 97.9]	0.0
**'Enterococcus faecalis CVM N52587 (STx).fna'**	**'Enterococcus faecalis R48 (ST228).fna'**	91.5	[88.5 - 93.8]	99.7	[99.4 - 99.8]	95.0	[93.1 - 96.4]	0.07
**'Enterococcus faecalis C144 (ST228).fna'**	**'Enterococcus faecalis R48 (ST228).fna'**	92.3	[89.4 - 94.4]	99.7	[99.5 - 99.8]	95.5	[93.7 - 96.8]	0.02
**'Enterococcus faecalis CVM N52467 (ST228).fna'**	**'Enterococcus faecalis R30 (ST228).fna'**	96.7	[94.9 - 97.9]	99.7	[99.5 - 99.8]	98.3	[97.3 - 98.9]	0.08
**'Enterococcus faecalis C146 (ST228).fna'**	**'Enterococcus faecalis CVM N52662 (ST228).fna'**	95.0	[92.6 - 96.6]	99.7	[99.4 - 99.8]	97.2	[95.9 - 98.2]	0.07
**'Enterococcus faecalis C144 (ST228).fna'**	**'Enterococcus faecalis CVM N52587 (STx).fna'**	95.5	[93.3 - 97.0]	99.7	[99.4 - 99.8]	97.6	[96.3 - 98.4]	0.09
**'Enterococcus faecalis C138 (ST228).fna'**	**'Enterococcus faecalis CVM N52587 (STx).fna'**	95.4	[93.2 - 96.9]	99.7	[99.4 - 99.8]	97.5	[96.3 - 98.4]	0.1
**'Enterococcus faecalis EN24 (ST228).fna'**	**'Enterococcus faecalis R48 (ST228).fna'**	94.1	[91.6 - 95.9]	99.6	[99.3 - 99.7]	96.7	[95.2 - 97.8]	0.03
**'Enterococcus faecalis CVM N52662 (ST228).fna'**	**'Enterococcus faecalis R48 (ST228).fna'**	93.9	[91.3 - 95.7]	99.6	[99.4 - 99.8]	96.6	[95.0 - 97.6]	0.04
**'Enterococcus faecalis C144 (ST228).fna'**	**'Enterococcus faecalis CVM N53420 (ST228).fna'**	92.0	[89.0 - 94.2]	99.6	[99.3 - 99.7]	95.3	[93.5 - 96.7]	0.14
**'Enterococcus faecalis CVM N55265 (ST228).fna'**	**'Enterococcus faecalis EN24 (ST228).fna'**	94.8	[92.4 - 96.4]	99.6	[99.4 - 99.8]	97.1	[95.8 - 98.1]	0.05
**'Enterococcus faecalis C146 (ST228).fna'**	**'Enterococcus faecalis CVM N53420 (ST228).fna'**	92.0	[89.0 - 94.2]	99.6	[99.3 - 99.8]	95.3	[93.4 - 96.6]	0.15
**'Enterococcus faecalis CVM N54548 (ST228).fna'**	**'Enterococcus faecalis EN24 (ST228).fna'**	96.5	[94.6 - 97.7]	99.6	[99.4 - 99.8]	98.2	[97.1 - 98.8]	0.03
**'Enterococcus faecalis 209EA1 (ST624).fna'**	**'Enterococcus faecalis G81 (ST1468).fna'**	94.7	[92.3 - 96.4]	99.6	[99.3 - 99.8]	97.1	[95.7 - 98.0]	0.18
**'Enterococcus faecalis C138 (ST228).fna'**	**'Enterococcus faecalis CVM N53420 (ST228).fna'**	92.0	[89.1 - 94.2]	99.6	[99.3 - 99.7]	95.3	[93.5 - 96.7]	0.16
**'Enterococcus faecalis C138 (ST228).fna'**	**'Enterococcus faecalis EN24 (ST228).fna'**	94.5	[92.1 - 96.2]	99.5	[99.1 - 99.7]	97.0	[95.5 - 97.9]	0.06
**'Enterococcus faecalis C116 (ST228).fna'**	**'Enterococcus faecalis CVM N53420 (ST228).fna'**	93.0	[90.3 - 95.0]	99.5	[99.2 - 99.7]	96.0	[94.3 - 97.2]	0.15

Query	Subject	d_0	C.I. d_0	d_4	C.I. d_4	d_6	C.I. d_6	Diff. G+C Percent
**'Enterococcus faecalis CVM N52467 (ST228).fna'**	**'Enterococcus faecalis EN24 (ST228).fna'**	94.8	[92.4 - 96.5]	99.5	[99.3 - 99.7]	97.1	[95.8 - 98.1]	0.09
**'Enterococcus faecalis C144 (ST228).fna'**	**'Enterococcus faecalis EN24 (ST228).fna'**	94.5	[92.0 - 96.2]	99.5	[99.1 - 99.7]	96.9	[95.5 - 97.9]	0.05
**'Enterococcus faecalis C146 (ST228).fna'**	**'Enterococcus faecalis EN24 (ST228).fna'**	94.5	[92.1 - 96.2]	99.5	[99.1 - 99.7]	96.9	[95.5 - 97.9]	0.05
**'Enterococcus faecalis CVM N52467 (ST228).fna'**	**'Enterococcus faecalis CVM N53420 (ST228).fna'**	94.2	[91.6 - 96.0]	99.5	[99.2 - 99.7]	96.7	[95.2 - 97.8]	0.18
**'Enterococcus faecalis CVM N52467 (ST228).fna'**	**'Enterococcus faecalis CVM N52662 (ST228).fna'**	96.3	[94.4 - 97.6]	99.4	[99.0 - 99.6]	98.0	[97.0 - 98.7]	0.1
**'Enterococcus faecalis CVM N53420 (ST228).fna'**	**'Enterococcus faecalis CVM N55265 (ST228).fna'**	92.5	[89.7 - 94.6]	99.4	[99.0 - 99.6]	95.6	[93.8 - 96.9]	0.15
**'Enterococcus faecalis CVM N52662 (ST228).fna'**	**'Enterococcus faecalis EN24 (ST228).fna'**	94.1	[91.6 - 95.9]	99.4	[99.0 - 99.6]	96.7	[95.2 - 97.7]	0.01
**'Enterococcus faecalis CVM N53420 (ST228).fna'**	**'Enterococcus faecalis CVM N54548 (ST228).fna'**	93.4	[90.8 - 95.4]	99.4	[99.1 - 99.6]	96.2	[94.6 - 97.4]	0.12
**'Enterococcus faecalis CVM N52467 (ST228).fna'**	**'Enterococcus faecalis CVM N55265 (ST228).fna'**	98.0	[96.7 - 98.8]	99.4	[99.1 - 99.7]	99.0	[98.3 - 99.4]	0.03
**'Enterococcus faecalis CVM N52587 (STx).fna'**	**'Enterococcus faecalis CVM N53420 (ST228).fna'**	91.7	[88.7 - 93.9]	99.3	[99.0 - 99.6]	95.1	[93.2 - 96.5]	0.05
**'Enterococcus faecalis CVM N53420 (ST228).fna'**	**'Enterococcus faecalis R48 (ST228).fna'**	93.5	[90.9 - 95.4]	99.3	[98.9 - 99.6]	96.3	[94.7 - 97.4]	0.12
**'Enterococcus faecalis CVM N52467 (ST228).fna'**	**'Enterococcus faecalis CVM N52587 (STx).fna'**	97.3	[95.6 - 98.3]	99.3	[99.0 - 99.6]	98.6	[97.7 - 99.1]	0.13
**'Enterococcus faecalis CVM N52662 (ST228).fna'**	**'Enterococcus faecalis CVM N53420 (ST228).fna'**	94.7	[92.3 - 96.3]	99.0	[98.5 - 99.4]	97.0	[95.6 - 98.0]	0.09
**'Enterococcus faecalis CVM N53420 (ST228).fna'**	**'Enterococcus faecalis EN24 (ST228).fna'**	93.3	[90.6 - 95.3]	99.0	[98.5 - 99.4]	96.1	[94.5 - 97.3]	0.1
**'Enterococcus faecalis 209EA1 (ST624).fna'**	**'Enterococcus faecalis DSM 111623 (ST624).fna'**	96.4	[94.5 - 97.7]	97.8	[96.9 - 98.5]	97.9	[96.8 - 98.7]	0.26
**'Enterococcus faecalis DSM 111623 (ST624).fna'**	**'Enterococcus faecalis G81 (ST1468).fna'**	89.5	[86.1 - 92.1]	97.8	[96.9 - 98.5]	93.3	[91.0 - 95.0]	0.08
**'Enterococcus faecalis 209EA1 (ST624).fna'**	**'Enterococcus faecalis CVM N54548 (ST228).fna'**	91.9	[88.9 - 94.1]	92.1	[90.0 - 93.7]	94.2	[92.1 - 95.8]	0.17
**'Enterococcus faecalis 209EA1 (ST624).fna'**	**'Enterococcus faecalis C144 (ST228).fna'**	92.5	[89.6 - 94.6]	92.1	[90.0 - 93.8]	94.7	[92.7 - 96.1]	0.15
**'Enterococcus faecalis 209EA1 (ST624).fna'**	**'Enterococcus faecalis CVM N52467 (ST228).fna'**	92.5	[89.7 - 94.6]	92.0	[90.0 - 93.7]	94.7	[92.7 - 96.2]	0.11
**'Enterococcus faecalis 209EA1 (ST624).fna'**	**'Enterococcus faecalis CVM N55265 (ST228).fna'**	92.2	[89.3 - 94.4]	92.0	[89.9 - 93.7]	94.4	[92.4 - 95.9]	0.15
**'Enterococcus faecalis 209EA1 (ST624).fna'**	**'Enterococcus faecalis CVM N52662 (ST228).fna'**	90.2	[87.0 - 92.7]	92.0	[89.9 - 93.7]	93.0	[90.6 - 94.7]	0.21
**'Enterococcus faecalis 209EA1 (ST624).fna'**	**'Enterococcus faecalis C146 (ST228).fna'**	92.6	[89.7 - 94.7]	92.0	[90.0 - 93.7]	94.7	[92.7 - 96.2]	0.14

Query	Subject	d_0	C.I. d_0	d_4	C.I. d_4	d_6	C.I. d_6	Diff. G+C Percent
**'Enterococcus faecalis 209EA1 (ST624).fna'**	**'Enterococcus faecalis C138 (ST228).fna'**	92.6	[89.8 - 94.7]	92.0	[89.9 - 93.7]	94.7	[92.7 - 96.2]	0.14
**'Enterococcus faecalis 209EA1 (ST624).fna'**	**'Enterococcus faecalis R30 (ST228).fna'**	93.0	[90.2 - 95.0]	92.0	[90.0 - 93.7]	95.0	[93.0 - 96.4]	0.03
**'Enterococcus faecalis 209EA1 (ST624).fna'**	**'Enterococcus faecalis C116 (ST228).fna'**	91.2	[88.1 - 93.6]	92.0	[89.9 - 93.7]	93.7	[91.5 - 95.4]	0.15
**'Enterococcus faecalis 209EA1 (ST624).fna'**	**'Enterococcus faecalis EN24 (ST228).fna'**	90.2	[87.0 - 92.7]	91.9	[89.9 - 93.6]	93.0	[90.7 - 94.8]	0.2
**'Enterococcus faecalis 209EA1 (ST624).fna'**	**'Enterococcus faecalis R48 (ST228).fna'**	89.9	[86.6 - 92.4]	91.9	[89.8 - 93.6]	92.7	[90.4 - 94.6]	0.17
**'Enterococcus faecalis CVM N52467 (ST228).fna'**	**'Enterococcus faecalis DSM 111623 (ST624).fna'**	89.5	[86.1 - 92.1]	91.8	[89.7 - 93.5]	92.4	[90.0 - 94.3]	0.15
**'Enterococcus faecalis 209EA1 (ST624).fna'**	**'Enterococcus faecalis CVM N53420 (ST228).fna'**	87.1	[83.5 - 90.0]	91.8	[89.7 - 93.5]	90.6	[87.9 - 92.7]	0.29
**'Enterococcus faecalis CVM N52587 (STx).fna'**	**'Enterococcus faecalis DSM 111623 (ST624).fna'**	87.4	[83.9 - 90.3]	91.7	[89.5 - 93.4]	90.8	[88.2 - 92.9]	0.02
**'Enterococcus faecalis C144 (ST228).fna'**	**'Enterococcus faecalis G81 (ST1468).fna'**	86.4	[82.8 - 89.4]	91.7	[89.6 - 93.4]	90.1	[87.3 - 92.3]	0.04
**'Enterococcus faecalis C116 (ST228).fna'**	**'Enterococcus faecalis DSM 111623 (ST624).fna'**	88.2	[84.8 - 91.0]	91.7	[89.6 - 93.4]	91.5	[88.9 - 93.5]	0.12
**'Enterococcus faecalis G81 (ST1468).fna'**	**'Enterococcus faecalis R30 (ST228).fna'**	87.9	[84.4 - 90.7]	91.7	[89.6 - 93.5]	91.2	[88.6 - 93.3]	0.15
**'Enterococcus faecalis CVM N54548 (ST228).fna'**	**'Enterococcus faecalis DSM 111623 (ST624).fna'**	89.4	[86.0 - 92.0]	91.7	[89.5 - 93.4]	92.3	[89.9 - 94.2]	0.09
**'Enterococcus faecalis DSM 111623 (ST624).fna'**	**'Enterococcus faecalis R30 (ST228).fna'**	90.2	[87.0 - 92.7]	91.7	[89.6 - 93.5]	92.9	[90.6 - 94.7]	0.23
**'Enterococcus faecalis CVM N52662 (ST228).fna'**	**'Enterococcus faecalis DSM 111623 (ST624).fna'**	86.7	[83.1 - 89.6]	91.6	[89.4 - 93.3]	90.2	[87.5 - 92.4]	0.06
**'Enterococcus faecalis 209EA1 (ST624).fna'**	**'Enterococcus faecalis CVM N52587 (STx).fna'**	92.1	[89.2 - 94.3]	91.6	[89.5 - 93.4]	94.3	[92.2 - 95.8]	0.24
**'Enterococcus faecalis DSM 111623 (ST624).fna'**	**'Enterococcus faecalis R48 (ST228).fna'**	85.9	[82.2 - 88.9]	91.6	[89.5 - 93.4]	89.6	[86.8 - 91.9]	0.09
**'Enterococcus faecalis C146 (ST228).fna'**	**'Enterococcus faecalis G81 (ST1468).fna'**	86.4	[82.8 - 89.4]	91.6	[89.5 - 93.4]	90.0	[87.3 - 92.3]	0.04
**'Enterococcus faecalis CVM N53420 (ST228).fna'**	**'Enterococcus faecalis DSM 111623 (ST624).fna'**	84.0	[80.2 - 87.1]	91.6	[89.4 - 93.3]	88.1	[85.1 - 90.5]	0.03
**'Enterococcus faecalis C146 (ST228).fna'**	**'Enterococcus faecalis DSM 111623 (ST624).fna'**	89.1	[85.8 - 91.8]	91.6	[89.4 - 93.3]	92.1	[89.6 - 94.0]	0.12
**'Enterococcus faecalis C138 (ST228).fna'**	**'Enterococcus faecalis DSM 111623 (ST624).fna'**	89.2	[85.9 - 91.8]	91.6	[89.4 - 93.3]	92.2	[89.7 - 94.1]	0.13
**'Enterococcus faecalis C144 (ST228).fna'**	**'Enterococcus faecalis DSM 111623 (ST624).fna'**	89.1	[85.7 - 91.7]	91.6	[89.5 - 93.4]	92.1	[89.6 - 94.0]	0.11
**'Enterococcus faecalis C138 (ST228).fna'**	**'Enterococcus faecalis G81 (ST1468).fna'**	86.5	[82.9 - 89.4]	91.6	[89.5 - 93.4]	90.1	[87.4 - 92.3]	0.05
**'Enterococcus faecalis CVM N55265 (ST228).fna'**	**'Enterococcus faecalis DSM 111623 (ST624).fna'**	89.0	[85.6 - 91.6]	91.6	[89.5 - 93.3]	92.0	[89.5 - 93.9]	0.12

Query	Subject	d_0	C.I. d_0	d_4	C.I. d_4	d_6	C.I. d_6	Diff. G+C Percent
**'Enterococcus faecalis CVM N53420 (ST228).fna'**	**'Enterococcus faecalis G81 (ST1468).fna'**	89.5	[86.2 - 92.1]	91.5	[89.4 - 93.2]	92.4	[89.9 - 94.2]	0.11
**'Enterococcus faecalis EN24 (ST228).fna'**	**'Enterococcus faecalis G81 (ST1468).fna'**	85.1	[81.3 - 88.2]	91.5	[89.3 - 93.2]	89.0	[86.1 - 91.3]	0.01
**'Enterococcus faecalis C116 (ST228).fna'**	**'Enterococcus faecalis G81 (ST1468).fna'**	88.8	[85.4 - 91.5]	91.5	[89.3 - 93.2]	91.9	[89.4 - 93.8]	0.04
**'Enterococcus faecalis CVM N54548 (ST228).fna'**	**'Enterococcus faecalis G81 (ST1468).fna'**	86.8	[83.2 - 89.7]	91.5	[89.4 - 93.2]	90.3	[87.6 - 92.5]	0.01
**'Enterococcus faecalis CVM N55265 (ST228).fna'**	**'Enterococcus faecalis G81 (ST1468).fna'**	87.9	[84.4 - 90.7]	91.4	[89.2 - 93.2]	91.2	[88.5 - 93.2]	0.04
**'Enterococcus faecalis DSM 111623 (ST624).fna'**	**'Enterococcus faecalis EN24 (ST228).fna'**	87.6	[84.1 - 90.5]	91.4	[89.2 - 93.1]	90.9	[88.3 - 93.0]	0.07
**'Enterococcus faecalis CVM N52467 (ST228).fna'**	**'Enterococcus faecalis G81 (ST1468).fna'**	88.5	[85.1 - 91.3]	91.2	[89.0 - 93.0]	91.6	[89.1 - 93.6]	0.07
**'Enterococcus faecalis G81 (ST1468).fna'**	**'Enterococcus faecalis R48 (ST228).fna'**	89.2	[85.9 - 91.9]	91.2	[89.0 - 93.0]	92.1	[89.7 - 94.0]	0.01
**'Enterococcus faecalis CVM N52662 (ST228).fna'**	**'Enterococcus faecalis G81 (ST1468).fna'**	88.2	[84.7 - 90.9]	91.2	[89.0 - 93.0]	91.3	[88.7 - 93.4]	0.02
**'Enterococcus faecalis CVM N52587 (STx).fna'**	**'Enterococcus faecalis G81 (ST1468).fna'**	88.4	[84.9 - 91.1]	91.2	[89.0 - 92.9]	91.5	[88.9 - 93.5]	0.06
**'Enterococcus faecalis 209EA1 (ST624).fna'**	*Enterococcus faecalis* NBRC 100480	81.5	[77.6 - 84.8]	76.9	[73.9 - 79.6]	83.6	[80.3 - 86.4]	0.21
**'Enterococcus faecalis DSM 111623 (ST624).fna'**	*Enterococcus faecalis* NBRC 100480	79.2	[75.2 - 82.6]	76.8	[73.8 - 79.6]	81.6	[78.3 - 84.6]	0.05
**'Enterococcus faecalis C144 (ST228).fna'**	*Enterococcus faecalis* NBRC 100480	79.7	[75.8 - 83.2]	76.6	[73.6 - 79.3]	82.1	[78.8 - 85.0]	0.06
**'Enterococcus faecalis C146 (ST228).fna'**	*Enterococcus faecalis* NBRC 100480	79.8	[75.8 - 83.2]	76.6	[73.6 - 79.4]	82.1	[78.8 - 85.0]	0.07
**'Enterococcus faecalis C138 (ST228).fna'**	*Enterococcus faecalis* NBRC 100480	79.8	[75.9 - 83.3]	76.6	[73.6 - 79.4]	82.2	[78.8 - 85.1]	0.08
**'Enterococcus faecalis R30 (ST228).fna'**	*Enterococcus faecalis* NBRC 100480	81.8	[78.0 - 85.2]	76.4	[73.4 - 79.1]	83.8	[80.6 - 86.6]	0.18
**'Enterococcus faecalis C116 (ST228).fna'**	*Enterococcus faecalis* NBRC 100480	79.5	[75.5 - 82.9]	76.4	[73.4 - 79.1]	81.8	[78.5 - 84.8]	0.07
**'Enterococcus faecalis CVM N54548 (ST228).fna'**	*Enterococcus faecalis* NBRC 100480	80.2	[76.2 - 83.6]	76.4	[73.4 - 79.1]	82.4	[79.1 - 85.3]	0.04
**'Enterococcus faecalis CVM N55265 (ST228).fna'**	*Enterococcus faecalis* NBRC 100480	80.4	[76.5 - 83.8]	76.3	[73.3 - 79.1]	82.6	[79.3 - 85.5]	0.07
**'Enterococcus faecalis CVM N52587 (STx).fna'**	*Enterococcus faecalis* NBRC 100480	79.5	[75.6 - 83.0]	76.3	[73.3 - 79.0]	81.9	[78.5 - 84.8]	0.03
**'Enterococcus faecalis CVM N52662 (ST228).fna'**	*Enterococcus faecalis* NBRC 100480	78.2	[74.2 - 81.7]	76.3	[73.3 - 79.0]	80.7	[77.3 - 83.7]	0.01
**'Enterococcus faecalis EN24 (ST228).fna'**	*Enterococcus faecalis* NBRC 100480	78.6	[74.7 - 82.1]	76.1	[73.1 - 78.9]	81.1	[77.7 - 84.0]	0.02
**'Enterococcus faecalis R48 (ST228).fna'**	*Enterococcus faecalis* NBRC 100480	78.7	[74.7 - 82.2]	76.1	[73.1 - 78.9]	81.1	[77.8 - 84.1]	0.04
**'Enterococcus faecalis G81 (ST1468).fna'**	*Enterococcus faecalis* NBRC 100480	78.3	[74.3 - 81.8]	75.9	[72.9 - 78.7]	80.7	[77.4 - 83.7]	0.03

Query	Subject	d_0	C.I. d_0	d_4	C.I. d_4	d_6	C.I. d_6	Diff. G+C Percent
**'Enterococcus faecalis CVM N52467 (ST228).fna'**	*Enterococcus faecalis* NBRC 100480	82.8	[79.0 - 86.1]	75.9	[72.9 - 78.6]	84.5	[81.3 - 87.3]	0.1
**'Enterococcus faecalis CVM N53420 (ST228).fna'**	*Enterococcus faecalis* NBRC 100480	76.4	[72.4 - 79.9]	75.4	[72.4 - 78.2]	79.0	[75.5 - 82.0]	0.08
**'Enterococcus faecalis R48 (ST228).fna'**	*Aequorivita lutea* q18	12.8	[10.1 - 16.1]	72.3	[69.3 - 75.1]	13.2	[10.9 - 16.0]	5.09
**'Enterococcus faecalis C116 (ST228).fna'**	*Aequorivita lutea* q18	12.8	[10.1 - 16.1]	71.4	[68.4 - 74.2]	13.2	[10.9 - 16.0]	5.07
**'Enterococcus faecalis C144 (ST228).fna'**	*Aequorivita lutea* q18	12.8	[10.1 - 16.1]	70.9	[67.9 - 73.7]	13.2	[10.9 - 16.0]	5.07
**'Enterococcus faecalis R30 (ST228).fna'**	*Aequorivita lutea* q18	12.8	[10.1 - 16.1]	70.7	[67.6 - 73.5]	13.2	[10.9 - 16.0]	4.95
**'Enterococcus faecalis DSM 111623 (ST624).fna'**	*Aequorivita lutea* q18	12.8	[10.1 - 16.1]	70.6	[67.6 - 73.4]	13.2	[10.9 - 16.0]	5.18
**'Enterococcus faecalis CVM N52467 (ST228).fna'**	*Aequorivita lutea* q18	12.8	[10.1 - 16.1]	70.0	[67.0 - 72.9]	13.2	[10.9 - 16.0]	5.03
**'Enterococcus faecalis G81 (ST1468).fna'**	*Aequorivita lutea* q18	12.8	[10.1 - 16.1]	70.0	[67.0 - 72.8]	13.2	[10.9 - 16.0]	5.1
**'Enterococcus faecalis C138 (ST228).fna'**	*Aequorivita lutea* q18	12.8	[10.1 - 16.1]	70.0	[66.9 - 72.8]	13.2	[10.9 - 16.0]	5.06
**'Enterococcus faecalis CVM N52587 (STx).fna'**	*Aequorivita lutea* q18	12.8	[10.1 - 16.1]	69.9	[66.9 - 72.7]	13.2	[10.9 - 16.0]	5.16
**'Enterococcus faecalis C146 (ST228).fna'**	*Aequorivita lutea* q18	12.8	[10.1 - 16.1]	69.9	[66.9 - 72.8]	13.2	[10.9 - 16.0]	5.06
**'Enterococcus faecalis CVM N53420 (ST228).fna'**	*Aequorivita lutea* q18	12.8	[10.1 - 16.1]	69.8	[66.8 - 72.7]	13.2	[10.9 - 16.0]	5.21
**'Enterococcus faecalis CVM N55265 (ST228).fna'**	*Aequorivita lutea* q18	12.8	[10.1 - 16.1]	69.7	[66.7 - 72.6]	13.2	[10.9 - 16.0]	5.07
**'Enterococcus faecalis CVM N52662 (ST228).fna'**	*Aequorivita lutea* q18	12.8	[10.1 - 16.1]	69.7	[66.7 - 72.6]	13.2	[10.9 - 16.0]	5.13
**'Enterococcus faecalis CVM N54548 (ST228).fna'**	*Aequorivita lutea* q18	12.8	[10.1 - 16.1]	69.6	[66.6 - 72.5]	13.2	[10.9 - 16.0]	5.09
**'Enterococcus faecalis EN24 (ST228).fna'**	*Aequorivita lutea* q18	12.8	[10.1 - 16.1]	69.4	[66.4 - 72.2]	13.2	[10.9 - 16.0]	5.12
**'Enterococcus faecalis 209EA1 (ST624).fna'**	*Aequorivita lutea* q18	12.8	[10.1 - 16.1]	61.4	[58.6 - 64.2]	13.3	[10.9 - 16.0]	4.92
**'Enterococcus faecalis CVM N53420 (ST228).fna'**	*Vagococcus martis* D7T301	13.1	[10.4 - 16.4]	30.3	[27.9 - 32.8]	13.5	[11.1 - 16.3]	3.68
**'Enterococcus faecalis CVM N52662 (ST228).fna'**	*Vagococcus martis* D7T301	13.1	[10.4 - 16.4]	30.1	[27.7 - 32.6]	13.5	[11.1 - 16.3]	3.77
**'Enterococcus faecalis R48 (ST228).fna'**	*Enterococcus durans* NBRC 100479	13.5	[10.7 - 16.8]	28.8	[26.4 - 31.3]	13.9	[11.5 - 16.7]	0.23
**'Enterococcus faecalis EN24 (ST228).fna'**	*Vagococcus martis* D7T301	13.1	[10.4 - 16.4]	28.4	[26.1 - 30.9]	13.5	[11.1 - 16.3]	3.78
**'Enterococcus faecalis CVM N52587 (STx).fna'**	*Enterococcus durans* NBRC 100479	13.6	[10.8 - 16.9]	28.3	[26.0 - 30.8]	14.0	[11.5 - 16.8]	0.3
**'Enterococcus faecalis G81 (ST1468).fna'**	*Enterococcus saigonensis* JCM 31193	13.5	[10.8 - 16.9]	28.0	[25.6 - 30.5]	13.9	[11.5 - 16.7]	1.27

Query	Subject	d_0	C.I. d_0	d_4	C.I. d_4	d_6	C.I. d_6	Diff. G+C Percent
**'Enterococcus faecalis 209EA1 (ST624).fna'**	*Vagococcus vulneris* SS1995	13.1	[10.3 - 16.4]	27.9	[25.5 - 30.4]	13.5	[11.1 - 16.2]	3.12
**'Enterococcus faecalis 209EA1 (ST624).fna'**	*Vagococcus bubulae* SS1994	13.4	[10.6 - 16.7]	27.7	[25.3 - 30.2]	13.8	[11.4 - 16.6]	4.18
**'Enterococcus faecalis CVM N52587 (STx).fna'**	*Enterococcus massiliensis* AM1	13.4	[10.6 - 16.7]	27.7	[25.4 - 30.2]	13.8	[11.4 - 16.5]	2.15
**'Enterococcus faecalis CVM N55265 (ST228).fna'**	*Enterococcus durans* NBRC 100479	13.4	[10.7 - 16.8]	27.6	[25.2 - 30.1]	13.8	[11.4 - 16.6]	0.2
**'Enterococcus faecalis CVM N52662 (ST228).fna'**	*Enterococcus durans* NBRC 100479	13.4	[10.7 - 16.7]	27.6	[25.2 - 30.1]	13.8	[11.4 - 16.6]	0.27
**'Enterococcus faecalis 209EA1 (ST624).fna'**	*Enterococcus durans* NBRC 100479	13.4	[10.7 - 16.8]	27.5	[25.1 - 30.0]	13.8	[11.4 - 16.6]	0.06
**'Enterococcus faecalis R48 (ST228).fna'**	*Enterococcus massiliensis* AM1	13.3	[10.6 - 16.6]	27.5	[25.2 - 30.0]	13.7	[11.3 - 16.5]	2.08
**'Enterococcus faecalis C144 (ST228).fna'**	*Enterococcus durans* NBRC 100479	13.5	[10.7 - 16.8]	27.5	[25.2 - 30.0]	13.8	[11.4 - 16.6]	0.21
**'Enterococcus faecalis EN24 (ST228).fna'**	*Candidatus* Enterococcus avicola CHK172-16539	13.3	[10.5 - 16.6]	27.4	[25.1 - 29.9]	13.7	[11.3 - 16.5]	0.66
**'Enterococcus faecalis C146 (ST228).fna'**	*Enterococcus durans* NBRC 100479	13.5	[10.7 - 16.8]	27.4	[25.0 - 29.9]	13.8	[11.4 - 16.6]	0.2
**'Enterococcus faecalis 209EA1 (ST624).fna'**	*Enterococcus massiliensis* AM1	13.5	[10.7 - 16.8]	27.3	[25.0 - 29.8]	13.8	[11.4 - 16.6]	1.91
**'Enterococcus faecalis CVM N52587 (STx).fna'**	*Candidatus* Enterococcus avicola CHK172-16539	13.3	[10.5 - 16.6]	27.2	[24.8 - 29.7]	13.7	[11.3 - 16.5]	0.62
**'Enterococcus faecalis C138 (ST228).fna'**	*Enterococcus durans* NBRC 100479	13.5	[10.7 - 16.8]	27.2	[24.9 - 29.7]	13.8	[11.4 - 16.6]	0.19
**'Enterococcus faecalis EN24 (ST228).fna'**	*Enterococcus casseliflavus* NBRC 100478	13.1	[10.4 - 16.4]	27.2	[24.8 - 29.7]	13.5	[11.1 - 16.3]	4.82
**'Enterococcus faecalis CVM N52662 (ST228).fna'**	*Candidatus* Enterococcus avicola CHK172-16539	13.3	[10.5 - 16.6]	27.1	[24.7 - 29.6]	13.6	[11.3 - 16.4]	0.65
**'Enterococcus faecalis G81 (ST1468).fna'**	*Candidatus* Enterococcus avicola CHK172-16539	13.4	[10.6 - 16.7]	27.0	[24.6 - 29.5]	13.8	[11.4 - 16.6]	0.67
**'Enterococcus faecalis CVM N55265 (ST228).fna'**	*Candidatus* Enterococcus avicola CHK172-16539	13.3	[10.5 - 16.6]	27.0	[24.7 - 29.5]	13.7	[11.3 - 16.4]	0.71
**'Enterococcus faecalis C144 (ST228).fna'**	*Candidatus* Enterococcus avicola CHK172-16539	13.3	[10.5 - 16.6]	26.9	[24.5 - 29.4]	13.7	[11.3 - 16.5]	0.71
**'Enterococcus faecalis C146 (ST228).fna'**	*Candidatus* Enterococcus avicola CHK172-16539	13.3	[10.5 - 16.6]	26.9	[24.6 - 29.4]	13.7	[11.3 - 16.5]	0.72
**'Enterococcus faecalis C138 (ST228).fna'**	*Candidatus* Enterococcus avicola CHK172-16539	13.3	[10.5 - 16.6]	26.9	[24.5 - 29.4]	13.7	[11.3 - 16.5]	0.72
**'Enterococcus faecalis CVM N52587 (STx).fna'**	*Enterococcus saigonensis* JCM 31193	13.4	[10.7 - 16.8]	26.9	[24.6 - 29.4]	13.8	[11.4 - 16.6]	1.22
**'Enterococcus faecalis R48 (ST228).fna'**	*Enterococcus saigonensis* JCM 31193	13.4	[10.7 - 16.7]	26.9	[24.6 - 29.4]	13.8	[11.4 - 16.6]	1.29
**'Enterococcus faecalis R48 (ST228).fna'**	*Candidatus* Enterococcus avicola CHK172-16539	13.3	[10.5 - 16.6]	26.9	[24.5 - 29.4]	13.6	[11.3 - 16.4]	0.69
**'Enterococcus faecalis CVM N53420 (ST228).fna'**	*Candidatus* Enterococcus avicola CHK172-16539	13.2	[10.5 - 16.6]	26.8	[24.4 - 29.3]	13.6	[11.2 - 16.4]	0.57
**'Enterococcus faecalis R30 (ST228).fna'**	*Candidatus* Enterococcus avicola CHK172-16539	13.3	[10.5 - 16.6]	26.8	[24.4 - 29.3]	13.7	[11.3 - 16.4]	0.83

Query	Subject	d_0	C.I. d_0	d_4	C.I. d_4	d_6	C.I. d_6	Diff. G+C Percent
**'Enterococcus faecalis CVM N52467 (ST228).fna'**	*Candidatus* Enterococcus avicola CHK172-16539	13.3	[10.5 - 16.6]	26.8	[24.5 - 29.3]	13.7	[11.3 - 16.4]	0.75
**'Enterococcus faecalis CVM N54548 (ST228).fna'**	*Candidatus* Enterococcus avicola CHK172-16539	13.3	[10.5 - 16.6]	26.8	[24.5 - 29.3]	13.6	[11.3 - 16.4]	0.69
**'Enterococcus faecalis 209EA1 (ST624).fna'**	*Enterococcus casseliflavus* NBRC 100478	13.1	[10.4 - 16.4]	26.8	[24.5 - 29.3]	13.5	[11.1 - 16.3]	4.62
**'Enterococcus faecalis G81 (ST1468).fna'**	*Enterococcus massiliensis* AM1	13.5	[10.8 - 16.8]	26.8	[24.4 - 29.3]	13.9	[11.5 - 16.7]	2.1
**'Enterococcus faecalis C116 (ST228).fna'**	*Candidatus* Enterococcus avicola CHK172-16539	13.3	[10.5 - 16.6]	26.8	[24.4 - 29.2]	13.7	[11.3 - 16.4]	0.71
**'Enterococcus faecalis 209EA1 (ST624).fna'**	*Enterococcus saigonensis* JCM 31193	13.5	[10.7 - 16.8]	26.7	[24.3 - 29.2]	13.9	[11.5 - 16.7]	1.46
**'Enterococcus faecalis CVM N52587 (STx).fna'**	*Vagococcus vulneris* SS1995	13.0	[10.3 - 16.3]	26.6	[24.2 - 29.0]	13.4	[11.1 - 16.2]	2.88
**'Enterococcus faecalis G81 (ST1468).fna'**	*Vagococcus vulneris* SS1995	13.0	[10.3 - 16.3]	26.5	[24.1 - 28.9]	13.4	[11.0 - 16.1]	2.94
**'Enterococcus faecalis 209EA1 (ST624).fna'**	*Enterococcus mediterraneensis* Marseille-P4358	13.3	[10.6 - 16.7]	26.4	[24.0 - 28.9]	13.7	[11.3 - 16.5]	3.16
**'Enterococcus faecalis G81 (ST1468).fna'**	*Vagococcus bubulae* SS1994	13.3	[10.6 - 16.6]	26.3	[23.9 - 28.8]	13.7	[11.3 - 16.5]	4.0
**'Enterococcus faecalis G81 (ST1468).fna'**	*Enterococcus porcinus* ATCC 700913	13.7	[10.9 - 17.0]	26.3	[24.0 - 28.8]	14.0	[11.6 - 16.9]	2.53
**'Enterococcus faecalis C146 (ST228).fna'**	*Enterococcus casseliflavus* NBRC 100478	13.1	[10.4 - 16.4]	26.2	[23.9 - 28.7]	13.5	[11.1 - 16.2]	4.76
**'Enterococcus faecalis CVM N52467 (ST228).fna'**	*Enterococcus durans* NBRC 100479	13.5	[10.7 - 16.8]	26.2	[23.9 - 28.7]	13.9	[11.5 - 16.7]	0.17
**'Enterococcus faecalis C144 (ST228).fna'**	*Enterococcus casseliflavus* NBRC 100478	13.1	[10.4 - 16.4]	26.2	[23.8 - 28.6]	13.5	[11.1 - 16.2]	4.77
**'Enterococcus faecalis EN24 (ST228).fna'**	*Enterococcus durans* NBRC 100479	13.4	[10.6 - 16.7]	26.1	[23.7 - 28.6]	13.8	[11.4 - 16.6]	0.26
**'Enterococcus faecalis G81 (ST1468).fna'**	*Enterococcus mediterraneensis* Marseille-P4358	13.4	[10.6 - 16.7]	26.1	[23.8 - 28.6]	13.7	[11.3 - 16.5]	3.34
**'Enterococcus faecalis R48 (ST228).fna'**	*Vagococcus martis* D7T301	13.0	[10.3 - 16.3]	26.0	[23.7 - 28.5]	13.4	[11.0 - 16.2]	3.8
**'Enterococcus faecalis C146 (ST228).fna'**	*Enterococcus massiliensis* AM1	13.3	[10.5 - 16.6]	26.0	[23.6 - 28.5]	13.6	[11.3 - 16.4]	2.05
**'Enterococcus faecalis DSM 111623 (ST624).fna'**	*Candidatus* Enterococcus avicola CHK172-16539	13.3	[10.6 - 16.6]	26.0	[23.7 - 28.5]	13.7	[11.3 - 16.5]	0.6
**'Enterococcus faecalis 209EA1 (ST624).fna'**	*Vagococcus martis* D7T301	13.1	[10.4 - 16.4]	26.0	[23.7 - 28.5]	13.5	[11.1 - 16.3]	3.97
**'Enterococcus faecalis C138 (ST228).fna'**	*Enterococcus casseliflavus* NBRC 100478	13.1	[10.4 - 16.4]	26.0	[23.6 - 28.5]	13.5	[11.1 - 16.2]	4.76
**'Enterococcus faecalis R48 (ST228).fna'**	*Enterococcus casseliflavus* NBRC 100478	13.1	[10.3 - 16.4]	25.9	[23.6 - 28.4]	13.4	[11.1 - 16.2]	4.79
**'Enterococcus faecalis C144 (ST228).fna'**	*Enterococcus massiliensis* AM1	13.3	[10.5 - 16.6]	25.9	[23.6 - 28.4]	13.6	[11.3 - 16.4]	2.06
**'Enterococcus faecalis EN24 (ST228).fna'**	*Enterococcus massiliensis* AM1	13.3	[10.5 - 16.6]	25.8	[23.5 - 28.3]	13.6	[11.3 - 16.4]	2.11

Query	Subject	d_0	C.I. d_0	d_4	C.I. d_4	d_6	C.I. d_6	Diff. G+C Percent
**'Enterococcus faecalis C138 (ST228).fna'**	*Enterococcus massiliensis* AM1	13.3	[10.5 - 16.6]	25.8	[23.4 - 28.3]	13.6	[11.3 - 16.4]	2.05
**'Enterococcus faecalis CVM N52467 (ST228).fna'**	*Enterococcus casseliflavus* NBRC 100478	13.1	[10.4 - 16.4]	25.8	[23.4 - 28.2]	13.5	[11.1 - 16.2]	4.73
**'Enterococcus faecalis CVM N52662 (ST228).fna'**	*Enterococcus saigonensis* JCM 31193	13.5	[10.7 - 16.8]	25.8	[23.5 - 28.3]	13.8	[11.4 - 16.6]	1.25
**'Enterococcus faecalis CVM N55265 (ST228).fna'**	*Enterococcus saigonensis* JCM 31193	13.4	[10.6 - 16.7]	25.8	[23.5 - 28.3]	13.7	[11.4 - 16.5]	1.31
**'Enterococcus faecalis 209EA1 (ST624).fna'**	*Enterococcus porcinus* ATCC 700913	13.6	[10.9 - 17.0]	25.8	[23.5 - 28.3]	14.0	[11.6 - 16.8]	2.71
**'Enterococcus faecalis 209EA1 (ST624).fna'**	*Candidatus* Enterococcus avicola CHK172-16539	13.4	[10.6 - 16.7]	25.7	[23.3 - 28.1]	13.7	[11.3 - 16.5]	0.86
**'Enterococcus faecalis CVM N52467 (ST228).fna'**	*Enterococcus massiliensis* AM1	13.2	[10.5 - 16.6]	25.7	[23.4 - 28.2]	13.6	[11.2 - 16.4]	2.02
**'Enterococcus faecalis CVM N54548 (ST228).fna'**	*Enterococcus mediterraneensis* Marseille-P4358	13.3	[10.6 - 16.6]	25.7	[23.4 - 28.2]	13.7	[11.3 - 16.5]	3.32
**'Enterococcus faecalis CVM N52587 (STx).fna'**	*Vagococcus bubulae* SS1994	13.3	[10.6 - 16.6]	25.6	[23.3 - 28.1]	13.7	[11.3 - 16.5]	3.94
**'Enterococcus faecalis C146 (ST228).fna'**	*Enterococcus lactis* DSM 23655	13.3	[10.5 - 16.6]	25.5	[23.2 - 28.0]	13.6	[11.3 - 16.4]	0.54
**'Enterococcus faecalis 209EA1 (ST624).fna'**	*Enterococcus lactis* CCM 8412	13.3	[10.5 - 16.6]	25.4	[23.0 - 27.8]	13.6	[11.2 - 16.4]	0.39
**'Enterococcus faecalis C144 (ST228).fna'**	*Enterococcus lactis* DSM 23655	13.3	[10.5 - 16.6]	25.4	[23.0 - 27.8]	13.6	[11.3 - 16.4]	0.55
**'Enterococcus faecalis C138 (ST228).fna'**	*Enterococcus lactis* DSM 23655	13.3	[10.5 - 16.6]	25.4	[23.0 - 27.8]	13.6	[11.3 - 16.4]	0.54
**'Enterococcus faecalis G81 (ST1468).fna'**	*Enterococcus casseliflavus* NBRC 100478	13.0	[10.3 - 16.3]	25.4	[23.1 - 27.9]	13.4	[11.1 - 16.2]	4.81
**'Enterococcus faecalis C146 (ST228).fna'**	*Enterococcus lactis* CCM 8412	13.3	[10.5 - 16.6]	25.4	[23.1 - 27.9]	13.6	[11.3 - 16.4]	0.53
**'Enterococcus faecalis CVM N53420 (ST228).fna'**	*Enterococcus saigonensis* JCM 31193	13.4	[10.7 - 16.8]	25.4	[23.0 - 27.8]	13.8	[11.4 - 16.6]	1.16
**'Enterococcus faecalis C138 (ST228).fna'**	*Enterococcus lactis* CCM 8412	13.3	[10.5 - 16.6]	25.4	[23.0 - 27.9]	13.6	[11.3 - 16.4]	0.53
**'Enterococcus faecalis G81 (ST1468).fna'**	*Enterococcus durans* NBRC 100479	13.3	[10.6 - 16.7]	25.3	[23.0 - 27.8]	13.7	[11.3 - 16.5]	0.24
**'Enterococcus faecalis G81 (ST1468).fna'**	*Enterococcus songbeiensis* NCIMB 15179	13.2	[10.5 - 16.6]	25.3	[23.0 - 27.8]	13.6	[11.2 - 16.4]	2.18
**'Enterococcus faecalis 209EA1 (ST624).fna'**	*Enterococcus thailandicus* DSM 21767	13.5	[10.7 - 16.8]	25.3	[23.0 - 27.8]	13.8	[11.4 - 16.6]	1.13
**'Enterococcus faecalis CVM N53420 (ST228).fna'**	*Enterococcus casseliflavus* NBRC 100478	13.0	[10.3 - 16.3]	25.3	[22.9 - 27.7]	13.4	[11.1 - 16.2]	4.91
**'Enterococcus faecalis CVM N52587 (STx).fna'**	*Enterococcus lactis* CCM 8412	13.2	[10.5 - 16.5]	25.2	[22.8 - 27.6]	13.6	[11.2 - 16.4]	0.63
**'Enterococcus faecalis EN24 (ST228).fna'**	*Enterococcus saigonensis* JCM 31193	13.4	[10.7 - 16.8]	25.2	[22.9 - 27.7]	13.8	[11.4 - 16.6]	1.26
**'Enterococcus faecalis CVM N52587 (STx).fna'**	*Enterococcus lactis* DSM 23655	13.2	[10.5 - 16.5]	25.2	[22.9 - 27.7]	13.6	[11.2 - 16.4]	0.64

Query	Subject	d_0	C.I. d_0	d_4	C.I. d_4	d_6	C.I. d_6	Diff. G+C Percent
**'Enterococcus faecalis C144 (ST228).fna'**	*Enterococcus lactis* CCM 8412	13.3	[10.5 - 16.6]	25.2	[22.9 - 27.7]	13.6	[11.2 - 16.4]	0.54
**'Enterococcus faecalis C146 (ST228).fna'**	*Enterococcus saigonensis* JCM 31193	13.4	[10.6 - 16.7]	25.2	[22.8 - 27.7]	13.7	[11.3 - 16.5]	1.32
**'Enterococcus faecalis CVM N53420 (ST228).fna'**	*Enterococcus durans* NBRC 100479	13.3	[10.6 - 16.6]	25.2	[22.9 - 27.7]	13.7	[11.3 - 16.5]	0.35
**'Enterococcus faecalis CVM N52587 (STx).fna'**	*Enterococcus casseliflavus* NBRC 100478	13.0	[10.3 - 16.3]	25.1	[22.8 - 27.6]	13.4	[11.1 - 16.2]	4.86
**'Enterococcus faecalis G81 (ST1468).fna'**	*Enterococcus thailandicus* DSM 21767	13.4	[10.7 - 16.8]	25.1	[22.8 - 27.6]	13.8	[11.4 - 16.6]	0.94
**'Enterococcus faecalis CVM N52587 (STx).fna'**	*Enterococcus porcinus* ATCC 700913	13.6	[10.9 - 17.0]	25.1	[22.8 - 27.6]	14.0	[11.6 - 16.8]	2.47
**'Enterococcus faecalis CVM N52467 (ST228).fna'**	*Enterococcus lactis* DSM 23655	13.2	[10.5 - 16.6]	25.1	[22.8 - 27.6]	13.6	[11.2 - 16.4]	0.51
**'Enterococcus faecalis C144 (ST228).fna'**	*Enterococcus saigonensis* JCM 31193	13.4	[10.6 - 16.7]	25.1	[22.8 - 27.6]	13.7	[11.3 - 16.5]	1.31
**'Enterococcus faecalis CVM N52467 (ST228).fna'**	*Vagococcus martis* D7T301	13.0	[10.3 - 16.3]	25.0	[22.6 - 27.4]	13.3	[11.0 - 16.1]	3.86
**'Enterococcus faecalis C138 (ST228).fna'**	*Enterococcus saigonensis* JCM 31193	13.4	[10.6 - 16.7]	25.0	[22.7 - 27.5]	13.7	[11.3 - 16.5]	1.32
**'Enterococcus faecalis CVM N52467 (ST228).fna'**	*Enterococcus lactis* CCM 8412	13.2	[10.5 - 16.6]	25.0	[22.7 - 27.5]	13.6	[11.2 - 16.4]	0.5
**'Enterococcus faecalis CVM N52467 (ST228).fna'**	*Enterococcus saigonensis* JCM 31193	13.3	[10.6 - 16.7]	25.0	[22.7 - 27.5]	13.7	[11.3 - 16.5]	1.35
**'Enterococcus faecalis CVM N54548 (ST228).fna'**	*Enterococcus lactis* DSM 23655	13.2	[10.5 - 16.5]	25.0	[22.6 - 27.4]	13.6	[11.2 - 16.4]	0.57
**'Enterococcus faecalis 209EA1 (ST624).fna'**	*Enterococcus lactis* DSM 23655	13.2	[10.5 - 16.6]	25.0	[22.7 - 27.5]	13.6	[11.2 - 16.4]	0.4
**'Enterococcus faecalis R30 (ST228).fna'**	*Enterococcus massiliensis* AM1	13.2	[10.5 - 16.6]	24.9	[22.6 - 27.3]	13.6	[11.2 - 16.4]	1.94
**'Enterococcus faecalis CVM N54548 (ST228).fna'**	*Enterococcus casseliflavus* NBRC 100478	13.0	[10.3 - 16.3]	24.9	[22.6 - 27.4]	13.4	[11.0 - 16.2]	4.79
**'Enterococcus faecalis CVM N55265 (ST228).fna'**	*Enterococcus casseliflavus* NBRC 100478	13.0	[10.3 - 16.3]	24.9	[22.6 - 27.4]	13.4	[11.0 - 16.2]	4.77
**'Enterococcus faecalis CVM N54548 (ST228).fna'**	*Enterococcus lactis* CCM 8412	13.2	[10.5 - 16.5]	24.9	[22.6 - 27.4]	13.6	[11.2 - 16.4]	0.56
**'Enterococcus faecalis CVM N52662 (ST228).fna'**	*Enterococcus casseliflavus* NBRC 100478	13.0	[10.3 - 16.3]	24.9	[22.5 - 27.3]	13.4	[11.0 - 16.2]	4.83
**'Enterococcus faecalis CVM N53420 (ST228).fna'**	*Enterococcus massiliensis* AM1	13.2	[10.5 - 16.5]	24.8	[22.4 - 27.2]	13.6	[11.2 - 16.4]	2.21
**'Enterococcus faecalis R48 (ST228).fna'**	*Enterococcus thailandicus* DSM 21767	13.4	[10.7 - 16.7]	24.8	[22.4 - 27.2]	13.8	[11.4 - 16.6]	0.96
**'Enterococcus faecalis 209EA1 (ST624).fna'**	*Enterococcus dispar* ATCC 51266	13.5	[10.7 - 16.8]	24.8	[22.5 - 27.3]	13.9	[11.5 - 16.7]	0.56
**'Enterococcus faecalis C116 (ST228).fna'**	*Enterococcus massiliensis* AM1	13.2	[10.5 - 16.5]	24.8	[22.5 - 27.3]	13.6	[11.2 - 16.4]	2.06

Query	Subject	d_0	C.I. d_0	d_4	C.I. d_4	d_6	C.I. d_6	Diff. G+C Percent
**'Enterococcus faecalis C146 (ST228).fna'**	*Vagococcus martis* D7T301	13.0	[10.3 - 16.3]	24.7	[22.4 - 27.2]	13.4	[11.0 - 16.1]	3.83
**'Enterococcus faecalis C146 (ST228).fna'**	*Enterococcus xiangfangensis* DSM 105127	13.2	[10.5 - 16.6]	24.7	[22.4 - 27.2]	13.6	[11.2 - 16.4]	1.43
**'Enterococcus faecalis R30 (ST228).fna'**	*Enterococcus durans* NBRC 100479	13.3	[10.6 - 16.7]	24.7	[22.4 - 27.2]	13.7	[11.3 - 16.5]	0.09
**'Enterococcus faecalis CVM N55265 (ST228).fna'**	*Vagococcus martis* D7T301	13.0	[10.3 - 16.3]	24.7	[22.4 - 27.2]	13.4	[11.0 - 16.1]	3.83
**'Enterococcus faecalis C144 (ST228).fna'**	*Enterococcus xiangfangensis* DSM 105127	13.2	[10.5 - 16.6]	24.7	[22.4 - 27.2]	13.6	[11.2 - 16.4]	1.44
**'Enterococcus faecalis C144 (ST228).fna'**	*Enterococcus xiangfangensis* NCIMB 14834	13.2	[10.5 - 16.6]	24.7	[22.3 - 27.1]	13.6	[11.2 - 16.4]	1.43
**'Enterococcus faecalis C146 (ST228).fna'**	*Enterococcus xiangfangensis* NCIMB 14834	13.2	[10.5 - 16.5]	24.7	[22.3 - 27.1]	13.6	[11.2 - 16.4]	1.42
**'Enterococcus faecalis R30 (ST228).fna'**	*Vagococcus martis* D7T301	13.0	[10.3 - 16.3]	24.7	[22.3 - 27.1]	13.4	[11.0 - 16.1]	3.94
**'Enterococcus faecalis G81 (ST1468).fna'**	*Enterococcus dispar* ATCC 51266	13.6	[10.8 - 16.9]	24.6	[22.3 - 27.0]	13.9	[11.5 - 16.7]	0.38
**'Enterococcus faecalis C138 (ST228).fna'**	*Vagococcus martis* D7T301	13.0	[10.3 - 16.3]	24.6	[22.2 - 27.0]	13.4	[11.0 - 16.1]	3.84
**'Enterococcus faecalis CVM N54548 (ST228).fna'**	*Vagococcus martis* D7T301	13.0	[10.3 - 16.3]	24.6	[22.3 - 27.0]	13.4	[11.0 - 16.1]	3.8
**'Enterococcus faecalis CVM N52587 (STx).fna'**	*Enterococcus thailandicus* DSM 21767	13.4	[10.7 - 16.7]	24.6	[22.3 - 27.1]	13.8	[11.4 - 16.6]	0.89
**'Enterococcus faecalis C116 (ST228).fna'**	*Enterococcus durans* NBRC 100479	13.3	[10.6 - 16.7]	24.6	[22.3 - 27.1]	13.7	[11.3 - 16.5]	0.21
**'Enterococcus faecalis CVM N55265 (ST228).fna'**	*Enterococcus massiliensis* AM1	13.2	[10.5 - 16.5]	24.6	[22.3 - 27.1]	13.6	[11.2 - 16.4]	2.06
**'Enterococcus faecalis CVM N52662 (ST228).fna'**	*Enterococcus massiliensis* AM1	13.2	[10.5 - 16.5]	24.6	[22.3 - 27.1]	13.6	[11.2 - 16.4]	2.12
**'Enterococcus faecalis CVM N52662 (ST228).fna'**	*Enterococcus lactis* DSM 23655	13.2	[10.5 - 16.5]	24.6	[22.3 - 27.1]	13.6	[11.2 - 16.4]	0.61
**'Enterococcus faecalis CVM N52587 (STx).fna'**	*Enterococcus mediterraneensis* Marseille-P4358	13.3	[10.5 - 16.6]	24.6	[22.3 - 27.1]	13.6	[11.3 - 16.4]	3.4
**'Enterococcus faecalis C144 (ST228).fna'**	*Vagococcus martis* D7T301	13.0	[10.3 - 16.3]	24.6	[22.3 - 27.1]	13.4	[11.0 - 16.1]	3.82
**'Enterococcus faecalis CVM N55265 (ST228).fna'**	*Enterococcus lactis* DSM 23655	13.2	[10.5 - 16.5]	24.6	[22.3 - 27.1]	13.6	[11.2 - 16.4]	0.55
**'Enterococcus faecalis CVM N54548 (ST228).fna'**	*Enterococcus durans* NBRC 100479	13.3	[10.6 - 16.6]	24.5	[22.2 - 27.0]	13.7	[11.3 - 16.5]	0.23
**'Enterococcus faecalis CVM N52587 (STx).fna'**	*Vagococcus martis* D7T301	13.0	[10.3 - 16.3]	24.5	[22.2 - 27.0]	13.4	[11.0 - 16.1]	3.73
**'Enterococcus faecalis C146 (ST228).fna'**	*Enterococcus mediterraneensis* Marseille-P4358	13.3	[10.5 - 16.6]	24.5	[22.2 - 27.0]	13.6	[11.3 - 16.4]	3.3
**'Enterococcus faecalis CVM N52662 (ST228).fna'**	*Enterococcus lactis* CCM 8412	13.2	[10.5 - 16.5]	24.5	[22.2 - 27.0]	13.6	[11.2 - 16.4]	0.6

Query	Subject	d_0	C.I. d_0	d_4	C.I. d_4	d_6	C.I. d_6	Diff. G+C Percent
**'Enterococcus faecalis CVM N52467 (ST228).fna'**	*Enterococcus xiangfangensis* DSM 105127	13.2	[10.5 - 16.6]	24.5	[22.2 - 27.0]	13.6	[11.2 - 16.4]	1.4
**'Enterococcus faecalis R48 (ST228).fna'**	*Vagococcus vulneris* SS1995	13.0	[10.3 - 16.2]	24.5	[22.2 - 27.0]	13.3	[11.0 - 16.1]	2.95
**'Enterococcus faecalis EN24 (ST228).fna'**	*Enterococcus lactis* DSM 23655	13.2	[10.5 - 16.5]	24.5	[22.1 - 26.9]	13.6	[11.2 - 16.3]	0.6
**'Enterococcus faecalis CVM N55265 (ST228).fna'**	*Enterococcus lactis* CCM 8412	13.2	[10.5 - 16.5]	24.5	[22.2 - 27.0]	13.6	[11.2 - 16.4]	0.54
**'Enterococcus faecalis C144 (ST228).fna'**	*Enterococcus mediterraneensis* Marseille-P4358	13.3	[10.5 - 16.6]	24.5	[22.2 - 27.0]	13.6	[11.3 - 16.4]	3.3
**'Enterococcus faecalis C138 (ST228).fna'**	*Enterococcus xiangfangensis* DSM 105127	13.2	[10.5 - 16.6]	24.5	[22.2 - 27.0]	13.6	[11.2 - 16.4]	1.43
**'Enterococcus faecalis C116 (ST228).fna'**	*Vagococcus martis* D7T301	13.0	[10.3 - 16.3]	24.5	[22.2 - 27.0]	13.3	[11.0 - 16.1]	3.82
**'Enterococcus faecalis 209EA1 (ST624).fna'**	*Enterococcus xiangfangensis* NCIMB 14834	13.2	[10.5 - 16.5]	24.4	[22.1 - 26.9]	13.6	[11.2 - 16.4]	1.28
**'Enterococcus faecalis CVM N52467 (ST228).fna'**	*Enterococcus mediterraneensis* Marseille-P4358	13.3	[10.5 - 16.6]	24.4	[22.1 - 26.9]	13.6	[11.2 - 16.4]	3.27
**'Enterococcus faecalis C138 (ST228).fna'**	*Enterococcus mediterraneensis* Marseille-P4358	13.3	[10.5 - 16.6]	24.4	[22.1 - 26.9]	13.6	[11.2 - 16.4]	3.29
**'Enterococcus faecalis CVM N52467 (ST228).fna'**	*Enterococcus xiangfangensis* NCIMB 14834	13.2	[10.5 - 16.5]	24.4	[22.1 - 26.9]	13.6	[11.2 - 16.4]	1.39
**'Enterococcus faecalis C138 (ST228).fna'**	*Enterococcus xiangfangensis* NCIMB 14834	13.2	[10.5 - 16.5]	24.4	[22.1 - 26.9]	13.6	[11.2 - 16.4]	1.41
**'Enterococcus faecalis 209EA1 (ST624).fna'**	*Enterococcus xiangfangensis* DSM 105127	13.2	[10.5 - 16.5]	24.3	[22.0 - 26.8]	13.6	[11.2 - 16.4]	1.29
**'Enterococcus faecalis EN24 (ST228).fna'**	*Enterococcus lactis* CCM 8412	13.2	[10.5 - 16.5]	24.3	[22.0 - 26.8]	13.6	[11.2 - 16.3]	0.59
**'Enterococcus faecalis 209EA1 (ST624).fna'**	*Enterococcus canintestini* DSM 21207	13.3	[10.6 - 16.6]	24.2	[21.9 - 26.7]	13.7	[11.3 - 16.4]	1.48
**'Enterococcus faecalis CVM N54548 (ST228).fna'**	*Enterococcus saigonensis* JCM 31193	13.3	[10.6 - 16.6]	24.2	[21.9 - 26.7]	13.7	[11.3 - 16.5]	1.29
**'Enterococcus faecalis R48 (ST228).fna'**	*Enterococcus lactis* DSM 23655	13.2	[10.5 - 16.5]	24.2	[21.9 - 26.6]	13.6	[11.2 - 16.3]	0.57
**'Enterococcus faecalis CVM N53420 (ST228).fna'**	*Enterococcus lactis* DSM 23655	13.2	[10.4 - 16.5]	24.2	[21.9 - 26.6]	13.5	[11.2 - 16.3]	0.69
**'Enterococcus faecalis CVM N54548 (ST228).fna'**	*Enterococcus massiliensis* AM1	13.2	[10.5 - 16.5]	24.2	[21.9 - 26.7]	13.6	[11.2 - 16.3]	2.08
**'Enterococcus faecalis R48 (ST228).fna'**	*Enterococcus lactis* CCM 8412	13.2	[10.5 - 16.5]	24.2	[21.9 - 26.6]	13.6	[11.2 - 16.3]	0.56
**'Enterococcus faecalis G81 (ST1468).fna'**	*Vagococcus martis* D7T301	13.0	[10.3 - 16.3]	24.1	[21.8 - 26.6]	13.4	[11.0 - 16.1]	3.79
**'Enterococcus faecalis G81 (ST1468).fna'**	*Enterococcus xiangfangensis* NCIMB 14834	13.1	[10.4 - 16.5]	24.1	[21.8 - 26.5]	13.5	[11.1 - 16.3]	1.46
**'Enterococcus faecalis G81 (ST1468).fna'**	*Enterococcus xiangfangensis* DSM 105127	13.2	[10.4 - 16.5]	24.1	[21.8 - 26.6]	13.5	[11.2 - 16.3]	1.47

Query	Subject	d_0	C.I. d_0	d_4	C.I. d_4	d_6	C.I. d_6	Diff. G+C Percent
**'Enterococcus faecalis 209EA1 (ST624).fna'**	*Enterococcus songbeiensis* NCIMB 15179	13.3	[10.5 - 16.6]	24.1	[21.8 - 26.6]	13.6	[11.2 - 16.4]	2.0
**'Enterococcus faecalis CVM N53420 (ST228).fna'**	*Enterococcus lactis* CCM 8412	13.2	[10.4 - 16.5]	24.1	[21.8 - 26.6]	13.5	[11.2 - 16.3]	0.69
**'Enterococcus faecalis CVM N52662 (ST228).fna'**	*Enterococcus lemanii* DSM 105069	13.4	[10.7 - 16.8]	24.1	[21.8 - 26.6]	13.8	[11.4 - 16.6]	0.3
**'Enterococcus faecalis EN24 (ST228).fna'**	*Enterococcus mediterraneensis* Marseille-P4358	13.3	[10.5 - 16.6]	24.1	[21.8 - 26.6]	13.6	[11.2 - 16.4]	3.35
**'Enterococcus faecalis R48 (ST228).fna'**	*Enterococcus mediterraneensis* Marseille-P4358	13.2	[10.5 - 16.5]	23.9	[21.6 - 26.4]	13.6	[11.2 - 16.4]	3.33
**'Enterococcus faecalis CVM N53420 (ST228).fna'**	*Enterococcus mediterraneensis* Marseille-P4358	13.2	[10.5 - 16.5]	23.9	[21.6 - 26.4]	13.6	[11.2 - 16.4]	3.45
**'Enterococcus faecalis CVM N55265 (ST228).fna'**	*Enterococcus mediterraneensis* Marseille-P4358	13.2	[10.5 - 16.5]	23.9	[21.6 - 26.3]	13.6	[11.2 - 16.4]	3.3
**'Enterococcus faecalis C116 (ST228).fna'**	*Enterococcus casseliflavus* NBRC 100478	13.0	[10.3 - 16.3]	23.8	[21.5 - 26.2]	13.4	[11.0 - 16.2]	4.77
**'Enterococcus faecalis CVM N53420 (ST228).fna'**	*Enterococcus lemanii* DSM 105069	13.4	[10.7 - 16.8]	23.8	[21.5 - 26.2]	13.8	[11.4 - 16.6]	0.22
**'Enterococcus faecalis R30 (ST228).fna'**	*Enterococcus casseliflavus* NBRC 100478	13.0	[10.3 - 16.3]	23.8	[21.5 - 26.3]	13.4	[11.0 - 16.2]	4.65
**'Enterococcus faecalis CVM N52662 (ST228).fna'**	*Enterococcus mediterraneensis* Marseille-P4358	13.2	[10.5 - 16.5]	23.8	[21.5 - 26.3]	13.6	[11.2 - 16.4]	3.36
**'Enterococcus faecalis EN24 (ST228).fna'**	*Enterococcus thailandicus* DSM 21767	13.4	[10.6 - 16.7]	23.7	[21.4 - 26.2]	13.7	[11.3 - 16.5]	0.93
**'Enterococcus faecalis C144 (ST228).fna'**	*Vagococcus vulneris* SS1995	12.9	[10.2 - 16.2]	23.7	[21.4 - 26.1]	13.3	[11.0 - 16.1]	2.97
**'Enterococcus faecalis CVM N52467 (ST228).fna'**	*Enterococcus dispar* ATCC 51266	13.4	[10.6 - 16.7]	23.7	[21.4 - 26.2]	13.7	[11.4 - 16.5]	0.45
**'Enterococcus faecalis DSM 111623 (ST624).fna'**	*Enterococcus casseliflavus* NBRC 100478	13.0	[10.3 - 16.3]	23.7	[21.4 - 26.2]	13.4	[11.0 - 16.2]	4.89
**'Enterococcus faecalis C146 (ST228).fna'**	*Vagococcus vulneris* SS1995	12.9	[10.2 - 16.2]	23.6	[21.4 - 26.1]	13.3	[11.0 - 16.1]	2.98
**'Enterococcus faecalis C146 (ST228).fna'**	*Enterococcus porcinus* ATCC 700913	13.5	[10.7 - 16.8]	23.6	[21.3 - 26.1]	13.8	[11.4 - 16.6]	2.57
**'Enterococcus faecalis C144 (ST228).fna'**	*Enterococcus porcinus* ATCC 700913	13.5	[10.7 - 16.8]	23.6	[21.3 - 26.1]	13.8	[11.4 - 16.6]	2.56
**'Enterococcus faecalis R30 (ST228).fna'**	*Enterococcus saigonensis* JCM 31193	13.3	[10.5 - 16.6]	23.6	[21.3 - 26.1]	13.6	[11.3 - 16.4]	1.43
**'Enterococcus faecalis EN24 (ST228).fna'**	*Vagococcus vulneris* SS1995	12.9	[10.2 - 16.2]	23.6	[21.3 - 26.1]	13.3	[11.0 - 16.1]	2.93
**'Enterococcus faecalis C138 (ST228).fna'**	*Enterococcus porcinus* ATCC 700913	13.5	[10.7 - 16.8]	23.5	[21.2 - 26.0]	13.8	[11.4 - 16.6]	2.57
**'Enterococcus faecalis DSM 111623 (ST624).fna'**	*Enterococcus mediterraneensis* Marseille-P4358	13.2	[10.5 - 16.5]	23.5	[21.2 - 26.0]	13.5	[11.2 - 16.3]	3.42
**'Enterococcus faecalis CVM N53420 (ST228).fna'**	*Vagococcus bubulae* SS1994	13.2	[10.5 - 16.5]	23.5	[21.2 - 25.9]	13.6	[11.2 - 16.3]	3.89

Query	Subject	d_0	C.I. d_0	d_4	C.I. d_4	d_6	C.I. d_6	Diff. G+C Percent
**'Enterococcus faecalis CVM N55265 (ST228).fna'**	*Enterococcus dispar* ATCC 51266	13.4	[10.6 - 16.7]	23.5	[21.2 - 26.0]	13.7	[11.3 - 16.5]	0.41
**'Enterococcus faecalis C116 (ST228).fna'**	*Enterococcus saigonensis* JCM 31193	13.3	[10.5 - 16.6]	23.5	[21.2 - 25.9]	13.6	[11.2 - 16.4]	1.31
**'Enterococcus faecalis R48 (ST228).fna'**	*Enterococcus porcinus* ATCC 700913	13.5	[10.7 - 16.8]	23.5	[21.2 - 26.0]	13.8	[11.4 - 16.6]	2.54
**'Enterococcus faecalis C144 (ST228).fna'**	*Enterococcus dispar* ATCC 51266	13.4	[10.7 - 16.7]	23.5	[21.2 - 25.9]	13.8	[11.4 - 16.6]	0.41
**'Enterococcus faecalis CVM N52587 (STx).fna'**	*Enterococcus dispar* ATCC 51266	13.4	[10.6 - 16.7]	23.5	[21.2 - 25.9]	13.7	[11.3 - 16.5]	0.32
**'Enterococcus faecalis CVM N52662 (ST228).fna'**	*Enterococcus dispar* ATCC 51266	13.4	[10.6 - 16.7]	23.5	[21.2 - 26.0]	13.7	[11.3 - 16.5]	0.35
**'Enterococcus faecalis C146 (ST228).fna'**	*Enterococcus dispar* ATCC 51266	13.4	[10.7 - 16.7]	23.5	[21.2 - 26.0]	13.8	[11.4 - 16.6]	0.42
**'Enterococcus faecalis R30 (ST228).fna'**	*Enterococcus lactis* DSM 23655	13.2	[10.5 - 16.5]	23.4	[21.1 - 25.8]	13.5	[11.2 - 16.3]	0.43
**'Enterococcus faecalis R30 (ST228).fna'**	*Enterococcus mediterraneensis* Marseille-P4358	13.2	[10.5 - 16.5]	23.4	[21.1 - 25.9]	13.6	[11.2 - 16.4]	3.19
**'Enterococcus faecalis C138 (ST228).fna'**	*Vagococcus vulneris* SS1995	12.9	[10.2 - 16.2]	23.4	[21.1 - 25.9]	13.3	[11.0 - 16.1]	2.99
**'Enterococcus faecalis R30 (ST228).fna'**	*Enterococcus lactis* CCM 8412	13.2	[10.5 - 16.5]	23.4	[21.1 - 25.9]	13.5	[11.2 - 16.3]	0.42
**'Enterococcus faecalis CVM N52467 (ST228).fna'**	*Vagococcus vulneris* SS1995	12.9	[10.2 - 16.2]	23.4	[21.1 - 25.9]	13.3	[11.0 - 16.1]	3.01
**'Enterococcus faecalis C138 (ST228).fna'**	*Enterococcus dispar* ATCC 51266	13.4	[10.7 - 16.7]	23.4	[21.1 - 25.9]	13.8	[11.4 - 16.6]	0.42
**'Enterococcus faecalis R48 (ST228).fna'**	*Enterococcus xiangfangensis* DSM 105127	13.2	[10.4 - 16.5]	23.3	[21.0 - 25.8]	13.5	[11.2 - 16.3]	1.46
**'Enterococcus faecalis CVM N53420 (ST228).fna'**	*Vagococcus vulneris* SS1995	12.9	[10.2 - 16.2]	23.3	[21.0 - 25.7]	13.3	[11.0 - 16.1]	2.83
**'Enterococcus faecalis DSM 111623 (ST624).fna'**	*Vagococcus martis* D7T301	13.0	[10.3 - 16.3]	23.3	[21.0 - 25.7]	13.4	[11.0 - 16.1]	3.71
**'Enterococcus faecalis CVM N55265 (ST228).fna'**	*Enterococcus xiangfangensis* DSM 105127	13.2	[10.4 - 16.5]	23.3	[21.0 - 25.7]	13.5	[11.2 - 16.3]	1.44
**'Enterococcus faecalis G81 (ST1468).fna'**	*Enterococcus lactis* DSM 23655	13.1	[10.4 - 16.4]	23.3	[21.0 - 25.8]	13.5	[11.1 - 16.3]	0.58
**'Enterococcus faecalis R30 (ST228).fna'**	*Enterococcus thailandicus* DSM 21767	13.4	[10.6 - 16.7]	23.2	[21.0 - 25.7]	13.7	[11.3 - 16.5]	1.1
**'Enterococcus faecalis C116 (ST228).fna'**	*Enterococcus mediterraneensis* Marseille-P4358	13.2	[10.5 - 16.5]	23.2	[20.9 - 25.7]	13.6	[11.2 - 16.4]	3.3
**'Enterococcus faecalis CVM N53420 (ST228).fna'**	*Enterococcus thailandicus* DSM 21767	13.3	[10.6 - 16.6]	23.2	[20.9 - 25.6]	13.7	[11.3 - 16.5]	0.83
**'Enterococcus faecalis G81 (ST1468).fna'**	*Enterococcus lactis* CCM 8412	13.1	[10.4 - 16.4]	23.2	[20.9 - 25.6]	13.5	[11.1 - 16.3]	0.58
**'Enterococcus faecalis EN24 (ST228).fna'**	*Enterococcus cecorum* DSM 20682	13.3	[10.5 - 16.6]	23.2	[20.9 - 25.7]	13.6	[11.2 - 16.4]	1.16
**'Enterococcus faecalis CVM N52587 (STx).fna'**	*Enterococcus xiangfangensis* NCIMB 14834	13.2	[10.4 - 16.5]	23.2	[20.9 - 25.6]	13.5	[11.2 - 16.3]	1.52

Query	Subject	d_0	C.I. d_0	d_4	C.I. d_4	d_6	C.I. d_6	Diff. G+C Percent
**'Enterococcus faecalis C116 (ST228).fna'**	*Enterococcus lactis* DSM 23655	13.2	[10.4 - 16.5]	23.2	[20.9 - 25.7]	13.5	[11.2 - 16.3]	0.55
**'Enterococcus faecalis R48 (ST228).fna'**	*Enterococcus xiangfangensis* NCIMB 14834	13.2	[10.4 - 16.5]	23.2	[20.9 - 25.6]	13.5	[11.1 - 16.3]	1.45
**'Enterococcus faecalis C116 (ST228).fna'**	*Enterococcus lactis* CCM 8412	13.2	[10.4 - 16.5]	23.2	[20.9 - 25.7]	13.5	[11.2 - 16.3]	0.54
**'Enterococcus faecalis CVM N52467 (ST228).fna'**	*Enterococcus porcinus* ATCC 700913	13.5	[10.8 - 16.9]	23.2	[21.0 - 25.7]	13.9	[11.5 - 16.7]	2.6
**'Enterococcus faecalis C116 (ST228).fna'**	*Enterococcus thailandicus* DSM 21767	13.3	[10.6 - 16.7]	23.2	[20.9 - 25.6]	13.7	[11.3 - 16.5]	0.98
**'Enterococcus faecalis EN24 (ST228).fna'**	*Enterococcus porcinus* ATCC 700913	13.6	[10.8 - 16.9]	23.2	[20.9 - 25.7]	13.9	[11.5 - 16.7]	2.51
**'Enterococcus faecalis CVM N55265 (ST228).fna'**	*Enterococcus xiangfangensis* NCIMB 14834	13.2	[10.4 - 16.5]	23.2	[20.9 - 25.6]	13.5	[11.2 - 16.3]	1.42
**'Enterococcus faecalis CVM N52587 (STx).fna'**	*Enterococcus xiangfangensis* DSM 105127	13.2	[10.4 - 16.5]	23.2	[21.0 - 25.7]	13.5	[11.2 - 16.3]	1.53
**'Enterococcus faecalis G81 (ST1468).fna'**	*Enterococcus canintestini* DSM 21207	13.3	[10.5 - 16.6]	23.2	[20.9 - 25.7]	13.6	[11.2 - 16.4]	1.3
**'Enterococcus faecalis R48 (ST228).fna'**	*Enterococcus songbeiensis* NCIMB 15179	13.2	[10.5 - 16.5]	23.1	[20.8 - 25.6]	13.5	[11.2 - 16.3]	2.17
**'Enterococcus faecalis CVM N54548 (ST228).fna'**	*Vagococcus vulneris* SS1995	12.9	[10.2 - 16.2]	23.1	[20.8 - 25.5]	13.3	[11.0 - 16.1]	2.95
**'Enterococcus faecalis CVM N55265 (ST228).fna'**	*Enterococcus porcinus* ATCC 700913	13.4	[10.7 - 16.8]	23.1	[20.8 - 25.5]	13.8	[11.4 - 16.6]	2.56
**'Enterococcus faecalis CVM N54548 (ST228).fna'**	*Enterococcus porcinus* ATCC 700913	13.5	[10.7 - 16.8]	23.1	[20.8 - 25.6]	13.8	[11.4 - 16.6]	2.54
**'Enterococcus faecalis CVM N55265 (ST228).fna'**	*Vagococcus vulneris* SS1995	12.9	[10.2 - 16.2]	23.1	[20.8 - 25.6]	13.3	[11.0 - 16.1]	2.98
**'Enterococcus faecalis C144 (ST228).fna'**	*Enterococcus thailandicus* DSM 21767	13.3	[10.6 - 16.6]	23.0	[20.7 - 25.5]	13.7	[11.3 - 16.5]	0.98
**'Enterococcus faecalis C146 (ST228).fna'**	*Enterococcus thailandicus* DSM 21767	13.3	[10.6 - 16.6]	23.0	[20.7 - 25.5]	13.7	[11.3 - 16.5]	0.99
**'Enterococcus faecalis R30 (ST228).fna'**	*Enterococcus dispar* ATCC 51266	13.4	[10.6 - 16.7]	23.0	[20.7 - 25.5]	13.7	[11.3 - 16.5]	0.53
**'Enterococcus faecalis CVM N52662 (ST228).fna'**	*Vagococcus vulneris* SS1995	12.9	[10.2 - 16.2]	23.0	[20.8 - 25.5]	13.3	[11.0 - 16.1]	2.91
**'Enterococcus faecalis CVM N53420 (ST228).fna'**	*Enterococcus xiangfangensis* DSM 105127	13.2	[10.5 - 16.5]	23.0	[20.7 - 25.4]	13.6	[11.2 - 16.4]	1.58
**'Enterococcus faecalis C138 (ST228).fna'**	*Enterococcus thailandicus* DSM 21767	13.3	[10.6 - 16.6]	23.0	[20.7 - 25.4]	13.7	[11.3 - 16.5]	0.99
**'Enterococcus faecalis CVM N53420 (ST228).fna'**	*Enterococcus dispar* ATCC 51266	13.4	[10.6 - 16.7]	23.0	[20.7 - 25.4]	13.7	[11.3 - 16.5]	0.27
**'Enterococcus faecalis CVM N54548 (ST228).fna'**	*Enterococcus dispar* ATCC 51266	13.4	[10.6 - 16.7]	23.0	[20.7 - 25.4]	13.7	[11.3 - 16.5]	0.39
**'Enterococcus faecalis R48 (ST228).fna'**	*Enterococcus dispar* ATCC 51266	13.3	[10.6 - 16.7]	22.9	[20.7 - 25.4]	13.7	[11.3 - 16.5]	0.39

Query	Subject	d_0	C.I. d_0	d_4	C.I. d_4	d_6	C.I. d_6	Diff. G+C Percent
**'Enterococcus faecalis 209EA1 (ST624).fna'**	*Enterococcus cecorum* DSM 20682	13.2	[10.4 - 16.5]	22.9	[20.6 - 25.3]	13.5	[11.1 - 16.3]	1.36
**'Enterococcus faecalis CVM N52467 (ST228).fna'**	*Enterococcus thailandicus* DSM 21767	13.3	[10.6 - 16.6]	22.9	[20.7 - 25.4]	13.7	[11.3 - 16.5]	1.02
**'Enterococcus faecalis EN24 (ST228).fna'**	*Enterococcus dispar* ATCC 51266	13.4	[10.6 - 16.7]	22.9	[20.6 - 25.3]	13.7	[11.3 - 16.5]	0.36
**'Enterococcus faecalis R30 (ST228).fna'**	*Vagococcus vulneris* SS1995	12.9	[10.2 - 16.2]	22.9	[20.6 - 25.3]	13.3	[11.0 - 16.1]	3.09
**'Enterococcus faecalis EN24 (ST228).fna'**	*Enterococcus lemanii* DSM 105069	13.4	[10.7 - 16.7]	22.8	[20.6 - 25.3]	13.7	[11.4 - 16.5]	0.32
**'Enterococcus faecalis 209EA1 (ST624).fna'**	*Enterococcus lemanii* DSM 105069	13.3	[10.6 - 16.6]	22.8	[20.5 - 25.2]	13.6	[11.3 - 16.4]	0.51
**'Enterococcus faecalis CVM N53420 (ST228).fna'**	*Enterococcus porcinus* ATCC 700913	13.5	[10.7 - 16.8]	22.8	[20.5 - 25.2]	13.8	[11.4 - 16.6]	2.42
**'Enterococcus faecalis 209EA1 (ST624).fna'**	*Enterococcus rotai* LMG 26678	14.2	[11.4 - 17.6]	22.8	[20.5 - 25.2]	14.5	[12.1 - 17.3]	1.6
**'Enterococcus faecalis CVM N52662 (ST228).fna'**	*Enterococcus xiangfangensis* DSM 105127	13.2	[10.5 - 16.6]	22.8	[20.5 - 25.3]	13.6	[11.2 - 16.4]	1.5
**'Enterococcus faecalis CVM N52662 (ST228).fna'**	*Enterococcus xiangfangensis* NCIMB 14834	13.2	[10.5 - 16.5]	22.8	[20.5 - 25.2]	13.6	[11.2 - 16.4]	1.49
**'Enterococcus faecalis C116 (ST228).fna'**	*Vagococcus vulneris* SS1995	12.9	[10.2 - 16.2]	22.8	[20.5 - 25.2]	13.3	[10.9 - 16.1]	2.97
**'Enterococcus faecalis CVM N52662 (ST228).fna'**	*Enterococcus porcinus* ATCC 700913	13.5	[10.7 - 16.8]	22.8	[20.5 - 25.3]	13.8	[11.4 - 16.6]	2.5
**'Enterococcus faecalis C116 (ST228).fna'**	*Enterococcus dispar* ATCC 51266	13.4	[10.6 - 16.7]	22.8	[20.5 - 25.3]	13.7	[11.3 - 16.5]	0.41
**'Enterococcus faecalis CVM N54548 (ST228).fna'**	*Enterococcus xiangfangensis* DSM 105127	13.2	[10.4 - 16.5]	22.7	[20.4 - 25.2]	13.5	[11.1 - 16.3]	1.46
**'Enterococcus faecalis C146 (ST228).fna'**	*Enterococcus songbeiensis* NCIMB 15179	13.2	[10.5 - 16.5]	22.7	[20.4 - 25.2]	13.5	[11.2 - 16.3]	2.14
**'Enterococcus faecalis CVM N52662 (ST228).fna'**	*Enterococcus thailandicus* DSM 21767	13.3	[10.6 - 16.6]	22.7	[20.4 - 25.1]	13.7	[11.3 - 16.4]	0.92
**'Enterococcus faecalis CVM N55265 (ST228).fna'**	*Enterococcus songbeiensis* NCIMB 15179	13.2	[10.4 - 16.5]	22.7	[20.4 - 25.1]	13.5	[11.2 - 16.3]	2.15
**'Enterococcus faecalis CVM N53420 (ST228).fna'**	*Enterococcus xiangfangensis* NCIMB 14834	13.2	[10.5 - 16.5]	22.7	[20.5 - 25.2]	13.6	[11.2 - 16.4]	1.57
**'Enterococcus faecalis CVM N55265 (ST228).fna'**	*Enterococcus thailandicus* DSM 21767	13.3	[10.6 - 16.6]	22.7	[20.4 - 25.1]	13.7	[11.3 - 16.5]	0.98
**'Enterococcus faecalis G81 (ST1468).fna'**	*Enterococcus hermanniensis* DSM 17122	13.4	[10.7 - 16.7]	22.7	[20.4 - 25.2]	13.8	[11.4 - 16.6]	0.19
**'Enterococcus faecalis 209EA1 (ST624).fna'**	*Enterococcus wangshanyuanii* MN05	14.1	[11.3 - 17.5]	22.7	[20.4 - 25.2]	14.4	[12.0 - 17.2]	0.46
**'Enterococcus faecalis C144 (ST228).fna'**	*Enterococcus songbeiensis* NCIMB 15179	13.2	[10.5 - 16.5]	22.6	[20.4 - 25.1]	13.5	[11.2 - 16.3]	2.15
**'Enterococcus faecalis CVM N54548 (ST228).fna'**	*Enterococcus thailandicus* DSM 21767	13.3	[10.6 - 16.6]	22.6	[20.4 - 25.1]	13.7	[11.3 - 16.5]	0.96

Query	Subject	d_0	C.I. d_0	d_4	C.I. d_4	d_6	C.I. d_6	Diff. G+C Percent
**'Enterococcus faecalis 209EA1 (ST624).fna'**	*Enterococcus hermanniensis* DSM 17122	13.4	[10.7 - 16.7]	22.6	[20.3 - 25.1]	13.8	[11.4 - 16.6]	0.38
**'Enterococcus faecalis DSM 111623 (ST624).fna'**	*Enterococcus canintestini* DSM 21207	13.2	[10.5 - 16.5]	22.6	[20.4 - 25.1]	13.6	[11.2 - 16.4]	1.22
**'Enterococcus faecalis DSM 111623 (ST624).fna'**	*Enterococcus lactis* CCM 8412	13.1	[10.4 - 16.4]	22.6	[20.3 - 25.0]	13.5	[11.1 - 16.3]	0.66
**'Enterococcus faecalis R48 (ST228).fna'**	*Vagococcus bubulae* SS1994	13.2	[10.5 - 16.5]	22.6	[20.3 - 25.1]	13.5	[11.2 - 16.3]	4.01
**'Enterococcus faecalis 209EA1 (ST624).fna'**	*Enterococcus rotai* DSM 102982	14.3	[11.5 - 17.7]	22.6	[20.3 - 25.0]	14.6	[12.1 - 17.4]	1.65
**'Enterococcus faecalis CVM N52587 (STx).fna'**	*Enterococcus songbeiensis* NCIMB 15179	13.2	[10.4 - 16.5]	22.6	[20.3 - 25.1]	13.5	[11.2 - 16.3]	2.24
**'Enterococcus faecalis DSM 111623 (ST624).fna'**	*Enterococcus saigonensis* JCM 31193	13.3	[10.5 - 16.6]	22.6	[20.4 - 25.1]	13.6	[11.2 - 16.4]	1.19
**'Enterococcus faecalis C138 (ST228).fna'**	*Enterococcus songbeiensis* NCIMB 15179	13.2	[10.5 - 16.5]	22.6	[20.3 - 25.0]	13.5	[11.2 - 16.3]	2.14
**'Enterococcus faecalis CVM N54548 (ST228).fna'**	*Enterococcus xiangfangensis* NCIMB 14834	13.1	[10.4 - 16.4]	22.6	[20.4 - 25.1]	13.5	[11.1 - 16.3]	1.45
**'Enterococcus faecalis DSM 111623 (ST624).fna'**	*Enterococcus lactis* DSM 23655	13.1	[10.4 - 16.4]	22.6	[20.3 - 25.0]	13.5	[11.1 - 16.2]	0.66
**'Enterococcus faecalis DSM 111623 (ST624).fna'**	*Vagococcus vulneris* SS1995	12.9	[10.2 - 16.2]	22.5	[20.2 - 25.0]	13.3	[10.9 - 16.0]	2.86
**'Enterococcus faecalis DSM 111623 (ST624).fna'**	*Enterococcus durans* NBRC 100479	13.2	[10.5 - 16.5]	22.5	[20.2 - 24.9]	13.5	[11.2 - 16.3]	0.32
**'Enterococcus faecalis G81 (ST1468).fna'**	*Enterococcus lemanii* DSM 105069	13.3	[10.5 - 16.6]	22.5	[20.3 - 25.0]	13.6	[11.2 - 16.4]	0.33
**'Enterococcus faecalis 209EA1 (ST624).fna'**	*Enterococcus moraviensis* ATCC BAA-383	14.1	[11.3 - 17.5]	22.5	[20.2 - 25.0]	14.4	[11.9 - 17.2]	1.76
**'Enterococcus faecalis DSM 111623 (ST624).fna'**	*Enterococcus dispar* ATCC 51266	13.3	[10.6 - 16.7]	22.4	[20.1 - 24.9]	13.7	[11.3 - 16.5]	0.3
**'Enterococcus faecalis R30 (ST228).fna'**	*Enterococcus porcinus* ATCC 700913	13.5	[10.7 - 16.8]	22.4	[20.1 - 24.8]	13.8	[11.4 - 16.6]	2.68
**'Enterococcus faecalis CVM N54548 (ST228).fna'**	*Enterococcus songbeiensis* NCIMB 15179	13.2	[10.4 - 16.5]	22.4	[20.1 - 24.8]	13.5	[11.2 - 16.3]	2.17
**'Enterococcus faecalis CVM N52467 (ST228).fna'**	*Enterococcus songbeiensis* NCIMB 15179	13.2	[10.4 - 16.5]	22.4	[20.2 - 24.9]	13.5	[11.2 - 16.3]	2.11
**'Enterococcus faecalis CVM N52467 (ST228).fna'**	*Vagococcus bubulae* SS1994	13.1	[10.4 - 16.4]	22.3	[20.0 - 24.7]	13.5	[11.1 - 16.3]	4.07
**'Enterococcus faecalis G81 (ST1468).fna'**	*Enterococcus cecorum* DSM 20682	13.1	[10.4 - 16.4]	22.3	[20.1 - 24.8]	13.5	[11.1 - 16.2]	1.17
**'Enterococcus faecalis CVM N52662 (ST228).fna'**	*Enterococcus songbeiensis* NCIMB 15179	13.2	[10.5 - 16.6]	22.3	[20.0 - 24.7]	13.6	[11.2 - 16.4]	2.21
**'Enterococcus faecalis G81 (ST1468).fna'**	*Enterococcus rotai* LMG 26678	14.1	[11.3 - 17.5]	22.2	[19.9 - 24.6]	14.4	[11.9 - 17.2]	1.42

Query	Subject	d_0	C.I. d_0	d_4	C.I. d_4	d_6	C.I. d_6	Diff. G+C Percent
**'Enterococcus faecalis 209EA1 (ST624).fna'**	*Enterococcus haemoperoxidus* ATCC BAA-382	14.5	[11.7 - 17.9]	22.2	[19.9 - 24.6]	14.7	[12.3 - 17.6]	2.0
**'Enterococcus faecalis C116 (ST228).fna'**	*Enterococcus porcinus* ATCC 700913	13.4	[10.7 - 16.8]	22.2	[19.9 - 24.6]	13.8	[11.4 - 16.6]	2.56
**'Enterococcus faecalis R30 (ST228).fna'**	*Enterococcus songbeiensis* NCIMB 15179	13.2	[10.4 - 16.5]	22.2	[19.9 - 24.6]	13.5	[11.2 - 16.3]	2.03
**'Enterococcus faecalis 209EA1 (ST624).fna'**	*Enterococcus silesiacus* LMG 23085	14.0	[11.2 - 17.3]	22.2	[20.0 - 24.7]	14.2	[11.8 - 17.1]	1.32
**'Enterococcus faecalis DSM 111623 (ST624).fna'**	*Enterococcus xiangfangensis* DSM 105127	13.1	[10.4 - 16.4]	22.2	[20.0 - 24.7]	13.5	[11.1 - 16.3]	1.55
**'Enterococcus faecalis EN24 (ST228).fna'**	*Enterococcus xiangfangensis* DSM 105127	13.2	[10.5 - 16.5]	22.2	[19.9 - 24.6]	13.5	[11.2 - 16.3]	1.49
**'Enterococcus faecalis EN24 (ST228).fna'**	*Enterococcus xiangfangensis* NCIMB 14834	13.2	[10.5 - 16.5]	22.1	[19.8 - 24.5]	13.5	[11.2 - 16.3]	1.47
**'Enterococcus faecalis DSM 111623 (ST624).fna'**	*Enterococcus rotai* LMG 26678	14.2	[11.3 - 17.5]	22.1	[19.8 - 24.6]	14.4	[12.0 - 17.3]	1.34
**'Enterococcus faecalis DSM 111623 (ST624).fna'**	*Enterococcus porcinus* ATCC 700913	13.4	[10.6 - 16.7]	22.1	[19.9 - 24.6]	13.7	[11.3 - 16.5]	2.45
**'Enterococcus faecalis C116 (ST228).fna'**	*Enterococcus songbeiensis* NCIMB 15179	13.2	[10.4 - 16.5]	22.1	[19.9 - 24.6]	13.5	[11.1 - 16.3]	2.15
**'Enterococcus faecalis C116 (ST228).fna'**	*Enterococcus xiangfangensis* DSM 105127	13.1	[10.4 - 16.4]	22.1	[19.8 - 24.6]	13.5	[11.1 - 16.3]	1.44
**'Enterococcus faecalis DSM 111623 (ST624).fna'**	*Enterococcus songbeiensis* NCIMB 15179	13.1	[10.4 - 16.5]	22.1	[19.9 - 24.6]	13.5	[11.1 - 16.3]	2.26
**'Enterococcus faecalis 209EA1 (ST624).fna'**	*Enterococcus ureasiticus* DSM 23328	14.3	[11.5 - 17.7]	22.1	[19.9 - 24.6]	14.5	[12.1 - 17.4]	1.92
**'Enterococcus faecalis R30 (ST228).fna'**	*Enterococcus xiangfangensis* DSM 105127	13.1	[10.4 - 16.4]	22.1	[19.9 - 24.6]	13.5	[11.1 - 16.3]	1.32
**'Enterococcus faecalis DSM 111623 (ST624).fna'**	*Enterococcus thailandicus* DSM 21767	13.3	[10.5 - 16.6]	22.1	[19.8 - 24.5]	13.6	[11.2 - 16.4]	0.86
**'Enterococcus faecalis CVM N52467 (ST228).fna'**	*Enterococcus rotai* LMG 26678	14.2	[11.4 - 17.6]	22.0	[19.7 - 24.4]	14.4	[12.0 - 17.3]	1.49
**'Enterococcus faecalis C146 (ST228).fna'**	*Vagococcus bubulae* SS1994	13.2	[10.4 - 16.5]	22.0	[19.7 - 24.4]	13.5	[11.2 - 16.3]	4.04
**'Enterococcus faecalis CVM N52662 (ST228).fna'**	*Vagococcus bubulae* SS1994	13.2	[10.4 - 16.5]	22.0	[19.7 - 24.4]	13.5	[11.1 - 16.3]	3.97
**'Enterococcus faecalis CVM N52662 (ST228).fna'**	*Enterococcus rotai* LMG 26678	14.2	[11.3 - 17.5]	22.0	[19.7 - 24.4]	14.4	[12.0 - 17.3]	1.4
**'Enterococcus faecalis DSM 111623 (ST624).fna'**	*Enterococcus xiangfangensis* NCIMB 14834	13.1	[10.4 - 16.4]	22.0	[19.7 - 24.5]	13.5	[11.1 - 16.2]	1.54
**'Enterococcus faecalis CVM N55265 (ST228).fna'**	*Vagococcus bubulae* SS1994	13.2	[10.4 - 16.5]	22.0	[19.8 - 24.5]	13.5	[11.2 - 16.3]	4.04
**'Enterococcus faecalis DSM 111623 (ST624).fna'**	*Enterococcus cecorum* DSM 20682	13.1	[10.4 - 16.4]	22.0	[19.7 - 24.4]	13.5	[11.1 - 16.2]	1.09

Query	Subject	d_0	C.I. d_0	d_4	C.I. d_4	d_6	C.I. d_6	Diff. G+C Percent
**'Enterococcus faecalis 209EA1 (ST624).fna'**	*Enterococcus rivorum* LMG 25899	13.9	[11.1 - 17.2]	22.0	[19.7 - 24.4]	14.2	[11.8 - 17.0]	2.83
**'Enterococcus faecalis CVM N53420 (ST228).fna'**	*Enterococcus songbeiensis* NCIMB 15179	13.2	[10.5 - 16.6]	22.0	[19.7 - 24.4]	13.6	[11.2 - 16.4]	2.29
**'Enterococcus faecalis CVM N52587 (STx).fna'**	*Enterococcus rotai* LMG 26678	14.2	[11.4 - 17.5]	22.0	[19.7 - 24.4]	14.4	[12.0 - 17.3]	1.36
**'Enterococcus faecalis EN24 (ST228).fna'**	*Enterococcus songbeiensis* NCIMB 15179	13.2	[10.5 - 16.5]	22.0	[19.7 - 24.4]	13.6	[11.2 - 16.4]	2.2
**'Enterococcus faecalis CVM N55265 (ST228).fna'**	*Enterococcus rotai* LMG 26678	14.2	[11.4 - 17.6]	22.0	[19.7 - 24.4]	14.4	[12.0 - 17.3]	1.46
**'Enterococcus faecalis R30 (ST228).fna'**	*Enterococcus xiangfangensis* NCIMB 14834	13.1	[10.4 - 16.4]	22.0	[19.8 - 24.5]	13.5	[11.1 - 16.3]	1.31
**'Enterococcus faecalis G81 (ST1468).fna'**	*Enterococcus rotai* DSM 102982	14.2	[11.4 - 17.6]	22.0	[19.8 - 24.5]	14.4	[12.0 - 17.3]	1.47
**'Enterococcus faecalis R30 (ST228).fna'**	*Enterococcus rotai* LMG 26678	14.2	[11.4 - 17.6]	21.9	[19.7 - 24.4]	14.5	[12.0 - 17.3]	1.57
**'Enterococcus faecalis 209EA1 (ST624).fna'**	*Enterococcus rivorum* DSM 104544	13.9	[11.1 - 17.2]	21.9	[19.6 - 24.3]	14.2	[11.7 - 17.0]	2.86
**'Enterococcus faecalis C116 (ST228).fna'**	*Enterococcus rotai* LMG 26678	14.2	[11.4 - 17.6]	21.9	[19.6 - 24.3]	14.5	[12.0 - 17.3]	1.46
**'Enterococcus faecalis C146 (ST228).fna'**	*Enterococcus cecorum* DSM 20682	13.2	[10.4 - 16.5]	21.9	[19.7 - 24.4]	13.5	[11.1 - 16.3]	1.22
**'Enterococcus faecalis DSM 111623 (ST624).fna'**	*Enterococcus rotai* DSM 102982	14.2	[11.4 - 17.6]	21.9	[19.7 - 24.4]	14.5	[12.1 - 17.3]	1.39
**'Enterococcus faecalis C144 (ST228).fna'**	*Vagococcus bubulae* SS1994	13.2	[10.5 - 16.5]	21.9	[19.7 - 24.4]	13.5	[11.2 - 16.3]	4.03
**'Enterococcus faecalis CVM N54548 (ST228).fna'**	*Enterococcus rotai* LMG 26678	14.2	[11.4 - 17.6]	21.9	[19.6 - 24.3]	14.5	[12.0 - 17.3]	1.43
**'Enterococcus faecalis R48 (ST228).fna'**	*Enterococcus lemanii* DSM 105069	13.3	[10.6 - 16.6]	21.9	[19.7 - 24.4]	13.7	[11.3 - 16.4]	0.34
**'Enterococcus faecalis CVM N52467 (ST228).fna'**	*Enterococcus cecorum* DSM 20682	13.2	[10.4 - 16.5]	21.9	[19.6 - 24.3]	13.5	[11.1 - 16.3]	1.25
**'Enterococcus faecalis C138 (ST228).fna'**	*Enterococcus cecorum* DSM 20682	13.2	[10.4 - 16.5]	21.9	[19.6 - 24.3]	13.5	[11.1 - 16.3]	1.22
**'Enterococcus faecalis R48 (ST228).fna'**	*Enterococcus rotai* LMG 26678	14.2	[11.4 - 17.6]	21.9	[19.6 - 24.3]	14.4	[12.0 - 17.3]	1.43
**'Enterococcus faecalis C146 (ST228).fna'**	*Enterococcus rotai* LMG 26678	14.2	[11.4 - 17.6]	21.9	[19.7 - 24.4]	14.5	[12.0 - 17.3]	1.46
**'Enterococcus faecalis C138 (ST228).fna'**	*Vagococcus bubulae* SS1994	13.2	[10.4 - 16.5]	21.9	[19.6 - 24.3]	13.5	[11.2 - 16.3]	4.05
**'Enterococcus faecalis DSM 111623 (ST624).fna'**	*Enterococcus massiliensis* AM1	13.2	[10.4 - 16.5]	21.9	[19.7 - 24.4]	13.5	[11.2 - 16.3]	2.18
**'Enterococcus faecalis EN24 (ST228).fna'**	*Enterococcus rotai* LMG 26678	14.2	[11.4 - 17.6]	21.9	[19.7 - 24.4]	14.5	[12.0 - 17.3]	1.41
**'Enterococcus faecalis 209EA1 (ST624).fna'**	*Enterococcus termitis* LMG 8895	14.1	[11.2 - 17.4]	21.9	[19.7 - 24.4]	14.3	[11.9 - 17.2]	0.88
**'Enterococcus faecalis C116 (ST228).fna'**	*Enterococcus xiangfangensis* NCIMB 14834	13.1	[10.4 - 16.4]	21.9	[19.7 - 24.4]	13.5	[11.1 - 16.2]	1.43

Query	Subject	d_0	C.I. d_0	d_4	C.I. d_4	d_6	C.I. d_6	Diff. G+C Percent
**'Enterococcus faecalis C138 (ST228).fna'**	*Enterococcus rotai* LMG 26678	14.2	[11.4 - 17.6]	21.9	[19.7 - 24.4]	14.5	[12.0 - 17.3]	1.47
**'Enterococcus faecalis C144 (ST228).fna'**	*Enterococcus rotai* LMG 26678	14.2	[11.4 - 17.6]	21.9	[19.7 - 24.4]	14.5	[12.0 - 17.3]	1.45
**'Enterococcus faecalis CVM N55265 (ST228).fna'**	*Enterococcus cecorum* DSM 20682	13.2	[10.4 - 16.5]	21.9	[19.6 - 24.3]	13.5	[11.1 - 16.3]	1.21
**'Enterococcus faecalis C144 (ST228).fna'**	*Enterococcus canintestini* DSM 21207	13.3	[10.6 - 16.6]	21.9	[19.6 - 24.3]	13.7	[11.3 - 16.4]	1.33
**'Enterococcus faecalis CVM N53420 (ST228).fna'**	*Enterococcus rotai* LMG 26678	14.2	[11.3 - 17.5]	21.9	[19.6 - 24.3]	14.4	[12.0 - 17.3]	1.31
**'Enterococcus faecalis C144 (ST228).fna'**	*Enterococcus cecorum* DSM 20682	13.2	[10.4 - 16.5]	21.9	[19.6 - 24.3]	13.5	[11.1 - 16.3]	1.21
**'Enterococcus faecalis CVM N52662 (ST228).fna'**	*Enterococcus canintestini* DSM 21207	13.3	[10.6 - 16.6]	21.8	[19.5 - 24.2]	13.6	[11.3 - 16.4]	1.28
**'Enterococcus faecalis CVM N52467 (ST228).fna'**	*Enterococcus rotai* DSM 102982	14.3	[11.4 - 17.6]	21.8	[19.6 - 24.3]	14.5	[12.1 - 17.3]	1.54
**'Enterococcus faecalis C146 (ST228).fna'**	*Enterococcus canintestini* DSM 21207	13.3	[10.6 - 16.6]	21.8	[19.6 - 24.3]	13.7	[11.3 - 16.4]	1.34
**'Enterococcus faecalis CVM N54548 (ST228).fna'**	*Enterococcus wangshanyuanii* MN05	13.9	[11.1 - 17.3]	21.8	[19.5 - 24.2]	14.2	[11.8 - 17.0]	0.29
**'Enterococcus faecalis EN24 (ST228).fna'**	*Enterococcus wangshanyuanii* MN05	13.9	[11.1 - 17.3]	21.8	[19.6 - 24.3]	14.2	[11.8 - 17.0]	0.26
**'Enterococcus faecalis CVM N52587 (STx).fna'**	*Enterococcus cecorum* DSM 20682	13.2	[10.4 - 16.5]	21.8	[19.6 - 24.3]	13.5	[11.1 - 16.3]	1.12
**'Enterococcus faecalis CVM N52662 (ST228).fna'**	*Enterococcus wangshanyuanii* MN05	13.9	[11.1 - 17.3]	21.8	[19.5 - 24.2]	14.2	[11.8 - 17.0]	0.25
**'Enterococcus faecalis CVM N52467 (ST228).fna'**	*Enterococcus hermanniensis* DSM 17122	13.4	[10.7 - 16.7]	21.8	[19.5 - 24.2]	13.8	[11.4 - 16.5]	0.27
**'Enterococcus faecalis C144 (ST228).fna'**	*Enterococcus rotai* DSM 102982	14.3	[11.4 - 17.6]	21.8	[19.6 - 24.2]	14.5	[12.1 - 17.3]	1.5
**'Enterococcus faecalis C116 (ST228).fna'**	*Enterococcus rotai* DSM 102982	14.3	[11.4 - 17.7]	21.8	[19.5 - 24.2]	14.5	[12.1 - 17.4]	1.5
**'Enterococcus faecalis 209EA1 (ST624).fna'**	*Enterococcus plantarum* LMG 26214	14.3	[11.5 - 17.7]	21.8	[19.6 - 24.3]	14.6	[12.1 - 17.4]	1.7
**'Enterococcus faecalis CVM N52587 (STx).fna'**	*Enterococcus rotai* DSM 102982	14.2	[11.4 - 17.6]	21.8	[19.6 - 24.3]	14.5	[12.0 - 17.3]	1.41
**'Enterococcus faecalis CVM N52662 (ST228).fna'**	*Enterococcus rotai* DSM 102982	14.2	[11.4 - 17.6]	21.8	[19.6 - 24.3]	14.5	[12.0 - 17.3]	1.45
**'Enterococcus faecalis CVM N52587 (STx).fna'**	*Enterococcus wangshanyuanii* MN05	13.9	[11.1 - 17.2]	21.8	[19.5 - 24.2]	14.2	[11.8 - 17.0]	0.22
**'Enterococcus faecalis CVM N54548 (ST228).fna'**	*Enterococcus rotai* DSM 102982	14.3	[11.4 - 17.6]	21.8	[19.5 - 24.2]	14.5	[12.1 - 17.3]	1.48
**'Enterococcus faecalis CVM N52467 (ST228).fna'**	*Enterococcus wangshanyuanii* MN05	13.9	[11.1 - 17.3]	21.8	[19.5 - 24.2]	14.2	[11.8 - 17.0]	0.35
**'Enterococcus faecalis C138 (ST228).fna'**	*Enterococcus canintestini* DSM 21207	13.3	[10.6 - 16.6]	21.8	[19.5 - 24.2]	13.7	[11.3 - 16.4]	1.35
**'Enterococcus faecalis EN24 (ST228).fna'**	*Enterococcus rotai* DSM 102982	14.3	[11.5 - 17.7]	21.8	[19.6 - 24.3]	14.5	[12.1 - 17.4]	1.46

Query	Subject	d_0	C.I. d_0	d_4	C.I. d_4	d_6	C.I. d_6	Diff. G+C Percent
**'Enterococcus faecalis CVM N55265 (ST228).fna'**	*Enterococcus wangshanyuanii* MN05	13.9	[11.1 - 17.3]	21.8	[19.5 - 24.2]	14.2	[11.8 - 17.0]	0.31
**'Enterococcus faecalis R48 (ST228).fna'**	*Enterococcus rotai* DSM 102982	14.2	[11.4 - 17.6]	21.8	[19.5 - 24.2]	14.5	[12.0 - 17.3]	1.48
**'Enterococcus faecalis R30 (ST228).fna'**	*Enterococcus wangshanyuanii* MN05	13.9	[11.1 - 17.3]	21.8	[19.6 - 24.3]	14.2	[11.8 - 17.0]	0.43
**'Enterococcus faecalis C138 (ST228).fna'**	*Enterococcus rotai* DSM 102982	14.3	[11.4 - 17.6]	21.8	[19.5 - 24.2]	14.5	[12.1 - 17.3]	1.52
**'Enterococcus faecalis CVM N55265 (ST228).fna'**	*Enterococcus rotai* DSM 102982	14.2	[11.4 - 17.6]	21.8	[19.6 - 24.3]	14.5	[12.1 - 17.3]	1.51
**'Enterococcus faecalis CVM N53420 (ST228).fna'**	*Enterococcus cecorum* DSM 20682	13.1	[10.4 - 16.4]	21.8	[19.6 - 24.2]	13.5	[11.1 - 16.3]	1.06
**'Enterococcus faecalis C146 (ST228).fna'**	*Enterococcus rotai* DSM 102982	14.3	[11.4 - 17.6]	21.8	[19.6 - 24.3]	14.5	[12.1 - 17.3]	1.51
**'Enterococcus faecalis CVM N52467 (ST228).fna'**	*Enterococcus canintestini* DSM 21207	13.3	[10.6 - 16.6]	21.8	[19.5 - 24.2]	13.7	[11.3 - 16.4]	1.37
**'Enterococcus faecalis R30 (ST228).fna'**	*Enterococcus canintestini* DSM 21207	13.3	[10.6 - 16.7]	21.8	[19.5 - 24.2]	13.7	[11.3 - 16.5]	1.45
**'Enterococcus faecalis C138 (ST228).fna'**	*Enterococcus wangshanyuanii* MN05	13.9	[11.1 - 17.3]	21.8	[19.5 - 24.2]	14.2	[11.8 - 17.0]	0.32
**'Enterococcus faecalis EN24 (ST228).fna'**	*Enterococcus canintestini* DSM 21207	13.3	[10.6 - 16.6]	21.8	[19.5 - 24.2]	13.7	[11.3 - 16.4]	1.29
**'Enterococcus faecalis CVM N52662 (ST228).fna'**	*Enterococcus cecorum* DSM 20682	13.1	[10.4 - 16.5]	21.8	[19.5 - 24.2]	13.5	[11.1 - 16.3]	1.15
**'Enterococcus faecalis R30 (ST228).fna'**	*Enterococcus rotai* DSM 102982	14.3	[11.5 - 17.7]	21.8	[19.6 - 24.3]	14.5	[12.1 - 17.4]	1.62
**'Enterococcus faecalis C144 (ST228).fna'**	*Enterococcus wangshanyuanii* MN05	13.9	[11.1 - 17.3]	21.8	[19.5 - 24.2]	14.2	[11.8 - 17.0]	0.31
**'Enterococcus faecalis C146 (ST228).fna'**	*Enterococcus wangshanyuanii* MN05	13.9	[11.1 - 17.3]	21.8	[19.6 - 24.3]	14.2	[11.8 - 17.0]	0.32
**'Enterococcus faecalis G81 (ST1468).fna'**	*Enterococcus moraviensis* ATCC BAA-383	14.0	[11.2 - 17.4]	21.8	[19.5 - 24.2]	14.3	[11.8 - 17.1]	1.57
**'Enterococcus faecalis C116 (ST228).fna'**	*Enterococcus wangshanyuanii* MN05	13.9	[11.1 - 17.3]	21.8	[19.5 - 24.2]	14.2	[11.8 - 17.0]	0.31
**'Enterococcus faecalis CVM N53420 (ST228).fna'**	*Enterococcus wangshanyuanii* MN05	13.9	[11.1 - 17.2]	21.8	[19.6 - 24.3]	14.2	[11.7 - 17.0]	0.17
**'Enterococcus faecalis R48 (ST228).fna'**	*Enterococcus wangshanyuanii* MN05	13.9	[11.1 - 17.2]	21.7	[19.4 - 24.1]	14.2	[11.8 - 17.0]	0.29
**'Enterococcus faecalis G81 (ST1468).fna'**	*Enterococcus wangshanyuanii* MN05	13.9	[11.1 - 17.3]	21.7	[19.5 - 24.2]	14.2	[11.8 - 17.0]	0.28
**'Enterococcus faecalis DSM 111623 (ST624).fna'**	*Enterococcus moraviensis* ATCC BAA-383	14.0	[11.2 - 17.4]	21.7	[19.4 - 24.1]	14.3	[11.8 - 17.1]	1.49
**'Enterococcus faecalis CVM N53420 (ST228).fna'**	*Enterococcus rotai* DSM 102982	14.2	[11.4 - 17.6]	21.7	[19.5 - 24.2]	14.5	[12.0 - 17.3]	1.36
**'Enterococcus faecalis CVM N52587 (STx).fna'**	*Enterococcus canintestini* DSM 21207	13.3	[10.6 - 16.6]	21.7	[19.5 - 24.2]	13.6	[11.3 - 16.4]	1.24
**'Enterococcus faecalis CVM N55265 (ST228).fna'**	*Enterococcus moraviensis* ATCC BAA-383	14.1	[11.3 - 17.4]	21.7	[19.4 - 24.1]	14.3	[11.9 - 17.2]	1.61

Query	Subject	d_0	C.I. d_0	d_4	C.I. d_4	d_6	C.I. d_6	Diff. G+C Percent
**'Enterococcus faecalis R48 (ST228).fna'**	*Enterococcus cecorum* DSM 20682	13.1	[10.4 - 16.4]	21.7	[19.5 - 24.2]	13.5	[11.1 - 16.3]	1.19
**'Enterococcus faecalis 209EA1 (ST624).fna'**	*Enterococcus quebecensis* LMG 26306	14.5	[11.6 - 17.9]	21.7	[19.5 - 24.2]	14.7	[12.3 - 17.6]	2.63
**'Enterococcus faecalis CVM N52587 (STx).fna'**	*Enterococcus moraviensis* ATCC BAA-383	14.0	[11.2 - 17.4]	21.7	[19.5 - 24.2]	14.3	[11.9 - 17.1]	1.52
**'Enterococcus faecalis 209EA1 (ST624).fna'**	*Enterococcus wangshanyuanii* CGMCC 1.15942	14.0	[11.2 - 17.4]	21.7	[19.4 - 24.1]	14.3	[11.9 - 17.1]	0.48
**'Enterococcus faecalis CVM N53420 (ST228).fna'**	*Enterococcus canintestini* DSM 21207	13.3	[10.6 - 16.6]	21.7	[19.5 - 24.2]	13.6	[11.3 - 16.4]	1.19
**'Enterococcus faecalis CVM N54548 (ST228).fna'**	*Enterococcus canintestini* DSM 21207	13.3	[10.6 - 16.6]	21.7	[19.5 - 24.2]	13.7	[11.3 - 16.4]	1.31
**'Enterococcus faecalis CVM N55265 (ST228).fna'**	*Enterococcus canintestini* DSM 21207	13.3	[10.6 - 16.6]	21.7	[19.5 - 24.2]	13.7	[11.3 - 16.4]	1.34
**'Enterococcus faecalis CVM N52662 (ST228).fna'**	*Enterococcus moraviensis* ATCC BAA-383	14.0	[11.2 - 17.4]	21.7	[19.5 - 24.2]	14.3	[11.9 - 17.1]	1.55
**'Enterococcus faecalis CVM N52467 (ST228).fna'**	*Enterococcus moraviensis* ATCC BAA-383	14.1	[11.3 - 17.4]	21.7	[19.5 - 24.2]	14.3	[11.9 - 17.2]	1.65
**'Enterococcus faecalis DSM 111623 (ST624).fna'**	*Enterococcus wangshanyuanii* MN05	14.0	[11.2 - 17.3]	21.7	[19.5 - 24.2]	14.2	[11.8 - 17.1]	0.2
**'Enterococcus faecalis G81 (ST1468).fna'**	*Enterococcus silesiacus* LMG 23085	13.9	[11.1 - 17.2]	21.6	[19.3 - 24.0]	14.1	[11.7 - 17.0]	1.14
**'Enterococcus faecalis C138 (ST228).fna'**	*Enterococcus haemoperoxidus* ATCC BAA-382	14.4	[11.6 - 17.8]	21.6	[19.3 - 24.0]	14.6	[12.2 - 17.5]	1.86
**'Enterococcus faecalis R30 (ST228).fna'**	*Enterococcus moraviensis* ATCC BAA-383	14.1	[11.3 - 17.5]	21.6	[19.4 - 24.1]	14.4	[11.9 - 17.2]	1.73
**'Enterococcus faecalis CVM N54548 (ST228).fna'**	*Enterococcus moraviensis* ATCC BAA-383	14.1	[11.3 - 17.5]	21.6	[19.3 - 24.0]	14.3	[11.9 - 17.2]	1.59
**'Enterococcus faecalis C144 (ST228).fna'**	*Enterococcus hermanniensis* DSM 17122	13.4	[10.7 - 16.8]	21.6	[19.4 - 24.1]	13.8	[11.4 - 16.6]	0.23
**'Enterococcus faecalis C138 (ST228).fna'**	*Enterococcus hermanniensis* DSM 17122	13.4	[10.7 - 16.8]	21.6	[19.3 - 24.0]	13.8	[11.4 - 16.6]	0.24
**'Enterococcus faecalis C146 (ST228).fna'**	*Enterococcus hermanniensis* DSM 17122	13.4	[10.7 - 16.8]	21.6	[19.4 - 24.0]	13.8	[11.4 - 16.6]	0.24
**'Enterococcus faecalis C116 (ST228).fna'**	*Enterococcus canintestini* DSM 21207	13.3	[10.6 - 16.7]	21.6	[19.3 - 24.0]	13.7	[11.3 - 16.5]	1.33
**'Enterococcus faecalis EN24 (ST228).fna'**	*Enterococcus moraviensis* ATCC BAA-383	14.1	[11.3 - 17.4]	21.6	[19.4 - 24.1]	14.3	[11.9 - 17.2]	1.56
**'Enterococcus faecalis CVM N52662 (ST228).fna'**	*Enterococcus termitis* LMG 8895	13.9	[11.1 - 17.3]	21.6	[19.3 - 24.0]	14.2	[11.8 - 17.0]	0.68
**'Enterococcus faecalis C138 (ST228).fna'**	*Enterococcus moraviensis* ATCC BAA-383	14.1	[11.3 - 17.5]	21.6	[19.4 - 24.0]	14.4	[11.9 - 17.2]	1.62
**'Enterococcus faecalis C144 (ST228).fna'**	*Enterococcus moraviensis* ATCC BAA-383	14.1	[11.3 - 17.5]	21.6	[19.4 - 24.0]	14.3	[11.9 - 17.2]	1.61

Query	Subject	d_0	C.I. d_0	d_4	C.I. d_4	d_6	C.I. d_6	Diff. G+C Percent
**'Enterococcus faecalis C146 (ST228).fna'**	*Enterococcus silesiacus* LMG 23085	13.8	[11.0 - 17.2]	21.6	[19.3 - 24.0]	14.1	[11.7 - 16.9]	1.18
**'Enterococcus faecalis CVM N54548 (ST228).fna'**	*Enterococcus cecorum* DSM 20682	13.1	[10.4 - 16.4]	21.6	[19.3 - 24.0]	13.5	[11.1 - 16.3]	1.19
**'Enterococcus faecalis CVM N54548 (ST228).fna'**	*Enterococcus haemoperoxidus* ATCC BAA-382	14.4	[11.6 - 17.8]	21.6	[19.3 - 24.0]	14.6	[12.2 - 17.5]	1.83
**'Enterococcus faecalis CVM N52467 (ST228).fna'**	*Enterococcus haemoperoxidus* ATCC BAA-382	14.4	[11.5 - 17.8]	21.6	[19.4 - 24.1]	14.6	[12.2 - 17.5]	1.89
**'Enterococcus faecalis R30 (ST228).fna'**	*Enterococcus haemoperoxidus* ATCC BAA-382	14.4	[11.6 - 17.8]	21.6	[19.4 - 24.0]	14.6	[12.2 - 17.5]	1.97
**'Enterococcus faecalis CVM N53420 (ST228).fna'**	*Enterococcus moraviensis* ATCC BAA-383	14.0	[11.2 - 17.4]	21.6	[19.4 - 24.1]	14.3	[11.9 - 17.1]	1.46
**'Enterococcus faecalis CVM N53420 (ST228).fna'**	*Enterococcus haemoperoxidus* ATCC BAA-382	14.3	[11.5 - 17.7]	21.6	[19.3 - 24.0]	14.6	[12.1 - 17.4]	1.7
**'Enterococcus faecalis EN24 (ST228).fna'**	*Enterococcus haemoperoxidus* ATCC BAA-382	14.4	[11.5 - 17.8]	21.6	[19.4 - 24.1]	14.6	[12.2 - 17.4]	1.8
**'Enterococcus faecalis C146 (ST228).fna'**	*Enterococcus haemoperoxidus* ATCC BAA-382	14.4	[11.5 - 17.8]	21.6	[19.4 - 24.1]	14.6	[12.2 - 17.5]	1.86
**'Enterococcus faecalis C146 (ST228).fna'**	*Enterococcus moraviensis* ATCC BAA-383	14.1	[11.3 - 17.5]	21.6	[19.4 - 24.1]	14.4	[11.9 - 17.2]	1.62
**'Enterococcus faecalis CVM N55265 (ST228).fna'**	*Enterococcus ureasiticus* DSM 23328	14.2	[11.4 - 17.6]	21.6	[19.3 - 24.0]	14.5	[12.0 - 17.3]	1.78
**'Enterococcus faecalis C144 (ST228).fna'**	*Enterococcus haemoperoxidus* ATCC BAA-382	14.4	[11.6 - 17.8]	21.6	[19.4 - 24.0]	14.6	[12.2 - 17.5]	1.85
**'Enterococcus faecalis CVM N52467 (ST228).fna'**	*Enterococcus ureasiticus* DSM 23328	14.2	[11.4 - 17.6]	21.6	[19.3 - 24.0]	14.5	[12.0 - 17.3]	1.81
**'Enterococcus faecalis C146 (ST228).fna'**	*Enterococcus ureasiticus* DSM 23328	14.2	[11.4 - 17.6]	21.6	[19.3 - 24.0]	14.5	[12.0 - 17.3]	1.78
**'Enterococcus faecalis CVM N52587 (STx).fna'**	*Enterococcus haemoperoxidus* ATCC BAA-382	14.4	[11.5 - 17.8]	21.6	[19.4 - 24.1]	14.6	[12.1 - 17.4]	1.76
**'Enterococcus faecalis C116 (ST228).fna'**	*Enterococcus moraviensis* ATCC BAA-383	14.1	[11.3 - 17.4]	21.6	[19.3 - 24.0]	14.3	[11.9 - 17.2]	1.61
**'Enterococcus faecalis CVM N55265 (ST228).fna'**	*Enterococcus haemoperoxidus* ATCC BAA-382	14.4	[11.5 - 17.8]	21.6	[19.4 - 24.1]	14.6	[12.2 - 17.5]	1.85
**'Enterococcus faecalis CVM N52662 (ST228).fna'**	*Enterococcus ureasiticus* DSM 23328	14.2	[11.4 - 17.6]	21.6	[19.3 - 24.0]	14.5	[12.0 - 17.3]	1.72
**'Enterococcus faecalis CVM N52662 (ST228).fna'**	*Enterococcus haemoperoxidus* ATCC BAA-382	14.3	[11.5 - 17.7]	21.6	[19.4 - 24.1]	14.6	[12.1 - 17.4]	1.79
**'Enterococcus faecalis R30 (ST228).fna'**	*Enterococcus silesiacus* LMG 23085	13.9	[11.1 - 17.2]	21.5	[19.3 - 24.0]	14.2	[11.7 - 17.0]	1.29
**'Enterococcus faecalis CVM N52467 (ST228).fna'**	*Enterococcus termitis* LMG 8895	13.9	[11.1 - 17.3]	21.5	[19.2 - 23.9]	14.2	[11.8 - 17.0]	0.77

Query	Subject	d_0	C.I. d_0	d_4	C.I. d_4	d_6	C.I. d_6	Diff. G+C Percent
**'Enterococcus faecalis DSM 111623 (ST624).fna'**	*Enterococcus ureasiticus* DSM 23328	14.2	[11.4 - 17.6]	21.5	[19.2 - 23.9]	14.5	[12.0 - 17.3]	1.66
**'Enterococcus faecalis CVM N52662 (ST228).fna'**	*Enterococcus silesiacus* LMG 23085	13.8	[11.0 - 17.2]	21.5	[19.3 - 24.0]	14.1	[11.7 - 16.9]	1.12
**'Enterococcus faecalis C116 (ST228).fna'**	*Enterococcus silesiacus* LMG 23085	13.8	[11.1 - 17.2]	21.5	[19.2 - 23.9]	14.1	[11.7 - 16.9]	1.17
**'Enterococcus faecalis R30 (ST228).fna'**	*Enterococcus ureasiticus* DSM 23328	14.3	[11.5 - 17.7]	21.5	[19.3 - 24.0]	14.5	[12.1 - 17.4]	1.9
**'Enterococcus faecalis C144 (ST228).fna'**	*Enterococcus silesiacus* LMG 23085	13.8	[11.0 - 17.2]	21.5	[19.3 - 24.0]	14.1	[11.7 - 16.9]	1.17
**'Enterococcus faecalis R30 (ST228).fna'**	*Enterococcus cecorum* DSM 20682	13.2	[10.4 - 16.5]	21.5	[19.3 - 24.0]	13.5	[11.1 - 16.3]	1.33
**'Enterococcus faecalis CVM N54548 (ST228).fna'**	*Enterococcus termitis* LMG 8895	14.0	[11.2 - 17.3]	21.5	[19.2 - 23.9]	14.2	[11.8 - 17.0]	0.71
**'Enterococcus faecalis EN24 (ST228).fna'**	*Enterococcus silesiacus* LMG 23085	13.8	[11.1 - 17.2]	21.5	[19.3 - 24.0]	14.1	[11.7 - 16.9]	1.13
**'Enterococcus faecalis C146 (ST228).fna'**	*Enterococcus termitis* LMG 8895	14.0	[11.2 - 17.3]	21.5	[19.2 - 23.9]	14.2	[11.8 - 17.0]	0.74
**'Enterococcus faecalis R48 (ST228).fna'**	*Enterococcus canintestini* DSM 21207	13.3	[10.6 - 16.6]	21.5	[19.3 - 24.0]	13.7	[11.3 - 16.4]	1.31
**'Enterococcus faecalis CVM N52467 (ST228).fna'**	*Enterococcus silesiacus* LMG 23085	13.8	[11.0 - 17.2]	21.5	[19.3 - 24.0]	14.1	[11.7 - 16.9]	1.21
**'Enterococcus faecalis R30 (ST228).fna'**	*Enterococcus termitis* LMG 8895	14.0	[11.2 - 17.3]	21.5	[19.2 - 23.9]	14.2	[11.8 - 17.1]	0.85
**'Enterococcus faecalis C116 (ST228).fna'**	*Enterococcus cecorum* DSM 20682	13.1	[10.4 - 16.4]	21.5	[19.2 - 23.9]	13.5	[11.1 - 16.3]	1.21
**'Enterococcus faecalis R48 (ST228).fna'**	*Enterococcus ureasiticus* DSM 23328	14.2	[11.4 - 17.6]	21.5	[19.2 - 23.9]	14.5	[12.0 - 17.3]	1.75
**'Enterococcus faecalis CVM N55265 (ST228).fna'**	*Enterococcus termitis* LMG 8895	13.9	[11.2 - 17.3]	21.5	[19.2 - 23.9]	14.2	[11.8 - 17.0]	0.74
**'Enterococcus faecalis C138 (ST228).fna'**	*Enterococcus ureasiticus* DSM 23328	14.3	[11.4 - 17.6]	21.5	[19.3 - 24.0]	14.5	[12.1 - 17.3]	1.79
**'Enterococcus faecalis CVM N53420 (ST228).fna'**	*Enterococcus ureasiticus* DSM 23328	14.2	[11.4 - 17.6]	21.5	[19.3 - 23.9]	14.5	[12.0 - 17.3]	1.63
**'Enterococcus faecalis R48 (ST228).fna'**	*Enterococcus haemoperoxidus* ATCC BAA-382	14.3	[11.5 - 17.7]	21.5	[19.2 - 23.9]	14.6	[12.1 - 17.4]	1.83
**'Enterococcus faecalis CVM N53420 (ST228).fna'**	*Enterococcus silesiacus* LMG 23085	13.8	[11.0 - 17.2]	21.5	[19.3 - 24.0]	14.1	[11.7 - 16.9]	1.03
**'Enterococcus faecalis C116 (ST228).fna'**	*Enterococcus ureasiticus* DSM 23328	14.3	[11.4 - 17.6]	21.5	[19.3 - 23.9]	14.5	[12.1 - 17.3]	1.78
**'Enterococcus faecalis CVM N52587 (STx).fna'**	*Enterococcus termitis* LMG 8895	13.9	[11.1 - 17.3]	21.5	[19.2 - 23.9]	14.2	[11.8 - 17.0]	0.64
**'Enterococcus faecalis CVM N55265 (ST228).fna'**	*Enterococcus silesiacus* LMG 23085	13.8	[11.0 - 17.2]	21.5	[19.3 - 24.0]	14.1	[11.7 - 16.9]	1.18
**'Enterococcus faecalis G81 (ST1468).fna'**	*Enterococcus ureasiticus* DSM 23328	14.2	[11.4 - 17.6]	21.5	[19.3 - 24.0]	14.4	[12.0 - 17.3]	1.74
**'Enterococcus faecalis EN24 (ST228).fna'**	*Enterococcus ureasiticus* DSM 23328	14.3	[11.4 - 17.6]	21.5	[19.3 - 24.0]	14.5	[12.1 - 17.3]	1.73

Query	Subject	d_0	C.I. d_0	d_4	C.I. d_4	d_6	C.I. d_6	Diff. G+C Percent
**'Enterococcus faecalis DSM 111623 (ST624).fna'**	*Enterococcus silesiacus* LMG 23085	13.9	[11.1 - 17.2]	21.5	[19.2 - 23.9]	14.2	[11.7 - 17.0]	1.06
**'Enterococcus faecalis G81 (ST1468).fna'**	*Enterococcus haemoperoxidus* ATCC BAA-382	14.4	[11.5 - 17.8]	21.5	[19.2 - 23.9]	14.6	[12.2 - 17.5]	1.81
**'Enterococcus faecalis R48 (ST228).fna'**	*Enterococcus moraviensis* ATCC BAA-383	14.0	[11.2 - 17.4]	21.5	[19.3 - 23.9]	14.3	[11.9 - 17.1]	1.59
**'Enterococcus faecalis R48 (ST228).fna'**	*Enterococcus silesiacus* LMG 23085	13.8	[11.0 - 17.2]	21.5	[19.2 - 23.9]	14.1	[11.7 - 16.9]	1.15
**'Enterococcus faecalis EN24 (ST228).fna'**	*Enterococcus termitis* LMG 8895	13.9	[11.1 - 17.3]	21.5	[19.2 - 23.9]	14.2	[11.8 - 17.0]	0.69
**'Enterococcus faecalis C116 (ST228).fna'**	*Enterococcus haemoperoxidus* ATCC BAA-382	14.4	[11.5 - 17.8]	21.5	[19.3 - 24.0]	14.6	[12.2 - 17.4]	1.85
**'Enterococcus faecalis CVM N54548 (ST228).fna'**	*Enterococcus ureasiticus* DSM 23328	14.2	[11.4 - 17.6]	21.5	[19.3 - 23.9]	14.5	[12.0 - 17.3]	1.76
**'Enterococcus faecalis CVM N52587 (STx).fna'**	*Enterococcus ureasiticus* DSM 23328	14.2	[11.4 - 17.6]	21.5	[19.3 - 24.0]	14.5	[12.0 - 17.3]	1.69
**'Enterococcus faecalis C138 (ST228).fna'**	*Enterococcus silesiacus* LMG 23085	13.8	[11.0 - 17.2]	21.5	[19.3 - 24.0]	14.1	[11.7 - 16.9]	1.19
**'Enterococcus faecalis C116 (ST228).fna'**	*Enterococcus termitis* LMG 8895	14.0	[11.2 - 17.3]	21.5	[19.2 - 23.9]	14.2	[11.8 - 17.1]	0.73
**'Enterococcus faecalis CVM N53420 (ST228).fna'**	*Enterococcus termitis* LMG 8895	13.9	[11.1 - 17.3]	21.5	[19.2 - 23.9]	14.2	[11.8 - 17.0]	0.59
**'Enterococcus faecalis CVM N52587 (STx).fna'**	*Enterococcus silesiacus* LMG 23085	13.8	[11.0 - 17.2]	21.5	[19.3 - 24.0]	14.1	[11.7 - 16.9]	1.08
**'Enterococcus faecalis C144 (ST228).fna'**	*Enterococcus ureasiticus* DSM 23328	14.2	[11.4 - 17.6]	21.5	[19.3 - 24.0]	14.5	[12.0 - 17.3]	1.78
**'Enterococcus faecalis R30 (ST228).fna'**	*Enterococcus quebecensis* LMG 26306	14.4	[11.5 - 17.8]	21.4	[19.1 - 23.8]	14.6	[12.2 - 17.5]	2.6
**'Enterococcus faecalis C144 (ST228).fna'**	*Enterococcus termitis* LMG 8895	14.0	[11.2 - 17.3]	21.4	[19.2 - 23.9]	14.2	[11.8 - 17.0]	0.73
**'Enterococcus faecalis C138 (ST228).fna'**	*Enterococcus termitis* LMG 8895	14.0	[11.2 - 17.3]	21.4	[19.2 - 23.9]	14.2	[11.8 - 17.0]	0.75
**'Enterococcus faecalis CVM N52467 (ST228).fna'**	*Enterococcus quebecensis* LMG 26306	14.3	[11.5 - 17.7]	21.4	[19.2 - 23.9]	14.6	[12.1 - 17.4]	2.52
**'Enterococcus faecalis C146 (ST228).fna'**	*Enterococcus quebecensis* LMG 26306	14.3	[11.5 - 17.7]	21.4	[19.2 - 23.8]	14.6	[12.1 - 17.4]	2.49
**'Enterococcus faecalis CVM N52587 (STx).fna'**	*Enterococcus quebecensis* LMG 26306	14.3	[11.5 - 17.7]	21.4	[19.2 - 23.8]	14.5	[12.1 - 17.4]	2.39
**'Enterococcus faecalis 209EA1 (ST624).fna'**	*Enterococcus ureilyticus* LMG 26676	14.4	[11.5 - 17.8]	21.4	[19.2 - 23.9]	14.6	[12.2 - 17.5]	1.6
**'Enterococcus faecalis CVM N55265 (ST228).fna'**	*Enterococcus quebecensis* LMG 26306	14.3	[11.5 - 17.7]	21.4	[19.2 - 23.8]	14.6	[12.1 - 17.4]	2.48
**'Enterococcus faecalis CVM N54548 (ST228).fna'**	*Enterococcus quebecensis* LMG 26306	14.3	[11.5 - 17.7]	21.4	[19.1 - 23.8]	14.6	[12.1 - 17.4]	2.46
**'Enterococcus faecalis C138 (ST228).fna'**	*Enterococcus quebecensis* LMG 26306	14.3	[11.5 - 17.7]	21.4	[19.1 - 23.8]	14.6	[12.1 - 17.4]	2.49
**'Enterococcus faecalis EN24 (ST228).fna'**	*Enterococcus quebecensis* LMG 26306	14.3	[11.5 - 17.7]	21.4	[19.1 - 23.8]	14.6	[12.1 - 17.4]	2.43

Query	Subject	d_0	C.I. d_0	d_4	C.I. d_4	d_6	C.I. d_6	Diff. G+C Percent
**'Enterococcus faecalis C144 (ST228).fna'**	*Enterococcus quebecensis* LMG 26306	14.3	[11.5 - 17.7]	21.4	[19.1 - 23.8]	14.6	[12.1 - 17.4]	2.48
**'Enterococcus faecalis CVM N52662 (ST228).fna'**	*Enterococcus quebecensis* LMG 26306	14.3	[11.5 - 17.7]	21.4	[19.2 - 23.9]	14.5	[12.1 - 17.4]	2.42
**'Enterococcus faecalis CVM N53420 (ST228).fna'**	*Enterococcus quebecensis* LMG 26306	14.3	[11.5 - 17.7]	21.4	[19.1 - 23.8]	14.5	[12.1 - 17.4]	2.34
**'Enterococcus faecalis CVM N54548 (ST228).fna'**	*Enterococcus silesiacus* LMG 23085	13.8	[11.1 - 17.2]	21.4	[19.2 - 23.9]	14.1	[11.7 - 16.9]	1.15
**'Enterococcus faecalis R48 (ST228).fna'**	*Enterococcus termitis* LMG 8895	13.9	[11.1 - 17.3]	21.4	[19.1 - 23.8]	14.2	[11.8 - 17.0]	0.71
**'Enterococcus faecalis DSM 111623 (ST624).fna'**	*Enterococcus wangshanyuanii* CGMCC 1.15942	14.0	[11.2 - 17.3]	21.3	[19.1 - 23.8]	14.2	[11.8 - 17.0]	0.21
**'Enterococcus faecalis R48 (ST228).fna'**	*Enterococcus quebecensis* LMG 26306	14.3	[11.5 - 17.7]	21.3	[19.1 - 23.8]	14.5	[12.1 - 17.4]	2.46
**'Enterococcus faecalis EN24 (ST228).fna'**	*Vagococcus bubulae* SS1994	13.1	[10.4 - 16.4]	21.3	[19.0 - 23.7]	13.5	[11.1 - 16.3]	3.99
**'Enterococcus faecalis DSM 111623 (ST624).fna'**	*Enterococcus lemanii* DSM 105069	13.2	[10.5 - 16.5]	21.3	[19.0 - 23.7]	13.5	[11.2 - 16.3]	0.25
**'Enterococcus faecalis C116 (ST228).fna'**	*Enterococcus quebecensis* LMG 26306	14.3	[11.5 - 17.7]	21.3	[19.1 - 23.8]	14.6	[12.1 - 17.4]	2.48
**'Enterococcus faecalis DSM 111623 (ST624).fna'**	*Vagococcus bubulae* SS1994	13.1	[10.4 - 16.4]	21.3	[19.0 - 23.7]	13.4	[11.1 - 16.2]	3.92
**'Enterococcus faecalis G81 (ST1468).fna'**	*Enterococcus termitis* LMG 8895	14.0	[11.2 - 17.4]	21.3	[19.1 - 23.8]	14.3	[11.8 - 17.1]	0.7
**'Enterococcus faecalis DSM 111623 (ST624).fna'**	*Enterococcus haemoperoxidus* ATCC BAA-382	14.4	[11.6 - 17.8]	21.3	[19.1 - 23.7]	14.6	[12.2 - 17.5]	1.73
**'Enterococcus faecalis G81 (ST1468).fna'**	*Enterococcus quebecensis* LMG 26306	14.4	[11.5 - 17.8]	21.2	[18.9 - 23.6]	14.6	[12.1 - 17.4]	2.44
**'Enterococcus faecalis C146 (ST228).fna'**	*Enterococcus wangshanyuanii* CGMCC 1.15942	13.9	[11.1 - 17.2]	21.2	[18.9 - 23.6]	14.2	[11.7 - 17.0]	0.34
**'Enterococcus faecalis CVM N52662 (ST228).fna'**	*Enterococcus plantarum* LMG 26214	14.2	[11.4 - 17.6]	21.2	[19.0 - 23.7]	14.4	[12.0 - 17.3]	1.5
**'Enterococcus faecalis C138 (ST228).fna'**	*Enterococcus plantarum* LMG 26214	14.2	[11.4 - 17.6]	21.2	[19.0 - 23.6]	14.5	[12.0 - 17.3]	1.57
**'Enterococcus faecalis C146 (ST228).fna'**	*Enterococcus lemanii* DSM 105069	13.3	[10.5 - 16.6]	21.2	[19.0 - 23.7]	13.6	[11.2 - 16.4]	0.37
**'Enterococcus faecalis C138 (ST228).fna'**	*Enterococcus lemanii* DSM 105069	13.3	[10.5 - 16.6]	21.2	[18.9 - 23.6]	13.6	[11.2 - 16.4]	0.38
**'Enterococcus faecalis CVM N52587 (STx).fna'**	*Enterococcus wangshanyuanii* CGMCC 1.15942	13.9	[11.1 - 17.2]	21.2	[18.9 - 23.6]	14.1	[11.7 - 17.0]	0.24
**'Enterococcus faecalis C116 (ST228).fna'**	*Enterococcus wangshanyuanii* CGMCC 1.15942	13.9	[11.1 - 17.3]	21.2	[18.9 - 23.6]	14.2	[11.8 - 17.0]	0.33
**'Enterococcus faecalis CVM N52587 (STx).fna'**	*Enterococcus plantarum* LMG 26214	14.2	[11.4 - 17.6]	21.2	[19.0 - 23.7]	14.4	[12.0 - 17.3]	1.47
**'Enterococcus faecalis C144 (ST228).fna'**	*Enterococcus plantarum* LMG 26214	14.2	[11.4 - 17.6]	21.2	[19.0 - 23.6]	14.5	[12.0 - 17.3]	1.56
**'Enterococcus faecalis CVM N52587 (STx).fna'**	*Enterococcus lemanii* DSM 105069	13.3	[10.5 - 16.6]	21.2	[18.9 - 23.6]	13.6	[11.2 - 16.4]	0.27

Query	Subject	d_0	C.I. d_0	d_4	C.I. d_4	d_6	C.I. d_6	Diff. G+C Percent
**'Enterococcus faecalis G81 (ST1468).fna'**	*Enterococcus plantarum* LMG 26214	14.2	[11.4 - 17.6]	21.2	[19.0 - 23.6]	14.4	[12.0 - 17.3]	1.52
**'Enterococcus faecalis C146 (ST228).fna'**	*Enterococcus plantarum* LMG 26214	14.2	[11.4 - 17.6]	21.2	[19.0 - 23.7]	14.5	[12.0 - 17.3]	1.56
**'Enterococcus faecalis G81 (ST1468).fna'**	*Enterococcus rivorum* DSM 104544	13.8	[11.0 - 17.1]	21.2	[19.0 - 23.6]	14.1	[11.6 - 16.9]	2.67
**'Enterococcus faecalis C116 (ST228).fna'**	*Enterococcus plantarum* LMG 26214	14.2	[11.4 - 17.6]	21.2	[18.9 - 23.6]	14.4	[12.0 - 17.3]	1.56
**'Enterococcus faecalis EN24 (ST228).fna'**	*Enterococcus wangshanyuanii* CGMCC 1.15942	13.9	[11.1 - 17.2]	21.2	[18.9 - 23.6]	14.2	[11.7 - 17.0]	0.28
**'Enterococcus faecalis R48 (ST228).fna'**	*Enterococcus plantarum* LMG 26214	14.2	[11.4 - 17.6]	21.2	[18.9 - 23.6]	14.4	[12.0 - 17.3]	1.53
**'Enterococcus faecalis CVM N55265 (ST228).fna'**	*Enterococcus plantarum* LMG 26214	14.2	[11.4 - 17.6]	21.2	[19.0 - 23.7]	14.4	[12.0 - 17.3]	1.56
**'Enterococcus faecalis CVM N53420 (ST228).fna'**	*Enterococcus plantarum* LMG 26214	14.2	[11.4 - 17.5]	21.2	[18.9 - 23.6]	14.4	[12.0 - 17.3]	1.41
**'Enterococcus faecalis DSM 111623 (ST624).fna'**	*Enterococcus termitis* LMG 8895	14.0	[11.2 - 17.4]	21.2	[19.0 - 23.7]	14.3	[11.8 - 17.1]	0.62
**'Enterococcus faecalis CVM N54548 (ST228).fna'**	*Vagococcus bubulae* SS1994	13.1	[10.4 - 16.5]	21.2	[19.0 - 23.7]	13.5	[11.1 - 16.3]	4.01
**'Enterococcus faecalis CVM N52467 (ST228).fna'**	*Enterococcus wangshanyuanii* CGMCC 1.15942	13.9	[11.1 - 17.2]	21.2	[18.9 - 23.6]	14.2	[11.7 - 17.0]	0.37
**'Enterococcus faecalis C144 (ST228).fna'**	*Enterococcus lemanii* DSM 105069	13.3	[10.5 - 16.6]	21.2	[18.9 - 23.6]	13.6	[11.2 - 16.4]	0.36
**'Enterococcus faecalis CVM N55265 (ST228).fna'**	*Enterococcus lemanii* DSM 105069	13.3	[10.5 - 16.6]	21.2	[19.0 - 23.6]	13.6	[11.2 - 16.4]	0.37
**'Enterococcus faecalis CVM N53420 (ST228).fna'**	*Enterococcus wangshanyuanii* CGMCC 1.15942	13.9	[11.1 - 17.2]	21.2	[18.9 - 23.6]	14.1	[11.7 - 17.0]	0.18
**'Enterococcus faecalis EN24 (ST228).fna'**	*Enterococcus plantarum* LMG 26214	14.2	[11.4 - 17.6]	21.2	[19.0 - 23.7]	14.4	[12.0 - 17.3]	1.51
**'Enterococcus faecalis CVM N52467 (ST228).fna'**	*Enterococcus plantarum* LMG 26214	14.2	[11.4 - 17.6]	21.2	[19.0 - 23.7]	14.5	[12.0 - 17.3]	1.59
**'Enterococcus faecalis R48 (ST228).fna'**	*Enterococcus wangshanyuanii* CGMCC 1.15942	13.9	[11.1 - 17.2]	21.2	[18.9 - 23.6]	14.2	[11.7 - 17.0]	0.31
**'Enterococcus faecalis R30 (ST228).fna'**	*Enterococcus plantarum* LMG 26214	14.2	[11.4 - 17.6]	21.2	[19.0 - 23.7]	14.5	[12.0 - 17.3]	1.68
**'Enterococcus faecalis CVM N54548 (ST228).fna'**	*Enterococcus plantarum* LMG 26214	14.2	[11.4 - 17.6]	21.2	[18.9 - 23.6]	14.5	[12.0 - 17.3]	1.54
**'Enterococcus faecalis CVM N52467 (ST228).fna'**	*Enterococcus lemanii* DSM 105069	13.3	[10.5 - 16.6]	21.2	[18.9 - 23.6]	13.6	[11.2 - 16.4]	0.4
**'Enterococcus faecalis G81 (ST1468).fna'**	*Enterococcus rivorum* LMG 25899	13.8	[11.0 - 17.1]	21.2	[19.0 - 23.7]	14.1	[11.6 - 16.9]	2.64
**'Enterococcus faecalis R30 (ST228).fna'**	*Enterococcus wangshanyuanii* CGMCC 1.15942	13.9	[11.1 - 17.3]	21.2	[18.9 - 23.6]	14.2	[11.8 - 17.0]	0.45
**'Enterococcus faecalis CVM N52662 (ST228).fna'**	*Enterococcus wangshanyuanii* CGMCC 1.15942	13.9	[11.1 - 17.2]	21.1	[18.9 - 23.6]	14.1	[11.7 - 17.0]	0.27

Query	Subject	d_0	C.I. d_0	d_4	C.I. d_4	d_6	C.I. d_6	Diff. G+C Percent
**'Enterococcus faecalis C144 (ST228).fna'**	*Enterococcus rivorum* DSM 104544	13.8	[11.1 - 17.2]	21.1	[18.8 - 23.5]	14.1	[11.7 - 16.9]	2.71
**'Enterococcus faecalis C138 (ST228).fna'**	*Enterococcus rivorum* DSM 104544	13.8	[11.1 - 17.2]	21.1	[18.8 - 23.5]	14.1	[11.7 - 16.9]	2.72
**'Enterococcus faecalis DSM 111623 (ST624).fna'**	*Enterococcus plantarum* LMG 26214	14.2	[11.4 - 17.6]	21.1	[18.9 - 23.5]	14.5	[12.0 - 17.3]	1.44
**'Enterococcus faecalis C138 (ST228).fna'**	*Enterococcus wangshanyuanii* CGMCC 1.15942	13.9	[11.1 - 17.2]	21.1	[18.9 - 23.6]	14.2	[11.7 - 17.0]	0.34
**'Enterococcus faecalis CVM N54548 (ST228).fna'**	*Enterococcus wangshanyuanii* CGMCC 1.15942	13.9	[11.1 - 17.2]	21.1	[18.9 - 23.6]	14.2	[11.7 - 17.0]	0.31
**'Enterococcus faecalis R30 (ST228).fna'**	*Enterococcus rivorum* LMG 25899	13.9	[11.1 - 17.2]	21.1	[18.8 - 23.5]	14.1	[11.7 - 17.0]	2.8
**'Enterococcus faecalis CVM N54548 (ST228).fna'**	*Enterococcus rivorum* DSM 104544	13.8	[11.1 - 17.2]	21.1	[18.8 - 23.5]	14.1	[11.7 - 16.9]	2.69
**'Enterococcus faecalis CVM N52662 (ST228).fna'**	*Enterococcus rivorum* DSM 104544	13.8	[11.0 - 17.2]	21.1	[18.8 - 23.5]	14.1	[11.7 - 16.9]	2.65
**'Enterococcus faecalis CVM N52587 (STx).fna'**	*Enterococcus rivorum* DSM 104544	13.8	[11.0 - 17.2]	21.1	[18.8 - 23.5]	14.1	[11.7 - 16.9]	2.62
**'Enterococcus faecalis DSM 111623 (ST624).fna'**	*Enterococcus rivorum* DSM 104544	13.8	[11.0 - 17.1]	21.1	[18.9 - 23.6]	14.1	[11.7 - 16.9]	2.59
**'Enterococcus faecalis EN24 (ST228).fna'**	*Enterococcus rivorum* LMG 25899	13.8	[11.1 - 17.2]	21.1	[18.8 - 23.5]	14.1	[11.7 - 16.9]	2.63
**'Enterococcus faecalis R30 (ST228).fna'**	*Enterococcus rivorum* DSM 104544	13.9	[11.1 - 17.2]	21.1	[18.8 - 23.5]	14.1	[11.7 - 17.0]	2.83
**'Enterococcus faecalis CVM N53420 (ST228).fna'**	*Enterococcus rivorum* LMG 25899	13.8	[11.0 - 17.2]	21.1	[18.8 - 23.5]	14.1	[11.7 - 16.9]	2.53
**'Enterococcus faecalis CVM N55265 (ST228).fna'**	*Enterococcus rivorum* LMG 25899	13.8	[11.1 - 17.2]	21.1	[18.9 - 23.5]	14.1	[11.7 - 16.9]	2.68
**'Enterococcus faecalis EN24 (ST228).fna'**	*Enterococcus rivorum* DSM 104544	13.8	[11.1 - 17.2]	21.1	[18.8 - 23.5]	14.1	[11.7 - 16.9]	2.66
**'Enterococcus faecalis CVM N55265 (ST228).fna'**	*Enterococcus wangshanyuanii* CGMCC 1.15942	13.9	[11.1 - 17.2]	21.1	[18.9 - 23.6]	14.2	[11.7 - 17.0]	0.33
**'Enterococcus faecalis CVM N52467 (ST228).fna'**	*Enterococcus rivorum* DSM 104544	13.8	[11.1 - 17.2]	21.1	[18.8 - 23.5]	14.1	[11.7 - 16.9]	2.75
**'Enterococcus faecalis C144 (ST228).fna'**	*Enterococcus wangshanyuanii* CGMCC 1.15942	13.9	[11.1 - 17.2]	21.1	[18.9 - 23.6]	14.2	[11.7 - 17.0]	0.33
**'Enterococcus faecalis C144 (ST228).fna'**	*Enterococcus rivorum* LMG 25899	13.8	[11.1 - 17.2]	21.1	[18.9 - 23.5]	14.1	[11.7 - 16.9]	2.68
**'Enterococcus faecalis DSM 111623 (ST624).fna'**	*Enterococcus rivorum* LMG 25899	13.8	[11.0 - 17.1]	21.1	[18.9 - 23.6]	14.1	[11.7 - 16.9]	2.56
**'Enterococcus faecalis CVM N52587 (STx).fna'**	*Enterococcus rivorum* LMG 25899	13.8	[11.0 - 17.2]	21.1	[18.8 - 23.5]	14.1	[11.7 - 16.9]	2.59
**'Enterococcus faecalis CVM N52662 (ST228).fna'**	*Enterococcus rivorum* LMG 25899	13.8	[11.0 - 17.2]	21.1	[18.9 - 23.5]	14.1	[11.7 - 16.9]	2.62
**'Enterococcus faecalis CVM N54548 (ST228).fna'**	*Enterococcus rivorum* LMG 25899	13.8	[11.1 - 17.2]	21.1	[18.8 - 23.5]	14.1	[11.7 - 16.9]	2.66

Query	Subject	d_0	C.I. d_0	d_4	C.I. d_4	d_6	C.I. d_6	Diff. G+C Percent
**'Enterococcus faecalis C138 (ST228).fna'**	*Enterococcus rivorum* LMG 25899	13.8	[11.1 - 17.2]	21.1	[18.9 - 23.5]	14.1	[11.7 - 16.9]	2.69
**'Enterococcus faecalis C146 (ST228).fna'**	*Enterococcus rivorum* DSM 104544	13.8	[11.1 - 17.2]	21.1	[18.9 - 23.5]	14.1	[11.7 - 16.9]	2.72
**'Enterococcus faecalis CVM N55265 (ST228).fna'**	*Enterococcus rivorum* DSM 104544	13.8	[11.1 - 17.2]	21.1	[18.8 - 23.5]	14.1	[11.7 - 16.9]	2.71
**'Enterococcus faecalis G81 (ST1468).fna'**	*Enterococcus wangshanyuanii* CGMCC 1.15942	13.9	[11.1 - 17.3]	21.1	[18.9 - 23.5]	14.2	[11.8 - 17.0]	0.29
**'Enterococcus faecalis C146 (ST228).fna'**	*Enterococcus rivorum* LMG 25899	13.8	[11.1 - 17.2]	21.1	[18.9 - 23.6]	14.1	[11.7 - 16.9]	2.69
**'Enterococcus faecalis CVM N52467 (ST228).fna'**	*Enterococcus rivorum* LMG 25899	13.8	[11.1 - 17.2]	21.1	[18.9 - 23.5]	14.1	[11.7 - 16.9]	2.72
**'Enterococcus faecalis DSM 111623 (ST624).fna'**	*Enterococcus quebecensis* LMG 26306	14.4	[11.6 - 17.8]	21.1	[18.9 - 23.5]	14.6	[12.2 - 17.4]	2.36
**'Enterococcus faecalis CVM N53420 (ST228).fna'**	*Enterococcus rivorum* DSM 104544	13.8	[11.0 - 17.2]	21.1	[18.8 - 23.5]	14.1	[11.7 - 16.9]	2.56
**'Enterococcus faecalis C116 (ST228).fna'**	*Enterococcus rivorum* DSM 104544	13.9	[11.1 - 17.2]	21.0	[18.8 - 23.4]	14.1	[11.7 - 16.9]	2.71
**'Enterococcus faecalis R30 (ST228).fna'**	*Vagococcus bubulae* SS1994	13.1	[10.4 - 16.5]	21.0	[18.7 - 23.4]	13.5	[11.1 - 16.3]	4.15
**'Enterococcus faecalis R48 (ST228).fna'**	*Enterococcus rivorum* LMG 25899	13.8	[11.0 - 17.2]	21.0	[18.8 - 23.4]	14.1	[11.7 - 16.9]	2.66
**'Enterococcus faecalis DSM 111623 (ST624).fna'**	*Enterococcus caccae* ATCC BAA-1240	14.1	[11.3 - 17.4]	21.0	[18.7 - 23.4]	14.3	[11.9 - 17.1]	1.71
**'Enterococcus faecalis C116 (ST228).fna'**	*Enterococcus rivorum* LMG 25899	13.9	[11.1 - 17.2]	21.0	[18.8 - 23.5]	14.1	[11.7 - 16.9]	2.68
**'Enterococcus faecalis R48 (ST228).fna'**	*Enterococcus rivorum* DSM 104544	13.8	[11.0 - 17.2]	21.0	[18.8 - 23.4]	14.1	[11.7 - 16.9]	2.69
**'Enterococcus faecalis CVM N53420 (ST228).fna'**	*Enterococcus caccae* ATCC BAA-1240	14.1	[11.3 - 17.4]	20.9	[18.6 - 23.3]	14.3	[11.9 - 17.2]	1.68
**'Enterococcus faecalis 209EA1 (ST624).fna'**	*Enterococcus caccae* ATCC BAA-1240	14.1	[11.3 - 17.5]	20.9	[18.7 - 23.4]	14.4	[11.9 - 17.2]	1.97
**'Enterococcus faecalis CVM N54548 (ST228).fna'**	*Enterococcus lemanii* DSM 105069	13.3	[10.5 - 16.6]	20.9	[18.7 - 23.4]	13.6	[11.2 - 16.4]	0.34
**'Enterococcus faecalis CVM N52587 (STx).fna'**	*Enterococcus hermanniensis* DSM 17122	13.3	[10.6 - 16.6]	20.9	[18.7 - 23.4]	13.6	[11.3 - 16.4]	0.14
**'Enterococcus faecalis CVM N52587 (STx).fna'**	*Enterococcus caccae* ATCC BAA-1240	14.1	[11.3 - 17.5]	20.9	[18.6 - 23.3]	14.3	[11.9 - 17.2]	1.73
**'Enterococcus faecalis G81 (ST1468).fna'**	*Enterococcus caccae* ATCC BAA-1240	14.1	[11.3 - 17.4]	20.9	[18.7 - 23.3]	14.3	[11.9 - 17.2]	1.79
**'Enterococcus faecalis C116 (ST228).fna'**	*Vagococcus bubulae* SS1994	13.1	[10.4 - 16.4]	20.9	[18.7 - 23.3]	13.5	[11.1 - 16.3]	4.03
**'Enterococcus faecalis CVM N52662 (ST228).fna'**	*Enterococcus caccae* ATCC BAA-1240	14.1	[11.3 - 17.5]	20.9	[18.6 - 23.3]	14.3	[11.9 - 17.2]	1.76
**'Enterococcus faecalis CVM N55265 (ST228).fna'**	*Enterococcus caccae* ATCC BAA-1240	14.1	[11.3 - 17.5]	20.8	[18.6 - 23.3]	14.4	[11.9 - 17.2]	1.83
**'Enterococcus faecalis G81 (ST1468).fna'**	*Enterococcus ureilyticus* LMG 26676	14.2	[11.4 - 17.6]	20.8	[18.5 - 23.2]	14.4	[12.0 - 17.3]	1.41

Query	Subject	d_0	C.I. d_0	d_4	C.I. d_4	d_6	C.I. d_6	Diff. G+C Percent
**'Enterococcus faecalis DSM 111623 (ST624).fna'**	*Enterococcus hermanniensis* DSM 17122	13.3	[10.6 - 16.6]	20.8	[18.5 - 23.2]	13.6	[11.3 - 16.4]	0.11
**'Enterococcus faecalis C144 (ST228).fna'**	*Enterococcus caccae* ATCC BAA-1240	14.1	[11.3 - 17.5]	20.8	[18.6 - 23.2]	14.4	[11.9 - 17.2]	1.82
**'Enterococcus faecalis C146 (ST228).fna'**	*Enterococcus caccae* ATCC BAA-1240	14.1	[11.3 - 17.5]	20.8	[18.6 - 23.3]	14.4	[11.9 - 17.2]	1.83
**'Enterococcus faecalis R30 (ST228).fna'**	*Enterococcus caccae* ATCC BAA-1240	14.1	[11.3 - 17.5]	20.8	[18.6 - 23.2]	14.4	[12.0 - 17.2]	1.94
**'Enterococcus faecalis C116 (ST228).fna'**	*Enterococcus caccae* ATCC BAA-1240	14.1	[11.3 - 17.5]	20.8	[18.5 - 23.2]	14.4	[11.9 - 17.2]	1.82
**'Enterococcus faecalis R30 (ST228).fna'**	*Enterococcus lemanii* DSM 105069	13.3	[10.6 - 16.6]	20.8	[18.5 - 23.2]	13.6	[11.2 - 16.4]	0.48
**'Enterococcus faecalis CVM N52467 (ST228).fna'**	*Enterococcus caccae* ATCC BAA-1240	14.1	[11.3 - 17.5]	20.8	[18.6 - 23.3]	14.4	[11.9 - 17.2]	1.86
**'Enterococcus faecalis EN24 (ST228).fna'**	*Enterococcus caccae* ATCC BAA-1240	14.1	[11.3 - 17.5]	20.8	[18.6 - 23.3]	14.4	[11.9 - 17.2]	1.78
**'Enterococcus faecalis CVM N54548 (ST228).fna'**	*Enterococcus caccae* ATCC BAA-1240	14.1	[11.3 - 17.5]	20.8	[18.5 - 23.2]	14.4	[11.9 - 17.2]	1.8
**'Enterococcus faecalis C138 (ST228).fna'**	*Enterococcus caccae* ATCC BAA-1240	14.1	[11.3 - 17.5]	20.8	[18.6 - 23.2]	14.4	[11.9 - 17.2]	1.84
**'Enterococcus faecalis R48 (ST228).fna'**	*Enterococcus caccae* ATCC BAA-1240	14.1	[11.3 - 17.5]	20.8	[18.5 - 23.2]	14.3	[11.9 - 17.2]	1.8
**'Enterococcus faecalis C116 (ST228).fna'**	*Enterococcus lemanii* DSM 105069	13.3	[10.5 - 16.6]	20.7	[18.5 - 23.2]	13.6	[11.2 - 16.4]	0.36
**'Enterococcus faecalis CVM N52662 (ST228).fna'**	*Enterococcus ureilyticus* LMG 26676	14.2	[11.4 - 17.6]	20.7	[18.4 - 23.1]	14.4	[12.0 - 17.3]	1.39
**'Enterococcus faecalis CVM N55265 (ST228).fna'**	*Enterococcus hermanniensis* DSM 17122	13.3	[10.6 - 16.7]	20.7	[18.4 - 23.1]	13.7	[11.3 - 16.5]	0.23
**'Enterococcus faecalis DSM 111623 (ST624).fna'**	*Enterococcus ureilyticus* LMG 26676	14.3	[11.5 - 17.7]	20.7	[18.4 - 23.1]	14.5	[12.1 - 17.3]	1.33
**'Enterococcus faecalis EN24 (ST228).fna'**	*Enterococcus ureilyticus* LMG 26676	14.3	[11.4 - 17.7]	20.6	[18.3 - 23.0]	14.5	[12.0 - 17.3]	1.4
**'Enterococcus faecalis R30 (ST228).fna'**	*Enterococcus ureilyticus* LMG 26676	14.3	[11.5 - 17.7]	20.6	[18.3 - 23.0]	14.5	[12.1 - 17.4]	1.57
**'Enterococcus faecalis C144 (ST228).fna'**	*Enterococcus ureilyticus* LMG 26676	14.3	[11.5 - 17.7]	20.6	[18.3 - 23.0]	14.5	[12.1 - 17.4]	1.45
**'Enterococcus faecalis C138 (ST228).fna'**	*Enterococcus ureilyticus* LMG 26676	14.3	[11.5 - 17.7]	20.6	[18.3 - 23.0]	14.5	[12.1 - 17.4]	1.46
**'Enterococcus faecalis CVM N52662 (ST228).fna'**	*Enterococcus hermanniensis* DSM 17122	13.3	[10.6 - 16.7]	20.6	[18.4 - 23.1]	13.7	[11.3 - 16.5]	0.17
**'Enterococcus faecalis C146 (ST228).fna'**	*Enterococcus ureilyticus* LMG 26676	14.3	[11.5 - 17.7]	20.6	[18.3 - 23.0]	14.5	[12.1 - 17.4]	1.46
**'Enterococcus faecalis CVM N52587 (STx).fna'**	*Enterococcus ureilyticus* LMG 26676	14.3	[11.4 - 17.6]	20.6	[18.4 - 23.0]	14.5	[12.0 - 17.3]	1.36
**'Enterococcus faecalis CVM N52467 (ST228).fna'**	*Enterococcus ureilyticus* LMG 26676	14.3	[11.5 - 17.7]	20.6	[18.4 - 23.0]	14.5	[12.1 - 17.3]	1.49
**'Enterococcus faecalis CVM N55265 (ST228).fna'**	*Enterococcus ureilyticus* LMG 26676	14.3	[11.4 - 17.7]	20.6	[18.4 - 23.0]	14.5	[12.0 - 17.3]	1.45

Query	Subject	d_0	C.I. d_0	d_4	C.I. d_4	d_6	C.I. d_6	Diff. G+C Percent
**'Enterococcus faecalis CVM N53420 (ST228).fna'**	*Enterococcus ureilyticus* LMG 26676	14.2	[11.4 - 17.6]	20.5	[18.3 - 23.0]	14.5	[12.0 - 17.3]	1.3
**'Enterococcus faecalis R48 (ST228).fna'**	*Enterococcus ureilyticus* LMG 26676	14.3	[11.4 - 17.6]	20.5	[18.3 - 22.9]	14.5	[12.0 - 17.3]	1.43
**'Enterococcus faecalis C116 (ST228).fna'**	*Enterococcus ureilyticus* LMG 26676	14.3	[11.5 - 17.7]	20.5	[18.3 - 22.9]	14.5	[12.1 - 17.3]	1.45
**'Enterococcus faecalis CVM N54548 (ST228).fna'**	*Enterococcus ureilyticus* LMG 26676	14.3	[11.5 - 17.7]	20.5	[18.3 - 22.9]	14.5	[12.1 - 17.4]	1.43
**'Enterococcus faecalis CVM N53420 (ST228).fna'**	*Enterococcus hermanniensis* DSM 17122	13.3	[10.6 - 16.7]	20.5	[18.3 - 22.9]	13.7	[11.3 - 16.5]	0.08
**'Enterococcus faecalis C116 (ST228).fna'**	*Enterococcus hermanniensis* DSM 17122	13.4	[10.6 - 16.7]	20.4	[18.2 - 22.8]	13.7	[11.3 - 16.5]	0.23
**'Enterococcus faecalis EN24 (ST228).fna'**	*Enterococcus hermanniensis* DSM 17122	13.4	[10.6 - 16.7]	20.4	[18.2 - 22.8]	13.7	[11.3 - 16.5]	0.18
**'Enterococcus faecalis R30 (ST228).fna'**	*Enterococcus hermanniensis* DSM 17122	13.4	[10.6 - 16.7]	20.4	[18.2 - 22.8]	13.7	[11.3 - 16.5]	0.35
**'Enterococcus faecalis CVM N54548 (ST228).fna'**	*Enterococcus hermanniensis* DSM 17122	13.4	[10.6 - 16.7]	20.4	[18.2 - 22.8]	13.7	[11.3 - 16.5]	0.21
**'Enterococcus faecalis R48 (ST228).fna'**	*Enterococcus hermanniensis* DSM 17122	13.3	[10.6 - 16.7]	20.3	[18.1 - 22.7]	13.7	[11.3 - 16.5]	0.21

Table 4: Strains in your dataset

Joint dataset of automatically determined closest type strains (if this mode was chosen), manually selected type strains (if selected accordingly) and the provided user strains, if provided (marked in yellow).

Strain	Authority	Other deposits	Synonyms	Base pairs	Percent G+C	No. proteins	Goldstamp	Bioproject accession	Biosample accession	Assembly accession	IMG OID
Enterococcus thailandicus DSM 21767	Tanasupawat et al. 2008	KCTC 13134; NRIC 107; NBRC 101867; FP48-3; PCU 282; TISTR 933	*Enterococcus thailandicus*	2773 849	36.6	2617		PRJNA270385	SAMN03267187	GCA_001886265	
Enterococcus canintestini DSM 21207	Naser et al. 2005	LMG 13590; CCUG 37857; CCUG 51312; CCM 7285	*Enterococcus canintestini*	2689 934	36.2	2517		PRJNA270385	SAMN03267163	GCA_001885735	
Enterococcus hermanniensis DSM 17122	Koort et al. 2004	LMG 12317; CCUG 48100	*Enterococcus hermanniensis*	2613 500	37.4	2550		PRJNA270385	SAMN03267173	GCA_001885945	
Vagococcus martis D7T301	Tak et al. 2017	KCTC 21069; JCM 31178	*Vagococcus martis*	2561 923	33.8	2472	Gp0208312	PRJNA224116	SAMN06241148	GCF_002026305	
Enterococcus cecorum DSM 20682	(Devriese et al. 1983) Williams et al. 1989	A60; LMG 12902; CIP 103676; NCIMB 702674; ATCC 43198; CCUG 27299; JCM 8724; NBRC 100674; NCTC 12421; NCDO 2674; NCFB 2674	*Enterococcus cecorum*; *Streptococcus cecorum*	2338 606	36.4	2363	Gp0013018	PRJNA165387	SAMN02256445	GCA_000379745	2515154031

Strain	Authority	Other deposits	Synonyms	Base pairs	Percent G+C	No. proteins	Goldstamp	Bioproject accession	Biosample accession	Assembly accession	IMG OID
Enterococcus wangshanyuanii MN05	Jin et al. 2017	CGMCC 1.15942; DSM 104047; MN05	*Enterococcus wangshanyuanii*	4155 954	37.3	4172	Gp0260508	PRJNA224116	SAMN07171652	GCF_002197645	
Enterococcus lemanii DSM 105069	Cotta et al. 2013	NRRL B-59661; CCUG 61260; PC32	*Enterococcus lemanii*	2715 351	37.2	2603	Gp0505788				2901272077
Enterococcus xiangfangensis DSM 105127	Li et al. 2014	11097; LMG 27495; NCIMB 14834; DSM 105127	*Enterococcus xiangfangensis*	2654 637	39.0	2594	Gp0505791				2901274788
Candidatus Enterococcus avicola CHK172-16539	Gilroy et al. 2021		*Candidatus* Enterococcus avicola	2214 113	36.9	2261		PRJNA543206	SAMN15816900	GCA_019116505	
Enterococcus rivorum DSM 104544	Niemi et al. 2012	LMG 25899; DSM 104544; CCM 7986; HAMBI 3055; S299	*Enterococcus rivorum*	3791 979	34.9	3596	Gp0505789				2913591362
Vagococcus bubulae SS1994	Shewmaker et al. 2019	LMG 30164; CCUG 70831	*Vagococcus bubulae*	2493 517	33.5	2360		PRJNA359498	SAMN06609019	GCA_003950315	
Vagococcus vulneris SS1995	Shewmaker et al. 2019	LMG 30165; CCUG 70832	*Vagococcus vulneris*	2341 193	34.6	2199		PRJNA359498	SAMN06609020	GCA_003950515	
Enterococcus songbeiensis NCIMB 15179	Li and Gu 2019	85-4; CCM 8923	*Enterococcus songbeiensis*	2268 269	39.7	2210		PRJDB7793	SAMD00164309	GCA_005405265	
Enterococcus rotai DSM 102982	Sedláček et al. 2013	LMG 26678; CCUG 61593; DSM 102982; CCM 4630	*Enterococcus rotai*	3823 370	36.1	3602	Gp0538786				2928218588

Strain	Authority	Other deposits	Synonyms	Base pairs	Percent G+C	No. proteins	Goldstamp	Bioproject accession	Biosample accession	Assembly accession	IMG OID
Enterococcus lactis DSM 23655	Morandi et al. 2012 emend. Li and Gu 2021	LMG 25958; DSM 23655; CCM 8412; BT159	*Enterococcus lactis*	2859 220	38.1	2799	Gp0538720				2928549275
Enterococcus saigonensis JCM 31193	Harada et al. 2016	CCUG 68827; VE80	*Enterococcus saigonensis*	2844 986	36.3	2700		PRJDB9118	SAMD00198474	GCA_011397115	
Enterococcus lactis CCM 8412	Morandi et al. 2012 emend. Li and Gu 2021	LMG 25958; DSM 23655; CCM 8412; BT159	*Enterococcus lactis*	2862 704	38.1	2745		PRJDB7793	SAMD00255145	GCA_015751045	
Enterococcus massiliensis AM1	Le Page et al. 2016	DSM 100308; CSUR P1927	*Enterococcus massiliensis*	2711 493	39.6	2614	Gp0144269	PRJEB9522	SAMEA3443460	GCA_001050095	
Enterococcus mediterraneensis Marseille-P4358	Takakura et al. 2019	CSUR P4358	*Enterococcus mediterraneensis*	2699 190	40.9	2598		PRJNA224116	SAMEA4983427	GCF_900604485	
Aequorivita lutea q18	Zhang et al. 2020	CICC 24821; KCTC 72764	*Aequorivita lutea*	3286 739	42.7	3418		PRJNA565545	SAMN12751400	GCA_009668655	
Enterococcus dispar ATCC 51266	Collins et al. 1991	LMG 13521; CIP 103646; NCIMB 13000; CCUG 33309; DSM 6630; NBRC 100678; E18-1; HAMBI 2231; NCFB 2821	*Enterococcus dispar*	2812 918	37.2	2637	Gp0037006	PRJNA191887	SAMN02596962	GCA_000407585	2545824666

Strain	Authority	Other deposits	Synonyms	Base pairs	Percent G+C	No. proteins	Goldstamp	Bioproject accession	Biosample accession	Assembly accession	IMG OID
Enterococcus haemoperoxidus ATCC BAA-382	Švec et al. 2001	440; LMG 19487; CIP 107129; CCUG 45916; DSM 15920; NBRC 100709; CCM 4851	*Enterococcus haemoperoxidus*	3578 766	35.7	3207	Gp0037011	PRJNA191901	SAMN02596950	GCA_000407165	2545824662
Enterococcus caccae ATCC BAA-1240	Carvalho et al. 2006	2215-02; CCUG 51564; DSM 19114; SS-1777	*Enterococcus caccae*	3547 077	35.8	3246	Gp0037005	PRJNA191898	SAMN02596947	GCA_000407145	2545824664
Enterococcus moraviensis ATCC BAA-383	Švec et al. 2001	330; LMG 19486; CIP 107130; CCUG 45913; DSM 15919; NBRC 100710; CCM 4856	*Enterococcus moraviensis*	3586 110	36.0	3341	Gp0037015	PRJNA191905	SAMN02596953	GCA_000407445	2545824649
Enterococcus porcinus ATCC 700913	Teixeira et al. 2001	CIP 107172; NCIMB 13634; CCUG 43229; DS 1390-83	*Enterococcus porcinus*	3056 699	35.0	2821	Gp0036838	PRJNA191912	SAMN02596958	GCA_000407205	2545824674

Strain	Authority	Other deposits	Synonyms	Base pairs	Percent G+C	No. proteins	Goldstamp	Bioproject accession	Biosample accession	Assembly accession	IMG OID
Enterococcus casseliflavus NBRC 100478	(ex Vaughan et al. 1979) Collins et al. 1984	LMG 10745; CIP 103018; NRRL B-3502; NCIMB 11449; ATCC 25788; CCUG 18657; DSM 20680; JCM 8723; NCTC 12361; MUTK 20; NCDO 2372; NCFB 2372; NCIB 11449	*Enterococcus casseliflavus*; *Streptococcus casseliflavus*; *Streptococcus faecium* subsp. *casseliflavus*	3498 264	42.4	3339	Gp0023879	PRJDB260	SAMD00045727	GCA_001544095	
Enterococcus durans NBRC 100479	(ex Sherman and Wing 1937) Collins et al. 1984	98D; ATCC 19432; DSM 20633; NCTC 8307; CCM 5612; NCDO 596; NCFB 596	*Enterococcus durans*; *Streptococcus durans*	3017 301	37.8	2874	Gp0023771	PRJDB261	SAMD00045728	GCA_001544215	

Strain	Authority	Other deposits	Synonyms	Base pairs	Percent G+C	No. proteins	Goldstamp	Bioproject accession	Biosample accession	Assembly accession	IMG OID
Enterococcus faecalis NBRC 100480	(Andrewes and Horder 1906) Schleifer and Kilpper-Bälz 1984	LMG 7937; CIP 103015; NCIMB 775; ATCC 19433; ATCC 19433-U; CCUG 19916; DSM 20478; JCM 5803; JCM 8726; NBIMCC 3360; NBRC 100481; NCTC 775; HAMBI 1711; NCAIM B.01312; NCDO 581; NCFB 581; NCIB 775	*Enterococcus faecalis*; *Streptococcus faecalis*	2833 208	37.5	2734	Gp0023751	PRJDB262	SAMD00045729	GCA_001544235	
Enterococcus wangshanyuanii CGMCC 1.15942	Jin et al. 2017	CGMCC 1.15942; DSM 104047; MN05	*Enterococcus wangshanyuanii*	3967 296	37.3	3969		PRJDB10509	SAMD00245100	GCA_014644255	
Enterococcus quebecensis LMG 26306	Sistek et al. 2012	CCUG 59306; DSM 23327; CCRI 16985	*Enterococcus quebecensis*	3162 767	35.1	2788		PRJNA224116	SAMN05420588	GCF_001730365	
Enterococcus ureasiticus DSM 23328	Sistek et al. 2012	LMG 26304; CCUG 59304; CCRI 16986	*Enterococcus ureasiticus*	3585 398	35.8	3255		PRJNA224116	SAMN05420582	GCF_001730285	
Enterococcus termitis LMG 8895	Švec et al. 2006	DSM 22803; CCM 7300	*Enterococcus termitis*	4155 153	36.8	3932		PRJNA224116	SAMN05420590	GCF_001730305	
Enterococcus silesiacus LMG 23085	Švec et al. 2006	DSM 22801; CCM 7319; W442	*Enterococcus silesiacus*	3928 141	36.4	3559	Gp0150893	PRJNA226735	SAMN04296138	GCA_001465115	

Strain	Authority	Other deposits	Synonyms	Base pairs	Percent G+C	No. proteins	Goldstamp	Bioproject accession	Biosample accession	Assembly accession	IMG OID
Enterococcus plantarum LMG 26214	Švec et al. 2012	DSM 26408; CCM 7889; C27	*Enterococcus plantarum*	3135 404	36.0	2907		PRJNA224116	SAMN05420586	GCF_001730295	
Enterococcus rotai LMG 26678	Sedláček et al. 2013	LMG 26678; CCUG 61593; DSM 102982; CCM 4630	*Enterococcus rotai*	3583 054	36.1	3253	Gp0150894	PRJNA226735	SAMN04296136	GCA_001465345	
Enterococcus rivorum LMG 25899	Niemi et al. 2012	LMG 25899; DSM 104544; CCM 7986; HAMBI 3055; S299	*Enterococcus rivorum*	3806 353	34.9	3542		PRJNA224116	SAMN05420592	GCF_001742285	
Enterococcus ureilyticus LMG 26676	Sedláček et al. 2013	CCUG 48799; DSM 102981; CCM 4629	*Enterococcus ureilyticus*	3472 706	36.1	3240		PRJNA224116	SAMN05420589	GCF_001730315	
Enterococcus xiangfangensis NCIMB 14834	Li et al. 2014	11097; LMG 27495; NCIMB 14834; DSM 105127	*Enterococcus xiangfangensis*	2647 587	39.0	2576		PRJDB7793	SAMD00164314	GCA_005405365	
Enterococcus faecalis 209EA1 (ST624).fna				2671 742	37.7	2455					
Enterococcus faecalis C116 (ST228).fna				2723 129	37.6	2566					
Enterococcus faecalis C138 (ST228).fna				2695 188	37.6	2571					
Enterococcus faecalis C144 (ST228).fna				2697 493	37.6	2542					
Enterococcus faecalis C146 (ST228).fna				2697 692	37.6	2581					

Strain	Authority	Other deposits	Synonyms	Base pairs	Percent G+C	No. proteins	Goldstamp	Bioproject accession	Biosample accession	Assembly accession	IMG OID
Enterococcus faecalis CVM N52467 (ST228).fna				2676 969	37.6	2537					
Enterococcus faecalis CVM N52587 (STx).fna				2746 314	37.5	2598					
Enterococcus faecalis CVM N52662 (ST228).fna				2775 983	37.5	2617					
Enterococcus faecalis CVM N53420 (ST228).fna				2866 934	37.4	2727					
Enterococcus faecalis CVM N54548 (ST228).fna				2685 933	37.6	2520					
Enterococcus faecalis CVM N55265 (ST228).fna				2699 907	37.6	2536					
Enterococcus faecalis DSM 111623 (ST624).fna				2705 646	37.5	2531					
Enterococcus faecalis EN24 (ST228).fna				2763 117	37.5	2602					
Enterococcus faecalis G81 (ST1468).fna				2835 934	37.5	2732					
Enterococcus faecalis R30 (ST228).fna				2647 103	37.7	2511					
Enterococcus faecalis R48 (ST228).fna				2812 753	37.6	2725					

Methods, Results and References

The genome sequence data were uploaded to the Type (Strain) Genome Server (TYGS), a free bioinformatics platform available under https://tygs.dsmz.de, for a whole genome-based taxonomic analysis [1]. The analysis also made use of recently introduced methodological updates and features [2]. Information on nomenclature, synonymy and associated taxonomic literature was provided by TYGS's sister database, the List of Prokaryotic names with Standing in Nomenclature (LPSN, available at https://lpsn.dsmz.de) [2]. The results were provided by the TYGS on 2024-06-25. The TYGS analysis was subdivided into the following steps:

Determination of closely related type strains

Determination of closest type strain genomes was done in two complementary ways: First, all user genomes were compared against all type strain genomes available in the TYGS database via the MASH algorithm, a fast approximation of intergenomic relatedness [3], and, the ten type strains with the smallest MASH distances chosen per user genome. Second, an additional set of ten closely related type strains was determined via the 16S rDNA gene sequences. These were extracted from the user genomes using RNAmmer [4] and each sequence was subsequently BLASTed [5] against the 16S rDNA gene sequence of each of the currently 21278 type strains available in the TYGS database. This was used as a proxy to find the best 50 matching type strains (according to the bitscore) for each user genome and to subsequently calculate precise distances using the Genome BLAST Distance Phylogeny approach (GBDP) under the algorithm 'coverage' and distance formula d_5 [6]. These distances were finally used to determine the 10 closest type strain genomes for each of the user genomes.

Pairwise comparison of genome sequences

For the phylogenomic inference, all pairwise comparisons among the set of genomes were conducted using GBDP and accurate intergenomic distances inferred under the algorithm 'trimming' and distance formula d_5 [6]. 100 distance replicates were calculated each. Digital DDH values and confidence intervals were calculated using the recommended settings of the GGDC 4.0 [2,6].

Phylogenetic inference

The resulting intergenomic distances were used to infer a balanced minimum evolution tree with branch support via FASTME 2.1.6.1 including SPR postprocessing [7]. Branch support was inferred from 100 pseudo-bootstrap replicates each. The trees were rooted at the midpoint [8] and visualized with PhyD3 [9].

Type-based species and subspecies clustering

The type-based species clustering using a 70% dDDH radius around each of the 38 type strains was done as previously described [1]. The resulting groups are shown in Table 1 and 4. Subspecies clustering was done using a 79% dDDH threshold as previously introduced [10].

Results

Type-based species and subspecies clustering

The resulting species and subspecies clusters are listed in Table 4, whereas the taxonomic identification of the query strains is found in Table 1. Briefly, the clustering yielded 31 species clusters and the provided query strains were assigned to 1 of these. Moreover, user strains were located in 1 of 31 subspecies clusters.

Figure caption SSU tree

Figure 1. Tree inferred with FastME 2.1.6.1 [7] from GBDP distances calculated from 16S rDNA gene sequences. The branch lengths are scaled in terms of GBDP distance formula d_5. The numbers above branches are GBDP pseudo-bootstrap support values > 60 % from 100 replications, with an average branch support of 59.2 %. The tree was rooted at the midpoint [8].

Figure caption genome tree

Figure 2. Tree inferred with FastME 2.1.6.1 [7] from GBDP distances calculated from genome sequences. The branch lengths are scaled in terms of GBDP distance formula d_5. The numbers above branches are GBDP pseudo-bootstrap support values > 60 % from 100 replications, with an average branch support of 38.4 %. The tree was rooted at the midpoint [8].

References

[1] Meier-Kolthoff JP, Göker M. TYGS is an automated high-throughput platform for state-of-the-art genome-based taxonomy. Nat. Commun. 2019;10: 2182. DOI: 10.1038/s41467-019-10210-3

[2] Meier-Kolthoff JP, Sardà Carbasse J, Peinado-Olarte RL, Göker M. TYGS and LPSN: a database tandem for fast and reliable genome-based classification and nomenclature of prokaryotes. Nucleic Acid Res. 2022;50: D801–D807. DOI: 10.1093/nar/gkab902

[3] Ondov BD, Treangen TJ, Melsted P, et al. Mash: Fast genome and metagenome distance estimation using MinHash. Genome Biol 2016;17: 1–14. DOI: 10.1186/s13059-016-0997-x

[4] Lagesen K, Hallin P. RNAmmer: consistent and rapid annotation of ribosomal RNA genes. Nucleic Acids Res. Oxford Univ Press; 2007;35: 3100–3108. DOI: 10.1093/nar/gkm160

[5] Camacho C, Coulouris G, Avagyan V, Ma N, Papadopoulos J, Bealer K, et al. BLAST+: architecture and applications. BMC Bioinformatics. 2009;10: 421. DOI: 10.1186/1471-2105-10-421

[6] Meier-Kolthoff JP, Auch AF, Klenk H-P, Göker M. Genome sequence-based species delimitation with confidence intervals and improved distance functions. BMC Bioinformatics. 2013;14: 60. DOI: 10.1186/1471-2105-14-60

[7] Lefort V, Desper R, Gascuel O. FastME 2.0: A comprehensive, accurate, and fast distance-based phylogeny inference program. Mol Biol Evol. 2015;32: 2798–2800. DOI: 10.1093/molbev/msv150

[8] Farris JS. Estimating phylogenetic trees from distance matrices. Am Nat. 1972;106: 645–667.

[9] Kreft L, Botzki A, Coppens F, Vandepoele K, Van Bel M. PhyD3: A phylogenetic tree viewer with extended phyloXML support for functional genomics data visualization. Bioinformatics. 2017;33: 2946–2947. DOI: 10.1093/bioinformatics/btx324

[10] Meier-Kolthoff JP, Hahnke RL, Petersen J, Scheuner C, Michael V, Fiebig A, et al. Complete genome sequence of DSM 30083^T, the type strain (U5/41^T) of *Escherichia coli*, and a proposal for delineating subspecies in microbial taxonomy. Stand Genomic Sci. 2014;9: 2. DOI: 10.1186/1944-3277-9-2
