# Supplementary material for: Hidden diversity in Enterococcus faecalis revealed by CRISPR2 screening: eco-evolutionary insights into a novel subspecies
Source: Microbiol Spectr. 2025 Sep 8;13(10):e01428-25. doi: 10.1128/spectrum.01428-25 (PMC12502612; doi:10.1128/spectrum.01428-25)
Supplement: Data S7 — TYGS analysis report for the 39 E. faecalis subspecies A genomes. [file spectrum.01428-25-s0007.pdf]

TYPE (STRAIN) GENOME SERVER

JOB SUMMARY

PRINT DATE:	2024-06-19 20:16:04 +0200
JOB ID:	2ff4240b-23e7-45ae-9535-a2e45faa7a2f
RESULT PAGE:	https://tygs.dsmz.de/user_results/show?guid=2ff4240b-23e7-45ae-9535-a2e45faa7a2f

Table 1: Phylogenies

Publication-ready versions of both the genome-scale GBDP tree and the 16S rRNA gene sequence tree can be customized and exported either in SVG (vector graphic) or PNG format from within the phylogeny viewers in your TYGS result page. For publications the **SVG format is recommended** because it is lossless, always keeps its high resolution and can also be easily converted to other popular formats such as PDF or EPS. Please follow the link provided above!

Table 2: Identification

The below list contains the result of the TYGS species identification routine.

Explanation of remarks that might occur in the below table:

remark [R1]: The TYGS type strain database is automatically updated on an almost daily basis. However, if a particular type strain genome is not available in the TYGS database, this can have several reasons which are detailed in the FAQ. You can request an extended 16S rRNA gene analysis via the 16S tree viewer found in your result page to detect **not yet genome-sequenced** type strains relevant for your study.

remark [R2]: > 70% dDDH value (formula d_4) and (almost) minimal dDDH values for gene-content formulae d_0 and d_6 indicate a potentially unreliable identification result and should thus be checked via the 16S rRNA gene sequence similarity. Such strong deviations can, in principle, be caused by sequence contamination.

remark [R3]: G+C content difference of > 1 % indicates a potentially unreliable identification result because within species G+C content varies no more than 1 %, if computed from genome sequences (PMID: 24505073).

Strain	Conclusion	Identification result	Remark
**'14EA1_(ST147)'**	belongs to known species	*Enterococcus faecalis*	
**'20_SD_W_06_(ST368)'**	belongs to known species	*Enterococcus faecalis*	see [R2]
**'209EA1_(ST624)'**	belongs to known species	*Enterococcus faecalis*	
**'244_EFLS_(ST443)'**	belongs to known species	*Enterococcus faecalis*	see [R2]
**'302EA1_(ST122)'**	belongs to known species	*Enterococcus faecalis*	
**'732_EFLS_(ST16)'**	belongs to known species	*Enterococcus faecalis*	see [R2]
**'4928STDY7071351_(ST30)'**	belongs to known species	*Enterococcus faecalis*	see [R2]
**'4928STDY7071435_(ST40)'**	belongs to known species	*Enterococcus faecalis*	see [R2]
**'15224_(ST16)'**	belongs to known species	*Enterococcus faecalis*	see [R2]
**'19910_(ST116)'**	belongs to known species	*Enterococcus faecalis*	see [R2]
**'BIOML_A5_(ST875)'**	belongs to known species	*Enterococcus faecalis*	see [R2]
**'BT00.E.21_(ST23)'**	belongs to known species	*Enterococcus faecalis*	see [R2]
**'C116_(ST228)'**	belongs to known species	*Enterococcus faecalis*	see [R2]
**'C138_(ST228)'**	belongs to known species	*Enterococcus faecalis*	
**'CL6870_(ST103)'**	belongs to known species	*Enterococcus faecalis*	see [R2]

Strain	Conclusion	Identification result	Remark
**'CL8682_(ST769)'**	belongs to known species	*Enterococcus faecalis*	see [R2]
**'CL9172_(ST21)'**	belongs to known species	*Enterococcus faecalis*	see [R2]
**'CL9314_(ST21)'**	belongs to known species	*Enterococcus faecalis*	see [R2]
**'CL9772_(ST525)'**	belongs to known species	*Enterococcus faecalis*	see [R2]
**'CL9797_(ST525)'**	belongs to known species	*Enterococcus faecalis*	see [R2]
**'CL9824_(ST631)'**	belongs to known species	*Enterococcus faecalis*	see [R2]
**'CL9924_(ST6)'**	belongs to known species	*Enterococcus faecalis*	see [R2]
**'CL9943_(ST4)'**	belongs to known species	*Enterococcus faecalis*	see [R2]
**'CL11199_(ST778)'**	belongs to known species	*Enterococcus faecalis*	see [R2]
**'CVM N52662_(ST228)'**	belongs to known species	*Enterococcus faecalis*	
**'CVM N53420_(ST228)'**	belongs to known species	*Enterococcus faecalis*	
**'CVM_N52587_(STx)'**	belongs to known species	*Enterococcus faecalis*	
**'CVM_N54595_(ST192)'**	belongs to known species	*Enterococcus faecalis*	see [R2]
**'CVM_N55265_(ST228)'**	belongs to known species	*Enterococcus faecalis*	
**'CVM_N60027F_(ST862)'**	belongs to known species	*Enterococcus faecalis*	see [R2]
**'D32_(ST40)'**	belongs to known species	*Enterococcus faecalis*	
**'DSM111623_(ST624)'**	belongs to known species	*Enterococcus faecalis*	see [R2]
**'E1_(ST40)'**	belongs to known species	*Enterococcus faecalis*	see [R2]
**'EF348_(ST16)'**	belongs to known species	*Enterococcus faecalis*	see [R2]
**'EF349_(ST631)'**	belongs to known species	*Enterococcus faecalis*	see [R2]<br>see [R3]
**'EN24_(ST228)'**	belongs to known species	*Enterococcus faecalis*	
**'EN242_(ST82)'**	belongs to known species	*Enterococcus faecalis*	see [R2]
**'EN788_(ST82)'**	belongs to known species	*Enterococcus faecalis*	see [R2]
**'EnGen0400_(ST86)'**	belongs to known species	*Enterococcus faecalis*	
**'F_(ST712)'**	belongs to known species	*Enterococcus faecalis*	see [R2]
**'F1_(ST72)'**	belongs to known species	*Enterococcus faecalis*	
**'G81_(ST1468)'**	belongs to known species	*Enterococcus faecalis*	
**'KUB3007_(ST729)'**	belongs to known species	*Enterococcus faecalis*	see [R2]
**'L14_(ST330)'**	belongs to known species	*Enterococcus faecalis*	
**'LB00.E.122_(ST21)'**	belongs to known species	*Enterococcus faecalis*	see [R2]
**'Merz96_(ST103)'**	belongs to known species	*Enterococcus faecalis*	see [R2]
**'P9 CL A7_(ST4)'**	belongs to known species	*Enterococcus faecalis*	see [R2]
**'Praia.S.M2.C4_(ST62)'**	belongs to known species	*Enterococcus faecalis*	see [R2]
**'R48_(ST228)'**	belongs to known species	*Enterococcus faecalis*	see [R2]
**'T11_(ST65)'**	belongs to known species	*Enterococcus faecalis*	see [R2]

Table 3: Pairwise comparisons of user genomes vs. type-strain genomes

The overall number of pairwise comparisons was too large for a proper display and was thus reduced to only those comparisons having a digital DDH value ≥ 65 % in at least one of the three formulae d_0, d_4 and d_6.

The following table contains the pairwise dDDH values between your user genomes and the selected type-strain genomes. The dDDH values are provided along with their confidence intervals (C.I.) for the three different GBDP formulas:

- formula d_0 (a.k.a. GGDC formula 1): length of all HSPs divided by total genome length
- formula d_4 (a.k.a. GGDC formula 2): sum of all identities found in HSPs divided by overall HSP length
- formula d_6 (a.k.a. GGDC formula 3): sum of all identities found in HSPs divided by total genome length

Note: Formula d_4 is independent of genome length and is thus robust against the use of incomplete draft genomes. For other reasons for preferring formula d_4, see the FAQ.

Query	Subject	d_0	C.I. d_0	d_4	C.I. d_4	d_6	C.I. d_6	Diff. G+C Percent
**'CL9172_(ST21).fasta'**	**'CL9314_(ST21).fasta'**	99.4	[98.8 - 99.7]	99.9	[99.8 - 100.0]	99.7	[99.5 - 99.9]	0.01
**'EN242_(ST82).fna'**	**'EN788_(ST82).fna'**	99.2	[98.5 - 99.6]	99.9	[99.9 - 100.0]	99.7	[99.4 - 99.8]	0.12
**'CL9824_(ST631).fasta'**	**'EF349_(ST631).fna'**	96.9	[95.2 - 98.0]	99.9	[99.9 - 100.0]	98.5	[97.6 - 99.0]	0.92
**'CL9172_(ST21).fasta'**	**'LB00.E.122_(ST21).fasta'**	98.6	[97.6 - 99.2]	99.9	[99.8 - 99.9]	99.4	[98.9 - 99.6]	0.09
**'C116_(ST228).fna'**	**'EN24_(ST228).fna'**	96.0	[93.9 - 97.3]	99.8	[99.6 - 99.9]	97.9	[96.8 - 98.6]	0.05
**'CVM N52662_(ST228).fna'**	**'CVM_N55265_(ST228).fna'**	99.4	[98.8 - 99.7]	99.8	[99.6 - 99.9]	99.7	[99.5 - 99.9]	0.06
**'C116_(ST228).fna'**	**'R48_(ST228).fna'**	97.1	[95.4 - 98.2]	99.8	[99.6 - 99.9]	98.5	[97.7 - 99.1]	0.02
**'C116_(ST228).fna'**	**'CVM_N52587_(STx).fna'**	94.8	[92.5 - 96.5]	99.8	[99.6 - 99.9]	97.2	[95.8 - 98.1]	0.09
**'C116_(ST228).fna'**	**'CVM_N55265_(ST228).fna'**	96.1	[94.1 - 97.4]	99.8	[99.6 - 99.9]	97.9	[96.8 - 98.7]	0.0
**'CVM_N52587_(STx).fna'**	**'CVM_N55265_(ST228).fna'**	99.6	[99.1 - 99.8]	99.8	[99.7 - 99.9]	99.8	[99.6 - 99.9]	0.09
**'C116_(ST228).fna'**	**'CVM N52662_(ST228).fna'**	97.0	[95.3 - 98.1]	99.8	[99.6 - 99.9]	98.5	[97.6 - 99.1]	0.06
**'CVM_N55265_(ST228).fna'**	**'R48_(ST228).fna'**	92.6	[89.8 - 94.7]	99.7	[99.5 - 99.8]	95.8	[94.0 - 97.0]	0.02
**'C138_(ST228).fna'**	**'R48_(ST228).fna'**	92.3	[89.5 - 94.5]	99.7	[99.5 - 99.8]	95.6	[93.8 - 96.9]	0.03
**'CL9314_(ST21).fasta'**	**'LB00.E.122_(ST21).fasta'**	98.3	[97.1 - 99.0]	99.7	[99.6 - 99.9]	99.2	[98.6 - 99.5]	0.08
**'C138_(ST228).fna'**	**'CVM_N52587_(STx).fna'**	95.4	[93.2 - 96.9]	99.7	[99.4 - 99.8]	97.5	[96.3 - 98.4]	0.1
**'CVM N52662_(ST228).fna'**	**'CVM_N52587_(STx).fna'**	98.4	[97.3 - 99.1]	99.7	[99.5 - 99.8]	99.3	[98.7 - 99.6]	0.03
**'CVM_N52587_(STx).fna'**	**'R48_(ST228).fna'**	91.5	[88.5 - 93.8]	99.7	[99.4 - 99.8]	95.0	[93.1 - 96.4]	0.07
**'CL6870_(ST103).fasta'**	**'Merz96_(ST103).fna'**	95.7	[93.6 - 97.1]	99.7	[99.5 - 99.8]	97.7	[96.5 - 98.5]	0.33
**'CVM_N52587_(STx).fna'**	**'EN24_(ST228).fna'**	94.0	[91.5 - 95.9]	99.7	[99.5 - 99.8]	96.7	[95.2 - 97.7]	0.04
**'C138_(ST228).fna'**	**'CVM N52662_(ST228).fna'**	94.9	[92.6 - 96.5]	99.7	[99.4 - 99.8]	97.2	[95.9 - 98.1]	0.07

Query	Subject	d_0	C.I. d_0	d_4	C.I. d_4	d_6	C.I. d_6	Diff. G+C Percent
**'C116_(ST228).fna'**	**'C138_(ST228).fna'**	94.6	[92.1 - 96.3]	99.7	[99.5 - 99.8]	97.0	[95.6 - 98.0]	0.01
**'C138_(ST228).fna'**	**'CVM_N55265_(ST228).fna'**	96.6	[94.7 - 97.8]	99.7	[99.5 - 99.8]	98.2	[97.2 - 98.9]	0.01
**'EN24_(ST228).fna'**	**'R48_(ST228).fna'**	94.1	[91.6 - 95.9]	99.6	[99.3 - 99.7]	96.7	[95.2 - 97.8]	0.03
**'CVM N52662_(ST228).fna'**	**'R48_(ST228).fna'**	93.9	[91.3 - 95.7]	99.6	[99.4 - 99.8]	96.6	[95.0 - 97.6]	0.04
**'C138_(ST228).fna'**	**'CVM N53420_(ST228).fna'**	92.0	[89.1 - 94.2]	99.6	[99.3 - 99.7]	95.3	[93.5 - 96.7]	0.16
**'209EA1_(ST624).fna'**	**'G81_(ST1468).fna'**	94.7	[92.3 - 96.4]	99.6	[99.3 - 99.8]	97.1	[95.7 - 98.0]	0.18
**'CVM_N55265_(ST228).fna'**	**'EN24_(ST228).fna'**	94.8	[92.4 - 96.4]	99.6	[99.4 - 99.8]	97.1	[95.8 - 98.1]	0.05
**'C138_(ST228).fna'**	**'EN24_(ST228).fna'**	94.5	[92.1 - 96.2]	99.5	[99.1 - 99.7]	97.0	[95.5 - 97.9]	0.06
**'C116_(ST228).fna'**	**'CVM N53420_(ST228).fna'**	93.0	[90.3 - 95.0]	99.5	[99.2 - 99.7]	96.0	[94.3 - 97.2]	0.15
**'CVM N53420_(ST228).fna'**	**'CVM_N55265_(ST228).fna'**	92.5	[89.7 - 94.6]	99.4	[99.0 - 99.6]	95.6	[93.8 - 96.9]	0.15
**'CVM N52662_(ST228).fna'**	**'EN24_(ST228).fna'**	94.1	[91.6 - 95.9]	99.4	[99.0 - 99.6]	96.7	[95.2 - 97.7]	0.01
**'CVM N53420_(ST228).fna'**	**'CVM_N52587_(STx).fna'**	91.7	[88.7 - 93.9]	99.3	[99.0 - 99.6]	95.1	[93.2 - 96.5]	0.05
**'CVM N53420_(ST228).fna'**	**'R48_(ST228).fna'**	93.5	[90.9 - 95.4]	99.3	[98.9 - 99.6]	96.3	[94.7 - 97.4]	0.12
**'732_EFLS_(ST16).fna'**	**'EF348_(ST16).fna'**	96.9	[95.2 - 98.0]	99.3	[98.9 - 99.6]	98.4	[97.4 - 99.0]	0.17
**'732_EFLS_(ST16).fna'**	**'15224_(ST16).fna'**	91.6	[88.6 - 93.9]	99.1	[98.7 - 99.4]	95.0	[93.1 - 96.4]	0.29
**'4928STDY7071435_(ST40).fna'**	**'E1_(ST40).fna'**	97.1	[95.4 - 98.2]	99.1	[98.6 - 99.4]	98.5	[97.6 - 99.0]	0.37
**'CVM N52662_(ST228).fna'**	**'CVM N53420_(ST228).fna'**	94.7	[92.3 - 96.3]	99.0	[98.5 - 99.4]	97.0	[95.6 - 98.0]	0.09
**'CVM N53420_(ST228).fna'**	**'EN24_(ST228).fna'**	93.3	[90.6 - 95.3]	99.0	[98.5 - 99.4]	96.1	[94.5 - 97.3]	0.1
**'CL9772_(ST525).fasta'**	**'CL9797_(ST525).fasta'**	97.5	[96.0 - 98.4]	98.8	[98.2 - 99.2]	98.7	[97.8 - 99.2]	0.08
**'EnGen0400_(ST86).fna'**	**'KUB3007_(ST729).fna'**	89.1	[85.7 - 91.7]	98.6	[97.9 - 99.0]	93.1	[90.8 - 94.9]	0.51
**'CL6870_(ST103).fasta'**	**'CL8682_(ST769).fasta'**	97.6	[96.1 - 98.5]	98.4	[97.6 - 98.9]	98.7	[97.9 - 99.2]	0.03
**'CL8682_(ST769).fasta'**	**'Merz96_(ST103).fna'**	92.7	[89.9 - 94.8]	98.3	[97.5 - 98.9]	95.6	[93.8 - 96.9]	0.3
**'15224_(ST16).fna'**	**'EF348_(ST16).fna'**	93.9	[91.4 - 95.8]	98.3	[97.5 - 98.8]	96.4	[94.8 - 97.5]	0.47
**'Praia.S.M2.C4_(ST62).fasta'**	*Enterococcus faecalis* NBRC 100480	96.0	[93.9 - 97.3]	98.2	[97.4 - 98.8]	97.7	[96.5 - 98.5]	0.15
**'CL11199_(ST778).fasta'**	**'Merz96_(ST103).fna'**	88.9	[85.5 - 91.6]	98.1	[97.2 - 98.7]	92.9	[90.6 - 94.7]	0.41
**'CL9797_(ST525).fasta'**	**'CL11199_(ST778).fasta'**	96.7	[94.9 - 97.9]	98.1	[97.3 - 98.7]	98.1	[97.1 - 98.8]	0.08
**'BIOML_A5_(ST875).fna'**	**'T11_(ST65).fna'**	93.6	[91.0 - 95.5]	97.9	[96.9 - 98.5]	96.1	[94.5 - 97.3]	0.33

Query	Subject	d_0	C.I. d_0	d_4	C.I. d_4	d_6	C.I. d_6	Diff. G+C Percent
**'209EA1_(ST624).fna'**	**'DSM111623_(ST624).fna'**	96.4	[94.5 - 97.7]	97.8	[96.9 - 98.5]	97.9	[96.8 - 98.7]	0.26
**'DSM111623_(ST624).fna'**	**'G81_(ST1468).fna'**	89.5	[86.1 - 92.1]	97.8	[96.9 - 98.5]	93.3	[91.0 - 95.0]	0.08
**'4928STDY7071435_(ST40).fna'**	**'D32_(ST40).fna'**	76.7	[72.7 - 80.2]	97.7	[96.7 - 98.3]	83.0	[79.7 - 85.9]	0.29
**'CL6870_(ST103).fasta'**	**'CL11199_(ST778).fasta'**	95.0	[92.8 - 96.6]	97.7	[96.8 - 98.4]	97.1	[95.7 - 98.0]	0.08
**'D32_(ST40).fna'**	**'E1_(ST40).fna'**	78.8	[74.9 - 82.3]	97.7	[96.7 - 98.4]	84.9	[81.7 - 87.6]	0.08
**'CL9797_(ST525).fasta'**	**'Merz96_(ST103).fna'**	89.2	[85.8 - 91.8]	97.7	[96.7 - 98.4]	93.0	[90.7 - 94.8]	0.49
**'CL9772_(ST525).fasta'**	**'Merz96_(ST103).fna'**	90.4	[87.2 - 92.9]	97.5	[96.5 - 98.2]	93.9	[91.7 - 95.5]	0.41
**'CL8682_(ST769).fasta'**	**'CL11199_(ST778).fasta'**	95.7	[93.6 - 97.1]	97.5	[96.4 - 98.2]	97.4	[96.2 - 98.3]	0.12
**'CL9772_(ST525).fasta'**	**'CL11199_(ST778).fasta'**	95.5	[93.3 - 97.0]	97.4	[96.3 - 98.1]	97.3	[96.0 - 98.2]	0.0
**'CL8682_(ST769).fasta'**	**'CL9797_(ST525).fasta'**	95.8	[93.7 - 97.2]	97.1	[96.0 - 97.9]	97.4	[96.2 - 98.3]	0.19
**'CL6870_(ST103).fasta'**	**'CL9797_(ST525).fasta'**	94.7	[92.3 - 96.4]	96.9	[95.7 - 97.8]	96.7	[95.2 - 97.8]	0.16
**'CL6870_(ST103).fasta'**	**'CL9772_(ST525).fasta'**	94.8	[92.4 - 96.4]	96.9	[95.8 - 97.8]	96.8	[95.3 - 97.8]	0.08
**'244_EFLS_(ST443).fna'**	**'F1_(ST72).fna'**	91.4	[88.3 - 93.7]	96.7	[95.5 - 97.6]	94.4	[92.4 - 96.0]	0.0
**'CL8682_(ST769).fasta'**	**'CL9772_(ST525).fasta'**	96.7	[94.9 - 97.9]	96.6	[95.3 - 97.5]	98.0	[96.9 - 98.7]	0.11
**'BT00.E.21_(ST23).fasta'**	*Enterococcus faecalis* NBRC 100480	87.2	[83.6 - 90.0]	96.4	[95.1 - 97.3]	91.3	[88.7 - 93.4]	0.42
**'CL9924_(ST6).fasta'**	**'T11_(ST65).fna'**	78.1	[74.1 - 81.6]	95.6	[94.1 - 96.7]	83.9	[80.6 - 86.7]	0.52
**'BT00.E.21_(ST23).fasta'**	**'Praia.S.M2.C4_(ST62).fasta'**	83.0	[79.2 - 86.3]	95.2	[93.6 - 96.4]	87.9	[84.9 - 90.4]	0.26
**'CL9943_(ST4).fasta'**	**'P9 CL A7_(ST4).fna'**	78.9	[75.0 - 82.4]	95.1	[93.6 - 96.3]	84.5	[81.3 - 87.3]	0.06
**'BIOML_A5_(ST875).fna'**	**'CL9924_(ST6).fasta'**	76.4	[72.5 - 80.0]	95.0	[93.4 - 96.2]	82.4	[79.0 - 85.3]	0.19
**'14EA1_(ST147).fna'**	**'19910_(ST116).fna'**	83.1	[79.3 - 86.4]	94.8	[93.1 - 96.0]	87.9	[85.0 - 90.4]	0.48
**'CL9172_(ST21).fasta'**	**'E1_(ST40).fna'**	90.2	[87.0 - 92.7]	94.4	[92.6 - 95.7]	93.3	[91.0 - 95.0]	0.08
**'4928STDY7071351_(ST30).fna'**	**'CL9314_(ST21).fasta'**	87.1	[83.5 - 90.0]	94.4	[92.6 - 95.7]	91.0	[88.3 - 93.0]	0.24
**'4928STDY7071351_(ST30).fna'**	**'CL9172_(ST21).fasta'**	88.2	[84.7 - 91.0]	94.3	[92.5 - 95.6]	91.8	[89.3 - 93.8]	0.25
**'P9 CL A7_(ST4).fna'**	*Arthrobacter sulfonylureivorans* LAM7117	12.5	[9.8 - 15.7]	94.3	[92.6 - 95.7]	12.9	[10.6 - 15.6]	28.93
**'CL9314_(ST21).fasta'**	**'E1_(ST40).fna'**	89.6	[86.3 - 92.2]	94.3	[92.5 - 95.6]	92.9	[90.5 - 94.7]	0.07
**'244_EFLS_(ST443).fna'**	**'BT00.E.21_(ST23).fasta'**	80.4	[76.5 - 83.8]	94.3	[92.5 - 95.6]	85.6	[82.5 - 88.3]	0.39
**'4928STDY7071435_(ST40).fna'**	**'CL9172_(ST21).fasta'**	88.2	[84.7 - 90.9]	94.2	[92.5 - 95.6]	91.8	[89.3 - 93.7]	0.29

Query	Subject	d_0	C.I. d_0	d_4	C.I. d_4	d_6	C.I. d_6	Diff. G+C Percent
**'244_EFLS_(ST443).fna'**	**'F_(ST712).fna'**	82.6	[78.7 - 85.8]	94.1	[92.4 - 95.5]	87.4	[84.3 - 89.9]	0.1
**'4928STDY7071435_(ST40).fna'**	**'CL9314_(ST21).fasta'**	87.7	[84.1 - 90.5]	94.1	[92.4 - 95.5]	91.4	[88.8 - 93.4]	0.3
**'14EA1_(ST147).fna'**	**'732_EFLS_(ST16).fna'**	84.9	[81.2 - 88.0]	94.0	[92.2 - 95.4]	89.2	[86.4 - 91.5]	0.4
**'E1_(ST40).fna'**	**'LB00.E.122_(ST21).fasta'**	87.9	[84.4 - 90.7]	94.0	[92.2 - 95.4]	91.6	[89.0 - 93.6]	0.01
**'732_EFLS_(ST16).fna'**	**'CVM_N60027F_(ST862).fna'**	90.3	[87.1 - 92.8]	94.0	[92.2 - 95.4]	93.3	[91.0 - 95.0]	0.19
**'4928STDY7071435_(ST40).fna'**	**'LB00.E.122_(ST21).fasta'**	86.2	[82.6 - 89.2]	93.9	[92.1 - 95.3]	90.2	[87.5 - 92.4]	0.38
**'F_(ST712).fna'**	**'F1_(ST72).fna'**	85.6	[81.9 - 88.6]	93.8	[92.0 - 95.2]	89.7	[86.9 - 92.0]	0.1
**'CVM_N60027F_(ST862).fna'**	**'EF348_(ST16).fna'**	85.9	[82.2 - 88.9]	93.8	[92.0 - 95.2]	90.0	[87.2 - 92.2]	0.02
**'4928STDY7071351_(ST30).fna'**	**'LB00.E.122_(ST21).fasta'**	87.1	[83.5 - 90.0]	93.7	[91.8 - 95.1]	90.9	[88.2 - 93.0]	0.16
**'CL9172_(ST21).fasta'**	**'D32_(ST40).fna'**	81.6	[77.7 - 85.0]	93.5	[91.6 - 95.0]	86.5	[83.4 - 89.1]	0.0
**'732_EFLS_(ST16).fna'**	**'P9 CL A7_(ST4).fna'**	84.8	[81.1 - 87.9]	93.5	[91.6 - 94.9]	89.1	[86.2 - 91.4]	0.32
**'732_EFLS_(ST16).fna'**	**'EnGen0400_(ST86).fna'**	85.8	[82.1 - 88.8]	93.5	[91.6 - 95.0]	89.8	[87.1 - 92.1]	0.32
**'732_EFLS_(ST16).fna'**	**'EN242_(ST82).fna'**	81.6	[77.7 - 84.9]	93.4	[91.6 - 94.9]	86.4	[83.3 - 89.0]	0.31
**'244_EFLS_(ST443).fna'**	*Enterococcus faecalis* NBRC 100480	88.2	[84.8 - 91.0]	93.4	[91.6 - 94.9]	91.7	[89.2 - 93.7]	0.03
**'732_EFLS_(ST16).fna'**	**'CL9172_(ST21).fasta'**	88.8	[85.4 - 91.5]	93.4	[91.5 - 94.9]	92.1	[89.6 - 94.0]	0.07
**'732_EFLS_(ST16).fna'**	**'LB00.E.122_(ST21).fasta'**	86.1	[82.4 - 89.1]	93.4	[91.5 - 94.9]	90.1	[87.3 - 92.3]	0.02
**'732_EFLS_(ST16).fna'**	**'CL9314_(ST21).fasta'**	88.3	[84.8 - 91.0]	93.4	[91.5 - 94.8]	91.7	[89.2 - 93.7]	0.06
**'CL9314_(ST21).fasta'**	**'EnGen0400_(ST86).fna'**	82.5	[78.6 - 85.8]	93.4	[91.5 - 94.9]	87.2	[84.1 - 89.7]	0.26
**'244_EFLS_(ST443).fna'**	**'Praia.S.M2.C4_(ST62).fasta'**	84.5	[80.7 - 87.6]	93.4	[91.6 - 94.9]	88.8	[85.9 - 91.2]	0.13
**'14EA1_(ST147).fna'**	**'EF348_(ST16).fna'**	82.4	[78.5 - 85.7]	93.4	[91.6 - 94.9]	87.1	[84.1 - 89.7]	0.23
**'CVM_N60027F_(ST862).fna'**	**'P9 CL A7_(ST4).fna'**	84.5	[80.7 - 87.6]	93.3	[91.4 - 94.8]	88.8	[85.9 - 91.1]	0.51
**'732_EFLS_(ST16).fna'**	**'E1_(ST40).fna'**	85.3	[81.6 - 88.4]	93.3	[91.4 - 94.8]	89.4	[86.6 - 91.7]	0.01
**'14EA1_(ST147).fna'**	**'CVM_N60027F_(ST862).fna'**	86.4	[82.8 - 89.4]	93.3	[91.4 - 94.8]	90.3	[87.6 - 92.5]	0.21
**'732_EFLS_(ST16).fna'**	**'EN788_(ST82).fna'**	81.3	[77.4 - 84.7]	93.3	[91.4 - 94.8]	86.2	[83.1 - 88.8]	0.44
**'CVM_N60027F_(ST862).fna'**	**'E1_(ST40).fna'**	80.6	[76.7 - 84.0]	93.3	[91.4 - 94.8]	85.6	[82.5 - 88.3]	0.2
**'4928STDY7071435_(ST40).fna'**	**'BT00.E.21_(ST23).fasta'**	80.7	[76.7 - 84.1]	93.3	[91.4 - 94.8]	85.7	[82.5 - 88.3]	0.62
**'15224_(ST16).fna'**	**'CVM_N60027F_(ST862).fna'**	81.5	[77.6 - 84.8]	93.3	[91.4 - 94.8]	86.4	[83.3 - 89.0]	0.48

Query	Subject	d_0	C.I. d_0	d_4	C.I. d_4	d_6	C.I. d_6	Diff. G+C Percent
**'CL9797_(ST525).fasta'**	**'CL9924_(ST6).fasta'**	89.7	[86.4 - 92.2]	93.3	[91.4 - 94.8]	92.8	[90.4 - 94.6]	0.03
**'732_EFLS_(ST16).fna'**	**'CL9943_(ST4).fasta'**	80.6	[76.6 - 84.0]	93.3	[91.4 - 94.8]	85.6	[82.4 - 88.3]	0.38
**'CVM_N60027F_(ST862).fna'**	**'EnGen0400_(ST86).fna'**	89.0	[85.6 - 91.7]	93.3	[91.4 - 94.8]	92.3	[89.8 - 94.2]	0.13
**'CL9772_(ST525).fasta'**	**'CL9924_(ST6).fasta'**	88.7	[85.3 - 91.4]	93.3	[91.4 - 94.8]	92.1	[89.6 - 94.0]	0.05
**'732_EFLS_(ST16).fna'**	**'19910_(ST116).fna'**	81.6	[77.7 - 85.0]	93.3	[91.5 - 94.8]	86.5	[83.4 - 89.1]	0.08
**'E1_(ST40).fna'**	**'EnGen0400_(ST86).fna'**	84.2	[80.4 - 87.4]	93.2	[91.3 - 94.7]	88.5	[85.6 - 90.9]	0.33
**'CL9314_(ST21).fasta'**	**'D32_(ST40).fna'**	81.4	[77.5 - 84.7]	93.2	[91.3 - 94.7]	86.3	[83.1 - 88.9]	0.01
**'14EA1_(ST147).fna'**	**'CL9172_(ST21).fasta'**	82.2	[78.3 - 85.5]	93.2	[91.2 - 94.7]	86.9	[83.8 - 89.4]	0.33
**'CL9824_(ST631).fasta'**	**'T11_(ST65).fna'**	94.1	[91.6 - 95.9]	93.2	[91.3 - 94.7]	95.9	[94.2 - 97.2]	0.05
**'BT00.E.21_(ST23).fasta'**	**'E1_(ST40).fna'**	82.5	[78.7 - 85.8]	93.2	[91.3 - 94.7]	87.2	[84.2 - 89.7]	0.25
**'14EA1_(ST147).fna'**	**'CL9314_(ST21).fasta'**	81.6	[77.7 - 84.9]	93.2	[91.3 - 94.7]	86.4	[83.3 - 89.0]	0.34
**'20_SD_W_06_(ST368).fna'**	**'EN242_(ST82).fna'**	72.5	[68.6 - 76.2]	93.2	[91.3 - 94.7]	78.6	[75.2 - 81.7]	0.3
**'EF348_(ST16).fna'**	**'EnGen0400_(ST86).fna'**	80.5	[76.6 - 83.9]	93.1	[91.2 - 94.6]	85.5	[82.4 - 88.2]	0.15
**'EnGen0400_(ST86).fna'**	**'P9 CL A7_(ST4).fna'**	80.2	[76.3 - 83.6]	93.1	[91.2 - 94.7]	85.3	[82.1 - 88.0]	0.64
**'EnGen0400_(ST86).fna'**	**'LB00.E.122_(ST21).fasta'**	80.8	[76.9 - 84.2]	93.1	[91.2 - 94.6]	85.7	[82.6 - 88.4]	0.34
**'CL9172_(ST21).fasta'**	**'EnGen0400_(ST86).fna'**	83.6	[79.8 - 86.8]	93.1	[91.2 - 94.7]	88.1	[85.1 - 90.5]	0.25
**'732_EFLS_(ST16).fna'**	**'4928STDY7071435_(ST40).fna'**	83.6	[79.7 - 86.8]	93.1	[91.2 - 94.7]	88.0	[85.1 - 90.5]	0.35
**'4928STDY7071435_(ST40).fna'**	**'CVM_N60027F_(ST862).fna'**	78.4	[74.5 - 81.9]	93.1	[91.2 - 94.6]	83.8	[80.5 - 86.6]	0.17
**'CVM_N60027F_(ST862).fna'**	**'D32_(ST40).fna'**	79.7	[75.7 - 83.1]	93.0	[91.1 - 94.6]	84.8	[81.6 - 87.5]	0.13
**'CL9943_(ST4).fasta'**	**'CVM_N60027F_(ST862).fna'**	77.1	[73.1 - 80.7]	93.0	[91.1 - 94.5]	82.6	[79.3 - 85.5]	0.57
**'302EA1_(ST122).fna'**	**'CVM_N60027F_(ST862).fna'**	84.3	[80.6 - 87.5]	93.0	[91.1 - 94.5]	88.6	[85.7 - 91.0]	0.08
**'732_EFLS_(ST16).fna'**	**'KUB3007_(ST729).fna'**	79.9	[76.0 - 83.3]	93.0	[91.1 - 94.5]	85.0	[81.8 - 87.7]	0.19
**'732_EFLS_(ST16).fna'**	**'D32_(ST40).fna'**	77.4	[73.4 - 81.0]	93.0	[91.0 - 94.5]	82.9	[79.6 - 85.7]	0.06
**'20_SD_W_06_(ST368).fna'**	**'EN788_(ST82).fna'**	72.0	[68.1 - 75.7]	93.0	[91.0 - 94.5]	78.1	[74.6 - 81.2]	0.42
**'E1_(ST40).fna'**	**'EF348_(ST16).fna'**	80.4	[76.5 - 83.8]	92.9	[91.0 - 94.5]	85.4	[82.2 - 88.1]	0.18
**'CL9172_(ST21).fasta'**	**'EF348_(ST16).fna'**	84.4	[80.7 - 87.6]	92.9	[90.9 - 94.4]	88.7	[85.8 - 91.0]	0.1
**'4928STDY7071351_(ST30).fna'**	**'E1_(ST40).fna'**	82.8	[79.0 - 86.1]	92.9	[91.0 - 94.5]	87.4	[84.4 - 89.9]	0.17

Query	Subject	d_0	C.I. d_0	d_4	C.I. d_4	d_6	C.I. d_6	Diff. G+C Percent
**'BIOML_A5_(ST875).fna'**	**'EN242_(ST82).fna'**	75.5	[71.5 - 79.1]	92.9	[91.0 - 94.5]	81.2	[77.8 - 84.1]	0.28
**'BIOML_A5_(ST875).fna'**	**'CL9824_(ST631).fasta'**	88.6	[85.1 - 91.3]	92.9	[91.0 - 94.5]	91.9	[89.4 - 93.8]	0.38
**'CL9797_(ST525).fasta'**	**'T11_(ST65).fna'**	76.2	[72.3 - 79.8]	92.9	[91.0 - 94.5]	81.9	[78.5 - 84.8]	0.54
**'302EA1_(ST122).fna'**	**'732_EFLS_(ST16).fna'**	82.1	[78.3 - 85.4]	92.9	[90.9 - 94.5]	86.8	[83.8 - 89.4]	0.1
**'244_EFLS_(ST443).fna'**	**'EnGen0400_(ST86).fna'**	81.5	[77.6 - 84.8]	92.9	[90.9 - 94.4]	86.3	[83.2 - 88.9]	0.19
**'CL9824_(ST631).fasta'**	**'CL9924_(ST6).fasta'**	75.0	[71.0 - 78.6]	92.9	[90.9 - 94.4]	80.8	[77.4 - 83.7]	0.57
**'4928STDY7071435_(ST40).fna'**	**'EF348_(ST16).fna'**	78.8	[74.9 - 82.3]	92.9	[91.0 - 94.5]	84.1	[80.8 - 86.9]	0.18
**'4928STDY7071435_(ST40).fna'**	**'EnGen0400_(ST86).fna'**	82.7	[78.8 - 86.0]	92.9	[91.0 - 94.5]	87.3	[84.3 - 89.8]	0.04
**'732_EFLS_(ST16).fna'**	**'4928STDY7071351_(ST30).fna'**	84.4	[80.6 - 87.5]	92.9	[91.0 - 94.5]	88.6	[85.7 - 91.0]	0.18
**'CL9772_(ST525).fasta'**	**'T11_(ST65).fna'**	78.7	[74.8 - 82.2]	92.9	[91.0 - 94.5]	84.0	[80.7 - 86.8]	0.46
**'14EA1_(ST147).fna'**	**'KUB3007_(ST729).fna'**	76.5	[72.5 - 80.1]	92.9	[91.0 - 94.5]	82.1	[78.8 - 85.0]	0.59
**'CL9172_(ST21).fasta'**	**'P9 CL A7_(ST4).fna'**	79.7	[75.8 - 83.2]	92.8	[90.8 - 94.4]	84.8	[81.6 - 87.5]	0.39
**'F1_(ST72).fna'**	*Enterococcus faecalis* NBRC 100480	85.6	[81.9 - 88.7]	92.8	[90.9 - 94.4]	89.6	[86.8 - 91.9]	0.02
**'CVM_N60027F_(ST862).fna'**	**'EN788_(ST82).fna'**	80.1	[76.1 - 83.5]	92.8	[90.8 - 94.4]	85.1	[81.9 - 87.8]	0.62
**'CVM_N60027F_(ST862).fna'**	**'LB00.E.122_(ST21).fasta'**	82.6	[78.7 - 85.8]	92.8	[90.8 - 94.4]	87.2	[84.1 - 89.7]	0.21
**'732_EFLS_(ST16).fna'**	**'BT00.E.21_(ST23).fasta'**	83.5	[79.6 - 86.7]	92.8	[90.8 - 94.4]	87.9	[84.9 - 90.3]	0.26
**'EF348_(ST16).fna'**	**'P9 CL A7_(ST4).fna'**	86.0	[82.3 - 89.0]	92.8	[90.9 - 94.4]	89.9	[87.1 - 92.1]	0.49
**'F1_(ST72).fna'**	**'Praia.S.M2.C4_(ST62).fasta'**	80.6	[76.6 - 84.0]	92.8	[90.8 - 94.4]	85.5	[82.3 - 88.2]	0.13
**'EN788_(ST82).fna'**	**'T11_(ST65).fna'**	80.2	[76.3 - 83.6]	92.8	[90.8 - 94.3]	85.2	[82.0 - 87.9]	0.73
**'EN242_(ST82).fna'**	**'T11_(ST65).fna'**	81.0	[77.1 - 84.4]	92.8	[90.8 - 94.4]	85.9	[82.8 - 88.6]	0.61
**'CL9314_(ST21).fasta'**	**'KUB3007_(ST729).fna'**	78.5	[74.6 - 82.0]	92.8	[90.8 - 94.3]	83.8	[80.5 - 86.6]	0.25
**'CL11199_(ST778).fasta'**	**'T11_(ST65).fna'**	75.7	[71.7 - 79.3]	92.8	[90.9 - 94.4]	81.3	[78.0 - 84.3]	0.47
**'EF349_(ST631).fna'**	**'T11_(ST65).fna'**	88.8	[85.4 - 91.4]	92.8	[90.9 - 94.4]	92.0	[89.5 - 93.9]	0.97
**'4928STDY7071351_(ST30).fna'**	**'4928STDY7071435_(ST40).fna'**	80.9	[77.0 - 84.3]	92.8	[90.8 - 94.4]	85.8	[82.7 - 88.5]	0.54
**'732_EFLS_(ST16).fna'**	**'T11_(ST65).fna'**	86.9	[83.3 - 89.8]	92.8	[90.8 - 94.3]	90.6	[87.9 - 92.7]	0.29
**'19910_(ST116).fna'**	**'CVM_N60027F_(ST862).fna'**	87.0	[83.4 - 89.9]	92.8	[90.9 - 94.4]	90.7	[88.0 - 92.8]	0.27
**'244_EFLS_(ST443).fna'**	**'EN242_(ST82).fna'**	72.0	[68.0 - 75.6]	92.7	[90.7 - 94.3]	78.0	[74.6 - 81.1]	0.44

Query	Subject	d_0	C.I. d_0	d_4	C.I. d_4	d_6	C.I. d_6	Diff. G+C Percent
**'244_EFLS_(ST443).fna'**	**'CL9172_(ST21).fasta'**	84.5	[80.7 - 87.6]	92.7	[90.7 - 94.3]	88.7	[85.8 - 91.1]	0.06
**'CL9172_(ST21).fasta'**	**'CVM_N60027F_(ST862).fna'**	85.3	[81.6 - 88.4]	92.7	[90.7 - 94.3]	89.3	[86.5 - 91.6]	0.12
**'244_EFLS_(ST443).fna'**	**'EN788_(ST82).fna'**	73.1	[69.2 - 76.8]	92.7	[90.7 - 94.3]	79.1	[75.6 - 82.1]	0.56
**'CL9314_(ST21).fasta'**	**'CVM_N60027F_(ST862).fna'**	84.7	[80.9 - 87.8]	92.7	[90.7 - 94.3]	88.8	[86.0 - 91.2]	0.13
**'LB00.E.122_(ST21).fasta'**	**'P9 CL A7_(ST4).fna'**	77.2	[73.2 - 80.8]	92.7	[90.7 - 94.3]	82.7	[79.3 - 85.5]	0.3
**'BT00.E.21_(ST23).fasta'**	**'CVM_N60027F_(ST862).fna'**	79.7	[75.7 - 83.1]	92.7	[90.7 - 94.3]	84.7	[81.5 - 87.5]	0.45
**'E1_(ST40).fna'**	**'EN242_(ST82).fna'**	73.7	[69.7 - 77.3]	92.7	[90.7 - 94.2]	79.6	[76.2 - 82.6]	0.3
**'BIOML_A5_(ST875).fna'**	**'EN788_(ST82).fna'**	76.9	[72.9 - 80.5]	92.7	[90.7 - 94.3]	82.4	[79.1 - 85.3]	0.4
**'CL9924_(ST6).fasta'**	**'CL11199_(ST778).fasta'**	87.3	[83.7 - 90.1]	92.7	[90.7 - 94.3]	90.9	[88.2 - 93.0]	0.05
**'BT00.E.21_(ST23).fasta'**	**'CL9172_(ST21).fasta'**	84.3	[80.5 - 87.5]	92.7	[90.8 - 94.3]	88.6	[85.6 - 90.9]	0.33
**'14EA1_(ST147).fna'**	**'EN242_(ST82).fna'**	73.5	[69.5 - 77.1]	92.7	[90.7 - 94.3]	79.4	[76.0 - 82.4]	0.71
**'EN242_(ST82).fna'**	**'LB00.E.122_(ST21).fasta'**	73.8	[69.8 - 77.4]	92.7	[90.7 - 94.2]	79.7	[76.2 - 82.7]	0.29
**'CL9314_(ST21).fasta'**	**'EF348_(ST16).fna'**	84.3	[80.6 - 87.5]	92.7	[90.7 - 94.3]	88.6	[85.7 - 91.0]	0.11
**'244_EFLS_(ST443).fna'**	**'732_EFLS_(ST16).fna'**	84.0	[80.2 - 87.2]	92.7	[90.7 - 94.3]	88.3	[85.4 - 90.7]	0.13
**'CVM_N60027F_(ST862).fna'**	**'EN242_(ST82).fna'**	80.9	[77.0 - 84.3]	92.7	[90.7 - 94.3]	85.8	[82.7 - 88.5]	0.5
**'19910_(ST116).fna'**	**'EF348_(ST16).fna'**	78.7	[74.7 - 82.2]	92.7	[90.7 - 94.3]	83.9	[80.6 - 86.7]	0.25
**'14EA1_(ST147).fna'**	**'EnGen0400_(ST86).fna'**	84.9	[81.2 - 88.0]	92.6	[90.6 - 94.2]	89.0	[86.1 - 91.3]	0.08
**'BT00.E.21_(ST23).fasta'**	**'EnGen0400_(ST86).fna'**	77.7	[73.7 - 81.2]	92.6	[90.6 - 94.2]	83.0	[79.7 - 85.9]	0.58
**'19910_(ST116).fna'**	**'E1_(ST40).fna'**	75.5	[71.5 - 79.1]	92.6	[90.6 - 94.2]	81.1	[77.8 - 84.1]	0.06
**'CL9172_(ST21).fasta'**	**'CL9943_(ST4).fasta'**	78.9	[74.9 - 82.4]	92.6	[90.6 - 94.2]	84.1	[80.8 - 86.9]	0.45
**'244_EFLS_(ST443).fna'**	**'E1_(ST40).fna'**	78.1	[74.1 - 81.6]	92.6	[90.6 - 94.2]	83.4	[80.1 - 86.2]	0.14
**'19910_(ST116).fna'**	**'CL9314_(ST21).fasta'**	80.7	[76.7 - 84.1]	92.6	[90.6 - 94.2]	85.6	[82.4 - 88.2]	0.13
**'CL9172_(ST21).fasta'**	**'EN242_(ST82).fna'**	76.3	[72.3 - 79.8]	92.6	[90.6 - 94.2]	81.8	[78.5 - 84.7]	0.38
**'19910_(ST116).fna'**	**'CL9172_(ST21).fasta'**	80.3	[76.3 - 83.7]	92.6	[90.6 - 94.2]	85.2	[82.1 - 87.9]	0.14
**'20_SD_W_06_(ST368).fna'**	**'732_EFLS_(ST16).fna'**	81.7	[77.8 - 85.0]	92.6	[90.7 - 94.2]	86.4	[83.3 - 89.0]	0.01
**'732_EFLS_(ST16).fna'**	**'Praia.S.M2.C4_(ST62).fasta'**	81.8	[77.9 - 85.1]	92.6	[90.6 - 94.2]	86.5	[83.4 - 89.1]	0.0
**'302EA1_(ST122).fna'**	**'EnGen0400_(ST86).fna'**	82.1	[78.3 - 85.5]	92.6	[90.6 - 94.2]	86.8	[83.7 - 89.3]	0.22

Query	Subject	d_0	C.I. d_0	d_4	C.I. d_4	d_6	C.I. d_6	Diff. G+C Percent
**'14EA1_(ST147).fna'**	**'EN788_(ST82).fna'**	73.7	[69.8 - 77.4]	92.6	[90.6 - 94.2]	79.6	[76.2 - 82.6]	0.84
**'EF348_(ST16).fna'**	**'EN242_(ST82).fna'**	77.5	[73.6 - 81.1]	92.6	[90.6 - 94.2]	82.9	[79.6 - 85.8]	0.48
**'19910_(ST116).fna'**	**'LB00.E.122_(ST21).fasta'**	77.8	[73.8 - 81.3]	92.6	[90.6 - 94.2]	83.2	[79.9 - 86.0]	0.05
**'D32_(ST40).fna'**	**'EnGen0400_(ST86).fna'**	76.7	[72.7 - 80.3]	92.6	[90.6 - 94.2]	82.2	[78.9 - 85.1]	0.26
**'CL9314_(ST21).fasta'**	**'EN242_(ST82).fna'**	75.7	[71.7 - 79.3]	92.6	[90.6 - 94.2]	81.3	[77.9 - 84.2]	0.37
**'BT00.E.21_(ST23).fasta'**	**'CL9314_(ST21).fasta'**	83.5	[79.6 - 86.7]	92.6	[90.6 - 94.2]	87.9	[84.9 - 90.3]	0.32
**'CL9172_(ST21).fasta'**	**'KUB3007_(ST729).fna'**	77.9	[74.0 - 81.5]	92.6	[90.6 - 94.2]	83.3	[80.0 - 86.1]	0.26
**'CL9314_(ST21).fasta'**	**'P9 CL A7_(ST4).fna'**	79.5	[75.6 - 83.0]	92.6	[90.6 - 94.2]	84.6	[81.4 - 87.4]	0.38
**'244_EFLS_(ST443).fna'**	**'CL9314_(ST21).fasta'**	83.4	[79.6 - 86.6]	92.6	[90.6 - 94.2]	87.8	[84.8 - 90.3]	0.07
**'15224_(ST16).fna'**	**'CL9172_(ST21).fasta'**	78.5	[74.5 - 82.0]	92.6	[90.6 - 94.2]	83.7	[80.4 - 86.5]	0.36
**'BT00.E.21_(ST23).fasta'**	**'D32_(ST40).fna'**	77.4	[73.4 - 80.9]	92.5	[90.5 - 94.1]	82.8	[79.5 - 85.7]	0.33
**'BT00.E.21_(ST23).fasta'**	**'F1_(ST72).fna'**	78.8	[74.8 - 82.2]	92.5	[90.5 - 94.1]	83.9	[80.7 - 86.7]	0.39
**'20_SD_W_06_(ST368).fna'**	**'T11_(ST65).fna'**	87.7	[84.2 - 90.6]	92.5	[90.5 - 94.1]	91.2	[88.6 - 93.3]	0.31
**'EF348_(ST16).fna'**	**'KUB3007_(ST729).fna'**	76.0	[72.0 - 79.6]	92.5	[90.5 - 94.1]	81.6	[78.2 - 84.5]	0.36
**'CL9172_(ST21).fasta'**	**'EN788_(ST82).fna'**	76.2	[72.3 - 79.8]	92.5	[90.5 - 94.1]	81.8	[78.4 - 84.7]	0.5
**'19910_(ST116).fna'**	**'EN242_(ST82).fna'**	75.5	[71.6 - 79.1]	92.5	[90.5 - 94.1]	81.2	[77.8 - 84.1]	0.24
**'D32_(ST40).fna'**	**'LB00.E.122_(ST21).fasta'**	80.5	[76.5 - 83.9]	92.5	[90.4 - 94.1]	85.4	[82.2 - 88.1]	0.09
**'EnGen0400_(ST86).fna'**	*Enterococcus faecalis* NBRC 100480	79.4	[75.4 - 82.8]	92.5	[90.5 - 94.1]	84.5	[81.2 - 87.2]	0.16
**'302EA1_(ST122).fna'**	**'E1_(ST40).fna'**	77.5	[73.5 - 81.0]	92.5	[90.5 - 94.1]	82.9	[79.6 - 85.7]	0.12
**'E1_(ST40).fna'**	**'EN788_(ST82).fna'**	73.6	[69.6 - 77.2]	92.5	[90.5 - 94.1]	79.4	[76.0 - 82.5]	0.42
**'302EA1_(ST122).fna'**	**'4928STDY7071435_(ST40).fna'**	75.7	[71.8 - 79.3]	92.4	[90.4 - 94.0]	81.3	[78.0 - 84.3]	0.25
**'19910_(ST116).fna'**	**'P9 CL A7_(ST4).fna'**	78.5	[74.5 - 82.0]	92.4	[90.3 - 94.0]	83.7	[80.4 - 86.5]	0.25
**'CVM_N60027F_(ST862).fna'**	**'KUB3007_(ST729).fna'**	84.3	[80.5 - 87.5]	92.4	[90.4 - 94.1]	88.5	[85.6 - 90.9]	0.38
**'CL9943_(ST4).fasta'**	**'EnGen0400_(ST86).fna'**	76.5	[72.5 - 80.1]	92.4	[90.3 - 94.0]	82.0	[78.7 - 84.9]	0.7
**'D32_(ST40).fna'**	**'Praia.S.M2.C4_(ST62).fasta'**	76.7	[72.7 - 80.2]	92.4	[90.4 - 94.0]	82.2	[78.8 - 85.1]	0.06
**'EF348_(ST16).fna'**	**'EN788_(ST82).fna'**	78.8	[74.8 - 82.3]	92.4	[90.3 - 94.0]	83.9	[80.7 - 86.7]	0.61
**'CL9314_(ST21).fasta'**	**'EN788_(ST82).fna'**	75.8	[71.9 - 79.4]	92.4	[90.3 - 94.0]	81.4	[78.0 - 84.4]	0.49

Query	Subject	d_0	C.I. d_0	d_4	C.I. d_4	d_6	C.I. d_6	Diff. G+C Percent
**'20_SD_W_06_(ST368).fna'**	**'CVM_N60027F_(ST862).fna'**	85.6	[81.9 - 88.6]	92.4	[90.4 - 94.1]	89.5	[86.7 - 91.8]	0.2
**'CL9314_(ST21).fasta'**	**'CL9943_(ST4).fasta'**	78.3	[74.3 - 81.8]	92.4	[90.4 - 94.1]	83.5	[80.3 - 86.4]	0.44
**'244_EFLS_(ST443).fna'**	**'CVM_N60027F_(ST862).fna'**	85.1	[81.4 - 88.2]	92.4	[90.4 - 94.0]	89.2	[86.3 - 91.5]	0.06
**'EN788_(ST82).fna'**	**'EnGen0400_(ST86).fna'**	76.2	[72.2 - 79.8]	92.4	[90.4 - 94.1]	81.7	[78.4 - 84.7]	0.76
**'D32_(ST40).fna'**	**'EN788_(ST82).fna'**	72.1	[68.1 - 75.7]	92.4	[90.4 - 94.1]	78.1	[74.6 - 81.2]	0.5
**'4928STDY7071435_(ST40).fna'**	**'KUB3007_(ST729).fna'**	71.5	[67.6 - 75.2]	92.4	[90.3 - 94.0]	77.6	[74.1 - 80.7]	0.55
**'EN242_(ST82).fna'**	**'P9 CL A7_(ST4).fna'**	78.6	[74.6 - 82.1]	92.4	[90.4 - 94.0]	83.8	[80.5 - 86.6]	0.01
**'4928STDY7071435_(ST40).fna'**	**'19910_(ST116).fna'**	73.6	[69.6 - 77.2]	92.4	[90.4 - 94.0]	79.4	[76.0 - 82.5]	0.43
**'KUB3007_(ST729).fna'**	**'P9 CL A7_(ST4).fna'**	74.0	[70.0 - 77.6]	92.4	[90.3 - 94.0]	79.8	[76.4 - 82.8]	0.13
**'14EA1_(ST147).fna'**	**'15224_(ST16).fna'**	76.2	[72.3 - 79.8]	92.3	[90.3 - 93.9]	81.8	[78.4 - 84.7]	0.7
**'CL9772_(ST525).fasta'**	**'CL9824_(ST631).fasta'**	77.9	[73.9 - 81.4]	92.3	[90.3 - 94.0]	83.2	[79.9 - 86.0]	0.51
**'19910_(ST116).fna'**	**'D32_(ST40).fna'**	73.7	[69.7 - 77.3]	92.3	[90.3 - 93.9]	79.5	[76.1 - 82.6]	0.14
**'244_EFLS_(ST443).fna'**	**'4928STDY7071435_(ST40).fna'**	76.6	[72.6 - 80.2]	92.3	[90.2 - 93.9]	82.1	[78.7 - 85.0]	0.23
**'244_EFLS_(ST443).fna'**	**'302EA1_(ST122).fna'**	83.5	[79.7 - 86.7]	92.3	[90.2 - 93.9]	87.8	[84.9 - 90.3]	0.02
**'732_EFLS_(ST16).fna'**	*Enterococcus faecalis* NBRC 100480	84.3	[80.5 - 87.4]	92.3	[90.3 - 94.0]	88.5	[85.6 - 90.9]	0.16
**'CL9924_(ST6).fasta'**	**'Merz96_(ST103).fna'**	79.3	[75.3 - 82.7]	92.3	[90.3 - 94.0]	84.3	[81.1 - 87.1]	0.46
**'BIOML_A5_(ST875).fna'**	**'EF349_(ST631).fna'**	83.7	[79.9 - 86.9]	92.3	[90.3 - 93.9]	88.0	[85.0 - 90.4]	1.3
**'EF348_(ST16).fna'**	**'T11_(ST65).fna'**	82.5	[78.6 - 85.8]	92.3	[90.3 - 94.0]	87.0	[84.0 - 89.5]	0.12
**'KUB3007_(ST729).fna'**	**'LB00.E.122_(ST21).fasta'**	75.0	[71.0 - 78.6]	92.3	[90.3 - 93.9]	80.6	[77.3 - 83.6]	0.17
**'20_SD_W_06_(ST368).fna'**	**'EnGen0400_(ST86).fna'**	83.1	[79.3 - 86.3]	92.3	[90.2 - 93.9]	87.5	[84.5 - 90.0]	0.33
**'BT00.E.21_(ST23).fasta'**	**'EF348_(ST16).fna'**	79.7	[75.8 - 83.2]	92.3	[90.2 - 93.9]	84.7	[81.5 - 87.5]	0.43
**'14EA1_(ST147).fna'**	**'Praia.S.M2.C4_(ST62).fasta'**	80.0	[76.1 - 83.5]	92.3	[90.3 - 94.0]	85.0	[81.8 - 87.7]	0.4
**'15224_(ST16).fna'**	**'CL9314_(ST21).fasta'**	79.8	[75.9 - 83.3]	92.3	[90.2 - 93.9]	84.8	[81.6 - 87.5]	0.35
**'302EA1_(ST122).fna'**	**'EF348_(ST16).fna'**	78.2	[74.3 - 81.7]	92.3	[90.3 - 94.0]	83.5	[80.2 - 86.3]	0.07
**'E1_(ST40).fna'**	**'KUB3007_(ST729).fna'**	73.7	[69.7 - 77.4]	92.3	[90.2 - 93.9]	79.5	[76.1 - 82.6]	0.18
**'BT00.E.21_(ST23).fasta'**	**'T11_(ST65).fna'**	79.2	[75.2 - 82.7]	92.3	[90.2 - 93.9]	84.3	[81.0 - 87.1]	0.56
**'E1_(ST40).fna'**	*Enterococcus faecalis* NBRC 100480	78.4	[74.4 - 81.9]	92.3	[90.3 - 94.0]	83.6	[80.3 - 86.4]	0.17

Query	Subject	d_0	C.I. d_0	d_4	C.I. d_4	d_6	C.I. d_6	Diff. G+C Percent
**'14EA1_(ST147).fna'**	**'BT00.E.21_(ST23).fasta'**	78.5	[74.5 - 82.0]	92.3	[90.3 - 93.9]	83.7	[80.4 - 86.5]	0.66
**'19910_(ST116).fna'**	**'EN788_(ST82).fna'**	75.3	[71.3 - 78.9]	92.3	[90.2 - 93.9]	80.9	[77.5 - 83.9]	0.36
**'14EA1_(ST147).fna'**	**'20_SD_W_06_(ST368).fna'**	82.5	[78.6 - 85.8]	92.3	[90.3 - 94.0]	87.0	[84.0 - 89.6]	0.42
**'732_EFLS_(ST16).fna'**	**'BIOML_A5_(ST875).fna'**	82.1	[78.2 - 85.4]	92.3	[90.3 - 94.0]	86.7	[83.6 - 89.3]	0.03
**'CL9797_(ST525).fasta'**	**'CL9824_(ST631).fasta'**	77.4	[73.4 - 80.9]	92.2	[90.2 - 93.9]	82.7	[79.4 - 85.6]	0.6
**'BT00.E.21_(ST23).fasta'**	**'KUB3007_(ST729).fna'**	70.5	[66.5 - 74.1]	92.2	[90.1 - 93.8]	76.5	[73.1 - 79.7]	0.07
**'14EA1_(ST147).fna'**	**'LB00.E.122_(ST21).fasta'**	80.7	[76.8 - 84.1]	92.2	[90.1 - 93.8]	85.5	[82.4 - 88.2]	0.42
**'4928STDY7071351_(ST30).fna'**	**'CVM_N60027F_(ST862).fna'**	80.2	[76.3 - 83.6]	92.2	[90.2 - 93.9]	85.1	[82.0 - 87.8]	0.37
**'14EA1_(ST147).fna'**	**'E1_(ST40).fna'**	77.9	[73.9 - 81.4]	92.2	[90.1 - 93.8]	83.2	[79.9 - 86.0]	0.41
**'4928STDY7071435_(ST40).fna'**	**'CL9943_(ST4).fasta'**	74.3	[70.3 - 77.9]	92.2	[90.1 - 93.8]	80.0	[76.6 - 83.0]	0.74
**'EN788_(ST82).fna'**	**'KUB3007_(ST729).fna'**	69.5	[65.6 - 73.2]	92.2	[90.1 - 93.8]	75.7	[72.2 - 78.9]	0.24
**'4928STDY7071351_(ST30).fna'**	**'Praia.S.M2.C4_(ST62).fasta'**	83.3	[79.5 - 86.6]	92.2	[90.1 - 93.8]	87.7	[84.7 - 90.2]	0.18
**'244_EFLS_(ST443).fna'**	**'D32_(ST40).fna'**	80.1	[76.1 - 83.5]	92.2	[90.2 - 93.9]	85.0	[81.8 - 87.7]	0.07
**'P9 CL A7_(ST4).fna'**	**'T11_(ST65).fna'**	82.7	[78.8 - 85.9]	92.2	[90.1 - 93.8]	87.1	[84.1 - 89.7]	0.62
**'EN788_(ST82).fna'**	**'P9 CL A7_(ST4).fna'**	78.1	[74.1 - 81.6]	92.2	[90.1 - 93.8]	83.3	[80.0 - 86.1]	0.11
**'CVM_N60027F_(ST862).fna'**	**'T11_(ST65).fna'**	92.6	[89.7 - 94.7]	92.2	[90.2 - 93.9]	94.7	[92.7 - 96.2]	0.11
**'4928STDY7071435_(ST40).fna'**	**'P9 CL A7_(ST4).fna'**	72.2	[68.2 - 75.8]	92.2	[90.2 - 93.9]	78.1	[74.7 - 81.2]	0.68
**'4928STDY7071351_(ST30).fna'**	**'EnGen0400_(ST86).fna'**	76.7	[72.7 - 80.3]	92.2	[90.2 - 93.9]	82.1	[78.8 - 85.0]	0.5
**'20_SD_W_06_(ST368).fna'**	**'EF348_(ST16).fna'**	76.8	[72.8 - 80.4]	92.2	[90.1 - 93.8]	82.2	[78.9 - 85.1]	0.19
**'CL9943_(ST4).fasta'**	**'T11_(ST65).fna'**	76.6	[72.7 - 80.2]	92.2	[90.2 - 93.9]	82.1	[78.7 - 85.0]	0.68
**'D32_(ST40).fna'**	**'EF348_(ST16).fna'**	75.8	[71.9 - 79.4]	92.2	[90.1 - 93.8]	81.4	[78.0 - 84.3]	0.11
**'302EA1_(ST122).fna'**	**'CL9172_(ST21).fasta'**	84.6	[80.8 - 87.7]	92.2	[90.2 - 93.9]	88.7	[85.8 - 91.1]	0.04
**'244_EFLS_(ST443).fna'**	**'T11_(ST65).fna'**	83.7	[79.9 - 86.9]	92.2	[90.2 - 93.9]	88.0	[85.1 - 90.5]	0.17
**'244_EFLS_(ST443).fna'**	**'EF348_(ST16).fna'**	80.9	[77.0 - 84.3]	92.2	[90.2 - 93.9]	85.7	[82.6 - 88.4]	0.04
**'4928STDY7071435_(ST40).fna'**	*Enterococcus faecalis* NBRC 100480	76.5	[72.5 - 80.0]	92.2	[90.1 - 93.8]	81.9	[78.6 - 84.8]	0.2
**'E1_(ST40).fna'**	**'P9 CL A7_(ST4).fna'**	74.1	[70.1 - 77.7]	92.2	[90.2 - 93.9]	79.8	[76.4 - 82.8]	0.31
**'CL9824_(ST631).fasta'**	**'CL11199_(ST778).fasta'**	75.0	[71.0 - 78.6]	92.2	[90.1 - 93.8]	80.6	[77.2 - 83.6]	0.52

Query	Subject	d_0	C.I. d_0	d_4	C.I. d_4	d_6	C.I. d_6	Diff. G+C Percent
**'CVM_N60027F_(ST862).fna'**	**'Praia.S.M2.C4_(ST62).fasta'**	82.7	[78.9 - 86.0]	92.2	[90.1 - 93.9]	87.2	[84.2 - 89.7]	0.19
**'14EA1_(ST147).fna'**	**'244_EFLS_(ST443).fna'**	83.9	[80.1 - 87.1]	92.2	[90.2 - 93.9]	88.1	[85.2 - 90.6]	0.27
**'14EA1_(ST147).fna'**	**'4928STDY7071435_(ST40).fna'**	75.7	[71.7 - 79.3]	92.1	[90.0 - 93.8]	81.2	[77.9 - 84.2]	0.05
**'BT00.E.21_(ST23).fasta'**	**'CL9824_(ST631).fasta'**	77.6	[73.6 - 81.1]	92.1	[90.1 - 93.8]	82.9	[79.6 - 85.8]	0.61
**'EN242_(ST82).fna'**	**'EnGen0400_(ST86).fna'**	78.2	[74.2 - 81.7]	92.1	[90.0 - 93.8]	83.4	[80.1 - 86.2]	0.63
**'244_EFLS_(ST443).fna'**	**'4928STDY7071351_(ST30).fna'**	83.4	[79.5 - 86.6]	92.1	[90.0 - 93.7]	87.7	[84.7 - 90.2]	0.31
**'15224_(ST16).fna'**	**'E1_(ST40).fna'**	76.4	[72.4 - 79.9]	92.1	[90.1 - 93.8]	81.8	[78.5 - 84.8]	0.28
**'CVM_N60027F_(ST862).fna'**	*Enterococcus faecalis* NBRC 100480	83.3	[79.5 - 86.5]	92.1	[90.0 - 93.7]	87.7	[84.7 - 90.1]	0.03
**'244_EFLS_(ST443).fna'**	**'19910_(ST116).fna'**	78.7	[74.7 - 82.2]	92.1	[90.0 - 93.7]	83.8	[80.5 - 86.6]	0.21
**'19910_(ST116).fna'**	**'BT00.E.21_(ST23).fasta'**	72.9	[69.0 - 76.6]	92.1	[90.0 - 93.8]	78.8	[75.3 - 81.9]	0.19
**'4928STDY7071435_(ST40).fna'**	**'EN242_(ST82).fna'**	71.5	[67.5 - 75.1]	92.1	[90.0 - 93.8]	77.5	[74.0 - 80.6]	0.67
**'14EA1_(ST147).fna'**	**'302EA1_(ST122).fna'**	85.2	[81.4 - 88.2]	92.1	[90.1 - 93.8]	89.1	[86.3 - 91.5]	0.3
**'20_SD_W_06_(ST368).fna'**	**'19910_(ST116).fna'**	81.0	[77.1 - 84.4]	92.1	[90.0 - 93.7]	85.8	[82.6 - 88.4]	0.06
**'D32_(ST40).fna'**	**'EN242_(ST82).fna'**	72.2	[68.3 - 75.9]	92.1	[90.0 - 93.7]	78.1	[74.7 - 81.2]	0.38
**'CL9943_(ST4).fasta'**	**'EF348_(ST16).fna'**	80.6	[76.7 - 84.0]	92.1	[90.1 - 93.8]	85.5	[82.3 - 88.1]	0.55
**'EnGen0400_(ST86).fna'**	**'Praia.S.M2.C4_(ST62).fasta'**	77.8	[73.8 - 81.3]	92.1	[90.0 - 93.7]	83.1	[79.8 - 85.9]	0.32
**'244_EFLS_(ST443).fna'**	**'BIOML_A5_(ST875).fna'**	80.0	[76.0 - 83.4]	92.1	[90.0 - 93.7]	84.9	[81.7 - 87.6]	0.16
**'CL9924_(ST6).fasta'**	**'CVM_N60027F_(ST862).fna'**	75.9	[72.0 - 79.5]	92.1	[90.0 - 93.8]	81.5	[78.1 - 84.4]	0.41
**'732_EFLS_(ST16).fna'**	**'CL9824_(ST631).fasta'**	86.9	[83.3 - 89.8]	92.1	[90.0 - 93.7]	90.5	[87.8 - 92.6]	0.35
**'CL9824_(ST631).fasta'**	**'F_(ST712).fna'**	79.3	[75.3 - 82.8]	92.0	[89.9 - 93.7]	84.3	[81.1 - 87.1]	0.11
**'209EA1_(ST624).fna'**	**'C138_(ST228).fna'**	92.6	[89.8 - 94.7]	92.0	[89.9 - 93.7]	94.7	[92.7 - 96.2]	0.14
**'4928STDY7071351_(ST30).fna'**	**'BT00.E.21_(ST23).fasta'**	83.2	[79.4 - 86.5]	92.0	[89.9 - 93.7]	87.6	[84.6 - 90.1]	0.08
**'4928STDY7071435_(ST40).fna'**	**'15224_(ST16).fna'**	74.6	[70.7 - 78.3]	92.0	[89.9 - 93.7]	80.3	[76.9 - 83.3]	0.65
**'4928STDY7071351_(ST30).fna'**	*Enterococcus faecalis* NBRC 100480	86.1	[82.4 - 89.1]	92.0	[89.9 - 93.7]	89.9	[87.1 - 92.1]	0.34
**'14EA1_(ST147).fna'**	**'T11_(ST65).fna'**	86.8	[83.2 - 89.7]	92.0	[89.9 - 93.7]	90.4	[87.7 - 92.5]	0.11
**'209EA1_(ST624).fna'**	**'CVM_N55265_(ST228).fna'**	92.2	[89.3 - 94.4]	92.0	[89.9 - 93.7]	94.4	[92.4 - 95.9]	0.15
**'BIOML_A5_(ST875).fna'**	**'CVM_N60027F_(ST862).fna'**	86.6	[83.0 - 89.5]	92.0	[89.9 - 93.7]	90.3	[87.5 - 92.4]	0.22

Query	Subject	d_0	C.I. d_0	d_4	C.I. d_4	d_6	C.I. d_6	Diff. G+C Percent
**'EF348_(ST16).fna'**	**'LB00.E.122_(ST21).fasta'**	83.2	[79.3 - 86.4]	92.0	[89.9 - 93.6]	87.5	[84.5 - 90.0]	0.19
**'CL9943_(ST4).fasta'**	**'E1_(ST40).fna'**	75.8	[71.8 - 79.4]	92.0	[89.9 - 93.7]	81.3	[78.0 - 84.3]	0.37
**'T11_(ST65).fna'**	*Enterococcus faecalis* NBRC 100480	82.4	[78.5 - 85.7]	92.0	[89.9 - 93.6]	86.9	[83.8 - 89.4]	0.14
**'CL9824_(ST631).fasta'**	**'CVM_N60027F_(ST862).fna'**	91.6	[88.5 - 93.8]	92.0	[89.9 - 93.7]	94.0	[91.8 - 95.6]	0.16
**'14EA1_(ST147).fna'**	**'D32_(ST40).fna'**	79.6	[75.7 - 83.1]	92.0	[90.0 - 93.7]	84.6	[81.4 - 87.4]	0.34
**'209EA1_(ST624).fna'**	**'CVM N52662_(ST228).fna'**	90.2	[87.0 - 92.7]	92.0	[89.9 - 93.7]	93.0	[90.6 - 94.7]	0.21
**'732_EFLS_(ST16).fna'**	**'CL9924_(ST6).fasta'**	79.6	[75.7 - 83.1]	92.0	[89.9 - 93.6]	84.6	[81.4 - 87.3]	0.22
**'BT00.E.21_(ST23).fasta'**	**'EN242_(ST82).fna'**	74.0	[70.0 - 77.6]	92.0	[89.9 - 93.7]	79.7	[76.3 - 82.8]	0.05
**'209EA1_(ST624).fna'**	**'C116_(ST228).fna'**	91.2	[88.1 - 93.6]	92.0	[89.9 - 93.7]	93.7	[91.5 - 95.4]	0.15
**'4928STDY7071435_(ST40).fna'**	**'EN788_(ST82).fna'**	71.3	[67.4 - 74.9]	92.0	[89.9 - 93.7]	77.3	[73.8 - 80.4]	0.79
**'4928STDY7071351_(ST30).fna'**	**'CL9943_(ST4).fasta'**	87.1	[83.6 - 90.0]	92.0	[90.0 - 93.7]	90.7	[88.0 - 92.8]	0.2
**'20_SD_W_06_(ST368).fna'**	**'P9 CL A7_(ST4).fna'**	76.4	[72.4 - 80.0]	92.0	[89.9 - 93.7]	81.8	[78.5 - 84.8]	0.31
**'CL9943_(ST4).fasta'**	**'EN242_(ST82).fna'**	69.7	[65.7 - 73.3]	91.9	[89.8 - 93.6]	75.8	[72.3 - 78.9]	0.07
**'CL9172_(ST21).fasta'**	**'CL9924_(ST6).fasta'**	77.9	[73.9 - 81.4]	91.9	[89.9 - 93.6]	83.1	[79.8 - 86.0]	0.29
**'15224_(ST16).fna'**	**'EnGen0400_(ST86).fna'**	78.5	[74.6 - 82.0]	91.9	[89.8 - 93.6]	83.7	[80.4 - 86.5]	0.61
**'244_EFLS_(ST443).fna'**	**'CL9824_(ST631).fasta'**	84.0	[80.3 - 87.2]	91.9	[89.8 - 93.6]	88.2	[85.3 - 90.6]	0.22
**'EN242_(ST82).fna'**	**'KUB3007_(ST729).fna'**	71.3	[67.3 - 74.9]	91.9	[89.8 - 93.6]	77.3	[73.8 - 80.4]	0.12
**'732_EFLS_(ST16).fna'**	**'F1_(ST72).fna'**	78.7	[74.7 - 82.2]	91.9	[89.8 - 93.6]	83.8	[80.5 - 86.6]	0.13
**'302EA1_(ST122).fna'**	**'D32_(ST40).fna'**	78.5	[74.6 - 82.0]	91.9	[89.8 - 93.6]	83.7	[80.4 - 86.5]	0.04
**'20_SD_W_06_(ST368).fna'**	**'CL9924_(ST6).fasta'**	71.2	[67.3 - 74.9]	91.9	[89.8 - 93.6]	77.2	[73.7 - 80.3]	0.21
**'244_EFLS_(ST443).fna'**	**'KUB3007_(ST729).fna'**	76.1	[72.1 - 79.7]	91.9	[89.8 - 93.6]	81.5	[78.2 - 84.5]	0.32
**'LB00.E.122_(ST21).fasta'**	**'T11_(ST65).fna'**	81.8	[77.9 - 85.1]	91.9	[89.8 - 93.6]	86.4	[83.3 - 89.0]	0.32
**'20_SD_W_06_(ST368).fna'**	**'BIOML_A5_(ST875).fna'**	88.3	[84.9 - 91.1]	91.9	[89.8 - 93.6]	91.6	[89.0 - 93.6]	0.02
**'209EA1_(ST624).fna'**	**'R48_(ST228).fna'**	89.9	[86.6 - 92.4]	91.9	[89.8 - 93.6]	92.7	[90.4 - 94.6]	0.17
**'302EA1_(ST122).fna'**	**'19910_(ST116).fna'**	77.5	[73.5 - 81.1]	91.9	[89.8 - 93.6]	82.8	[79.5 - 85.7]	0.18
**'209EA1_(ST624).fna'**	**'EN24_(ST228).fna'**	90.2	[87.0 - 92.7]	91.9	[89.9 - 93.6]	93.0	[90.7 - 94.8]	0.2
**'EnGen0400_(ST86).fna'**	**'T11_(ST65).fna'**	90.6	[87.4 - 93.0]	91.9	[89.8 - 93.6]	93.2	[90.9 - 95.0]	0.03

Query	Subject	d_0	C.I. d_0	d_4	C.I. d_4	d_6	C.I. d_6	Diff. G+C Percent
**'EF348_(ST16).fna'**	**'Praia.S.M2.C4_(ST62).fasta'**	79.8	[75.8 - 83.2]	91.8	[89.6 - 93.5]	84.7	[81.5 - 87.4]	0.17
**'BT00.E.21_(ST23).fasta'**	**'EN788_(ST82).fna'**	74.8	[70.8 - 78.4]	91.8	[89.7 - 93.5]	80.4	[77.0 - 83.4]	0.17
**'14EA1_(ST147).fna'**	**'P9 CL A7_(ST4).fna'**	78.4	[74.5 - 81.9]	91.8	[89.7 - 93.5]	83.6	[80.3 - 86.4]	0.72
**'4928STDY7071435_(ST40).fna'**	**'Praia.S.M2.C4_(ST62).fasta'**	75.6	[71.6 - 79.2]	91.8	[89.7 - 93.5]	81.1	[77.7 - 84.1]	0.35
**'CL9824_(ST631).fasta'**	**'EF348_(ST16).fna'**	81.7	[77.8 - 85.1]	91.8	[89.7 - 93.5]	86.3	[83.2 - 88.9]	0.17
**'BIOML_A5_(ST875).fna'**	**'CL11199_(ST778).fasta'**	73.9	[69.9 - 77.5]	91.8	[89.7 - 93.5]	79.6	[76.2 - 82.6]	0.14
**'302EA1_(ST122).fna'**	**'CL9824_(ST631).fasta'**	83.5	[79.7 - 86.7]	91.8	[89.7 - 93.5]	87.8	[84.8 - 90.2]	0.24
**'302EA1_(ST122).fna'**	**'CL9314_(ST21).fasta'**	84.8	[81.1 - 87.9]	91.8	[89.7 - 93.5]	88.8	[85.9 - 91.2]	0.05
**'CL9172_(ST21).fasta'**	**'CL9824_(ST631).fasta'**	85.5	[81.8 - 88.5]	91.8	[89.6 - 93.5]	89.3	[86.5 - 91.6]	0.28
**'BT00.E.21_(ST23).fasta'**	**'LB00.E.122_(ST21).fasta'**	83.0	[79.2 - 86.3]	91.8	[89.7 - 93.5]	87.4	[84.4 - 89.9]	0.24
**'CL9314_(ST21).fasta'**	**'CL9924_(ST6).fasta'**	77.6	[73.7 - 81.2]	91.8	[89.7 - 93.5]	82.9	[79.6 - 85.8]	0.28
**'CL9172_(ST21).fasta'**	**'T11_(ST65).fna'**	84.6	[80.8 - 87.7]	91.8	[89.7 - 93.5]	88.6	[85.7 - 91.0]	0.23
**'E1_(ST40).fna'**	**'Praia.S.M2.C4_(ST62).fasta'**	77.3	[73.3 - 80.8]	91.8	[89.7 - 93.5]	82.6	[79.3 - 85.5]	0.02
**'CL9824_(ST631).fasta'**	**'EnGen0400_(ST86).fna'**	87.8	[84.3 - 90.6]	91.8	[89.7 - 93.5]	91.1	[88.5 - 93.2]	0.03
**'CL6870_(ST103).fasta'**	**'CL9824_(ST631).fasta'**	80.2	[76.3 - 83.6]	91.8	[89.7 - 93.5]	85.1	[81.9 - 87.8]	0.44
**'302EA1_(ST122).fna'**	**'KUB3007_(ST729).fna'**	76.0	[72.0 - 79.6]	91.8	[89.7 - 93.5]	81.5	[78.1 - 84.4]	0.3
**'209EA1_(ST624).fna'**	**'CVM N53420_(ST228).fna'**	87.1	[83.5 - 90.0]	91.8	[89.7 - 93.5]	90.6	[87.9 - 92.7]	0.29
**'19910_(ST116).fna'**	*Enterococcus faecalis* NBRC 100480	77.9	[73.9 - 81.4]	91.8	[89.7 - 93.5]	83.1	[79.8 - 85.9]	0.23
**'CL9824_(ST631).fasta'**	**'Merz96_(ST103).fna'**	80.0	[76.1 - 83.5]	91.8	[89.7 - 93.5]	84.9	[81.7 - 87.6]	0.11
**'CL9824_(ST631).fasta'**	**'LB00.E.122_(ST21).fasta'**	82.7	[78.8 - 86.0]	91.8	[89.7 - 93.5]	87.1	[84.1 - 89.6]	0.37
**'20_SD_W_06_(ST368).fna'**	**'244_EFLS_(ST443).fna'**	79.6	[75.7 - 83.1]	91.8	[89.7 - 93.5]	84.6	[81.4 - 87.3]	0.14
**'CL9314_(ST21).fasta'**	**'T11_(ST65).fna'**	83.9	[80.1 - 87.1]	91.8	[89.7 - 93.5]	88.1	[85.1 - 90.5]	0.24
**'BIOML_A5_(ST875).fna'**	**'CL9797_(ST525).fasta'**	74.3	[70.4 - 78.0]	91.8	[89.7 - 93.5]	80.0	[76.6 - 83.0]	0.22
**'14EA1_(ST147).fna'**	**'4928STDY7071351_(ST30).fna'**	78.6	[74.6 - 82.1]	91.7	[89.6 - 93.4]	83.7	[80.4 - 86.5]	0.58
**'20_SD_W_06_(ST368).fna'**	**'BT00.E.21_(ST23).fasta'**	76.9	[73.0 - 80.5]	91.7	[89.6 - 93.5]	82.3	[78.9 - 85.2]	0.25
**'CL9824_(ST631).fasta'**	**'EN788_(ST82).fna'**	78.1	[74.1 - 81.6]	91.7	[89.6 - 93.4]	83.3	[80.0 - 86.1]	0.78
**'CVM_N60027F_(ST862).fna'**	**'F1_(ST72).fna'**	81.3	[77.4 - 84.6]	91.7	[89.6 - 93.4]	85.9	[82.8 - 88.6]	0.06

Query	Subject	d_0	C.I. d_0	d_4	C.I. d_4	d_6	C.I. d_6	Diff. G+C Percent
**'CL9824_(ST631).fasta'**	**'CVM_N54595_(ST192).fna'**	81.2	[77.3 - 84.6]	91.7	[89.6 - 93.4]	85.9	[82.7 - 88.5]	0.31
**'CL9924_(ST6).fasta'**	**'EF349_(ST631).fna'**	73.6	[69.6 - 77.2]	91.7	[89.6 - 93.4]	79.3	[75.9 - 82.4]	1.49
**'302EA1_(ST122).fna'**	**'T11_(ST65).fna'**	83.4	[79.6 - 86.6]	91.7	[89.6 - 93.4]	87.7	[84.7 - 90.2]	0.19
**'14EA1_(ST147).fna'**	*Enterococcus faecalis* NBRC 100480	85.1	[81.3 - 88.1]	91.7	[89.6 - 93.5]	89.0	[86.1 - 91.3]	0.25
**'20_SD_W_06_(ST368).fna'**	**'KUB3007_(ST729).fna'**	75.8	[71.8 - 79.3]	91.7	[89.6 - 93.4]	81.2	[77.9 - 84.2]	0.18
**'CL9172_(ST21).fasta'**	*Enterococcus faecalis* NBRC 100480	85.5	[81.8 - 88.6]	91.7	[89.5 - 93.4]	89.4	[86.5 - 91.6]	0.09
**'C116_(ST228).fna'**	**'DSM111623_(ST624).fna'**	88.2	[84.8 - 91.0]	91.7	[89.6 - 93.4]	91.5	[88.9 - 93.5]	0.12
**'302EA1_(ST122).fna'**	**'P9 CL A7_(ST4).fna'**	75.5	[71.5 - 79.1]	91.7	[89.6 - 93.4]	81.0	[77.6 - 84.0]	0.43
**'CL9824_(ST631).fasta'**	**'F1_(ST72).fna'**	80.6	[76.7 - 84.0]	91.7	[89.6 - 93.4]	85.4	[82.2 - 88.0]	0.21
**'CL9824_(ST631).fasta'**	**'EN242_(ST82).fna'**	78.9	[74.9 - 82.4]	91.7	[89.6 - 93.4]	84.0	[80.7 - 86.7]	0.66
**'BT00.E.21_(ST23).fasta'**	**'P9 CL A7_(ST4).fna'**	75.3	[71.3 - 78.9]	91.7	[89.6 - 93.4]	80.8	[77.4 - 83.8]	0.06
**'19910_(ST116).fna'**	**'EnGen0400_(ST86).fna'**	85.0	[81.3 - 88.1]	91.7	[89.5 - 93.4]	89.0	[86.1 - 91.3]	0.4
**'BIOML_A5_(ST875).fna'**	**'CL9772_(ST525).fasta'**	76.7	[72.7 - 80.3]	91.7	[89.5 - 93.4]	82.0	[78.7 - 85.0]	0.14
**'CVM_N60027F_(ST862).fna'**	**'EF349_(ST631).fna'**	88.3	[84.9 - 91.1]	91.7	[89.6 - 93.4]	91.5	[89.0 - 93.5]	1.08
**'CL9314_(ST21).fasta'**	**'CL9824_(ST631).fasta'**	84.9	[81.1 - 88.0]	91.7	[89.6 - 93.4]	88.8	[86.0 - 91.2]	0.29
**'732_EFLS_(ST16).fna'**	**'EF349_(ST631).fna'**	82.2	[78.4 - 85.5]	91.7	[89.6 - 93.4]	86.7	[83.6 - 89.3]	1.27
**'BIOML_A5_(ST875).fna'**	**'P9 CL A7_(ST4).fna'**	79.4	[75.5 - 82.9]	91.7	[89.6 - 93.4]	84.4	[81.2 - 87.2]	0.29
**'CVM_N52587_(STx).fna'**	**'DSM111623_(ST624).fna'**	87.4	[83.9 - 90.3]	91.7	[89.5 - 93.4]	90.8	[88.2 - 92.9]	0.02
**'CL9824_(ST631).fasta'**	**'CL9943_(ST4).fasta'**	72.1	[68.1 - 75.7]	91.6	[89.4 - 93.3]	77.9	[74.5 - 81.0]	0.73
**'4928STDY7071351_(ST30).fna'**	**'EF348_(ST16).fna'**	81.1	[77.2 - 84.5]	91.6	[89.5 - 93.3]	85.8	[82.6 - 88.4]	0.35
**'CVM N53420_(ST228).fna'**	**'DSM111623_(ST624).fna'**	84.0	[80.2 - 87.1]	91.6	[89.4 - 93.3]	88.1	[85.1 - 90.5]	0.03
**'20_SD_W_06_(ST368).fna'**	**'CL9314_(ST21).fasta'**	83.5	[79.7 - 86.7]	91.6	[89.4 - 93.3]	87.7	[84.8 - 90.2]	0.07
**'CL9943_(ST4).fasta'**	**'LB00.E.122_(ST21).fasta'**	78.5	[74.5 - 82.0]	91.6	[89.5 - 93.4]	83.6	[80.3 - 86.4]	0.36
**'EN788_(ST82).fna'**	**'LB00.E.122_(ST21).fasta'**	74.8	[70.9 - 78.5]	91.6	[89.5 - 93.3]	80.4	[77.0 - 83.4]	0.41
**'209EA1_(ST624).fna'**	**'CVM_N52587_(STx).fna'**	92.1	[89.2 - 94.3]	91.6	[89.5 - 93.4]	94.3	[92.2 - 95.8]	0.24
**'732_EFLS_(ST16).fna'**	**'F_(ST712).fna'**	74.5	[70.5 - 78.1]	91.6	[89.5 - 93.4]	80.1	[76.7 - 83.1]	0.23
**'4928STDY7071351_(ST30).fna'**	**'P9 CL A7_(ST4).fna'**	74.8	[70.8 - 78.4]	91.6	[89.5 - 93.4]	80.4	[77.0 - 83.4]	0.14

Query	Subject	d_0	C.I. d_0	d_4	C.I. d_4	d_6	C.I. d_6	Diff. G+C Percent
**'15224_(ST16).fna'**	**'KUB3007_(ST729).fna'**	75.3	[71.3 - 78.9]	91.6	[89.5 - 93.3]	80.8	[77.4 - 83.8]	0.1
**'D32_(ST40).fna'**	**'T11_(ST65).fna'**	79.0	[75.0 - 82.4]	91.6	[89.5 - 93.3]	84.0	[80.7 - 86.8]	0.23
**'CL9924_(ST6).fasta'**	**'CL9943_(ST4).fasta'**	83.2	[79.4 - 86.5]	91.6	[89.4 - 93.3]	87.5	[84.5 - 90.0]	0.16
**'BIOML_A5_(ST875).fna'**	**'CL9172_(ST21).fasta'**	79.7	[75.7 - 83.1]	91.6	[89.5 - 93.3]	84.6	[81.4 - 87.3]	0.1
**'DSM111623_(ST624).fna'**	**'R48_(ST228).fna'**	85.9	[82.2 - 88.9]	91.6	[89.5 - 93.4]	89.6	[86.8 - 91.9]	0.09
**'EF348_(ST16).fna'**	*Enterococcus faecalis* NBRC 100480	81.9	[78.0 - 85.2]	91.6	[89.4 - 93.3]	86.4	[83.3 - 89.0]	0.02
**'CVM_N55265_(ST228).fna'**	**'DSM111623_(ST624).fna'**	89.0	[85.6 - 91.6]	91.6	[89.5 - 93.3]	92.0	[89.5 - 93.9]	0.12
**'CL8682_(ST769).fasta'**	**'CL9824_(ST631).fasta'**	80.4	[76.5 - 83.8]	91.6	[89.5 - 93.4]	85.2	[82.0 - 87.9]	0.4
**'EN788_(ST82).fna'**	*Enterococcus faecalis* NBRC 100480	75.4	[71.4 - 79.0]	91.6	[89.4 - 93.3]	80.9	[77.5 - 83.9]	0.59
**'4928STDY7071351_(ST30).fna'**	**'CL9824_(ST631).fasta'**	77.0	[73.0 - 80.5]	91.6	[89.4 - 93.3]	82.3	[78.9 - 85.2]	0.53
**'BIOML_A5_(ST875).fna'**	**'EF348_(ST16).fna'**	79.1	[75.2 - 82.6]	91.6	[89.4 - 93.3]	84.1	[80.9 - 86.9]	0.21
**'C138_(ST228).fna'**	**'G81_(ST1468).fna'**	86.5	[82.9 - 89.4]	91.6	[89.5 - 93.4]	90.1	[87.4 - 92.3]	0.05
**'Praia.S.M2.C4_(ST62).fasta'**	**'T11_(ST65).fna'**	79.7	[75.7 - 83.1]	91.6	[89.5 - 93.3]	84.6	[81.4 - 87.3]	0.29
**'20_SD_W_06_(ST368).fna'**	**'CL9824_(ST631).fasta'**	87.7	[84.2 - 90.5]	91.6	[89.5 - 93.4]	91.1	[88.4 - 93.1]	0.36
**'244_EFLS_(ST443).fna'**	**'LB00.E.122_(ST21).fasta'**	83.5	[79.7 - 86.7]	91.6	[89.5 - 93.3]	87.7	[84.8 - 90.2]	0.15
**'14EA1_(ST147).fna'**	**'CL9824_(ST631).fasta'**	84.6	[80.9 - 87.8]	91.6	[89.4 - 93.3]	88.6	[85.7 - 91.0]	0.06
**'CL9924_(ST6).fasta'**	**'EnGen0400_(ST86).fna'**	76.2	[72.2 - 79.8]	91.6	[89.4 - 93.3]	81.6	[78.3 - 84.6]	0.54
**'CVM N52662_(ST228).fna'**	**'DSM111623_(ST624).fna'**	86.7	[83.1 - 89.6]	91.6	[89.4 - 93.3]	90.2	[87.5 - 92.4]	0.06
**'F_(ST712).fna'**	**'T11_(ST65).fna'**	79.6	[75.6 - 83.0]	91.6	[89.4 - 93.3]	84.5	[81.3 - 87.3]	0.06
**'20_SD_W_06_(ST368).fna'**	**'15224_(ST16).fna'**	72.4	[68.4 - 76.0]	91.6	[89.5 - 93.3]	78.2	[74.8 - 81.3]	0.28
**'15224_(ST16).fna'**	**'EN242_(ST82).fna'**	76.1	[72.1 - 79.7]	91.6	[89.4 - 93.3]	81.5	[78.1 - 84.4]	0.02
**'E1_(ST40).fna'**	**'T11_(ST65).fna'**	79.9	[76.0 - 83.4]	91.6	[89.5 - 93.3]	84.8	[81.6 - 87.5]	0.31
**'C138_(ST228).fna'**	**'DSM111623_(ST624).fna'**	89.2	[85.9 - 91.8]	91.6	[89.4 - 93.3]	92.2	[89.7 - 94.1]	0.13
**'BIOML_A5_(ST875).fna'**	**'BT00.E.21_(ST23).fasta'**	78.5	[74.6 - 82.0]	91.6	[89.4 - 93.3]	83.6	[80.3 - 86.4]	0.23
**'CVM N53420_(ST228).fna'**	**'G81_(ST1468).fna'**	89.5	[86.2 - 92.1]	91.5	[89.4 - 93.2]	92.4	[89.9 - 94.2]	0.11
**'CL9824_(ST631).fasta'**	**'KUB3007_(ST729).fna'**	79.7	[75.8 - 83.2]	91.5	[89.3 - 93.2]	84.6	[81.4 - 87.4]	0.54
**'15224_(ST16).fna'**	**'P9 CL A7_(ST4).fna'**	83.8	[80.0 - 87.0]	91.5	[89.4 - 93.2]	88.0	[85.0 - 90.4]	0.03

Query	Subject	d_0	C.I. d_0	d_4	C.I. d_4	d_6	C.I. d_6	Diff. G+C Percent
**'20_SD_W_06_(ST368).fna'**	**'4928STDY7071351_(ST30).fna'**	76.3	[72.3 - 79.8]	91.5	[89.4 - 93.2]	81.6	[78.3 - 84.6]	0.17
**'CL9314_(ST21).fasta'**	*Enterococcus faecalis* NBRC 100480	85.8	[82.2 - 88.8]	91.5	[89.3 - 93.2]	89.6	[86.8 - 91.8]	0.1
**'CL9824_(ST631).fasta'**	**'E1_(ST40).fna'**	79.6	[75.6 - 83.0]	91.5	[89.4 - 93.3]	84.5	[81.2 - 87.2]	0.36
**'C116_(ST228).fna'**	**'G81_(ST1468).fna'**	88.8	[85.4 - 91.5]	91.5	[89.3 - 93.2]	91.9	[89.4 - 93.8]	0.04
**'CL9824_(ST631).fasta'**	**'P9 CL A7_(ST4).fna'**	80.4	[76.5 - 83.8]	91.5	[89.3 - 93.2]	85.2	[82.0 - 87.9]	0.67
**'15224_(ST16).fna'**	**'T11_(ST65).fna'**	78.6	[74.7 - 82.1]	91.5	[89.3 - 93.2]	83.7	[80.4 - 86.5]	0.59
**'KUB3007_(ST729).fna'**	**'T11_(ST65).fna'**	81.4	[77.5 - 84.8]	91.5	[89.3 - 93.2]	86.0	[82.9 - 88.6]	0.49
**'20_SD_W_06_(ST368).fna'**	**'Praia.S.M2.C4_(ST62).fasta'**	75.9	[72.0 - 79.5]	91.5	[89.3 - 93.2]	81.4	[78.0 - 84.3]	0.02
**'19910_(ST116).fna'**	**'T11_(ST65).fna'**	86.0	[82.4 - 89.0]	91.5	[89.3 - 93.2]	89.7	[86.9 - 92.0]	0.37
**'14EA1_(ST147).fna'**	**'BIOML_A5_(ST875).fna'**	83.0	[79.1 - 86.2]	91.5	[89.4 - 93.3]	87.3	[84.3 - 89.8]	0.43
**'EF348_(ST16).fna'**	**'EF349_(ST631).fna'**	79.2	[75.3 - 82.7]	91.5	[89.3 - 93.2]	84.2	[80.9 - 87.0]	1.09
**'EN242_(ST82).fna'**	*Enterococcus faecalis* NBRC 100480	74.2	[70.2 - 77.8]	91.5	[89.4 - 93.2]	79.8	[76.4 - 82.8]	0.47
**'BT00.E.21_(ST23).fasta'**	**'CL9943_(ST4).fasta'**	77.2	[73.2 - 80.8]	91.5	[89.3 - 93.2]	82.5	[79.1 - 85.4]	0.12
**'EnGen0400_(ST86).fna'**	**'F_(ST712).fna'**	75.7	[71.7 - 79.3]	91.5	[89.3 - 93.2]	81.1	[77.8 - 84.1]	0.09
**'EN24_(ST228).fna'**	**'G81_(ST1468).fna'**	85.1	[81.3 - 88.2]	91.5	[89.3 - 93.2]	89.0	[86.1 - 91.3]	0.01
**'20_SD_W_06_(ST368).fna'**	**'CL9172_(ST21).fasta'**	84.2	[80.4 - 87.3]	91.5	[89.4 - 93.3]	88.3	[85.3 - 90.7]	0.08
**'BIOML_A5_(ST875).fna'**	**'EnGen0400_(ST86).fna'**	85.0	[81.3 - 88.1]	91.5	[89.4 - 93.2]	88.9	[86.0 - 91.2]	0.35
**'BT00.E.21_(ST23).fasta'**	**'F_(ST712).fna'**	74.0	[70.0 - 77.6]	91.5	[89.3 - 93.2]	79.7	[76.2 - 82.7]	0.5
**'19910_(ST116).fna'**	**'Praia.S.M2.C4_(ST62).fasta'**	78.9	[74.9 - 82.4]	91.5	[89.4 - 93.3]	83.9	[80.6 - 86.7]	0.08
**'20_SD_W_06_(ST368).fna'**	**'D32_(ST40).fna'**	77.4	[73.4 - 80.9]	91.4	[89.3 - 93.2]	82.6	[79.3 - 85.5]	0.08
**'CL9824_(ST631).fasta'**	**'L14_(ST330).fna'**	90.7	[87.5 - 93.1]	91.4	[89.2 - 93.1]	93.3	[91.0 - 95.0]	0.1
**'CVM_N55265_(ST228).fna'**	**'G81_(ST1468).fna'**	87.9	[84.4 - 90.7]	91.4	[89.2 - 93.2]	91.2	[88.5 - 93.2]	0.04
**'CVM_N60027F_(ST862).fna'**	**'L14_(ST330).fna'**	85.5	[81.8 - 88.5]	91.4	[89.2 - 93.1]	89.3	[86.4 - 91.6]	0.05
**'CL9314_(ST21).fasta'**	**'F1_(ST72).fna'**	78.4	[74.4 - 81.9]	91.4	[89.3 - 93.2]	83.5	[80.2 - 86.3]	0.08
**'CL9172_(ST21).fasta'**	**'F1_(ST72).fna'**	79.2	[75.3 - 82.7]	91.4	[89.3 - 93.2]	84.2	[80.9 - 86.9]	0.06
**'732_EFLS_(ST16).fna'**	**'L14_(ST330).fna'**	80.4	[76.5 - 83.8]	91.4	[89.2 - 93.1]	85.2	[82.0 - 87.9]	0.24
**'15224_(ST16).fna'**	**'CL9824_(ST631).fasta'**	76.7	[72.7 - 80.3]	91.4	[89.3 - 93.2]	82.0	[78.7 - 84.9]	0.64

Query	Subject	d_0	C.I. d_0	d_4	C.I. d_4	d_6	C.I. d_6	Diff. G+C Percent
**'20_SD_W_06_(ST368).fna'**	*Enterococcus faecalis* NBRC 100480	80.6	[76.6 - 84.0]	91.4	[89.2 - 93.1]	85.3	[82.1 - 88.0]	0.17
**'302EA1_(ST122).fna'**	**'BIOML_A5_(ST875).fna'**	80.2	[76.2 - 83.6]	91.4	[89.2 - 93.1]	84.9	[81.8 - 87.7]	0.14
**'EN242_(ST82).fna'**	**'F1_(ST72).fna'**	73.2	[69.2 - 76.9]	91.4	[89.2 - 93.2]	78.9	[75.5 - 82.0]	0.45
**'CL9924_(ST6).fasta'**	**'EN242_(ST82).fna'**	73.9	[69.9 - 77.5]	91.4	[89.2 - 93.1]	79.5	[76.1 - 82.6]	0.09
**'EN788_(ST82).fna'**	**'F1_(ST72).fna'**	74.3	[70.3 - 77.9]	91.4	[89.3 - 93.2]	79.9	[76.5 - 82.9]	0.57
**'CL9172_(ST21).fasta'**	**'EF349_(ST631).fna'**	80.8	[76.9 - 84.2]	91.4	[89.2 - 93.2]	85.5	[82.4 - 88.2]	1.2
**'CL9824_(ST631).fasta'**	**'D32_(ST40).fna'**	78.8	[74.8 - 82.3]	91.4	[89.2 - 93.1]	83.8	[80.5 - 86.6]	0.28
**'CL9172_(ST21).fasta'**	**'Praia.S.M2.C4_(ST62).fasta'**	83.6	[79.8 - 86.8]	91.4	[89.3 - 93.2]	87.8	[84.8 - 90.2]	0.07
**'19910_(ST116).fna'**	**'CL9824_(ST631).fasta'**	80.6	[76.7 - 84.0]	91.4	[89.3 - 93.2]	85.3	[82.2 - 88.0]	0.42
**'4928STDY7071351_(ST30).fna'**	**'D32_(ST40).fna'**	77.8	[73.8 - 81.3]	91.4	[89.2 - 93.1]	82.9	[79.6 - 85.8]	0.24
**'Merz96_(ST103).fna'**	**'T11_(ST65).fna'**	79.9	[76.0 - 83.4]	91.4	[89.2 - 93.1]	84.8	[81.5 - 87.5]	0.05
**'20_SD_W_06_(ST368).fna'**	**'CL9943_(ST4).fasta'**	70.2	[66.3 - 73.9]	91.4	[89.3 - 93.2]	76.2	[72.7 - 79.4]	0.37
**'CL9314_(ST21).fasta'**	**'EF349_(ST631).fna'**	80.3	[76.4 - 83.7]	91.4	[89.2 - 93.1]	85.1	[81.9 - 87.8]	1.21
**'4928STDY7071435_(ST40).fna'**	**'CL9824_(ST631).fasta'**	77.4	[73.4 - 80.9]	91.4	[89.2 - 93.2]	82.6	[79.3 - 85.5]	0.01
**'19910_(ST116).fna'**	**'CL9943_(ST4).fasta'**	75.6	[71.6 - 79.1]	91.4	[89.2 - 93.1]	81.0	[77.6 - 84.0]	0.3
**'F1_(ST72).fna'**	**'T11_(ST65).fna'**	82.9	[79.1 - 86.2]	91.4	[89.2 - 93.1]	87.2	[84.2 - 89.8]	0.16
**'KUB3007_(ST729).fna'**	*Enterococcus faecalis* NBRC 100480	74.8	[70.8 - 78.4]	91.4	[89.3 - 93.2]	80.3	[76.9 - 83.4]	0.35
**'DSM111623_(ST624).fna'**	**'EN24_(ST228).fna'**	87.6	[84.1 - 90.5]	91.4	[89.2 - 93.1]	90.9	[88.3 - 93.0]	0.07
**'244_EFLS_(ST443).fna'**	**'CL9943_(ST4).fasta'**	76.3	[72.3 - 79.9]	91.3	[89.1 - 93.0]	81.6	[78.3 - 84.6]	0.51
**'20_SD_W_06_(ST368).fna'**	**'CL11199_(ST778).fasta'**	70.6	[66.7 - 74.3]	91.3	[89.2 - 93.1]	76.6	[73.1 - 79.7]	0.16
**'244_EFLS_(ST443).fna'**	**'P9 CL A7_(ST4).fna'**	75.8	[71.8 - 79.4]	91.3	[89.1 - 93.1]	81.2	[77.8 - 84.2]	0.45
**'D32_(ST40).fna'**	**'KUB3007_(ST729).fna'**	72.9	[68.9 - 76.5]	91.3	[89.1 - 93.0]	78.6	[75.2 - 81.7]	0.26
**'CL9824_(ST631).fasta'**	**'Praia.S.M2.C4_(ST62).fasta'**	80.5	[76.6 - 83.9]	91.3	[89.1 - 93.0]	85.2	[82.0 - 87.9]	0.34
**'20_SD_W_06_(ST368).fna'**	**'LB00.E.122_(ST21).fasta'**	81.8	[77.9 - 85.1]	91.3	[89.2 - 93.1]	86.3	[83.2 - 88.9]	0.01
**'302EA1_(ST122).fna'**	**'EN242_(ST82).fna'**	73.3	[69.4 - 77.0]	91.3	[89.2 - 93.1]	79.0	[75.6 - 82.1]	0.42
**'E1_(ST40).fna'**	**'F1_(ST72).fna'**	76.3	[72.4 - 79.9]	91.3	[89.2 - 93.1]	81.7	[78.3 - 84.6]	0.15
**'15224_(ST16).fna'**	**'LB00.E.122_(ST21).fasta'**	78.1	[74.1 - 81.6]	91.3	[89.2 - 93.1]	83.2	[79.9 - 86.0]	0.27

Query	Subject	d_0	C.I. d_0	d_4	C.I. d_4	d_6	C.I. d_6	Diff. G+C Percent
**'302EA1_(ST122).fna'**	**'EN788_(ST82).fna'**	74.1	[70.1 - 77.7]	91.3	[89.1 - 93.1]	79.7	[76.3 - 82.7]	0.54
**'EF349_(ST631).fna'**	**'LB00.E.122_(ST21).fasta'**	78.3	[74.4 - 81.8]	91.3	[89.1 - 93.1]	83.4	[80.1 - 86.2]	1.29
**'P9 CL A7_(ST4).fna'**	**'Praia.S.M2.C4_(ST62).fasta'**	73.8	[69.8 - 77.4]	91.3	[89.1 - 93.1]	79.4	[76.0 - 82.5]	0.33
**'EnGen0400_(ST86).fna'**	**'F1_(ST72).fna'**	80.2	[76.3 - 83.7]	91.3	[89.2 - 93.1]	85.0	[81.8 - 87.7]	0.19
**'CL6870_(ST103).fasta'**	**'T11_(ST65).fna'**	79.5	[75.5 - 82.9]	91.3	[89.1 - 93.1]	84.3	[81.1 - 87.1]	0.38
**'D32_(ST40).fna'**	**'P9 CL A7_(ST4).fna'**	72.5	[68.6 - 76.2]	91.3	[89.2 - 93.1]	78.3	[74.9 - 81.4]	0.39
**'CL9824_(ST631).fasta'**	*Enterococcus faecalis* NBRC 100480	83.4	[79.6 - 86.6]	91.3	[89.1 - 93.1]	87.6	[84.6 - 90.1]	0.19
**'244_EFLS_(ST443).fna'**	**'EF349_(ST631).fna'**	79.2	[75.2 - 82.6]	91.3	[89.2 - 93.1]	84.1	[80.9 - 86.9]	1.14
**'BIOML_A5_(ST875).fna'**	*Enterococcus faecalis* NBRC 100480	79.0	[75.1 - 82.5]	91.3	[89.1 - 93.0]	84.0	[80.7 - 86.8]	0.19
**'BIOML_A5_(ST875).fna'**	**'CL9314_(ST21).fasta'**	79.4	[75.4 - 82.9]	91.3	[89.1 - 93.1]	84.3	[81.1 - 87.1]	0.09
**'20_SD_W_06_(ST368).fna'**	**'Merz96_(ST103).fna'**	73.5	[69.5 - 77.1]	91.3	[89.2 - 93.1]	79.2	[75.8 - 82.2]	0.25
**'4928STDY7071351_(ST30).fna'**	**'EN242_(ST82).fna'**	72.0	[68.0 - 75.6]	91.2	[89.0 - 93.0]	77.8	[74.3 - 80.9]	0.13
**'4928STDY7071351_(ST30).fna'**	**'T11_(ST65).fna'**	78.9	[74.9 - 82.3]	91.2	[89.0 - 93.0]	83.8	[80.6 - 86.6]	0.48
**'CL9314_(ST21).fasta'**	**'Praia.S.M2.C4_(ST62).fasta'**	83.9	[80.1 - 87.1]	91.2	[89.0 - 93.0]	88.0	[85.0 - 90.4]	0.05
**'732_EFLS_(ST16).fna'**	**'CVM_N54595_(ST192).fna'**	76.2	[72.2 - 79.8]	91.2	[89.1 - 93.0]	81.6	[78.2 - 84.5]	0.03
**'CL9772_(ST525).fasta'**	**'EF349_(ST631).fna'**	76.7	[72.7 - 80.3]	91.2	[89.0 - 92.9]	82.0	[78.6 - 84.9]	1.44
**'CVM_N52587_(STx).fna'**	**'G81_(ST1468).fna'**	88.4	[84.9 - 91.1]	91.2	[89.0 - 92.9]	91.5	[88.9 - 93.5]	0.06
**'14EA1_(ST147).fna'**	**'CL9943_(ST4).fasta'**	76.3	[72.3 - 79.9]	91.2	[89.0 - 93.0]	81.6	[78.2 - 84.5]	0.78
**'G81_(ST1468).fna'**	**'R48_(ST228).fna'**	89.2	[85.9 - 91.9]	91.2	[89.0 - 93.0]	92.1	[89.7 - 94.0]	0.01
**'E1_(ST40).fna'**	**'F_(ST712).fna'**	72.3	[68.3 - 75.9]	91.2	[89.1 - 93.0]	78.1	[74.6 - 81.2]	0.25
**'4928STDY7071351_(ST30).fna'**	**'F1_(ST72).fna'**	80.5	[76.6 - 83.9]	91.2	[89.1 - 93.0]	85.2	[82.0 - 87.9]	0.31
**'244_EFLS_(ST443).fna'**	**'15224_(ST16).fna'**	76.4	[72.5 - 80.0]	91.2	[89.0 - 93.0]	81.8	[78.4 - 84.7]	0.42
**'BIOML_A5_(ST875).fna'**	**'D32_(ST40).fna'**	75.7	[71.7 - 79.2]	91.2	[89.0 - 92.9]	81.0	[77.7 - 84.0]	0.1
**'CVM N52662_(ST228).fna'**	**'G81_(ST1468).fna'**	88.2	[84.7 - 90.9]	91.2	[89.0 - 93.0]	91.3	[88.7 - 93.4]	0.02
**'CL9924_(ST6).fasta'**	**'EN788_(ST82).fna'**	74.9	[70.9 - 78.5]	91.2	[89.0 - 93.0]	80.4	[77.0 - 83.4]	0.21
**'20_SD_W_06_(ST368).fna'**	**'CL9797_(ST525).fasta'**	71.9	[67.9 - 75.5]	91.2	[89.0 - 93.0]	77.7	[74.2 - 80.8]	0.24
**'CL9943_(ST4).fasta'**	**'EN788_(ST82).fna'**	70.9	[66.9 - 74.5]	91.2	[89.0 - 93.0]	76.8	[73.3 - 79.9]	0.05

Query	Subject	d_0	C.I. d_0	d_4	C.I. d_4	d_6	C.I. d_6	Diff. G+C Percent
**'302EA1_(ST122).fna'**	**'15224_(ST16).fna'**	74.3	[70.3 - 77.9]	91.2	[89.0 - 93.0]	79.8	[76.4 - 82.9]	0.4
**'20_SD_W_06_(ST368).fna'**	**'302EA1_(ST122).fna'**	81.8	[77.9 - 85.1]	91.2	[89.0 - 93.0]	86.3	[83.1 - 88.9]	0.12
**'244_EFLS_(ST443).fna'**	**'CL9924_(ST6).fasta'**	75.4	[71.4 - 79.0]	91.2	[89.0 - 92.9]	80.8	[77.4 - 83.8]	0.35
**'EF349_(ST631).fna'**	**'Merz96_(ST103).fna'**	76.3	[72.3 - 79.9]	91.2	[89.0 - 92.9]	81.6	[78.3 - 84.6]	1.03
**'20_SD_W_06_(ST368).fna'**	**'E1_(ST40).fna'**	80.9	[77.0 - 84.3]	91.2	[89.0 - 92.9]	85.5	[82.4 - 88.2]	0.0
**'BIOML_A5_(ST875).fna'**	**'CL9943_(ST4).fasta'**	75.1	[71.1 - 78.7]	91.2	[89.0 - 93.0]	80.5	[77.1 - 83.5]	0.35
**'20_SD_W_06_(ST368).fna'**	**'4928STDY7071435_(ST40).fna'**	78.6	[74.6 - 82.1]	91.1	[88.9 - 92.9]	83.6	[80.3 - 86.4]	0.37
**'4928STDY7071435_(ST40).fna'**	**'F_(ST712).fna'**	71.0	[67.0 - 74.6]	91.1	[88.9 - 92.9]	76.9	[73.4 - 80.0]	0.12
**'F_(ST712).fna'**	*Enterococcus faecalis* NBRC 100480	80.0	[76.1 - 83.5]	91.1	[88.9 - 92.9]	84.8	[81.6 - 87.5]	0.08
**'EN242_(ST82).fna'**	**'F_(ST712).fna'**	67.7	[63.8 - 71.3]	91.1	[88.9 - 92.9]	73.8	[70.3 - 77.0]	0.55
**'4928STDY7071435_(ST40).fna'**	**'T11_(ST65).fna'**	78.2	[74.2 - 81.7]	91.1	[89.0 - 92.9]	83.2	[79.9 - 86.1]	0.06
**'KUB3007_(ST729).fna'**	**'Praia.S.M2.C4_(ST62).fasta'**	74.2	[70.3 - 77.9]	91.1	[88.9 - 92.9]	79.8	[76.4 - 82.8]	0.2
**'CL11199_(ST778).fasta'**	**'CVM_N60027F_(ST862).fna'**	74.1	[70.1 - 77.7]	91.1	[88.9 - 92.9]	79.6	[76.2 - 82.7]	0.36
**'302EA1_(ST122).fna'**	**'CVM_N54595_(ST192).fna'**	80.8	[76.9 - 84.2]	91.1	[88.9 - 92.9]	85.4	[82.3 - 88.1]	0.07
**'EF348_(ST16).fna'**	**'F1_(ST72).fna'**	76.5	[72.5 - 80.1]	91.1	[89.0 - 92.9]	81.8	[78.4 - 84.7]	0.04
**'CL8682_(ST769).fasta'**	**'T11_(ST65).fna'**	80.1	[76.2 - 83.6]	91.1	[88.9 - 92.9]	84.9	[81.7 - 87.6]	0.35
**'20_SD_W_06_(ST368).fna'**	**'F1_(ST72).fna'**	77.5	[73.5 - 81.1]	91.1	[88.9 - 92.9]	82.7	[79.3 - 85.5]	0.15
**'302EA1_(ST122).fna'**	**'BT00.E.21_(ST23).fasta'**	79.3	[75.3 - 82.8]	91.1	[88.9 - 92.9]	84.2	[80.9 - 87.0]	0.37
**'302EA1_(ST122).fna'**	**'LB00.E.122_(ST21).fasta'**	84.0	[80.2 - 87.1]	91.1	[88.9 - 92.9]	88.0	[85.1 - 90.5]	0.13
**'302EA1_(ST122).fna'**	**'Praia.S.M2.C4_(ST62).fasta'**	79.6	[75.6 - 83.0]	91.1	[88.9 - 92.9]	84.4	[81.2 - 87.2]	0.1
**'15224_(ST16).fna'**	**'19910_(ST116).fna'**	79.2	[75.3 - 82.7]	91.1	[88.9 - 92.9]	84.1	[80.9 - 86.9]	0.22
**'CL9797_(ST525).fasta'**	**'EF349_(ST631).fna'**	76.3	[72.3 - 79.9]	91.0	[88.8 - 92.8]	81.6	[78.2 - 84.5]	1.52
**'4928STDY7071435_(ST40).fna'**	**'F1_(ST72).fna'**	74.0	[70.0 - 77.6]	91.0	[88.8 - 92.8]	79.5	[76.1 - 82.6]	0.22
**'4928STDY7071351_(ST30).fna'**	**'19910_(ST116).fna'**	75.8	[71.8 - 79.4]	91.0	[88.8 - 92.8]	81.2	[77.8 - 84.1]	0.1
**'BT00.E.21_(ST23).fasta'**	**'CL9924_(ST6).fasta'**	77.4	[73.4 - 81.0]	91.0	[88.8 - 92.8]	82.6	[79.2 - 85.4]	0.04
**'4928STDY7071435_(ST40).fna'**	**'EF349_(ST631).fna'**	73.6	[69.6 - 77.2]	91.0	[88.8 - 92.8]	79.2	[75.8 - 82.2]	0.91
**'EF349_(ST631).fna'**	**'EnGen0400_(ST86).fna'**	83.5	[79.7 - 86.7]	91.0	[88.8 - 92.8]	87.6	[84.6 - 90.1]	0.95

Query	Subject	d_0	C.I. d_0	d_4	C.I. d_4	d_6	C.I. d_6	Diff. G+C Percent
**'CL11199_(ST778).fasta'**	**'EF349_(ST631).fna'**	73.9	[69.9 - 77.5]	91.0	[88.8 - 92.8]	79.4	[76.0 - 82.5]	1.44
**'CL9943_(ST4).fasta'**	**'Praia.S.M2.C4_(ST62).fasta'**	76.4	[72.4 - 80.0]	91.0	[88.8 - 92.8]	81.7	[78.3 - 84.6]	0.38
**'BIOML_A5_(ST875).fna'**	**'E1_(ST40).fna'**	77.9	[73.9 - 81.4]	91.0	[88.8 - 92.8]	83.0	[79.7 - 85.8]	0.02
**'E1_(ST40).fna'**	**'EF349_(ST631).fna'**	75.7	[71.7 - 79.2]	91.0	[88.8 - 92.8]	81.0	[77.6 - 84.0]	1.28
**'CL9797_(ST525).fasta'**	**'CVM_N60027F_(ST862).fna'**	75.6	[71.6 - 79.2]	91.0	[88.8 - 92.8]	81.0	[77.6 - 83.9]	0.44
**'E1_(ST40).fna'**	**'L14_(ST330).fna'**	78.3	[74.3 - 81.8]	91.0	[88.8 - 92.8]	83.3	[80.0 - 86.2]	0.26
**'CL6870_(ST103).fasta'**	**'CL9924_(ST6).fasta'**	85.0	[81.2 - 88.1]	90.9	[88.6 - 92.7]	88.8	[85.9 - 91.1]	0.13
**'302EA1_(ST122).fna'**	**'F1_(ST72).fna'**	80.6	[76.7 - 84.0]	90.9	[88.6 - 92.7]	85.3	[82.1 - 88.0]	0.03
**'14EA1_(ST147).fna'**	**'CL9924_(ST6).fasta'**	74.3	[70.3 - 77.9]	90.9	[88.7 - 92.7]	79.8	[76.4 - 82.8]	0.62
**'BIOML_A5_(ST875).fna'**	**'F1_(ST72).fna'**	80.1	[76.2 - 83.5]	90.9	[88.7 - 92.7]	84.8	[81.6 - 87.6]	0.17
**'732_EFLS_(ST16).fna'**	**'CL9772_(ST525).fasta'**	79.0	[75.0 - 82.4]	90.9	[88.7 - 92.7]	83.9	[80.6 - 86.7]	0.17
**'CL9172_(ST21).fasta'**	**'CVM_N54595_(ST192).fna'**	78.4	[74.4 - 81.9]	90.9	[88.7 - 92.8]	83.4	[80.1 - 86.2]	0.03
**'EF349_(ST631).fna'**	**'F_(ST712).fna'**	76.8	[72.8 - 80.4]	90.9	[88.6 - 92.7]	82.0	[78.7 - 84.9]	1.03
**'EF349_(ST631).fna'**	**'EN788_(ST82).fna'**	74.9	[70.9 - 78.5]	90.9	[88.7 - 92.7]	80.3	[76.9 - 83.3]	1.7
**'4928STDY7071351_(ST30).fna'**	**'EF349_(ST631).fna'**	73.2	[69.2 - 76.8]	90.9	[88.6 - 92.7]	78.8	[75.3 - 81.9]	1.45
**'CVM_N54595_(ST192).fna'**	**'CVM_N60027F_(ST862).fna'**	79.4	[75.4 - 82.8]	90.9	[88.7 - 92.8]	84.2	[81.0 - 87.0]	0.15
**'15224_(ST16).fna'**	**'EF349_(ST631).fna'**	74.8	[70.8 - 78.4]	90.9	[88.7 - 92.7]	80.3	[76.9 - 83.3]	1.56
**'4928STDY7071435_(ST40).fna'**	**'BIOML_A5_(ST875).fna'**	75.6	[71.6 - 79.2]	90.9	[88.7 - 92.8]	81.0	[77.6 - 84.0]	0.39
**'CL9924_(ST6).fasta'**	**'E1_(ST40).fna'**	77.6	[73.7 - 81.2]	90.9	[88.7 - 92.7]	82.7	[79.4 - 85.6]	0.21
**'BT00.E.21_(ST23).fasta'**	**'EF349_(ST631).fna'**	75.9	[72.0 - 79.5]	90.9	[88.7 - 92.7]	81.3	[77.9 - 84.2]	1.53
**'CL9172_(ST21).fasta'**	**'CL9772_(ST525).fasta'**	78.7	[74.8 - 82.2]	90.9	[88.7 - 92.7]	83.7	[80.4 - 86.5]	0.24
**'CL9314_(ST21).fasta'**	**'F_(ST712).fna'**	76.8	[72.8 - 80.3]	90.9	[88.7 - 92.7]	82.0	[78.7 - 84.9]	0.18
**'CVM_N54595_(ST192).fna'**	**'T11_(ST65).fna'**	82.9	[79.1 - 86.2]	90.9	[88.6 - 92.7]	87.1	[84.1 - 89.7]	0.26
**'CL9772_(ST525).fasta'**	**'CVM_N60027F_(ST862).fna'**	76.0	[72.0 - 79.6]	90.9	[88.7 - 92.7]	81.3	[77.9 - 84.3]	0.36
**'CL9314_(ST21).fasta'**	**'CVM_N54595_(ST192).fna'**	78.0	[74.0 - 81.5]	90.9	[88.7 - 92.7]	83.0	[79.7 - 85.9]	0.02
**'732_EFLS_(ST16).fna'**	**'CL9797_(ST525).fasta'**	79.1	[75.2 - 82.6]	90.9	[88.7 - 92.7]	84.0	[80.8 - 86.8]	0.25
**'CL9172_(ST21).fasta'**	**'F_(ST712).fna'**	77.7	[73.7 - 81.2]	90.9	[88.7 - 92.7]	82.8	[79.5 - 85.7]	0.16

Query	Subject	d_0	C.I. d_0	d_4	C.I. d_4	d_6	C.I. d_6	Diff. G+C Percent
**'CL9797_(ST525).fasta'**	**'CL9943_(ST4).fasta'**	85.8	[82.2 - 88.8]	90.8	[88.5 - 92.6]	89.5	[86.6 - 91.7]	0.13
**'4928STDY7071351_(ST30).fna'**	**'15224_(ST16).fna'**	77.2	[73.2 - 80.7]	90.8	[88.6 - 92.6]	82.3	[79.0 - 85.2]	0.11
**'EF349_(ST631).fna'**	**'L14_(ST330).fna'**	85.9	[82.2 - 88.9]	90.8	[88.6 - 92.6]	89.5	[86.7 - 91.8]	1.02
**'CL9924_(ST6).fasta'**	**'LB00.E.122_(ST21).fasta'**	76.7	[72.7 - 80.3]	90.8	[88.6 - 92.6]	81.9	[78.6 - 84.8]	0.2
**'CL8682_(ST769).fasta'**	**'CL9924_(ST6).fasta'**	86.4	[82.8 - 89.3]	90.8	[88.6 - 92.7]	89.9	[87.1 - 92.1]	0.16
**'15224_(ST16).fna'**	**'EN788_(ST82).fna'**	77.9	[73.9 - 81.4]	90.8	[88.6 - 92.7]	82.9	[79.6 - 85.8]	0.14
**'19910_(ST116).fna'**	**'EF349_(ST631).fna'**	80.1	[76.1 - 83.5]	90.8	[88.5 - 92.6]	84.8	[81.6 - 87.5]	1.34
**'CL9943_(ST4).fasta'**	**'D32_(ST40).fna'**	70.9	[67.0 - 74.5]	90.8	[88.6 - 92.6]	76.7	[73.3 - 79.9]	0.44
**'CL9772_(ST525).fasta'**	**'CL9943_(ST4).fasta'**	83.0	[79.2 - 86.3]	90.8	[88.5 - 92.6]	87.2	[84.2 - 89.7]	0.21
**'244_EFLS_(ST443).fna'**	**'Merz96_(ST103).fna'**	73.9	[70.0 - 77.6]	90.8	[88.6 - 92.6]	79.5	[76.1 - 82.5]	0.11
**'BIOML_A5_(ST875).fna'**	**'Praia.S.M2.C4_(ST62).fasta'**	77.0	[73.0 - 80.6]	90.8	[88.6 - 92.7]	82.2	[78.8 - 85.1]	0.04
**'CL9943_(ST4).fasta'**	**'CL11199_(ST778).fasta'**	83.2	[79.4 - 86.5]	90.8	[88.6 - 92.6]	87.4	[84.4 - 89.9]	0.21
**'EF349_(ST631).fna'**	**'EN242_(ST82).fna'**	75.8	[71.9 - 79.4]	90.8	[88.6 - 92.6]	81.2	[77.8 - 84.1]	1.58
**'F_(ST712).fna'**	**'Praia.S.M2.C4_(ST62).fasta'**	76.9	[72.9 - 80.4]	90.8	[88.6 - 92.7]	82.1	[78.7 - 85.0]	0.23
**'CL9924_(ST6).fasta'**	**'EF348_(ST16).fna'**	78.1	[74.1 - 81.6]	90.8	[88.5 - 92.6]	83.1	[79.8 - 86.0]	0.39
**'20_SD_W_06_(ST368).fna'**	**'CL9772_(ST525).fasta'**	72.6	[68.6 - 76.2]	90.8	[88.5 - 92.6]	78.3	[74.8 - 81.4]	0.16
**'244_EFLS_(ST443).fna'**	**'CVM_N54595_(ST192).fna'**	77.1	[73.1 - 80.7]	90.8	[88.6 - 92.6]	82.3	[78.9 - 85.2]	0.09
**'19910_(ST116).fna'**	**'F1_(ST72).fna'**	77.9	[73.9 - 81.4]	90.8	[88.6 - 92.6]	82.9	[79.6 - 85.8]	0.21
**'BIOML_A5_(ST875).fna'**	**'KUB3007_(ST729).fna'**	77.3	[73.3 - 80.8]	90.8	[88.6 - 92.7]	82.4	[79.1 - 85.3]	0.16
**'EN788_(ST82).fna'**	**'L14_(ST330).fna'**	74.5	[70.5 - 78.1]	90.8	[88.6 - 92.6]	79.9	[76.5 - 83.0]	0.68
**'EnGen0400_(ST86).fna'**	**'L14_(ST330).fna'**	83.5	[79.7 - 86.7]	90.7	[88.4 - 92.5]	87.6	[84.6 - 90.1]	0.08
**'CL9172_(ST21).fasta'**	**'Merz96_(ST103).fna'**	79.0	[75.0 - 82.5]	90.7	[88.5 - 92.6]	83.8	[80.6 - 86.6]	0.17
**'CL9943_(ST4).fasta'**	**'KUB3007_(ST729).fna'**	74.1	[70.1 - 77.7]	90.7	[88.4 - 92.5]	79.6	[76.2 - 82.6]	0.19
**'EN242_(ST82).fna'**	**'Praia.S.M2.C4_(ST62).fasta'**	72.7	[68.8 - 76.4]	90.7	[88.5 - 92.6]	78.4	[75.0 - 81.5]	0.32
**'CL9924_(ST6).fasta'**	**'P9 CL A7_(ST4).fna'**	73.9	[69.9 - 77.6]	90.7	[88.5 - 92.5]	79.5	[76.0 - 82.5]	0.1
**'BIOML_A5_(ST875).fna'**	**'LB00.E.122_(ST21).fasta'**	78.2	[74.2 - 81.7]	90.7	[88.5 - 92.5]	83.2	[79.9 - 86.0]	0.01
**'15224_(ST16).fna'**	**'Praia.S.M2.C4_(ST62).fasta'**	75.0	[71.0 - 78.6]	90.7	[88.5 - 92.5]	80.4	[77.0 - 83.4]	0.3

Query	Subject	d_0	C.I. d_0	d_4	C.I. d_4	d_6	C.I. d_6	Diff. G+C Percent
**'EF348_(ST16).fna'**	**'L14_(ST330).fna'**	78.2	[74.2 - 81.7]	90.7	[88.5 - 92.6]	83.2	[79.9 - 86.0]	0.07
**'4928STDY7071435_(ST40).fna'**	**'L14_(ST330).fna'**	76.3	[72.3 - 79.8]	90.7	[88.4 - 92.5]	81.5	[78.1 - 84.4]	0.11
**'14EA1_(ST147).fna'**	**'Merz96_(ST103).fna'**	72.8	[68.8 - 76.4]	90.7	[88.5 - 92.6]	78.5	[75.0 - 81.5]	0.16
**'19910_(ST116).fna'**	**'CL9924_(ST6).fasta'**	71.9	[67.9 - 75.5]	90.7	[88.4 - 92.5]	77.6	[74.1 - 80.7]	0.14
**'CL8682_(ST769).fasta'**	**'CL9172_(ST21).fasta'**	79.1	[75.1 - 82.5]	90.7	[88.4 - 92.5]	83.9	[80.7 - 86.7]	0.12
**'L14_(ST330).fna'**	**'T11_(ST65).fna'**	86.4	[82.8 - 89.4]	90.7	[88.5 - 92.6]	89.9	[87.1 - 92.1]	0.05
**'CL9172_(ST21).fasta'**	**'CL9797_(ST525).fasta'**	78.6	[74.6 - 82.1]	90.7	[88.4 - 92.5]	83.5	[80.2 - 86.3]	0.32
**'302EA1_(ST122).fna'**	**'EF349_(ST631).fna'**	79.5	[75.5 - 83.0]	90.7	[88.4 - 92.5]	84.3	[81.0 - 87.1]	1.16
**'EF349_(ST631).fna'**	**'Praia.S.M2.C4_(ST62).fasta'**	78.5	[74.5 - 82.0]	90.7	[88.5 - 92.5]	83.4	[80.1 - 86.2]	1.26
**'244_EFLS_(ST443).fna'**	**'CL9772_(ST525).fasta'**	74.2	[70.2 - 77.8]	90.7	[88.5 - 92.6]	79.7	[76.3 - 82.7]	0.3
**'BIOML_A5_(ST875).fna'**	**'Merz96_(ST103).fna'**	76.5	[72.5 - 80.1]	90.7	[88.5 - 92.6]	81.7	[78.4 - 84.7]	0.27
**'EN788_(ST82).fna'**	**'Praia.S.M2.C4_(ST62).fasta'**	74.3	[70.3 - 77.9]	90.7	[88.5 - 92.5]	79.8	[76.4 - 82.8]	0.44
**'15224_(ST16).fna'**	**'CL9943_(ST4).fasta'**	78.2	[74.2 - 81.7]	90.7	[88.4 - 92.5]	83.2	[79.9 - 86.0]	0.09
**'EF349_(ST631).fna'**	**'P9 CL A7_(ST4).fna'**	78.4	[74.4 - 81.9]	90.7	[88.4 - 92.5]	83.3	[80.0 - 86.1]	1.59
**'CL6870_(ST103).fasta'**	**'EF349_(ST631).fna'**	78.8	[74.8 - 82.2]	90.7	[88.5 - 92.5]	83.7	[80.4 - 86.5]	1.36
**'15224_(ST16).fna'**	**'BT00.E.21_(ST23).fasta'**	75.1	[71.1 - 78.7]	90.7	[88.4 - 92.5]	80.5	[77.1 - 83.5]	0.03
**'302EA1_(ST122).fna'**	*Enterococcus faecalis* NBRC 100480	83.9	[80.1 - 87.0]	90.7	[88.4 - 92.5]	87.9	[84.9 - 90.3]	0.05
**'D32_(ST40).fna'**	*Enterococcus faecalis* NBRC 100480	81.5	[77.6 - 84.9]	90.7	[88.5 - 92.6]	86.0	[82.8 - 88.6]	0.09
**'EF349_(ST631).fna'**	**'KUB3007_(ST729).fna'**	78.4	[74.4 - 81.9]	90.7	[88.4 - 92.5]	83.3	[80.1 - 86.2]	1.46
**'CVM_N60027F_(ST862).fna'**	**'Merz96_(ST103).fna'**	77.8	[73.8 - 81.3]	90.6	[88.3 - 92.4]	82.8	[79.5 - 85.7]	0.05
**'CL8682_(ST769).fasta'**	**'EF349_(ST631).fna'**	78.5	[74.5 - 82.0]	90.6	[88.4 - 92.5]	83.4	[80.1 - 86.2]	1.32
**'CL9172_(ST21).fasta'**	**'L14_(ST330).fna'**	83.2	[79.4 - 86.4]	90.6	[88.3 - 92.4]	87.3	[84.3 - 89.8]	0.18
**'14EA1_(ST147).fna'**	**'L14_(ST330).fna'**	82.0	[78.1 - 85.3]	90.6	[88.3 - 92.5]	86.3	[83.2 - 88.9]	0.16
**'P9 CL A7_(ST4).fna'**	*Enterococcus faecalis* NBRC 100480	76.2	[72.2 - 79.7]	90.6	[88.3 - 92.4]	81.4	[78.0 - 84.4]	0.48
**'732_EFLS_(ST16).fna'**	**'CL11199_(ST778).fasta'**	74.8	[70.8 - 78.4]	90.6	[88.3 - 92.5]	80.2	[76.8 - 83.2]	0.17
**'CL6870_(ST103).fasta'**	**'CL9172_(ST21).fasta'**	79.7	[75.8 - 83.2]	90.6	[88.3 - 92.4]	84.4	[81.2 - 87.2]	0.16
**'LB00.E.122_(ST21).fasta'**	*Enterococcus faecalis* NBRC 100480	84.3	[80.5 - 87.4]	90.6	[88.4 - 92.5]	88.2	[85.3 - 90.6]	0.18

Query	Subject	d_0	C.I. d_0	d_4	C.I. d_4	d_6	C.I. d_6	Diff. G+C Percent
**'L14_(ST330).fna'**	**'P9 CL A7_(ST4).fna'**	76.2	[72.2 - 79.7]	90.6	[88.4 - 92.5]	81.4	[78.0 - 84.4]	0.57
**'CL9172_(ST21).fasta'**	**'CL11199_(ST778).fasta'**	75.4	[71.4 - 79.0]	90.6	[88.4 - 92.5]	80.8	[77.4 - 83.7]	0.24
**'19910_(ST116).fna'**	**'BIOML_A5_(ST875).fna'**	84.4	[80.7 - 87.6]	90.6	[88.4 - 92.5]	88.3	[85.4 - 90.7]	0.04
**'20_SD_W_06_(ST368).fna'**	**'EF349_(ST631).fna'**	82.5	[78.6 - 85.8]	90.5	[88.3 - 92.4]	86.8	[83.7 - 89.3]	1.28
**'CL6870_(ST103).fasta'**	**'CVM_N60027F_(ST862).fna'**	78.7	[74.8 - 82.2]	90.5	[88.2 - 92.3]	83.6	[80.3 - 86.4]	0.28
**'BT00.E.21_(ST23).fasta'**	**'Merz96_(ST103).fna'**	75.2	[71.2 - 78.8]	90.5	[88.2 - 92.4]	80.6	[77.2 - 83.6]	0.5
**'F_(ST712).fna'**	**'LB00.E.122_(ST21).fasta'**	77.1	[73.1 - 80.6]	90.5	[88.3 - 92.4]	82.2	[78.9 - 85.1]	0.26
**'244_EFLS_(ST443).fna'**	**'CL9797_(ST525).fasta'**	73.9	[70.0 - 77.6]	90.5	[88.2 - 92.3]	79.4	[76.0 - 82.5]	0.38
**'LB00.E.122_(ST21).fasta'**	**'Merz96_(ST103).fna'**	76.5	[72.5 - 80.1]	90.5	[88.2 - 92.3]	81.7	[78.3 - 84.6]	0.26
**'D32_(ST40).fna'**	**'EF349_(ST631).fna'**	75.6	[71.6 - 79.2]	90.5	[88.2 - 92.4]	80.9	[77.5 - 83.9]	1.2
**'D32_(ST40).fna'**	**'L14_(ST330).fna'**	80.7	[76.7 - 84.1]	90.5	[88.2 - 92.4]	85.2	[82.1 - 87.9]	0.18
**'BIOML_A5_(ST875).fna'**	**'F_(ST712).fna'**	76.6	[72.6 - 80.2]	90.5	[88.3 - 92.4]	81.8	[78.4 - 84.7]	0.27
**'D32_(ST40).fna'**	**'F1_(ST72).fna'**	77.2	[73.2 - 80.7]	90.5	[88.2 - 92.3]	82.2	[78.9 - 85.2]	0.07
**'CL8682_(ST769).fasta'**	**'CVM_N60027F_(ST862).fna'**	78.3	[74.4 - 81.8]	90.5	[88.2 - 92.3]	83.3	[80.0 - 86.1]	0.25
**'19910_(ST116).fna'**	**'KUB3007_(ST729).fna'**	81.6	[77.7 - 84.9]	90.5	[88.2 - 92.4]	86.0	[82.8 - 88.6]	0.12
**'20_SD_W_06_(ST368).fna'**	**'CL6870_(ST103).fasta'**	74.4	[70.4 - 78.0]	90.5	[88.3 - 92.4]	79.9	[76.4 - 82.9]	0.08
**'15224_(ST16).fna'**	**'D32_(ST40).fna'**	71.7	[67.7 - 75.3]	90.5	[88.2 - 92.3]	77.4	[73.9 - 80.5]	0.36
**'4928STDY7071435_(ST40).fna'**	**'CL9924_(ST6).fasta'**	75.8	[71.8 - 79.4]	90.5	[88.3 - 92.4]	81.1	[77.7 - 84.0]	0.58
**'244_EFLS_(ST443).fna'**	**'L14_(ST330).fna'**	81.3	[77.4 - 84.7]	90.5	[88.2 - 92.3]	85.8	[82.6 - 88.4]	0.11
**'EN242_(ST82).fna'**	**'L14_(ST330).fna'**	75.5	[71.5 - 79.1]	90.4	[88.1 - 92.3]	80.8	[77.4 - 83.8]	0.56
**'CL9314_(ST21).fasta'**	**'CL9772_(ST525).fasta'**	80.1	[76.1 - 83.5]	90.4	[88.1 - 92.3]	84.7	[81.5 - 87.5]	0.23
**'14EA1_(ST147).fna'**	**'EF349_(ST631).fna'**	81.2	[77.2 - 84.5]	90.4	[88.1 - 92.3]	85.6	[82.5 - 88.3]	0.87
**'732_EFLS_(ST16).fna'**	**'CL6870_(ST103).fasta'**	79.2	[75.3 - 82.7]	90.4	[88.1 - 92.3]	84.0	[80.7 - 86.8]	0.09
**'244_EFLS_(ST443).fna'**	**'CL6870_(ST103).fasta'**	75.2	[71.2 - 78.8]	90.4	[88.2 - 92.3]	80.6	[77.2 - 83.5]	0.22
**'244_EFLS_(ST443).fna'**	**'CL8682_(ST769).fasta'**	75.5	[71.5 - 79.1]	90.4	[88.1 - 92.3]	80.8	[77.4 - 83.8]	0.19
**'CL9943_(ST4).fasta'**	*Enterococcus faecalis* NBRC 100480	78.7	[74.7 - 82.2]	90.4	[88.2 - 92.3]	83.6	[80.3 - 86.4]	0.54
**'CVM_N54595_(ST192).fna'**	**'EF348_(ST16).fna'**	72.7	[68.7 - 76.4]	90.4	[88.1 - 92.3]	78.3	[74.9 - 81.4]	0.14

Query	Subject	d_0	C.I. d_0	d_4	C.I. d_4	d_6	C.I. d_6	Diff. G+C Percent
**'15224_(ST16).fna'**	*Enterococcus faecalis* NBRC 100480	77.0	[73.0 - 80.5]	90.4	[88.1 - 92.3]	82.1	[78.7 - 85.0]	0.45
**'4928STDY7071351_(ST30).fna'**	**'KUB3007_(ST729).fna'**	73.8	[69.8 - 77.4]	90.4	[88.1 - 92.3]	79.3	[75.9 - 82.3]	0.01
**'LB00.E.122_(ST21).fasta'**	**'Praia.S.M2.C4_(ST62).fasta'**	82.6	[78.8 - 85.9]	90.4	[88.1 - 92.3]	86.8	[83.8 - 89.4]	0.03
**'EN788_(ST82).fna'**	**'F_(ST712).fna'**	68.0	[64.1 - 71.6]	90.4	[88.2 - 92.3]	73.9	[70.5 - 77.1]	0.67
**'CVM_N54595_(ST192).fna'**	**'EF349_(ST631).fna'**	78.3	[74.3 - 81.8]	90.4	[88.1 - 92.2]	83.2	[79.9 - 86.1]	1.23
**'CL9772_(ST525).fasta'**	**'LB00.E.122_(ST21).fasta'**	77.1	[73.1 - 80.6]	90.4	[88.1 - 92.3]	82.2	[78.8 - 85.1]	0.15
**'19910_(ST116).fna'**	**'F_(ST712).fna'**	74.3	[70.4 - 78.0]	90.4	[88.2 - 92.3]	79.8	[76.4 - 82.8]	0.31
**'BIOML_A5_(ST875).fna'**	**'CL8682_(ST769).fasta'**	77.0	[73.1 - 80.6]	90.4	[88.1 - 92.2]	82.1	[78.8 - 85.1]	0.02
**'732_EFLS_(ST16).fna'**	**'Merz96_(ST103).fna'**	77.7	[73.8 - 81.3]	90.4	[88.2 - 92.3]	82.7	[79.4 - 85.6]	0.24
**'20_SD_W_06_(ST368).fna'**	**'F_(ST712).fna'**	75.7	[71.7 - 79.3]	90.4	[88.1 - 92.2]	81.0	[77.6 - 83.9]	0.25
**'CL9797_(ST525).fasta'**	**'LB00.E.122_(ST21).fasta'**	76.5	[72.5 - 80.1]	90.3	[88.0 - 92.2]	81.7	[78.3 - 84.6]	0.23
**'20_SD_W_06_(ST368).fna'**	**'L14_(ST330).fna'**	85.6	[81.9 - 88.6]	90.3	[88.0 - 92.2]	89.2	[86.4 - 91.5]	0.26
**'302EA1_(ST122).fna'**	**'4928STDY7071351_(ST30).fna'**	79.6	[75.6 - 83.0]	90.3	[88.1 - 92.2]	84.3	[81.0 - 87.0]	0.29
**'CL9797_(ST525).fasta'**	**'EnGen0400_(ST86).fna'**	76.4	[72.4 - 80.0]	90.3	[88.0 - 92.1]	81.6	[78.2 - 84.5]	0.57
**'20_SD_W_06_(ST368).fna'**	**'CL8682_(ST769).fasta'**	75.0	[71.0 - 78.6]	90.3	[88.0 - 92.2]	80.4	[77.0 - 83.4]	0.04
**'14EA1_(ST147).fna'**	**'F1_(ST72).fna'**	82.5	[78.7 - 85.8]	90.3	[88.1 - 92.2]	86.7	[83.7 - 89.3]	0.27
**'CL6870_(ST103).fasta'**	**'LB00.E.122_(ST21).fasta'**	77.5	[73.5 - 81.0]	90.3	[88.0 - 92.2]	82.5	[79.2 - 85.4]	0.07
**'14EA1_(ST147).fna'**	**'CVM_N54595_(ST192).fna'**	77.6	[73.6 - 81.1]	90.3	[88.0 - 92.2]	82.6	[79.3 - 85.5]	0.37
**'F1_(ST72).fna'**	**'LB00.E.122_(ST21).fasta'**	78.5	[74.5 - 82.0]	90.3	[88.0 - 92.2]	83.3	[80.1 - 86.2]	0.16
**'CVM_N60027F_(ST862).fna'**	**'F_(ST712).fna'**	79.9	[76.0 - 83.3]	90.3	[88.0 - 92.2]	84.6	[81.3 - 87.3]	0.04
**'BT00.E.21_(ST23).fasta'**	**'L14_(ST330).fna'**	74.8	[70.8 - 78.4]	90.3	[88.0 - 92.1]	80.1	[76.7 - 83.1]	0.51
**'BIOML_A5_(ST875).fna'**	**'CL6870_(ST103).fasta'**	76.8	[72.9 - 80.4]	90.3	[88.0 - 92.2]	81.9	[78.6 - 84.9]	0.06
**'CVM_N54595_(ST192).fna'**	**'LB00.E.122_(ST21).fasta'**	77.0	[73.0 - 80.6]	90.3	[88.0 - 92.2]	82.1	[78.7 - 85.0]	0.06
**'EF349_(ST631).fna'**	**'F1_(ST72).fna'**	78.1	[74.1 - 81.6]	90.3	[88.0 - 92.2]	83.0	[79.7 - 85.9]	1.13
**'CVM_N54595_(ST192).fna'**	**'E1_(ST40).fna'**	77.7	[73.7 - 81.2]	90.3	[88.0 - 92.2]	82.7	[79.4 - 85.6]	0.05
**'CL9314_(ST21).fasta'**	**'Merz96_(ST103).fna'**	80.1	[76.2 - 83.5]	90.3	[88.0 - 92.2]	84.7	[81.5 - 87.5]	0.18
**'CL8682_(ST769).fasta'**	**'LB00.E.122_(ST21).fasta'**	77.1	[73.1 - 80.6]	90.3	[88.0 - 92.2]	82.1	[78.8 - 85.1]	0.03

Query	Subject	d_0	C.I. d_0	d_4	C.I. d_4	d_6	C.I. d_6	Diff. G+C Percent
**'732_EFLS_(ST16).fna'**	**'CL8682_(ST769).fasta'**	78.4	[74.5 - 81.9]	90.2	[87.9 - 92.1]	83.3	[80.0 - 86.1]	0.06
**'CL9924_(ST6).fasta'**	**'KUB3007_(ST729).fna'**	74.1	[70.1 - 77.7]	90.2	[87.9 - 92.1]	79.5	[76.1 - 82.6]	0.03
**'CL9314_(ST21).fasta'**	**'CL9797_(ST525).fasta'**	79.9	[76.0 - 83.4]	90.2	[88.0 - 92.1]	84.6	[81.3 - 87.3]	0.31
**'EF348_(ST16).fna'**	**'F_(ST712).fna'**	72.5	[68.5 - 76.1]	90.2	[87.9 - 92.1]	78.1	[74.6 - 81.2]	0.06
**'F1_(ST72).fna'**	**'KUB3007_(ST729).fna'**	76.1	[72.1 - 79.6]	90.2	[88.0 - 92.1]	81.3	[77.9 - 84.2]	0.33
**'EF349_(ST631).fna'**	*Enterococcus faecalis* NBRC 100480	79.9	[75.9 - 83.3]	90.2	[87.9 - 92.1]	84.5	[81.3 - 87.3]	1.11
**'CL8682_(ST769).fasta'**	**'CL9314_(ST21).fasta'**	80.4	[76.4 - 83.8]	90.2	[87.9 - 92.1]	84.9	[81.7 - 87.6]	0.11
**'244_EFLS_(ST443).fna'**	**'CL11199_(ST778).fasta'**	72.7	[68.7 - 76.3]	90.2	[87.9 - 92.1]	78.3	[74.8 - 81.3]	0.3
**'CL9314_(ST21).fasta'**	**'CL11199_(ST778).fasta'**	76.6	[72.6 - 80.2]	90.2	[87.9 - 92.1]	81.7	[78.4 - 84.7]	0.23
**'CL9943_(ST4).fasta'**	**'EF349_(ST631).fna'**	71.7	[67.7 - 75.3]	90.2	[87.9 - 92.1]	77.3	[73.9 - 80.5]	1.65
**'CL9772_(ST525).fasta'**	**'EF348_(ST16).fna'**	76.0	[72.0 - 79.6]	90.1	[87.8 - 92.0]	81.2	[77.8 - 84.2]	0.34
**'4928STDY7071351_(ST30).fna'**	**'EN788_(ST82).fna'**	72.9	[69.0 - 76.6]	90.1	[87.8 - 92.0]	78.5	[75.0 - 81.5]	0.25
**'CVM_N54595_(ST192).fna'**	**'EnGen0400_(ST86).fna'**	82.3	[78.5 - 85.6]	90.1	[87.8 - 92.0]	86.6	[83.5 - 89.1]	0.29
**'4928STDY7071351_(ST30).fna'**	**'CL9772_(ST525).fasta'**	77.6	[73.6 - 81.1]	90.1	[87.8 - 92.0]	82.5	[79.2 - 85.4]	0.01
**'BT00.E.21_(ST23).fasta'**	**'CL9772_(ST525).fasta'**	76.7	[72.7 - 80.3]	90.1	[87.8 - 92.0]	81.8	[78.4 - 84.7]	0.09
**'L14_(ST330).fna'**	**'Praia.S.M2.C4_(ST62).fasta'**	78.3	[74.3 - 81.8]	90.1	[87.8 - 92.0]	83.2	[79.9 - 86.0]	0.24
**'BIOML_A5_(ST875).fna'**	**'CVM_N54595_(ST192).fna'**	79.7	[75.7 - 83.1]	90.1	[87.8 - 92.0]	84.3	[81.1 - 87.1]	0.07
**'4928STDY7071351_(ST30).fna'**	**'Merz96_(ST103).fna'**	78.3	[74.4 - 81.8]	90.1	[87.8 - 92.0]	83.2	[79.9 - 86.0]	0.42
**'302EA1_(ST122).fna'**	**'CL9943_(ST4).fasta'**	75.2	[71.2 - 78.8]	90.1	[87.8 - 92.0]	80.4	[77.0 - 83.4]	0.49
**'CL9924_(ST6).fasta'**	*Enterococcus faecalis* NBRC 100480	77.4	[73.4 - 80.9]	90.1	[87.8 - 92.0]	82.4	[79.0 - 85.3]	0.38
**'CL6870_(ST103).fasta'**	**'CL9314_(ST21).fasta'**	80.9	[77.0 - 84.3]	90.1	[87.8 - 92.0]	85.4	[82.2 - 88.1]	0.15
**'CVM_N54595_(ST192).fna'**	**'Merz96_(ST103).fna'**	76.0	[72.0 - 79.6]	90.0	[87.7 - 91.9]	81.2	[77.8 - 84.1]	0.2
**'20_SD_W_06_(ST368).fna'**	**'CVM_N54595_(ST192).fna'**	79.3	[75.4 - 82.8]	90.0	[87.7 - 92.0]	84.0	[80.8 - 86.8]	0.05
**'BT00.E.21_(ST23).fasta'**	**'CL9797_(ST525).fasta'**	75.5	[71.5 - 79.1]	90.0	[87.7 - 91.9]	80.7	[77.3 - 83.7]	0.01
**'Merz96_(ST103).fna'**	**'P9 CL A7_(ST4).fna'**	73.1	[69.2 - 76.8]	90.0	[87.7 - 92.0]	78.6	[75.2 - 81.7]	0.56
**'F_(ST712).fna'**	**'Merz96_(ST103).fna'**	71.4	[67.4 - 75.0]	90.0	[87.7 - 92.0]	77.1	[73.6 - 80.2]	0.01
**'CL11199_(ST778).fasta'**	**'EN788_(ST82).fna'**	70.4	[66.5 - 74.1]	90.0	[87.7 - 91.9]	76.2	[72.7 - 79.3]	0.26

Query	Subject	d_0	C.I. d_0	d_4	C.I. d_4	d_6	C.I. d_6	Diff. G+C Percent
**'EnGen0400_(ST86).fna'**	**'Merz96_(ST103).fna'**	78.7	[74.8 - 82.2]	90.0	[87.7 - 92.0]	83.5	[80.3 - 86.4]	0.08
**'CL9314_(ST21).fasta'**	**'L14_(ST330).fna'**	84.3	[80.5 - 87.5]	90.0	[87.7 - 91.9]	88.1	[85.2 - 90.6]	0.19
**'BIOML_A5_(ST875).fna'**	**'L14_(ST330).fna'**	84.2	[80.4 - 87.4]	90.0	[87.6 - 91.9]	88.0	[85.1 - 90.5]	0.28
**'F_(ST712).fna'**	**'P9 CL A7_(ST4).fna'**	70.7	[66.7 - 74.3]	90.0	[87.7 - 91.9]	76.4	[72.9 - 79.5]	0.56
**'F1_(ST72).fna'**	**'Merz96_(ST103).fna'**	73.2	[69.2 - 76.8]	90.0	[87.7 - 92.0]	78.7	[75.3 - 81.8]	0.11
**'302EA1_(ST122).fna'**	**'CL9924_(ST6).fasta'**	74.1	[70.1 - 77.7]	89.9	[87.6 - 91.8]	79.5	[76.1 - 82.5]	0.33
**'BT00.E.21_(ST23).fasta'**	**'CL8682_(ST769).fasta'**	77.7	[73.7 - 81.2]	89.9	[87.6 - 91.8]	82.6	[79.3 - 85.5]	0.21
**'302EA1_(ST122).fna'**	**'F_(ST712).fna'**	79.1	[75.1 - 82.5]	89.9	[87.6 - 91.9]	83.8	[80.5 - 86.6]	0.13
**'302EA1_(ST122).fna'**	**'L14_(ST330).fna'**	81.6	[77.7 - 84.9]	89.9	[87.6 - 91.8]	85.9	[82.8 - 88.5]	0.14
**'CL9924_(ST6).fasta'**	**'D32_(ST40).fna'**	71.0	[67.1 - 74.7]	89.9	[87.5 - 91.8]	76.7	[73.2 - 79.8]	0.28
**'14EA1_(ST147).fna'**	**'CL9772_(ST525).fasta'**	73.9	[69.9 - 77.5]	89.9	[87.5 - 91.8]	79.3	[75.9 - 82.3]	0.57
**'CL11199_(ST778).fasta'**	**'EN242_(ST82).fna'**	70.8	[66.8 - 74.4]	89.9	[87.5 - 91.8]	76.5	[73.0 - 79.6]	0.14
**'CL9943_(ST4).fasta'**	**'Merz96_(ST103).fna'**	78.0	[74.0 - 81.5]	89.9	[87.6 - 91.9]	82.9	[79.6 - 85.7]	0.62
**'302EA1_(ST122).fna'**	**'Merz96_(ST103).fna'**	73.8	[69.8 - 77.4]	89.9	[87.5 - 91.8]	79.2	[75.8 - 82.3]	0.13
**'D32_(ST40).fna'**	**'Merz96_(ST103).fna'**	69.8	[65.9 - 73.5]	89.9	[87.6 - 91.8]	75.6	[72.1 - 78.8]	0.18
**'CL9772_(ST525).fasta'**	**'E1_(ST40).fna'**	76.0	[72.0 - 79.6]	89.9	[87.5 - 91.8]	81.1	[77.8 - 84.1]	0.16
**'BT00.E.21_(ST23).fasta'**	**'CL6870_(ST103).fasta'**	77.4	[73.4 - 80.9]	89.9	[87.6 - 91.8]	82.3	[79.0 - 85.2]	0.17
**'CL9772_(ST525).fasta'**	**'EnGen0400_(ST86).fna'**	78.9	[75.0 - 82.4]	89.9	[87.6 - 91.8]	83.7	[80.4 - 86.5]	0.49
**'14EA1_(ST147).fna'**	**'CL9797_(ST525).fasta'**	73.1	[69.1 - 76.7]	89.9	[87.6 - 91.8]	78.6	[75.1 - 81.6]	0.65
**'Merz96_(ST103).fna'**	*Enterococcus faecalis* NBRC 100480	77.4	[73.4 - 80.9]	89.8	[87.5 - 91.7]	82.3	[79.0 - 85.2]	0.08
**'CVM_N54595_(ST192).fna'**	**'KUB3007_(ST729).fna'**	72.9	[68.9 - 76.5]	89.8	[87.4 - 91.7]	78.4	[74.9 - 81.5]	0.23
**'L14_(ST330).fna'**	*Enterococcus faecalis* NBRC 100480	82.7	[78.9 - 86.0]	89.8	[87.5 - 91.8]	86.8	[83.8 - 89.4]	0.09
**'CVM_N54595_(ST192).fna'**	**'P9 CL A7_(ST4).fna'**	72.1	[68.1 - 75.7]	89.8	[87.4 - 91.7]	77.6	[74.2 - 80.7]	0.36
**'F_(ST712).fna'**	**'KUB3007_(ST729).fna'**	72.4	[68.4 - 76.0]	89.8	[87.5 - 91.8]	77.9	[74.5 - 81.0]	0.43
**'BT00.E.21_(ST23).fasta'**	**'CL11199_(ST778).fasta'**	74.5	[70.5 - 78.1]	89.8	[87.5 - 91.7]	79.8	[76.4 - 82.9]	0.09
**'CL6870_(ST103).fasta'**	**'EnGen0400_(ST86).fna'**	80.0	[76.1 - 83.4]	89.8	[87.5 - 91.7]	84.6	[81.3 - 87.3]	0.41
**'CL9772_(ST525).fasta'**	**'P9 CL A7_(ST4).fna'**	75.2	[71.2 - 78.8]	89.8	[87.4 - 91.7]	80.4	[77.0 - 83.4]	0.15

Query	Subject	d_0	C.I. d_0	d_4	C.I. d_4	d_6	C.I. d_6	Diff. G+C Percent
**'EF348_(ST16).fna'**	**'Merz96_(ST103).fna'**	74.1	[70.1 - 77.7]	89.8	[87.5 - 91.7]	79.5	[76.0 - 82.5]	0.07
**'4928STDY7071351_(ST30).fna'**	**'CL9797_(ST525).fasta'**	77.5	[73.5 - 81.0]	89.8	[87.4 - 91.7]	82.4	[79.1 - 85.3]	0.07
**'4928STDY7071351_(ST30).fna'**	**'CL9924_(ST6).fasta'**	79.1	[75.2 - 82.6]	89.8	[87.4 - 91.7]	83.8	[80.6 - 86.6]	0.04
**'4928STDY7071351_(ST30).fna'**	**'CL8682_(ST769).fasta'**	77.5	[73.5 - 81.1]	89.8	[87.4 - 91.7]	82.4	[79.1 - 85.3]	0.12
**'4928STDY7071435_(ST40).fna'**	**'CL9772_(ST525).fasta'**	75.2	[71.2 - 78.8]	89.8	[87.4 - 91.7]	80.4	[77.0 - 83.4]	0.52
**'19910_(ST116).fna'**	**'L14_(ST330).fna'**	79.1	[75.1 - 82.6]	89.8	[87.4 - 91.7]	83.8	[80.5 - 86.6]	0.32
**'CL9797_(ST525).fasta'**	**'EF348_(ST16).fna'**	78.0	[74.0 - 81.5]	89.8	[87.4 - 91.7]	82.9	[79.6 - 85.7]	0.42
**'CL11199_(ST778).fasta'**	**'P9 CL A7_(ST4).fna'**	73.6	[69.7 - 77.3]	89.7	[87.3 - 91.6]	79.0	[75.6 - 82.1]	0.15
**'CVM_N54595_(ST192).fna'**	**'EN242_(ST82).fna'**	74.1	[70.2 - 77.8]	89.7	[87.3 - 91.6]	79.5	[76.1 - 82.5]	0.35
**'4928STDY7071435_(ST40).fna'**	**'CVM_N54595_(ST192).fna'**	77.0	[73.1 - 80.6]	89.7	[87.4 - 91.7]	82.0	[78.7 - 84.9]	0.32
**'19910_(ST116).fna'**	**'CL9797_(ST525).fasta'**	74.1	[70.1 - 77.7]	89.7	[87.4 - 91.7]	79.4	[76.0 - 82.5]	0.17
**'CL9924_(ST6).fasta'**	**'Praia.S.M2.C4_(ST62).fasta'**	75.5	[71.5 - 79.1]	89.7	[87.4 - 91.7]	80.7	[77.3 - 83.7]	0.22
**'F1_(ST72).fna'**	**'L14_(ST330).fna'**	79.5	[75.5 - 82.9]	89.7	[87.4 - 91.7]	84.1	[80.9 - 86.9]	0.11
**'14EA1_(ST147).fna'**	**'F_(ST712).fna'**	80.1	[76.2 - 83.5]	89.7	[87.3 - 91.6]	84.6	[81.4 - 87.4]	0.17
**'CL9943_(ST4).fasta'**	**'L14_(ST330).fna'**	72.6	[68.7 - 76.3]	89.7	[87.4 - 91.7]	78.1	[74.7 - 81.2]	0.62
**'E1_(ST40).fna'**	**'Merz96_(ST103).fna'**	75.8	[71.8 - 79.3]	89.7	[87.4 - 91.7]	80.9	[77.5 - 83.9]	0.25
**'14EA1_(ST147).fna'**	**'CL11199_(ST778).fasta'**	71.8	[67.8 - 75.4]	89.7	[87.4 - 91.7]	77.4	[73.9 - 80.5]	0.57
**'CL11199_(ST778).fasta'**	**'EnGen0400_(ST86).fna'**	76.4	[72.4 - 79.9]	89.7	[87.4 - 91.7]	81.4	[78.1 - 84.4]	0.49
**'L14_(ST330).fna'**	**'Merz96_(ST103).fna'**	77.9	[73.9 - 81.4]	89.7	[87.4 - 91.7]	82.8	[79.4 - 85.6]	0.0
**'CL9924_(ST6).fasta'**	**'F1_(ST72).fna'**	76.4	[72.5 - 80.0]	89.7	[87.4 - 91.7]	81.5	[78.2 - 84.5]	0.35
**'CL9797_(ST525).fasta'**	**'P9 CL A7_(ST4).fna'**	75.8	[71.8 - 79.4]	89.6	[87.2 - 91.5]	80.9	[77.6 - 83.9]	0.07
**'19910_(ST116).fna'**	**'CVM_N54595_(ST192).fna'**	77.1	[73.2 - 80.7]	89.6	[87.2 - 91.6]	82.1	[78.8 - 85.0]	0.11
**'CL9772_(ST525).fasta'**	**'F_(ST712).fna'**	72.7	[68.8 - 76.4]	89.6	[87.2 - 91.5]	78.2	[74.8 - 81.3]	0.4
**'14EA1_(ST147).fna'**	**'CL6870_(ST103).fasta'**	74.5	[70.5 - 78.1]	89.6	[87.2 - 91.6]	79.8	[76.4 - 82.8]	0.49
**'CL11199_(ST778).fasta'**	**'LB00.E.122_(ST21).fasta'**	74.3	[70.3 - 77.9]	89.6	[87.2 - 91.5]	79.6	[76.2 - 82.6]	0.15
**'15224_(ST16).fna'**	**'CVM_N54595_(ST192).fna'**	71.3	[67.3 - 74.9]	89.6	[87.2 - 91.5]	76.9	[73.4 - 80.0]	0.33
**'4928STDY7071351_(ST30).fna'**	**'F_(ST712).fna'**	75.2	[71.2 - 78.8]	89.6	[87.2 - 91.5]	80.4	[77.0 - 83.4]	0.41

Query	Subject	d_0	C.I. d_0	d_4	C.I. d_4	d_6	C.I. d_6	Diff. G+C Percent
**'F1_(ST72).fna'**	**'P9 CL A7_(ST4).fna'**	74.3	[70.4 - 78.0]	89.6	[87.3 - 91.6]	79.6	[76.2 - 82.7]	0.46
**'CL6870_(ST103).fasta'**	**'EF348_(ST16).fna'**	75.9	[72.0 - 79.5]	89.6	[87.2 - 91.5]	81.0	[77.7 - 84.0]	0.26
**'CL11199_(ST778).fasta'**	**'EF348_(ST16).fna'**	74.6	[70.6 - 78.2]	89.6	[87.2 - 91.6]	79.9	[76.5 - 82.9]	0.34
**'CL11199_(ST778).fasta'**	**'E1_(ST40).fna'**	73.1	[69.1 - 76.7]	89.6	[87.2 - 91.6]	78.5	[75.1 - 81.6]	0.16
**'L14_(ST330).fna'**	**'LB00.E.122_(ST21).fasta'**	81.8	[78.0 - 85.2]	89.6	[87.3 - 91.6]	86.1	[82.9 - 88.7]	0.27
**'CL8682_(ST769).fasta'**	**'E1_(ST40).fna'**	76.8	[72.8 - 80.4]	89.6	[87.3 - 91.6]	81.8	[78.5 - 84.7]	0.04
**'CL9797_(ST525).fasta'**	**'E1_(ST40).fna'**	76.1	[72.2 - 79.7]	89.6	[87.3 - 91.6]	81.2	[77.9 - 84.2]	0.24
**'CL6870_(ST103).fasta'**	**'P9 CL A7_(ST4).fna'**	75.4	[71.4 - 79.0]	89.6	[87.2 - 91.6]	80.6	[77.2 - 83.6]	0.23
**'CL8682_(ST769).fasta'**	**'D32_(ST40).fna'**	72.0	[68.0 - 75.7]	89.6	[87.2 - 91.5]	77.5	[74.1 - 80.7]	0.12
**'15224_(ST16).fna'**	**'CL9924_(ST6).fasta'**	75.8	[71.8 - 79.4]	89.6	[87.2 - 91.6]	80.9	[77.5 - 83.9]	0.07
**'4928STDY7071435_(ST40).fna'**	**'CL8682_(ST769).fasta'**	75.7	[71.7 - 79.3]	89.6	[87.2 - 91.5]	80.8	[77.4 - 83.8]	0.41
**'4928STDY7071351_(ST30).fna'**	**'CVM_N54595_(ST192).fna'**	75.1	[71.1 - 78.7]	89.6	[87.2 - 91.6]	80.3	[76.9 - 83.3]	0.22
**'4928STDY7071435_(ST40).fna'**	**'CL9797_(ST525).fasta'**	74.6	[70.7 - 78.3]	89.6	[87.3 - 91.6]	79.9	[76.5 - 82.9]	0.61
**'CL9797_(ST525).fasta'**	**'EN242_(ST82).fna'**	71.6	[67.6 - 75.2]	89.6	[87.2 - 91.5]	77.1	[73.7 - 80.3]	0.06
**'CVM_N54595_(ST192).fna'**	**'EN788_(ST82).fna'**	73.6	[69.6 - 77.3]	89.5	[87.1 - 91.5]	79.0	[75.6 - 82.1]	0.47
**'4928STDY7071351_(ST30).fna'**	**'BIOML_A5_(ST875).fna'**	76.0	[72.0 - 79.6]	89.5	[87.1 - 91.5]	81.1	[77.7 - 84.0]	0.15
**'CL9797_(ST525).fasta'**	**'EN788_(ST82).fna'**	71.3	[67.4 - 75.0]	89.5	[87.1 - 91.5]	76.9	[73.4 - 80.0]	0.19
**'CL8682_(ST769).fasta'**	**'EF348_(ST16).fna'**	74.8	[70.9 - 78.4]	89.5	[87.1 - 91.5]	80.1	[76.7 - 83.1]	0.23
**'CL9924_(ST6).fasta'**	**'L14_(ST330).fna'**	73.1	[69.1 - 76.7]	89.5	[87.2 - 91.5]	78.5	[75.1 - 81.6]	0.46
**'CVM_N54595_(ST192).fna'**	**'D32_(ST40).fna'**	74.1	[70.1 - 77.7]	89.5	[87.2 - 91.5]	79.4	[76.0 - 82.4]	0.03
**'KUB3007_(ST729).fna'**	**'L14_(ST330).fna'**	79.5	[75.5 - 82.9]	89.5	[87.1 - 91.4]	84.1	[80.8 - 86.9]	0.44
**'CL9943_(ST4).fasta'**	**'F1_(ST72).fna'**	77.8	[73.8 - 81.3]	89.5	[87.1 - 91.4]	82.6	[79.3 - 85.5]	0.51
**'CL9772_(ST525).fasta'**	*Enterococcus faecalis* NBRC 100480	79.1	[75.1 - 82.6]	89.5	[87.1 - 91.5]	83.8	[80.5 - 86.6]	0.33
**'CL9772_(ST525).fasta'**	**'EN788_(ST82).fna'**	72.3	[68.3 - 75.9]	89.5	[87.1 - 91.5]	77.8	[74.3 - 80.9]	0.27
**'CL6870_(ST103).fasta'**	**'L14_(ST330).fna'**	78.9	[75.0 - 82.4]	89.5	[87.1 - 91.4]	83.6	[80.3 - 86.4]	0.33
**'CL9772_(ST525).fasta'**	**'F1_(ST72).fna'**	75.0	[71.0 - 78.6]	89.4	[87.0 - 91.3]	80.2	[76.8 - 83.2]	0.3
**'EN242_(ST82).fna'**	**'Merz96_(ST103).fna'**	72.5	[68.5 - 76.1]	89.4	[87.0 - 91.4]	78.0	[74.5 - 81.1]	0.55

Query	Subject	d_0	C.I. d_0	d_4	C.I. d_4	d_6	C.I. d_6	Diff. G+C Percent
**'CL8682_(ST769).fasta'**	**'P9 CL A7_(ST4).fna'**	74.9	[70.9 - 78.5]	89.4	[87.0 - 91.4]	80.1	[76.7 - 83.1]	0.27
**'CL6870_(ST103).fasta'**	**'D32_(ST40).fna'**	72.5	[68.5 - 76.1]	89.4	[87.0 - 91.4]	78.0	[74.5 - 81.1]	0.15
**'14EA1_(ST147).fna'**	**'CL8682_(ST769).fasta'**	74.7	[70.7 - 78.3]	89.4	[87.1 - 91.4]	79.9	[76.5 - 83.0]	0.46
**'4928STDY7071351_(ST30).fna'**	**'CL6870_(ST103).fasta'**	78.0	[74.0 - 81.5]	89.4	[87.0 - 91.3]	82.8	[79.4 - 85.6]	0.09
**'CVM_N54595_(ST192).fna'**	**'L14_(ST330).fna'**	80.1	[76.1 - 83.5]	89.4	[87.0 - 91.3]	84.5	[81.3 - 87.3]	0.21
**'CL8682_(ST769).fasta'**	**'CL9943_(ST4).fasta'**	81.2	[77.3 - 84.6]	89.4	[87.0 - 91.4]	85.5	[82.4 - 88.2]	0.32
**'19910_(ST116).fna'**	**'CL11199_(ST778).fasta'**	72.6	[68.7 - 76.3]	89.4	[87.1 - 91.4]	78.1	[74.6 - 81.2]	0.1
**'CVM_N54595_(ST192).fna'**	**'F1_(ST72).fna'**	78.1	[74.2 - 81.7]	89.4	[87.0 - 91.4]	82.9	[79.6 - 85.8]	0.1
**'4928STDY7071435_(ST40).fna'**	**'Merz96_(ST103).fna'**	74.6	[70.7 - 78.3]	89.4	[87.0 - 91.4]	79.9	[76.5 - 82.9]	0.12
**'15224_(ST16).fna'**	**'BIOML_A5_(ST875).fna'**	77.1	[73.1 - 80.7]	89.4	[87.1 - 91.4]	82.0	[78.7 - 85.0]	0.26
**'4928STDY7071435_(ST40).fna'**	**'CL11199_(ST778).fasta'**	71.8	[67.8 - 75.4]	89.4	[87.0 - 91.4]	77.3	[73.8 - 80.4]	0.53
**'4928STDY7071435_(ST40).fna'**	**'CL6870_(ST103).fasta'**	75.3	[71.4 - 78.9]	89.4	[87.0 - 91.4]	80.5	[77.1 - 83.5]	0.44
**'CL8682_(ST769).fasta'**	**'EnGen0400_(ST86).fna'**	80.3	[76.4 - 83.7]	89.4	[87.0 - 91.4]	84.8	[81.5 - 87.5]	0.38
**'CL6870_(ST103).fasta'**	**'E1_(ST40).fna'**	77.1	[73.1 - 80.7]	89.4	[87.1 - 91.4]	82.0	[78.7 - 85.0]	0.08
**'CL9772_(ST525).fasta'**	**'Praia.S.M2.C4_(ST62).fasta'**	77.2	[73.2 - 80.7]	89.3	[86.9 - 91.3]	82.1	[78.7 - 85.0]	0.17
**'302EA1_(ST122).fna'**	**'CL9797_(ST525).fasta'**	72.6	[68.6 - 76.2]	89.3	[86.9 - 91.3]	78.0	[74.6 - 81.1]	0.35
**'19910_(ST116).fna'**	**'CL9772_(ST525).fasta'**	75.2	[71.3 - 78.8]	89.3	[87.0 - 91.3]	80.4	[77.0 - 83.4]	0.09
**'BT00.E.21_(ST23).fasta'**	**'CVM_N54595_(ST192).fna'**	72.5	[68.5 - 76.1]	89.3	[86.9 - 91.3]	77.9	[74.5 - 81.0]	0.3
**'CL9772_(ST525).fasta'**	**'EN242_(ST82).fna'**	73.4	[69.4 - 77.1]	89.3	[86.9 - 91.3]	78.8	[75.3 - 81.8]	0.14
**'F_(ST712).fna'**	**'L14_(ST330).fna'**	77.8	[73.8 - 81.3]	89.3	[87.0 - 91.3]	82.6	[79.3 - 85.5]	0.01
**'302EA1_(ST122).fna'**	**'CL11199_(ST778).fasta'**	72.2	[68.2 - 75.8]	89.2	[86.8 - 91.2]	77.6	[74.2 - 80.7]	0.28
**'EN788_(ST82).fna'**	**'Merz96_(ST103).fna'**	72.3	[68.3 - 75.9]	89.2	[86.8 - 91.2]	77.7	[74.3 - 80.8]	0.67
**'19910_(ST116).fna'**	**'Merz96_(ST103).fna'**	75.1	[71.1 - 78.7]	89.2	[86.8 - 91.2]	80.3	[76.9 - 83.3]	0.32
**'302EA1_(ST122).fna'**	**'CL9772_(ST525).fasta'**	73.2	[69.2 - 76.8]	89.2	[86.9 - 91.2]	78.6	[75.1 - 81.6]	0.27
**'15224_(ST16).fna'**	**'L14_(ST330).fna'**	76.3	[72.3 - 79.9]	89.2	[86.8 - 91.2]	81.3	[78.0 - 84.3]	0.54
**'CL9797_(ST525).fasta'**	**'F1_(ST72).fna'**	73.4	[69.4 - 77.0]	89.2	[86.8 - 91.2]	78.7	[75.3 - 81.8]	0.38
**'CL9797_(ST525).fasta'**	**'F_(ST712).fna'**	71.7	[67.7 - 75.3]	89.2	[86.8 - 91.2]	77.2	[73.7 - 80.3]	0.48

Query	Subject	d_0	C.I. d_0	d_4	C.I. d_4	d_6	C.I. d_6	Diff. G+C Percent
**'CL9797_(ST525).fasta'**	*Enterococcus faecalis* NBRC 100480	79.0	[75.1 - 82.5]	89.2	[86.8 - 91.2]	83.6	[80.4 - 86.5]	0.41
**'CL6870_(ST103).fasta'**	**'EN242_(ST82).fna'**	73.3	[69.3 - 76.9]	89.1	[86.7 - 91.1]	78.6	[75.1 - 81.7]	0.22
**'CVM_N54595_(ST192).fna'**	**'F_(ST712).fna'**	75.5	[71.6 - 79.1]	89.1	[86.7 - 91.1]	80.6	[77.2 - 83.6]	0.2
**'CL11199_(ST778).fasta'**	*Enterococcus faecalis* NBRC 100480	76.3	[72.3 - 79.9]	89.1	[86.7 - 91.1]	81.3	[77.9 - 84.2]	0.33
**'CL9797_(ST525).fasta'**	**'L14_(ST330).fna'**	76.2	[72.2 - 79.8]	89.1	[86.7 - 91.1]	81.2	[77.8 - 84.2]	0.49
**'CL8682_(ST769).fasta'**	**'EN242_(ST82).fna'**	72.7	[68.7 - 76.3]	89.1	[86.7 - 91.2]	78.1	[74.6 - 81.2]	0.26
**'CL11199_(ST778).fasta'**	**'F1_(ST72).fna'**	72.6	[68.6 - 76.2]	89.1	[86.7 - 91.1]	78.0	[74.5 - 81.1]	0.3
**'CL9797_(ST525).fasta'**	**'Praia.S.M2.C4_(ST62).fasta'**	76.9	[72.9 - 80.4]	89.1	[86.7 - 91.1]	81.8	[78.4 - 84.7]	0.25
**'15224_(ST16).fna'**	**'CL9797_(ST525).fasta'**	76.7	[72.7 - 80.3]	89.0	[86.6 - 91.1]	81.6	[78.3 - 84.6]	0.04
**'CL6870_(ST103).fasta'**	**'CVM_N54595_(ST192).fna'**	78.7	[74.7 - 82.2]	89.0	[86.5 - 91.0]	83.3	[80.0 - 86.1]	0.12
**'CL11199_(ST778).fasta'**	**'CVM_N54595_(ST192).fna'**	74.7	[70.7 - 78.3]	89.0	[86.6 - 91.1]	79.9	[76.5 - 82.9]	0.21
**'CL8682_(ST769).fasta'**	**'EN788_(ST82).fna'**	72.1	[68.2 - 75.8]	89.0	[86.5 - 91.0]	77.6	[74.1 - 80.7]	0.38
**'CL6870_(ST103).fasta'**	**'CL9943_(ST4).fasta'**	79.9	[75.9 - 83.3]	89.0	[86.6 - 91.0]	84.3	[81.1 - 87.1]	0.29
**'CL8682_(ST769).fasta'**	**'L14_(ST330).fna'**	78.9	[75.0 - 82.4]	89.0	[86.6 - 91.0]	83.5	[80.3 - 86.4]	0.3
**'CL9924_(ST6).fasta'**	**'F_(ST712).fna'**	70.4	[66.4 - 74.0]	89.0	[86.6 - 91.0]	76.0	[72.5 - 79.1]	0.45
**'CL9924_(ST6).fasta'**	**'CVM_N54595_(ST192).fna'**	75.8	[71.8 - 79.4]	89.0	[86.6 - 91.0]	80.8	[77.4 - 83.8]	0.26
**'KUB3007_(ST729).fna'**	**'Merz96_(ST103).fna'**	72.9	[69.0 - 76.6]	89.0	[86.6 - 91.0]	78.3	[74.9 - 81.4]	0.43
**'15224_(ST16).fna'**	**'F_(ST712).fna'**	69.6	[65.7 - 73.2]	89.0	[86.6 - 91.0]	75.2	[71.7 - 78.4]	0.53
**'Merz96_(ST103).fna'**	**'Praia.S.M2.C4_(ST62).fasta'**	76.2	[72.3 - 79.8]	89.0	[86.5 - 91.0]	81.2	[77.8 - 84.2]	0.24
**'CL8682_(ST769).fasta'**	**'F1_(ST72).fna'**	75.1	[71.1 - 78.7]	88.9	[86.5 - 90.9]	80.2	[76.8 - 83.2]	0.19
**'CVM_N54595_(ST192).fna'**	*Enterococcus faecalis* NBRC 100480	76.2	[72.2 - 79.8]	88.9	[86.5 - 90.9]	81.2	[77.8 - 84.2]	0.12
**'CL6870_(ST103).fasta'**	**'EN788_(ST82).fna'**	72.6	[68.7 - 76.3]	88.9	[86.4 - 90.9]	78.0	[74.5 - 81.1]	0.35
**'4928STDY7071351_(ST30).fna'**	**'CL11199_(ST778).fasta'**	74.8	[70.8 - 78.4]	88.9	[86.5 - 91.0]	79.9	[76.5 - 83.0]	0.01
**'CL8682_(ST769).fasta'**	**'F_(ST712).fna'**	73.3	[69.4 - 77.0]	88.9	[86.5 - 90.9]	78.6	[75.2 - 81.7]	0.29
**'CL6870_(ST103).fasta'**	**'F_(ST712).fna'**	73.4	[69.4 - 77.1]	88.9	[86.5 - 90.9]	78.7	[75.3 - 81.8]	0.32
**'CL8682_(ST769).fasta'**	**'Praia.S.M2.C4_(ST62).fasta'**	77.0	[73.0 - 80.5]	88.9	[86.5 - 90.9]	81.9	[78.5 - 84.8]	0.06
**'CL9797_(ST525).fasta'**	**'CVM_N54595_(ST192).fna'**	75.6	[71.6 - 79.1]	88.9	[86.5 - 90.9]	80.6	[77.2 - 83.6]	0.28

Query	Subject	d_0	C.I. d_0	d_4	C.I. d_4	d_6	C.I. d_6	Diff. G+C Percent
**'15224_(ST16).fna'**	**'F1_(ST72).fna'**	73.8	[69.8 - 77.5]	88.9	[86.5 - 91.0]	79.1	[75.6 - 82.1]	0.43
**'CL9772_(ST525).fasta'**	**'L14_(ST330).fna'**	77.6	[73.6 - 81.1]	88.9	[86.5 - 91.0]	82.4	[79.1 - 85.3]	0.41
**'19910_(ST116).fna'**	**'CL6870_(ST103).fasta'**	76.7	[72.7 - 80.2]	88.9	[86.4 - 90.9]	81.6	[78.2 - 84.5]	0.01
**'4928STDY7071351_(ST30).fna'**	**'L14_(ST330).fna'**	78.3	[74.3 - 81.8]	88.8	[86.3 - 90.8]	82.9	[79.6 - 85.8]	0.42
**'CL11199_(ST778).fasta'**	**'Praia.S.M2.C4_(ST62).fasta'**	74.8	[70.8 - 78.4]	88.8	[86.4 - 90.9]	79.9	[76.5 - 83.0]	0.17
**'CL8682_(ST769).fasta'**	**'CVM_N54595_(ST192).fna'**	78.3	[74.3 - 81.8]	88.8	[86.4 - 90.9]	83.0	[79.7 - 85.8]	0.09
**'CL9772_(ST525).fasta'**	**'CVM_N54595_(ST192).fna'**	76.8	[72.8 - 80.4]	88.8	[86.4 - 90.9]	81.7	[78.3 - 84.6]	0.2
**'CL11199_(ST778).fasta'**	**'L14_(ST330).fna'**	76.5	[72.6 - 80.1]	88.8	[86.4 - 90.9]	81.4	[78.1 - 84.4]	0.42
**'302EA1_(ST122).fna'**	**'CL6870_(ST103).fasta'**	75.7	[71.7 - 79.3]	88.8	[86.3 - 90.8]	80.7	[77.4 - 83.7]	0.19
**'CL6870_(ST103).fasta'**	**'KUB3007_(ST729).fna'**	78.1	[74.1 - 81.6]	88.8	[86.4 - 90.9]	82.8	[79.5 - 85.7]	0.1
**'CL11199_(ST778).fasta'**	**'KUB3007_(ST729).fna'**	74.2	[70.2 - 77.8]	88.8	[86.4 - 90.9]	79.4	[76.0 - 82.4]	0.02
**'19910_(ST116).fna'**	**'CL8682_(ST769).fasta'**	75.5	[71.6 - 79.1]	88.7	[86.3 - 90.8]	80.6	[77.2 - 83.6]	0.02
**'CL6870_(ST103).fasta'**	**'F1_(ST72).fna'**	75.6	[71.6 - 79.2]	88.7	[86.3 - 90.8]	80.6	[77.2 - 83.6]	0.22
**'15224_(ST16).fna'**	**'CL9772_(ST525).fasta'**	75.2	[71.2 - 78.8]	88.7	[86.3 - 90.8]	80.2	[76.8 - 83.2]	0.13
**'CL6870_(ST103).fasta'**	**'Praia.S.M2.C4_(ST62).fasta'**	77.4	[73.4 - 80.9]	88.7	[86.3 - 90.8]	82.2	[78.8 - 85.1]	0.09
**'CL8682_(ST769).fasta'**	*Enterococcus faecalis* NBRC 100480	79.8	[75.9 - 83.2]	88.7	[86.3 - 90.8]	84.2	[81.0 - 87.0]	0.21
**'302EA1_(ST122).fna'**	**'CL8682_(ST769).fasta'**	75.6	[71.6 - 79.2]	88.7	[86.2 - 90.7]	80.6	[77.2 - 83.6]	0.16
**'CL9772_(ST525).fasta'**	**'D32_(ST40).fna'**	72.0	[68.0 - 75.6]	88.7	[86.3 - 90.8]	77.4	[73.9 - 80.5]	0.23
**'CL9797_(ST525).fasta'**	**'D32_(ST40).fna'**	70.1	[66.2 - 73.7]	88.7	[86.3 - 90.8]	75.7	[72.2 - 78.8]	0.31
**'CL6870_(ST103).fasta'**	*Enterococcus faecalis* NBRC 100480	79.5	[75.5 - 82.9]	88.6	[86.1 - 90.7]	83.9	[80.7 - 86.7]	0.25
**'CVM_N54595_(ST192).fna'**	**'Praia.S.M2.C4_(ST62).fasta'**	74.6	[70.6 - 78.2]	88.6	[86.1 - 90.6]	79.7	[76.3 - 82.7]	0.03
**'CL9943_(ST4).fasta'**	**'CVM_N54595_(ST192).fna'**	73.6	[69.6 - 77.2]	88.6	[86.1 - 90.7]	78.8	[75.4 - 81.9]	0.42
**'CL9797_(ST525).fasta'**	**'KUB3007_(ST729).fna'**	75.8	[71.8 - 79.4]	88.6	[86.1 - 90.7]	80.8	[77.4 - 83.7]	0.06
**'CL9943_(ST4).fasta'**	**'F_(ST712).fna'**	73.2	[69.2 - 76.8]	88.6	[86.1 - 90.7]	78.5	[75.0 - 81.5]	0.61
**'D32_(ST40).fna'**	**'F_(ST712).fna'**	74.7	[70.8 - 78.4]	88.5	[86.0 - 90.5]	79.8	[76.4 - 82.8]	0.17
**'15224_(ST16).fna'**	**'Merz96_(ST103).fna'**	72.2	[68.3 - 75.9]	88.5	[86.1 - 90.6]	77.6	[74.1 - 80.7]	0.53
**'CL9772_(ST525).fasta'**	**'KUB3007_(ST729).fna'**	76.7	[72.7 - 80.3]	88.4	[85.9 - 90.5]	81.5	[78.1 - 84.5]	0.02

Query	Subject	d_0	C.I. d_0	d_4	C.I. d_4	d_6	C.I. d_6	Diff. G+C Percent
**'CL11199_(ST778).fasta'**	**'D32_(ST40).fna'**	72.8	[68.8 - 76.4]	88.3	[85.8 - 90.4]	78.1	[74.6 - 81.2]	0.24
**'CL8682_(ST769).fasta'**	**'KUB3007_(ST729).fna'**	77.1	[73.1 - 80.6]	88.3	[85.9 - 90.4]	81.9	[78.5 - 84.8]	0.14
**'15224_(ST16).fna'**	**'CL6870_(ST103).fasta'**	74.6	[70.6 - 78.2]	88.3	[85.8 - 90.4]	79.7	[76.3 - 82.7]	0.2
**'P9 CL A7_(ST4).fna'**	*Aequorivita lutea* q18	12.8	[10.1 - 16.0]	88.2	[85.7 - 90.3]	13.2	[10.9 - 16.0]	5.61
**'15224_(ST16).fna'**	**'CL8682_(ST769).fasta'**	73.6	[69.6 - 77.2]	88.0	[85.5 - 90.1]	78.7	[75.3 - 81.8]	0.24
**'15224_(ST16).fna'**	**'CL11199_(ST778).fasta'**	74.2	[70.3 - 77.9]	87.9	[85.3 - 90.0]	79.3	[75.8 - 82.3]	0.12
**'CL11199_(ST778).fasta'**	**'F_(ST712).fna'**	72.7	[68.8 - 76.4]	87.4	[84.8 - 89.6]	77.9	[74.4 - 81.0]	0.41
**'EF349_(ST631).fna'**	*Arthrobacter sulfonylureivorans* LAM7117	12.7	[10.0 - 16.0]	83.5	[80.7 - 85.9]	13.1	[10.8 - 15.9]	27.34
**'BT00.E.21_(ST23).fasta'**	*Aequorivita lutea* q18	12.8	[10.1 - 16.1]	83.0	[80.2 - 85.5]	13.2	[10.9 - 16.0]	5.55
**'T11_(ST65).fna'**	*Aequorivita lutea* q18	12.8	[10.1 - 16.1]	78.3	[75.4 - 81.0]	13.3	[10.9 - 16.0]	4.99
**'19910_(ST116).fna'**	*Aequorivita lutea* q18	12.8	[10.1 - 16.1]	78.2	[75.2 - 80.9]	13.2	[10.9 - 16.0]	5.37
**'P9 CL A7_(ST4).fna'**	*Amedibacillus hominis* NSJ-176	12.5	[9.9 - 15.8]	78.2	[75.2 - 80.9]	12.9	[10.6 - 15.7]	1.51
**'EN242_(ST82).fna'**	*Aequorivita lutea* q18	12.8	[10.1 - 16.1]	78.1	[75.1 - 80.8]	13.2	[10.9 - 16.0]	5.6
**'732_EFLS_(ST16).fna'**	*Aequorivita lutea* q18	12.8	[10.1 - 16.1]	78.1	[75.1 - 80.8]	13.2	[10.9 - 16.0]	5.29
**'CVM_N60027F_(ST862).fna'**	*Aequorivita lutea* q18	12.8	[10.1 - 16.1]	78.0	[75.1 - 80.7]	13.2	[10.9 - 16.0]	5.1
**'Praia.S.M2.C4_(ST62).fasta'**	*Aequorivita lutea* q18	12.8	[10.1 - 16.1]	77.9	[75.0 - 80.6]	13.3	[10.9 - 16.0]	5.29
**'209EA1_(ST624).fna'**	**'BT00.E.21_(ST23).fasta'**	71.8	[67.9 - 75.5]	77.6	[74.6 - 80.3]	75.4	[71.9 - 78.6]	0.63
**'209EA1_(ST624).fna'**	**'732_EFLS_(ST16).fna'**	80.5	[76.6 - 83.9]	77.6	[74.6 - 80.3]	82.9	[79.6 - 85.8]	0.37
**'BIOML_A5_(ST875).fna'**	*Aequorivita lutea* q18	12.8	[10.1 - 16.1]	77.5	[74.5 - 80.2]	13.2	[10.9 - 16.0]	5.32
**'EN788_(ST82).fna'**	*Aequorivita lutea* q18	12.8	[10.1 - 16.1]	77.5	[74.6 - 80.3]	13.2	[10.9 - 16.0]	5.72
**'732_EFLS_(ST16).fna'**	**'G81_(ST1468).fna'**	76.5	[72.6 - 80.1]	77.4	[74.5 - 80.2]	79.5	[76.1 - 82.6]	0.19
**'732_EFLS_(ST16).fna'**	**'DSM111623_(ST624).fna'**	78.4	[74.4 - 81.9]	77.4	[74.4 - 80.1]	81.1	[77.7 - 84.0]	0.11
**'BT00.E.21_(ST23).fasta'**	**'DSM111623_(ST624).fna'**	73.0	[69.0 - 76.6]	77.4	[74.4 - 80.1]	76.4	[72.9 - 79.6]	0.37
**'209EA1_(ST624).fna'**	**'L14_(ST330).fna'**	82.1	[78.2 - 85.4]	77.4	[74.4 - 80.1]	84.2	[81.0 - 87.0]	0.13
**'209EA1_(ST624).fna'**	**'F1_(ST72).fna'**	75.7	[71.7 - 79.3]	77.4	[74.4 - 80.1]	78.7	[75.3 - 81.8]	0.24
**'BT00.E.21_(ST23).fasta'**	**'C138_(ST228).fna'**	70.7	[66.8 - 74.4]	77.3	[74.4 - 80.1]	74.3	[70.9 - 77.5]	0.5
**'14EA1_(ST147).fna'**	**'C138_(ST228).fna'**	82.9	[79.0 - 86.1]	77.3	[74.4 - 80.1]	84.9	[81.7 - 87.6]	0.17

Query	Subject	d_0	C.I. d_0	d_4	C.I. d_4	d_6	C.I. d_6	Diff. G+C Percent
**'14EA1_(ST147).fna'**	**'209EA1_(ST624).fna'**	80.3	[76.4 - 83.7]	77.3	[74.3 - 80.0]	82.7	[79.4 - 85.6]	0.03
**'209EA1_(ST624).fna'**	**'D32_(ST40).fna'**	76.1	[72.1 - 79.7]	77.3	[74.3 - 80.0]	79.1	[75.7 - 82.2]	0.31
**'CL9314_(ST21).fasta'**	**'G81_(ST1468).fna'**	73.7	[69.7 - 77.3]	77.3	[74.3 - 80.0]	77.0	[73.5 - 80.1]	0.13
**'209EA1_(ST624).fna'**	**'244_EFLS_(ST443).fna'**	79.8	[75.8 - 83.2]	77.3	[74.3 - 80.0]	82.2	[78.9 - 85.1]	0.24
**'244_EFLS_(ST443).fna'**	*Aequorivita lutea* q18	12.8	[10.1 - 16.1]	77.2	[74.2 - 80.0]	13.2	[10.9 - 16.0]	5.16
**'CL9172_(ST21).fasta'**	**'G81_(ST1468).fna'**	74.3	[70.3 - 77.9]	77.2	[74.2 - 79.9]	77.5	[74.0 - 80.6]	0.12
**'209EA1_(ST624).fna'**	**'302EA1_(ST122).fna'**	78.5	[74.6 - 82.0]	77.2	[74.2 - 79.9]	81.2	[77.8 - 84.1]	0.26
**'209EA1_(ST624).fna'**	**'CL9314_(ST21).fasta'**	79.4	[75.4 - 82.8]	77.2	[74.2 - 79.9]	81.9	[78.5 - 84.8]	0.31
**'CL9943_(ST4).fasta'**	*Aequorivita lutea* q18	12.8	[10.1 - 16.1]	77.2	[74.2 - 80.0]	13.2	[10.9 - 16.0]	5.67
**'CL9824_(ST631).fasta'**	*Aequorivita lutea* q18	12.8	[10.1 - 16.1]	77.2	[74.3 - 80.0]	13.2	[10.9 - 16.0]	4.94
**'732_EFLS_(ST16).fna'**	**'CVM N52662_(ST228).fna'**	77.9	[74.0 - 81.5]	77.2	[74.2 - 79.9]	80.7	[77.3 - 83.7]	0.16
**'209EA1_(ST624).fna'**	**'CL9943_(ST4).fasta'**	68.8	[64.9 - 72.5]	77.2	[74.2 - 79.9]	72.6	[69.2 - 75.8]	0.75
**'732_EFLS_(ST16).fna'**	**'CVM_N52587_(STx).fna'**	76.1	[72.1 - 79.7]	77.2	[74.2 - 80.0]	79.1	[75.6 - 82.1]	0.13
**'209EA1_(ST624).fna'**	**'EnGen0400_(ST86).fna'**	80.3	[76.3 - 83.7]	77.2	[74.2 - 79.9]	82.6	[79.3 - 85.5]	0.05
**'732_EFLS_(ST16).fna'**	**'CVM_N55265_(ST228).fna'**	77.2	[73.2 - 80.7]	77.2	[74.2 - 80.0]	80.0	[76.6 - 83.0]	0.22
**'209EA1_(ST624).fna'**	**'CL9824_(ST631).fasta'**	88.3	[84.9 - 91.1]	77.2	[74.2 - 79.9]	89.3	[86.4 - 91.6]	0.02
**'E1_(ST40).fna'**	**'G81_(ST1468).fna'**	73.9	[69.9 - 77.5]	77.2	[74.2 - 79.9]	77.1	[73.7 - 80.3]	0.2
**'244_EFLS_(ST443).fna'**	**'DSM111623_(ST624).fna'**	78.1	[74.1 - 81.6]	77.1	[74.1 - 79.8]	80.8	[77.4 - 83.7]	0.02
**'732_EFLS_(ST16).fna'**	**'R48_(ST228).fna'**	78.1	[74.1 - 81.6]	77.1	[74.1 - 79.9]	80.8	[77.4 - 83.8]	0.2
**'CL9772_(ST525).fasta'**	*Aequorivita lutea* q18	12.8	[10.1 - 16.1]	77.1	[74.1 - 79.9]	13.2	[10.9 - 16.0]	5.46
**'4928STDY7071351_(ST30).fna'**	*Aequorivita lutea* q18	12.8	[10.1 - 16.1]	77.1	[74.1 - 79.9]	13.2	[10.9 - 16.0]	5.47
**'BT00.E.21_(ST23).fasta'**	**'C116_(ST228).fna'**	70.6	[66.6 - 74.2]	77.1	[74.1 - 79.8]	74.2	[70.7 - 77.4]	0.48
**'209EA1_(ST624).fna'**	**'LB00.E.122_(ST21).fasta'**	77.4	[73.4 - 81.0]	77.1	[74.1 - 79.8]	80.2	[76.8 - 83.2]	0.39
**'14EA1_(ST147).fna'**	**'DSM111623_(ST624).fna'**	78.0	[74.1 - 81.6]	77.1	[74.1 - 79.9]	80.7	[77.4 - 83.7]	0.3
**'CL9824_(ST631).fasta'**	**'G81_(ST1468).fna'**	82.3	[78.4 - 85.6]	77.1	[74.1 - 79.8]	84.3	[81.1 - 87.1]	0.16
**'DSM111623_(ST624).fna'**	**'P9 CL A7_(ST4).fna'**	71.0	[67.1 - 74.7]	77.1	[74.1 - 79.9]	74.6	[71.1 - 77.8]	0.43
**'CL9314_(ST21).fasta'**	**'CVM N52662_(ST228).fna'**	75.1	[71.1 - 78.7]	77.1	[74.1 - 79.9]	78.2	[74.7 - 81.3]	0.11

Query	Subject	d_0	C.I. d_0	d_4	C.I. d_4	d_6	C.I. d_6	Diff. G+C Percent
**'732_EFLS_(ST16).fna'**	**'EN24_(ST228).fna'**	77.1	[73.1 - 80.7]	77.1	[74.1 - 79.8]	79.9	[76.5 - 83.0]	0.17
**'209EA1_(ST624).fna'**	**'P9 CL A7_(ST4).fna'**	71.9	[68.0 - 75.6]	77.1	[74.2 - 79.9]	75.4	[71.9 - 78.6]	0.69
**'732_EFLS_(ST16).fna'**	**'C138_(ST228).fna'**	77.6	[73.6 - 81.2]	77.1	[74.2 - 79.9]	80.4	[77.0 - 83.4]	0.23
**'209EA1_(ST624).fna'**	**'15224_(ST16).fna'**	69.4	[65.5 - 73.0]	77.1	[74.1 - 79.8]	73.1	[69.6 - 76.3]	0.66
**'209EA1_(ST624).fna'**	**'CL9172_(ST21).fasta'**	80.1	[76.1 - 83.5]	77.1	[74.1 - 79.9]	82.5	[79.1 - 85.4]	0.3
**'209EA1_(ST624).fna'**	**'E1_(ST40).fna'**	75.3	[71.3 - 78.9]	77.1	[74.1 - 79.8]	78.3	[74.9 - 81.4]	0.38
**'209EA1_(ST624).fna'**	**'EF348_(ST16).fna'**	76.4	[72.4 - 80.0]	77.1	[74.1 - 79.8]	79.3	[75.9 - 82.4]	0.2
**'732_EFLS_(ST16).fna'**	**'CVM N53420_(ST228).fna'**	76.1	[72.1 - 79.7]	77.1	[74.2 - 79.9]	79.1	[75.7 - 82.1]	0.08
**'CL9314_(ST21).fasta'**	**'CVM_N52587_(STx).fna'**	74.7	[70.7 - 78.3]	77.0	[74.0 - 79.7]	77.8	[74.4 - 80.9]	0.07
**'209EA1_(ST624).fna'**	**'T11_(ST65).fna'**	85.1	[81.4 - 88.2]	77.0	[74.0 - 79.8]	86.6	[83.6 - 89.2]	0.07
**'CL9314_(ST21).fasta'**	**'CVM_N55265_(ST228).fna'**	76.0	[72.0 - 79.6]	77.0	[74.0 - 79.7]	79.0	[75.5 - 82.0]	0.17
**'209EA1_(ST624).fna'**	**'BIOML_A5_(ST875).fna'**	79.5	[75.6 - 83.0]	77.0	[74.0 - 79.7]	82.0	[78.7 - 84.9]	0.4
**'209EA1_(ST624).fna'**	**'CL11199_(ST778).fasta'**	70.1	[66.2 - 73.8]	77.0	[74.0 - 79.7]	73.7	[70.3 - 76.9]	0.54
**'BT00.E.21_(ST23).fasta'**	**'CVM_N55265_(ST228).fna'**	72.8	[68.8 - 76.4]	77.0	[74.0 - 79.7]	76.2	[72.7 - 79.3]	0.49
**'209EA1_(ST624).fna'**	**'CL9924_(ST6).fasta'**	69.4	[65.5 - 73.0]	77.0	[74.0 - 79.7]	73.1	[69.6 - 76.3]	0.59
**'CL9943_(ST4).fasta'**	**'DSM111623_(ST624).fna'**	67.2	[63.4 - 70.9]	77.0	[74.0 - 79.8]	71.1	[67.7 - 74.4]	0.49
**'CVM N53420_(ST228).fna'**	**'CVM_N54595_(ST192).fna'**	79.8	[75.9 - 83.3]	77.0	[74.1 - 79.8]	82.3	[78.9 - 85.2]	0.04
**'732_EFLS_(ST16).fna'**	**'C116_(ST228).fna'**	78.3	[74.3 - 81.8]	77.0	[74.1 - 79.8]	80.9	[77.6 - 83.9]	0.22
**'CVM N52662_(ST228).fna'**	**'LB00.E.122_(ST21).fasta'**	73.4	[69.4 - 77.0]	77.0	[74.0 - 79.7]	76.6	[73.2 - 79.8]	0.19
**'CL9172_(ST21).fasta'**	**'CVM N52662_(ST228).fna'**	75.8	[71.8 - 79.4]	77.0	[74.0 - 79.8]	78.8	[75.3 - 81.8]	0.09
**'C116_(ST228).fna'**	**'CL9314_(ST21).fasta'**	75.7	[71.7 - 79.3]	77.0	[74.0 - 79.7]	78.7	[75.2 - 81.8]	0.16
**'C138_(ST228).fna'**	**'LB00.E.122_(ST21).fasta'**	74.5	[70.5 - 78.1]	77.0	[74.0 - 79.7]	77.7	[74.2 - 80.8]	0.26
**'209EA1_(ST624).fna'**	**'EN788_(ST82).fna'**	72.2	[68.2 - 75.8]	77.0	[74.0 - 79.7]	75.6	[72.1 - 78.8]	0.8
**'209EA1_(ST624).fna'**	**'EN242_(ST82).fna'**	72.9	[69.0 - 76.6]	77.0	[74.0 - 79.8]	76.3	[72.8 - 79.4]	0.68
**'209EA1_(ST624).fna'**	**'Merz96_(ST103).fna'**	74.5	[70.5 - 78.1]	77.0	[74.0 - 79.7]	77.7	[74.2 - 80.8]	0.13
**'CL9172_(ST21).fasta'**	**'CVM_N55265_(ST228).fna'**	76.7	[72.7 - 80.3]	76.9	[73.9 - 79.6]	79.5	[76.1 - 82.6]	0.16
**'209EA1_(ST624).fna'**	**'KUB3007_(ST729).fna'**	74.2	[70.2 - 77.8]	76.9	[73.9 - 79.7]	77.4	[73.9 - 80.5]	0.56

Query	Subject	d_0	C.I. d_0	d_4	C.I. d_4	d_6	C.I. d_6	Diff. G+C Percent
**'244_EFLS_(ST443).fna'**	**'G81_(ST1468).fna'**	74.9	[70.9 - 78.5]	76.9	[73.9 - 79.7]	78.0	[74.5 - 81.1]	0.06
**'209EA1_(ST624).fna'**	**'Praia.S.M2.C4_(ST62).fasta'**	78.4	[74.5 - 81.9]	76.9	[73.9 - 79.7]	81.1	[77.7 - 84.0]	0.37
**'DSM111623_(ST624).fna'**	**'EN242_(ST82).fna'**	71.1	[67.1 - 74.7]	76.9	[73.9 - 79.6]	74.6	[71.1 - 77.8]	0.42
**'CVM_N52587_(STx).fna'**	**'EF348_(ST16).fna'**	72.3	[68.3 - 76.0]	76.9	[73.9 - 79.6]	75.7	[72.2 - 78.9]	0.04
**'CL9924_(ST6).fasta'**	**'DSM111623_(ST624).fna'**	67.7	[63.8 - 71.3]	76.9	[73.9 - 79.6]	71.5	[68.1 - 74.7]	0.33
**'CL8682_(ST769).fasta'**	*Aequorivita lutea* q18	12.8	[10.1 - 16.1]	76.9	[73.9 - 79.6]	13.2	[10.9 - 16.0]	5.35
**'CL9314_(ST21).fasta'**	*Aequorivita lutea* q18	12.8	[10.1 - 16.1]	76.9	[73.9 - 79.6]	13.2	[10.9 - 16.0]	5.23
**'CVM_N52587_(STx).fna'**	**'LB00.E.122_(ST21).fasta'**	73.0	[69.0 - 76.6]	76.9	[73.9 - 79.6]	76.3	[72.8 - 79.4]	0.15
**'Merz96_(ST103).fna'**	*Aequorivita lutea* q18	12.8	[10.1 - 16.1]	76.9	[73.9 - 79.6]	13.2	[10.9 - 16.0]	5.05
**'EN24_(ST228).fna'**	**'P9 CL A7_(ST4).fna'**	73.5	[69.5 - 77.1]	76.9	[73.9 - 79.7]	76.7	[73.3 - 79.9]	0.5
**'C138_(ST228).fna'**	**'CL9314_(ST21).fasta'**	76.5	[72.5 - 80.1]	76.9	[73.9 - 79.7]	79.4	[76.0 - 82.4]	0.18
**'209EA1_(ST624).fna'**	*Enterococcus faecalis* NBRC 100480	81.5	[77.6 - 84.8]	76.9	[73.9 - 79.6]	83.6	[80.3 - 86.4]	0.21
**'G81_(ST1468).fna'**	**'LB00.E.122_(ST21).fasta'**	72.3	[68.4 - 76.0]	76.9	[73.9 - 79.7]	75.7	[72.2 - 78.9]	0.21
**'LB00.E.122_(ST21).fasta'**	*Aequorivita lutea* q18	12.8	[10.1 - 16.1]	76.9	[73.9 - 79.6]	13.2	[10.9 - 16.0]	5.31
**'CL9172_(ST21).fasta'**	*Aequorivita lutea* q18	12.8	[10.1 - 16.1]	76.9	[74.0 - 79.7]	13.2	[10.9 - 16.0]	5.22
**'CL9172_(ST21).fasta'**	**'CVM_N52587_(STx).fna'**	75.4	[71.4 - 79.0]	76.9	[73.9 - 79.6]	78.4	[75.0 - 81.5]	0.06
**'EF348_(ST16).fna'**	**'G81_(ST1468).fna'**	72.9	[68.9 - 76.5]	76.9	[73.9 - 79.7]	76.2	[72.8 - 79.4]	0.01
**'CL9824_(ST631).fasta'**	**'CVM_N52587_(STx).fna'**	83.6	[79.8 - 86.8]	76.9	[73.9 - 79.7]	85.3	[82.2 - 88.0]	0.22
**'CL11199_(ST778).fasta'**	**'DSM111623_(ST624).fna'**	67.6	[63.7 - 71.2]	76.9	[73.9 - 79.7]	71.4	[67.9 - 74.6]	0.28
**'209EA1_(ST624).fna'**	**'EF349_(ST631).fna'**	82.5	[78.6 - 85.8]	76.9	[73.9 - 79.6]	84.4	[81.2 - 87.2]	0.9
**'209EA1_(ST624).fna'**	**'CVM_N54595_(ST192).fna'**	75.9	[71.9 - 79.5]	76.8	[73.8 - 79.6]	78.8	[75.4 - 81.9]	0.33
**'DSM111623_(ST624).fna'**	**'Praia.S.M2.C4_(ST62).fasta'**	75.8	[71.9 - 79.4]	76.8	[73.8 - 79.6]	78.8	[75.4 - 81.9]	0.1
**'C138_(ST228).fna'**	**'D32_(ST40).fna'**	73.1	[69.1 - 76.7]	76.8	[73.8 - 79.5]	76.4	[72.9 - 79.5]	0.17
**'CVM N52662_(ST228).fna'**	**'EF348_(ST16).fna'**	74.0	[70.1 - 77.7]	76.8	[73.8 - 79.5]	77.2	[73.7 - 80.3]	0.01
**'CL9772_(ST525).fasta'**	**'DSM111623_(ST624).fna'**	69.7	[65.7 - 73.3]	76.8	[73.8 - 79.5]	73.3	[69.8 - 76.5]	0.28
**'CVM_N55265_(ST228).fna'**	**'E1_(ST40).fna'**	71.6	[67.6 - 75.2]	76.8	[73.8 - 79.5]	75.0	[71.6 - 78.2]	0.24
**'EN242_(ST82).fna'**	**'R48_(ST228).fna'**	69.9	[65.9 - 73.5]	76.8	[73.8 - 79.5]	73.5	[70.0 - 76.7]	0.51

Query	Subject	d_0	C.I. d_0	d_4	C.I. d_4	d_6	C.I. d_6	Diff. G+C Percent
**'209EA1_(ST624).fna'**	**'CL9772_(ST525).fasta'**	72.0	[68.1 - 75.7]	76.8	[73.8 - 79.5]	75.4	[72.0 - 78.6]	0.54
**'C116_(ST228).fna'**	**'CL9824_(ST631).fasta'**	84.7	[80.9 - 87.8]	76.8	[73.8 - 79.5]	86.2	[83.1 - 88.9]	0.12
**'BT00.E.21_(ST23).fasta'**	**'R48_(ST228).fna'**	70.8	[66.8 - 74.4]	76.8	[73.8 - 79.6]	74.3	[70.8 - 77.5]	0.46
**'15224_(ST16).fna'**	**'DSM111623_(ST624).fna'**	67.6	[63.7 - 71.2]	76.8	[73.8 - 79.6]	71.4	[67.9 - 74.6]	0.4
**'C116_(ST228).fna'**	**'E1_(ST40).fna'**	72.6	[68.7 - 76.3]	76.8	[73.8 - 79.6]	76.0	[72.5 - 79.1]	0.23
**'CL9824_(ST631).fasta'**	**'CVM N52662_(ST228).fna'**	82.7	[78.9 - 86.0]	76.8	[73.8 - 79.5]	84.6	[81.4 - 87.4]	0.18
**'CL9824_(ST631).fasta'**	**'DSM111623_(ST624).fna'**	86.3	[82.7 - 89.3]	76.8	[73.8 - 79.6]	87.6	[84.6 - 90.1]	0.24
**'CL9314_(ST21).fasta'**	**'R48_(ST228).fna'**	76.6	[72.6 - 80.1]	76.8	[73.8 - 79.5]	79.4	[76.0 - 82.5]	0.14
**'C138_(ST228).fna'**	**'CL9824_(ST631).fasta'**	85.4	[81.7 - 88.4]	76.8	[73.8 - 79.5]	86.8	[83.8 - 89.4]	0.11
**'CVM_N60027F_(ST862).fna'**	**'EN24_(ST228).fna'**	83.7	[79.9 - 86.9]	76.8	[73.9 - 79.6]	85.4	[82.3 - 88.1]	0.02
**'CL9824_(ST631).fasta'**	**'R48_(ST228).fna'**	82.8	[78.9 - 86.1]	76.8	[73.9 - 79.6]	84.7	[81.5 - 87.4]	0.15
**'CVM N52662_(ST228).fna'**	**'E1_(ST40).fna'**	73.3	[69.3 - 76.9]	76.8	[73.8 - 79.5]	76.5	[73.1 - 79.7]	0.18
**'14EA1_(ST147).fna'**	**'G81_(ST1468).fna'**	76.0	[72.0 - 79.6]	76.8	[73.8 - 79.5]	78.9	[75.5 - 82.0]	0.22
**'DSM111623_(ST624).fna'**	**'F_(ST712).fna'**	72.2	[68.3 - 75.9]	76.8	[73.8 - 79.5]	75.6	[72.1 - 78.8]	0.13
**'CVM_N55265_(ST228).fna'**	**'LB00.E.122_(ST21).fasta'**	74.2	[70.2 - 77.8]	76.8	[73.9 - 79.6]	77.4	[73.9 - 80.5]	0.25
**'DSM111623_(ST624).fna'**	**'T11_(ST65).fna'**	83.1	[79.3 - 86.3]	76.8	[73.8 - 79.6]	84.9	[81.7 - 87.7]	0.19
**'C116_(ST228).fna'**	**'F_(ST712).fna'**	74.6	[70.6 - 78.2]	76.8	[73.8 - 79.5]	77.7	[74.3 - 80.8]	0.01
**'CVM_N52587_(STx).fna'**	**'E1_(ST40).fna'**	70.4	[66.5 - 74.0]	76.8	[73.8 - 79.5]	74.0	[70.5 - 77.2]	0.14
**'DSM111623_(ST624).fna'**	**'EF348_(ST16).fna'**	74.3	[70.3 - 77.9]	76.8	[73.8 - 79.6]	77.4	[74.0 - 80.6]	0.07
**'4928STDY7071435_(ST40).fna'**	**'C138_(ST228).fna'**	70.1	[66.2 - 73.8]	76.8	[73.8 - 79.5]	73.7	[70.2 - 76.9]	0.12
**'CVM_N55265_(ST228).fna'**	**'EF348_(ST16).fna'**	73.2	[69.3 - 76.9]	76.8	[73.8 - 79.6]	76.5	[73.0 - 79.6]	0.05
**'BT00.E.21_(ST23).fasta'**	**'CVM N52662_(ST228).fna'**	72.5	[68.5 - 76.1]	76.8	[73.8 - 79.5]	75.8	[72.4 - 79.0]	0.42
**'DSM111623_(ST624).fna'**	**'EnGen0400_(ST86).fna'**	78.6	[74.6 - 82.1]	76.8	[73.8 - 79.6]	81.2	[77.8 - 84.1]	0.21
**'209EA1_(ST624).fna'**	**'4928STDY7071351_(ST30).fna'**	75.7	[71.7 - 79.3]	76.8	[73.8 - 79.5]	78.7	[75.2 - 81.8]	0.55
**'C138_(ST228).fna'**	**'CL9172_(ST21).fasta'**	77.2	[73.2 - 80.8]	76.8	[73.8 - 79.6]	80.0	[76.6 - 83.0]	0.17
**'209EA1_(ST624).fna'**	**'CL9797_(ST525).fasta'**	71.6	[67.6 - 75.2]	76.8	[73.8 - 79.6]	75.0	[71.5 - 78.2]	0.62
**'BIOML_A5_(ST875).fna'**	**'DSM111623_(ST624).fna'**	80.5	[76.6 - 83.9]	76.8	[73.8 - 79.6]	82.8	[79.5 - 85.7]	0.14

Query	Subject	d_0	C.I. d_0	d_4	C.I. d_4	d_6	C.I. d_6	Diff. G+C Percent
**'G81_(ST1468).fna'**	**'L14_(ST330).fna'**	77.4	[73.4 - 81.0]	76.8	[73.8 - 79.5]	80.1	[76.7 - 83.2]	0.06
**'CL9314_(ST21).fasta'**	**'DSM111623_(ST624).fna'**	78.6	[74.7 - 82.1]	76.8	[73.8 - 79.6]	81.2	[77.8 - 84.2]	0.05
**'CL9172_(ST21).fasta'**	**'DSM111623_(ST624).fna'**	79.3	[75.3 - 82.8]	76.8	[73.8 - 79.5]	81.8	[78.4 - 84.7]	0.04
**'DSM111623_(ST624).fna'**	**'F1_(ST72).fna'**	74.2	[70.2 - 77.8]	76.8	[73.8 - 79.5]	77.3	[73.9 - 80.5]	0.03
**'DSM111623_(ST624).fna'**	**'Merz96_(ST103).fna'**	72.2	[68.2 - 75.8]	76.8	[73.8 - 79.6]	75.6	[72.1 - 78.8]	0.13
**'CVM_N60027F_(ST862).fna'**	**'DSM111623_(ST624).fna'**	81.9	[78.0 - 85.2]	76.8	[73.9 - 79.6]	83.9	[80.7 - 86.7]	0.08
**'209EA1_(ST624).fna'**	**'4928STDY7071435_(ST40).fna'**	73.5	[69.5 - 77.1]	76.8	[73.8 - 79.6]	76.7	[73.3 - 79.9]	0.01
**'302EA1_(ST122).fna'**	**'C138_(ST228).fna'**	77.7	[73.8 - 81.3]	76.8	[73.8 - 79.5]	80.4	[77.0 - 83.4]	0.13
**'C116_(ST228).fna'**	**'LB00.E.122_(ST21).fasta'**	74.5	[70.5 - 78.1]	76.8	[73.8 - 79.6]	77.6	[74.1 - 80.7]	0.24
**'EnGen0400_(ST86).fna'**	**'G81_(ST1468).fna'**	78.7	[74.7 - 82.2]	76.8	[73.8 - 79.5]	81.2	[77.9 - 84.2]	0.13
**'209EA1_(ST624).fna'**	**'CL6870_(ST103).fasta'**	75.2	[71.2 - 78.8]	76.8	[73.8 - 79.6]	78.2	[74.8 - 81.3]	0.46
**'CL9824_(ST631).fasta'**	**'CVM N53420_(ST228).fna'**	80.1	[76.1 - 83.5]	76.8	[73.8 - 79.6]	82.4	[79.1 - 85.3]	0.27
**'C138_(ST228).fna'**	**'E1_(ST40).fna'**	72.2	[68.3 - 75.9]	76.8	[73.9 - 79.6]	75.6	[72.1 - 78.8]	0.25
**'CL9824_(ST631).fasta'**	**'CVM_N55265_(ST228).fna'**	85.1	[81.4 - 88.2]	76.8	[73.9 - 79.6]	86.6	[83.6 - 89.2]	0.12
**'DSM111623_(ST624).fna'**	*Enterococcus faecalis* NBRC 100480	79.2	[75.2 - 82.6]	76.8	[73.8 - 79.6]	81.6	[78.3 - 84.6]	0.05
**'C138_(ST228).fna'**	**'EF348_(ST16).fna'**	73.5	[69.5 - 77.1]	76.8	[73.8 - 79.6]	76.7	[73.2 - 79.9]	0.06
**'DSM111623_(ST624).fna'**	**'EN788_(ST82).fna'**	70.3	[66.4 - 74.0]	76.8	[73.8 - 79.6]	73.9	[70.4 - 77.1]	0.54
**'C138_(ST228).fna'**	**'L14_(ST330).fna'**	79.7	[75.8 - 83.2]	76.8	[73.8 - 79.5]	82.1	[78.8 - 85.0]	0.01
**'14EA1_(ST147).fna'**	**'R48_(ST228).fna'**	77.1	[73.1 - 80.6]	76.7	[73.7 - 79.5]	79.8	[76.4 - 82.9]	0.2
**'CL9314_(ST21).fasta'**	**'CVM N53420_(ST228).fna'**	73.3	[69.3 - 76.9]	76.7	[73.7 - 79.4]	76.5	[73.0 - 79.6]	0.02
**'C138_(ST228).fna'**	**'Praia.S.M2.C4_(ST62).fasta'**	76.4	[72.4 - 80.0]	76.7	[73.7 - 79.5]	79.3	[75.8 - 82.3]	0.23
**'C138_(ST228).fna'**	**'P9 CL A7_(ST4).fna'**	70.9	[67.0 - 74.6]	76.7	[73.7 - 79.5]	74.4	[70.9 - 77.6]	0.56
**'244_EFLS_(ST443).fna'**	**'C138_(ST228).fna'**	78.2	[74.2 - 81.7]	76.7	[73.7 - 79.4]	80.8	[77.4 - 83.8]	0.1
**'209EA1_(ST624).fna'**	**'CVM_N60027F_(ST862).fna'**	86.3	[82.7 - 89.3]	76.7	[73.7 - 79.5]	87.6	[84.6 - 90.1]	0.18
**'C116_(ST228).fna'**	**'CL9172_(ST21).fasta'**	77.0	[73.0 - 80.6]	76.7	[73.7 - 79.5]	79.8	[76.4 - 82.8]	0.15
**'CL9797_(ST525).fasta'**	**'DSM111623_(ST624).fna'**	69.0	[65.1 - 72.7]	76.7	[73.7 - 79.5]	72.7	[69.2 - 75.9]	0.36
**'EN788_(ST82).fna'**	**'R48_(ST228).fna'**	69.1	[65.2 - 72.7]	76.7	[73.7 - 79.4]	72.8	[69.3 - 76.0]	0.63

Query	Subject	d_0	C.I. d_0	d_4	C.I. d_4	d_6	C.I. d_6	Diff. G+C Percent
**'20_SD_W_06_(ST368).fna'**	**'R48_(ST228).fna'**	75.5	[71.5 - 79.1]	76.7	[73.7 - 79.4]	78.5	[75.0 - 81.5]	0.21
**'DSM111623_(ST624).fna'**	**'LB00.E.122_(ST21).fasta'**	76.7	[72.7 - 80.3]	76.7	[73.7 - 79.4]	79.5	[76.1 - 82.6]	0.13
**'4928STDY7071435_(ST40).fna'**	**'C116_(ST228).fna'**	71.9	[68.0 - 75.6]	76.7	[73.7 - 79.4]	75.3	[71.8 - 78.5]	0.13
**'4928STDY7071435_(ST40).fna'**	**'G81_(ST1468).fna'**	73.0	[69.0 - 76.6]	76.7	[73.7 - 79.5]	76.3	[72.8 - 79.4]	0.17
**'CL8682_(ST769).fasta'**	**'DSM111623_(ST624).fna'**	72.5	[68.6 - 76.2]	76.7	[73.7 - 79.4]	75.8	[72.4 - 79.0]	0.16
**'CL9824_(ST631).fasta'**	**'EN24_(ST228).fna'**	83.5	[79.6 - 86.7]	76.7	[73.8 - 79.5]	85.2	[82.0 - 87.9]	0.17
**'209EA1_(ST624).fna'**	**'F_(ST712).fna'**	76.0	[72.0 - 79.6]	76.7	[73.7 - 79.5]	78.9	[75.5 - 82.0]	0.14
**'CL9172_(ST21).fasta'**	**'CVM N53420_(ST228).fna'**	73.7	[69.8 - 77.4]	76.7	[73.7 - 79.5]	76.9	[73.5 - 80.1]	0.01
**'CVM_N60027F_(ST862).fna'**	**'G81_(ST1468).fna'**	79.8	[75.8 - 83.2]	76.7	[73.7 - 79.4]	82.1	[78.8 - 85.1]	0.0
**'CL6870_(ST103).fasta'**	**'DSM111623_(ST624).fna'**	72.4	[68.4 - 76.0]	76.7	[73.7 - 79.5]	75.7	[72.2 - 78.9]	0.2
**'CVM_N55265_(ST228).fna'**	**'Praia.S.M2.C4_(ST62).fasta'**	76.2	[72.2 - 79.7]	76.7	[73.7 - 79.4]	79.0	[75.6 - 82.1]	0.22
**'CVM N53420_(ST228).fna'**	**'E1_(ST40).fna'**	75.1	[71.1 - 78.7]	76.7	[73.7 - 79.5]	78.2	[74.7 - 81.3]	0.09
**'CVM_N54595_(ST192).fna'**	**'G81_(ST1468).fna'**	81.6	[77.7 - 84.9]	76.7	[73.7 - 79.5]	83.7	[80.4 - 86.5]	0.15
**'14EA1_(ST147).fna'**	**'CVM_N52587_(STx).fna'**	80.8	[76.9 - 84.2]	76.7	[73.7 - 79.5]	83.0	[79.7 - 85.9]	0.27
**'CVM N53420_(ST228).fna'**	**'EN242_(ST82).fna'**	71.6	[67.7 - 75.3]	76.7	[73.7 - 79.5]	75.1	[71.6 - 78.2]	0.39
**'DSM111623_(ST624).fna'**	**'L14_(ST330).fna'**	80.5	[76.6 - 83.9]	76.7	[73.7 - 79.5]	82.8	[79.5 - 85.7]	0.14
**'G81_(ST1468).fna'**	**'T11_(ST65).fna'**	79.8	[75.9 - 83.3]	76.7	[73.7 - 79.5]	82.2	[78.9 - 85.1]	0.11
**'BT00.E.21_(ST23).fasta'**	**'CVM_N52587_(STx).fna'**	71.6	[67.7 - 75.3]	76.7	[73.7 - 79.4]	75.1	[71.6 - 78.2]	0.39
**'C116_(ST228).fna'**	**'EN788_(ST82).fna'**	69.2	[65.3 - 72.8]	76.6	[73.6 - 79.4]	72.8	[69.4 - 76.1]	0.66
**'EF349_(ST631).fna'**	**'EN24_(ST228).fna'**	80.0	[76.1 - 83.5]	76.6	[73.6 - 79.3]	82.3	[79.0 - 85.2]	1.09
**'DSM111623_(ST624).fna'**	**'E1_(ST40).fna'**	76.4	[72.4 - 80.0]	76.6	[73.6 - 79.4]	79.2	[75.8 - 82.3]	0.12
**'EF348_(ST16).fna'**	**'EN24_(ST228).fna'**	73.4	[69.5 - 77.1]	76.6	[73.6 - 79.3]	76.6	[73.2 - 79.8]	0.0
**'244_EFLS_(ST443).fna'**	**'CVM_N52587_(STx).fna'**	77.1	[73.1 - 80.6]	76.6	[73.6 - 79.3]	79.8	[76.4 - 82.8]	0.0
**'20_SD_W_06_(ST368).fna'**	**'C138_(ST228).fna'**	76.8	[72.8 - 80.3]	76.6	[73.6 - 79.4]	79.6	[76.2 - 82.6]	0.25
**'20_SD_W_06_(ST368).fna'**	**'C116_(ST228).fna'**	76.2	[72.2 - 79.8]	76.6	[73.6 - 79.4]	79.1	[75.7 - 82.2]	0.24
**'14EA1_(ST147).fna'**	**'CVM_N55265_(ST228).fna'**	81.9	[78.0 - 85.2]	76.6	[73.6 - 79.4]	83.9	[80.7 - 86.7]	0.18
**'CVM_N52587_(STx).fna'**	**'Praia.S.M2.C4_(ST62).fasta'**	75.1	[71.1 - 78.7]	76.6	[73.6 - 79.3]	78.1	[74.7 - 81.2]	0.13

Query	Subject	d_0	C.I. d_0	d_4	C.I. d_4	d_6	C.I. d_6	Diff. G+C Percent
**'E1_(ST40).fna'**	**'R48_(ST228).fna'**	73.9	[69.9 - 77.5]	76.6	[73.6 - 79.4]	77.0	[73.6 - 80.2]	0.21
**'DSM111623_(ST624).fna'**	**'EF349_(ST631).fna'**	80.3	[76.4 - 83.8]	76.6	[73.6 - 79.3]	82.6	[79.3 - 85.5]	1.16
**'C138_(ST228).fna'**	*Enterococcus faecalis* NBRC 100480	79.8	[75.9 - 83.3]	76.6	[73.6 - 79.4]	82.2	[78.8 - 85.1]	0.08
**'CVM_N60027F_(ST862).fna'**	**'R48_(ST228).fna'**	80.8	[76.9 - 84.2]	76.6	[73.6 - 79.3]	83.0	[79.7 - 85.8]	0.01
**'CL9924_(ST6).fasta'**	*Aequorivita lutea* q18	12.8	[10.1 - 16.1]	76.6	[73.6 - 79.4]	13.2	[10.9 - 16.0]	5.51
**'CVM_N54595_(ST192).fna'**	**'DSM111623_(ST624).fna'**	73.6	[69.6 - 77.2]	76.6	[73.6 - 79.4]	76.8	[73.3 - 79.9]	0.07
**'P9 CL A7_(ST4).fna'**	**'R48_(ST228).fna'**	69.8	[65.9 - 73.4]	76.6	[73.6 - 79.3]	73.4	[69.9 - 76.6]	0.52
**'4928STDY7071435_(ST40).fna'**	**'CVM N52662_(ST228).fna'**	72.3	[68.4 - 76.0]	76.6	[73.6 - 79.3]	75.6	[72.2 - 78.8]	0.19
**'C116_(ST228).fna'**	**'EF348_(ST16).fna'**	74.5	[70.5 - 78.1]	76.6	[73.6 - 79.3]	77.6	[74.1 - 80.7]	0.05
**'244_EFLS_(ST443).fna'**	**'CVM_N55265_(ST228).fna'**	78.2	[74.2 - 81.7]	76.6	[73.6 - 79.4]	80.8	[77.4 - 83.8]	0.09
**'4928STDY7071351_(ST30).fna'**	**'DSM111623_(ST624).fna'**	73.0	[69.0 - 76.6]	76.6	[73.6 - 79.3]	76.2	[72.8 - 79.4]	0.29
**'EnGen0400_(ST86).fna'**	**'R48_(ST228).fna'**	80.7	[76.8 - 84.1]	76.6	[73.6 - 79.3]	82.9	[79.6 - 85.8]	0.12
**'EF348_(ST16).fna'**	**'R48_(ST228).fna'**	74.5	[70.5 - 78.1]	76.6	[73.6 - 79.3]	77.5	[74.1 - 80.7]	0.03
**'D32_(ST40).fna'**	**'DSM111623_(ST624).fna'**	74.8	[70.8 - 78.4]	76.6	[73.6 - 79.4]	77.8	[74.4 - 80.9]	0.04
**'15224_(ST16).fna'**	**'G81_(ST1468).fna'**	67.7	[63.8 - 71.4]	76.6	[73.6 - 79.4]	71.5	[68.0 - 74.7]	0.48
**'209EA1_(ST624).fna'**	**'19910_(ST116).fna'**	74.9	[70.9 - 78.5]	76.6	[73.6 - 79.4]	77.9	[74.5 - 81.0]	0.45
**'20_SD_W_06_(ST368).fna'**	**'209EA1_(ST624).fna'**	81.4	[77.5 - 84.7]	76.6	[73.6 - 79.3]	83.5	[80.2 - 86.3]	0.38
**'209EA1_(ST624).fna'**	**'CL8682_(ST769).fasta'**	75.2	[71.2 - 78.8]	76.6	[73.6 - 79.4]	78.2	[74.8 - 81.3]	0.43
**'G81_(ST1468).fna'**	**'Merz96_(ST103).fna'**	71.8	[67.8 - 75.4]	76.5	[73.5 - 79.3]	75.2	[71.7 - 78.4]	0.05
**'302EA1_(ST122).fna'**	**'DSM111623_(ST624).fna'**	76.9	[72.9 - 80.4]	76.5	[73.5 - 79.2]	79.6	[76.2 - 82.7]	0.0
**'302EA1_(ST122).fna'**	**'CVM_N52587_(STx).fna'**	77.4	[73.4 - 80.9]	76.5	[73.5 - 79.3]	80.1	[76.7 - 83.1]	0.02
**'C116_(ST228).fna'**	**'P9 CL A7_(ST4).fna'**	70.5	[66.5 - 74.1]	76.5	[73.5 - 79.3]	74.0	[70.5 - 77.2]	0.54
**'CVM N53420_(ST228).fna'**	**'EnGen0400_(ST86).fna'**	80.9	[77.0 - 84.3]	76.5	[73.6 - 79.3]	83.1	[79.8 - 85.9]	0.24
**'CVM_N55265_(ST228).fna'**	**'P9 CL A7_(ST4).fna'**	71.5	[67.6 - 75.2]	76.5	[73.5 - 79.3]	74.9	[71.4 - 78.1]	0.55
**'CL9314_(ST21).fasta'**	**'EN24_(ST228).fna'**	76.0	[72.0 - 79.5]	76.5	[73.5 - 79.3]	78.8	[75.4 - 81.9]	0.12
**'CVM_N52587_(STx).fna'**	**'EN788_(ST82).fna'**	69.0	[65.1 - 72.6]	76.5	[73.5 - 79.3]	72.6	[69.1 - 75.8]	0.56
**'C138_(ST228).fna'**	**'Merz96_(ST103).fna'**	72.6	[68.6 - 76.2]	76.5	[73.5 - 79.3]	75.9	[72.4 - 79.0]	0.01

Query	Subject	d_0	C.I. d_0	d_4	C.I. d_4	d_6	C.I. d_6	Diff. G+C Percent
**'4928STDY7071435_(ST40).fna'**	**'DSM111623_(ST624).fna'**	74.2	[70.3 - 77.9]	76.5	[73.5 - 79.3]	77.3	[73.9 - 80.5]	0.25
**'L14_(ST330).fna'**	**'R48_(ST228).fna'**	78.2	[74.2 - 81.7]	76.5	[73.5 - 79.2]	80.8	[77.4 - 83.7]	0.04
**'CL9172_(ST21).fasta'**	**'R48_(ST228).fna'**	77.9	[73.9 - 81.4]	76.5	[73.5 - 79.3]	80.5	[77.1 - 83.5]	0.13
**'244_EFLS_(ST443).fna'**	**'C116_(ST228).fna'**	77.8	[73.8 - 81.3]	76.5	[73.5 - 79.3]	80.4	[77.0 - 83.4]	0.09
**'19910_(ST116).fna'**	**'G81_(ST1468).fna'**	70.8	[66.8 - 74.4]	76.5	[73.5 - 79.3]	74.3	[70.8 - 77.4]	0.26
**'DSM111623_(ST624).fna'**	**'KUB3007_(ST729).fna'**	71.3	[67.3 - 74.9]	76.5	[73.5 - 79.3]	74.7	[71.2 - 77.9]	0.3
**'G81_(ST1468).fna'**	**'Praia.S.M2.C4_(ST62).fasta'**	74.6	[70.6 - 78.2]	76.5	[73.5 - 79.3]	77.6	[74.2 - 80.7]	0.18
**'20_SD_W_06_(ST368).fna'**	**'EN24_(ST228).fna'**	75.4	[71.4 - 79.0]	76.5	[73.5 - 79.3]	78.3	[74.9 - 81.4]	0.19
**'CVM_N54595_(ST192).fna'**	**'R48_(ST228).fna'**	76.9	[73.0 - 80.5]	76.5	[73.5 - 79.3]	79.7	[76.3 - 82.7]	0.16
**'C116_(ST228).fna'**	**'EN242_(ST82).fna'**	71.8	[67.8 - 75.4]	76.5	[73.5 - 79.2]	75.1	[71.7 - 78.3]	0.53
**'BT00.E.21_(ST23).fasta'**	**'EN24_(ST228).fna'**	71.9	[67.9 - 75.5]	76.5	[73.5 - 79.2]	75.3	[71.8 - 78.4]	0.44
**'CVM_N55265_(ST228).fna'**	**'EN788_(ST82).fna'**	70.2	[66.3 - 73.9]	76.5	[73.5 - 79.3]	73.8	[70.3 - 77.0]	0.66
**'CVM_N52587_(STx).fna'**	**'EN242_(ST82).fna'**	71.4	[67.4 - 75.0]	76.5	[73.5 - 79.2]	74.8	[71.3 - 78.0]	0.44
**'C116_(ST228).fna'**	**'CVM_N60027F_(ST862).fna'**	81.2	[77.3 - 84.5]	76.5	[73.5 - 79.3]	83.3	[80.0 - 86.1]	0.03
**'302EA1_(ST122).fna'**	**'R48_(ST228).fna'**	77.1	[73.2 - 80.7]	76.5	[73.5 - 79.3]	79.9	[76.5 - 82.9]	0.09
**'EF348_(ST16).fna'**	*Aequorivita lutea* q18	12.8	[10.1 - 16.1]	76.5	[73.5 - 79.2]	13.2	[10.9 - 16.0]	5.12
**'D32_(ST40).fna'**	**'G81_(ST1468).fna'**	72.3	[68.3 - 75.9]	76.5	[73.5 - 79.2]	75.6	[72.1 - 78.8]	0.12
**'15224_(ST16).fna'**	**'C138_(ST228).fna'**	67.3	[63.5 - 71.0]	76.5	[73.5 - 79.2]	71.1	[67.7 - 74.3]	0.53
**'EN242_(ST82).fna'**	**'G81_(ST1468).fna'**	71.7	[67.8 - 75.4]	76.5	[73.5 - 79.2]	75.1	[71.6 - 78.3]	0.5
**'4928STDY7071435_(ST40).fna'**	**'R48_(ST228).fna'**	74.2	[70.2 - 77.8]	76.5	[73.5 - 79.3]	77.3	[73.8 - 80.4]	0.16
**'EN24_(ST228).fna'**	**'L14_(ST330).fna'**	78.5	[74.5 - 82.0]	76.5	[73.5 - 79.3]	81.0	[77.6 - 84.0]	0.07
**'20_SD_W_06_(ST368).fna'**	**'CVM N53420_(ST228).fna'**	72.5	[68.5 - 76.1]	76.5	[73.5 - 79.2]	75.8	[72.3 - 78.9]	0.09
**'14EA1_(ST147).fna'**	**'CVM N52662_(ST228).fna'**	79.6	[75.6 - 83.0]	76.5	[73.5 - 79.3]	82.0	[78.6 - 84.9]	0.24
**'EN24_(ST228).fna'**	**'EN788_(ST82).fna'**	71.4	[67.5 - 75.1]	76.5	[73.5 - 79.2]	74.8	[71.4 - 78.0]	0.61
**'CVM_N55265_(ST228).fna'**	**'CVM_N60027F_(ST862).fna'**	82.6	[78.8 - 85.9]	76.5	[73.5 - 79.2]	84.5	[81.3 - 87.2]	0.03
**'EN24_(ST228).fna'**	**'EN242_(ST82).fna'**	72.2	[68.2 - 75.8]	76.5	[73.5 - 79.2]	75.5	[72.0 - 78.7]	0.49
**'CVM N52662_(ST228).fna'**	**'CVM_N60027F_(ST862).fna'**	81.3	[77.4 - 84.7]	76.5	[73.5 - 79.2]	83.4	[80.1 - 86.2]	0.03

Query	Subject	d_0	C.I. d_0	d_4	C.I. d_4	d_6	C.I. d_6	Diff. G+C Percent
**'CVM N52662_(ST228).fna'**	**'EN242_(ST82).fna'**	72.4	[68.4 - 76.0]	76.5	[73.5 - 79.2]	75.7	[72.2 - 78.9]	0.48
**'C138_(ST228).fna'**	**'F1_(ST72).fna'**	75.9	[71.9 - 79.5]	76.5	[73.5 - 79.2]	78.8	[75.4 - 81.9]	0.1
**'G81_(ST1468).fna'**	**'P9 CL A7_(ST4).fna'**	68.9	[65.0 - 72.6]	76.5	[73.5 - 79.2]	72.6	[69.1 - 75.8]	0.51
**'302EA1_(ST122).fna'**	**'CVM_N55265_(ST228).fna'**	78.3	[74.4 - 81.8]	76.5	[73.5 - 79.2]	80.9	[77.5 - 83.9]	0.12
**'4928STDY7071435_(ST40).fna'**	**'CVM_N55265_(ST228).fna'**	69.5	[65.6 - 73.2]	76.5	[73.5 - 79.3]	73.1	[69.6 - 76.3]	0.13
**'CVM_N54595_(ST192).fna'**	*Aequorivita lutea* q18	12.8	[10.1 - 16.1]	76.5	[73.5 - 79.2]	13.2	[10.9 - 16.0]	5.25
**'4928STDY7071435_(ST40).fna'**	**'CVM_N52587_(STx).fna'**	68.4	[64.5 - 72.0]	76.5	[73.5 - 79.3]	72.1	[68.6 - 75.3]	0.23
**'CVM N53420_(ST228).fna'**	**'P9 CL A7_(ST4).fna'**	68.6	[64.7 - 72.2]	76.5	[73.5 - 79.2]	72.3	[68.8 - 75.5]	0.4
**'C138_(ST228).fna'**	**'CL9943_(ST4).fasta'**	68.2	[64.3 - 71.8]	76.5	[73.5 - 79.2]	71.9	[68.4 - 75.1]	0.62
**'CVM N52662_(ST228).fna'**	**'P9 CL A7_(ST4).fna'**	70.1	[66.1 - 73.7]	76.5	[73.5 - 79.2]	73.6	[70.1 - 76.8]	0.49
**'EF349_(ST631).fna'**	**'R48_(ST228).fna'**	77.5	[73.5 - 81.0]	76.5	[73.5 - 79.2]	80.1	[76.7 - 83.1]	1.07
**'CVM_N52587_(STx).fna'**	**'T11_(ST65).fna'**	81.4	[77.5 - 84.8]	76.5	[73.5 - 79.2]	83.5	[80.2 - 86.3]	0.16
**'20_SD_W_06_(ST368).fna'**	**'G81_(ST1468).fna'**	75.8	[71.8 - 79.4]	76.5	[73.5 - 79.3]	78.7	[75.3 - 81.8]	0.2
**'14EA1_(ST147).fna'**	**'C116_(ST228).fna'**	77.7	[73.7 - 81.2]	76.5	[73.5 - 79.3]	80.3	[76.9 - 83.3]	0.18
**'BIOML_A5_(ST875).fna'**	**'G81_(ST1468).fna'**	74.8	[70.8 - 78.4]	76.5	[73.5 - 79.2]	77.8	[74.3 - 80.9]	0.22
**'C138_(ST228).fna'**	**'T11_(ST65).fna'**	82.7	[78.8 - 86.0]	76.5	[73.5 - 79.2]	84.5	[81.3 - 87.3]	0.06
**'C116_(ST228).fna'**	**'D32_(ST40).fna'**	73.4	[69.4 - 77.0]	76.5	[73.5 - 79.3]	76.6	[73.1 - 79.8]	0.16
**'4928STDY7071351_(ST30).fna'**	**'C138_(ST228).fna'**	73.1	[69.1 - 76.7]	76.5	[73.5 - 79.2]	76.3	[72.9 - 79.5]	0.42
**'244_EFLS_(ST443).fna'**	**'CVM N52662_(ST228).fna'**	76.0	[72.0 - 79.5]	76.5	[73.5 - 79.3]	78.8	[75.4 - 81.9]	0.03
**'EN24_(ST228).fna'**	**'F_(ST712).fna'**	76.4	[72.4 - 79.9]	76.5	[73.5 - 79.2]	79.2	[75.8 - 82.3]	0.06
**'C138_(ST228).fna'**	**'EF349_(ST631).fna'**	80.3	[76.4 - 83.7]	76.5	[73.5 - 79.2]	82.6	[79.2 - 85.4]	1.03
**'F1_(ST72).fna'**	**'G81_(ST1468).fna'**	75.0	[71.0 - 78.6]	76.5	[73.5 - 79.2]	78.0	[74.6 - 81.1]	0.05
**'BT00.E.21_(ST23).fasta'**	**'G81_(ST1468).fna'**	69.8	[65.9 - 73.5]	76.5	[73.5 - 79.2]	73.4	[69.9 - 76.6]	0.45
**'19910_(ST116).fna'**	**'CVM_N52587_(STx).fna'**	73.7	[69.7 - 77.3]	76.4	[73.4 - 79.1]	76.9	[73.4 - 80.0]	0.21
**'CVM_N52587_(STx).fna'**	**'Merz96_(ST103).fna'**	71.1	[67.1 - 74.7]	76.4	[73.4 - 79.1]	74.5	[71.0 - 77.7]	0.11
**'C138_(ST228).fna'**	**'F_(ST712).fna'**	75.2	[71.2 - 78.8]	76.4	[73.4 - 79.2]	78.2	[74.7 - 81.3]	0.0
**'CVM_N55265_(ST228).fna'**	**'L14_(ST330).fna'**	81.5	[77.6 - 84.9]	76.4	[73.4 - 79.1]	83.5	[80.3 - 86.4]	0.02

Query	Subject	d_0	C.I. d_0	d_4	C.I. d_4	d_6	C.I. d_6	Diff. G+C Percent
**'EN788_(ST82).fna'**	**'G81_(ST1468).fna'**	70.7	[66.8 - 74.4]	76.4	[73.4 - 79.2]	74.2	[70.7 - 77.4]	0.62
**'CVM N52662_(ST228).fna'**	**'EN788_(ST82).fna'**	70.0	[66.0 - 73.6]	76.4	[73.4 - 79.2]	73.5	[70.0 - 76.7]	0.6
**'C138_(ST228).fna'**	**'EN242_(ST82).fna'**	71.0	[67.0 - 74.6]	76.4	[73.4 - 79.2]	74.4	[70.9 - 77.6]	0.55
**'F_(ST712).fna'**	**'R48_(ST228).fna'**	76.3	[72.4 - 79.9]	76.4	[73.4 - 79.2]	79.2	[75.7 - 82.2]	0.03
**'C116_(ST228).fna'**	**'EF349_(ST631).fna'**	78.9	[74.9 - 82.4]	76.4	[73.4 - 79.2]	81.4	[78.0 - 84.3]	1.04
**'302EA1_(ST122).fna'**	**'CVM N52662_(ST228).fna'**	76.4	[72.4 - 80.0]	76.4	[73.4 - 79.2]	79.2	[75.8 - 82.3]	0.06
**'CVM N53420_(ST228).fna'**	**'T11_(ST65).fna'**	80.2	[76.3 - 83.7]	76.4	[73.4 - 79.1]	82.5	[79.2 - 85.4]	0.22
**'C138_(ST228).fna'**	**'EN788_(ST82).fna'**	70.1	[66.2 - 73.8]	76.4	[73.4 - 79.2]	73.7	[70.2 - 76.9]	0.67
**'15224_(ST16).fna'**	**'CVM N52662_(ST228).fna'**	68.8	[64.9 - 72.4]	76.4	[73.4 - 79.1]	72.4	[68.9 - 75.6]	0.46
**'4928STDY7071351_(ST30).fna'**	**'CVM_N55265_(ST228).fna'**	72.3	[68.3 - 76.0]	76.4	[73.4 - 79.2]	75.6	[72.1 - 78.8]	0.41
**'EN24_(ST228).fna'**	**'LB00.E.122_(ST21).fasta'**	74.7	[70.7 - 78.3]	76.4	[73.4 - 79.1]	77.7	[74.3 - 80.8]	0.2
**'C138_(ST228).fna'**	**'CVM_N54595_(ST192).fna'**	74.6	[70.6 - 78.2]	76.4	[73.4 - 79.1]	77.6	[74.2 - 80.7]	0.2
**'CVM N53420_(ST228).fna'**	**'CVM_N60027F_(ST862).fna'**	79.0	[75.0 - 82.5]	76.4	[73.4 - 79.2]	81.4	[78.1 - 84.4]	0.11
**'CVM_N55265_(ST228).fna'**	**'F_(ST712).fna'**	77.2	[73.2 - 80.7]	76.4	[73.4 - 79.1]	79.9	[76.4 - 82.9]	0.01
**'CVM_N52587_(STx).fna'**	**'P9 CL A7_(ST4).fna'**	70.7	[66.8 - 74.4]	76.4	[73.4 - 79.1]	74.2	[70.7 - 77.4]	0.45
**'CVM_N52587_(STx).fna'**	**'L14_(ST330).fna'**	80.1	[76.1 - 83.5]	76.4	[73.4 - 79.1]	82.3	[79.0 - 85.2]	0.11
**'15224_(ST16).fna'**	**'CVM_N52587_(STx).fna'**	66.4	[62.6 - 70.1]	76.4	[73.4 - 79.2]	70.3	[66.8 - 73.5]	0.42
**'D32_(ST40).fna'**	**'EN24_(ST228).fna'**	71.6	[67.6 - 75.2]	76.4	[73.4 - 79.2]	75.0	[71.5 - 78.1]	0.11
**'CVM_N55265_(ST228).fna'**	**'T11_(ST65).fna'**	82.9	[79.1 - 86.2]	76.4	[73.4 - 79.2]	84.7	[81.5 - 87.5]	0.07
**'CL11199_(ST778).fasta'**	**'R48_(ST228).fna'**	69.9	[66.0 - 73.6]	76.4	[73.4 - 79.1]	73.5	[70.0 - 76.7]	0.37
**'15224_(ST16).fna'**	**'CVM_N55265_(ST228).fna'**	67.3	[63.4 - 70.9]	76.4	[73.4 - 79.2]	71.1	[67.6 - 74.3]	0.52
**'C138_(ST228).fna'**	**'CVM_N60027F_(ST862).fna'**	82.9	[79.0 - 86.1]	76.4	[73.5 - 79.2]	84.7	[81.5 - 87.4]	0.04
**'LB00.E.122_(ST21).fasta'**	**'R48_(ST228).fna'**	76.2	[72.2 - 79.7]	76.4	[73.4 - 79.2]	79.0	[75.6 - 82.1]	0.22
**'C116_(ST228).fna'**	*Enterococcus faecalis* NBRC 100480	79.5	[75.5 - 82.9]	76.4	[73.4 - 79.1]	81.8	[78.5 - 84.8]	0.07
**'CVM_N55265_(ST228).fna'**	**'EN242_(ST82).fna'**	72.7	[68.8 - 76.4]	76.4	[73.4 - 79.2]	76.0	[72.5 - 79.1]	0.54
**'D32_(ST40).fna'**	**'R48_(ST228).fna'**	72.4	[68.4 - 76.0]	76.4	[73.4 - 79.1]	75.7	[72.2 - 78.8]	0.14
**'BIOML_A5_(ST875).fna'**	**'C138_(ST228).fna'**	77.5	[73.5 - 81.0]	76.4	[73.4 - 79.2]	80.2	[76.7 - 83.2]	0.27

Query	Subject	d_0	C.I. d_0	d_4	C.I. d_4	d_6	C.I. d_6	Diff. G+C Percent
**'14EA1_(ST147).fna'**	**'EN24_(ST228).fna'**	77.8	[73.8 - 81.3]	76.4	[73.4 - 79.1]	80.4	[77.0 - 83.4]	0.23
**'C116_(ST228).fna'**	**'Praia.S.M2.C4_(ST62).fasta'**	76.4	[72.4 - 80.0]	76.4	[73.4 - 79.2]	79.2	[75.8 - 82.3]	0.22
**'15224_(ST16).fna'**	**'R48_(ST228).fna'**	69.3	[65.4 - 73.0]	76.3	[73.3 - 79.1]	72.9	[69.4 - 76.1]	0.49
**'CL9924_(ST6).fasta'**	**'R48_(ST228).fna'**	70.2	[66.3 - 73.9]	76.3	[73.3 - 79.1]	73.7	[70.2 - 76.9]	0.42
**'CVM_N55265_(ST228).fna'**	*Enterococcus faecalis* NBRC 100480	80.4	[76.5 - 83.8]	76.3	[73.3 - 79.1]	82.6	[79.3 - 85.5]	0.07
**'CVM_N52587_(STx).fna'**	*Enterococcus faecalis* NBRC 100480	79.5	[75.6 - 83.0]	76.3	[73.3 - 79.0]	81.9	[78.5 - 84.8]	0.03
**'15224_(ST16).fna'**	**'C116_(ST228).fna'**	69.0	[65.1 - 72.7]	76.3	[73.3 - 79.0]	72.6	[69.2 - 75.9]	0.52
**'EN24_(ST228).fna'**	**'EnGen0400_(ST86).fna'**	79.9	[75.9 - 83.3]	76.3	[73.3 - 79.1]	82.2	[78.8 - 85.1]	0.15
**'CVM N52662_(ST228).fna'**	*Enterococcus faecalis* NBRC 100480	78.2	[74.2 - 81.7]	76.3	[73.3 - 79.0]	80.7	[77.3 - 83.7]	0.01
**'CVM_N52587_(STx).fna'**	**'F1_(ST72).fna'**	77.4	[73.4 - 80.9]	76.3	[73.3 - 79.1]	80.0	[76.6 - 83.1]	0.0
**'20_SD_W_06_(ST368).fna'**	**'CVM_N55265_(ST228).fna'**	78.3	[74.3 - 81.8]	76.3	[73.3 - 79.0]	80.8	[77.4 - 83.8]	0.24
**'G81_(ST1468).fna'**	**'KUB3007_(ST729).fna'**	71.9	[68.0 - 75.6]	76.3	[73.3 - 79.1]	75.3	[71.8 - 78.4]	0.38
**'R48_(ST228).fna'**	**'T11_(ST65).fna'**	81.0	[77.1 - 84.4]	76.3	[73.3 - 79.1]	83.1	[79.8 - 86.0]	0.1
**'19910_(ST116).fna'**	**'CVM_N55265_(ST228).fna'**	74.5	[70.6 - 78.2]	76.3	[73.3 - 79.1]	77.6	[74.1 - 80.7]	0.3
**'CVM_N55265_(ST228).fna'**	**'Merz96_(ST103).fna'**	72.4	[68.4 - 76.1]	76.3	[73.3 - 79.0]	75.7	[72.2 - 78.8]	0.02
**'302EA1_(ST122).fna'**	**'C116_(ST228).fna'**	77.6	[73.6 - 81.1]	76.3	[73.3 - 79.1]	80.2	[76.8 - 83.2]	0.12
**'E1_(ST40).fna'**	**'EN24_(ST228).fna'**	71.8	[67.8 - 75.4]	76.3	[73.3 - 79.1]	75.1	[71.6 - 78.3]	0.19
**'C138_(ST228).fna'**	**'EnGen0400_(ST86).fna'**	78.6	[74.7 - 82.1]	76.3	[73.3 - 79.1]	81.1	[77.7 - 84.1]	0.09
**'302EA1_(ST122).fna'**	**'EN24_(ST228).fna'**	76.1	[72.1 - 79.7]	76.3	[73.3 - 79.1]	78.9	[75.5 - 82.0]	0.07
**'20_SD_W_06_(ST368).fna'**	**'CVM N52662_(ST228).fna'**	76.0	[72.0 - 79.6]	76.3	[73.3 - 79.1]	78.8	[75.4 - 81.9]	0.18
**'CVM_N52587_(STx).fna'**	**'CVM_N60027F_(ST862).fna'**	82.9	[79.0 - 86.1]	76.3	[73.3 - 79.1]	84.7	[81.4 - 87.4]	0.06
**'EF349_(ST631).fna'**	**'G81_(ST1468).fna'**	78.1	[74.1 - 81.6]	76.3	[73.3 - 79.0]	80.6	[77.2 - 83.6]	1.08
**'CL9797_(ST525).fasta'**	*Aequorivita lutea* q18	12.8	[10.1 - 16.1]	76.3	[73.3 - 79.0]	13.2	[10.9 - 16.0]	5.54
**'4928STDY7071351_(ST30).fna'**	**'CVM N52662_(ST228).fna'**	71.2	[67.3 - 74.9]	76.3	[73.3 - 79.0]	74.6	[71.2 - 77.8]	0.34
**'244_EFLS_(ST443).fna'**	**'R48_(ST228).fna'**	77.6	[73.6 - 81.1]	76.3	[73.3 - 79.1]	80.2	[76.8 - 83.2]	0.07
**'C116_(ST228).fna'**	**'T11_(ST65).fna'**	82.3	[78.4 - 85.6]	76.3	[73.3 - 79.0]	84.2	[80.9 - 86.9]	0.07
**'CL9943_(ST4).fasta'**	**'EN24_(ST228).fna'**	70.8	[66.9 - 74.4]	76.2	[73.2 - 78.9]	74.2	[70.7 - 77.4]	0.55

Query	Subject	d_0	C.I. d_0	d_4	C.I. d_4	d_6	C.I. d_6	Diff. G+C Percent
**'C116_(ST228).fna'**	**'F1_(ST72).fna'**	75.2	[71.2 - 78.8]	76.2	[73.2 - 79.0]	78.1	[74.7 - 81.2]	0.09
**'CVM N53420_(ST228).fna'**	**'Merz96_(ST103).fna'**	72.7	[68.8 - 76.4]	76.2	[73.2 - 79.0]	76.0	[72.5 - 79.1]	0.16
**'C116_(ST228).fna'**	**'CL6870_(ST103).fasta'**	74.6	[70.6 - 78.2]	76.2	[73.2 - 78.9]	77.6	[74.1 - 80.7]	0.31
**'BIOML_A5_(ST875).fna'**	**'CVM_N52587_(STx).fna'**	76.6	[72.6 - 80.2]	76.2	[73.2 - 79.0]	79.4	[75.9 - 82.4]	0.16
**'CVM_N55265_(ST228).fna'**	**'D32_(ST40).fna'**	74.6	[70.7 - 78.3]	76.2	[73.2 - 78.9]	77.6	[74.2 - 80.7]	0.16
**'15224_(ST16).fna'**	**'EN24_(ST228).fna'**	68.4	[64.5 - 72.0]	76.2	[73.2 - 79.0]	72.0	[68.6 - 75.3]	0.47
**'20_SD_W_06_(ST368).fna'**	**'CVM_N52587_(STx).fna'**	77.0	[73.0 - 80.6]	76.2	[73.2 - 79.0]	79.7	[76.3 - 82.7]	0.14
**'CL9772_(ST525).fasta'**	**'CVM N53420_(ST228).fna'**	72.3	[68.3 - 75.9]	76.2	[73.2 - 79.0]	75.5	[72.1 - 78.7]	0.25
**'CL6870_(ST103).fasta'**	**'R48_(ST228).fna'**	73.8	[69.9 - 77.5]	76.2	[73.2 - 79.0]	76.9	[73.5 - 80.1]	0.29
**'4928STDY7071351_(ST30).fna'**	**'C116_(ST228).fna'**	73.2	[69.2 - 76.8]	76.2	[73.2 - 78.9]	76.3	[72.9 - 79.5]	0.4
**'BIOML_A5_(ST875).fna'**	**'CVM_N55265_(ST228).fna'**	78.1	[74.1 - 81.6]	76.2	[73.2 - 78.9]	80.6	[77.2 - 83.6]	0.26
**'19910_(ST116).fna'**	**'DSM111623_(ST624).fna'**	73.5	[69.5 - 77.1]	76.2	[73.2 - 78.9]	76.6	[73.1 - 79.8]	0.18
**'C116_(ST228).fna'**	**'Merz96_(ST103).fna'**	73.1	[69.1 - 76.7]	76.2	[73.2 - 78.9]	76.2	[72.8 - 79.4]	0.02
**'C116_(ST228).fna'**	**'EnGen0400_(ST86).fna'**	81.8	[77.9 - 85.1]	76.2	[73.2 - 78.9]	83.7	[80.5 - 86.6]	0.1
**'EN24_(ST228).fna'**	**'F1_(ST72).fna'**	77.1	[73.2 - 80.7]	76.2	[73.2 - 79.0]	79.8	[76.4 - 82.8]	0.04
**'CVM N53420_(ST228).fna'**	**'EF348_(ST16).fna'**	73.2	[69.2 - 76.8]	76.2	[73.2 - 79.0]	76.3	[72.9 - 79.5]	0.1
**'Merz96_(ST103).fna'**	**'R48_(ST228).fna'**	72.3	[68.3 - 75.9]	76.2	[73.2 - 79.0]	75.6	[72.1 - 78.7]	0.04
**'CL9797_(ST525).fasta'**	**'CVM N53420_(ST228).fna'**	71.4	[67.5 - 75.1]	76.2	[73.2 - 79.0]	74.8	[71.3 - 78.0]	0.33
**'EN24_(ST228).fna'**	**'T11_(ST65).fna'**	82.9	[79.1 - 86.2]	76.2	[73.2 - 79.0]	84.7	[81.5 - 87.4]	0.12
**'4928STDY7071435_(ST40).fna'**	**'CVM N53420_(ST228).fna'**	73.2	[69.2 - 76.8]	76.2	[73.2 - 79.0]	76.4	[72.9 - 79.5]	0.28
**'EN24_(ST228).fna'**	**'Praia.S.M2.C4_(ST62).fasta'**	79.8	[75.9 - 83.3]	76.2	[73.2 - 79.0]	82.1	[78.7 - 85.0]	0.17
**'302EA1_(ST122).fna'**	**'G81_(ST1468).fna'**	75.6	[71.6 - 79.2]	76.2	[73.2 - 79.0]	78.5	[75.0 - 81.6]	0.08
**'EN24_(ST228).fna'**	**'KUB3007_(ST729).fna'**	74.2	[70.2 - 77.8]	76.2	[73.2 - 78.9]	77.2	[73.8 - 80.4]	0.37
**'4928STDY7071435_(ST40).fna'**	**'EN24_(ST228).fna'**	69.6	[65.7 - 73.3]	76.2	[73.2 - 79.0]	73.2	[69.7 - 76.4]	0.18
**'CVM_N54595_(ST192).fna'**	**'CVM_N55265_(ST228).fna'**	75.8	[71.8 - 79.4]	76.2	[73.2 - 79.0]	78.7	[75.2 - 81.7]	0.19
**'CL9797_(ST525).fasta'**	**'R48_(ST228).fna'**	71.8	[67.9 - 75.5]	76.2	[73.2 - 79.0]	75.2	[71.7 - 78.3]	0.45
**'CL8682_(ST769).fasta'**	**'R48_(ST228).fna'**	74.1	[70.2 - 77.8]	76.2	[73.2 - 79.0]	77.2	[73.7 - 80.3]	0.26

Query	Subject	d_0	C.I. d_0	d_4	C.I. d_4	d_6	C.I. d_6	Diff. G+C Percent
**'CVM N52662_(ST228).fna'**	**'Praia.S.M2.C4_(ST62).fasta'**	75.4	[71.4 - 79.0]	76.2	[73.2 - 79.0]	78.3	[74.8 - 81.4]	0.16
**'4928STDY7071351_(ST30).fna'**	**'CVM_N52587_(STx).fna'**	72.6	[68.6 - 76.2]	76.2	[73.2 - 79.0]	75.8	[72.3 - 79.0]	0.31
**'CVM N53420_(ST228).fna'**	**'LB00.E.122_(ST21).fasta'**	73.2	[69.2 - 76.8]	76.2	[73.2 - 79.0]	76.4	[72.9 - 79.5]	0.1
**'CVM N53420_(ST228).fna'**	**'EF349_(ST631).fna'**	76.2	[72.3 - 79.8]	76.2	[73.2 - 79.0]	79.0	[75.6 - 82.1]	1.19
**'CL9172_(ST21).fasta'**	**'EN24_(ST228).fna'**	77.4	[73.4 - 80.9]	76.2	[73.2 - 79.0]	80.0	[76.6 - 83.0]	0.11
**'19910_(ST116).fna'**	**'EN24_(ST228).fna'**	76.4	[72.4 - 80.0]	76.2	[73.2 - 79.0]	79.2	[75.7 - 82.2]	0.25
**'CVM N52662_(ST228).fna'**	**'L14_(ST330).fna'**	79.5	[75.6 - 83.0]	76.2	[73.2 - 78.9]	81.8	[78.5 - 84.8]	0.08
**'C116_(ST228).fna'**	**'CL8682_(ST769).fasta'**	75.2	[71.2 - 78.8]	76.2	[73.2 - 79.0]	78.1	[74.7 - 81.2]	0.28
**'20_SD_W_06_(ST368).fna'**	**'DSM111623_(ST624).fna'**	83.4	[79.5 - 86.6]	76.2	[73.2 - 79.0]	85.0	[81.9 - 87.8]	0.12
**'CVM_N55265_(ST228).fna'**	**'F1_(ST72).fna'**	78.3	[74.3 - 81.8]	76.2	[73.2 - 79.0]	80.8	[77.4 - 83.8]	0.09
**'CVM N52662_(ST228).fna'**	**'CVM_N54595_(ST192).fna'**	76.8	[72.8 - 80.4]	76.2	[73.2 - 79.0]	79.5	[76.1 - 82.6]	0.13
**'19910_(ST116).fna'**	**'CVM N52662_(ST228).fna'**	74.5	[70.5 - 78.1]	76.2	[73.2 - 79.0]	77.5	[74.0 - 80.6]	0.24
**'C116_(ST228).fna'**	**'CVM_N54595_(ST192).fna'**	75.8	[71.8 - 79.4]	76.2	[73.2 - 79.0]	78.6	[75.2 - 81.7]	0.19
**'19910_(ST116).fna'**	**'R48_(ST228).fna'**	73.5	[69.5 - 77.1]	76.1	[73.1 - 78.9]	76.6	[73.1 - 79.8]	0.28
**'C138_(ST228).fna'**	**'CL9797_(ST525).fasta'**	72.7	[68.7 - 76.3]	76.1	[73.1 - 78.9]	75.9	[72.4 - 79.0]	0.48
**'244_EFLS_(ST443).fna'**	**'EN24_(ST228).fna'**	76.8	[72.8 - 80.4]	76.1	[73.1 - 78.9]	79.5	[76.1 - 82.5]	0.04
**'C116_(ST228).fna'**	**'CL9797_(ST525).fasta'**	72.6	[68.7 - 76.3]	76.1	[73.1 - 78.9]	75.9	[72.4 - 79.0]	0.47
**'C138_(ST228).fna'**	**'CL11199_(ST778).fasta'**	71.0	[67.1 - 74.7]	76.1	[73.1 - 78.9]	74.4	[70.9 - 77.6]	0.41
**'CVM_N52587_(STx).fna'**	**'EnGen0400_(ST86).fna'**	79.0	[75.0 - 82.4]	76.1	[73.1 - 78.9]	81.3	[78.0 - 84.3]	0.19
**'CL9924_(ST6).fasta'**	**'CVM_N55265_(ST228).fna'**	68.8	[64.9 - 72.4]	76.1	[73.1 - 78.9]	72.4	[68.9 - 75.6]	0.44
**'4928STDY7071351_(ST30).fna'**	**'G81_(ST1468).fna'**	72.5	[68.6 - 76.2]	76.1	[73.1 - 78.9]	75.8	[72.3 - 78.9]	0.37
**'CL8682_(ST769).fasta'**	**'CVM N53420_(ST228).fna'**	74.1	[70.1 - 77.7]	76.1	[73.1 - 78.9]	77.1	[73.7 - 80.3]	0.13
**'CVM_N52587_(STx).fna'**	**'CVM_N54595_(ST192).fna'**	75.0	[71.0 - 78.6]	76.1	[73.1 - 78.9]	77.9	[74.5 - 81.0]	0.1
**'C116_(ST228).fna'**	**'CL9943_(ST4).fasta'**	69.8	[65.8 - 73.4]	76.1	[73.1 - 78.9]	73.3	[69.8 - 76.5]	0.6
**'BIOML_A5_(ST875).fna'**	**'C116_(ST228).fna'**	77.1	[73.1 - 80.7]	76.1	[73.1 - 78.9]	79.8	[76.4 - 82.8]	0.26
**'R48_(ST228).fna'**	*Enterococcus faecalis* NBRC 100480	78.7	[74.7 - 82.2]	76.1	[73.1 - 78.9]	81.1	[77.8 - 84.1]	0.04
**'CVM_N55265_(ST228).fna'**	**'EF349_(ST631).fna'**	81.2	[77.3 - 84.6]	76.1	[73.1 - 78.8]	83.3	[80.0 - 86.1]	1.04

Query	Subject	d_0	C.I. d_0	d_4	C.I. d_4	d_6	C.I. d_6	Diff. G+C Percent
**'Praia.S.M2.C4_(ST62).fasta'**	**'R48_(ST228).fna'**	76.8	[72.9 - 80.4]	76.1	[73.1 - 78.9]	79.5	[76.1 - 82.6]	0.2
**'C116_(ST228).fna'**	**'CL9924_(ST6).fasta'**	70.2	[66.2 - 73.8]	76.1	[73.1 - 78.9]	73.6	[70.2 - 76.8]	0.44
**'19910_(ST116).fna'**	**'C138_(ST228).fna'**	73.4	[69.5 - 77.1]	76.1	[73.1 - 78.9]	76.5	[73.1 - 79.7]	0.31
**'CVM_N55265_(ST228).fna'**	**'EnGen0400_(ST86).fna'**	80.5	[76.5 - 83.9]	76.1	[73.1 - 78.8]	82.6	[79.3 - 85.5]	0.1
**'CL9772_(ST525).fasta'**	**'R48_(ST228).fna'**	71.9	[67.9 - 75.5]	76.1	[73.1 - 78.9]	75.2	[71.7 - 78.4]	0.37
**'KUB3007_(ST729).fna'**	**'R48_(ST228).fna'**	73.7	[69.7 - 77.3]	76.1	[73.1 - 78.8]	76.8	[73.3 - 79.9]	0.39
**'CL6870_(ST103).fasta'**	*Aequorivita lutea* q18	12.8	[10.1 - 16.1]	76.1	[73.1 - 78.9]	13.2	[10.9 - 16.0]	5.38
**'C116_(ST228).fna'**	**'L14_(ST330).fna'**	81.3	[77.4 - 84.7]	76.1	[73.1 - 78.9]	83.3	[80.1 - 86.2]	0.02
**'CVM N52662_(ST228).fna'**	**'T11_(ST65).fna'**	81.5	[77.6 - 84.8]	76.1	[73.1 - 78.9]	83.5	[80.2 - 86.3]	0.13
**'CL9943_(ST4).fasta'**	**'CVM_N55265_(ST228).fna'**	68.9	[65.0 - 72.6]	76.1	[73.1 - 78.8]	72.5	[69.0 - 75.7]	0.61
**'CVM N52662_(ST228).fna'**	**'D32_(ST40).fna'**	72.6	[68.6 - 76.2]	76.1	[73.0 - 78.8]	75.8	[72.3 - 79.0]	0.1
**'CVM N52662_(ST228).fna'**	**'F_(ST712).fna'**	75.6	[71.6 - 79.2]	76.1	[73.1 - 78.9]	78.5	[75.0 - 81.6]	0.07
**'CL9943_(ST4).fasta'**	**'R48_(ST228).fna'**	70.0	[66.1 - 73.7]	76.1	[73.1 - 78.9]	73.5	[70.1 - 76.7]	0.58
**'BIOML_A5_(ST875).fna'**	**'R48_(ST228).fna'**	76.1	[72.1 - 79.6]	76.1	[73.1 - 78.8]	78.8	[75.4 - 81.9]	0.23
**'CL9924_(ST6).fasta'**	**'CVM N52662_(ST228).fna'**	70.5	[66.5 - 74.1]	76.1	[73.1 - 78.9]	73.9	[70.4 - 77.1]	0.38
**'CL11199_(ST778).fasta'**	**'EN24_(ST228).fna'**	70.9	[67.0 - 74.6]	76.1	[73.1 - 78.8]	74.3	[70.8 - 77.5]	0.35
**'C116_(ST228).fna'**	**'CL11199_(ST778).fasta'**	71.3	[67.3 - 74.9]	76.1	[73.1 - 78.9]	74.6	[71.1 - 77.8]	0.39
**'C138_(ST228).fna'**	**'CL9772_(ST525).fasta'**	73.5	[69.5 - 77.1]	76.1	[73.1 - 78.8]	76.6	[73.1 - 79.7]	0.4
**'EN24_(ST228).fna'**	*Enterococcus faecalis* NBRC 100480	78.6	[74.7 - 82.1]	76.1	[73.1 - 78.9]	81.1	[77.7 - 84.0]	0.02
**'C138_(ST228).fna'**	**'CL9924_(ST6).fasta'**	70.8	[66.8 - 74.4]	76.1	[73.1 - 78.9]	74.2	[70.7 - 77.4]	0.45
**'19910_(ST116).fna'**	**'C116_(ST228).fna'**	72.7	[68.7 - 76.3]	76.1	[73.1 - 78.9]	75.9	[72.4 - 79.0]	0.3
**'CVM N52662_(ST228).fna'**	**'F1_(ST72).fna'**	76.7	[72.8 - 80.3]	76.1	[73.1 - 78.9]	79.4	[76.0 - 82.5]	0.03
**'19910_(ST116).fna'**	**'CVM N53420_(ST228).fna'**	73.0	[69.0 - 76.6]	76.0	[73.0 - 78.8]	76.1	[72.7 - 79.3]	0.15
**'CL9924_(ST6).fasta'**	**'CVM_N52587_(STx).fna'**	68.2	[64.3 - 71.9]	76.0	[73.0 - 78.7]	71.9	[68.4 - 75.1]	0.35
**'C138_(ST228).fna'**	**'CL8682_(ST769).fasta'**	75.9	[71.9 - 79.5]	76.0	[73.0 - 78.7]	78.7	[75.2 - 81.8]	0.29
**'CVM N52662_(ST228).fna'**	**'EnGen0400_(ST86).fna'**	82.3	[78.5 - 85.6]	76.0	[73.0 - 78.8]	84.2	[80.9 - 86.9]	0.16
**'EN24_(ST228).fna'**	**'Merz96_(ST103).fna'**	75.0	[71.0 - 78.6]	76.0	[72.9 - 78.7]	77.9	[74.5 - 81.0]	0.07

Query	Subject	d_0	C.I. d_0	d_4	C.I. d_4	d_6	C.I. d_6	Diff. G+C Percent
**'CL9924_(ST6).fasta'**	**'EN24_(ST228).fna'**	69.8	[65.9 - 73.5]	76.0	[73.0 - 78.8]	73.3	[69.8 - 76.5]	0.39
**'CL9943_(ST4).fasta'**	**'CVM N53420_(ST228).fna'**	70.3	[66.4 - 73.9]	76.0	[73.0 - 78.8]	73.7	[70.3 - 76.9]	0.46
**'CVM_N52587_(STx).fna'**	**'D32_(ST40).fna'**	74.2	[70.2 - 77.8]	76.0	[73.0 - 78.7]	77.2	[73.7 - 80.3]	0.07
**'CVM_N54595_(ST192).fna'**	**'EN24_(ST228).fna'**	74.4	[70.5 - 78.1]	76.0	[73.0 - 78.8]	77.4	[74.0 - 80.5]	0.14
**'CVM_N55265_(ST228).fna'**	**'KUB3007_(ST729).fna'**	73.7	[69.7 - 77.3]	76.0	[72.9 - 78.7]	76.7	[73.3 - 79.9]	0.42
**'F1_(ST72).fna'**	**'R48_(ST228).fna'**	74.9	[70.9 - 78.5]	76.0	[73.0 - 78.7]	77.8	[74.4 - 80.9]	0.07
**'CL8682_(ST769).fasta'**	**'CVM_N55265_(ST228).fna'**	73.8	[69.8 - 77.4]	76.0	[73.0 - 78.7]	76.9	[73.4 - 80.0]	0.28
**'CL6870_(ST103).fasta'**	**'CVM N53420_(ST228).fna'**	74.3	[70.3 - 77.9]	76.0	[73.0 - 78.8]	77.3	[73.8 - 80.4]	0.17
**'CVM N53420_(ST228).fna'**	**'EN788_(ST82).fna'**	71.8	[67.9 - 75.5]	76.0	[73.0 - 78.8]	75.1	[71.6 - 78.3]	0.51
**'CVM N53420_(ST228).fna'**	**'F_(ST712).fna'**	74.0	[70.1 - 77.7]	76.0	[73.0 - 78.8]	77.1	[73.6 - 80.2]	0.16
**'302EA1_(ST122).fna'**	**'CVM N53420_(ST228).fna'**	74.3	[70.3 - 77.9]	76.0	[73.0 - 78.8]	77.3	[73.8 - 80.4]	0.03
**'CL9797_(ST525).fasta'**	**'CVM_N55265_(ST228).fna'**	70.5	[66.5 - 74.1]	76.0	[73.0 - 78.8]	73.9	[70.4 - 77.1]	0.47
**'14EA1_(ST147).fna'**	**'CVM N53420_(ST228).fna'**	76.2	[72.2 - 79.8]	76.0	[73.0 - 78.7]	78.9	[75.5 - 82.0]	0.33
**'CL9772_(ST525).fasta'**	**'CVM_N55265_(ST228).fna'**	72.5	[68.6 - 76.2]	75.9	[72.9 - 78.6]	75.7	[72.2 - 78.9]	0.39
**'CVM_N52587_(STx).fna'**	**'KUB3007_(ST729).fna'**	74.0	[70.0 - 77.6]	75.9	[72.9 - 78.6]	77.0	[73.6 - 80.2]	0.32
**'CVM N52662_(ST228).fna'**	**'EF349_(ST631).fna'**	79.6	[75.7 - 83.1]	75.9	[72.9 - 78.7]	81.9	[78.5 - 84.8]	1.1
**'4928STDY7071351_(ST30).fna'**	**'R48_(ST228).fna'**	73.5	[69.5 - 77.1]	75.9	[72.9 - 78.7]	76.6	[73.1 - 79.7]	0.38
**'C116_(ST228).fna'**	**'CL9772_(ST525).fasta'**	73.5	[69.5 - 77.1]	75.9	[72.9 - 78.6]	76.6	[73.1 - 79.7]	0.39
**'CVM N53420_(ST228).fna'**	**'KUB3007_(ST729).fna'**	72.2	[68.3 - 75.9]	75.9	[72.9 - 78.6]	75.4	[72.0 - 78.6]	0.27
**'C138_(ST228).fna'**	**'CL6870_(ST103).fasta'**	75.7	[71.7 - 79.3]	75.9	[72.9 - 78.7]	78.5	[75.1 - 81.6]	0.32
**'CL6870_(ST103).fasta'**	**'G81_(ST1468).fna'**	74.5	[70.5 - 78.1]	75.9	[72.9 - 78.6]	77.5	[74.0 - 80.6]	0.28
**'BIOML_A5_(ST875).fna'**	**'EN24_(ST228).fna'**	78.7	[74.8 - 82.2]	75.9	[72.9 - 78.7]	81.1	[77.7 - 84.1]	0.21
**'BIOML_A5_(ST875).fna'**	**'CVM N52662_(ST228).fna'**	76.6	[72.6 - 80.1]	75.9	[72.9 - 78.6]	79.2	[75.8 - 82.3]	0.2
**'CL11199_(ST778).fasta'**	*Aequorivita lutea* q18	12.8	[10.1 - 16.1]	75.9	[72.9 - 78.7]	13.2	[10.9 - 16.0]	5.46
**'CL9924_(ST6).fasta'**	**'CVM N53420_(ST228).fna'**	71.8	[67.9 - 75.5]	75.9	[72.9 - 78.7]	75.1	[71.6 - 78.3]	0.3
**'CL9943_(ST4).fasta'**	**'G81_(ST1468).fna'**	68.7	[64.8 - 72.4]	75.9	[72.9 - 78.6]	72.3	[68.8 - 75.5]	0.57
**'G81_(ST1468).fna'**	*Enterococcus faecalis* NBRC 100480	78.3	[74.3 - 81.8]	75.9	[72.9 - 78.7]	80.7	[77.4 - 83.7]	0.03

Query	Subject	d_0	C.I. d_0	d_4	C.I. d_4	d_6	C.I. d_6	Diff. G+C Percent
**'CL9943_(ST4).fasta'**	**'CVM N52662_(ST228).fna'**	69.7	[65.8 - 73.3]	75.9	[72.9 - 78.7]	73.2	[69.7 - 76.4]	0.54
**'CL8682_(ST769).fasta'**	**'CVM_N52587_(STx).fna'**	73.3	[69.3 - 76.9]	75.9	[72.9 - 78.7]	76.4	[72.9 - 79.5]	0.19
**'CVM_N52587_(STx).fna'**	**'EF349_(ST631).fna'**	80.6	[76.6 - 84.0]	75.9	[72.9 - 78.7]	82.7	[79.4 - 85.5]	1.14
**'CVM N53420_(ST228).fna'**	**'L14_(ST330).fna'**	76.4	[72.5 - 80.0]	75.9	[72.9 - 78.7]	79.2	[75.7 - 82.2]	0.17
**'C138_(ST228).fna'**	**'KUB3007_(ST729).fna'**	75.2	[71.2 - 78.8]	75.9	[72.9 - 78.7]	78.1	[74.6 - 81.2]	0.43
**'CVM N53420_(ST228).fna'**	**'D32_(ST40).fna'**	69.8	[65.9 - 73.5]	75.9	[72.9 - 78.6]	73.3	[69.8 - 76.5]	0.01
**'CL11199_(ST778).fasta'**	**'CVM_N55265_(ST228).fna'**	70.3	[66.4 - 74.0]	75.8	[72.8 - 78.6]	73.7	[70.2 - 76.9]	0.4
**'CL11199_(ST778).fasta'**	**'G81_(ST1468).fna'**	69.8	[65.9 - 73.4]	75.8	[72.8 - 78.6]	73.2	[69.8 - 76.4]	0.36
**'CL9772_(ST525).fasta'**	**'EN24_(ST228).fna'**	73.4	[69.4 - 77.0]	75.8	[72.8 - 78.6]	76.5	[73.0 - 79.6]	0.34
**'CL6870_(ST103).fasta'**	**'CVM_N55265_(ST228).fna'**	73.7	[69.8 - 77.4]	75.8	[72.8 - 78.6]	76.8	[73.3 - 79.9]	0.31
**'4928STDY7071351_(ST30).fna'**	**'EN24_(ST228).fna'**	73.1	[69.2 - 76.8]	75.8	[72.8 - 78.6]	76.2	[72.8 - 79.4]	0.35
**'CVM N52662_(ST228).fna'**	**'KUB3007_(ST729).fna'**	75.1	[71.1 - 78.7]	75.8	[72.8 - 78.5]	77.9	[74.5 - 81.0]	0.36
**'CL9924_(ST6).fasta'**	**'G81_(ST1468).fna'**	71.2	[67.3 - 74.9]	75.8	[72.8 - 78.5]	74.5	[71.0 - 77.7]	0.41
**'CL6870_(ST103).fasta'**	**'CVM_N52587_(STx).fna'**	73.6	[69.6 - 77.2]	75.8	[72.8 - 78.5]	76.6	[73.1 - 79.7]	0.22
**'CL8682_(ST769).fasta'**	**'EN24_(ST228).fna'**	75.4	[71.4 - 79.0]	75.8	[72.8 - 78.6]	78.2	[74.8 - 81.3]	0.23
**'CL6870_(ST103).fasta'**	**'EN24_(ST228).fna'**	75.8	[71.8 - 79.4]	75.8	[72.8 - 78.6]	78.6	[75.1 - 81.6]	0.26
**'BT00.E.21_(ST23).fasta'**	**'CVM N53420_(ST228).fna'**	70.5	[66.6 - 74.2]	75.8	[72.8 - 78.6]	73.9	[70.4 - 77.1]	0.34
**'CL9797_(ST525).fasta'**	**'CVM_N52587_(STx).fna'**	69.9	[66.0 - 73.6]	75.8	[72.8 - 78.6]	73.4	[69.9 - 76.6]	0.38
**'CL11199_(ST778).fasta'**	**'CVM N53420_(ST228).fna'**	71.2	[67.3 - 74.9]	75.8	[72.8 - 78.6]	74.5	[71.1 - 77.7]	0.25
**'CVM N52662_(ST228).fna'**	**'Merz96_(ST103).fna'**	73.5	[69.5 - 77.1]	75.8	[72.8 - 78.6]	76.6	[73.1 - 79.7]	0.08
**'CL9797_(ST525).fasta'**	**'EN24_(ST228).fna'**	71.8	[67.8 - 75.4]	75.8	[72.8 - 78.6]	75.0	[71.6 - 78.2]	0.42
**'20_SD_W_06_(ST368).fna'**	*Aequorivita lutea* q18	12.8	[10.1 - 16.1]	75.8	[72.8 - 78.6]	13.2	[10.9 - 16.0]	5.3
**'CL9943_(ST4).fasta'**	**'CVM_N52587_(STx).fna'**	68.8	[64.9 - 72.4]	75.8	[72.8 - 78.6]	72.3	[68.9 - 75.5]	0.51
**'CVM_N52587_(STx).fna'**	**'F_(ST712).fna'**	78.1	[74.2 - 81.7]	75.8	[72.8 - 78.6]	80.6	[77.2 - 83.6]	0.1
**'C116_(ST228).fna'**	**'KUB3007_(ST729).fna'**	74.3	[70.3 - 77.9]	75.8	[72.8 - 78.6]	77.3	[73.8 - 80.4]	0.41
**'CL8682_(ST769).fasta'**	**'CVM N52662_(ST228).fna'**	75.9	[71.9 - 79.5]	75.7	[72.7 - 78.5]	78.6	[75.2 - 81.7]	0.22
**'244_EFLS_(ST443).fna'**	**'CVM N53420_(ST228).fna'**	75.1	[71.1 - 78.7]	75.7	[72.7 - 78.5]	78.0	[74.5 - 81.1]	0.05

Query	Subject	d_0	C.I. d_0	d_4	C.I. d_4	d_6	C.I. d_6	Diff. G+C Percent
**'CL9772_(ST525).fasta'**	**'CVM_N52587_(STx).fna'**	71.7	[67.7 - 75.3]	75.7	[72.7 - 78.5]	74.9	[71.4 - 78.1]	0.3
**'CL9797_(ST525).fasta'**	**'G81_(ST1468).fna'**	71.6	[67.6 - 75.2]	75.7	[72.7 - 78.5]	74.8	[71.3 - 78.0]	0.44
**'CL9797_(ST525).fasta'**	**'CVM N52662_(ST228).fna'**	73.1	[69.1 - 76.7]	75.7	[72.7 - 78.5]	76.2	[72.7 - 79.3]	0.41
**'CL8682_(ST769).fasta'**	**'G81_(ST1468).fna'**	74.5	[70.6 - 78.2]	75.6	[72.6 - 78.4]	77.4	[74.0 - 80.6]	0.24
**'CL9772_(ST525).fasta'**	**'G81_(ST1468).fna'**	71.8	[67.8 - 75.4]	75.6	[72.6 - 78.3]	75.0	[71.5 - 78.2]	0.35
**'CL11199_(ST778).fasta'**	**'CVM N52662_(ST228).fna'**	72.3	[68.4 - 76.0]	75.6	[72.6 - 78.4]	75.5	[72.0 - 78.7]	0.33
**'CL11199_(ST778).fasta'**	**'CVM_N52587_(STx).fna'**	69.7	[65.8 - 73.4]	75.6	[72.6 - 78.4]	73.1	[69.7 - 76.3]	0.3
**'CL6870_(ST103).fasta'**	**'CVM N52662_(ST228).fna'**	75.5	[71.5 - 79.1]	75.6	[72.6 - 78.4]	78.2	[74.8 - 81.3]	0.25
**'15224_(ST16).fna'**	*Aequorivita lutea* q18	12.8	[10.1 - 16.1]	75.5	[72.4 - 78.2]	13.2	[10.9 - 16.0]	5.58
**'CL9772_(ST525).fasta'**	**'CVM N52662_(ST228).fna'**	74.5	[70.5 - 78.1]	75.4	[72.4 - 78.2]	77.3	[73.9 - 80.5]	0.33
**'15224_(ST16).fna'**	**'CVM N53420_(ST228).fna'**	69.7	[65.8 - 73.3]	75.4	[72.4 - 78.2]	73.1	[69.6 - 76.3]	0.37
**'4928STDY7071351_(ST30).fna'**	**'CVM N53420_(ST228).fna'**	71.6	[67.6 - 75.2]	75.4	[72.4 - 78.2]	74.8	[71.3 - 78.0]	0.26
**'CVM N53420_(ST228).fna'**	*Enterococcus faecalis* NBRC 100480	76.4	[72.4 - 79.9]	75.4	[72.4 - 78.2]	79.0	[75.5 - 82.0]	0.08
**'BIOML_A5_(ST875).fna'**	**'CVM N53420_(ST228).fna'**	77.0	[73.0 - 80.5]	75.3	[72.3 - 78.1]	79.5	[76.1 - 82.5]	0.11
**'CVM N53420_(ST228).fna'**	**'F1_(ST72).fna'**	78.1	[74.1 - 81.6]	75.3	[72.3 - 78.1]	80.4	[77.0 - 83.4]	0.06
**'F_(ST712).fna'**	**'G81_(ST1468).fna'**	73.5	[69.5 - 77.1]	75.3	[72.3 - 78.1]	76.4	[73.0 - 79.6]	0.05
**'CVM N53420_(ST228).fna'**	**'Praia.S.M2.C4_(ST62).fasta'**	74.7	[70.7 - 78.3]	75.0	[72.0 - 77.8]	77.5	[74.0 - 80.6]	0.07
**'E1_(ST40).fna'**	*Aequorivita lutea* q18	12.8	[10.1 - 16.1]	74.4	[71.4 - 77.2]	13.2	[10.9 - 16.0]	5.3
**'EF349_(ST631).fna'**	*Aequorivita lutea* q18	12.8	[10.1 - 16.1]	74.3	[71.2 - 77.1]	13.3	[10.9 - 16.0]	4.02
**'4928STDY7071435_(ST40).fna'**	*Aequorivita lutea* q18	12.8	[10.1 - 16.1]	73.7	[70.6 - 76.5]	13.2	[10.9 - 16.0]	4.93
**'R48_(ST228).fna'**	*Aequorivita lutea* q18	12.8	[10.1 - 16.1]	72.3	[69.3 - 75.1]	13.2	[10.9 - 16.0]	5.09
**'F_(ST712).fna'**	*Aequorivita lutea* q18	12.8	[10.1 - 16.1]	71.8	[68.8 - 74.7]	13.3	[10.9 - 16.0]	5.06
**'C116_(ST228).fna'**	*Aequorivita lutea* q18	12.8	[10.1 - 16.1]	71.4	[68.4 - 74.2]	13.2	[10.9 - 16.0]	5.07
**'DSM111623_(ST624).fna'**	*Aequorivita lutea* q18	12.8	[10.1 - 16.1]	70.6	[67.6 - 73.4]	13.2	[10.9 - 16.0]	5.18
**'KUB3007_(ST729).fna'**	*Aequorivita lutea* q18	12.8	[10.1 - 16.1]	70.1	[67.1 - 72.9]	13.3	[10.9 - 16.0]	5.48
**'C138_(ST228).fna'**	*Aequorivita lutea* q18	12.8	[10.1 - 16.1]	70.0	[66.9 - 72.8]	13.2	[10.9 - 16.0]	5.06
**'G81_(ST1468).fna'**	*Aequorivita lutea* q18	12.8	[10.1 - 16.1]	70.0	[67.0 - 72.8]	13.2	[10.9 - 16.0]	5.1

Query	Subject	d_0	C.I. d_0	d_4	C.I. d_4	d_6	C.I. d_6	Diff. G+C Percent
**'CVM_N52587_(STx).fna'**	*Aequorivita lutea* q18	12.8	[10.1 - 16.1]	69.9	[66.9 - 72.7]	13.2	[10.9 - 16.0]	5.16
**'D32_(ST40).fna'**	*Aequorivita lutea* q18	12.8	[10.1 - 16.1]	69.8	[66.8 - 72.6]	13.3	[10.9 - 16.0]	5.23
**'CVM N53420_(ST228).fna'**	*Aequorivita lutea* q18	12.8	[10.1 - 16.1]	69.8	[66.8 - 72.7]	13.2	[10.9 - 16.0]	5.21
**'CVM N52662_(ST228).fna'**	*Aequorivita lutea* q18	12.8	[10.1 - 16.1]	69.7	[66.7 - 72.6]	13.2	[10.9 - 16.0]	5.13
**'CVM_N55265_(ST228).fna'**	*Aequorivita lutea* q18	12.8	[10.1 - 16.1]	69.7	[66.7 - 72.6]	13.2	[10.9 - 16.0]	5.07
**'EN24_(ST228).fna'**	*Aequorivita lutea* q18	12.8	[10.1 - 16.1]	69.4	[66.4 - 72.2]	13.2	[10.9 - 16.0]	5.12
**'L14_(ST330).fna'**	*Aequorivita lutea* q18	12.8	[10.1 - 16.1]	68.7	[65.7 - 71.5]	13.3	[10.9 - 16.0]	5.05
**'14EA1_(ST147).fna'**	*Aequorivita lutea* q18	12.8	[10.1 - 16.1]	68.6	[65.6 - 71.4]	13.3	[10.9 - 16.0]	4.89
**'EnGen0400_(ST86).fna'**	*Aequorivita lutea* q18	12.8	[10.1 - 16.1]	67.2	[64.3 - 70.1]	13.3	[10.9 - 16.0]	4.97
**'EF348_(ST16).fna'**	*Arthrobacter sulfonylureivorans* LAM7117	12.6	[9.9 - 15.8]	66.9	[63.9 - 69.7]	13.0	[10.7 - 15.8]	28.43
**'302EA1_(ST122).fna'**	*Aequorivita lutea* q18	12.8	[10.1 - 16.1]	64.8	[61.9 - 67.7]	13.3	[10.9 - 16.0]	5.18
**'F1_(ST72).fna'**	*Aequorivita lutea* q18	12.8	[10.1 - 16.1]	64.8	[61.8 - 67.6]	13.3	[10.9 - 16.0]	5.16
**'209EA1_(ST624).fna'**	*Aequorivita lutea* q18	12.8	[10.1 - 16.1]	61.4	[58.6 - 64.2]	13.3	[10.9 - 16.0]	4.92
**'KUB3007_(ST729).fna'**	*Amedibacillus hominis* NSJ-176	12.8	[10.1 - 16.0]	52.6	[49.9 - 55.2]	13.2	[10.8 - 15.9]	1.64
**'LB00.E.122_(ST21).fasta'**	*Amedibacillus hominis* NSJ-176	12.6	[9.9 - 15.9]	51.6	[48.9 - 54.2]	13.0	[10.7 - 15.8]	1.81
**'CL9172_(ST21).fasta'**	*Amedibacillus hominis* NSJ-176	12.6	[9.9 - 15.9]	51.6	[48.9 - 54.2]	13.0	[10.7 - 15.8]	1.9
**'CL9314_(ST21).fasta'**	*Amedibacillus hominis* NSJ-176	12.6	[9.9 - 15.9]	51.6	[48.9 - 54.2]	13.0	[10.7 - 15.8]	1.89
**'4928STDY7071435_(ST40).fna'**	*Amedibacillus hominis* NSJ-176	12.6	[9.9 - 15.9]	50.6	[48.0 - 53.3]	13.0	[10.7 - 15.8]	2.18
**'EF348_(ST16).fna'**	*Amedibacillus hominis* NSJ-176	12.6	[10.0 - 15.9]	50.4	[47.8 - 53.0]	13.1	[10.7 - 15.8]	2.0
**'CVM N53420_(ST228).fna'**	*Amedibacillus hominis* NSJ-176	12.6	[9.9 - 15.9]	50.3	[47.7 - 52.9]	13.0	[10.7 - 15.8]	1.91
**'CVM N52662_(ST228).fna'**	*Amedibacillus hominis* NSJ-176	12.6	[9.9 - 15.9]	49.9	[47.3 - 52.5]	13.0	[10.7 - 15.8]	1.99
**'732_EFLS_(ST16).fna'**	*Amedibacillus hominis* NSJ-176	12.6	[9.9 - 15.9]	49.6	[47.0 - 52.2]	13.0	[10.7 - 15.8]	1.83
**'15224_(ST16).fna'**	*Amedibacillus hominis* NSJ-176	12.6	[9.9 - 15.9]	49.4	[46.8 - 52.0]	13.0	[10.7 - 15.8]	1.53
**'CL9924_(ST6).fasta'**	*Amedibacillus hominis* NSJ-176	12.7	[10.0 - 15.9]	49.2	[46.6 - 51.8]	13.1	[10.7 - 15.8]	1.61
**'D32_(ST40).fna'**	*Amedibacillus hominis* NSJ-176	12.6	[9.9 - 15.9]	48.6	[46.0 - 51.2]	13.0	[10.7 - 15.8]	1.89
**'E1_(ST40).fna'**	*Amedibacillus hominis* NSJ-176	12.6	[9.9 - 15.9]	48.5	[45.9 - 51.1]	13.0	[10.7 - 15.8]	1.82
**'EN242_(ST82).fna'**	*Amedibacillus hominis* NSJ-176	12.6	[9.9 - 15.9]	48.2	[45.6 - 50.8]	13.0	[10.7 - 15.8]	1.52

Query	Subject	d_0	C.I. d_0	d_4	C.I. d_4	d_6	C.I. d_6	Diff. G+C Percent
**'EN788_(ST82).fna'**	*Amedibacillus hominis* NSJ-176	12.6	[9.9 - 15.9]	48.2	[45.6 - 50.8]	13.0	[10.7 - 15.8]	1.39
**'EF349_(ST631).fna'**	*Amedibacillus hominis* NSJ-176	12.6	[9.9 - 15.9]	47.0	[44.4 - 49.6]	13.0	[10.7 - 15.8]	3.1
**'CVM_N60027F_(ST862).fna'**	*Amedibacillus hominis* NSJ-176	12.6	[9.9 - 15.9]	46.7	[44.2 - 49.3]	13.0	[10.7 - 15.8]	2.02
**'BT00.E.21_(ST23).fasta'**	*Amedibacillus hominis* NSJ-176	12.6	[9.9 - 15.9]	46.3	[43.8 - 48.9]	13.0	[10.7 - 15.8]	1.57
**'19910_(ST116).fna'**	*Amedibacillus hominis* NSJ-176	12.6	[9.9 - 15.9]	46.3	[43.8 - 48.9]	13.0	[10.7 - 15.8]	1.75
**'CL9797_(ST525).fasta'**	*Amedibacillus hominis* NSJ-176	12.7	[10.0 - 15.9]	45.3	[42.8 - 47.9]	13.1	[10.7 - 15.8]	1.58
**'CL9772_(ST525).fasta'**	*Amedibacillus hominis* NSJ-176	12.7	[10.0 - 15.9]	45.2	[42.6 - 47.8]	13.1	[10.7 - 15.8]	1.66
**'R48_(ST228).fna'**	*Amedibacillus hominis* NSJ-176	12.5	[9.9 - 15.8]	45.1	[42.5 - 47.6]	13.0	[10.6 - 15.7]	2.03
**'EnGen0400_(ST86).fna'**	*Amedibacillus hominis* NSJ-176	12.6	[10.0 - 15.9]	44.8	[42.2 - 47.4]	13.1	[10.7 - 15.8]	2.15
**'CL9314_(ST21).fasta'**	*Streptococcus gwangjuense* KCOM -1679T	12.8	[10.1 - 16.1]	43.9	[41.3 - 46.4]	13.2	[10.9 - 16.0]	2.78
**'EN24_(ST228).fna'**	*Amedibacillus hominis* NSJ-176	12.6	[9.9 - 15.9]	43.8	[41.3 - 46.4]	13.0	[10.7 - 15.7]	2.0
**'4928STDY7071435_(ST40).fna'**	*Arthrobacter sulfonylureivorans* LAM7117	12.5	[9.9 - 15.8]	43.6	[41.1 - 46.2]	13.0	[10.6 - 15.7]	28.25
**'CL6870_(ST103).fasta'**	*Amedibacillus hominis* NSJ-176	12.6	[9.9 - 15.9]	43.4	[40.9 - 46.0]	13.0	[10.7 - 15.8]	1.74
**'CL9172_(ST21).fasta'**	*Streptococcus gwangjuense* KCOM -1679T	12.8	[10.1 - 16.1]	43.4	[40.9 - 46.0]	13.2	[10.9 - 16.0]	2.77
**'CL11199_(ST778).fasta'**	*Amedibacillus hominis* NSJ-176	12.6	[9.9 - 15.9]	43.3	[40.8 - 45.9]	13.0	[10.7 - 15.8]	1.66
**'CL8682_(ST769).fasta'**	*Amedibacillus hominis* NSJ-176	12.6	[9.9 - 15.9]	42.8	[40.3 - 45.4]	13.0	[10.7 - 15.8]	1.77
**'Merz96_(ST103).fna'**	*Amedibacillus hominis* NSJ-176	12.6	[9.9 - 15.9]	42.8	[40.3 - 45.4]	13.0	[10.7 - 15.8]	2.07
**'BT00.E.21_(ST23).fasta'**	*Streptococcus gwangjuense* KCOM -1679T	12.7	[10.0 - 16.0]	42.1	[39.6 - 44.6]	13.1	[10.8 - 15.9]	3.1
**'LB00.E.122_(ST21).fasta'**	*Streptococcus gwangjuense* KCOM -1679T	12.8	[10.1 - 16.1]	41.7	[39.2 - 44.2]	13.2	[10.9 - 16.0]	2.86
**'KUB3007_(ST729).fna'**	*Streptococcus gwangjuense* KCOM -1679T	12.9	[10.2 - 16.1]	41.6	[39.1 - 44.2]	13.3	[10.9 - 16.1]	3.03
**'CL9924_(ST6).fasta'**	*Streptococcus gwangjuense* KCOM -1679T	12.8	[10.1 - 16.0]	41.5	[39.0 - 44.1]	13.2	[10.8 - 15.9]	3.06
**'732_EFLS_(ST16).fna'**	*Streptococcus gwangjuense* KCOM -1679T	12.8	[10.1 - 16.1]	40.9	[38.4 - 43.5]	13.2	[10.9 - 16.0]	2.84
**'Praia.S.M2.C4_(ST62).fasta'**	*Amedibacillus hominis* NSJ-176	12.6	[9.9 - 15.9]	40.8	[38.3 - 43.3]	13.0	[10.7 - 15.8]	1.83
**'CL9772_(ST525).fasta'**	*Streptococcus gwangjuense* KCOM -1679T	12.8	[10.1 - 16.1]	40.4	[37.9 - 42.9]	13.2	[10.9 - 16.0]	3.01

Query	Subject	d_0	C.I. d_0	d_4	C.I. d_4	d_6	C.I. d_6	Diff. G+C Percent
**'CL6870_(ST103).fasta'**	*Streptococcus gwangjuense* KCOM -1679T	12.8	[10.1 - 16.1]	40.4	[37.9 - 42.9]	13.2	[10.9 - 16.0]	2.93
**'CL8682_(ST769).fasta'**	*Streptococcus gwangjuense* KCOM -1679T	12.8	[10.1 - 16.1]	40.4	[37.9 - 42.9]	13.2	[10.9 - 16.0]	2.9
**'EF348_(ST16).fna'**	*Streptococcus gwangjuense* KCOM -1679T	12.8	[10.1 - 16.1]	40.2	[37.7 - 42.7]	13.2	[10.9 - 16.0]	2.67
**'Merz96_(ST103).fna'**	*Streptococcus gwangjuense* KCOM -1679T	12.8	[10.1 - 16.1]	40.0	[37.5 - 42.6]	13.2	[10.9 - 16.0]	2.6
**'CL11199_(ST778).fasta'**	*Streptococcus gwangjuense* KCOM -1679T	12.8	[10.1 - 16.1]	40.0	[37.5 - 42.6]	13.2	[10.9 - 16.0]	3.01
**'BT00.E.21_(ST23).fasta'**	*Arthrobacter sulfonylureivorans* LAM7117	12.5	[9.9 - 15.8]	40.0	[37.5 - 42.5]	12.9	[10.6 - 15.7]	28.87
**'EN788_(ST82).fna'**	*Streptococcus gwangjuense* KCOM -1679T	12.8	[10.1 - 16.0]	39.8	[37.3 - 42.3]	13.2	[10.8 - 15.9]	3.28
**'CVM N53420_(ST228).fna'**	*Streptococcus gwangjuense* KCOM -1679T	12.8	[10.1 - 16.1]	39.8	[37.3 - 42.3]	13.2	[10.8 - 16.0]	2.76
**'15224_(ST16).fna'**	*Streptococcus gwangjuense* KCOM -1679T	12.8	[10.1 - 16.0]	39.8	[37.3 - 42.3]	13.2	[10.8 - 15.9]	3.14
**'CL9797_(ST525).fasta'**	*Streptococcus gwangjuense* KCOM -1679T	12.8	[10.1 - 16.1]	39.6	[37.2 - 42.2]	13.2	[10.9 - 16.0]	3.09
**'CVM N52662_(ST228).fna'**	*Streptococcus gwangjuense* KCOM -1679T	12.8	[10.1 - 16.1]	39.5	[37.1 - 42.1]	13.2	[10.9 - 16.0]	2.68
**'EN242_(ST82).fna'**	*Streptococcus gwangjuense* KCOM -1679T	12.8	[10.1 - 16.0]	39.3	[36.9 - 41.9]	13.2	[10.8 - 15.9]	3.15
**'P9 CL A7_(ST4).fna'**	*Streptococcus gwangjuense* KCOM -1679T	12.6	[9.9 - 15.9]	39.0	[36.6 - 41.6]	13.0	[10.7 - 15.8]	3.16
**'4928STDY7071435_(ST 40).fna'**	*Streptococcus gwangjuense* KCOM -1679T	12.8	[10.1 - 16.0]	38.7	[36.2 - 41.2]	13.2	[10.8 - 15.9]	2.49
**'EN242_(ST82).fna'**	*Enterococcus gallinarum* NBRC 100675	13.9	[11.1 - 17.2]	38.1	[35.6 - 40.6]	14.3	[11.9 - 17.1]	2.7
**'EN788_(ST82).fna'**	*Enterococcus gallinarum* NBRC 100675	13.8	[11.1 - 17.2]	38.1	[35.6 - 40.6]	14.3	[11.9 - 17.1]	2.82
**'E1_(ST40).fna'**	*Streptococcus gwangjuense* KCOM -1679T	12.8	[10.1 - 16.1]	37.9	[35.5 - 40.5]	13.2	[10.9 - 16.0]	2.85
**'EnGen0400_(ST86).fna'**	*Streptococcus gwangjuense* KCOM -1679T	12.9	[10.2 - 16.1]	37.8	[35.3 - 40.3]	13.3	[10.9 - 16.0]	2.52
**'R48_(ST228).fna'**	*Arthrobacter sulfonylureivorans* LAM7117	12.5	[9.9 - 15.8]	37.0	[34.5 - 39.5]	12.9	[10.6 - 15.7]	28.41
**'CVM_N60027F_(ST862). fna'**	*Streptococcus gwangjuense* KCOM -1679T	12.8	[10.1 - 16.0]	36.9	[34.5 - 39.5]	13.2	[10.8 - 15.9]	2.65
**'CL6870_(ST103).fasta'**	*Enterococcus raffinosus* NBRC 100492	13.3	[10.6 - 16.6]	36.9	[34.5 - 39.5]	13.7	[11.3 - 16.5]	2.08

Query	Subject	d_0	C.I. d_0	d_4	C.I. d_4	d_6	C.I. d_6	Diff. G+C Percent
**'19910_(ST116).fna'**	*Streptococcus gwangjuense* KCOM -1679T	12.8	[10.1 - 16.0]	36.8	[34.4 - 39.3]	13.2	[10.8 - 15.9]	2.92
**'Praia.S.M2.C4_(ST62).fasta'**	*Streptococcus gwangjuense* KCOM -1679T	12.8	[10.1 - 16.0]	36.8	[34.3 - 39.3]	13.2	[10.8 - 15.9]	2.84
**'CL11199_(ST778).fasta'**	*Enterococcus raffinosus* NBRC 100492	13.3	[10.5 - 16.6]	36.6	[34.2 - 39.1]	13.7	[11.3 - 16.5]	2.17
**'C116_(ST228).fna'**	*Arthrobacter sulfonylureivorans* LAM7117	12.5	[9.9 - 15.8]	36.3	[33.9 - 38.8]	12.9	[10.6 - 15.7]	28.38
**'Merz96_(ST103).fna'**	*Enterococcus raffinosus* NBRC 100492	13.2	[10.5 - 16.5]	36.3	[33.9 - 38.8]	13.6	[11.3 - 16.4]	1.76
**'DSM111623_(ST624).fna'**	*Arthrobacter sulfonylureivorans* LAM7117	12.5	[9.9 - 15.8]	36.1	[33.7 - 38.6]	12.9	[10.6 - 15.7]	28.5
**'302EA1_(ST122).fna'**	*Arthrobacter sulfonylureivorans* LAM7117	12.6	[9.9 - 15.8]	36.0	[33.5 - 38.5]	13.0	[10.7 - 15.7]	28.5
**'EN24_(ST228).fna'**	*Streptococcus gwangjuense* KCOM -1679T	12.8	[10.1 - 16.0]	35.6	[33.1 - 38.1]	13.2	[10.8 - 15.9]	2.67
**'CL8682_(ST769).fasta'**	*Enterococcus raffinosus* NBRC 100492	13.2	[10.5 - 16.5]	35.6	[33.2 - 38.1]	13.7	[11.3 - 16.4]	2.05
**'209EA1_(ST624).fna'**	*Arthrobacter sulfonylureivorans* LAM7117	12.6	[9.9 - 15.8]	35.5	[33.1 - 38.1]	13.0	[10.7 - 15.7]	28.24
**'F_(ST712).fna'**	*Arthrobacter sulfonylureivorans* LAM7117	12.6	[9.9 - 15.8]	35.4	[33.0 - 37.9]	13.0	[10.6 - 15.7]	28.37
**'F1_(ST72).fna'**	*Arthrobacter sulfonylureivorans* LAM7117	12.6	[9.9 - 15.9]	35.4	[33.0 - 37.9]	13.0	[10.7 - 15.8]	28.47
**'EnGen0400_(ST86).fna'**	*Arthrobacter sulfonylureivorans* LAM7117	12.6	[9.9 - 15.8]	35.3	[32.9 - 37.8]	13.0	[10.7 - 15.7]	28.29
**'D32_(ST40).fna'**	*Arthrobacter sulfonylureivorans* LAM7117	12.6	[9.9 - 15.8]	35.3	[32.9 - 37.9]	13.0	[10.6 - 15.7]	28.54
**'L14_(ST330).fna'**	*Arthrobacter sulfonylureivorans* LAM7117	12.6	[9.9 - 15.8]	35.2	[32.7 - 37.7]	13.0	[10.6 - 15.7]	28.36
**'KUB3007_(ST729).fna'**	*Arthrobacter sulfonylureivorans* LAM7117	12.6	[9.9 - 15.8]	35.2	[32.7 - 37.7]	13.0	[10.6 - 15.7]	28.8
**'P9 CL A7_(ST4).fna'**	*Enterococcus gallinarum* NBRC 100675	13.9	[11.1 - 17.2]	35.1	[32.7 - 37.7]	14.3	[11.9 - 17.1]	2.71
**'14EA1_(ST147).fna'**	*Arthrobacter sulfonylureivorans* LAM7117	12.6	[9.9 - 15.8]	35.1	[32.6 - 37.6]	13.0	[10.7 - 15.7]	28.2
**'15224_(ST16).fna'**	*Arthrobacter sulfonylureivorans* LAM7117	12.5	[9.9 - 15.8]	35.0	[32.5 - 37.5]	12.9	[10.6 - 15.7]	28.9
**'G81_(ST1468).fna'**	*Arthrobacter sulfonylureivorans* LAM7117	12.5	[9.9 - 15.8]	34.9	[32.4 - 37.4]	12.9	[10.6 - 15.7]	28.42
**'CVM_N60027F_(ST862).fna'**	*Arthrobacter sulfonylureivorans* LAM7117	12.5	[9.9 - 15.8]	34.9	[32.5 - 37.4]	12.9	[10.6 - 15.7]	28.42
**'CVM_N54595_(ST192).fna'**	*Arthrobacter sulfonylureivorans* LAM7117	12.5	[9.9 - 15.8]	34.8	[32.4 - 37.3]	12.9	[10.6 - 15.7]	28.57

Query	Subject	d_0	C.I. d_0	d_4	C.I. d_4	d_6	C.I. d_6	Diff. G+C Percent
**'C138_(ST228).fna'**	*Arthrobacter sulfonylureivorans* LAM7117	12.5	[9.9 - 15.8]	34.8	[32.4 - 37.3]	12.9	[10.6 - 15.7]	28.37
**'19910_(ST116).fna'**	*Arthrobacter sulfonylureivorans* LAM7117	12.5	[9.9 - 15.8]	34.8	[32.4 - 37.3]	12.9	[10.6 - 15.7]	28.68
**'EN242_(ST82).fna'**	*Arthrobacter sulfonylureivorans* LAM7117	12.5	[9.9 - 15.8]	34.7	[32.2 - 37.2]	12.9	[10.6 - 15.7]	28.92
**'732_EFLS_(ST16).fna'**	*Arthrobacter sulfonylureivorans* LAM7117	12.5	[9.9 - 15.8]	34.7	[32.3 - 37.3]	12.9	[10.6 - 15.7]	28.6
**'4928STDY7071351_(ST30).fna'**	*Arthrobacter sulfonylureivorans* LAM7117	12.5	[9.9 - 15.8]	34.7	[32.2 - 37.2]	12.9	[10.6 - 15.7]	28.79
**'EN788_(ST82).fna'**	*Arthrobacter sulfonylureivorans* LAM7117	12.5	[9.9 - 15.8]	34.7	[32.2 - 37.2]	12.9	[10.6 - 15.7]	29.04
**'E1_(ST40).fna'**	*Arthrobacter sulfonylureivorans* LAM7117	12.5	[9.9 - 15.8]	34.7	[32.3 - 37.2]	12.9	[10.6 - 15.7]	28.62
**'BIOML_A5_(ST875).fna'**	*Arthrobacter sulfonylureivorans* LAM7117	12.5	[9.9 - 15.8]	34.6	[32.2 - 37.1]	12.9	[10.6 - 15.7]	28.64
**'CL9772_(ST525).fasta'**	*Arthrobacter sulfonylureivorans* LAM7117	12.5	[9.9 - 15.8]	34.6	[32.1 - 37.1]	12.9	[10.6 - 15.7]	28.77
**'LB00.E.122_(ST21).fasta'**	*Arthrobacter sulfonylureivorans* LAM7117	12.5	[9.9 - 15.8]	34.6	[32.1 - 37.1]	12.9	[10.6 - 15.7]	28.63
**'T11_(ST65).fna'**	*Arthrobacter sulfonylureivorans* LAM7117	12.5	[9.9 - 15.8]	34.6	[32.2 - 37.1]	12.9	[10.6 - 15.7]	28.31
**'244_EFLS_(ST443).fna'**	*Arthrobacter sulfonylureivorans* LAM7117	12.5	[9.9 - 15.8]	34.6	[32.1 - 37.1]	12.9	[10.6 - 15.7]	28.48
**'CL9314_(ST21).fasta'**	*Arthrobacter sulfonylureivorans* LAM7117	12.5	[9.9 - 15.8]	34.6	[32.1 - 37.1]	12.9	[10.6 - 15.7]	28.55
**'CL9172_(ST21).fasta'**	*Arthrobacter sulfonylureivorans* LAM7117	12.5	[9.9 - 15.8]	34.6	[32.2 - 37.1]	12.9	[10.6 - 15.7]	28.54
**'CL6870_(ST103).fasta'**	*Arthrobacter sulfonylureivorans* LAM7117	12.5	[9.9 - 15.8]	34.6	[32.1 - 37.1]	12.9	[10.6 - 15.7]	28.69
**'CL11199_(ST778).fasta'**	*Arthrobacter sulfonylureivorans* LAM7117	12.5	[9.9 - 15.8]	34.6	[32.1 - 37.1]	12.9	[10.6 - 15.7]	28.78
**'CL9797_(ST525).fasta'**	*Arthrobacter sulfonylureivorans* LAM7117	12.5	[9.9 - 15.8]	34.6	[32.1 - 37.1]	12.9	[10.6 - 15.7]	28.86
**'Merz96_(ST103).fna'**	*Arthrobacter sulfonylureivorans* LAM7117	12.5	[9.9 - 15.8]	34.6	[32.1 - 37.1]	12.9	[10.6 - 15.7]	28.37
**'CL9824_(ST631).fasta'**	*Arthrobacter sulfonylureivorans* LAM7117	12.5	[9.9 - 15.8]	34.6	[32.2 - 37.1]	12.9	[10.6 - 15.7]	28.26
**'CL9943_(ST4).fasta'**	*Arthrobacter sulfonylureivorans* LAM7117	12.5	[9.9 - 15.8]	34.5	[32.1 - 37.0]	12.9	[10.6 - 15.7]	28.99
**'Praia.S.M2.C4_(ST62).fasta'**	*Arthrobacter sulfonylureivorans* LAM7117	12.5	[9.9 - 15.8]	34.5	[32.1 - 37.0]	12.9	[10.6 - 15.7]	28.6

Query	Subject	d_0	C.I. d_0	d_4	C.I. d_4	d_6	C.I. d_6	Diff. G+C Percent
**'CL8682_(ST769).fasta'**	*Arthrobacter sulfonylureivorans* LAM7117	12.5	[9.9 - 15.8]	34.4	[32.0 - 37.0]	12.9	[10.6 - 15.7]	28.66
**'20_SD_W_06_(ST368).fna'**	*Arthrobacter sulfonylureivorans* LAM7117	12.5	[9.9 - 15.8]	34.4	[32.0 - 36.9]	12.9	[10.6 - 15.7]	28.62
**'CL9797_(ST525).fasta'**	*Enterococcus raffinosus* NBRC 100492	13.2	[10.5 - 16.6]	34.4	[32.0 - 37.0]	13.7	[11.3 - 16.5]	2.25
**'CL9772_(ST525).fasta'**	*Enterococcus raffinosus* NBRC 100492	13.2	[10.5 - 16.6]	34.2	[31.8 - 36.7]	13.7	[11.3 - 16.5]	2.16
**'F_(ST712).fna'**	*Enterococcus durans* NBRC 100479	13.7	[11.0 - 17.1]	34.2	[31.8 - 36.7]	14.2	[11.7 - 17.0]	0.2
**'CL9924_(ST6).fasta'**	*Arthrobacter sulfonylureivorans* LAM7117	12.5	[9.9 - 15.8]	34.2	[31.7 - 36.7]	12.9	[10.6 - 15.7]	28.83
**'CVM N53420_(ST228).fna'**	*Arthrobacter sulfonylureivorans* LAM7117	12.5	[9.9 - 15.8]	34.0	[31.5 - 36.5]	12.9	[10.6 - 15.7]	28.53
**'CVM_N52587_(STx).fna'**	*Arthrobacter sulfonylureivorans* LAM7117	12.5	[9.9 - 15.8]	34.0	[31.6 - 36.5]	12.9	[10.6 - 15.7]	28.48
**'EF349_(ST631).fna'**	*Streptococcus gwangjuense* KCOM -1679T	12.7	[10.0 - 16.0]	34.0	[31.6 - 36.5]	13.1	[10.8 - 15.9]	1.57
**'BT00.E.21_(ST23).fasta'**	*Enterococcus raffinosus* NBRC 100492	13.3	[10.6 - 16.6]	34.0	[31.5 - 36.5]	13.7	[11.4 - 16.5]	2.26
**'CVM N52662_(ST228).fna'**	*Arthrobacter sulfonylureivorans* LAM7117	12.5	[9.9 - 15.8]	33.9	[31.4 - 36.4]	12.9	[10.6 - 15.7]	28.44
**'CVM_N55265_(ST228).fna'**	*Arthrobacter sulfonylureivorans* LAM7117	12.5	[9.9 - 15.8]	33.9	[31.5 - 36.5]	12.9	[10.6 - 15.7]	28.38
**'EN24_(ST228).fna'**	*Arthrobacter sulfonylureivorans* LAM7117	12.5	[9.9 - 15.8]	33.9	[31.5 - 36.5]	12.9	[10.6 - 15.7]	28.43
**'15224_(ST16).fna'**	*Enterococcus gallinarum* NBRC 100675	13.5	[10.7 - 16.8]	33.6	[31.2 - 36.1]	13.9	[11.5 - 16.7]	2.68
**'F1_(ST72).fna'**	*Enterococcus durans* NBRC 100479	13.6	[10.9 - 17.0]	33.6	[31.2 - 36.2]	14.0	[11.6 - 16.9]	0.3
**'4928STDY7071351_(ST30).fna'**	*Enterococcus durans* NBRC 100479	13.5	[10.7 - 16.8]	33.4	[31.0 - 35.9]	13.9	[11.5 - 16.7]	0.61
**'CL9943_(ST4).fasta'**	*Amedibacillus hominis* NSJ-176	12.5	[9.9 - 15.8]	33.4	[30.9 - 35.9]	12.9	[10.6 - 15.7]	1.45
**'F_(ST712).fna'**	*Amedibacillus hominis* NSJ-176	12.5	[9.9 - 15.8]	33.3	[30.9 - 35.8]	12.9	[10.6 - 15.7]	2.06
**'CL11199_(ST778).fasta'**	*Enterococcus gallinarum* NBRC 100675	13.5	[10.7 - 16.8]	33.3	[30.9 - 35.8]	13.9	[11.5 - 16.7]	2.56
**'4928STDY7071351_(ST30).fna'**	*Enterococcus raffinosus* NBRC 100492	13.3	[10.5 - 16.6]	33.2	[30.7 - 35.7]	13.7	[11.3 - 16.5]	2.18
**'G81_(ST1468).fna'**	*Amedibacillus hominis* NSJ-176	12.5	[9.9 - 15.8]	33.1	[30.7 - 35.6]	12.9	[10.6 - 15.7]	2.01
**'L14_(ST330).fna'**	*Amedibacillus hominis* NSJ-176	12.6	[9.9 - 15.8]	33.0	[30.6 - 35.5]	13.0	[10.6 - 15.7]	2.07
**'CL9797_(ST525).fasta'**	*Enterococcus gallinarum* NBRC 100675	13.5	[10.8 - 16.9]	32.8	[30.4 - 35.3]	13.9	[11.5 - 16.7]	2.64
**'EF348_(ST16).fna'**	*Enterococcus gallinarum* NBRC 100675	13.5	[10.8 - 16.9]	32.7	[30.3 - 35.2]	13.9	[11.5 - 16.7]	2.22

Query	Subject	d_0	C.I. d_0	d_4	C.I. d_4	d_6	C.I. d_6	Diff. G+C Percent
**'F1_(ST72).fna'**	*Enterococcus italicus* DSM 15952	13.5	[10.7 - 16.8]	32.7	[30.3 - 35.2]	13.9	[11.5 - 16.7]	1.65
**'302EA1_(ST122).fna'**	*Amedibacillus hominis* NSJ-176	12.6	[9.9 - 15.8]	32.6	[30.2 - 35.1]	13.0	[10.6 - 15.7]	1.93
**'4928STDY7071351_(ST30).fna'**	*Enterococcus lactis* DSM 23655	13.4	[10.7 - 16.7]	32.6	[30.2 - 35.1]	13.8	[11.4 - 16.6]	0.95
**'4928STDY7071351_(ST30).fna'**	*Amedibacillus hominis* NSJ-176	12.5	[9.9 - 15.8]	32.6	[30.2 - 35.1]	12.9	[10.6 - 15.7]	1.65
**'F_(ST712).fna'**	*Enterococcus italicus* DSM 15952	13.8	[11.0 - 17.1]	32.6	[30.1 - 35.1]	14.2	[11.8 - 17.0]	1.55
**'CL9943_(ST4).fasta'**	*Enterococcus raffinosus* NBRC 100492	13.3	[10.5 - 16.6]	32.5	[30.1 - 35.0]	13.7	[11.3 - 16.5]	2.38
**'4928STDY7071351_(ST30).fna'**	*Enterococcus lactis* CCM 8412	13.4	[10.7 - 16.7]	32.4	[30.0 - 34.9]	13.8	[11.4 - 16.6]	0.94
**'F1_(ST72).fna'**	*Enterococcus lactis* CCM 8412	13.5	[10.8 - 16.8]	32.4	[30.0 - 34.9]	13.9	[11.5 - 16.7]	0.63
**'CL9943_(ST4).fasta'**	*Enterococcus gallinarum* NBRC 100675	13.5	[10.8 - 16.9]	32.4	[30.0 - 34.9]	13.9	[11.5 - 16.7]	2.77
**'CL9924_(ST6).fasta'**	*Enterococcus raffinosus* NBRC 100492	13.3	[10.6 - 16.7]	32.4	[30.0 - 34.9]	13.8	[11.4 - 16.6]	2.22
**'F1_(ST72).fna'**	*Enterococcus lactis* DSM 23655	13.5	[10.7 - 16.8]	32.3	[29.9 - 34.8]	13.9	[11.5 - 16.7]	0.64
**'F_(ST712).fna'**	*Enterococcus lactis* CCM 8412	13.5	[10.7 - 16.8]	32.3	[29.8 - 34.8]	13.9	[11.5 - 16.7]	0.53
**'F_(ST712).fna'**	*Enterococcus massiliensis* AM1	13.6	[10.8 - 16.9]	32.2	[29.8 - 34.8]	14.0	[11.6 - 16.8]	2.05
**'244_EFLS_(ST443).fna'**	*Amedibacillus hominis* NSJ-176	12.5	[9.9 - 15.8]	32.2	[29.8 - 34.7]	12.9	[10.6 - 15.7]	1.96
**'F_(ST712).fna'**	*Enterococcus lactis* DSM 23655	13.5	[10.7 - 16.8]	32.2	[29.8 - 34.7]	13.9	[11.5 - 16.7]	0.54
**'R48_(ST228).fna'**	*Streptococcus gwangjuense* KCOM -1679T	12.7	[10.0 - 15.9]	32.1	[29.7 - 34.6]	13.1	[10.7 - 15.8]	2.64
**'CVM_N52587_(STx).fna'**	*Amedibacillus hominis* NSJ-176	12.5	[9.9 - 15.8]	32.0	[29.6 - 34.5]	12.9	[10.6 - 15.7]	1.96
**'F1_(ST72).fna'**	*Amedibacillus hominis* NSJ-176	12.6	[9.9 - 15.8]	31.9	[29.5 - 34.4]	13.0	[10.7 - 15.7]	1.96
**'E1_(ST40).fna'**	*Enterococcus gallinarum* NBRC 100675	13.3	[10.6 - 16.6]	31.9	[29.5 - 34.5]	13.7	[11.3 - 16.5]	2.4
**'DSM111623_(ST624).fna'**	*Amedibacillus hominis* NSJ-176	12.5	[9.9 - 15.8]	31.8	[29.4 - 34.3]	12.9	[10.6 - 15.7]	1.93
**'14EA1_(ST147).fna'**	*Amedibacillus hominis* NSJ-176	12.6	[9.9 - 15.8]	31.7	[29.3 - 34.2]	13.0	[10.6 - 15.7]	2.23
**'Praia.S.M2.C4_(ST62).fasta'**	*Enterococcus raffinosus* NBRC 100492	13.3	[10.6 - 16.7]	31.7	[29.3 - 34.2]	13.7	[11.4 - 16.5]	1.99
**'209EA1_(ST624).fna'**	*Amedibacillus hominis* NSJ-176	12.6	[9.9 - 15.8]	31.5	[29.1 - 34.0]	13.0	[10.6 - 15.7]	2.2
**'E1_(ST40).fna'**	*Enterococcus lactis* CCM 8412	13.4	[10.6 - 16.7]	31.5	[29.0 - 34.0]	13.8	[11.4 - 16.6]	0.77
**'E1_(ST40).fna'**	*Enterococcus lactis* DSM 23655	13.4	[10.6 - 16.7]	31.4	[29.0 - 33.9]	13.8	[11.4 - 16.6]	0.78
**'C116_(ST228).fna'**	*Amedibacillus hominis* NSJ-176	12.5	[9.9 - 15.8]	31.3	[28.9 - 33.8]	12.9	[10.6 - 15.7]	2.05
**'EN788_(ST82).fna'**	*Enterococcus cecorum* DSM 20682	13.3	[10.6 - 16.7]	31.2	[28.8 - 33.7]	13.7	[11.4 - 16.5]	0.55

Query	Subject	d_0	C.I. d_0	d_4	C.I. d_4	d_6	C.I. d_6	Diff. G+C Percent
**'244_EFLS_(ST443).fna'**	*Enterococcus durans* NBRC 100479	13.5	[10.7 - 16.8]	31.1	[28.7 - 33.6]	13.9	[11.5 - 16.7]	0.3
**'CL9172_(ST21).fasta'**	*Enterococcus durans* NBRC 100479	13.4	[10.6 - 16.7]	31.1	[28.7 - 33.6]	13.8	[11.4 - 16.6]	0.36
**'LB00.E.122_(ST21).fasta'**	*Enterococcus durans* NBRC 100479	13.4	[10.6 - 16.7]	31.1	[28.7 - 33.6]	13.8	[11.4 - 16.6]	0.45
**'4928STDY7071435_(ST40).fna'**	*Enterococcus lactis* DSM 23655	13.4	[10.6 - 16.7]	31.0	[28.6 - 33.5]	13.8	[11.4 - 16.6]	0.41
**'4928STDY7071435_(ST40).fna'**	*Enterococcus lactis* CCM 8412	13.4	[10.6 - 16.7]	31.0	[28.6 - 33.5]	13.8	[11.4 - 16.6]	0.41
**'LB00.E.122_(ST21).fasta'**	*Enterococcus lactis* DSM 23655	13.4	[10.6 - 16.7]	30.7	[28.3 - 33.2]	13.8	[11.4 - 16.6]	0.79
**'LB00.E.122_(ST21).fasta'**	*Enterococcus lactis* CCM 8412	13.4	[10.6 - 16.7]	30.7	[28.3 - 33.2]	13.8	[11.4 - 16.6]	0.78
**'C138_(ST228).fna'**	*Amedibacillus hominis* NSJ-176	12.5	[9.9 - 15.8]	30.7	[28.3 - 33.2]	12.9	[10.6 - 15.7]	2.06
**'CL9172_(ST21).fasta'**	*Enterococcus lactis* DSM 23655	13.4	[10.7 - 16.7]	30.7	[28.3 - 33.2]	13.8	[11.4 - 16.6]	0.7
**'Praia.S.M2.C4_(ST62).fasta'**	*Enterococcus durans* NBRC 100479	13.6	[10.8 - 16.9]	30.7	[28.3 - 33.2]	14.0	[11.6 - 16.8]	0.43
**'Praia.S.M2.C4_(ST62).fasta'**	*Enterococcus saigonensis* JCM 31193	13.5	[10.7 - 16.8]	30.7	[28.3 - 33.2]	13.9	[11.5 - 16.7]	1.09
**'CL9172_(ST21).fasta'**	*Enterococcus lactis* CCM 8412	13.4	[10.7 - 16.7]	30.6	[28.2 - 33.1]	13.8	[11.4 - 16.6]	0.69
**'F1_(ST72).fna'**	*Enterococcus massiliensis* AM1	13.5	[10.7 - 16.8]	30.6	[28.2 - 33.1]	13.9	[11.5 - 16.7]	2.15
**'Praia.S.M2.C4_(ST62).fasta'**	*Enterococcus devriesei* DSM 22802	13.3	[10.6 - 16.6]	30.6	[28.2 - 33.1]	13.7	[11.3 - 16.5]	2.86
**'EN242_(ST82).fna'**	*Enterococcus cecorum* DSM 20682	13.2	[10.5 - 16.5]	30.6	[28.2 - 33.1]	13.6	[11.3 - 16.4]	0.68
**'CL9943_(ST4).fasta'**	*Enterococcus durans* NBRC 100479	13.4	[10.7 - 16.8]	30.5	[28.1 - 33.0]	13.8	[11.5 - 16.6]	0.81
**'CVM N53420_(ST228).fna'**	*Vagococcus martis* D7T301	13.1	[10.4 - 16.4]	30.3	[27.9 - 32.8]	13.5	[11.1 - 16.3]	3.68
**'E1_(ST40).fna'**	*Enterococcus pseudoavium* NBRC 100491	13.6	[10.9 - 17.0]	30.2	[27.8 - 32.7]	14.0	[11.6 - 16.8]	2.72
**'KUB3007_(ST729).fna'**	*Vagococcus martis* D7T301	13.2	[10.5 - 16.5]	30.2	[27.8 - 32.7]	13.6	[11.3 - 16.4]	3.41
**'CL9314_(ST21).fasta'**	*Enterococcus lactis* DSM 23655	13.4	[10.6 - 16.7]	30.2	[27.8 - 32.7]	13.8	[11.4 - 16.6]	0.71
**'BT00.E.21_(ST23).fasta'**	*Enterococcus lactis* CCM 8412	13.3	[10.5 - 16.6]	30.1	[27.7 - 32.6]	13.7	[11.3 - 16.5]	1.02
**'CL9314_(ST21).fasta'**	*Enterococcus lactis* CCM 8412	13.4	[10.6 - 16.7]	30.1	[27.7 - 32.6]	13.8	[11.4 - 16.6]	0.71
**'CVM N52662_(ST228).fna'**	*Vagococcus martis* D7T301	13.1	[10.4 - 16.4]	30.1	[27.7 - 32.6]	13.5	[11.1 - 16.3]	3.77
**'4928STDY7071351_(ST30).fna'**	*Enterococcus massiliensis* AM1	13.6	[10.8 - 16.9]	30.0	[27.6 - 32.5]	14.0	[11.6 - 16.8]	2.46
**'EnGen0400_(ST86).fna'**	*Vagococcus martis* D7T301	13.2	[10.5 - 16.5]	30.0	[27.6 - 32.5]	13.6	[11.2 - 16.4]	3.92
**'CL9314_(ST21).fasta'**	*Enterococcus durans* NBRC 100479	13.3	[10.6 - 16.6]	29.9	[27.5 - 32.4]	13.7	[11.3 - 16.5]	0.37
**'14EA1_(ST147).fna'**	*Enterococcus durans* NBRC 100479	13.5	[10.7 - 16.8]	29.9	[27.5 - 32.4]	13.9	[11.5 - 16.7]	0.03

Query	Subject	d_0	C.I. d_0	d_4	C.I. d_4	d_6	C.I. d_6	Diff. G+C Percent
**'BT00.E.21_(ST23).fasta'**	*Enterococcus lactis* DSM 23655	13.3	[10.5 - 16.6]	29.9	[27.5 - 32.4]	13.7	[11.3 - 16.5]	1.03
**'CVM_N55265_(ST228).fna'**	*Amedibacillus hominis* NSJ-176	12.5	[9.9 - 15.8]	29.8	[27.5 - 32.4]	12.9	[10.6 - 15.7]	2.05
**'T11_(ST65).fna'**	*Amedibacillus hominis* NSJ-176	12.5	[9.9 - 15.8]	29.8	[27.4 - 32.3]	12.9	[10.6 - 15.7]	2.12
**'302EA1_(ST122).fna'**	*Streptococcus gwangjuense* KCOM -1679T	12.7	[10.0 - 16.0]	29.8	[27.4 - 32.3]	13.1	[10.8 - 15.9]	2.74
**'CVM_N54595_(ST192).fna'**	*Amedibacillus hominis* NSJ-176	12.5	[9.9 - 15.8]	29.7	[27.3 - 32.2]	12.9	[10.6 - 15.7]	1.86
**'302EA1_(ST122).fna'**	*Enterococcus durans* NBRC 100479	13.4	[10.7 - 16.7]	29.7	[27.3 - 32.2]	13.8	[11.4 - 16.6]	0.32
**'20_SD_W_06_(ST368).fna'**	*Amedibacillus hominis* NSJ-176	12.5	[9.9 - 15.8]	29.7	[27.4 - 32.2]	12.9	[10.6 - 15.7]	1.81
**'CL9824_(ST631).fasta'**	*Amedibacillus hominis* NSJ-176	12.5	[9.9 - 15.8]	29.7	[27.3 - 32.2]	12.9	[10.6 - 15.7]	2.17
**'BIOML_A5_(ST875).fna'**	*Amedibacillus hominis* NSJ-176	12.5	[9.9 - 15.8]	29.7	[27.3 - 32.2]	12.9	[10.6 - 15.7]	1.8
**'244_EFLS_(ST443).fna'**	*Enterococcus italicus* DSM 15952	13.4	[10.7 - 16.7]	29.6	[27.2 - 32.1]	13.8	[11.4 - 16.6]	1.66
**'BT00.E.21_(ST23).fasta'**	*Enterococcus devriesei* DSM 22802	13.2	[10.5 - 16.5]	29.5	[27.2 - 32.0]	13.6	[11.3 - 16.4]	3.13
**'CL9924_(ST6).fasta'**	*Enterococcus durans* NBRC 100479	13.4	[10.6 - 16.7]	29.5	[27.1 - 32.0]	13.8	[11.4 - 16.6]	0.65
**'BT00.E.21_(ST23).fasta'**	*Enterococcus durans* NBRC 100479	13.3	[10.6 - 16.6]	29.4	[27.0 - 31.9]	13.7	[11.3 - 16.5]	0.69
**'E1_(ST40).fna'**	*Enterococcus durans* NBRC 100479	13.3	[10.6 - 16.7]	29.3	[26.9 - 31.8]	13.7	[11.4 - 16.5]	0.44
**'Praia.S.M2.C4_(ST62).fasta'**	*Enterococcus massiliensis* AM1	13.5	[10.7 - 16.8]	29.2	[26.8 - 31.7]	13.8	[11.5 - 16.7]	2.28
**'F1_(ST72).fna'**	*Enterococcus montenegrensis* CoE-012-22	13.4	[10.6 - 16.7]	29.2	[26.8 - 31.7]	13.8	[11.4 - 16.6]	0.08
**'D32_(ST40).fna'**	*Streptococcus gwangjuense* KCOM -1679T	12.7	[10.0 - 16.0]	29.1	[26.7 - 31.6]	13.1	[10.8 - 15.9]	2.78
**'209EA1_(ST624).fna'**	*Streptococcus gwangjuense* KCOM -1679T	12.7	[10.0 - 16.0]	29.1	[26.7 - 31.6]	13.1	[10.8 - 15.9]	2.47
**'F1_(ST72).fna'**	*Streptococcus gwangjuense* KCOM -1679T	12.7	[10.1 - 16.0]	29.1	[26.7 - 31.6]	13.1	[10.8 - 15.9]	2.71
**'CL9943_(ST4).fasta'**	*Enterococcus lactis* CCM 8412	13.3	[10.6 - 16.7]	29.0	[26.6 - 31.5]	13.7	[11.3 - 16.5]	1.14
**'CL9943_(ST4).fasta'**	*Enterococcus lactis* DSM 23655	13.3	[10.6 - 16.7]	29.0	[26.6 - 31.5]	13.7	[11.3 - 16.5]	1.15
**'D32_(ST40).fna'**	*Enterococcus durans* NBRC 100479	13.3	[10.6 - 16.6]	28.9	[26.5 - 31.3]	13.7	[11.3 - 16.5]	0.37
**'E1_(ST40).fna'**	*Vagococcus martis* D7T301	13.1	[10.4 - 16.4]	28.9	[26.5 - 31.4]	13.5	[11.2 - 16.3]	3.59
**'4928STDY7071351_(ST30).fna'**	*Enterococcus xinjiangensis* JCM 30200	13.4	[10.7 - 16.8]	28.9	[26.6 - 31.4]	13.8	[11.4 - 16.6]	1.14
**'E1_(ST40).fna'**	*Enterococcus xinjiangensis* JCM 30200	13.3	[10.6 - 16.7]	28.8	[26.4 - 31.2]	13.7	[11.3 - 16.5]	0.97
**'CL9943_(ST4).fasta'**	*Enterococcus saigonensis* JCM 31193	13.3	[10.6 - 16.7]	28.8	[26.4 - 31.3]	13.7	[11.3 - 16.5]	0.71

Query	Subject	d_0	C.I. d_0	d_4	C.I. d_4	d_6	C.I. d_6	Diff. G+C Percent
**'R48_(ST228).fna'**	*Enterococcus durans* NBRC 100479	13.5	[10.7 - 16.8]	28.8	[26.4 - 31.3]	13.9	[11.5 - 16.7]	0.23
**'KUB3007_(ST729).fna'**	*Enterococcus durans* NBRC 100479	13.3	[10.6 - 16.6]	28.8	[26.4 - 31.3]	13.7	[11.3 - 16.5]	0.62
**'KUB3007_(ST729).fna'**	*Enterococcus massiliensis* AM1	13.4	[10.6 - 16.7]	28.8	[26.5 - 31.3]	13.8	[11.4 - 16.6]	2.48
**'244_EFLS_(ST443).fna'**	*Enterococcus lactis* DSM 23655	13.3	[10.6 - 16.7]	28.7	[26.3 - 31.2]	13.7	[11.3 - 16.5]	0.64
**'4928STDY7071435_(ST40).fna'**	*Enterococcus durans* NBRC 100479	13.4	[10.6 - 16.7]	28.7	[26.3 - 31.2]	13.8	[11.4 - 16.6]	0.07
**'302EA1_(ST122).fna'**	*Enterococcus montenegrensis* CoE-012-22	13.7	[10.9 - 17.0]	28.7	[26.4 - 31.2]	14.0	[11.6 - 16.8]	0.05
**'CL9172_(ST21).fasta'**	*Enterococcus raffinosus* NBRC 100492	13.2	[10.4 - 16.5]	28.7	[26.3 - 31.2]	13.6	[11.2 - 16.4]	1.93
**'CL9172_(ST21).fasta'**	*Enterococcus xinjiangensis* JCM 30200	13.4	[10.6 - 16.7]	28.7	[26.3 - 31.2]	13.8	[11.4 - 16.6]	0.89
**'244_EFLS_(ST443).fna'**	*Enterococcus lactis* CCM 8412	13.3	[10.6 - 16.7]	28.7	[26.3 - 31.2]	13.7	[11.3 - 16.5]	0.63
**'LB00.E.122_(ST21).fasta'**	*Enterococcus xinjiangensis* JCM 30200	13.4	[10.6 - 16.7]	28.7	[26.3 - 31.2]	13.8	[11.4 - 16.6]	0.98
**'F1_(ST72).fna'**	*Enterococcus mediterraneensis* Marseille-P4358	13.5	[10.7 - 16.8]	28.6	[26.2 - 31.1]	13.9	[11.5 - 16.7]	3.39
**'F1_(ST72).fna'**	*Enterococcus saigonensis* JCM 31193	13.3	[10.6 - 16.7]	28.6	[26.2 - 31.1]	13.7	[11.3 - 16.5]	1.22
**'244_EFLS_(ST443).fna'**	*Enterococcus massiliensis* AM1	13.4	[10.6 - 16.7]	28.6	[26.2 - 31.1]	13.8	[11.4 - 16.6]	2.15
**'KUB3007_(ST729).fna'**	*Enterococcus saigonensis* JCM 31193	13.6	[10.8 - 16.9]	28.5	[26.1 - 31.0]	13.9	[11.5 - 16.7]	0.89
**'CL9943_(ST4).fasta'**	*Streptococcus gwangjuense* KCOM -1679T	12.6	[10.0 - 15.9]	28.5	[26.1 - 31.0]	13.0	[10.7 - 15.8]	3.22
**'CL9924_(ST6).fasta'**	*Vagococcus martis* D7T301	13.1	[10.4 - 16.4]	28.5	[26.1 - 31.0]	13.5	[11.1 - 16.3]	3.38
**'CL9314_(ST21).fasta'**	*Enterococcus raffinosus* NBRC 100492	13.2	[10.4 - 16.5]	28.5	[26.2 - 31.0]	13.6	[11.2 - 16.3]	1.94
**'4928STDY7071435_(ST40).fna'**	*Vagococcus martis* D7T301	13.1	[10.4 - 16.4]	28.4	[26.0 - 30.9]	13.5	[11.1 - 16.3]	3.96
**'CL9924_(ST6).fasta'**	*Enterococcus lactis* DSM 23655	13.3	[10.6 - 16.6]	28.4	[26.0 - 30.9]	13.7	[11.3 - 16.5]	0.99
**'F1_(ST72).fna'**	*Enterococcus casseliflavus* NBRC 100478	13.1	[10.4 - 16.4]	28.4	[26.1 - 30.9]	13.5	[11.1 - 16.3]	4.86
**'EN24_(ST228).fna'**	*Vagococcus martis* D7T301	13.1	[10.4 - 16.4]	28.4	[26.1 - 30.9]	13.5	[11.1 - 16.3]	3.78
**'BT00.E.21_(ST23).fasta'**	*Enterococcus xinjiangensis* JCM 30200	13.3	[10.6 - 16.6]	28.4	[26.0 - 30.9]	13.7	[11.3 - 16.5]	1.22
**'BT00.E.21_(ST23).fasta'**	*Enterococcus saigonensis* JCM 31193	13.3	[10.6 - 16.6]	28.3	[25.9 - 30.8]	13.7	[11.3 - 16.5]	0.82
**'Merz96_(ST103).fna'**	*Enterococcus gallinarum* NBRC 100675	13.2	[10.5 - 16.6]	28.3	[25.9 - 30.8]	13.6	[11.3 - 16.4]	2.15
**'F_(ST712).fna'**	*Enterococcus devriesei* DSM 22802	13.2	[10.5 - 16.5]	28.3	[26.0 - 30.8]	13.6	[11.2 - 16.4]	2.63
**'CVM_N52587_(STx).fna'**	*Enterococcus durans* NBRC 100479	13.6	[10.8 - 16.9]	28.3	[26.0 - 30.8]	14.0	[11.5 - 16.8]	0.3

Query	Subject	d_0	C.I. d_0	d_4	C.I. d_4	d_6	C.I. d_6	Diff. G+C Percent
**'BT00.E.21_(ST23).fasta'**	*Enterococcus massiliensis* AM1	13.3	[10.5 - 16.6]	28.3	[26.0 - 30.8]	13.7	[11.3 - 16.5]	2.54
**'4928STDY7071435_(ST40).fna'**	*Enterococcus xinjiangensis* JCM 30200	13.3	[10.6 - 16.6]	28.3	[26.0 - 30.8]	13.7	[11.3 - 16.5]	0.6
**'CL9924_(ST6).fasta'**	*Enterococcus lactis* CCM 8412	13.3	[10.6 - 16.6]	28.3	[25.9 - 30.8]	13.7	[11.3 - 16.5]	0.98
**'14EA1_(ST147).fna'**	*Streptococcus gwangjuense* KCOM -1679T	12.7	[10.0 - 16.0]	28.3	[26.0 - 30.8]	13.1	[10.8 - 15.9]	2.44
**'CL8682_(ST769).fasta'**	*Enterococcus lactis* DSM 23655	13.3	[10.6 - 16.6]	28.2	[25.8 - 30.7]	13.7	[11.3 - 16.5]	0.83
**'CL8682_(ST769).fasta'**	*Enterococcus gallinarum* NBRC 100675	13.2	[10.5 - 16.5]	28.2	[25.8 - 30.7]	13.6	[11.2 - 16.4]	2.45
**'KUB3007_(ST729).fna'**	*Enterococcus porcinus* ATCC 700913	13.7	[10.9 - 17.0]	28.2	[25.8 - 30.7]	14.1	[11.6 - 16.9]	2.15
**'CL6870_(ST103).fasta'**	*Enterococcus durans* NBRC 100479	13.4	[10.6 - 16.7]	28.1	[25.7 - 30.6]	13.7	[11.4 - 16.5]	0.52
**'CL8682_(ST769).fasta'**	*Enterococcus lactis* CCM 8412	13.3	[10.6 - 16.6]	28.1	[25.7 - 30.6]	13.7	[11.3 - 16.5]	0.82
**'CVM_N54595_(ST192).fna'**	*Candidatus* Enterococcus avicola CHK172-16539	13.3	[10.5 - 16.6]	28.1	[25.7 - 30.6]	13.6	[11.3 - 16.4]	0.52
**'CL9314_(ST21).fasta'**	*Enterococcus xinjiangensis* JCM 30200	13.3	[10.6 - 16.7]	28.1	[25.7 - 30.6]	13.7	[11.3 - 16.5]	0.9
**'Praia.S.M2.C4_(ST62).fasta'**	*Enterococcus lactis* DSM 23655	13.3	[10.6 - 16.6]	28.1	[25.7 - 30.6]	13.7	[11.3 - 16.5]	0.77
**'CL6870_(ST103).fasta'**	*Enterococcus gallinarum* NBRC 100675	13.2	[10.5 - 16.5]	28.1	[25.8 - 30.6]	13.6	[11.2 - 16.4]	2.48
**'L14_(ST330).fna'**	*Streptococcus gwangjuense* KCOM -1679T	12.7	[10.0 - 16.0]	28.1	[25.8 - 30.6]	13.1	[10.8 - 15.9]	2.6
**'CL6870_(ST103).fasta'**	*Enterococcus lactis* DSM 23655	13.3	[10.6 - 16.6]	28.0	[25.6 - 30.5]	13.7	[11.3 - 16.5]	0.86
**'CL9797_(ST525).fasta'**	*Enterococcus lactis* DSM 23655	13.3	[10.6 - 16.6]	28.0	[25.6 - 30.5]	13.7	[11.3 - 16.5]	1.02
**'14EA1_(ST147).fna'**	*Enterococcus casseliflavus* NBRC 100478	13.1	[10.4 - 16.4]	28.0	[25.7 - 30.5]	13.5	[11.1 - 16.3]	4.59
**'G81_(ST1468).fna'**	*Enterococcus saigonensis* JCM 31193	13.5	[10.8 - 16.9]	28.0	[25.6 - 30.5]	13.9	[11.5 - 16.7]	1.27
**'CL9797_(ST525).fasta'**	*Enterococcus lactis* CCM 8412	13.3	[10.6 - 16.6]	28.0	[25.6 - 30.5]	13.7	[11.3 - 16.5]	1.01
**'Praia.S.M2.C4_(ST62).fasta'**	*Enterococcus lactis* CCM 8412	13.3	[10.6 - 16.6]	28.0	[25.7 - 30.5]	13.7	[11.3 - 16.5]	0.76
**'CL9924_(ST6).fasta'**	*Enterococcus gallinarum* NBRC 100675	13.3	[10.5 - 16.6]	27.9	[25.5 - 30.4]	13.7	[11.3 - 16.4]	2.61
**'BT00.E.21_(ST23).fasta'**	*Enterococcus gallinarum* NBRC 100675	13.2	[10.5 - 16.5]	27.9	[25.5 - 30.4]	13.6	[11.2 - 16.4]	2.65
**'4928STDY7071351_(ST30).fna'**	*Enterococcus porcinus* ATCC 700913	13.5	[10.8 - 16.9]	27.9	[25.6 - 30.4]	13.9	[11.5 - 16.7]	2.16
**'G81_(ST1468).fna'**	*Streptococcus gwangjuense* KCOM -1679T	12.6	[10.0 - 15.9]	27.9	[25.5 - 30.4]	13.0	[10.7 - 15.8]	2.66
**'CL9772_(ST525).fasta'**	*Enterococcus lactis* CCM 8412	13.3	[10.6 - 16.6]	27.9	[25.5 - 30.4]	13.7	[11.3 - 16.5]	0.93
**'CL6870_(ST103).fasta'**	*Enterococcus lactis* CCM 8412	13.3	[10.6 - 16.6]	27.9	[25.5 - 30.4]	13.7	[11.3 - 16.5]	0.85

Query	Subject	d_0	C.I. d_0	d_4	C.I. d_4	d_6	C.I. d_6	Diff. G+C Percent
**'CL9772_(ST525).fasta'**	*Enterococcus lactis* DSM 23655	13.3	[10.6 - 16.6]	27.9	[25.6 - 30.4]	13.7	[11.3 - 16.5]	0.94
**'F_(ST712).fna'**	*Vagococcus bubulae* SS1994	13.5	[10.7 - 16.8]	27.8	[25.5 - 30.3]	13.8	[11.4 - 16.6]	4.04
**'Merz96_(ST103).fna'**	*Enterococcus lactis* DSM 23655	13.3	[10.6 - 16.6]	27.8	[25.4 - 30.3]	13.7	[11.3 - 16.5]	0.53
**'4928STDY7071351_(ST30).fna'**	*Enterococcus saigonensis* JCM 31193	13.4	[10.6 - 16.7]	27.8	[25.4 - 30.3]	13.7	[11.4 - 16.5]	0.91
**'CL9797_(ST525).fasta'**	*Enterococcus saigonensis* JCM 31193	13.4	[10.6 - 16.7]	27.8	[25.5 - 30.3]	13.8	[11.4 - 16.6]	0.84
**'302EA1_(ST122).fna'**	*Enterococcus gallinarum* NBRC 100675	13.2	[10.5 - 16.5]	27.8	[25.4 - 30.3]	13.6	[11.2 - 16.4]	2.28
**'F_(ST712).fna'**	*Streptococcus gwangjuense* KCOM -1679T	12.7	[10.0 - 15.9]	27.8	[25.4 - 30.3]	13.1	[10.7 - 15.8]	2.61
**'CL9797_(ST525).fasta'**	*Enterococcus durans* NBRC 100479	13.3	[10.6 - 16.7]	27.8	[25.5 - 30.3]	13.7	[11.3 - 16.5]	0.68
**'CVM_N54595_(ST192).fna'**	*Streptococcus gwangjuense* KCOM -1679T	12.6	[10.0 - 15.9]	27.8	[25.4 - 30.3]	13.0	[10.7 - 15.8]	2.81
**'4928STDY7071351_(ST30).fna'**	*Enterococcus thailandicus* DSM 21767	13.4	[10.7 - 16.7]	27.7	[25.3 - 30.2]	13.8	[11.4 - 16.6]	0.58
**'CL11199_(ST778).fasta'**	*Enterococcus saigonensis* JCM 31193	13.4	[10.6 - 16.7]	27.7	[25.3 - 30.2]	13.8	[11.4 - 16.5]	0.91
**'F1_(ST72).fna'**	*Enterococcus xinjiangensis* JCM 30200	13.5	[10.7 - 16.8]	27.7	[25.4 - 30.2]	13.9	[11.5 - 16.7]	0.82
**'F1_(ST72).fna'**	*Enterococcus devriesei* DSM 22802	13.2	[10.5 - 16.6]	27.7	[25.3 - 30.2]	13.6	[11.2 - 16.4]	2.73
**'CVM_N52587_(STx).fna'**	*Enterococcus massiliensis* AM1	13.4	[10.6 - 16.7]	27.7	[25.4 - 30.2]	13.8	[11.4 - 16.5]	2.15
**'CL11199_(ST778).fasta'**	*Enterococcus lactis* DSM 23655	13.3	[10.5 - 16.6]	27.7	[25.4 - 30.2]	13.7	[11.3 - 16.5]	0.94
**'209EA1_(ST624).fna'**	*Vagococcus bubulae* SS1994	13.4	[10.6 - 16.7]	27.7	[25.3 - 30.2]	13.8	[11.4 - 16.6]	4.18
**'CL9772_(ST525).fasta'**	*Enterococcus gallinarum* NBRC 100675	13.2	[10.5 - 16.6]	27.7	[25.3 - 30.2]	13.6	[11.3 - 16.4]	2.56
**'Merz96_(ST103).fna'**	*Enterococcus lactis* CCM 8412	13.3	[10.6 - 16.6]	27.7	[25.3 - 30.2]	13.7	[11.3 - 16.5]	0.52
**'15224_(ST16).fna'**	*Vagococcus martis* D7T301	13.1	[10.4 - 16.4]	27.7	[25.4 - 30.2]	13.5	[11.1 - 16.3]	3.31
**'LB00.E.122_(ST21).fasta'**	*Enterococcus raffinosus* NBRC 100492	13.1	[10.4 - 16.4]	27.7	[25.3 - 30.2]	13.5	[11.2 - 16.3]	2.02
**'F_(ST712).fna'**	*Enterococcus saigonensis* JCM 31193	13.3	[10.6 - 16.7]	27.7	[25.4 - 30.2]	13.7	[11.3 - 16.5]	1.32
**'19910_(ST116).fna'**	*Enterococcus saigonensis* JCM 31193	13.5	[10.7 - 16.8]	27.7	[25.4 - 30.2]	13.8	[11.4 - 16.6]	1.01
**'4928STDY7071351_(ST30).fna'**	*Streptococcus gwangjuense* KCOM -1679T	12.6	[10.0 - 15.9]	27.7	[25.3 - 30.2]	13.0	[10.7 - 15.8]	3.02
**'KUB3007_(ST729).fna'**	*Enterococcus casseliflavus* NBRC 100478	13.1	[10.3 - 16.4]	27.6	[25.3 - 30.1]	13.4	[11.1 - 16.2]	5.18
**'CL11199_(ST778).fasta'**	*Enterococcus lactis* CCM 8412	13.3	[10.6 - 16.6]	27.6	[25.3 - 30.1]	13.7	[11.3 - 16.5]	0.93
**'CL9314_(ST21).fasta'**	*Vagococcus martis* D7T301	13.2	[10.5 - 16.5]	27.6	[25.2 - 30.1]	13.6	[11.2 - 16.4]	3.66

Query	Subject	d_0	C.I. d_0	d_4	C.I. d_4	d_6	C.I. d_6	Diff. G+C Percent
**'CL9924_(ST6).fasta'**	*Enterococcus saigonensis* JCM 31193	13.4	[10.7 - 16.8]	27.6	[25.2 - 30.1]	13.8	[11.4 - 16.6]	0.87
**'LB00.E.122_(ST21).fasta'**	*Enterococcus montenegrensis* CoE-012-22	13.5	[10.7 - 16.8]	27.6	[25.3 - 30.1]	13.8	[11.5 - 16.7]	0.08
**'LB00.E.122_(ST21).fasta'**	*Vagococcus martis* D7T301	13.2	[10.4 - 16.5]	27.6	[25.2 - 30.1]	13.6	[11.2 - 16.3]	3.58
**'CVM_N55265_(ST228).fna'**	*Enterococcus durans* NBRC 100479	13.4	[10.7 - 16.8]	27.6	[25.2 - 30.1]	13.8	[11.4 - 16.6]	0.2
**'CVM N52662_(ST228).fna'**	*Enterococcus durans* NBRC 100479	13.4	[10.7 - 16.7]	27.6	[25.2 - 30.1]	13.8	[11.4 - 16.6]	0.27
**'EF348_(ST16).fna'**	*Vagococcus martis* D7T301	13.2	[10.4 - 16.5]	27.6	[25.3 - 30.1]	13.5	[11.2 - 16.3]	3.77
**'Praia.S.M2.C4_(ST62).fasta'**	*Candidatus* Enterococcus avicola CHK172-16539	13.3	[10.6 - 16.6]	27.6	[25.3 - 30.1]	13.7	[11.3 - 16.5]	0.49
**'R48_(ST228).fna'**	*Enterococcus massiliensis* AM1	13.3	[10.6 - 16.6]	27.5	[25.2 - 30.0]	13.7	[11.3 - 16.5]	2.08
**'CL9172_(ST21).fasta'**	*Vagococcus martis* D7T301	13.2	[10.5 - 16.5]	27.5	[25.1 - 30.0]	13.6	[11.2 - 16.4]	3.67
**'4928STDY7071351_(ST30).fna'**	*Enterococcus gallinarum* NBRC 100675	13.3	[10.6 - 16.6]	27.5	[25.1 - 30.0]	13.7	[11.3 - 16.5]	2.57
**'209EA1_(ST624).fna'**	*Enterococcus durans* NBRC 100479	13.4	[10.7 - 16.8]	27.5	[25.1 - 30.0]	13.8	[11.4 - 16.6]	0.06
**'19910_(ST116).fna'**	*Candidatus* Enterococcus avicola CHK172-16539	13.2	[10.5 - 16.5]	27.5	[25.2 - 30.0]	13.6	[11.2 - 16.4]	0.41
**'244_EFLS_(ST443).fna'**	*Enterococcus montenegrensis* CoE-012-22	13.4	[10.6 - 16.7]	27.5	[25.1 - 29.9]	13.8	[11.4 - 16.6]	0.07
**'Praia.S.M2.C4_(ST62).fasta'**	*Enterococcus italicus* DSM 15952	13.3	[10.6 - 16.6]	27.5	[25.1 - 30.0]	13.7	[11.3 - 16.5]	1.78
**'CVM_N54595_(ST192).fna'**	*Enterococcus durans* NBRC 100479	13.3	[10.5 - 16.6]	27.4	[25.0 - 29.9]	13.7	[11.3 - 16.5]	0.39
**'Praia.S.M2.C4_(ST62).fasta'**	*Enterococcus porcinus* ATCC 700913	13.5	[10.8 - 16.9]	27.4	[25.1 - 29.9]	13.9	[11.5 - 16.7]	2.34
**'EN242_(ST82).fna'**	*Vagococcus martis* D7T301	13.2	[10.4 - 16.5]	27.4	[25.0 - 29.8]	13.6	[11.2 - 16.3]	3.29
**'EN788_(ST82).fna'**	*Vagococcus martis* D7T301	13.2	[10.4 - 16.5]	27.4	[25.0 - 29.9]	13.6	[11.2 - 16.3]	3.17
**'F_(ST712).fna'**	*Enterococcus casseliflavus* NBRC 100478	13.1	[10.4 - 16.4]	27.4	[25.0 - 29.9]	13.5	[11.1 - 16.3]	4.76
**'P9 CL A7_(ST4).fna'**	*Enterococcus casseliflavus* NBRC 100478	13.1	[10.3 - 16.4]	27.4	[25.0 - 29.9]	13.5	[11.1 - 16.2]	5.31
**'EN24_(ST228).fna'**	*Candidatus* Enterococcus avicola CHK172-16539	13.3	[10.5 - 16.6]	27.4	[25.1 - 29.9]	13.7	[11.3 - 16.5]	0.66
**'C116_(ST228).fna'**	*Streptococcus gwangjuense* KCOM -1679T	12.6	[10.0 - 15.9]	27.4	[25.1 - 29.9]	13.0	[10.7 - 15.8]	2.62
**'732_EFLS_(ST16).fna'**	*Vagococcus martis* D7T301	13.2	[10.5 - 16.5]	27.4	[25.1 - 29.9]	13.6	[11.2 - 16.4]	3.6
**'209EA1_(ST624).fna'**	*Enterococcus massiliensis* AM1	13.5	[10.7 - 16.8]	27.3	[25.0 - 29.8]	13.8	[11.4 - 16.6]	1.91
**'CL8682_(ST769).fasta'**	*Enterococcus saigonensis* JCM 31193	13.4	[10.6 - 16.7]	27.3	[24.9 - 29.7]	13.7	[11.4 - 16.5]	1.03
**'CL11199_(ST778).fasta'**	*Enterococcus durans* NBRC 100479	13.3	[10.6 - 16.6]	27.3	[25.0 - 29.8]	13.7	[11.3 - 16.5]	0.6

Query	Subject	d_0	C.I. d_0	d_4	C.I. d_4	d_6	C.I. d_6	Diff. G+C Percent
**'D32_(ST40).fna'**	*Enterococcus montenegrensis* CoE-012-22	13.4	[10.6 - 16.7]	27.3	[25.0 - 29.8]	13.7	[11.4 - 16.5]	0.01
**'F_(ST712).fna'**	*Candidatus* Enterococcus avicola CHK172-16539	13.2	[10.5 - 16.5]	27.3	[24.9 - 29.7]	13.6	[11.2 - 16.4]	0.72
**'D32_(ST40).fna'**	*Enterococcus lactis* CCM 8412	13.3	[10.5 - 16.6]	27.3	[25.0 - 29.8]	13.6	[11.3 - 16.4]	0.7
**'302EA1_(ST122).fna'**	*Enterococcus lactis* CCM 8412	13.3	[10.5 - 16.6]	27.3	[24.9 - 29.8]	13.7	[11.3 - 16.4]	0.66
**'302EA1_(ST122).fna'**	*Enterococcus casseliflavus* NBRC 100478	13.1	[10.4 - 16.4]	27.2	[24.9 - 29.7]	13.5	[11.1 - 16.3]	4.89
**'EN788_(ST82).fna'**	*Candidatus* Enterococcus avicola CHK172-16539	13.2	[10.4 - 16.5]	27.2	[24.9 - 29.7]	13.6	[11.2 - 16.3]	0.05
**'P9 CL A7_(ST4).fna'**	*Enterococcus cecorum* DSM 20682	13.2	[10.5 - 16.6]	27.2	[24.8 - 29.6]	13.6	[11.2 - 16.4]	0.67
**'F1_(ST72).fna'**	*Enterococcus gallinarum* NBRC 100675	13.1	[10.4 - 16.4]	27.2	[24.8 - 29.7]	13.5	[11.1 - 16.3]	2.26
**'D32_(ST40).fna'**	*Enterococcus lactis* DSM 23655	13.2	[10.5 - 16.6]	27.2	[24.8 - 29.7]	13.6	[11.3 - 16.4]	0.71
**'CL9772_(ST525).fasta'**	*Enterococcus durans* NBRC 100479	13.3	[10.6 - 16.6]	27.2	[24.8 - 29.7]	13.7	[11.3 - 16.5]	0.6
**'CVM_N52587_(STx).fna'**	*Candidatus* Enterococcus avicola CHK172-16539	13.3	[10.5 - 16.6]	27.2	[24.8 - 29.7]	13.7	[11.3 - 16.5]	0.62
**'14EA1_(ST147).fna'**	*Enterococcus massiliensis* AM1	13.3	[10.6 - 16.7]	27.2	[24.9 - 29.7]	13.7	[11.3 - 16.5]	1.88
**'EN242_(ST82).fna'**	*Candidatus* Enterococcus avicola CHK172-16539	13.2	[10.5 - 16.5]	27.2	[24.9 - 29.7]	13.6	[11.2 - 16.3]	0.18
**'C138_(ST228).fna'**	*Enterococcus durans* NBRC 100479	13.5	[10.7 - 16.8]	27.2	[24.9 - 29.7]	13.8	[11.4 - 16.6]	0.19
**'EN24_(ST228).fna'**	*Enterococcus casseliflavus* NBRC 100478	13.1	[10.4 - 16.4]	27.2	[24.8 - 29.7]	13.5	[11.1 - 16.3]	4.82
**'CL9314_(ST21).fasta'**	*Enterococcus montenegrensis* CoE-012-22	13.4	[10.7 - 16.8]	27.2	[24.8 - 29.7]	13.8	[11.4 - 16.6]	0.0
**'302EA1_(ST122).fna'**	*Enterococcus mediterraneensis* Marseille-P4358	13.5	[10.7 - 16.8]	27.1	[24.8 - 29.6]	13.9	[11.5 - 16.7]	3.42
**'CL9943_(ST4).fasta'**	*Enterococcus xinjiangensis* JCM 30200	13.3	[10.6 - 16.6]	27.1	[24.7 - 29.6]	13.7	[11.3 - 16.5]	1.34
**'CL9943_(ST4).fasta'**	*Enterococcus massiliensis* AM1	13.3	[10.6 - 16.7]	27.1	[24.8 - 29.6]	13.7	[11.3 - 16.5]	2.66
**'DSM111623_(ST624).fna'**	*Streptococcus gwangjuense* KCOM -1679T	12.6	[10.0 - 15.9]	27.1	[24.8 - 29.6]	13.0	[10.7 - 15.8]	2.74
**'F1_(ST72).fna'**	*Enterococcus thailandicus* DSM 21767	13.5	[10.7 - 16.8]	27.1	[24.8 - 29.6]	13.8	[11.4 - 16.6]	0.89
**'CVM N52662_(ST228).fna'**	*Candidatus* Enterococcus avicola CHK172-16539	13.3	[10.5 - 16.6]	27.1	[24.7 - 29.6]	13.6	[11.3 - 16.4]	0.65
**'302EA1_(ST122).fna'**	*Enterococcus lactis* DSM 23655	13.3	[10.5 - 16.6]	27.1	[24.7 - 29.6]	13.6	[11.3 - 16.4]	0.67
**'302EA1_(ST122).fna'**	*Enterococcus massiliensis* AM1	13.4	[10.6 - 16.7]	27.1	[24.8 - 29.6]	13.8	[11.4 - 16.6]	2.18
**'20_SD_W_06_(ST368).fna'**	*Candidatus* Enterococcus avicola CHK172-16539	13.2	[10.5 - 16.5]	27.1	[24.8 - 29.6]	13.6	[11.2 - 16.4]	0.48

Query	Subject	d_0	C.I. d_0	d_4	C.I. d_4	d_6	C.I. d_6	Diff. G+C Percent
**'G81_(ST1468).fna'**	*Candidatus* Enterococcus avicola CHK172-16539	13.4	[10.6 - 16.7]	27.0	[24.6 - 29.5]	13.8	[11.4 - 16.6]	0.67
**'Praia.S.M2.C4_(ST62).fasta'**	*Enterococcus montenegrensis* CoE-012-22	13.4	[10.7 - 16.8]	27.0	[24.7 - 29.5]	13.8	[11.4 - 16.6]	0.05
**'CVM_N55265_(ST228).fna'**	*Candidatus* Enterococcus avicola CHK172-16539	13.3	[10.5 - 16.6]	27.0	[24.7 - 29.5]	13.7	[11.3 - 16.4]	0.71
**'CL8682_(ST769).fasta'**	*Vagococcus martis* D7T301	13.1	[10.4 - 16.4]	27.0	[24.7 - 29.5]	13.5	[11.1 - 16.3]	3.55
**'P9 CL A7_(ST4).fna'**	*Enterococcus raffinosus* NBRC 100492	13.1	[10.4 - 16.4]	27.0	[24.7 - 29.5]	13.5	[11.1 - 16.3]	2.32
**'CL11199_(ST778).fasta'**	*Vagococcus martis* D7T301	13.1	[10.4 - 16.4]	27.0	[24.6 - 29.4]	13.5	[11.1 - 16.3]	3.43
**'BIOML_A5_(ST875).fna'**	*Candidatus* Enterococcus avicola CHK172-16539	13.2	[10.5 - 16.5]	27.0	[24.7 - 29.5]	13.6	[11.2 - 16.4]	0.46
**'F_(ST712).fna'**	*Enterococcus mediterraneensis* Marseille-P4358	13.4	[10.6 - 16.7]	27.0	[24.6 - 29.5]	13.7	[11.4 - 16.5]	3.29
**'CL9772_(ST525).fasta'**	*Candidatus* Enterococcus avicola CHK172-16539	13.2	[10.5 - 16.5]	27.0	[24.6 - 29.5]	13.6	[11.2 - 16.4]	0.32
**'CL9172_(ST21).fasta'**	*Enterococcus montenegrensis* CoE-012-22	13.4	[10.7 - 16.7]	26.9	[24.6 - 29.4]	13.8	[11.4 - 16.6]	0.01
**'F_(ST712).fna'**	*Enterococcus xinjiangensis* JCM 30200	13.5	[10.7 - 16.8]	26.9	[24.6 - 29.4]	13.8	[11.5 - 16.6]	0.72
**'R48_(ST228).fna'**	*Enterococcus saigonensis* JCM 31193	13.4	[10.7 - 16.7]	26.9	[24.6 - 29.4]	13.8	[11.4 - 16.6]	1.29
**'R48_(ST228).fna'**	*Candidatus* Enterococcus avicola CHK172-16539	13.3	[10.5 - 16.6]	26.9	[24.5 - 29.4]	13.6	[11.3 - 16.4]	0.69
**'CL9797_(ST525).fasta'**	*Vagococcus martis* D7T301	13.1	[10.4 - 16.4]	26.9	[24.6 - 29.4]	13.5	[11.1 - 16.3]	3.35
**'4928STDY7071351_(ST30).fna'**	*Enterococcus montenegrensis* CoE-012-22	13.5	[10.7 - 16.8]	26.9	[24.6 - 29.4]	13.9	[11.5 - 16.7]	0.24
**'CL9924_(ST6).fasta'**	*Candidatus* Enterococcus avicola CHK172-16539	13.2	[10.5 - 16.5]	26.9	[24.6 - 29.4]	13.6	[11.2 - 16.4]	0.27
**'C138_(ST228).fna'**	*Candidatus* Enterococcus avicola CHK172-16539	13.3	[10.5 - 16.6]	26.9	[24.5 - 29.4]	13.7	[11.3 - 16.5]	0.72
**'CL8682_(ST769).fasta'**	*Enterococcus durans* NBRC 100479	13.3	[10.6 - 16.6]	26.9	[24.5 - 29.4]	13.7	[11.3 - 16.5]	0.49
**'D32_(ST40).fna'**	*Enterococcus massiliensis* AM1	13.3	[10.6 - 16.6]	26.9	[24.5 - 29.4]	13.7	[11.3 - 16.5]	2.22
**'EN242_(ST82).fna'**	*Enterococcus raffinosus* NBRC 100492	13.3	[10.5 - 16.6]	26.9	[24.5 - 29.4]	13.7	[11.3 - 16.5]	2.31
**'CVM_N52587_(STx).fna'**	*Enterococcus saigonensis* JCM 31193	13.4	[10.7 - 16.8]	26.9	[24.6 - 29.4]	13.8	[11.4 - 16.6]	1.22
**'C138_(ST228).fna'**	*Streptococcus gwangjuense* KCOM -1679T	12.6	[10.0 - 15.9]	26.9	[24.6 - 29.4]	13.0	[10.7 - 15.8]	2.61
**'244_EFLS_(ST443).fna'**	*Streptococcus gwangjuense* KCOM -1679T	12.6	[10.0 - 15.9]	26.9	[24.6 - 29.4]	13.0	[10.7 - 15.8]	2.71
**'19910_(ST116).fna'**	*Vagococcus martis* D7T301	13.1	[10.4 - 16.4]	26.9	[24.6 - 29.4]	13.5	[11.1 - 16.3]	3.53
**'4928STDY7071351_(ST30).fna'**	*Candidatus* Enterococcus avicola CHK172-16539	13.2	[10.5 - 16.5]	26.9	[24.5 - 29.4]	13.6	[11.2 - 16.4]	0.31

Query	Subject	d_0	C.I. d_0	d_4	C.I. d_4	d_6	C.I. d_6	Diff. G+C Percent
**'Praia.S.M2.C4_(ST62).fasta'**	*Enterococcus songbeiensis* NCIMB 15179	13.3	[10.6 - 16.7]	26.8	[24.4 - 29.3]	13.7	[11.3 - 16.5]	2.37
**'KUB3007_(ST729).fna'**	*Enterococcus lemanii* DSM 105069	13.7	[10.9 - 17.0]	26.8	[24.4 - 29.3]	14.0	[11.6 - 16.9]	0.05
**'209EA1_(ST624).fna'**	*Enterococcus casseliflavus* NBRC 100478	13.1	[10.4 - 16.4]	26.8	[24.5 - 29.3]	13.5	[11.1 - 16.3]	4.62
**'F_(ST712).fna'**	*Enterococcus montenegrensis* CoE-012-22	13.3	[10.6 - 16.7]	26.8	[24.5 - 29.3]	13.7	[11.3 - 16.5]	0.18
**'G81_(ST1468).fna'**	*Enterococcus massiliensis* AM1	13.5	[10.8 - 16.8]	26.8	[24.4 - 29.3]	13.9	[11.5 - 16.7]	2.1
**'302EA1_(ST122).fna'**	*Enterococcus saigonensis* JCM 31193	13.4	[10.7 - 16.7]	26.8	[24.5 - 29.3]	13.8	[11.4 - 16.6]	1.19
**'14EA1_(ST147).fna'**	*Enterococcus saigonensis* JCM 31193	13.4	[10.6 - 16.7]	26.8	[24.5 - 29.3]	13.7	[11.4 - 16.5]	1.49
**'14EA1_(ST147).fna'**	*Candidatus* Enterococcus avicola CHK172-16539	13.3	[10.5 - 16.6]	26.8	[24.4 - 29.3]	13.7	[11.3 - 16.5]	0.89
**'EF349_(ST631).fna'**	*Candidatus* Enterococcus avicola CHK172-16539	13.3	[10.5 - 16.6]	26.8	[24.5 - 29.3]	13.6	[11.3 - 16.4]	1.76
**'14EA1_(ST147).fna'**	*Enterococcus lactis* DSM 23655	13.3	[10.6 - 16.6]	26.8	[24.4 - 29.3]	13.7	[11.3 - 16.5]	0.37
**'C116_(ST228).fna'**	*Candidatus* Enterococcus avicola CHK172-16539	13.3	[10.5 - 16.6]	26.8	[24.4 - 29.2]	13.7	[11.3 - 16.4]	0.71
**'EN788_(ST82).fna'**	*Enterococcus raffinosus* NBRC 100492	13.3	[10.5 - 16.6]	26.8	[24.5 - 29.3]	13.7	[11.3 - 16.4]	2.43
**'CVM N53420_(ST228).fna'**	*Candidatus* Enterococcus avicola CHK172-16539	13.2	[10.5 - 16.6]	26.8	[24.4 - 29.3]	13.6	[11.2 - 16.4]	0.57
**'CL9797_(ST525).fasta'**	*Candidatus* Enterococcus avicola CHK172-16539	13.2	[10.5 - 16.5]	26.8	[24.4 - 29.3]	13.6	[11.2 - 16.4]	0.24
**'CL9172_(ST21).fasta'**	*Enterococcus massiliensis* AM1	13.3	[10.6 - 16.7]	26.8	[24.4 - 29.3]	13.7	[11.3 - 16.5]	2.21
**'Merz96_(ST103).fna'**	*Enterococcus durans* NBRC 100479	13.3	[10.5 - 16.6]	26.8	[24.4 - 29.2]	13.7	[11.3 - 16.4]	0.19
**'209EA1_(ST624).fna'**	*Enterococcus saigonensis* JCM 31193	13.5	[10.7 - 16.8]	26.7	[24.3 - 29.2]	13.9	[11.5 - 16.7]	1.46
**'BIOML_A5_(ST875).fna'**	*Streptococcus gwangjuense* KCOM -1679T	12.6	[10.0 - 15.9]	26.7	[24.3 - 29.2]	13.0	[10.7 - 15.8]	2.87
**'KUB3007_(ST729).fna'**	*Enterococcus thailandicus* DSM 21767	13.4	[10.6 - 16.7]	26.7	[24.3 - 29.1]	13.7	[11.4 - 16.5]	0.57
**'4928STDY7071435_(ST40).fna'**	*Enterococcus italicus* DSM 15952	13.2	[10.5 - 16.5]	26.7	[24.4 - 29.2]	13.6	[11.2 - 16.4]	1.43
**'BT00.E.21_(ST23).fasta'**	*Enterococcus lemanii* DSM 105069	13.5	[10.7 - 16.8]	26.7	[24.4 - 29.2]	13.8	[11.4 - 16.6]	0.12
**'CL9824_(ST631).fasta'**	*Candidatus* Enterococcus avicola CHK172-16539	13.3	[10.5 - 16.6]	26.7	[24.3 - 29.2]	13.7	[11.3 - 16.5]	0.83
**'20_SD_W_06_(ST368).fna'**	*Streptococcus gwangjuense* KCOM -1679T	12.6	[10.0 - 15.9]	26.7	[24.3 - 29.2]	13.0	[10.7 - 15.8]	2.85
**'KUB3007_(ST729).fna'**	*Enterococcus mediterraneensis* Marseille-P4358	13.4	[10.6 - 16.7]	26.7	[24.3 - 29.2]	13.7	[11.4 - 16.5]	3.72
**'19910_(ST116).fna'**	*Enterococcus durans* NBRC 100479	13.3	[10.5 - 16.6]	26.7	[24.4 - 29.2]	13.6	[11.3 - 16.4]	0.51

Query	Subject	d_0	C.I. d_0	d_4	C.I. d_4	d_6	C.I. d_6	Diff. G+C Percent
**'CL9772_(ST525).fasta'**	*Vagococcus martis* D7T301	13.1	[10.4 - 16.4]	26.6	[24.3 - 29.1]	13.5	[11.1 - 16.3]	3.43
**'CVM_N60027F_(ST862).fna'**	*Vagococcus bubulae* SS1994	13.3	[10.6 - 16.6]	26.6	[24.2 - 29.1]	13.7	[11.3 - 16.5]	4.0
**'CL11199_(ST778).fasta'**	*Candidatus* Enterococcus avicola CHK172-16539	13.2	[10.5 - 16.5]	26.6	[24.2 - 29.1]	13.6	[11.2 - 16.4]	0.32
**'EnGen0400_(ST86).fna'**	*Candidatus* Enterococcus avicola CHK172-16539	13.3	[10.5 - 16.6]	26.6	[24.2 - 29.0]	13.6	[11.3 - 16.4]	0.81
**'KUB3007_(ST729).fna'**	*Candidatus* Enterococcus avicola CHK172-16539	13.2	[10.5 - 16.5]	26.6	[24.3 - 29.1]	13.6	[11.2 - 16.4]	0.3
**'LB00.E.122_(ST21).fasta'**	*Enterococcus massiliensis* AM1	13.3	[10.6 - 16.7]	26.6	[24.2 - 29.1]	13.7	[11.3 - 16.5]	2.31
**'CL9772_(ST525).fasta'**	*Enterococcus saigonensis* JCM 31193	13.3	[10.6 - 16.7]	26.6	[24.2 - 29.1]	13.7	[11.3 - 16.5]	0.92
**'CL9314_(ST21).fasta'**	*Candidatus* Enterococcus avicola CHK172-16539	13.2	[10.5 - 16.6]	26.6	[24.3 - 29.1]	13.6	[11.2 - 16.4]	0.55
**'CVM_N52587_(STx).fna'**	*Streptococcus gwangjuense* KCOM -1679T	12.6	[10.0 - 15.9]	26.6	[24.3 - 29.1]	13.0	[10.7 - 15.8]	2.71
**'LB00.E.122_(ST21).fasta'**	*Candidatus* Enterococcus avicola CHK172-16539	13.2	[10.5 - 16.5]	26.6	[24.3 - 29.1]	13.6	[11.2 - 16.4]	0.47
**'CL9824_(ST631).fasta'**	*Streptococcus gwangjuense* KCOM -1679T	12.6	[10.0 - 15.9]	26.6	[24.3 - 29.1]	13.0	[10.7 - 15.8]	2.5
**'CVM_N55265_(ST228).fna'**	*Streptococcus gwangjuense* KCOM -1679T	12.6	[10.0 - 15.9]	26.6	[24.3 - 29.1]	13.0	[10.7 - 15.8]	2.62
**'302EA1_(ST122).fna'**	*Candidatus* Enterococcus avicola CHK172-16539	13.2	[10.5 - 16.5]	26.6	[24.2 - 29.1]	13.6	[11.2 - 16.4]	0.59
**'BT00.E.21_(ST23).fasta'**	*Enterococcus montenegrensis* CoE-012-22	13.3	[10.6 - 16.7]	26.6	[24.2 - 29.1]	13.7	[11.3 - 16.5]	0.32
**'CL9172_(ST21).fasta'**	*Candidatus* Enterococcus avicola CHK172-16539	13.2	[10.5 - 16.6]	26.6	[24.3 - 29.1]	13.6	[11.2 - 16.4]	0.56
**'14EA1_(ST147).fna'**	*Enterococcus lactis* CCM 8412	13.3	[10.6 - 16.6]	26.6	[24.3 - 29.1]	13.7	[11.3 - 16.5]	0.36
**'CL8682_(ST769).fasta'**	*Enterococcus cecorum* DSM 20682	13.1	[10.4 - 16.4]	26.5	[24.2 - 29.0]	13.5	[11.1 - 16.3]	0.93
**'CL6870_(ST103).fasta'**	*Enterococcus saigonensis* JCM 31193	13.3	[10.6 - 16.7]	26.5	[24.2 - 29.0]	13.7	[11.3 - 16.5]	1.0
**'BT00.E.21_(ST23).fasta'**	*Candidatus* Enterococcus avicola CHK172-16539	13.2	[10.5 - 16.5]	26.5	[24.2 - 29.0]	13.6	[11.2 - 16.4]	0.23
**'CVM_N60027F_(ST862).fna'**	*Enterococcus massiliensis* AM1	13.3	[10.6 - 16.6]	26.5	[24.1 - 29.0]	13.7	[11.3 - 16.5]	2.09
**'4928STDY7071435_(ST40).fna'**	*Enterococcus raffinosus* NBRC 100492	13.1	[10.4 - 16.4]	26.5	[24.1 - 28.9]	13.5	[11.1 - 16.3]	1.64
**'CL11199_(ST778).fasta'**	*Enterococcus cecorum* DSM 20682	13.1	[10.4 - 16.4]	26.5	[24.2 - 29.0]	13.5	[11.1 - 16.2]	0.82
**'4928STDY7071351_(ST30).fna'**	*Enterococcus mediterraneensis* Marseille-P4358	13.5	[10.7 - 16.8]	26.5	[24.2 - 29.0]	13.8	[11.4 - 16.6]	3.71
**'E1_(ST40).fna'**	*Enterococcus montenegrensis* CoE-012-22	13.3	[10.6 - 16.7]	26.5	[24.2 - 29.0]	13.7	[11.3 - 16.5]	0.07
**'CVM_N60027F_(ST862).fna'**	*Enterococcus saigonensis* JCM 31193	13.4	[10.7 - 16.8]	26.5	[24.2 - 29.0]	13.8	[11.4 - 16.6]	1.28

Query	Subject	d_0	C.I. d_0	d_4	C.I. d_4	d_6	C.I. d_6	Diff. G+C Percent
**'T11_(ST65).fna'**	*Candidatus* Enterococcus avicola CHK172-16539	13.3	[10.5 - 16.6]	26.5	[24.1 - 29.0]	13.6	[11.3 - 16.4]	0.78
**'LB00.E.122_(ST21).fasta'**	*Enterococcus gallinarum* NBRC 100675	13.2	[10.4 - 16.5]	26.5	[24.1 - 29.0]	13.5	[11.2 - 16.3]	2.41
**'CL6870_(ST103).fasta'**	*Enterococcus cecorum* DSM 20682	13.1	[10.4 - 16.4]	26.5	[24.2 - 29.0]	13.5	[11.1 - 16.3]	0.9
**'BT00.E.21_(ST23).fasta'**	*Enterococcus songbeiensis* NCIMB 15179	13.2	[10.5 - 16.6]	26.5	[24.1 - 29.0]	13.6	[11.2 - 16.4]	2.63
**'Merz96_(ST103).fna'**	*Enterococcus cecorum* DSM 20682	13.1	[10.4 - 16.4]	26.5	[24.1 - 28.9]	13.5	[11.1 - 16.3]	1.23
**'CL8682_(ST769).fasta'**	*Enterococcus xinjiangensis* JCM 30200	13.3	[10.5 - 16.6]	26.4	[24.1 - 28.9]	13.6	[11.3 - 16.4]	1.01
**'F_(ST712).fna'**	*Enterococcus thailandicus* DSM 21767	13.5	[10.7 - 16.8]	26.4	[24.1 - 28.9]	13.8	[11.4 - 16.6]	0.99
**'CL9924_(ST6).fasta'**	*Enterococcus mediterraneensis* Marseille-P4358	13.4	[10.7 - 16.7]	26.4	[24.0 - 28.8]	13.8	[11.4 - 16.6]	3.75
**'4928STDY7071435_(ST40).fna'**	*Enterococcus massiliensis* AM1	13.3	[10.5 - 16.6]	26.4	[24.0 - 28.9]	13.7	[11.3 - 16.5]	1.93
**'E1_(ST40).fna'**	*Enterococcus raffinosus* NBRC 100492	13.1	[10.4 - 16.4]	26.4	[24.0 - 28.9]	13.5	[11.1 - 16.3]	2.01
**'L14_(ST330).fna'**	*Candidatus* Enterococcus avicola CHK172-16539	13.3	[10.5 - 16.6]	26.4	[24.1 - 28.9]	13.7	[11.3 - 16.5]	0.73
**'209EA1_(ST624).fna'**	*Enterococcus mediterraneensis* Marseille-P4358	13.3	[10.6 - 16.7]	26.4	[24.0 - 28.9]	13.7	[11.3 - 16.5]	3.16
**'4928STDY7071351_(ST30).fna'**	*Enterococcus italicus* DSM 15952	13.3	[10.6 - 16.6]	26.4	[24.1 - 28.9]	13.7	[11.3 - 16.5]	1.97
**'15224_(ST16).fna'**	*Enterococcus saigonensis* JCM 31193	13.4	[10.6 - 16.7]	26.4	[24.0 - 28.9]	13.7	[11.3 - 16.5]	0.79
**'CL9797_(ST525).fasta'**	*Enterococcus xinjiangensis* JCM 30200	13.3	[10.5 - 16.6]	26.4	[24.0 - 28.9]	13.6	[11.3 - 16.4]	1.21
**'CL9772_(ST525).fasta'**	*Enterococcus xinjiangensis* JCM 30200	13.3	[10.5 - 16.6]	26.4	[24.0 - 28.9]	13.6	[11.3 - 16.4]	1.13
**'F1_(ST72).fna'**	*Candidatus* Enterococcus avicola CHK172-16539	13.2	[10.5 - 16.6]	26.3	[24.0 - 28.8]	13.6	[11.2 - 16.4]	0.62
**'4928STDY7071435_(ST40).fna'**	*Enterococcus montenegrensis* CoE-012-22	13.3	[10.6 - 16.6]	26.3	[24.0 - 28.8]	13.7	[11.3 - 16.5]	0.3
**'15224_(ST16).fna'**	*Enterococcus raffinosus* NBRC 100492	13.1	[10.4 - 16.4]	26.3	[23.9 - 28.8]	13.5	[11.2 - 16.3]	2.29
**'G81_(ST1468).fna'**	*Enterococcus porcinus* ATCC 700913	13.7	[10.9 - 17.0]	26.3	[24.0 - 28.8]	14.0	[11.6 - 16.9]	2.53
**'15224_(ST16).fna'**	*Enterococcus cecorum* DSM 20682	13.1	[10.4 - 16.4]	26.3	[23.9 - 28.8]	13.5	[11.1 - 16.3]	0.69
**'302EA1_(ST122).fna'**	*Enterococcus raffinosus* NBRC 100492	13.2	[10.5 - 16.5]	26.3	[24.0 - 28.8]	13.6	[11.2 - 16.3]	1.89
**'CL9314_(ST21).fasta'**	*Enterococcus gallinarum* NBRC 100675	13.1	[10.4 - 16.4]	26.3	[24.0 - 28.8]	13.5	[11.1 - 16.3]	2.33
**'CL9924_(ST6).fasta'**	*Enterococcus xinjiangensis* JCM 30200	13.3	[10.5 - 16.6]	26.3	[24.0 - 28.8]	13.7	[11.3 - 16.5]	1.18
**'244_EFLS_(ST443).fna'**	*Enterococcus saigonensis* JCM 31193	13.3	[10.6 - 16.6]	26.3	[24.0 - 28.8]	13.7	[11.3 - 16.5]	1.22
**'G81_(ST1468).fna'**	*Vagococcus bubulae* SS1994	13.3	[10.6 - 16.6]	26.3	[23.9 - 28.8]	13.7	[11.3 - 16.5]	4.0

Query	Subject	d_0	C.I. d_0	d_4	C.I. d_4	d_6	C.I. d_6	Diff. G+C Percent
**'CVM_N54595_(ST192).fna'**	*Enterococcus raffinosus* NBRC 100492	13.1	[10.3 - 16.4]	26.3	[24.0 - 28.8]	13.4	[11.1 - 16.2]	1.96
**'15224_(ST16).fna'**	*Candidatus* Enterococcus avicola CHK172-16539	13.2	[10.5 - 16.5]	26.3	[24.0 - 28.8]	13.6	[11.2 - 16.4]	0.19
**'CVM_N54595_(ST192).fna'**	*Enterococcus montenegrensis* CoE-012-22	13.4	[10.7 - 16.7]	26.3	[23.9 - 28.7]	13.8	[11.4 - 16.6]	0.02
**'P9 CL A7_(ST4).fna'**	*Candidatus* Enterococcus avicola CHK172-16539	13.3	[10.5 - 16.6]	26.2	[23.9 - 28.7]	13.6	[11.3 - 16.4]	0.17
**'244_EFLS_(ST443).fna'**	*Candidatus* Enterococcus avicola CHK172-16539	13.3	[10.5 - 16.6]	26.2	[23.8 - 28.7]	13.6	[11.3 - 16.4]	0.62
**'CL6870_(ST103).fasta'**	*Enterococcus xinjiangensis* JCM 30200	13.3	[10.5 - 16.6]	26.2	[23.8 - 28.6]	13.6	[11.3 - 16.4]	1.05
**'E1_(ST40).fna'**	*Candidatus* Enterococcus avicola CHK172-16539	13.2	[10.5 - 16.6]	26.2	[23.8 - 28.7]	13.6	[11.2 - 16.4]	0.48
**'CL8682_(ST769).fasta'**	*Candidatus* Enterococcus avicola CHK172-16539	13.2	[10.5 - 16.5]	26.2	[23.9 - 28.7]	13.6	[11.2 - 16.4]	0.43
**'4928STDY7071435_(ST40).fna'**	*Candidatus* Enterococcus avicola CHK172-16539	13.2	[10.5 - 16.5]	26.2	[23.9 - 28.7]	13.6	[11.2 - 16.4]	0.84
**'244_EFLS_(ST443).fna'**	*Enterococcus devriesei* DSM 22802	13.2	[10.5 - 16.5]	26.2	[23.8 - 28.7]	13.6	[11.2 - 16.3]	2.74
**'15224_(ST16).fna'**	*Enterococcus casseliflavus* NBRC 100478	13.0	[10.3 - 16.3]	26.2	[23.8 - 28.6]	13.4	[11.0 - 16.2]	5.29
**'Merz96_(ST103).fna'**	*Candidatus* Enterococcus avicola CHK172-16539	13.2	[10.5 - 16.6]	26.2	[23.8 - 28.7]	13.6	[11.2 - 16.4]	0.73
**'CL6870_(ST103).fasta'**	*Candidatus* Enterococcus avicola CHK172-16539	13.2	[10.5 - 16.6]	26.2	[23.8 - 28.7]	13.6	[11.2 - 16.4]	0.4
**'CVM_N60027F_(ST862).fna'**	*Candidatus* Enterococcus avicola CHK172-16539	13.3	[10.6 - 16.6]	26.2	[23.9 - 28.7]	13.7	[11.3 - 16.5]	0.68
**'CL9943_(ST4).fasta'**	*Candidatus* Enterococcus avicola CHK172-16539	13.2	[10.5 - 16.5]	26.2	[23.8 - 28.7]	13.6	[11.2 - 16.4]	0.11
**'302EA1_(ST122).fna'**	*Enterococcus thailandicus* DSM 21767	13.5	[10.8 - 16.9]	26.2	[23.8 - 28.7]	13.9	[11.5 - 16.7]	0.86
**'G81_(ST1468).fna'**	*Enterococcus mediterraneensis* Marseille-P4358	13.4	[10.6 - 16.7]	26.1	[23.8 - 28.6]	13.7	[11.3 - 16.5]	3.34
**'15224_(ST16).fna'**	*Enterococcus durans* NBRC 100479	13.2	[10.5 - 16.5]	26.1	[23.8 - 28.6]	13.6	[11.2 - 16.4]	0.72
**'CL6870_(ST103).fasta'**	*Vagococcus martis* D7T301	13.2	[10.4 - 16.5]	26.1	[23.8 - 28.6]	13.5	[11.2 - 16.3]	3.51
**'CL11199_(ST778).fasta'**	*Enterococcus xinjiangensis* JCM 30200	13.2	[10.5 - 16.6]	26.1	[23.8 - 28.6]	13.6	[11.2 - 16.4]	1.13
**'EF348_(ST16).fna'**	*Candidatus* Enterococcus avicola CHK172-16539	13.3	[10.5 - 16.6]	26.1	[23.7 - 28.6]	13.6	[11.3 - 16.4]	0.66
**'EnGen0400_(ST86).fna'**	*Enterococcus casseliflavus* NBRC 100478	13.0	[10.3 - 16.3]	26.1	[23.8 - 28.6]	13.4	[11.1 - 16.2]	4.67
**'CL9772_(ST525).fasta'**	*Enterococcus cecorum* DSM 20682	13.1	[10.4 - 16.4]	26.1	[23.7 - 28.6]	13.5	[11.1 - 16.3]	0.82
**'4928STDY7071435_(ST40).fna'**	*Enterococcus gallinarum* NBRC 100675	13.2	[10.4 - 16.5]	26.1	[23.8 - 28.6]	13.5	[11.2 - 16.3]	2.03
**'CVM_N60027F_(ST862).fna'**	*Vagococcus martis* D7T301	13.2	[10.5 - 16.5]	26.1	[23.7 - 28.5]	13.6	[11.2 - 16.3]	3.79
**'14EA1_(ST147).fna'**	*Enterococcus montenegrensis* CoE-012-22	13.3	[10.6 - 16.7]	26.1	[23.7 - 28.5]	13.7	[11.3 - 16.5]	0.35

Query	Subject	d_0	C.I. d_0	d_4	C.I. d_4	d_6	C.I. d_6	Diff. G+C Percent
**'KUB3007_(ST729).fna'**	*Vagococcus bubulae* SS1994	13.3	[10.6 - 16.7]	26.1	[23.8 - 28.6]	13.7	[11.3 - 16.5]	3.62
**'D32_(ST40).fna'**	*Candidatus* Enterococcus avicola CHK172-16539	13.3	[10.5 - 16.6]	26.1	[23.8 - 28.6]	13.6	[11.2 - 16.4]	0.55
**'KUB3007_(ST729).fna'**	*Enterococcus gallinarum* NBRC 100675	13.0	[10.3 - 16.3]	26.1	[23.7 - 28.6]	13.4	[11.0 - 16.2]	2.58
**'Merz96_(ST103).fna'**	*Enterococcus xinjiangensis* JCM 30200	13.3	[10.5 - 16.6]	26.1	[23.8 - 28.6]	13.6	[11.3 - 16.4]	0.72
**'F_(ST712).fna'**	*Enterococcus porcinus* ATCC 700913	13.6	[10.8 - 17.0]	26.1	[23.7 - 28.6]	14.0	[11.6 - 16.8]	2.57
**'EN24_(ST228).fna'**	*Enterococcus durans* NBRC 100479	13.4	[10.6 - 16.7]	26.1	[23.7 - 28.6]	13.8	[11.4 - 16.6]	0.26
**'244_EFLS_(ST443).fna'**	*Enterococcus xinjiangensis* JCM 30200	13.4	[10.6 - 16.7]	26.1	[23.8 - 28.6]	13.8	[11.4 - 16.5]	0.83
**'Merz96_(ST103).fna'**	*Vagococcus martis* D7T301	13.2	[10.4 - 16.5]	26.0	[23.7 - 28.5]	13.5	[11.2 - 16.3]	3.84
**'CVM_N60027F_(ST862).fna'**	*Enterococcus durans* NBRC 100479	13.3	[10.6 - 16.6]	26.0	[23.6 - 28.4]	13.7	[11.3 - 16.5]	0.24
**'BT00.E.21_(ST23).fasta'**	*Enterococcus xiangfangensis* DSM 105127	13.3	[10.5 - 16.6]	26.0	[23.6 - 28.5]	13.6	[11.3 - 16.4]	1.92
**'R48_(ST228).fna'**	*Vagococcus martis* D7T301	13.0	[10.3 - 16.3]	26.0	[23.7 - 28.5]	13.4	[11.0 - 16.2]	3.8
**'CL9172_(ST21).fasta'**	*Enterococcus gallinarum* NBRC 100675	13.2	[10.4 - 16.5]	26.0	[23.7 - 28.5]	13.5	[11.2 - 16.3]	2.32
**'DSM111623_(ST624).fna'**	*Candidatus* Enterococcus avicola CHK172-16539	13.3	[10.6 - 16.6]	26.0	[23.7 - 28.5]	13.7	[11.3 - 16.5]	0.6
**'732_EFLS_(ST16).fna'**	*Enterococcus gallinarum* NBRC 100675	13.2	[10.4 - 16.5]	26.0	[23.6 - 28.4]	13.6	[11.2 - 16.3]	2.39
**'14EA1_(ST147).fna'**	*Enterococcus mediterraneensis* Marseille-P4358	13.4	[10.7 - 16.7]	26.0	[23.7 - 28.5]	13.8	[11.4 - 16.6]	3.12
**'209EA1_(ST624).fna'**	*Vagococcus martis* D7T301	13.1	[10.4 - 16.4]	26.0	[23.7 - 28.5]	13.5	[11.1 - 16.3]	3.97
**'C138_(ST228).fna'**	*Enterococcus casseliflavus* NBRC 100478	13.1	[10.4 - 16.4]	26.0	[23.6 - 28.5]	13.5	[11.1 - 16.2]	4.76
**'BT00.E.21_(ST23).fasta'**	*Enterococcus xiangfangensis* NCIMB 14834	13.3	[10.5 - 16.6]	26.0	[23.7 - 28.5]	13.6	[11.3 - 16.4]	1.91
**'CL9797_(ST525).fasta'**	*Enterococcus mediterraneensis* Marseille-P4358	13.4	[10.6 - 16.7]	25.9	[23.6 - 28.4]	13.8	[11.4 - 16.5]	3.78
**'KUB3007_(ST729).fna'**	*Enterococcus italicus* DSM 15952	13.3	[10.6 - 16.6]	25.9	[23.6 - 28.4]	13.7	[11.3 - 16.5]	1.98
**'CL9943_(ST4).fasta'**	*Enterococcus thailandicus* DSM 21767	13.3	[10.6 - 16.6]	25.9	[23.5 - 28.3]	13.7	[11.3 - 16.5]	0.38
**'CL9797_(ST525).fasta'**	*Enterococcus cecorum* DSM 20682	13.1	[10.4 - 16.4]	25.9	[23.6 - 28.4]	13.5	[11.1 - 16.3]	0.74
**'CVM_N54595_(ST192).fna'**	*Enterococcus massiliensis* AM1	13.2	[10.5 - 16.6]	25.9	[23.6 - 28.4]	13.6	[11.2 - 16.4]	2.25
**'EF348_(ST16).fna'**	*Enterococcus raffinosus* NBRC 100492	13.2	[10.5 - 16.5]	25.9	[23.6 - 28.4]	13.6	[11.2 - 16.3]	1.82
**'19910_(ST116).fna'**	*Enterococcus massiliensis* AM1	13.2	[10.5 - 16.5]	25.9	[23.5 - 28.3]	13.6	[11.2 - 16.3]	2.36
**'BT00.E.21_(ST23).fasta'**	*Enterococcus italicus* DSM 15952	13.2	[10.4 - 16.5]	25.9	[23.5 - 28.3]	13.6	[11.2 - 16.3]	2.05

Query	Subject	d_0	C.I. d_0	d_4	C.I. d_4	d_6	C.I. d_6	Diff. G+C Percent
**'732_EFLS_(ST16).fna'**	*Candidatus* Enterococcus avicola CHK172-16539	13.3	[10.5 - 16.6]	25.9	[23.6 - 28.4]	13.7	[11.3 - 16.4]	0.49
**'R48_(ST228).fna'**	*Enterococcus casseliflavus* NBRC 100478	13.1	[10.3 - 16.4]	25.9	[23.6 - 28.4]	13.4	[11.1 - 16.2]	4.79
**'CL9314_(ST21).fasta'**	*Enterococcus devriesei* DSM 22802	13.3	[10.6 - 16.6]	25.9	[23.5 - 28.3]	13.7	[11.3 - 16.5]	2.81
**'CL9924_(ST6).fasta'**	*Enterococcus montenegrensis* CoE-012-22	13.4	[10.6 - 16.7]	25.9	[23.6 - 28.4]	13.8	[11.4 - 16.6]	0.28
**'LB00.E.122_(ST21).fasta'**	*Enterococcus devriesei* DSM 22802	13.3	[10.6 - 16.6]	25.9	[23.5 - 28.3]	13.7	[11.3 - 16.5]	2.89
**'EN24_(ST228).fna'**	*Enterococcus massiliensis* AM1	13.3	[10.5 - 16.6]	25.8	[23.5 - 28.3]	13.6	[11.3 - 16.4]	2.11
**'F1_(ST72).fna'**	*Enterococcus pseudoavium* NBRC 100491	13.4	[10.6 - 16.7]	25.8	[23.5 - 28.3]	13.7	[11.4 - 16.5]	2.57
**'EF348_(ST16).fna'**	*Enterococcus casseliflavus* NBRC 100478	13.0	[10.3 - 16.3]	25.8	[23.5 - 28.3]	13.4	[11.0 - 16.2]	4.82
**'302EA1_(ST122).fna'**	*Enterococcus dispar* ATCC 51266	13.6	[10.9 - 17.0]	25.8	[23.4 - 28.2]	14.0	[11.6 - 16.8]	0.29
**'EF349_(ST631).fna'**	*Vagococcus martis* D7T301	13.1	[10.4 - 16.4]	25.8	[23.5 - 28.3]	13.5	[11.1 - 16.3]	4.87
**'CVM N52662_(ST228).fna'**	*Enterococcus saigonensis* JCM 31193	13.5	[10.7 - 16.8]	25.8	[23.5 - 28.3]	13.8	[11.4 - 16.6]	1.25
**'CVM_N60027F_(ST862).fna'**	*Enterococcus porcinus* ATCC 700913	13.7	[10.9 - 17.0]	25.8	[23.5 - 28.3]	14.0	[11.6 - 16.8]	2.53
**'4928STDY7071351_(ST30).fna'**	*Enterococcus pseudoavium* NBRC 100491	13.4	[10.6 - 16.7]	25.8	[23.5 - 28.3]	13.7	[11.4 - 16.5]	2.88
**'CVM_N55265_(ST228).fna'**	*Enterococcus saigonensis* JCM 31193	13.4	[10.6 - 16.7]	25.8	[23.5 - 28.3]	13.7	[11.4 - 16.5]	1.31
**'EnGen0400_(ST86).fna'**	*Enterococcus montenegrensis* CoE-012-22	13.3	[10.6 - 16.7]	25.8	[23.4 - 28.3]	13.7	[11.3 - 16.5]	0.26
**'C138_(ST228).fna'**	*Enterococcus massiliensis* AM1	13.3	[10.5 - 16.6]	25.8	[23.4 - 28.3]	13.6	[11.3 - 16.4]	2.05
**'CVM_N52587_(STx).fna'**	*Enterococcus italicus* DSM 15952	13.4	[10.7 - 16.7]	25.8	[23.4 - 28.2]	13.8	[11.4 - 16.6]	1.66
**'F1_(ST72).fna'**	*Enterococcus porcinus* ATCC 700913	13.5	[10.7 - 16.8]	25.8	[23.5 - 28.3]	13.9	[11.5 - 16.7]	2.47
**'CL9943_(ST4).fasta'**	*Enterococcus casseliflavus* NBRC 100478	13.0	[10.3 - 16.3]	25.8	[23.4 - 28.2]	13.4	[11.0 - 16.2]	5.37
**'209EA1_(ST624).fna'**	*Enterococcus porcinus* ATCC 700913	13.6	[10.9 - 17.0]	25.8	[23.5 - 28.3]	14.0	[11.6 - 16.8]	2.71
**'D32_(ST40).fna'**	*Enterococcus casseliflavus* NBRC 100478	13.0	[10.3 - 16.3]	25.8	[23.5 - 28.3]	13.4	[11.0 - 16.2]	4.93
**'D32_(ST40).fna'**	*Enterococcus saigonensis* JCM 31193	13.4	[10.6 - 16.7]	25.8	[23.5 - 28.3]	13.7	[11.4 - 16.5]	1.15
**'Praia.S.M2.C4_(ST62).fasta'**	*Enterococcus xinjiangensis* JCM 30200	13.4	[10.6 - 16.7]	25.8	[23.5 - 28.3]	13.7	[11.3 - 16.5]	0.95
**'CL6870_(ST103).fasta'**	*Enterococcus mediterraneensis* Marseille-P4358	13.4	[10.7 - 16.8]	25.7	[23.3 - 28.2]	13.8	[11.4 - 16.6]	3.61

Query	Subject	d_0	C.I. d_0	d_4	C.I. d_4	d_6	C.I. d_6	Diff. G+C Percent
**'F1_(ST72).fna'**	*Vagococcus martis* D7T301	13.1	[10.4 - 16.4]	25.7	[23.4 - 28.2]	13.5	[11.1 - 16.3]	3.74
**'EN242_(ST82).fna'**	*Enterococcus montenegrensis* CoE-012-22	13.3	[10.6 - 16.7]	25.7	[23.4 - 28.2]	13.7	[11.3 - 16.5]	0.37
**'CVM_N54595_(ST192).fna'**	*Enterococcus gallinarum* NBRC 100675	13.1	[10.4 - 16.4]	25.7	[23.4 - 28.2]	13.5	[11.1 - 16.3]	2.35
**'CL9943_(ST4).fasta'**	*Enterococcus mediterraneensis* Marseille-P4358	13.4	[10.7 - 16.7]	25.7	[23.4 - 28.2]	13.8	[11.4 - 16.6]	3.91
**'KUB3007_(ST729).fna'**	*Enterococcus lactis* DSM 23655	13.2	[10.5 - 16.5]	25.7	[23.3 - 28.1]	13.6	[11.2 - 16.3]	0.96
**'209EA1_(ST624).fna'**	*Candidatus* Enterococcus avicola CHK172-16539	13.4	[10.6 - 16.7]	25.7	[23.3 - 28.1]	13.7	[11.3 - 16.5]	0.86
**'D32_(ST40).fna'**	*Enterococcus raffinosus* NBRC 100492	13.0	[10.3 - 16.3]	25.7	[23.4 - 28.2]	13.3	[11.0 - 16.1]	1.93
**'D32_(ST40).fna'**	*Enterococcus pseudoavium* NBRC 100491	13.3	[10.5 - 16.6]	25.7	[23.4 - 28.2]	13.7	[11.3 - 16.4]	2.64
**'G81_(ST1468).fna'**	*Enterococcus gallinarum* NBRC 100675	13.1	[10.4 - 16.4]	25.7	[23.3 - 28.1]	13.5	[11.1 - 16.2]	2.2
**'CL9772_(ST525).fasta'**	*Enterococcus mediterraneensis* Marseille-P4358	13.4	[10.6 - 16.7]	25.7	[23.4 - 28.2]	13.7	[11.4 - 16.5]	3.69
**'L14_(ST330).fna'**	*Enterococcus durans* NBRC 100479	13.2	[10.5 - 16.5]	25.7	[23.4 - 28.2]	13.6	[11.2 - 16.3]	0.19
**'Praia.S.M2.C4_(ST62).fasta'**	*Enterococcus mediterraneensis* Marseille-P4358	13.5	[10.7 - 16.8]	25.7	[23.4 - 28.2]	13.8	[11.4 - 16.6]	3.52
**'CL9172_(ST21).fasta'**	*Enterococcus devriesei* DSM 22802	13.3	[10.5 - 16.6]	25.6	[23.3 - 28.1]	13.7	[11.3 - 16.4]	2.8
**'CL9943_(ST4).fasta'**	*Enterococcus italicus* DSM 15952	13.2	[10.4 - 16.5]	25.6	[23.3 - 28.1]	13.6	[11.2 - 16.3]	2.17
**'19910_(ST116).fna'**	*Enterococcus cecorum* DSM 20682	13.1	[10.3 - 16.4]	25.6	[23.3 - 28.1]	13.4	[11.1 - 16.2]	0.91
**'CVM_N52587_(STx).fna'**	*Vagococcus bubulae* SS1994	13.3	[10.6 - 16.6]	25.6	[23.3 - 28.1]	13.7	[11.3 - 16.5]	3.94
**'T11_(ST65).fna'**	*Streptococcus gwangjuense* KCOM -1679T	12.7	[10.0 - 15.9]	25.6	[23.2 - 28.0]	13.1	[10.7 - 15.8]	2.55
**'D32_(ST40).fna'**	*Enterococcus thailandicus* DSM 21767	13.3	[10.6 - 16.6]	25.6	[23.2 - 28.1]	13.7	[11.3 - 16.5]	0.82
**'19910_(ST116).fna'**	*Enterococcus casseliflavus* NBRC 100478	13.0	[10.3 - 16.3]	25.6	[23.2 - 28.1]	13.4	[11.0 - 16.2]	5.07
**'CVM_N54595_(ST192).fna'**	*Enterococcus mediterraneensis* Marseille-P4358	13.4	[10.6 - 16.7]	25.6	[23.3 - 28.1]	13.7	[11.3 - 16.5]	3.49
**'E1_(ST40).fna'**	*Enterococcus massiliensis* AM1	13.3	[10.5 - 16.6]	25.6	[23.3 - 28.1]	13.6	[11.2 - 16.4]	2.3
**'L14_(ST330).fna'**	*Enterococcus montenegrensis* CoE-012-22	13.3	[10.6 - 16.6]	25.5	[23.1 - 27.9]	13.7	[11.3 - 16.5]	0.19
**'15224_(ST16).fna'**	*Enterococcus massiliensis* AM1	13.2	[10.5 - 16.5]	25.5	[23.2 - 28.0]	13.6	[11.2 - 16.4]	2.58
**'EN788_(ST82).fna'**	*Enterococcus montenegrensis* CoE-012-22	13.4	[10.6 - 16.7]	25.5	[23.2 - 28.0]	13.7	[11.3 - 16.5]	0.49

Query	Subject	d_0	C.I. d_0	d_4	C.I. d_4	d_6	C.I. d_6	Diff. G+C Percent
**'244_EFLS_(ST443).fna'**	*Enterococcus thailandicus* DSM 21767	13.4	[10.7 - 16.7]	25.5	[23.2 - 28.0]	13.8	[11.4 - 16.6]	0.89
**'BT00.E.21_(ST23).fasta'**	*Vagococcus martis* D7T301	13.1	[10.4 - 16.4]	25.5	[23.2 - 28.0]	13.5	[11.1 - 16.3]	3.34
**'Praia.S.M2.C4_(ST62).fasta'**	*Enterococcus xiangfangensis* NCIMB 14834	13.4	[10.6 - 16.7]	25.5	[23.1 - 27.9]	13.7	[11.3 - 16.5]	1.65
**'Praia.S.M2.C4_(ST62).fasta'**	*Enterococcus xiangfangensis* DSM 105127	13.4	[10.6 - 16.7]	25.5	[23.1 - 28.0]	13.7	[11.4 - 16.5]	1.66
**'CL8682_(ST769).fasta'**	*Enterococcus montenegrensis* CoE-012-22	13.3	[10.6 - 16.6]	25.5	[23.1 - 27.9]	13.7	[11.3 - 16.5]	0.11
**'F1_(ST72).fna'**	*Enterococcus larvae* BWM-S5	13.0	[10.3 - 16.3]	25.5	[23.1 - 27.9]	13.4	[11.0 - 16.2]	2.58
**'302EA1_(ST122).fna'**	*Vagococcus martis* D7T301	13.1	[10.4 - 16.4]	25.5	[23.2 - 28.0]	13.5	[11.1 - 16.3]	3.71
**'CL9314_(ST21).fasta'**	*Enterococcus massiliensis* AM1	13.3	[10.5 - 16.6]	25.5	[23.2 - 28.0]	13.6	[11.3 - 16.4]	2.23
**'D32_(ST40).fna'**	*Enterococcus xinjiangensis* JCM 30200	13.3	[10.5 - 16.6]	25.4	[23.1 - 27.9]	13.6	[11.2 - 16.4]	0.89
**'Merz96_(ST103).fna'**	*Enterococcus montenegrensis* CoE-012-22	13.3	[10.6 - 16.6]	25.4	[23.1 - 27.9]	13.7	[11.3 - 16.5]	0.18
**'LB00.E.122_(ST21).fasta'**	*Enterococcus thailandicus* DSM 21767	13.5	[10.7 - 16.8]	25.4	[23.1 - 27.9]	13.8	[11.4 - 16.6]	0.74
**'14EA1_(ST147).fna'**	*Enterococcus gallinarum* NBRC 100675	13.1	[10.4 - 16.4]	25.4	[23.1 - 27.9]	13.5	[11.1 - 16.2]	1.99
**'KUB3007_(ST729).fna'**	*Enterococcus montenegrensis* CoE-012-22	13.3	[10.6 - 16.6]	25.4	[23.0 - 27.8]	13.7	[11.3 - 16.5]	0.25
**'CL9797_(ST525).fasta'**	*Enterococcus montenegrensis* CoE-012-22	13.3	[10.6 - 16.6]	25.4	[23.1 - 27.9]	13.7	[11.3 - 16.5]	0.31
**'CL11199_(ST778).fasta'**	*Enterococcus montenegrensis* CoE-012-22	13.3	[10.6 - 16.6]	25.4	[23.1 - 27.9]	13.7	[11.3 - 16.5]	0.23
**'EF348_(ST16).fna'**	*Enterococcus saigonensis* JCM 31193	13.4	[10.6 - 16.7]	25.4	[23.1 - 27.9]	13.8	[11.4 - 16.6]	1.26
**'C138_(ST228).fna'**	*Enterococcus lactis* DSM 23655	13.3	[10.5 - 16.6]	25.4	[23.0 - 27.8]	13.6	[11.3 - 16.4]	0.54
**'209EA1_(ST624).fna'**	*Enterococcus lactis* CCM 8412	13.3	[10.5 - 16.6]	25.4	[23.0 - 27.8]	13.6	[11.2 - 16.4]	0.39
**'CL9943_(ST4).fasta'**	*Enterococcus montenegrensis* CoE-012-22	13.4	[10.6 - 16.7]	25.4	[23.1 - 27.9]	13.7	[11.3 - 16.5]	0.44
**'C138_(ST228).fna'**	*Enterococcus lactis* CCM 8412	13.3	[10.5 - 16.6]	25.4	[23.0 - 27.9]	13.6	[11.3 - 16.4]	0.53
**'CL9772_(ST525).fasta'**	*Enterococcus montenegrensis* CoE-012-22	13.3	[10.6 - 16.6]	25.4	[23.1 - 27.9]	13.7	[11.3 - 16.5]	0.23
**'CL11199_(ST778).fasta'**	*Enterococcus mediterraneensis* Marseille-P4358	13.4	[10.6 - 16.7]	25.4	[23.1 - 27.9]	13.8	[11.4 - 16.6]	3.7
**'G81_(ST1468).fna'**	*Enterococcus casseliflavus* NBRC 100478	13.0	[10.3 - 16.3]	25.4	[23.1 - 27.9]	13.4	[11.1 - 16.2]	4.81
**'E1_(ST40).fna'**	*Enterococcus italicus* DSM 15952	13.2	[10.4 - 16.5]	25.4	[23.0 - 27.9]	13.5	[11.2 - 16.3]	1.8

Query	Subject	d_0	C.I. d_0	d_4	C.I. d_4	d_6	C.I. d_6	Diff. G+C Percent
**'CVM N53420_(ST228).fna'**	*Enterococcus saigonensis* JCM 31193	13.4	[10.7 - 16.8]	25.4	[23.0 - 27.8]	13.8	[11.4 - 16.6]	1.16
**'CL6870_(ST103).fasta'**	*Enterococcus montenegrensis* CoE-012-22	13.3	[10.6 - 16.6]	25.4	[23.1 - 27.9]	13.7	[11.3 - 16.5]	0.15
**'KUB3007_(ST729).fna'**	*Enterococcus lactis* CCM 8412	13.2	[10.4 - 16.5]	25.4	[23.0 - 27.8]	13.5	[11.2 - 16.3]	0.95
**'244_EFLS_(ST443).fna'**	*Enterococcus casseliflavus* NBRC 100478	13.0	[10.3 - 16.3]	25.4	[23.1 - 27.9]	13.4	[11.0 - 16.2]	4.86
**'CL8682_(ST769).fasta'**	*Enterococcus mediterraneensis* Marseille-P4358	13.4	[10.7 - 16.7]	25.3	[23.0 - 27.8]	13.8	[11.4 - 16.6]	3.58
**'G81_(ST1468).fna'**	*Enterococcus durans* NBRC 100479	13.3	[10.6 - 16.7]	25.3	[23.0 - 27.8]	13.7	[11.3 - 16.5]	0.24
**'EN788_(ST82).fna'**	*Enterococcus mediterraneensis* Marseille-P4358	13.3	[10.6 - 16.6]	25.3	[22.9 - 27.7]	13.7	[11.3 - 16.5]	3.96
**'EF348_(ST16).fna'**	*Enterococcus durans* NBRC 100479	13.2	[10.5 - 16.5]	25.3	[23.0 - 27.8]	13.6	[11.2 - 16.4]	0.26
**'D32_(ST40).fna'**	*Enterococcus italicus* DSM 15952	13.2	[10.5 - 16.5]	25.3	[22.9 - 27.8]	13.6	[11.2 - 16.3]	1.72
**'4928STDY7071435_(ST40).fna'**	*Enterococcus saigonensis* JCM 31193	13.4	[10.6 - 16.7]	25.3	[22.9 - 27.7]	13.7	[11.4 - 16.5]	1.44
**'F1_(ST72).fna'**	*Enterococcus dispar* ATCC 51266	13.4	[10.7 - 16.7]	25.3	[23.0 - 27.8]	13.8	[11.4 - 16.6]	0.32
**'EF348_(ST16).fna'**	*Enterococcus massiliensis* AM1	13.2	[10.5 - 16.6]	25.3	[23.0 - 27.8]	13.6	[11.2 - 16.4]	2.11
**'Merz96_(ST103).fna'**	*Enterococcus saigonensis* JCM 31193	13.3	[10.6 - 16.6]	25.3	[23.0 - 27.8]	13.7	[11.3 - 16.4]	1.33
**'CL6870_(ST103).fasta'**	*Enterococcus porcinus* ATCC 700913	13.5	[10.8 - 16.9]	25.3	[22.9 - 27.7]	13.9	[11.5 - 16.7]	2.25
**'G81_(ST1468).fna'**	*Enterococcus songbeiensis* NCIMB 15179	13.2	[10.5 - 16.6]	25.3	[23.0 - 27.8]	13.6	[11.2 - 16.4]	2.18
**'D32_(ST40).fna'**	*Enterococcus mediterraneensis* Marseille-P4358	13.3	[10.6 - 16.6]	25.3	[22.9 - 27.8]	13.7	[11.3 - 16.5]	3.46
**'CVM N53420_(ST228).fna'**	*Enterococcus casseliflavus* NBRC 100478	13.0	[10.3 - 16.3]	25.3	[22.9 - 27.7]	13.4	[11.1 - 16.2]	4.91
**'EN242_(ST82).fna'**	*Enterococcus mediterraneensis* Marseille-P4358	13.3	[10.6 - 16.7]	25.3	[23.0 - 27.8]	13.7	[11.3 - 16.5]	3.84
**'209EA1_(ST624).fna'**	*Enterococcus thailandicus* DSM 21767	13.5	[10.7 - 16.8]	25.3	[23.0 - 27.8]	13.8	[11.4 - 16.6]	1.13
**'KUB3007_(ST729).fna'**	*Enterococcus cecorum* DSM 20682	13.1	[10.4 - 16.4]	25.3	[23.0 - 27.8]	13.5	[11.1 - 16.2]	0.8
**'F1_(ST72).fna'**	*Vagococcus bubulae* SS1994	13.3	[10.6 - 16.6]	25.2	[22.9 - 27.7]	13.7	[11.3 - 16.4]	3.94
**'BT00.E.21_(ST23).fasta'**	*Enterococcus porcinus* ATCC 700913	13.4	[10.7 - 16.8]	25.2	[22.8 - 27.6]	13.8	[11.4 - 16.6]	2.08
**'Merz96_(ST103).fna'**	*Enterococcus mediterraneensis* Marseille-P4358	13.4	[10.7 - 16.7]	25.2	[22.9 - 27.7]	13.8	[11.4 - 16.6]	3.29
**'4928STDY7071435_(ST40).fna'**	*Enterococcus mediterraneensis* Marseille-P4358	13.3	[10.6 - 16.6]	25.2	[22.9 - 27.7]	13.7	[11.3 - 16.5]	3.17

Query	Subject	d_0	C.I. d_0	d_4	C.I. d_4	d_6	C.I. d_6	Diff. G+C Percent
**'EN24_(ST228).fna'**	*Enterococcus saigonensis* JCM 31193	13.4	[10.7 - 16.8]	25.2	[22.9 - 27.7]	13.8	[11.4 - 16.6]	1.26
**'209EA1_(ST624).fna'**	*Enterococcus gallinarum* NBRC 100675	13.0	[10.3 - 16.3]	25.2	[22.8 - 27.6]	13.4	[11.1 - 16.2]	2.02
**'KUB3007_(ST729).fna'**	*Enterococcus raffinosus* NBRC 100492	13.1	[10.4 - 16.4]	25.2	[22.9 - 27.7]	13.5	[11.1 - 16.2]	2.19
**'CVM_N60027F_(ST862).fna'**	*Enterococcus casseliflavus* NBRC 100478	13.0	[10.3 - 16.3]	25.2	[22.9 - 27.7]	13.4	[11.1 - 16.2]	4.8
**'CVM_N54595_(ST192).fna'**	*Enterococcus saigonensis* JCM 31193	13.2	[10.5 - 16.6]	25.2	[22.9 - 27.7]	13.6	[11.2 - 16.4]	1.12
**'LB00.E.122_(ST21).fasta'**	*Enterococcus mediterraneensis* Marseille-P4358	13.3	[10.6 - 16.6]	25.2	[22.9 - 27.7]	13.7	[11.3 - 16.5]	3.55
**'4928STDY7071351_(ST30).fna'**	*Vagococcus bubulae* SS1994	13.3	[10.5 - 16.6]	25.2	[22.8 - 27.6]	13.6	[11.3 - 16.4]	3.63
**'CVM_N52587_(STx).fna'**	*Enterococcus lactis* DSM 23655	13.2	[10.5 - 16.5]	25.2	[22.9 - 27.7]	13.6	[11.2 - 16.4]	0.64
**'EnGen0400_(ST86).fna'**	*Enterococcus saigonensis* JCM 31193	13.4	[10.6 - 16.7]	25.2	[22.8 - 27.6]	13.8	[11.4 - 16.6]	1.41
**'302EA1_(ST122).fna'**	*Enterococcus devriesei* DSM 22802	13.3	[10.5 - 16.6]	25.2	[22.9 - 27.7]	13.6	[11.3 - 16.4]	2.76
**'CVM_N52587_(STx).fna'**	*Enterococcus lactis* CCM 8412	13.2	[10.5 - 16.5]	25.2	[22.8 - 27.6]	13.6	[11.2 - 16.4]	0.63
**'CVM N53420_(ST228).fna'**	*Enterococcus durans* NBRC 100479	13.3	[10.6 - 16.6]	25.2	[22.9 - 27.7]	13.7	[11.3 - 16.5]	0.35
**'G81_(ST1468).fna'**	*Enterococcus thailandicus* DSM 21767	13.4	[10.7 - 16.8]	25.1	[22.8 - 27.6]	13.8	[11.4 - 16.6]	0.94
**'CVM_N52587_(STx).fna'**	*Enterococcus casseliflavus* NBRC 100478	13.0	[10.3 - 16.3]	25.1	[22.8 - 27.6]	13.4	[11.1 - 16.2]	4.86
**'CL9172_(ST21).fasta'**	*Enterococcus mediterraneensis* Marseille-P4358	13.3	[10.6 - 16.6]	25.1	[22.8 - 27.6]	13.7	[11.3 - 16.5]	3.46
**'4928STDY7071351_(ST30).fna'**	*Enterococcus casseliflavus* NBRC 100478	13.0	[10.3 - 16.3]	25.1	[22.8 - 27.6]	13.4	[11.0 - 16.2]	5.17
**'CL9943_(ST4).fasta'**	*Enterococcus porcinus* ATCC 700913	13.4	[10.7 - 16.8]	25.1	[22.8 - 27.6]	13.8	[11.4 - 16.6]	1.96
**'EF349_(ST631).fna'**	*Enterococcus saigonensis* JCM 31193	13.4	[10.6 - 16.7]	25.1	[22.8 - 27.6]	13.7	[11.3 - 16.5]	2.35
**'F_(ST712).fna'**	*Enterococcus dispar* ATCC 51266	13.4	[10.6 - 16.7]	25.1	[22.7 - 27.5]	13.7	[11.3 - 16.5]	0.42
**'Praia.S.M2.C4_(ST62).fasta'**	*Enterococcus cecorum* DSM 20682	13.1	[10.4 - 16.4]	25.1	[22.8 - 27.6]	13.5	[11.1 - 16.3]	0.99
**'Praia.S.M2.C4_(ST62).fasta'**	*Vagococcus martis* D7T301	13.2	[10.5 - 16.5]	25.1	[22.8 - 27.6]	13.6	[11.2 - 16.4]	3.61
**'BT00.E.21_(ST23).fasta'**	*Enterococcus mediterraneensis* Marseille-P4358	13.4	[10.6 - 16.7]	25.1	[22.8 - 27.6]	13.7	[11.3 - 16.5]	3.79
**'4928STDY7071435_(ST40).fna'**	*Enterococcus pseudoavium* NBRC 100491	13.4	[10.7 - 16.8]	25.1	[22.7 - 27.5]	13.8	[11.4 - 16.6]	2.35
**'CL9797_(ST525).fasta'**	*Enterococcus massiliensis* AM1	13.2	[10.5 - 16.6]	25.1	[22.7 - 27.6]	13.6	[11.2 - 16.4]	2.53
**'732_EFLS_(ST16).fna'**	*Enterococcus cecorum* DSM 20682	13.1	[10.4 - 16.4]	25.1	[22.8 - 27.6]	13.5	[11.1 - 16.3]	0.99

Query	Subject	d_0	C.I. d_0	d_4	C.I. d_4	d_6	C.I. d_6	Diff. G+C Percent
**'CVM_N52587_(STx).fna'**	*Enterococcus porcinus* ATCC 700913	13.6	[10.9 - 17.0]	25.1	[22.8 - 27.6]	14.0	[11.6 - 16.8]	2.47
**'732_EFLS_(ST16).fna'**	*Enterococcus durans* NBRC 100479	13.2	[10.5 - 16.5]	25.1	[22.8 - 27.6]	13.6	[11.2 - 16.4]	0.43
**'LB00.E.122_(ST21).fasta'**	*Enterococcus saigonensis* JCM 31193	13.4	[10.6 - 16.7]	25.1	[22.7 - 27.5]	13.7	[11.4 - 16.5]	1.06
**'14EA1_(ST147).fna'**	*Enterococcus italicus* DSM 15952	13.2	[10.5 - 16.5]	25.1	[22.8 - 27.6]	13.6	[11.2 - 16.4]	1.38
**'F1_(ST72).fna'**	*Enterococcus cecorum* DSM 20682	13.1	[10.4 - 16.4]	25.1	[22.8 - 27.6]	13.5	[11.1 - 16.3]	1.12
**'CL9314_(ST21).fasta'**	*Enterococcus mediterraneensis* Marseille-P4358	13.3	[10.6 - 16.6]	25.1	[22.8 - 27.6]	13.7	[11.3 - 16.5]	3.47
**'LB00.E.122_(ST21).fasta'**	*Enterococcus casseliflavus* NBRC 100478	13.0	[10.3 - 16.3]	25.0	[22.6 - 27.4]	13.4	[11.0 - 16.1]	5.01
**'E1_(ST40).fna'**	*Enterococcus cecorum* DSM 20682	13.1	[10.4 - 16.4]	25.0	[22.7 - 27.5]	13.5	[11.1 - 16.3]	0.98
**'732_EFLS_(ST16).fna'**	*Enterococcus raffinosus* NBRC 100492	13.2	[10.4 - 16.5]	25.0	[22.7 - 27.5]	13.5	[11.2 - 16.3]	1.99
**'732_EFLS_(ST16).fna'**	*Enterococcus saigonensis* JCM 31193	13.4	[10.6 - 16.7]	25.0	[22.7 - 27.5]	13.7	[11.4 - 16.5]	1.09
**'4928STDY7071351_(ST30).fna'**	*Enterococcus cecorum* DSM 20682	13.1	[10.4 - 16.4]	25.0	[22.7 - 27.5]	13.5	[11.1 - 16.2]	0.81
**'R48_(ST228).fna'**	*Enterococcus devriesei* DSM 22802	13.1	[10.4 - 16.4]	25.0	[22.6 - 27.4]	13.5	[11.1 - 16.2]	2.67
**'CL9314_(ST21).fasta'**	*Enterococcus casseliflavus* NBRC 100478	13.0	[10.3 - 16.3]	25.0	[22.6 - 27.4]	13.4	[11.0 - 16.1]	4.93
**'CL9314_(ST21).fasta'**	*Enterococcus saigonensis* JCM 31193	13.4	[10.6 - 16.7]	25.0	[22.7 - 27.5]	13.7	[11.3 - 16.5]	1.14
**'D32_(ST40).fna'**	*Enterococcus devriesei* DSM 22802	13.2	[10.5 - 16.5]	25.0	[22.7 - 27.5]	13.6	[11.2 - 16.4]	2.8
**'302EA1_(ST122).fna'**	*Enterococcus xinjiangensis* JCM 30200	13.3	[10.5 - 16.6]	25.0	[22.7 - 27.5]	13.6	[11.2 - 16.4]	0.85
**'CL9314_(ST21).fasta'**	*Enterococcus cecorum* DSM 20682	13.2	[10.4 - 16.5]	25.0	[22.7 - 27.5]	13.5	[11.2 - 16.3]	1.04
**'BT00.E.21_(ST23).fasta'**	*Enterococcus cecorum* DSM 20682	13.0	[10.3 - 16.3]	25.0	[22.7 - 27.5]	13.4	[11.0 - 16.2]	0.73
**'L14_(ST330).fna'**	*Enterococcus casseliflavus* NBRC 100478	13.1	[10.4 - 16.4]	25.0	[22.7 - 27.5]	13.5	[11.1 - 16.2]	4.75
**'L14_(ST330).fna'**	*Enterococcus saigonensis* JCM 31193	13.3	[10.6 - 16.6]	25.0	[22.7 - 27.5]	13.7	[11.3 - 16.5]	1.33
**'CL9172_(ST21).fasta'**	*Enterococcus cecorum* DSM 20682	13.2	[10.4 - 16.5]	25.0	[22.7 - 27.5]	13.5	[11.2 - 16.3]	1.06
**'4928STDY7071351_(ST30).fna'**	*Enterococcus dispar* ATCC 51266	13.5	[10.8 - 16.9]	25.0	[22.7 - 27.5]	13.9	[11.5 - 16.7]	0.01
**'CL9314_(ST21).fasta'**	*Enterococcus italicus* DSM 15952	13.2	[10.5 - 16.5]	25.0	[22.7 - 27.5]	13.6	[11.2 - 16.4]	1.73
**'C138_(ST228).fna'**	*Enterococcus saigonensis* JCM 31193	13.4	[10.6 - 16.7]	25.0	[22.7 - 27.5]	13.7	[11.3 - 16.5]	1.32
**'E1_(ST40).fna'**	*Enterococcus mediterraneensis* Marseille-P4358	13.3	[10.6 - 16.7]	25.0	[22.7 - 27.5]	13.7	[11.3 - 16.5]	3.54
**'CL9172_(ST21).fasta'**	*Enterococcus saigonensis* JCM 31193	13.4	[10.6 - 16.7]	25.0	[22.7 - 27.5]	13.7	[11.3 - 16.5]	1.15

Query	Subject	d_0	C.I. d_0	d_4	C.I. d_4	d_6	C.I. d_6	Diff. G+C Percent
**'19910_(ST116).fna'**	*Enterococcus porcinus* ATCC 700913	13.5	[10.8 - 16.8]	25.0	[22.6 - 27.4]	13.9	[11.5 - 16.7]	2.26
**'209EA1_(ST624).fna'**	*Enterococcus lactis* DSM 23655	13.2	[10.5 - 16.6]	25.0	[22.7 - 27.5]	13.6	[11.2 - 16.4]	0.4
**'EN242_(ST82).fna'**	*Enterococcus saigonensis* JCM 31193	13.3	[10.6 - 16.6]	24.9	[22.6 - 27.4]	13.7	[11.3 - 16.5]	0.77
**'209EA1_(ST624).fna'**	*Enterococcus devriesei* DSM 22802	13.1	[10.4 - 16.4]	24.9	[22.5 - 27.3]	13.5	[11.1 - 16.3]	2.5
**'C138_(ST228).fna'**	*Enterococcus montenegrensis* CoE-012-22	13.2	[10.5 - 16.6]	24.9	[22.6 - 27.4]	13.6	[11.2 - 16.4]	0.18
**'CVM_N55265_(ST228).fna'**	*Enterococcus casseliflavus* NBRC 100478	13.0	[10.3 - 16.3]	24.9	[22.6 - 27.4]	13.4	[11.0 - 16.2]	4.77
**'EN788_(ST82).fna'**	*Enterococcus saigonensis* JCM 31193	13.3	[10.6 - 16.6]	24.9	[22.5 - 27.3]	13.7	[11.3 - 16.5]	0.65
**'EnGen0400_(ST86).fna'**	*Enterococcus durans* NBRC 100479	13.2	[10.5 - 16.5]	24.9	[22.6 - 27.4]	13.6	[11.2 - 16.3]	0.11
**'CL9172_(ST21).fasta'**	*Enterococcus thailandicus* DSM 21767	13.4	[10.7 - 16.8]	24.9	[22.6 - 27.4]	13.8	[11.4 - 16.6]	0.83
**'CVM N52662_(ST228).fna'**	*Enterococcus casseliflavus* NBRC 100478	13.0	[10.3 - 16.3]	24.9	[22.5 - 27.3]	13.4	[11.0 - 16.2]	4.83
**'CVM_N55265_(ST228).fna'**	*Enterococcus montenegrensis* CoE-012-22	13.2	[10.5 - 16.6]	24.9	[22.6 - 27.4]	13.6	[11.2 - 16.4]	0.17
**'CL9172_(ST21).fasta'**	*Enterococcus casseliflavus* NBRC 100478	13.0	[10.3 - 16.3]	24.9	[22.6 - 27.4]	13.4	[11.0 - 16.1]	4.92
**'CVM_N60027F_(ST862).fna'**	*Enterococcus thailandicus* DSM 21767	13.4	[10.6 - 16.7]	24.8	[22.4 - 27.2]	13.7	[11.3 - 16.5]	0.95
**'Praia.S.M2.C4_(ST62).fasta'**	*Enterococcus gallinarum* NBRC 100675	13.2	[10.5 - 16.6]	24.8	[22.4 - 27.2]	13.6	[11.2 - 16.4]	2.39
**'209EA1_(ST624).fna'**	*Enterococcus dispar* ATCC 51266	13.5	[10.7 - 16.8]	24.8	[22.5 - 27.3]	13.9	[11.5 - 16.7]	0.56
**'C116_(ST228).fna'**	*Enterococcus massiliensis* AM1	13.2	[10.5 - 16.5]	24.8	[22.5 - 27.3]	13.6	[11.2 - 16.4]	2.06
**'LB00.E.122_(ST21).fasta'**	*Enterococcus italicus* DSM 15952	13.2	[10.5 - 16.5]	24.8	[22.5 - 27.3]	13.6	[11.2 - 16.3]	1.81
**'CL9172_(ST21).fasta'**	*Enterococcus italicus* DSM 15952	13.2	[10.5 - 16.5]	24.8	[22.5 - 27.3]	13.6	[11.2 - 16.3]	1.72
**'F1_(ST72).fna'**	*Enterococcus raffinosus* NBRC 100492	13.0	[10.3 - 16.3]	24.8	[22.5 - 27.3]	13.4	[11.0 - 16.2]	1.86
**'E1_(ST40).fna'**	*Enterococcus saigonensis* JCM 31193	13.4	[10.6 - 16.7]	24.8	[22.5 - 27.3]	13.7	[11.3 - 16.5]	1.07
**'D32_(ST40).fna'**	*Enterococcus porcinus* ATCC 700913	13.5	[10.8 - 16.8]	24.8	[22.5 - 27.3]	13.9	[11.5 - 16.7]	2.4
**'R48_(ST228).fna'**	*Enterococcus thailandicus* DSM 21767	13.4	[10.7 - 16.7]	24.8	[22.4 - 27.2]	13.8	[11.4 - 16.6]	0.96
**'4928STDY7071435_(ST40).fna'**	*Enterococcus cecorum* DSM 20682	13.1	[10.4 - 16.4]	24.8	[22.4 - 27.2]	13.5	[11.1 - 16.2]	1.34
**'EN24_(ST228).fna'**	*Enterococcus italicus* DSM 15952	13.3	[10.5 - 16.6]	24.8	[22.5 - 27.3]	13.6	[11.3 - 16.4]	1.61
**'14EA1_(ST147).fna'**	*Enterococcus raffinosus* NBRC 100492	13.0	[10.3 - 16.3]	24.8	[22.5 - 27.3]	13.4	[11.0 - 16.2]	1.59

Query	Subject	d_0	C.I. d_0	d_4	C.I. d_4	d_6	C.I. d_6	Diff. G+C Percent
**'CL9797_(ST525).fasta'**	*Enterococcus casseliflavus* NBRC 100478	13.0	[10.3 - 16.3]	24.8	[22.4 - 27.2]	13.4	[11.0 - 16.2]	5.24
**'14EA1_(ST147).fna'**	*Vagococcus martis* D7T301	13.1	[10.4 - 16.4]	24.8	[22.5 - 27.3]	13.5	[11.1 - 16.3]	4.0
**'302EA1_(ST122).fna'**	*Enterococcus italicus* DSM 15952	13.2	[10.4 - 16.5]	24.8	[22.5 - 27.3]	13.5	[11.2 - 16.3]	1.68
**'CVM_N52587_(STx).fna'**	*Enterococcus montenegrensis* CoE-012-22	13.2	[10.5 - 16.6]	24.8	[22.5 - 27.3]	13.6	[11.2 - 16.4]	0.07
**'E1_(ST40).fna'**	*Enterococcus casseliflavus* NBRC 100478	13.0	[10.3 - 16.3]	24.8	[22.5 - 27.3]	13.4	[11.0 - 16.2]	5.0
**'CL9924_(ST6).fasta'**	*Enterococcus dispar* ATCC 51266	13.5	[10.8 - 16.8]	24.8	[22.4 - 27.2]	13.9	[11.5 - 16.7]	0.03
**'CVM_N54595_(ST192).fna'**	*Enterococcus casseliflavus* NBRC 100478	13.1	[10.3 - 16.4]	24.8	[22.5 - 27.3]	13.4	[11.1 - 16.2]	4.96
**'CVM N53420_(ST228).fna'**	*Enterococcus massiliensis* AM1	13.2	[10.5 - 16.5]	24.8	[22.4 - 27.2]	13.6	[11.2 - 16.4]	2.21
**'CVM N52662_(ST228).fna'**	*Enterococcus montenegrensis* CoE-012-22	13.2	[10.5 - 16.6]	24.8	[22.5 - 27.3]	13.6	[11.2 - 16.4]	0.11
**'14EA1_(ST147).fna'**	*Enterococcus dispar* ATCC 51266	13.5	[10.7 - 16.8]	24.8	[22.5 - 27.3]	13.8	[11.4 - 16.6]	0.59
**'L14_(ST330).fna'**	*Enterococcus mediterraneensis* Marseille-P4358	13.3	[10.6 - 16.6]	24.7	[22.4 - 27.2]	13.7	[11.3 - 16.5]	3.28

Table 4: Strains in your dataset

Joint dataset of automatically determined closest type strains (if this mode was chosen), manually selected type strains (if selected accordingly) and the provided user strains, if provided (marked in yellow).

Strain	Authority	Other deposits	Synonyms	Base pairs	Percent G+C	No. proteins	Goldstamp	Bioproject accession	Biosample accession	Assembly accession	IMG OID
Streptococcus gwangjuense KCOM -1679T	Park et al. 2019	JCM 33299; ChDC -B345	*Streptococcus gwangjuense*	1972 481	40.2	1829		PRJNA493538	SAMN10135937	GCA_003627155	
Amedibacillus hominis NSJ-176	Abdugheni et al. 2023	CGMCC 1.17933; KCTC 25355	*Amedibacillus hominis*	4345 597	35.5	4183		PRJNA782776	SAMN23395456	GCA_022487425	
Enterococcus thailandicus DSM 21767	Tanasupawat et al. 2008	KCTC 13134; NRIC 107; NBRC 101867; FP48-3; PCU 282; TISTR 933	*Enterococcus thailandicus*	2773 849	36.6	2617		PRJNA270385	SAMN03267187	GCA_001886265	
Enterococcus devriesei DSM 22802	Švec et al. 2005	LMG 14595; CCUG 37865; CCM 7299	*Enterococcus devriesei*	3320 590	40.2	3135		PRJNA270385	SAMN03267169	GCA_001885905	
Enterococcus canintestini DSM 21207	Naser et al. 2005	LMG 13590; CCUG 37857; CCUG 51312; CCM 7285	*Enterococcus canintestini*	2689 934	36.2	2517		PRJNA270385	SAMN03267163	GCA_001885735	
Enterococcus hermanniensis DSM 17122	Koort et al. 2004	LMG 12317; CCUG 48100	*Enterococcus hermanniensis*	2613 500	37.4	2550		PRJNA270385	SAMN03267173	GCA_001885945	
Vagococcus martis D7T301	Tak et al. 2017	KCTC 21069; JCM 31178	*Vagococcus martis*	2561 923	33.8	2472	Gp0208312	PRJNA224116	SAMN06241148	GCF_002026305	

Strain	Authority	Other deposits	Synonyms	Base pairs	Percent G+C	No. proteins	Goldstamp	Bioproject accession	Biosample accession	Assembly accession	IMG OID
Enterococcus cecorum DSM 20682	(Devriese et al. 1983) Williams et al. 1989	A60; LMG 12902; CIP 103676; NCIMB 702674; ATCC 43198; CCUG 27299; JCM 8724; NBRC 100674; NCTC 12421; NCDO 2674; NCFB 2674	*Enterococcus cecorum*; *Streptococcus cecorum*	2338 606	36.4	2363	Gp0013018	PRJNA165387	SAMN02256445	GCA_000379745	2515154031
Enterococcus wangshanyuanii MN05	Jin et al. 2017	CGMCC 1.15942; DSM 104047; MN05	*Enterococcus wangshanyuanii*	4155 954	37.3	4172	Gp0260508	PRJNA224116	SAMN07171652	GCF_002197645	
Enterococcus lemanii DSM 105069	Cotta et al. 2013	NRRL B-59661; CCUG 61260; PC32	*Enterococcus lemanii*	2715 351	37.2	2603	Gp0505788				2901272077
Enterococcus xiangfangensis DSM 105127	Li et al. 2014	11097; LMG 27495; NCIMB 14834; DSM 105127	*Enterococcus xiangfangensis*	2654 637	39.0	2594	Gp0505791				2901274788
Enterococcus larvae BWM-S5	Kim et al. 2022	CCM 9075; KACC 22156	*Enterococcus larvae*	4160 192	40.1	3863		PRJNA682502	SAMN16992986	GCA_017830045	
Candidatus Enterococcus avicola CHK172-16539	Gilroy et al. 2021		*Candidatus* Enterococcus avicola	2214 113	36.9	2261		PRJNA543206	SAMN15816900	GCA_019116505	
Enterococcus rivorum DSM 104544	Niemi et al. 2012	LMG 25899; DSM 104544; CCM 7986; HAMBI 3055; S299	*Enterococcus rivorum*	3791 979	34.9	3596	Gp0505789				2913591362

Strain	Authority	Other deposits	Synonyms	Base pairs	Percent G+C	No. proteins	Goldstamp	Bioproject accession	Biosample accession	Assembly accession	IMG OID
Enterococcus alcedinis DSM 107709	Frolková et al. 2013	LMG 27164; DSM 107709; CCM 8433; L34	*Enterococcus alcedinis*	2676 781	37.6	2487	Gp0505786				2913599697
Vagococcus bubulae SS1994	Shewmaker et al. 2019	LMG 30164; CCUG 70831	*Vagococcus bubulae*	2493 517	33.5	2360		PRJNA359498	SAMN06609019	GCA_003950315	
Enterococcus songbeiensis NCIMB 15179	Li and Gu 2019	85-4; CCM 8923	*Enterococcus songbeiensis*	2268 269	39.7	2210		PRJDB7793	SAMD00164309	GCA_005405265	
Enterococcus montenegrensis CoE-012-22	Daza-Prieto et al. 2024	NCIMB 15468; DSM 115843	*Enterococcus montenegrensis*	2829 429	37.4	2580		PRJNA940651	SAMN33576457	GCA_029983095	
Candidatus Tetragenococcu s pullicola CHK175-10598	Gilroy et al. 2021		*Candidatus* Tetragenococcu s pullicola	2601 468	36.4	2483		PRJNA543206	SAMN15816709	GCA_019115705	
Enterococcus xinjiangensis JCM 30200	Ren et al. 2020	48; CCTCC AB 2014041	*Enterococcus xinjiangensis*	2569 341	38.3	2477		PRJNA224116	SAMD00255146	GCF_015751065	
Enterococcus rotai DSM 102982	Sedláček et al. 2013	LMG 26678; CCUG 61593; DSM 102982; CCM 4630	*Enterococcus rotai*	3823 370	36.1	3602	Gp0538786				2928218588
Enterococcus lactis DSM 23655	Morandi et al. 2012 emend. Li and Gu 2021	LMG 25958; DSM 23655; CCM 8412; BT159	*Enterococcus lactis*	2859 220	38.1	2799	Gp0538720				2928549275
Enterococcus saigonensis JCM 31193	Harada et al. 2016	CCUG 68827; VE80	*Enterococcus saigonensis*	2844 986	36.3	2700		PRJDB9118	SAMD00198474	GCA_011397115	
Arthrobacter sulfonylureivoran s LAM7117	Han et al. 2021	CGMCC 1.16681; JCM 32824	*Arthrobacter sulfonylureivoran s*	4090 012	66.0	3772		PRJNA503709	SAMN10371981	GCA_004000035	

Strain	Authority	Other deposits	Synonyms	Base pairs	Percent G+C	No. proteins	Goldstamp	Bioproject accession	Biosample accession	Assembly accession	IMG OID
Enterococcus lactis CCM 8412	Morandi et al. 2012 emend. Li and Gu 2021	LMG 25958; DSM 23655; CCM 8412; BT159	*Enterococcus lactis*	2862 704	38.1	2745		PRJDB7793	SAMD00255145	GCA_015751045	
Enterococcus massiliensis AM1	Le Page et al. 2016	DSM 100308; CSUR P1927	*Enterococcus massiliensis*	2711 493	39.6	2614	Gp0144269	PRJEB9522	SAMEA3443460	GCA_001050095	
Enterococcus mediterraneensis Marseille-P4358	Takakura et al. 2019	CSUR P4358	*Enterococcus mediterraneensis*	2699 190	40.9	2598		PRJNA224116	SAMEA4983427	GCF_900604485	
Aequorivita lutea q18	Zhang et al. 2020	CICC 24821; KCTC 72764	*Aequorivita lutea*	3286 739	42.7	3418		PRJNA565545	SAMN12751400	GCA_009668655	
Enterococcus italicus DSM 15952	Fortina et al. 2004	LMG 22039; CCUG 50447; TP1.5	*Enterococcus italicus*	2302 158	39.1	2405	Gp0005449	PRJNA53039	SAMN00215989	GCA_000185365	649989943
Enterococcus dispar ATCC 51266	Collins et al. 1991	LMG 13521; CIP 103646; NCIMB 13000; CCUG 33309; DSM 6630; NBRC 100678; E18-1; HAMBI 2231; NCFB 2821	*Enterococcus dispar*	2812 918	37.2	2637	Gp0037006	PRJNA191887	SAMN02596962	GCA_000407585	2545824666
Enterococcus haemoperoxidus ATCC BAA-382	Švec et al. 2001	440; LMG 19487; CIP 107129; CCUG 45916; DSM 15920; NBRC 100709; CCM 4851	*Enterococcus haemoperoxidus*	3578 766	35.7	3207	Gp0037011	PRJNA191901	SAMN02596950	GCA_000407165	2545824662

Strain	Authority	Other deposits	Synonyms	Base pairs	Percent G+C	No. proteins	Goldstamp	Bioproject accession	Biosample accession	Assembly accession	IMG OID
Enterococcus caccae ATCC BAA-1240	Carvalho et al. 2006	2215-02; CCUG 51564; DSM 19114; SS-1777	*Enterococcus caccae*	3547 077	35.8	3246	Gp0037005	PRJNA191898	SAMN02596947	GCA_000407145	2545824664
Enterococcus moraviensis ATCC BAA-383	Švec et al. 2001	330; LMG 19486; CIP 107130; CCUG 45913; DSM 15919; NBRC 100710; CCM 4856	*Enterococcus moraviensis*	3586 110	36.0	3341	Gp0037015	PRJNA191905	SAMN02596953	GCA_000407445	2545824649
Enterococcus porcinus ATCC 700913	Teixeira et al. 2001	CIP 107172; NCIMB 13634; CCUG 43229; DS 1390-83	*Enterococcus porcinus*	3056 699	35.0	2821	Gp0036838	PRJNA191912	SAMN02596958	GCA_000407205	2545824674
Enterococcus saccharolyticus ATCC 43076	(Farrow et al. 1985) Rodrigues and Collins 1991 emend. Chen et al. 2013	LMG 11427; CIP 103246; NCIMB 702594; CCUG 27643; CCUG 33311; DSM 20726; JCM 8734; NBRC 100493; HAMBI 1576; NCDO 2594; NCFB 2594	*Enterococcus saccharolyticus*; *Enterococcus saccharolyticus* subsp. *saccharolyticus*; *Streptococcus saccharolyticus*	2591 178	36.9	2582	Gp0020466	PRJNA191890	SAMN02596896	GCA_000407285	2541047445
Enterococcus canis NBRC 100695	De Graef et al. 2003	LMG 12316; CCUG 46666; DSM 17029	*Enterococcus canis*	2854 214	41.9	2745	Gp0075767	PRJDB1349	SAMD00046312	GCA_001544375	

Strain	Authority	Other deposits	Synonyms	Base pairs	Percent G+C	No. proteins	Goldstamp	Bioproject accession	Biosample accession	Assembly accession	IMG OID
Enterococcus alcedinis CCM8433	Frolková et al. 2013	LMG 27164; DSM 107709; CCM 8433; L34	*Enterococcus alcedinis*	2682 154	37.6	2491		PRJDB10511	SAMD00244887	GCA_014635985	
Enterococcus casseliflavus NBRC 100478	(ex Vaughan et al. 1979) Collins et al. 1984	LMG 10745; CIP 103018; NRRL B-3502; NCIMB 11449; ATCC 25788; CCUG 18657; DSM 20680; JCM 8723; NCTC 12361; MUTK 20; NCDO 2372; NCFB 2372; NCIB 11449	*Enterococcus casseliflavus*; *Streptococcus casseliflavus*; *Streptococcus faecium* subsp. *casseliflavus*	3498 264	42.4	3339	Gp0023879	PRJDB260	SAMD00045727	GCA_001544095	
Enterococcus durans NBRC 100479	(ex Sherman and Wing 1937) Collins et al. 1984	98D; ATCC 19432; DSM 20633; NCTC 8307; CCM 5612; NCDO 596; NCFB 596	*Enterococcus durans*; *Streptococcus durans*	3017 301	37.8	2874	Gp0023771	PRJDB261	SAMD00045728	GCA_001544215	

Strain	Authority	Other deposits	Synonyms	Base pairs	Percent G+C	No. proteins	Goldstamp	Bioproject accession	Biosample accession	Assembly accession	IMG OID
Enterococcus faecalis NBRC 100480	(Andrewes and Horder 1906) Schleifer and Kilpper-Bälz 1984	LMG 7937; CIP 103015; NCIMB 775; ATCC 19433; ATCC 19433-U; CCUG 19916; DSM 20478; JCM 5803; JCM 8726; NBIMCC 3360; NBRC 100481; NCTC 775; HAMBI 1711; NCAIM B.01312; NCDO 581; NCFB 581; NCIB 775	*Enterococcus faecalis*; *Streptococcus faecalis*	2833 208	37.5	2734	Gp0023751	PRJDB262	SAMD00045729	GCA_001544235	
Enterococcus gallinarum NBRC 100675	(Bridge and Sneath 1982) Collins et al. 1984	LMG 13129; CIP 103013; NCIMB 702313; NCIMB 702313 (formerly NCDO 2313); ATCC 49573; CCUG 18658; DSM 24841; JCM 8728; NCTC 12359; F87/276; NCDO 2313; NCFB 2313; PB21	*Enterococcus gallinarum*; *Streptococcus gallinarum*	3774 884	39.8	3600	Gp0023878	PRJDB264	SAMD00045734	GCA_001544275	

Strain	Authority	Other deposits	Synonyms	Base pairs	Percent G+C	No. proteins	Goldstamp	Bioproject accession	Biosample accession	Assembly accession	IMG OID
Enterococcus pseudoavium NBRC 100491	Collins et al. 1989	LMG 11426; CIP 103647; NCIMB 13084; ATCC 49372; CCUG 33310; DSM 5632; JCM 8732; NCDO 2138; NCFB 2138	*Enterococcus pseudoavium*	2731 874	40.1	2588	Gp0023579	PRJDB265	SAMD00045731	GCA_001544295	
Enterococcus raffinosus NBRC 100492	Collins et al. 1989	LMG 12888; CIP 103329; NCIMB 12901; ATCC 49427; CCUG 29292; DSM 5633; JCM 8733; NCTC 12192; Strain 1789/79	*Enterococcus raffinosus*	4210 362	39.4	4064	Gp0023853	PRJDB266	SAMD00045732	GCA_001544115	
Enterococcus wangshanyuanii CGMCC 1.15942	Jin et al. 2017	CGMCC 1.15942; DSM 104047; MN05	*Enterococcus wangshanyuanii*	3967 296	37.3	3969		PRJDB10509	SAMD00245100	GCA_014644255	
Enterococcus quebecensis LMG 26306	Sistek et al. 2012	CCUG 59306; DSM 23327; CCRI 16985	*Enterococcus quebecensis*	3162 767	35.1	2788		PRJNA224116	SAMN05420588	GCF_001730365	
Enterococcus ureasiticus DSM 23328	Sistek et al. 2012	LMG 26304; CCUG 59304; CCRI 16986	*Enterococcus ureasiticus*	3585 398	35.8	3255		PRJNA224116	SAMN05420582	GCF_001730285	
Enterococcus termitis LMG 8895	Švec et al. 2006	DSM 22803; CCM 7300	*Enterococcus termitis*	4155 153	36.8	3932		PRJNA224116	SAMN05420590	GCF_001730305	

Strain	Authority	Other deposits	Synonyms	Base pairs	Percent G+C	No. proteins	Goldstamp	Bioproject accession	Biosample accession	Assembly accession	IMG OID
Enterococcus silesiacus LMG 23085	Švec et al. 2006	DSM 22801; CCM 7319; W442	*Enterococcus silesiacus*	3928 141	36.4	3559	Gp0150893	PRJNA226735	SAMN04296138	GCA_001465115	
Enterococcus plantarum LMG 26214	Švec et al. 2012	DSM 26408; CCM 7889; C27	*Enterococcus plantarum*	3135 404	36.0	2907		PRJNA224116	SAMN05420586	GCF_001730295	
Enterococcus rotai LMG 26678	Sedláček et al. 2013	LMG 26678; CCUG 61593; DSM 102982; CCM 4630	*Enterococcus rotai*	3583 054	36.1	3253	Gp0150894	PRJNA226735	SAMN04296136	GCA_001465345	
Enterococcus rivorum LMG 25899	Niemi et al. 2012	LMG 25899; DSM 104544; CCM 7986; HAMBI 3055; S299	*Enterococcus rivorum*	3806 353	34.9	3542		PRJNA224116	SAMN05420592	GCF_001742285	
Enterococcus ureilyticus LMG 26676	Sedláček et al. 2013	CCUG 48799; DSM 102981; CCM 4629	*Enterococcus ureilyticus*	3472 706	36.1	3240		PRJNA224116	SAMN05420589	GCF_001730315	
Enterococcus xiangfangensis NCIMB 14834	Li et al. 2014	11097; LMG 27495; NCIMB 14834; DSM 105127	*Enterococcus xiangfangensis*	2647 587	39.0	2576		PRJDB7793	SAMD00164314	GCA_005405365	
14EA1_(ST147).fna				2797 286	37.8	2639					
20_SD_W_06_(ST368).fna				2844 029	37.3	2711					
209EA1_(ST624).fna				2671 742	37.7	2455					
244_EFLS_(ST443).fna				2887 109	37.5	2816					
302EA1_(ST122).fna				2995 860	37.5	2784					

Strain	Authority	Other deposits	Synonyms	Base pairs	Percent G+C	No. proteins	Goldstamp	Bioproject accession	Biosample accession	Assembly accession	IMG OID
732_EFLS_(ST16).fna				2815769	37.4	2698					
4928STDY7071351_(ST30).fna				3062673	37.2	2993					
4928STDY7071435_(ST40).fna				3178436	37.7	3137					
15224_(ST16).fna				3360605	37.1	3599					
19910_(ST116).fna				3076955	37.3	2994					
BIOML_A5_(ST875).fna				2924887	37.3	2763					
BT00.E.21_(ST23).fasta				3020567	37.1	3252					
C116_(ST228).fna				2723129	37.6	2566					
C138_(ST228).fna				2695188	37.6	2571					
CL6870_(ST103).fasta				3084613	37.3	2998					
CL8682_(ST769).fasta				3082864	37.3	3035					
CL9172_(ST21).fasta				2873912	37.4	2713					
CL9314_(ST21).fasta				2892664	37.4	2757					
CL9772_(ST525).fasta				3169716	37.2	3185					
CL9797_(ST525).fasta				3193568	37.1	3218					
CL9824_(ST631).fasta				2644265	37.7	2436					
CL9924_(ST6).fasta				3257374	37.1	3236					
CL9943_(ST4).fasta				3289758	37.0	3325					

Strain	Authority	Other deposits	Synonyms	Base pairs	Percent G+C	No. proteins	Goldstamp	Bioproject accession	Biosample accession	Assembly accession	IMG OID
CL11199_(ST778).fasta				3250910	37.2	3276					
CVM N52662_(ST228).fna				2775983	37.5	2617					
CVM N53420_(ST228).fna				2866934	37.4	2727					
CVM_N52587_(STx).fna				2746314	37.5	2598					
CVM_N54595_(ST192).fna				3053384	37.4	2946					
CVM_N55265_(ST228).fna				2699907	37.6	2536					
CVM_N60027F_(ST862).fna				2782488	37.6	2584					
D32_(ST40).fna				3062505	37.4	2977					
DSM111623_(ST624).fna				2705646	37.5	2531					
E1_(ST40).fna				3105472	37.3	2958					
EF348_(ST16).fna				2980422	37.5	2854					
EF349_(ST631).fna				2858773	38.6	2727					
EN24_(ST228).fna				2763117	37.5	2602					
EN242_(ST82).fna				3157284	37.0	3026					
EN788_(ST82).fna				3185961	36.9	3072					
EnGen0400_(ST86).fna				2921810	37.7	2723					
F_(ST712).fna				3441804	37.6	3295					

Strain	Authority	Other deposits	Synonyms	Base pairs	Percent G+C	No. proteins	Goldstamp	Bioproject accession	Biosample accession	Assembly accession	IMG OID
F1_(ST72).fna				3086314	37.5	2938					
G81_(ST1468).fna				2835934	37.5	2732					
KUB3007_(ST729).fna				3171737	37.2	2988					
L14_(ST330).fna				2821743	37.6	2670					
LB00.E.122_(ST21).fasta				2961483	37.3	2834					
Merz96_(ST103).fna				3085251	37.6	2902					
P9 CL A7_(ST4).fna				3095653	37.0	2977					
Praia.S.M2.C4_(ST62).fasta				2953839	37.4	2867					
R48_(ST228).fna				2812753	37.6	2725					
T11_(ST65).fna				2748724	37.7	2536					

Methods, Results and References

The genome sequence data were uploaded to the Type (Strain) Genome Server (TYGS), a free bioinformatics platform available under https://tygs.dsmz.de, for a whole genome-based taxonomic analysis [1]. The analysis also made use of recently introduced methodological updates and features [2]. Information on nomenclature, synonymy and associated taxonomic literature was provided by TYGS's sister database, the List of Prokaryotic names with Standing in Nomenclature (LPSN, available at https://lpsn.dsmz.de) [2]. The results were provided by the TYGS on 2024-06-18. The TYGS analysis was subdivided into the following steps:

Determination of closely related type strains

Determination of closest type strain genomes was done in two complementary ways: First, all user genomes were compared against all type strain genomes available in the TYGS database via the MASH algorithm, a fast approximation of intergenomic relatedness [3], and, the ten type strains with the smallest MASH distances chosen per user genome. Second, an additional set of ten closely related type strains was determined via the 16S rDNA gene sequences. These were extracted from the user genomes using RNAmmer [4] and each sequence was subsequently BLASTed [5] against the 16S rDNA gene sequence of each of the currently 21253 type strains available in the TYGS database. This was used as a proxy to find the best 50 matching type strains (according to the bitscore) for each user genome and to subsequently calculate precise distances using the Genome BLAST Distance Phylogeny approach (GBDP) under the algorithm 'coverage' and distance formula d_5 [6]. These distances were finally used to determine the 10 closest type strain genomes for each of the user genomes.

Pairwise comparison of genome sequences

For the phylogenomic inference, all pairwise comparisons among the set of genomes were conducted using GBDP and accurate intergenomic distances inferred under the algorithm 'trimming' and distance formula d_5 [6]. 100 distance replicates were calculated each. Digital DDH values and confidence intervals were calculated using the recommended settings of the GGDC 4.0 [2,6].

Phylogenetic inference

The resulting intergenomic distances were used to infer a balanced minimum evolution tree with branch support via FASTME 2.1.6.1 including SPR postprocessing [7]. Branch support was inferred from 100 pseudo-bootstrap replicates each. The trees were rooted at the midpoint [8] and visualized with PhyD3 [9].

Type-based species and subspecies clustering

The type-based species clustering using a 70% dDDH radius around each of the 53 type strains was done as previously described [1]. The resulting groups are shown in Table 1 and 4. Subspecies clustering was done using a 79% dDDH threshold as previously introduced [10].

Results

Type-based species and subspecies clustering

The resulting species and subspecies clusters are listed in Table 4, whereas the taxonomic identification of the query strains is found in Table 1. Briefly, the clustering yielded 43 species clusters and the provided query strains were assigned to 1 of these. Moreover, user strains were located in 2 of 44 subspecies clusters.

Figure caption SSU tree

Figure 1. Tree inferred with FastME 2.1.6.1 [7] from GBDP distances calculated from 16S rDNA gene sequences. The branch lengths are scaled in terms of GBDP distance formula d_5. The numbers above branches are GBDP pseudo-bootstrap support values > 60 % from 100 replications, with an average branch support of 39.0 %. The tree was rooted at the midpoint [8].

Figure caption genome tree

Figure 2. Tree inferred with FastME 2.1.6.1 [7] from GBDP distances calculated from genome sequences. The branch lengths are scaled in terms of GBDP distance formula d_5. The numbers above branches are GBDP pseudo-bootstrap support values > 60 % from 100 replications, with an average branch support of 29.7 %. The tree was rooted at the midpoint [8].

References

[1] Meier-Kolthoff JP, Göker M. TYGS is an automated high-throughput platform for state-of-the-art genome-based taxonomy. Nat. Commun. 2019;10: 2182. DOI: 10.1038/s41467-019-10210-3

[2] Meier-Kolthoff JP, Sardà Carbasse J, Peinado-Olarte RL, Göker M. TYGS and LPSN: a database tandem for fast and reliable genome-based classification and nomenclature of prokaryotes. Nucleic Acid Res. 2022;50: D801–D807. DOI: 10.1093/nar/gkab902

[3] Ondov BD, Treangen TJ, Melsted P, et al. Mash: Fast genome and metagenome distance estimation using MinHash. Genome Biol 2016;17: 1–14. DOI: 10.1186/s13059-016-0997-x

[4] Lagesen K, Hallin P. RNAmmer: consistent and rapid annotation of ribosomal RNA genes. Nucleic Acids Res. Oxford Univ Press; 2007;35: 3100–3108. DOI: 10.1093/nar/gkm160

[5] Camacho C, Coulouris G, Avagyan V, Ma N, Papadopoulos J, Bealer K, et al. BLAST+: architecture and applications. BMC Bioinformatics. 2009;10: 421. DOI: 10.1186/1471-2105-10-421

[6] Meier-Kolthoff JP, Auch AF, Klenk H-P, Göker M. Genome sequence-based species delimitation with confidence intervals and improved distance functions. BMC Bioinformatics. 2013;14: 60. DOI: 10.1186/1471-2105-14-60

[7] Lefort V, Desper R, Gascuel O. FastME 2.0: A comprehensive, accurate, and fast distance-based phylogeny inference program. Mol Biol Evol. 2015;32: 2798–2800. DOI: 10.1093/molbev/msv150

[8] Farris JS. Estimating phylogenetic trees from distance matrices. Am Nat. 1972;106: 645–667.

[9] Kreft L, Botzki A, Coppens F, Vandepoele K, Van Bel M. PhyD3: A phylogenetic tree viewer with extended phyloXML support for functional genomics data visualization. Bioinformatics. 2017;33: 2946–2947. DOI: 10.1093/bioinformatics/btx324

[10] Meier-Kolthoff JP, Hahnke RL, Petersen J, Scheuner C, Michael V, Fiebig A, et al. Complete genome sequence of DSM 30083^T, the type strain (U5/41^T) of *Escherichia coli*, and a proposal for delineating subspecies in microbial taxonomy. Stand Genomic Sci. 2014;9: 2. DOI: 10.1186/1944-3277-9-2
