# Supplementary material for: Hidden diversity in Enterococcus faecalis revealed by CRISPR2 screening: eco-evolutionary insights into a novel subspecies
Source: Microbiol Spectr. 2025 Sep 8;13(10):e01428-25. doi: 10.1128/spectrum.01428-25 (PMC12502612; doi:10.1128/spectrum.01428-25)
Supplement: Supplemental figures — Fig. S1 and S2. [file spectrum.01428-25-s0008.pdf]

Supplementary Figures:

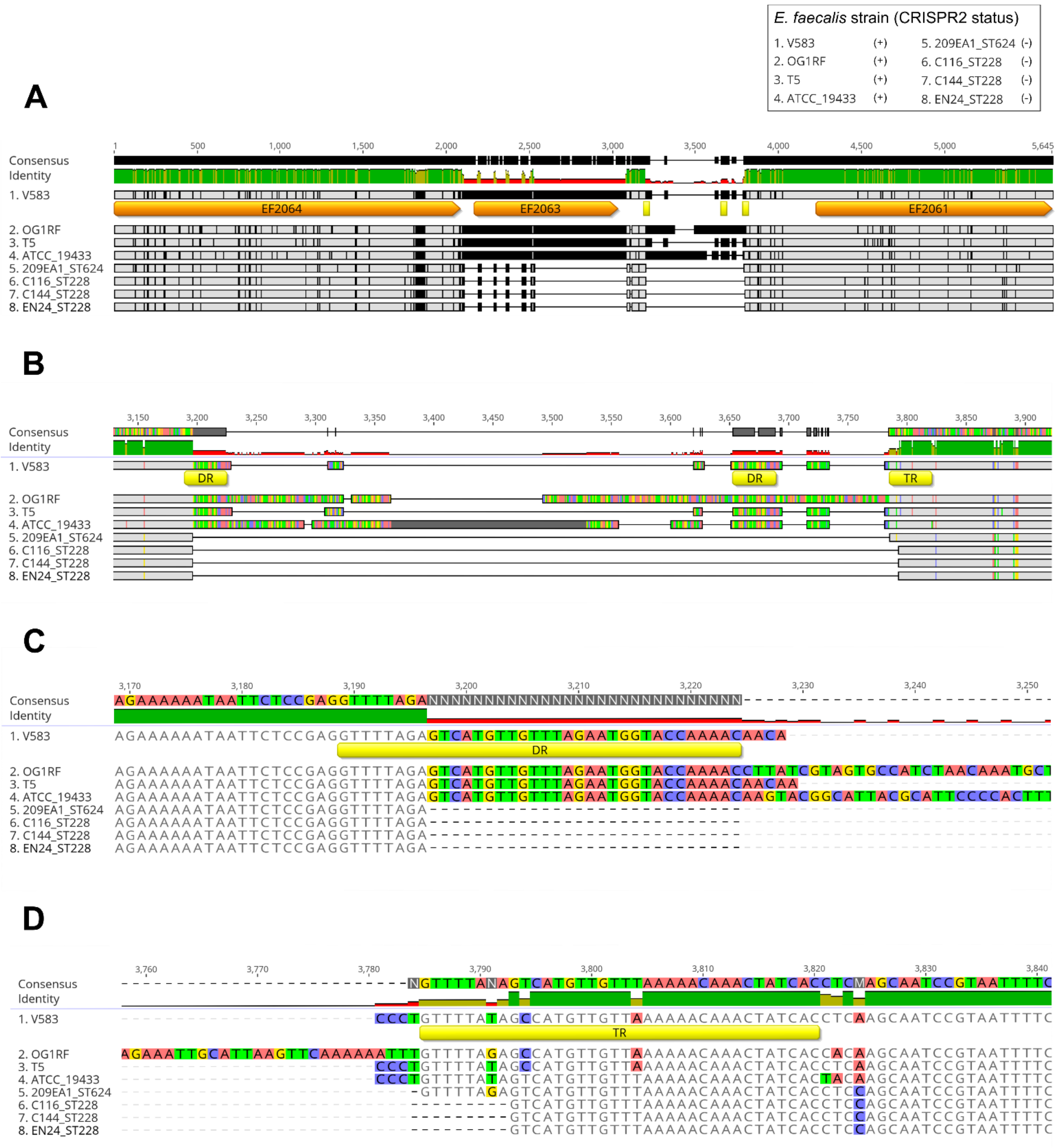

Fig. S1 Genomic alignment of the CRISPR2 region across representative *Enterococcus faecalis* strains. (**A**) Overview of the chromosomal region surrounding the CRISPR2 locus, including flanking ORFs EF2064 and EF2061. (**B**) Zoom into the intergenic region corresponding to the CRISPR2 array (or its absence). (**C**) Nucleotide alignment of the leader-proximal direct repeat (DR) of the CRISPR2 array. (**D**) Nucleotide alignment of the terminal repeat (TR) of the CRISPR2 array. The V583 genome was used as a reference for ORF (orange arrows) and CRISPR2 repeat (yellow blocks) annotations. Alignments were performed with MAFFT v7.515 and visualized in Geneious Prime. In all panels, the “Identity” track represents the mean pairwise identity across sequences at each alignment column (green: 100% identity; green-brown: ≥30% and <100% identity; red: <30% identity). Nucleotide-level comparisons highlight identical bases in light gray and variable sites in black. Undefined bases ('N') are shown in dark gray.

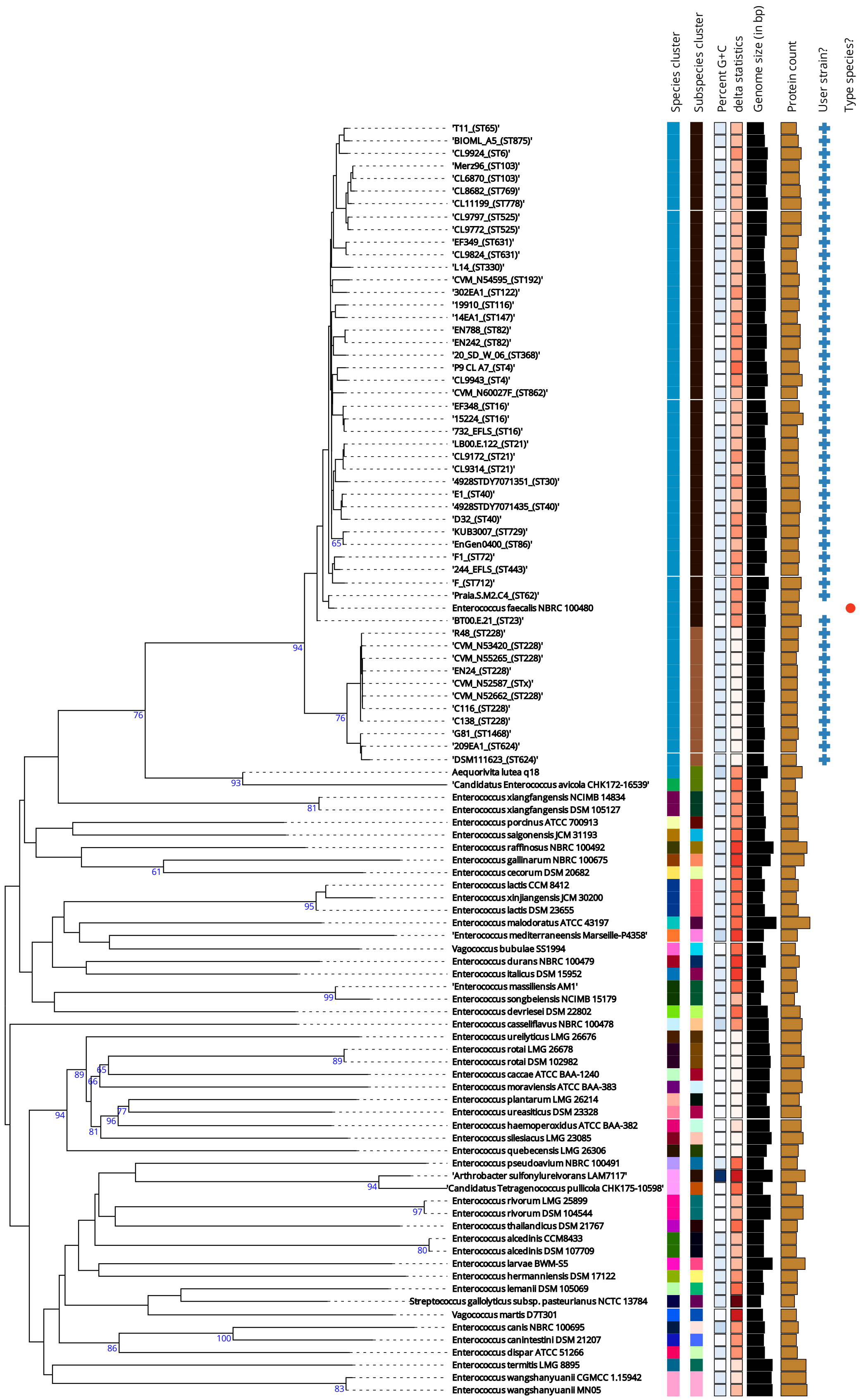

Fig. S2 The 39 subspecies A genomes cluster with the *Enterococcus faecalis* type strain, separate from subspecies B. The tree was inferred using FastME 2.1.6.1 from GBDP distances calculated from genome sequences. Branch lengths are scaled according to the GBDP distance formula d5. The numbers above branches represent GBDP pseudo-bootstrap support values > 60% from 100 replications. The tree was midpoint-rooted. Taxonomic assignments at the species and subspecies levels are color-coded according to dDDH values, using the established thresholds of ≥70% for species and ≥79% for subspecies delineation. As highlighted, the 39 genomes analyzed as subspecies A cluster with the *E. faecalis* type strain and form a clade distinct from the CRISPR2-negative representatives of subspecies B. Sequence types (STs) of each strain are shown in parentheses.
